# Trends and disparities of disease burden in infections among pregnant women in 131 low-income and middle-income countries, 1990–2019

**DOI:** 10.7189/jogh.14.04130

**Published:** 2024-09-06

**Authors:** Chenyuan Qin, Min Liu, Jue Liu

**Affiliations:** 1 School of Public Health, Peking University, Beijing, China; 2 Institute for Global Health and Development, Peking University, Beijing, China; 3 National Health Commission Key Laboratory of Reproductive Health, Peking University, Beijing, China; 4 Peking University Health Science Center-Weifang Joint Research Center for Maternal and Child Health, Peking University, Beijing, China

## Abstract

**Background:**

In low- and middle-income countries (LMICs) and territories, maternal infections impose a non-negligible disease burden. We aimed to analyse the secular trends, age distribution, and associated factors of maternal sepsis and other maternal infections (MSMI) across 131 LMICs from 1990 to 2019.

**Methods:**

We collected yearly data of incidences, deaths, and disability adjusted life years (DALYs) on MSMI in 131 LMICs from 1990 to 2019 from the Global Burden of Disease 2019 (GBD 2019). The sociodemographic index (SDI) and universal health coverage effective coverage index (UHCI) were also acquired. Relative percent change and estimated annual percentage change (EAPC) were used to assess the secular trends. Correlation analyses were also employed to explore the associations between the burden of MSMI with SDI and UHCI.

**Results:**

Between 1990 and 2019, the age-standardised incident rates (ASIRs), age-standardised maternal mortality ratios (ASMMRs) and age-standardised DALYs rates of low-income countries (LICs) were much higher than that of lower-middle-income countries (LMCs) and upper-middle income countries (UMCs), although they all continued to decline. At least six of 131 LMICs had ASMMR greater than 70.00 per 100 000 live births in 2019. The incidences of MSMI increased first till 20–24 years and then decreased with age both in 1990 and 2019, while the ASMMRs were higher in the youngest and the oldest age group. With the growth of SDI and UHCI in 2109, the decreasing trend of ASIR, ASMMR, and age-standardised DALYs rates slowed down.

**Conclusions:**

Although the progress has been made in reducing the burden of MSMI in 131 LMICs, the disease burden in LICs far exceeded that of LMCs and UMCs. Socio-economic status and universal health coverage were both associated with the MSMI burden, and further research is needed to explore the underlying factors contributing to these disparities and to identify effective strategies for reducing the burden of MSMI.

Maternal infections encompass a wide range of conditions, such as urinary tract infections, chorioamnionitis, cervicitis, and postpartum uterine infections, which may lead to severe complications, including sepsis, multi-organ failure, and even death [1]. Maternal sepsis is defined as a life-threatening condition characterised by organ dysfunction resulting from infection during pregnancy, childbirth, postpartum, and post-abortion [2,3]. Actually, 99% of maternal deaths occurred in developing countries, and the majority are preventable [4–6]. Low- and middle-income countries and territories (LMICs) still face challenges in the prevention and management of maternal infections, where limited resources and access to care exacerbate the risk of poor pregnancy outcomes [1,7–10]. A systematic analysis by the World Health Organization (WHO) indicated that direct obstetric infection is one of the leading causes of maternal mortality, accounting for approximately 10.7% of maternal deaths [7]. The highest mortality rates, at 10.7%, were observed in LMICs, compared to 4.7% in high-income countries (HICs) [7]. The Global Maternal Sepsis Study (GLOSS), conducted in 52 LMICs and HICs, estimated that approximately 70 women per 1000 livebirths had maternal infections requiring hospital management, and 10.9 women per 1000 livebirths experienced severe maternal outcomes related to infection (either as an underlying or contributing cause) [8]. The highest intrahospital fatality proportion among women with severe maternal outcomes and maternal mortality ratios (MMRs) were observed in LMICs [8]. Additionally, the placenta serves as an active immune site, and the tropism of specific pathogens for the placenta can influence susceptibility and severity of certain infectious diseases during pregnancy, poor pregnancy outcomes, as well as long-term neurodevelopmental or other sequelae in the offspring [1,11,12].

Efforts to mitigate the prevalence and impact of maternal sepsis have been accorded high priority [13]. Millennium Development Goals (MDGs) sought to attain a substantial reduction of 75% in MMR between 1990 and 2015 [14,15]. In alignment with the Sustainable Development Goals (SDGs), all nations are now targeted to achieve MMR below 70 per 100 000 livebirths by 2030 [4]. The WHO, alongside its collaborative partners, launched the Global Maternal Sepsis and Neonatal Initiative in 2016 [9]. This pivotal undertaking paved the way for the implementation of the GLOSS in 2017, with the overarching aim of alleviating the burden associated with maternal sepsis and other infections (MSMI) [9]. A study conducted in 2018 investigated the availability of facility resources in LMICs and revealed the existing limitations in management [16]. As the epoch of SDGs commences, the significance of preventing maternal infection-related morbidity and mortality must be elevated to the vanguard of developmental priorities [4,14]. Advanced technology and medicines have been able to quickly reduce the incidence of infections, including maternal infections, which also happen in LMICs [4,9]. Indicators, such as incident rate, MMR, and disability-adjusted life years (DALYs) of MSMI, not only reflect the direct health status of pregnant women but also serve as proxies for assessing the overall effectiveness of health systems and the penetration of maternal care services. In the context of the SDGs, these indicators offer vital benchmarks to evaluate progress towards improving maternal health. However, there is a lack of studies that comprehensively describe the trends and disparities of disease burden of maternal infections at the overall, regional, and national levels in LMICs.

Hence, in this study, we aim to provide epidemiological characteristics, secular trends, and age distribution of MSMI in 131 LMICs from 1990 to 2019. Additionally, we also qualitatively analyse their association with sociodemographic status and universal health coverage. Our study may empower policymakers and health care providers with actionable insights to prioritise resource allocation, enhance care delivery, and implement preventative measures that could substantially curb the incidence and impact of maternal infections in LMICs.

## METHODS

### Overview

The Global Burden of Disease (GBD) database, a publicly available database coordinated by the Institute for Health Metrics and Evaluation, provides a comprehensive assessment of the epidemiological levels and trends for 369 diseases and injuries across 204 countries and territories from 1990 to 2019 [17]. The estimation of disease burden utilises standardised tools and methodologies, which have been detailed in previous studies and supplementary files [17,18]. Specifically, DisMod-MR version 2.1, a meta-analysis tool employing a compartmental model structure, is used to model nonfatal disease burden by synthesising sparse and heterogeneous epidemiologic data. Mortality estimates are obtained through a standardised pooling process of available data on causes of death or incidence, which are then utilised in various models such as cause of death ensemble modelling, disease model-Bayesian meta-regression, and comorbidity correction to ensure comparability across different diseases globally.

### Study design and data sources

We collected yearly data on maternal sepsis and other maternal infections (MSMI) in LMICs from 1990 to 2019 at country levels from the extensively utilising GBD 2019 query tool [17]. The World Bank Group assigns the world’s economies to four income groups, namely low, lower-middle, upper-middle, and high [19], and we ultimately identified 131 LMICSs Based on the World Bank Group country classifications in 2019 in this study (Table S1 in the **Online Supplementary Document**) [20]. The sociodemographic index (SDI) and universal health coverage effective coverage index (UHCI) were also acquired from the GBD 2019 [21,22].

### Maternal sepsis and other maternal infections

Maternal sepsis and other maternal infections (MSMI) encompass two distinct components [17]. Maternal sepsis is characterised by deviations in body temperature (<36°C or >38°C) and clinical indicators of shock including systolic blood pressure (<90 mm of mercury pressure (mmHg)) and tachycardia (>120 beats per minute (bpm)). Other maternal infections refer to any non-HIV or non-sexually transmitted infections that are not considered to have an epidemiological association with pregnancy. This category includes conditions such as urinary tract infections, mastitis, candidiasis, and bacterial vaginosis during pregnancy.

For this study, we retrieved annual data on its incident cases, incident rate (per 100 000 population), deaths, MMR and, DALYs (per 100 000 population) between 1990 and 2019. MMR is defined as the number of maternal deaths per 100 000 livebirths. The DALYs, a composite metric encompassing years lived with disability and years of life lost, serves as a comprehensive indicator of population health [23]. In addition, we also arranged the data into successive five-year age intervals as 10–14 years, 15–19 years, 20–24 years, 25–29 years, 30–34 years, 35–39, 40–44, 45–49 years, and 50–54 years. To ascertain the uncertainty surrounding each metric, 95% uncertainty intervals (UIs) based on the 25th and 975th ordered values were derived from 1000 draws of the posterior distribution.

### Sociodemographic index and UHCI

The SDI is a composite indicator of development status that is highly correlated with health outcomes, calculated by taking the geometric mean of three indices: total fertility rate under the age of 25, mean education for those ages 15 and older, and lag distributed income per capita [22,24]. The SDI indicates a theoretical minimum level of development relevant to health at 0 and a theoretical maximum level at 1 [22]. An index that estimates global progress towards universal health coverage and specifically UHC effective coverage varying from 0 to 100 has been created using estimates from the GBD 2019 [25]. The UHCI is comprised of 23 indicators drawn across a range of health service areas, involving either direct measures of intervention coverage (e.g. antiretroviral therapy coverage) or outcome-based indicators (e.g. mortality-to-incidence ratios), and is meant to represent average coverage of primary health care services [21,25]. Tables S2–3 in the **Online Supplementary Document** display the SDIs and UHCIs of 131 LMICs in 2019.

### Statistical analysis

The absolute incidences, deaths, and DALYs served as robust indicators of the prevailing status of MSMI in individual nations and regions, with their relative percent change calculated by the formula as (Numbers 2019 – Numbers 1990)/Numbers 1990 × 100%. To compare between countries and regions over time in which the age profiles change accordingly, the GBD group applied the age-specific rates for each location, sex, and year to a GBD world standard age structure, presented as the age-standardised incident rates (ASIRs), age-standardised MMR (ASMMR) and age-standardised DALYs rates per 100 000 population or per 100 000 livebirths [26,27]. The estimated annual percentage change (EAPC) was determined through a regression model, Y = α + βX + ε, where Y represents the natural logarithm of the rate (e.g. ASIR, ASMMR, or age-standardised DALYs rate), X denotes the calendar year, ε signifies the error term, and β signifies the directional trend [28]. The EAPC was derived as 100 × (exp(β) - 1). Notably, a rising trend was indicated by both a positive EAPC estimation and a positive lower boundary of 95% UI, while a declining trajectory was confirmed by negative values for both the EAPC and its upper boundary of 95% UI [28]. Conversely, stability in ASIR was inferred when neither of these conditions were met. Spearman and Pearson correlation analyses and the correlation coefficients (ρ) were employed to explore the associations between the burden of MSMI with SDI and UHCI in LMICs. Furthermore, polynomial curves were also utilised to model the observed trends. All statistical analyses were performed by R version 4.2.2, and statistical significance was attributed to a two-sided *P*-value less than 0.05.

## RESULTS

### Global and regional trend of MSMI

**Figure 1:**
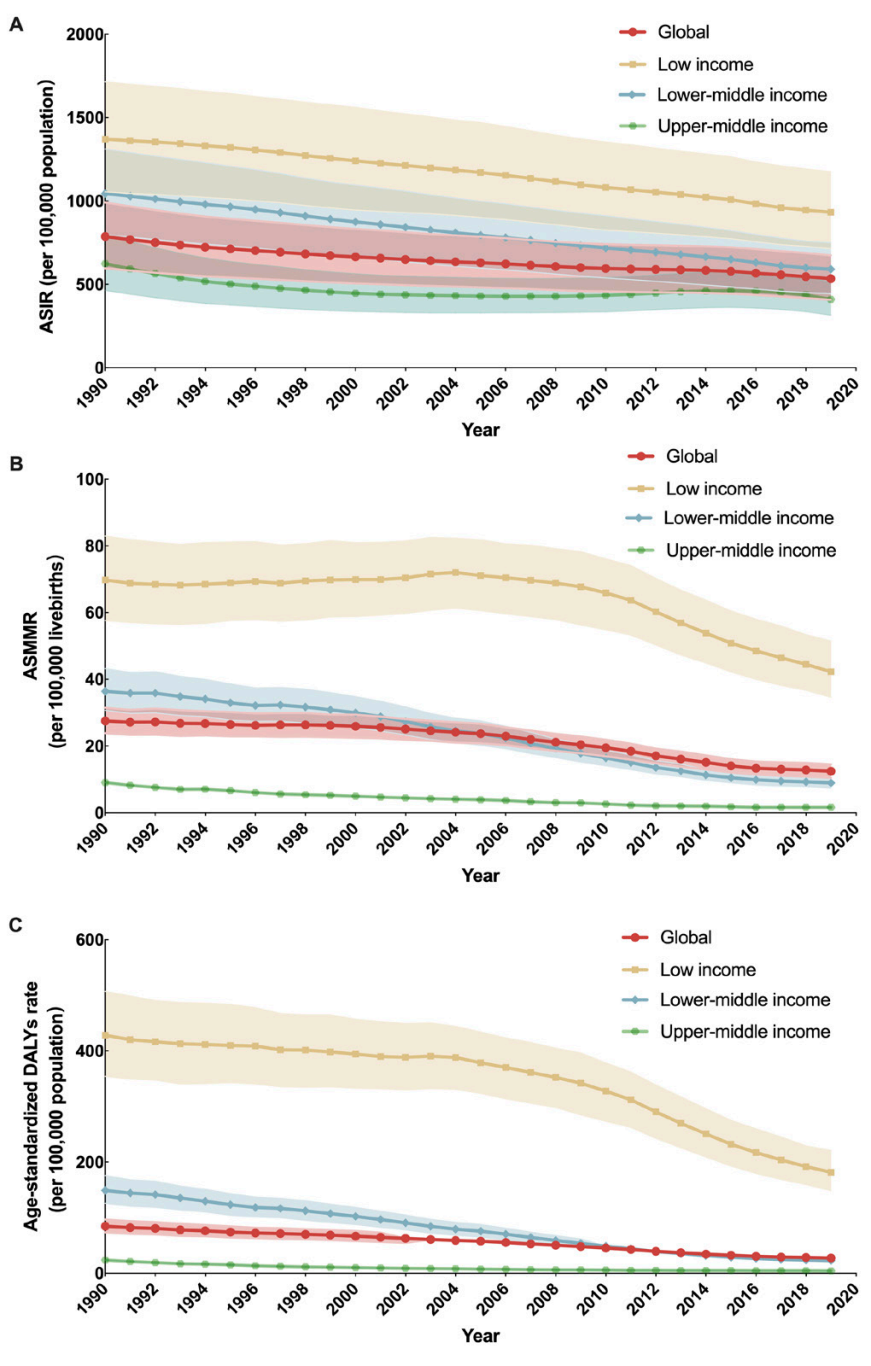
ASIR per 100 000 population (**Panel A**), ASMMR per 100 000 livebirths (**Panel B**), and age-standardised DALY rates per 100 000 population (**Panel C**) for MSMI globally and in LIMCs over 30 years, 1990–2019. ASIR – age-standardised incidence rate, ASMMR – age-standardised maternal mortality ratio, DALYs – disability-adjusted life years, LIMCs – low- and middle-income countries and territories, MSMI – maternal sepsis and other maternal infections.

Globally, the incident case number of MSMI decreased by −10.68% (95% CI = −14.28, −5.76) from 23.03 million in 1990 to 20.57 million in 2019, and the death number reduced from 38 027 in 1990 to 16 840 in 2019 with a relative percent change of −55.72% (95% CI = −63.78, −47.52) (**Table 1**). The total ASIR decreased from 787.04 in 1990 to 534.21 in 2019 per 100 000 population, with an EAPC of −1.17% (**Table 2****,** **Figure 1**, panel A). The ASMMR was steadily declining from 27.53 in 1990 to 12.44 per 100 000 livebirths in 2019 (EAPC = −2.83%) (**Table 2****,** **Figure 1**, panel B). In 1990, there were a total of 2.37 million DALYs (or 84.83 DALYs per 100 000 population), while it changed to 1.07 million DALYs (or 27.34 DALYs per 100 000 population) in 2019 (**Table 2****,** **Figure 1**, panel C).

**Table 1:** Incident cases, deaths, and DALYs of MSMI in 131 low- and middle-income countries and territories and their relative percent change from 1990 to 2019

Location	Incident cases			Deaths			DALYs		
	**1990, No. ×10^5^ (95% UI)**	**2019, No. ×10^5^ (95% UI)**	**Relative percent change, % (95% CI)**	**1990, No. ×10^3^ (95% UI)**	**2019, No. ×10^3^ (95% UI)**	**Relative percent change, % (95% CI)**	**1990, No. ×10^5^ (95% UI)**	**2019, No. ×10^5^ (95% UI)**	**Relative percent change, % (95% CI)**
Global	230.29 (174.00, 290.85)	205.70 (156.89, 259.72)	−10.68 (−14.28, −5.76)	38.0 (33.01, 43.51)	16.84 (14.23, 19.63)	−55.72 (−63.78, −47.52)	23.70 (20.57, 27.11)	10.65 (8.96, 12.56)	−55.09 (−63.27, −47.24)
LICs	22.95 (17.45, 29.02)	35.34 (26.69, 45.00)	53.97 (48.14, 60.03)	10.78 (8.91, 12.82)	10.41 (8.53, 12.67)	−3.44 (−24.42, 17.04)	6.28 (5.22, 7.43)	6.13 (5.01, 7.44)	−2.37 (−23.06, 18.07)
LMCs	105.98 (79.805, 134.17)	103.14 (77.38, 130.81)	−2.68 (−6.15, 1.41)	22.92 (19.30, 27.19)	5.83 (4.83, 6.98)	−74.57 (−79.99, −68.68)	14.42 (12.14, 17.09)	3.89 (3.21, 4.67)	−73.03 (−78.33, −66.89)
UMCs	77.47 (57.13, 99.62)	50.82 (39.08, 64.07)	−34.41 (−40.92, −26.62)	4.15 (3.71, 4.68)	0.55 (0.48, 0.62)	−86.75 (−88.77, −84.36)	2.80 (2.46, 3.27)	0.53 (0.41, 0.71)	−81.04 (−84.67, −76.68)

CI – confidence interval, DALYs – disability-adjusted life years, LICs – low-income countries and territories, LMCs – lower-middle-income countries and territories, MSMI – maternal sepsis and other maternal infections, UI – uncertain interval, UMCs – upper-middle-income countries and territories.

**Table 2:** ASIR, ASMMR, and age-standardised DALYs of MSMI in 131 low- and middle-income countries and territories with EAPC from 1990 to 2019

Location	ASIR*			ASMMR†			Age-standardised DALYs rate‡		
**1990 (95% UI)**	**2019 (95% UI)**	**EAPC, % (95% CI)**	**1990 (95% UI)**	**2019 (95% UI)**	**EAPC, % (95% CI)**	**1990 (95% UI)**	**2019 (95% UI)**	**EAPC, % (95% CI)**
Global	787.04 (595.71, 990.76)	534.21 (407.34, 673.73)	−1.17% (−1.23, −1.11)	27.53 (23.90, 31.49)	12.44 (10.51, 14.51)	−2.83% (−3.22, −2.43)	84.83 (73.76, 96.71)	27.34 (22.97, 32.3)	−3.89% (−4.18, −3.59)
LICs	1370.98 (1054.65, 1714.58)	932.14 (713.57, 1175.26)	−1.34% (−1.39, −1.29)	69.78 (57.65, 82.95)	42.31 (34.65, 51.48)	−1.38% (−1.79, −0.96)	428.22 (354.29, 507.11)	181.56 (148.64, 220.94)	−2.63% (−3.1, −2.16)
LMCs	1044.71 (797.69, 1312.25)	590.93 (444.80, 748.91)	−1.96% (−1.99, −1.92)	36.43 (30.67, 43.21)	8.94 (7.41, 10.71)	−5.18% (−5.64, −4.73)	149.04 (125.94, 176.01)	22.8 (18.91, 27.26)	−6.68% (−7.12, −6.24)
UMCs	623.74 (462.87, 798.70)	410.35 (314.31, 514.42)	−0.83% (−1.13, −0.54)	9.07 (8.11, 10.24)	1.65 (1.44, 1.87)	−6.02% (−6.24, −5.79)	23.88 (21.00, 27.68)	4.21 (3.22, 5.68)	−5.70% (−6.04, −5.35)

ASIR – age-standardised incidence rate, ASMMR – age-standardised maternal mortality ratio, CI – confidence interval, DALYs – disability-adjusted life years, EAPC – estimated annual percentage change, LICs – low-income countries and territories, LMCs – lower-middle-income countries (and territories), MSMI – maternal sepsis and other maternal infections, UI – uncertain interval, UMCs – upper-middle-income countries (and territories)

*Per 100 000 population.

†Per 100 000 livebirths.

‡Per 100 000 population.

**Table 1** also presents the absolute numbers of the disease burden of MSMI in regions. It is worth noting that only the number of incident cases in LICs increased with a relative percentage change of more than 50%. The ASIR in 2019 was highest in LICs (932.14 per 100 000 population), followed by LMCs (590.93) and UMCs (410.35) (**Table 2**). The ASIR, ASMMR, and age-standardised DALYs rates continued to decline between 1990 and 2019 in all regions, with the EAPC of ASMMR and age-standardised DALYs rates even decreased by more than 5% in both LMCs and UMCs (**Table 2****,** **Figure 1**, panels A–C). From 1990 to 2019, the ASMMR of LICs has been much higher than that of LMCs and UMCs, while it dropped significantly since the year 2010. In addition, the ASMMR of LMCs changed from higher than the global average to a lower one around the year 2005 (**Figure 1**, panel B).

### National trend of MSMI

**Figure 2:**
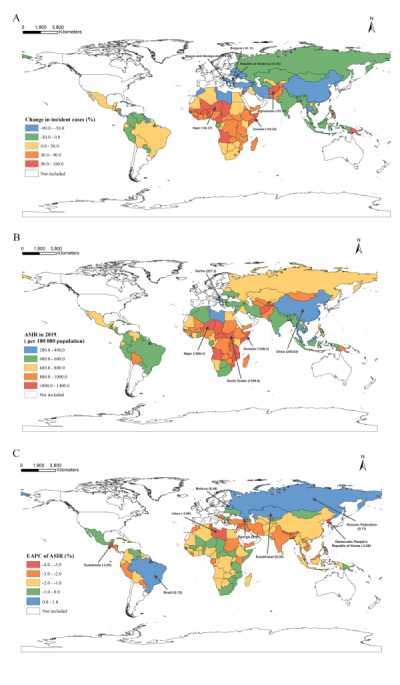
National trends in the incidence of MSMI in 131 LIMCs. The percentage of relative percent change in incident cases of MSMI between 1990 and 2019 (**Panel A**), age-standardised incidence rates (ASIRs) of MSMI in 2019 (**Panel B**), and estimated annual percentage changes (EAPCs) of ASIRs of MSMI from 1990 to 2019 (**Panel C**) are reported. LIMCs – low- and middle-income countries (and territories), MSMI – maternal sepsis and other maternal infections.

At the national level in 2019, India and China had the highest absolute number of incident cases (3.76 million, 95% UI = 2.75, 4.86; 1.60 million, 95% UI = 1.17, 2.12), while Democratic Republic of the Congo and India had the highest absolute number of deaths (2770, 95% UI = 2026, 3675; 1919, 95% UI = 1344, 2641) and DALYs (0.16 million, 95% UI = 1.17, 2.09; 0.13 million, 95% UI = 0.10, 0.18) (Tables S4–6 in the **Online Supplementary Document**). The country with the most pronounced increase in incident cases of MSMI was Afghanistan (167.81%), followed by Niger (162.27%) and Somalia (153.53%), whereas the incident case number of 12 nations, such as Saint Lucia and Bosnia and Herzegovina, all decreased by more than 50% in 2019 (**Figure 2**, panel A, Table S4 in the **Online Supplementary Document**). The largest increase in deaths of MSMI was observed in Somalia (167.69%), followed by Democratic Republic of the Congo (157.43%), while the most pronounced decrease was detected in Bosnia and Herzegovina (−99.80%) and China (−98.84%) (Table S5, Figure S1 in the **Online Supplementary Document**). Among the 131 LMICs, Chad (93.96%) and El Salvador (93.90%) had the highest fall-off in DALYs, while Somalia had the largest increase (172.10%), followed by the Democratic Republic of the Congo and Central African Republic (Table S6, Figure S2 in the **Online Supplementary Document**).

The epidemic state of MSMI varied significantly across 131 LMICs in 2019. Somalia showed the highest estimated national-level ASIR (1328.40 per 100 000 population, 95% UI = 1006.01, 1679.61) and age-standardised DALYs rate (656.45 per 100 000 population, 95% UI = 433.86, 934.27), while Serbia and China were on the contrast (**Figure 2**, panel B, Figure S2, Tables S4 and S6 in the **Online Supplementary Document**). At least six countries and territories had ASMMR greater than 70.00 per 100 000 livebirths in 2019. In the Central African Republic, every 100 000 livebirths caused more than 120 maternal lives lost, with the highest ASMMR of the 131 LIMCs. At least 26 LMICs had ASMMR less than 1.00 per 100 000 livebirths, including Montenegro, Bosnia and Herzegovina, North Macedonia, Viet Nam, and China (Table S5, Figure S1 in the **Online Supplementary Document**).

Of the 131 LMICs, the negative EAPCs of ASIR were observed in almost all countries and territories, ranging from −0.06% in Cuba to −3.88% in Libya, except Georgia (EAPC = 0.91%, 95% CI = 0.40, 1.41), Brazil (EAPC = 0.72%, 95% CI = 0.45, 0.99), Russian Federation (EAPC = 0.71%, 95% CI = 0.17, 1.25), Belarus (EAPC = 0.48%, 95% CI = 0.01, 0.95) and Kazakhstan (EAPC = 0.04%, 95% CI = −0.34, 0.42) (**Figure 2**, panel C, Table S4 in the **Online Supplementary Document**). As for the EAPCs of ASMMR, 13 countries, such as Zimbabwe and Central African Republic, showed an overall upward trend between 1990 and 2019 (all EAPCs and 95% CIs >0), 10 countries, such as Albania and the Marshall Islands, remained stable, while the other 108 LMICs declined steadily over the 30-year period (all EAPCs and 95% CIs <0). The ASMMR of Zimbabwe (EAPC = 5.21%) showed the most significant increase in the estimated annual change, while China (EAPC = −13.70%) and Bosnia and Herzegovina (EAPC = −21.25%) showed the fastest decline (Table S5, Figure S1 in the **Online Supplementary Document**). In addition, the estimated annual percentage change differed in age-standardised DALYs rates between 1990 and 2019, from the highest in Zimbabwe (EAPC = 4.02%) to the lowest in the Lao People’s Democratic Republic (EAPC = −10.05%), Nepal (EAPC = −10.10%), and Equatorial Guinea (EAPC = −12.56%). Only Zimbabwe, Georgia, Democratic Republic of the Congo, and Kazakhstan showed an overall upward trend between 1990 and 2019 (all EAPCs and 95% UIs >0) (Table S6, Figure S2 in the **Online Supplementary Document**).

### Age-specific distribution of MSMI in LMICs

**Figure 3:**
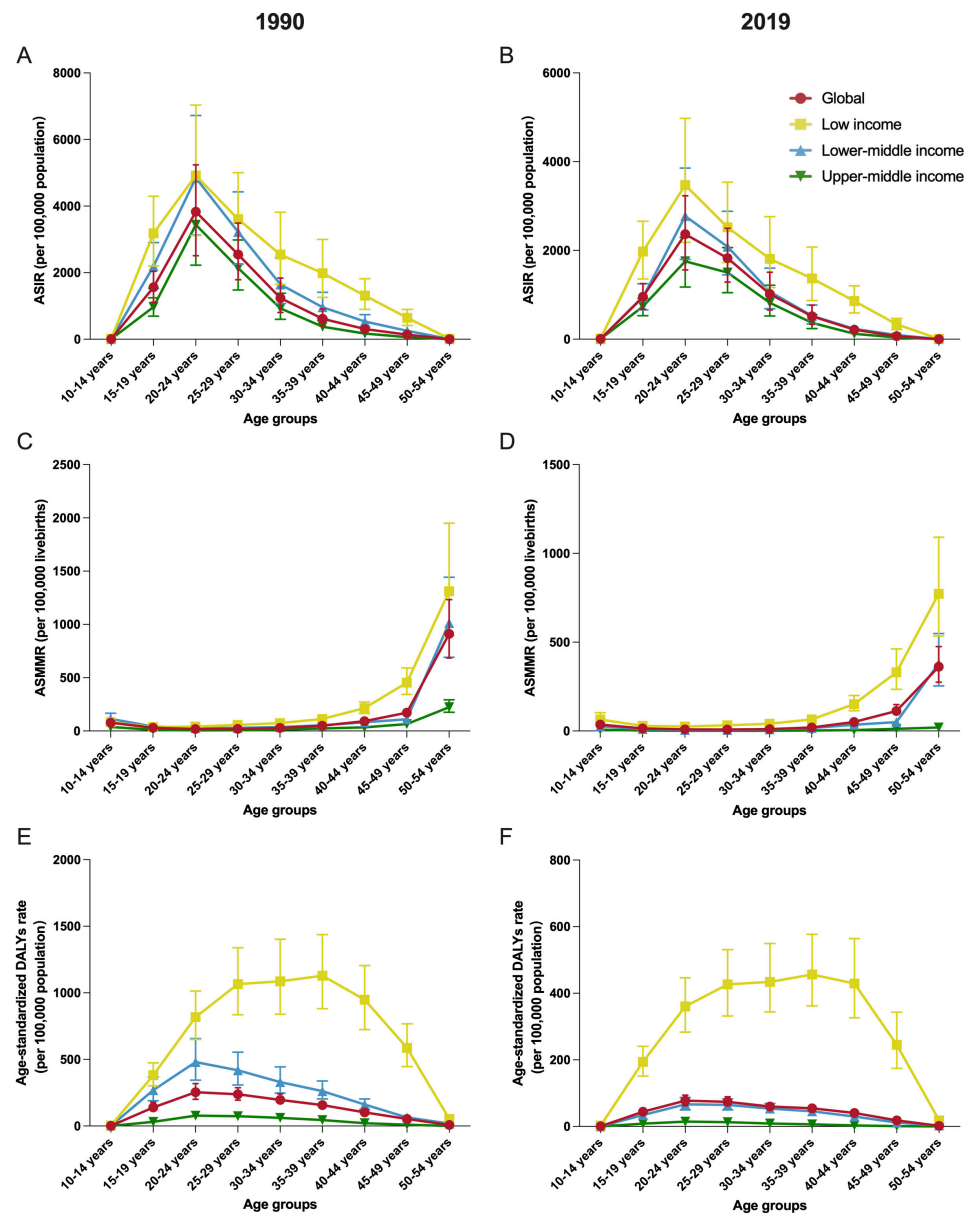
ASIR per 100 000 population, ASMMR per 100 000 livebirths, and age-standardised DALY rates per 100 000 population for MSMI among different age groups globally and in LIMCs in 1990 **(Panel A, C, and E)** and in 2019 (**Panel B, D, and F**). ASIR – age-standardised incidence rate, ASMMR – age-standardised maternal mortality ratio, DALYs – disability-adjusted life years, LIMCs – low- and middle-income countries (and territories), MSMI – maternal sepsis and other maternal infections.

The global incidences of MSMI consistently increased first and then decreased with age both in 1990 and 2019, with ASIR peaking in the 20–24 years (**Table 3**, **Figure 3**, panels A–B). The age distribution of global ASMMR was the same as that in different low- and middle-income regions: relatively higher in the youngest age group and remarkedly higher in the oldest age group, in 1990 and 2019 (**Table 4**, **Figure 3**, panels C–D). As for the age-standardised DALYs rates, the age distribution of global, LMCs and UMCs was the same as that of ASIR, with the highest rates in the 20–24 years, while there was a clear inverted U shape with the peak between 30–39 years (**Table 5**, **Figure 3**, panels E–F). Notably, the ASIR, ASMMR and age-standardised DALYs rates of almost all age groups in LICs were significantly higher than the global average, LMCs or UMCs. The disease burden of incidence, death and DALYs of all age groups in 131 LMICs are shown in Tables S7–33 in the **Online Supplementary Document**.

**Table 3:** Incidence of MSMI in 131 low- and middle-income countries and territories with EAPC in different age groups from 1990 and 2019

Location	Age group, in years	Incident cases in 1990 (95% UI)	Incident cases in 2019 (95% UI)	Relative change, % (95% CI)	ASIR in 1990 (95% UI)	ASIR in 2019 (95% UI)	EAPC, % (95% CI)
Global	10–14	57 887 (41 262, 72 280)	62 939 (44 439, 79 300)	8.73% (5.98, 11.06)	6.78 (4.84, 8.47)	6.64 (4.69, 8.36)	-0.07% (-0.14, 0.00)
LICs	10–14	12 629 (8741, 16 132)	21 709 (14 838, 28 020)	71.91% (64.21, 78.25)	16.84 (11.65, 21.51)	14.71 (10.06, 18.99)	-0.49% (-0.5, -0.47)
LMCs	10–14	25 306 (17 781, 32 010)	26 450 (18 430, 33 737)	4.52% (1.91, 7.54)	6.84 (4.8, 8.65)	5.68 (3.96, 7.25)	-0.83% (-0.91, -0.76)
UMCs	10–14	15 991 (11 711, 19 629)	12 210 (8854, 14 848)	−23.64% (−26.43, −20.77)	5.24 (3.84, 6.43)	5.09 (3.69, 6.19)	0.32% (0.07, 0.58)
Global	15–19	3 988 690 (2 833 027, 5 210 443)	2 887 793 (2 034 645, 3 779 060)	−27.60% (−28.77, −26.2)	1560.96 (1108.69, 2039.08)	956.99 (674.26, 1252.34)	-1.72% (-1.79, -1.65)
LICs	15–19	549 383 (380 485, 740 846)	766 760 (526 565, 1 031 956)	39.57% (34.56, 44.66)	3184.62 (2205.57, 4294.48)	1974.05 (1355.66, 2656.81)	-1.68% (-1.78, -1.58)
LMCs	15–19	2 084 217 (1 455 413, 2 759 727)	1 417 269 (992 804, 1 878 340)	−32% (−33.4, −30.16)	2193.32 (1531.6, 2904.2)	943.33 (660.81, 1250.22)	-3.0% (-3.13, -2.88)
UMCs	15–19	1 027 234 (737 674, 1 320 533)	585 704 (422 316, 730 621)	−42.98% (−45.28, −40.7)	968.18 (695.27, 1244.62)	735.92 (530.63, 918)	-0.59% (-0.91, -0.28)
Global	20–24	9 364 130 (6 135 124, 12 799 640)	7 004 614 (4 621 514, 9 559 767)	−25.20% (−26.5, −24.02)	3831.99 (2510.62, 5237.88)	2368.21 (1562.5, 3232.09)	-1.51% (-1.55, -1.47)
LICs	20–24	729 133 (461 855, 1 043 180)	1 162 503 (730 000, 1 665 531)	59.44% (52.15, 66.76)	4916.24 (3114.1, 7033.73)	3472.47 (2180.56, 4975.05)	-1.21% (-1.25, -1.16)
LMCs	20–24	4 156 248 (2 683 211, 5 760 740)	3 946 323 (2 561 236, 5 485 880)	−5.05% (−7.41, −2.74)	4849.72 (3130.91, 6721.93)	2773.21 (1799.86, 3855.11)	−1.82% (−1.92, −1.71)
UMCs	20–24	3 626 692 (2 347 200, 4 982 537)	1 493 745 (999 050, 1 953 015)	−58.81% (−61.73, −56.12)	3439.64 (2226.14, 4725.55)	1755.89 (1174.38, 2295.76)	−1.94% (−2.14, −1.73)
Global	25–29	5 606 513 (3 939 975, 7 684 054)	5 490 967 (3 864 321, 7 512 201)	−2.06% (−3.64, −0.49)	2547.15 (1790.01, 3491.02)	1826.10 (1285.14, 2498.30)	−0.93% (−1.00, −0.85)
LICs	25–29	454 226 (315 995, 630 134)	710 925 (487 285, 996 782)	56.51% (49.26, 64.12)	3607.59 (2509.72, 5004.7)	2522.64 (1729.08, 3536.98)	−1.26% (−1.31, −1.2)
LMCs	25–29	2 438 904 (1 707 462, 3 353 290)	2 744 056 (1 911 517, 3 799 326)	12.51% (9.66, 14.96)	3221.54 (2255.38, 4429.35)	2082.41 (1450.61, 2883.23)	−1.54% (−1.57, −1.5)
UMCs	25–29	1 963 811 (1 358 589, 2 736 775)	1 540 939 (1 076 474, 2 119 875)	−21.53% (−24.32, −18.98)	2140.9 (1481.1, 2983.57)	1501.37 (1048.83, 2065.44)	−0.53% (−0.82, −0.25)
Global	30–34	2 350 276 (1 530 057, 3 494 310)	3 034 188 (1 969 911, 4 504 000)	29.10% (26.29, 31.85)	1235.92 (804.6, 1837.53)	1016.28 (659.81, 1508.59)	−0.32% (−0.47, −0.18)
LICs	30–34	259 131 (167 921, 388 940)	423 464 (271 463, 645 750)	63.42% (54.08, 73.79)	2542.53 (1647.6, 3816.19)	1812.37 (1161.82, 2763.72)	−1.13% (−1.17, −1.09)
LMCs	30–34	1 050 848 (686 806, 1 569 120)	1 299 430 (836 718, 1 947 394)	23.66% (19.74, 27.41)	1643.04 (1073.85, 2453.38)	1068.96 (688.31, 1602)	−1.57% (−1.66, −1.48)
UMCs	30–34	712 393 (459 407, 1 062 240)	935 547 (598 204, 1 386 349)	31.32% (26.9, 36.14)	926.48 (597.47, 1381.46)	819.44 (523.96, 1214.30)	0.87% (0.39, 1.35)
Global	35–39	1 069 279 (698 567, 1 582 333)	1 390 759 (905 238, 2 057 087)	30.07% (26.84, 33.14)	615.46 (402.08, 910.76)	518.1(337.23, 766.32)	−0.34% (−0.54, −0.15)
LICs	35–39	168 137 (106 448, 253 929)	267 946 (170 782, 406 502)	59.36% (51.64, 68.84)	1984.8 (1256.58, 2997.54)	1368.86 (872.47, 2076.7)	−1.23% (−1.26, −1.2)
LMCs	35–39	522 607 (342 147, 767 491)	579 188 (378 587, 847 885)	10.83% (6.66, 14.56)	960.17 (628.62, 1410.09)	525.55 (343.52, 769.36)	−2.14% (−2.21, −2.07)
UMCs	35–39	278 146 (177 582, 417 163)	360 447 (234 690, 540 639)	29.59% (25.02, 34.22)	376.75 (240.54, 565.06)	369.61 (240.65, 554.38)	1.16% (0.53, 1.79)
Global	40–44	431 230 (300 232, 598 296)	528 481 (365 267, 735 798)	22.55% (19.94, 25.23)	307.78 (214.29, 427.03)	215.97 (149.27, 300.70)	−0.99% (−1.19, −0.79)
LICs	40–44	85 646 (58 472, 118 869)	136 461 (93 725, 190 590)	59.33% (53.29, 66.55)	1311.16 (895.15, 1819.77)	860.26 (590.85, 1201.49)	−1.54% (−1.59, −1.48)
LMCs	40–44	227 543(158 578, 316 101)	221 125 (154 761, 307 435)	−2.82% (−5.77, 0.26)	531.18 (370.18, 737.91)	231.89 (162.30, 322.41)	−2.74% (−2.79, −2.69)
UMCs	40–44	94 169 (65 769, 130 725)	114 750 (79 076, 160 644)	21.86% (18.07, 24.96)	169.23 (118.2, 234.93)	123.04 (84.79, 172.25)	0.07% (−0.63, 0.77)
Global	45–49	157 796 (102 029, 225 593)	166 584 (107 500, 236 628)	5.57% (3.15, 8.09)	138.55 (89.59, 198.08)	70.76 (45.66, 100.51)	−1.87% (−2.12, −1.63)
LICs	45–49	36 063 (23 147, 50 236)	43 273 (27 777, 60 527)	19.99% (15.18, 24.89)	642.7 (412.53, 895.31)	333.56 (214.11, 466.56)	−2.33% (−2.54, −2.12)
LMCs	45–49	90 466 (58 405, 131 217)	78 433 (51 179, 109 888)	−13.30% (−17.41, −9.68)	251.35 (162.27, 364.57)	95.27 (62.16, 133.47)	−3.13% (−3.23, −3.03)
UMCs	45–49	28 318 (18 769, 39 260)	37 354 (23 317, 54 993)	31.91% (22.43, 43.88)	65.43 (43.37, 90.72)	37.8 (23.6, 55.65)	−0.32% (−1.33, 0.7)
Global	50–54	3326 (2479, 4365)	3566 (2619, 4661)	7.19% (5.04, 9.29)	3.17 (2.36, 4.16)	1.63 (1.19, 2.13)	−2.07% (−2.27, −1.86)
LICs	50–54	742 (542, 969)	814 (590, 1061)	9.67% (6.08, 13.06)	15.56 (11.36, 20.31)	7.93 (5.76, 10.35)	−2.27% (−2.49, −2.04)
LMCs	50–54	1879 (1400, 2495)	1616 (1208, 2099)	−13.96% (−17.11, −11.35)	6.07 (4.52, 8.06)	2.31 (1.73, 3.00)	−3.26% (−3.36, −3.17)
UMCs	50–54	638 (480, 819)	945 (675, 1293)	48.14% (38.48, 58.49)	1.51 (1.14, 1.94)	0.96 (0.69, 1.32)	−0.38% (−1.31, 0.56)

ASIR – age-standardised incidence rate (per 100 000 population), CI – confidence interval, EAPC – estimated annual percentage change, LICs – low-income countries and territories, LMCs – lower-middle-income countries and territories, MSMI – maternal sepsis and other maternal infections, UI – uncertain interval, UMCs – upper-middle-income countries and territories

**Table 4:** MMR of MSMI in 131 low- and middle-income countries and territories with EAPC in different age groups from 1990 to 2019

Location	Age group, in years	Deaths in 1990 (95% UI)	Deaths in 2019 (95% UI)	Relative change, % (95% CI)	Age-standardised MMR in 1990 (95% UI)	Age-standardised MMR in 2019 (95% UI)	EAPC, % (95% CI)
Global	10–14	151 (114, 199)	76 (55, 108)	−49.93% (−66.57, −26.1)	77.37 (58.59, 101.92)	35.74 (26.08, 51.06)	–2.37% (–2.55, –2.19)
LICs	10–14	36 (25, 51)	50 (32, 79)	36.78% (−17.6, 130.39)	82.51 (56.96, 115.85)	64.77 (41.67, 103.43)	0.00% (–0.57, 0.58)
LMCs	10–14	92 (62, 136)	23 (15, 33)	−75.52% (−84.81, −60)	113.39 (76.02, 166.95)	26.78 (18.26, 38.65)	–5.43% (–5.65, –5.21)
UMCs	10–14	22 (17, 28)	3 (2, 4)	−85.61% (−90.22, −79.12)	37.76 (29.77, 49.35)	7.28 (5.45, 9.54)	–5.29% (–5.51, –5.07)
Global	15–19	4773 (3619, 6127)	1713 (1355, 2099)	−64.11% (−73.58, −51.44)	30.54 (23.16, 39.2)	14.34 (11.34, 17.57)	–2.71% (–3.02, –2.38)
LICs	15–19	889 (704, 1117)	1012 (778, 1264)	13.80% (−14.01, 48.09)	36.78 (29.09, 46.18)	28.61 (22, 35.73)	–0.36% (–0.86, 0.14)
LMCs	15–19	3464 (2418, 4769)	640 (478, 870)	−81.54% (−87.75, −71.45)	42.03 (29.33, 57.85)	11.04 (8.25, 15.01)	–5.06% (–5.54, –4.58)
UMCs	15–19	408 (350, 475)	59 (47, 71)	−85.66% (−88.7, −81.43)	10.53 (9.03, 12.24)	2.62 (2.11, 3.2)	–4.44% (–4.66, –4.22)
Global	20–24	8745 (6784, 11207)	3026 (2421, 3637)	−65.40% (−74.39, −54.7)	19.09 (14.81, 24.47)	8.54 (6.84, 10.27)	–3.06% (–3.42, –2.69)
LICs	20–24	1780 (1397, 2226)	1743 (1356, 2179)	−2.10% (−27.7, 26.96)	42.09 (33.05, 52.64)	24.99 (19.45, 31.26)	–1.45% (–1.93, –0.97)
LMCs	20–24	5929 (4134, 8219)	1179 (878, 1566)	−80.11% (−86.76, −69.8)	29.11 (20.3, 40.36)	5.94 (4.42, 7.89)	–5.94% (–6.39, –5.48)
UMCs	20–24	1007 (861, 1194)	97 (80, 118)	−90.37% (−92.58, −87.69)	5.68 (4.86, 6.74)	1.39 (1.16, 1.69)	–5.19% (–5.47, –4.9)
Global	25–29	8127 (6573, 9874)	3231 (2643, 3880)	−60.25% (−69.21, −48.98)	20.30 (16.42, 24.66)	8.12 (6.64, 9.75)	–3.26% (–3.78, –2.74)
LICs	25–29	2147 (1679, 2716)	1905 (1477, 2388)	−11.28% (−33.13, 18.71)	57.69 (45.12, 72.97)	32.17 (24.94, 40.33)	–1.61% (–2.06, –1.17)
LMCs	25–29	4977 (3615, 6627)	1203 (907, 1571)	−75.82% (−83.51, −65.04)	29.15 (21.17, 38.81)	6.05 (4.56, 7.9)	–5.72% (–6.26, –5.18)
UMCs	25–29	961 (812, 1133)	113 (90, 140)	−88.26% (−91.16, −84.94)	6.76 (5.71, 7.97)	1.06 (0.84, 1.32)	–6.74% (–7.2, –6.29)
Global	30–34	6393 (5265, 7912)	2935 (2415, 3555)	−54.10% (−64.43, −42.02)	29.21 (24.05, 36.15)	10.10 (8.31, 12.24)	–3.55% (–3.97, –3.14)
LICs	30–34	1934 (1482, 2513)	1759 (1380, 2238)	−9.07% (−32.47, 19.78)	74.11 (56.78, 96.29)	40.90 (32.08, 52.04)	–1.71% (–2.1, –1.32)
LMCs	30–34	3639 (2726, 4940)	1053 (819, 1359)	−71.05% (−79.72, −58.88)	37.03 (27.74, 50.27)	8.71 (6.77, 11.23)	–5.20% (–5.74, –4.67)
UMCs	30–34	781 (678, 904)	112 (91, 137)	−85.66% (−88.79, −81.46)	12.36 (10.74, 14.32)	1.29 (1.06, 1.58)	–7.74% (–8.01, –7.46)
Global	35–39	5182 (4336, 6211)	2717 (2251, 3308)	−47.56% (−58.46, −34.36)	49.68 (41.57, 59.54)	19.45 (16.11, 23.68)	–3.25% (–3.6, –2.9)
LICs	35–39	1835 (1425, 2342)	1705 (1349, 2164)	−7.06% (−31.01, 23.19)	112.11 (87.06, 143.1)	65.34 (51.7, 82.91)	–1.65% (–2.01, –1.29)
LMCs	35–39	2703 (2094, 3492)	909 (710, 1161)	−66.36% (−75.95, −52.91)	53.45 (41.41, 69.06)	16.70 (13.04, 21.33)	–4.18% (–4.66, –3.69)
UMCs	35–39	616 (535, 710)	93 (76, 113)	−84.87% (−88.01, −80.66)	23.24 (20.17, 26.76)	2.56 (2.1, 3.1)	–7.68% (–7.86, –7.51)
Global	40–44	3019 (2463, 3596)	2064 (1658, 2605)	−31.63% (−47.34, −11.44)	91.51 (74.66, 109)	50.75 (40.78, 64.05)	–2.15% (–2.55, –1.76)
LICs	40–44	1315 (1005, 1673)	1439 (1086, 1899)	9.48% (−22.47, 52.87)	213.84 (163.44, 272.03)	151.23 (114.11, 199.56)	–0.94% (–1.33, –0.54)
LMCs	40–44	1441 (1103, 1832)	570 (446, 720)	−60.45% (−70.99, −44.24)	81.54 (62.44, 103.69)	35.39 (27.73, 44.74)	–3.23% (–3.57, –2.89)
UMCs	40–44	248 (214, 284)	48 (40, 58)	−80.49% (−84.52, −75.87)	34.66 (29.91, 39.79)	4.87 (3.99, 5.85)	–7.05% (–7.28, –6.82)
Global	45–49	1419 (1170, 1721)	985 (748, 1300)	−30.62% (−48.96, −7.21)	171.83 (141.63, 208.4)	112.49 (85.42, 148.46)	–1.53% (–1.94, –1.12)
LICs	45–49	780 (590, 1020)	751 (533, 1053)	−3.67% (−34.67, 42.03)	452.97 (342.67, 592.49)	329.84 (234.17, 462.14)	–0.70% (–1.09, –0.31)
LMCs	45–49	538 (412, 709)	207 (160, 268)	−61.57% (−72.87, −45.3)	109.87 (84.19, 144.75)	50.18 (38.89, 65.08)	–2.90% (–3.21, –2.6)
UMCs	45–49	94 (79, 112)	23 (19, 28)	−75.21% (−80.4, −67.97)	64.49 (54.13, 76.99)	11.88 (9.8, 14.53)	–6.39% (–6.67, –6.11)
Global	50–54	218 (164, 295)	94 (72, 124)	−56.89% (–70.43, –38.82)	912.09 (685.73, 1232.86)	361.35 (274.66, 475.48)	–3.22% (–3.52, –2.92)
LICs	50–54	66 (45, 98)	47 (33, 67)	–28.26% (–55.89, 13.67)	1312.19 (892.79, 1949.36)	772.62 (534.5, 1091.33)	–1.54% (–1.93, –1.15)
LMCs	50-54	142 (97, 202)	45 (30, 66)	–67.96% (–81.2, –46.22)	1013.58 (691.82, 1443.1)	380.08 (253.4, 548.48)	–3.33% (–3.57, –3.1)
UMCs	50–54	10 (8, 13)	1 (1, 2)	–86.39% (–90.12, –81.71)	223.42 (175.04, 292.11)	20.03 (16.44, 24.41)	–8.46% (–8.99, –7.92)

ASMMR – age-standardised maternal mortality ratio (per 100 000 livebirths), CI – confidence interval, EAPC – estimated annual percentage change, LICs – low-income countries and territories, LMCs – lower-middle-income countries and territories, MSMI – maternal sepsis and other maternal infections, UI – uncertain interval, UMCs – upper-middle-income countries and territories

**Table 5:** DALYs of MSMI in 131 low- and middle-income countries with EAPC in different age groups from 1990 and 2019

Location	Age group, in years	DALYs in 1990 (95% UI)	DALYs in 2019 (95% UI)	Relative change, % (95% CI)	Age-standardised DALYs rate in 1990 (95% UI)*	Age-standardised DALYs rate in 2019 (95% UI)*	EAPC, % (95% CI)
Global	10–14	11 776 (8951, 15 403)	6037 (4432, 8494)	−48.73% (−65.16, −25.37)	1.38 (1.05, 1.81)	0.64 (0.47, 0.9)	−2.38% (−2.65, −2.11)
LICs	10–14	2829 (1967, 3944)	3888 (2535, 6133)	37.40% (−15.47, 128.32)	3.77 (2.62, 5.26)	2.63 (1.72, 4.16)	−0.49% (−1.06, 0.08)
LMCs	10–14	7171 (4835, 10 486)	1840 (1270, 2601)	−74.34% (−83.93, −58.96)	1.94 (1.31, 2.83)	0.4 (0.27, 0.56)	−6.03% (−6.3, −5.76)
UMCs	10–14	1726 (1378, 2247)	290 (222, 369)	−83.22% (−88.25, −76.25)	0.57 (0.45, 0.74)	0.12 (0.09, 0.15)	−4.62% (−4.9, −4.34)
Global	15–19	357 050 (273 775, 455 659)	134 087 (108 142, 163 063)	−62.45% (−71.79, −49.95)	139.73 (107.14, 178.32)	44.44 (35.84, 54.04)	−4.05% (−4.35, −3.74)
LICs	15–19	65 760 (52 070, 81 826)	75 431 (58 837, 93 439)	14.71% (−13.22, 47.95)	381.19 (301.84, 474.32)	194.2 (151.48, 240.56)	−1.89% (−2.46, −1.31)
LMCs	15–19	255 852 (180 309, 350 034)	51 407 (39 403, 68 228)	−79.91% (−86.21, −69.76)	269.25 (189.75, 368.36)	34.22 (26.23, 45.41)	−7.42% (−7.97, −6.88)
UMCs	15–19	33 350 (28 425, 39 057)	6562 (4935, 8513)	−80.32% (−84.65, −75.49)	31.43 (26.79, 36.81)	8.25 (6.2, 10.7)	−4.11% (−4.57, −3.64)
Global	20–24	619 895 (489 991, 778 300)	229 815 (185 098, 278 688)	−62.93% (−71.82, −52.8)	253.67 (200.51, 318.5)	77.7 (62.58, 94.22)	−4.25% (−4.58, −3.93)
LICs	20–24	121 326 (96 432, 150 345)	120 680 (94 928, 149 496)	−0.53% (−25.27, 27.71)	818.05 (650.2, 1013.71)	360.48 (283.56, 446.55)	−2.49% (−3.00, −1.97)
LMCs	20–24	411 468 (295 490, 562 349)	94 523 (70 538, 124 032)	−77.03% (−84.29, −66.38)	480.12 (344.79, 656.18)	66.42 (49.57, 87.16)	−7.03% (−7.48, −6.57)
UMCs	20–24	81 660 (67 492, 100 791)	12 521 (8599, 17 906)	−84.67% (−88.47, −80.23)	77.45 (64.01, 95.59)	14.72 (10.11, 21.05)	−5.65% (−6.00, −5.29)
Global	25–29	523 802 (426 844, 632 636)	221 685 (182 076, 267 770)	−57.68% (−67.07, −46.65)	237.97 (193.92, 287.42)	73.72 (60.55, 89.05)	−4.03% (−4.44, −3.62)
LICs	25–29	134 154 (105 214, 168 525)	120 304 (93 515, 149 668)	−10.32% (−32.15, 19.3)	1065.49 (835.64, 1338.48)	426.88 (331.83, 531.08)	−2.76% (−3.25, −2.27)
LMCs	25–29	316 933 (233 559, 419 463)	85 583 (65 445, 109 223)	−73% (−81.32, −61.78)	418.64 (308.51, 554.07)	64.95 (49.66, 82.89)	−6.67% (−7.14, −6.20)
UMCs	25–29	67 146 (55 960, 80 131)	13 216 (9327, 19 204)	−80.32% (−85.71, −73.63)	73.2 (61.01, 87.36)	12.88 (9.09, 18.71)	−5.83% (−6.07, −5.60)
Global	30–34	372 251 (307 466, 459 303)	179 234 (147 168, 215 704)	−51.85% (−62.42, −39.44)	195.75 (161.68, 241.53)	60.03 (49.29, 72.25)	−3.81% (−4.11, −3.50)
LICs	30–34	110 712 (85 547, 142 974)	101 458 (80 414, 128 428)	−8.36% (−31.69, 20.34)	1086.28 (839.37, 1402.83)	434.22 (344.16, 549.65)	−2.80% (−3.23, −2.37)
LMCs	30–34	210 876 (158 158, 284 103)	65 505 (52 053, 83 780)	−68.94% (−77.93, −56.8)	329.71 (247.29, 444.21)	53.89 (42.82, 68.92)	−6.44% (−6.88, −6.00)
UMCs	30–34	47 081 (40 956, 54 325)	10 155 (7494, 14 231)	−78.43% (−83.84, −71.06)	61.23 (53.26, 70.65)	8.9 (6.56, 12.47)	−6.16% (−6.47, −5.85)
Global	35–39	272 825 (228 787, 327 491)	146 974 (122 142, 177 855)	−46.13% (−57.08, −33.01)	157.03 (131.69, 188.5)	54.75 (45.5, 66.26)	−3.53% (−3.72, −3.33)
LICs	35–39	95 612 (74 583, 121 729)	89 375 (70 913, 112 973)	−6.52% (−30.34, 23.6)	1128.67 (880.43, 1436.97)	456.59 (362.28, 577.14)	−2.86% (−3.25, −2.47)
LMCs	35–39	142 351 (110 668, 183 813)	50 030 (39 279, 63 399)	−64.85% (−74.56, −51.38)	261.54 (203.33, 337.72)	45.4 (35.64, 57.53)	−6.16% (−6.58, −5.74)
UMCs	35–39	32 978 (28 707, 37 860)	6316 (5023, 7976)	−80.85% (−85.08, −75.42)	44.67 (38.88, 51.28)	6.48 (5.15, 8.18)	−6.35% (−6.91, −5.79)
Global	40–44	143 587 (117 670, 170 145)	99 662 (80 765, 125 155)	−30.59% (−46.33, −10.74)	102.48 (83.99, 121.44)	40.73 (33.01, 51.15)	−3.18% (−3.39, −2.97)
LICs	40–44	61 945 (47 309, 78 724)	68 058 (51 747, 89 500)	9.87% (−22.18, 53.05)	948.32 (724.25, 1205.19)	429.04 (326.22, 564.21)	−2.55% (−3.02, −2.08)
LMCs	40–44	68 793 (53 117, 87 233)	28 274 (22 593, 35 485)	−58.90% (−69.58, −42.6)	160.59 (124, 203.64)	29.65 (23.69, 37.21)	−5.99% (−6.30, −5.68)
UMCs	40–44	12 002 (10 351, 13 687)	2790 (2261, 3399)	−76.75% (−81.57, −71.11)	21.57 (18.6, 24.6)	2.99 (2.42, 3.64)	−6.61% (−7.14, −6.08)
Global	45–49	60 692 (50 182, 73 386)	42 877 (32 999, 56 156)	−29.35% (−47.43, −6.46)	53.29 (44.06, 64.44)	18.21 (14.02, 23.85)	−3.42% (−3.69, −3.16)
LICs	45–49	32 918 (25 025, 43 066)	31 823 (22 634, 44 522)	−3.33% (−34.02, 42.04)	586.66 (445.99, 767.52)	245.3 (174.47, 343.19)	−2.71% (−3.24, −2.19)
LMCs	45–49	23 334 (18 069, 30 368)	9652 (7533, 12 199)	−58.64% (−70.39, −42.24)	64.83 (50.2, 84.37)	11.72 (9.15, 14.82)	−5.89% (−6.17, −5.62)
UMCs	45–49	4083 (3442, 4830)	1198 (980, 1460)	−70.65% (−77.04, −62.67)	9.43 (7.95, 11.16)	1.21 (0.99, 1.48)	−6.37% (−7.09, −5.65)
Global	50–54	8617 (6490, 11 464)	4298 (3232, 5642)	−50.13% (−65.75, −30.13)	8.21 (6.18, 10.92)	1.96 (1.47, 2.57)	−4.81% (−4.96, -4.66)
LICs	50–54	2510 (1739, 3686)	1881 (1340, 2612)	−25.03% (−53.73, 15.73)	52.63 (36.46, 77.31)	18.35 (13.07, 25.47)	−3.36% (−3.9, -2.81)
LMCs	50–54	5650 (3913, 7943)	2264 (1556, 3206)	−59.92% (−75.05, −35.81)	18.25 (12.64, 25.66)	3.24 (2.22, 4.58)	−5.92% (−6.05, −5.79)
UMCs	50–54	403 (322, 517)	118 (76, 190)	−70.63% (−81.32, −54.69)	0.95 (0.76, 1.22)	0.12 (0.08, 0.19)	−6.50% (−6.91, −6.10)

CI – confidence interval, DALYs – disability-adjusted life years, EAPC – estimated annual percentage change, LICs – low-income countries and territories, LMCs – lower-middle-income countries and territories, MSMI – maternal sepsis and other maternal infections, UI – uncertain interval, UMCs – upper-middle-income countries and territories

*Age-standardised DALYs per 100 000 population.

**Figure 4:**
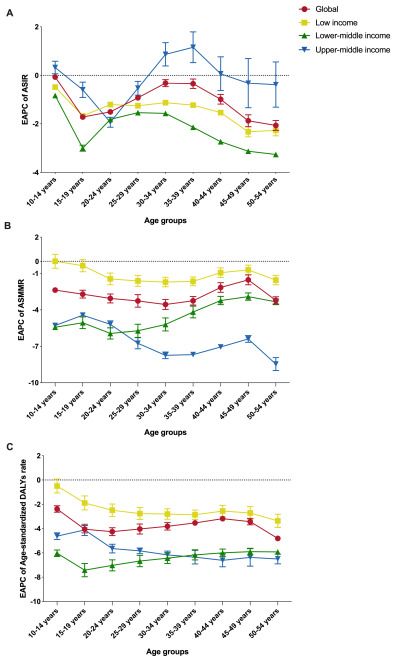
The EAPC of ASIR per 100 000 population (**Panel A**), ASMMR per 100 000 livebirths (**Panel B**), and age-standardised DALY rates per 100 000 population (**Panel C**) for MSMI among different age groups globally and in LIMCs, 1990–2019. ASIR – age-standardised incidence rate, ASMMR – age-standardised maternal mortality ratio, DALYs – disability-adjusted life years, EAPC – estimated annual percentage change, LIMCs – low- and middle-income countries (and territories), MSMI – maternal sepsis and other maternal infections.

Globally, almost all of the age groups of ASIR, ASMMR, age-standardised DALYs rates showed a downward trend at global level between 1990 and 2019, with the speediest decrease in ASIR (EAPC = −2.07%, 95% CI = −2.27, −1.86%) and age-standardised DALYs rates in 50–54 years group (EAPC = −4.81%, 95% CI = −4.96, −4.66%), and the speediest decrease in ASMMR in 30–34 years group (EAPC = −3.55%, 95% CI = −3.97, −3.14%) (**Figure 4**, panels A–C). Among income-classified regions, the vast majority of all age groups of ASIR, ASMMR, age-standardised DALYs rates of LMICs had downtrends between 1990 and 2019, except for the uptrends of ASIR among 10–14 years (EAPC = 0.32%), 30–34 years (EAPC = 0.87%), and 35–39 years group (EAPC = 1.16%) and the stable status in ≥40 years group in UMCs. In LICs, ASMMR and age-standardised DALYs rates in 10–14 years group remained stable over these three decades. Overall, the decreasing trends of disease burden in LMCs and UMCs was more significant than that in LICs in all age groups, along with better changes than those of global average, except in ASIR. The ASMMR and age-standardised DALYs rates of LMCs decreased more rapidly in younger age groups than in UMCs, but the opposite was true in older age groups. The national trend of ASIR, ASMMR, age-standardised DALYs rates in 131 LMICs of all age groups are also presented in Tables S7–33 in the **Online Supplementary Document**.

### Factors associated with MSMI burden and EAPCs

**Figure 5:**
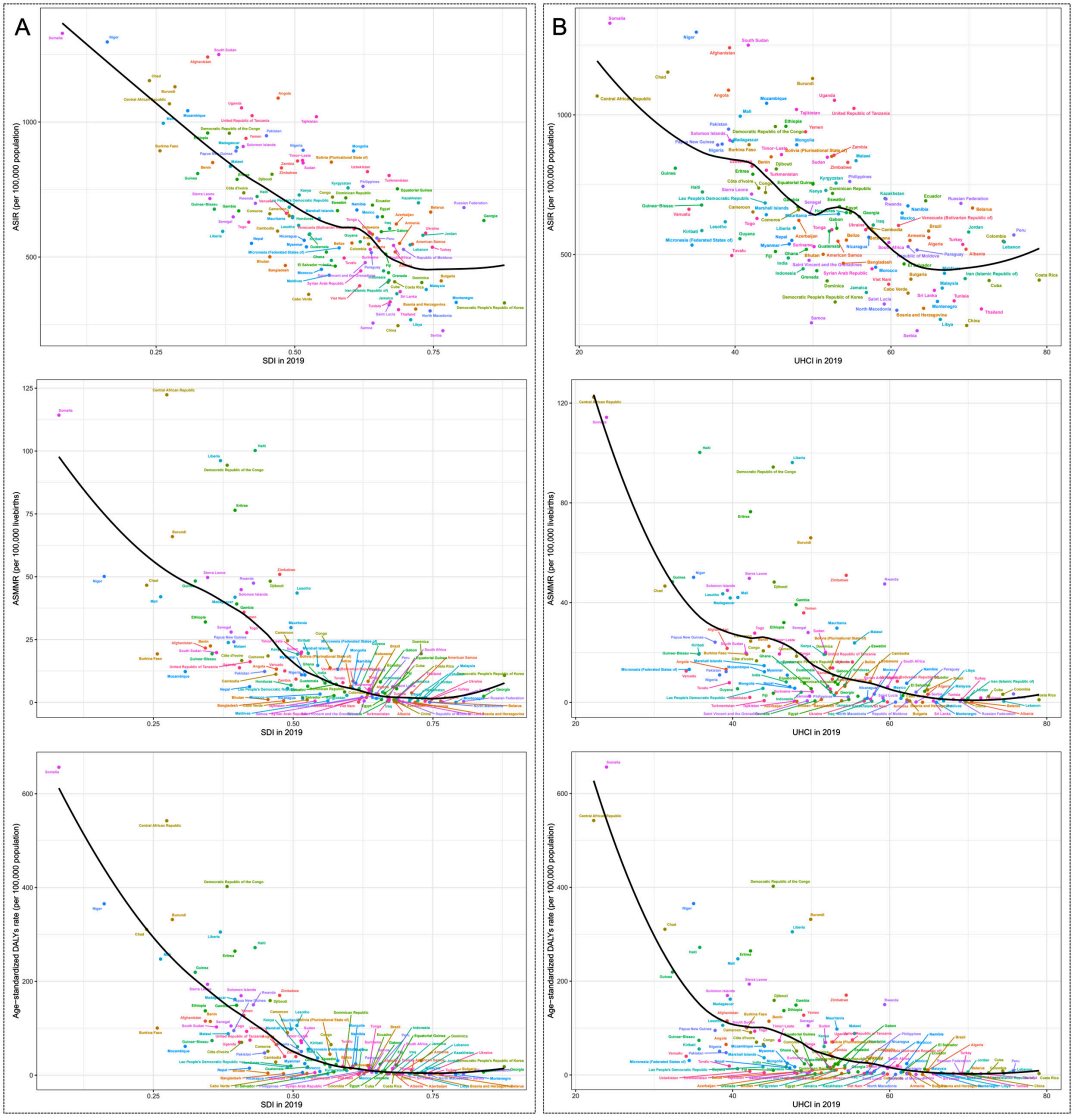
ASIR per 100 000 population, ASMMR per 100 000 livebirths, and age-standardised DALY rates per 100 000 population for MSMI globally by SDI (**Panel A**) and UHCI (**Panel B**) in 2019. ASIR – age-standardised incidence rate, ASMMR – age-standardised maternal mortality ratio, DALYs – disability-adjusted life years, MSMI – maternal sepsis and other maternal infections, SDI – sociodemographic index, UHCI – universal health coverage effective coverage index.

Global ASIR, ASMMR, and age-standardised DALYs rates all decreased significantly with increasing SDI between 1990 and 2019 (all ρ<0, *P* < 0.05) (Figure S3 in the **Online Supplementary Document**). In 2019, there was an inverse association between the ASIR, ASMMR, age-standardised DALYs rates for MSMI and SDI and UHCI in 131 LMICs, with some exceptions (**Figure 5**, panels A–B). With the growth of SDI and UHCI, the decreasing trend of ASIR, ASMMR, and age-standardised DALYs rates slowed down.

**Figure 6:**
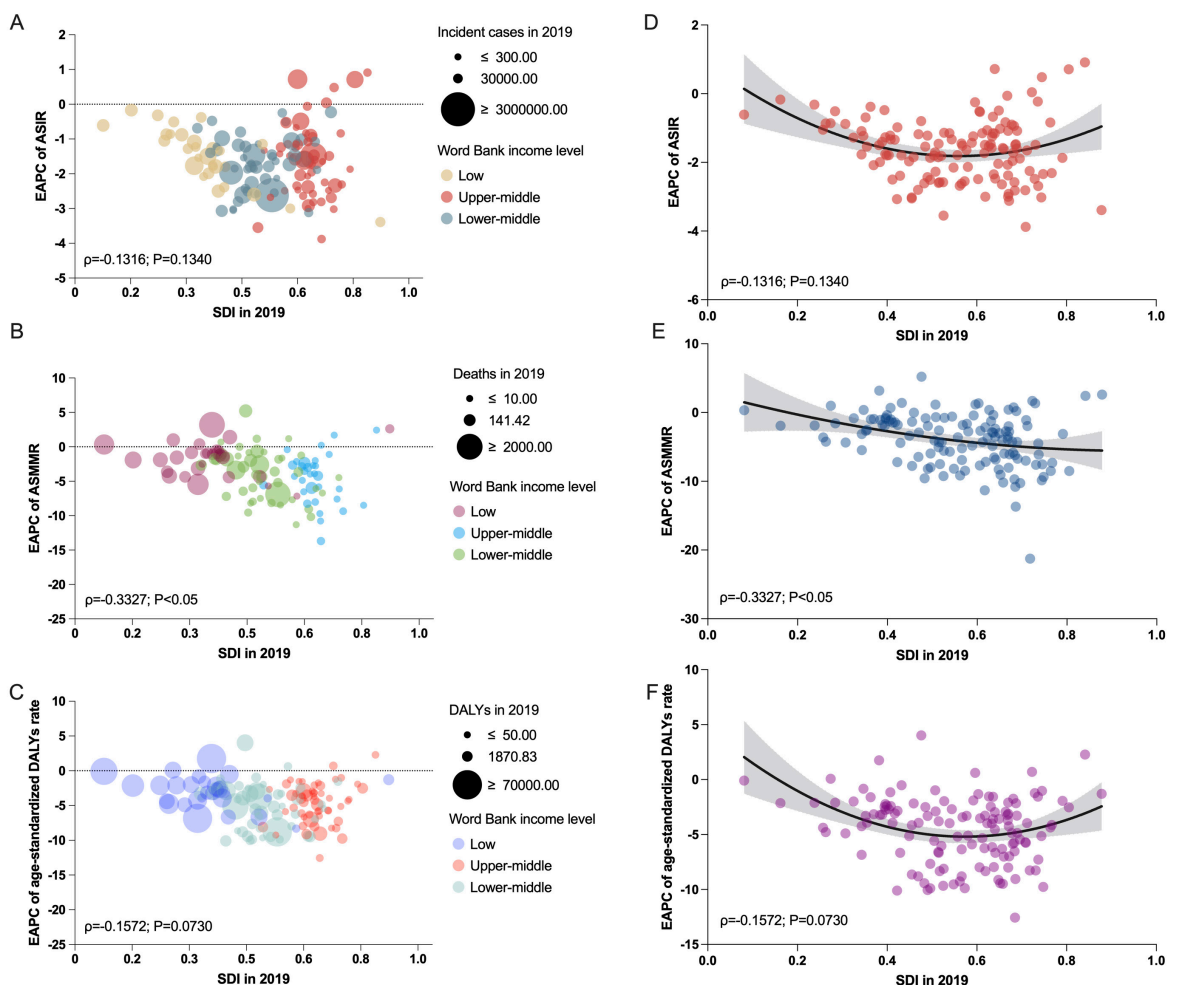
The correlation between the EAPC of ASIR per 100 000 population (**Panels A** and (**D**)/ASMMR per 100 000 livebirths (**Panels B** and (**E**)/age-standardised DALY rates per 100 000 population (**Panels C** and (**F**) of MSMI and SDI (in 2019) in LMICs. Spearman correlation analyses were employed. ASIR – age-standardised incidence rate, ASMMR – age-standardised maternal mortality ratio, DALYs – disability-adjusted life years, EAPC – estimated annual percentage change, LIMCs – low- and middle-income countries (and territories), MSMI – maternal sepsis and other maternal infections, SDI – sociodemographic index.

**Figure 7:**
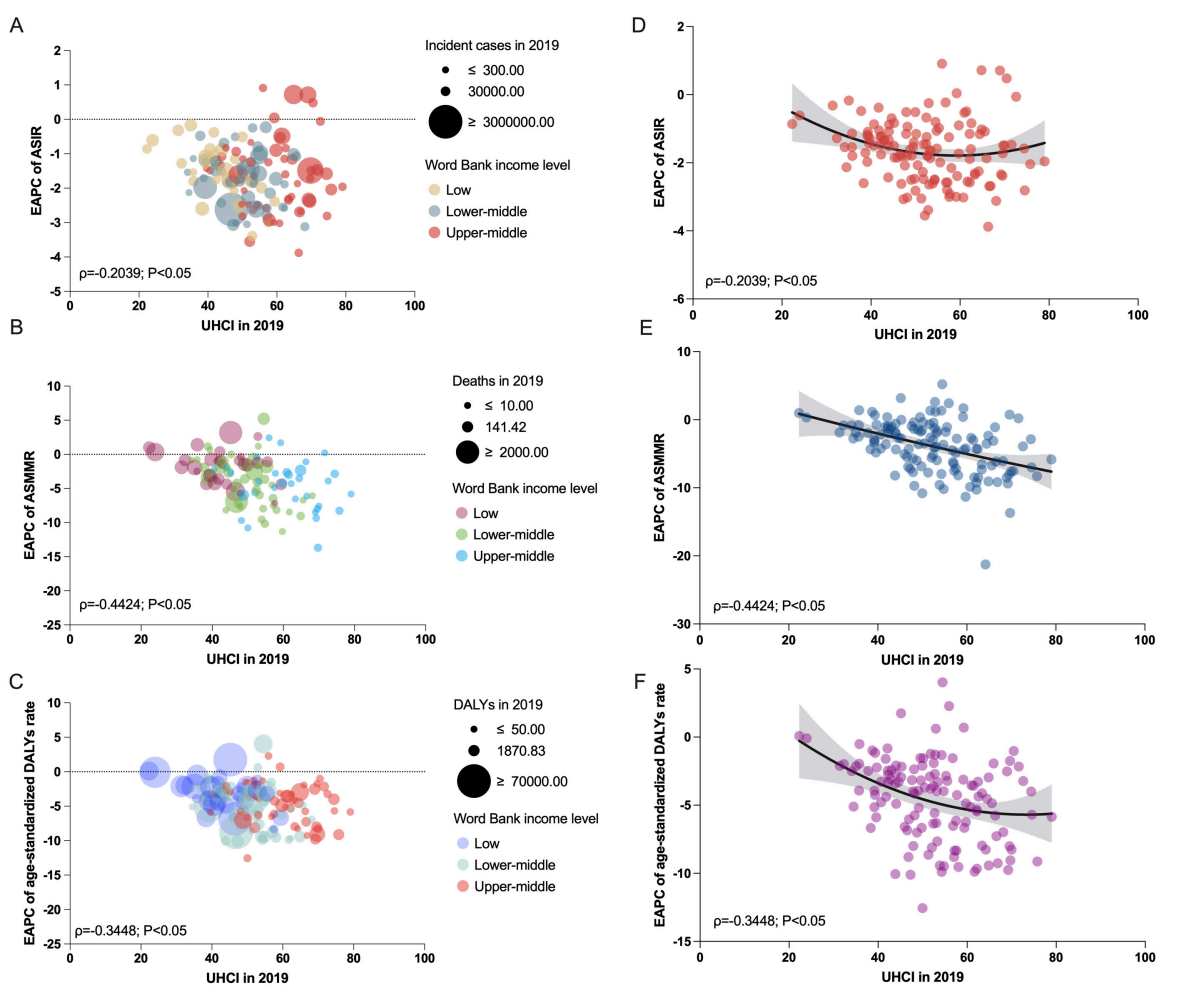
The correlation between the EAPC of ASIR per 100 000 population (**Panels A** and (**D**)/ASMMR per 100 000 livebirths (**Panels B** and (**E**)/age-standardised DALY rates per 100 000 population (**Panels C** and (**F**) of MSMI and UHCI (in 2019) in LMICs. Spearman correlation analyses were employed. ASIR – age-standardised incidence rate, ASMMR – age-standardised maternal mortality ratio, DALYs – disability-adjusted life years, EAPC – estimated annual percentage change, LIMCs – low- and middle-income countries (and territories), MSMI – maternal sepsis and other maternal infections, UHCI – universal health coverage effective coverage index.

The observed global EAPCs of ASIR, ASMMR, and age-standardised DALYs rates in relation to SDI and UHCI in 2019 are shown in **Figure 6**, panels A–F and **Figure 7**, panels A–F. Globally, the EAPCs of ASIR and age-standardised DALYs rates of MSMI had a U-shaped association with SDI in 2019, with the peak point appearing SDI between 0.5 and 0.6. Similar patterns were observed in all country levels, except for the negative association between the EAPC of ASIR and SDI in LICs (Figure S4 in the **Online Supplementary Document**). A significant negative correlation was detected between EAPC of ASMMR of MSMI and SDI in 2019 (ρ = −0.33, *P* < 0.05) (**Figure 6**, panel E). In terms of the country classifications by income level, a similar negative correlation was present only for LMCs, with a U-shaped correlation in LICs (Figure S4 in the **Online Supplementary Document**). The EAPCs of ASIR, ASMMR, and age-standardised DALYs rates of 131 LMICs all negatively in relation to the UHCI in 2019 (all ρ<0, *P* < 0.05) (**Figure 7**, panels A–F). Moreover, the EAPC of ASIR in LICs, the EAPC of ASMMR in LMCs and UMCs, the EAPC of age-standardised DALYs rates in LICs and LMCs were inversely associated with the UHCI in 2019 (Figure S5 in the **Online Supplementary Document**).

## DISCUSSION

To our knowledge, this is the first systematic overview to integrate the disease burden of MSMI in 131 LIMICs over past three decades, exploring its secular trends, regional variations, and associations with SDI and UHCI. In this study, we found that only the number of incident cases in LICs increased, with a relative percentage change over 50%. Between 1990 and 2019, the ASIRs, ASMMRs and age-standardised DALYs rates of LICs were much higher than that of LMCs and UMCs, although they all continued to decline. At least six of 131 LMICs had ASMMR greater than 70.00 per 100 000 livebirths in 2019. The EAPCs of ASIR, ASMMR, and age-standardised DALYs rates in vast majority of LMICs showed downward trends, but there are some exceptions. The incidences of MSMI increased first till 20–24 years and then decreased with age both in 1990 and 2019, while the ASMMRs were higher in the youngest and the oldest age group. With the growth of SDI and UHCI in 2109, the decreasing trend of ASIR, ASMMR, and age-standardised DALYs rates slowed down. A significant negative correlation was detected between EAPC of ASMMR of MSMI and SDI in 2019, while the EAPCs of all age-standardised rates were negatively in relation to the UHCI in 2019.

Our findings on the global and regional trends of MSMI align with previous researches. Chen et al. discerned a consistent decline in the ASIR, ASMMR, and age-standardised DALYs rates attributable to MSMI on a global scale, spanning the temporal continuum from 1990 to 2019 [18]. Significantly, this descent was most pronounced in regions characterised by high MSMI burden and fasted in regions with lower SDI. Of note, MSMI stands distinguished as one of the triumvirates of principal causes underpinning maternal mortality [29]. An exhaustive scrutiny conducted by the United Nations Maternal Mortality Estimation Inter-Agency Group elucidated a noteworthy reduction in the global MMR – plummeting from 385 deaths per 100 000 livebirths in 1990 to 216 deaths in 2015, a substantial 43.9% relative decrease over the course of 25 years [30]. Encouragingly, advancements in diminishing MMR exhibit regional differentials, manifesting as an annualised decrement spanning from 1.8% in the Caribbean to a more robust 5.0% in East Asia since 1990 [30]. The discernments proffered by the GLOSS accentuated a higher incidences and deaths of maternal sepsis within economies characterised by diminished fiscal affluence [8]. Furthermore, the investigation accentuated the imperativeness of elevating the echelons of health care provision, fortifying infection control paradigms, and ameliorating health care infrastructural conditions [8]. Such imperatives, culled from empirical observations, furnish pivotal data and strategic counsel germane to the amelioration of maternal health on a global scale.

Maternal mortality, with a particular emphasis on deaths entwined with infections, has long held a pivotal and apprehension-inducing position in the ambit of global health concerns [29, 31]. A salient example of this is its conspicuous alignment with the objectives encapsulated in both the MDGs and the SDGs [4,14]. Numerous scholarly inquiries have documented a discernible decrease in the incidence rate and MMR of MSMI over the preceding decades, emblematic of strides made in enhancing maternal health outcomes [5,29,30]. Our findings reveal that, despite a consistent annual decline in the ASIR and ASMMR in LMICs, LICs manifested markedly higher ASIR and ASMMR than LMCs and UMCs, with the slowest downtrend. In 2019, LICs reported an ASMMR of 42.31 per 100 000 livebirths, significantly deviating from the benchmarks set by the SDGs [4]. Although strides have been made in mitigating the burden of MSMI in low-income regions, the deficiencies in health care systems have exacerbated the gap among LMICs in the management of maternal infections [9,10,32]. LMICs, particularly those with lower fiscal capacity, such as Central African Republic, Somalia, Haiti, Liberia, the Democratic Republic of the Congo, and Eritrea, persist in grappling with formidable challenges in addressing maternal health issues [10,20]. This glaring burden disparity necessitates targeted interventions and resource allocation to address the specific challenges confronted by LMICs. The epidemiological landscape of MSMI exhibited substantial heterogeneity across 131 LMICs in 2019, with Somalia registering the highest estimated national-level ASIR (1328.40 per 100 000 population) and age-standardised DALYs rate. The occurrence of maternal sepsis events is intimately linked to the ubiquity of health care conditions, sanitation facilities, and health care services, with these factors being particularly accentuated in Somalia [10,33–36]. Those governments grappling with famine, conflict, and environmental pollution were confronted to resource constraints in addressing the dearth of health resources [34–37]. As one of the most fragile nations, Somalia’s health conditions continue to deteriorate due to weak governance, deficient public services, corrupt public administration, a high influx of refugees and involuntary migrants, and rapid economic decline, markedly amplifying the risk of maternal sepsis [33–39]. Additionally, Georgia, Brazil, the Russian Federation, Belarus, and Kazakhstan witnessed an estimated annual increase in ASIR of MSMI. Limited access to health care facilities, inadequate resources, and socio-economic disparities collectively contribute to the heightened burden of MSMI in LICs [10].

The age-specific distribution of deaths of MSMI exhibited a consistent pattern, characterised by elevated ASMMR in both the youngest and oldest age groups within in LMICs, which may be attributed to the heightened susceptibility of women in these age brackets to infections and complications during and after childbirth [1,40]. Presently, a prevailing trend of delayed marriage and childbearing in numerous countries and regions directly contributes to an increase in the average age of parturient [41–43]. Some policies aiming at encouraging birth have, to a certain extent, augmented the prevalence of advanced maternal age pregnancies, typically defined as pregnancies in women aged 35 and above [43,44]. The status of advanced maternal age as a risk factor for adverse maternal events is widely acknowledged [45,46]. Consistent with our findings, the MMR in the group aged 40 and above was significantly higher than in other younger age groups. This concurred with Kendle et al.’s findings, which asserted that older women have the highest likelihood of sepsis-related in-hospital mortality [47]. Furthermore, the vast majority of all age groups of MSMI burden in LMICs exhibited declining trends between 1990 and 2019. Exceptions were observed in the increasing trends of ASIR among the 10–14 years, 30–34 years, and 35–39 years group in UMCs. The gradual shift in sexual attitudes in some UMCs may partially explain these trends, but there are still deeper underlying causes warranting further exploration. Another notable concern is the apparent lack of significant improvement in ASMMR for the 10- to 20-year age group in LICs over the past three decades. These results accentuate the imperative for age-specific interventions, such as enhanced antenatal care, infection prevention and control measures, and postpartum care, to effectively address the distinctive needs and risks faced by diverse maternal age groups.

This study revealed an inverse relationship between the burden of MSMI and the SDI among 131 LMICs. This association, albeit with some exceptions, underscored those countries with lower socio-economic status, education level and insufficient awareness in health workers tend to bear a higher MSMI burden, aligning with previous epidemiological researches [10,18]. This correlation may be explicable by the limited opportunities for accessing health care services and resources in low-income environments, resulting in elevated incidence and mortality rates [10]. We also anticipated a negative correlation between the MSMI disease burden and the UHCI, a representative measure of the global average coverage of primary health care services [21]. UHC encompasses the entire spectrum of essential health services, spanning health promotion, prevention, treatment, rehabilitation, and palliative care across the life course [4,48]. However, progress towards achieving UHC, a key component of SDGs, has been insufficient. Health service coverage improvements have stagnated since 2015, and the proportion of the population facing catastrophic out-of-pocket health spending has surged [48]. Fundamental challenges persist, particularly the pervasive inequalities in health coverage, even in nations making strides in health care service coverage, with the overall data masking internal disparities, especially in low-income countries [48]. Furthermore, an observed negative correlation between the estimated annual percentage change of ASMMR and SDI in 2019 indicated that countries with lower socio-economic status experience slower progress in mitigating maternal mortality attributed to MSMI. Additionally, the estimated annual percentage change of ASIR and SDI in 2019 revealed a U-shaped correlation, peaking at 0.5–0.6. This phenomenon may be linked to the limited health care resources accessible to lower SDI countries and the lower baseline incidence rates in higher SDI countries [9,10]. As anticipated, the global estimated annual percentage change of ASIR, ASMMR, and age-standardised DALYs rates all demonstrated a negative relationship with the UHCI in 2019. These findings underscore the pressing and targeted necessity for policy interventions and investments in health care infrastructure, human resources, and health financing to enhance access to available maternal health care services, particularly in low-income settings.

To address the burden of maternal sepsis and other infections (MSMI) and improve maternal health outcomes in LMICs, a comprehensive and multifaceted approach is essential. Strengthening health care systems and infrastructure in these countries is of paramount importance, requiring increased investment and enhanced accessibility to essential medicines and supplies [10]. Simultaneously, it is crucial to enhance antenatal care and infection prevention practices, with a specific emphasis on early detection and effective management of infections [9,31]. Additionally, improving postpartum care and follow-up is equally significant, ensuring timely identification and intervention for postpartum infections [9]. Moreover, addressing the social determinants of health, including poverty and gender inequality, through gender-equitable policies and women’s empowerment initiatives, can have a substantial impact on MSMI outcomes in LMICs [48]. Strengthening health information systems and surveillance is vital for accurately tracking the burden of MSMI and guiding evidence-based decision-making. Facilitating multi-sectoral collaboration and partnerships among government agencies, non-governmental organisations (NGOs), and community-based organisations will further augment the effectiveness of interventions. Expanding coverage and improving service quality are imperative to scale up access to quality maternal health care services in LMICs [10]. Investment in research and innovation is necessary to identify and implement effective strategies and technologies for preventing and managing MSMI. Empowering women and involving communities in decision-making and health care planning are critical components of comprehensive interventions. Regular monitoring and evaluation of interventions will enable the assessment of their effectiveness and facilitate necessary adjustments [48]. By implementing these recommendations, tangible reductions in the burden of MSMI may be achieved, leading to improved maternal health outcomes and the realisation of global maternal health goals. However, it is essential to underscore the ongoing need for sustained efforts and investments to maintain progress in addressing MSMI and promoting maternal health in LMICs.

Focusing on LMICs is what distinguishes this study from other conventional GBD studies. As mentioned elsewhere, the GBD study has some limitations. First, besides data extracted from vital registration, verbal autopsy, registry, survey, police report, some unavailable location or year data relied on model estimates, which may underestimate the real disease burden of MSMI. Second, due to the characteristics of GBD 2019, our results are presented in limited depth and cannot be subdivided into the aetiology or pathogens of MSMI. Third, the use of the EAPC for each age-standardised rate and the relative percent change in the number of events to assess secular trends over the three decades from 1990 to 2019 may have obscured dynamic processes due to some preventive measures. Finally, because of the special characteristics of pregnant women and the trend of late marriage and late childbearing, we suggest that the world, especially LMICs, should pay more attention to MSMI to protect the health of pregnant women and their children, and promote social stability.

## CONCLUSIONS

Although progress has been made in reducing the burden of MSMI in 131 LMICs, the disease burden in LICs far exceeded that of LMCs and UMCs. Socio-economic status and universal health coverage were both associated with the MSMI burden, and further research is needed to explore the underlying factors contributing to these disparities and to identify effective strategies for reducing the disparities in disease burden. Our findings provide valuable insights into the burden of MSMI in LMICs, which can inform policy and programmatic interventions to improve maternal health outcomes.

## Additional material


Online Supplementary Document

## References

[1] Kourtis AP Read JS Jamieson DJ Pregnancy and infection. N Engl J Med. 2014;370:2211–8. 10.1056/NEJMra1213566 24897084 PMC4459512

[2] World Health Organization. Statement on maternal sepsis. 2017. Available: http://apps.who.int/iris/handle/10665/254608. Accessed: 23 November 2023.

[3] Singer M Deutschman CS Seymour CW Shankar-Hari M Annane D Bauer M The Third International Consensus Definitions for Sepsis and Septic Shock (Sepsis-3). JAMA. 2016;315:801–10. 10.1001/jama.2016.0287 26903338 PMC4968574

[4] The United Nations. Sustainable Development Goals. 2015. Available: https://unstats.un.org/wiki/display/SDGeHandbook/Indicator+3.2.2. Accessed: 21 December 2023.

[5] GBD 2015 Maternal Mortality Collaborators Global, regional, and national levels of maternal mortality, 1990-2015: a systematic analysis for the Global Burden of Disease Study 2015. Lancet. 2016;388:1775–812. 10.1016/S0140-6736(16)31470-2 27733286 PMC5224694

[6] Liu J Jing W Liu M Risk management of pregnant women and the associated low maternal mortality from 2008-2017 in China: a national longitude study. BMC Health Serv Res. 2022;22:335. 10.1186/s12913-022-07721-z 35287680 PMC8920427

[7] Say L Chou D Gemmill A Tunçalp Ö Moller AB Daniels J Global causes of maternal death: a WHO systematic analysis. Lancet Glob Health. 2014;2:e323–33. 10.1016/S2214-109X(14)70227-X 25103301

[8] WHO Global Maternal Sepsis Study (GLOSS) Research Group Frequency and management of maternal infection in health facilities in 52 countries (GLOSS): a 1-week inception cohort study. Lancet Glob Health. 2020;8:e661–71. 10.1016/S2214-109X(20)30109-1 32353314 PMC7196885

[9] Brizuela V Cuesta C Bartolelli G Abdosh AA Abou Malham S Assarag B Availability of facility resources and services and infection-related maternal outcomes in the WHO Global Maternal Sepsis Study: a cross-sectional study. Lancet Glob Health. 2021;9:e1252–61. 10.1016/S2214-109X(21)00248-5 34273300 PMC8370881

[10] Maswime S Buga E Surviving maternal sepsis in low-income countries. Lancet Glob Health. 2021;9:e1183–4. 10.1016/S2214-109X(21)00294-1 34273301

[11] Mor G Cardenas I The immune system in pregnancy: a unique complexity. Am J Reprod Immunol. 2010;63:425–33. 10.1111/j.1600-0897.2010.00836.x 20367629 PMC3025805

[12] Cardenas I Aldo P Koga K Means R Lang S Mor G Subclinical viral infection in pregnancy lead to inflammatory process at the placenta with non-lethal fetal damage. Am J Reprod Immunol. 2009;61:397–402.

[13] Reinhart K Daniels R Kissoon N Machado FR Schachter RD Finfer S Recognizing Sepsis as a Global Health Priority - A WHO Resolution. N Engl J Med. 2017;377:414–7. 10.1056/NEJMp1707170 28658587

[14] The United Nations. Millennium Development Goals. 2000. Available: https://www.un.org/zh/millenniumgoals/archive_2012.shtml. Accessed: 29 November 2023.

[15] Yamin AE Boulanger VM Embedding sexual and reproductive health and rights in a transformational development framework: lessons learned from the MDG targets and indicators. Reprod Health Matters. 2013;21:74–85. 10.1016/S0968-8080(13)42727-1 24315065

[16] Abdu M Wilson A Mhango C Taki F Coomarasamy A Lissauer D Resource availability for the management of maternal sepsis in Malawi, other low-income countries, and lower-middle-income countries. Int J Gynaecol Obstet. 2018;140:175–83. 10.1002/ijgo.12350 29027207

[17] GBD 2019 Diseases and Injuries Collaborators Global burden of 369 diseases and injuries in 204 countries and territories, 1990-2019: a systematic analysis for the Global Burden of Disease Study 2019. Lancet. 2020;396:1204–22. 10.1016/S0140-6736(20)30925-9 33069326 PMC7567026

[18] Chen L Wang Q Gao Y Zhang J Cheng S Chen H The global burden and trends of maternal sepsis and other maternal infections in 204 countries and territories from 1990 to 2019. BMC Infect Dis. 2021;21:1074. 10.1186/s12879-021-06779-0 34663264 PMC8524924

[19] The World Bnak. World Bank Group country classifications by income level for FY24 (July 1, 2023- June 30, 2024). 2023. Available: https://blogs.worldbank.org/opendata/new-world-bank-group-country-classifications-income-level-fy24. Accessed: 29 November 2023.

[20] The World Bnak. World Bank Country and Lending Groups. 2023. Available: https://datahelpdesk.worldbank.org/knowledgebase/articles/906519-world-bank-country-and-lending-groups. Accessed: 29 November 2023.

[21] Global Burden of Disease. 2019. Global Burden of Disease Study 2019 (GBD 2019) UHC Effective Coverage Index 1990-2019. 2023. Available: https://ghdx.healthdata.org/record/ihme-data/gbd-2019-uhc-effective-coverage-index-1990-2019. Accessed: 30 November 2023.

[22] Global Burden of Disease. 2019. Global Burden of Disease Study 2019 (GBD 2019) Socio-Demographic Index (SDI) 1950–2019. 2023. Available: https://ghdx.healthdata.org/record/ihme-data/gbd-2019-socio-demographic-index-sdi-1950-2019. Accessed: 30 November 2023.

[23] Culyer AJ. Encyclopedia of health economics: Newnes; 2014.

[24] Collaborators GMaCoD Global, regional, and national age-sex specific all-cause and cause-specific mortality for 240 causes of death, 1990-2013: a systematic analysis for the Global Burden of Disease Study 2013. Lancet. 2015;385:117–71. 10.1016/S0140-6736(14)61682-2 25530442 PMC4340604

[25] GBD 2019 Universal Health Coverage Collaborators Measuring universal health coverage based on an index of effective coverage of health services in 204 countries and territories, 1990-2019: a systematic analysis for the Global Burden of Disease Study 2019. Lancet. 2020;396:1250–84. 10.1016/S0140-6736(20)30750-9 32861314 PMC7562819

[26] GBD 2017 Disease and Injury Incidence and Prevalence Collaborators Global, regional, and national incidence, prevalence, and years lived with disability for 354 diseases and injuries for 195 countries and territories, 1990-2017: a systematic analysis for the Global Burden of Disease Study 2017. Lancet. 2018;392:1789–858. 10.1016/S0140-6736(18)32279-7 30496104 PMC6227754

[27] GBD 2017 Causes of Death Collaborators Global, regional, and national age-sex-specific mortality for 282 causes of death in 195 countries and territories, 1980-2017: a systematic analysis for the Global Burden of Disease Study 2017. Lancet. 2018;392:1736–88. 10.1016/S0140-6736(18)32203-7 30496103 PMC6227606

[28] Jin X Ren J Li R Gao Y Zhang H Li J Global burden of upper respiratory infections in 204 countries and territories, from 1990 to 2019. EClinicalMedicine. 2021;37:100986. 10.1016/j.eclinm.2021.100986 34386754 PMC8343248

[29] WHO Global Maternal Sepsis Study (GLOSS) Frequency and management of maternal infection in health facilities in 52 countries (GLOSS): a 1-week inception cohort study. Lancet Glob Health. 2020;8:e661–71. 10.1016/S2214-109X(20)30109-1 32353314 PMC7196885

[30] Alkema L Chou D Hogan D Zhang S Moller AB Gemmill A Global, regional, and national levels and trends in maternal mortality between 1990 and 2015, with scenario-based projections to 2030: a systematic analysis by the UN Maternal Mortality Estimation Inter-Agency Group. Lancet. 2016;387:462–74. 10.1016/S0140-6736(15)00838-7 26584737 PMC5515236

[31] Shields A de Assis V Halscott T Top 10 Pearls for the Recognition, Evaluation, and Management of Maternal Sepsis. Obstet Gynecol. 2021;138:289–304. 10.1097/AOG.0000000000004471 34237760 PMC8288480

[32] Abdu M Wilson A Mhango C Taki F Coomarasamy A Lissauer D Resource availability for the management of maternal sepsis in Malawi, other low-income countries, and lower-middle-income countries. Int J Gynaecol Obstet. 2018;140:175–83. 10.1002/ijgo.12350 29027207

[33] Gele A Challenges Facing the Health System in Somalia and Implications for Achieving the SDGs. European Journal of Public Health. 2020;30(Supplement_5). 10.1093/eurpub/ckaa165.1147

[34] Lalla AT Ginsbach KF Penney N Shamsudin A Oka R Exploring sources of insecurity for Ethiopian Oromo and Somali women who have given birth in Kakuma Refugee Camp: A Qualitative Study. PLoS Med. 2020;17:e1003066. 10.1371/journal.pmed.1003066 32208416 PMC7092956

[35] Ahrne M Schytt E Andersson E Small R Adan A Essén B Antenatal care for Somali-born women in Sweden: Perspectives from mothers, fathers and midwives. Midwifery. 2019;74:107–15. 10.1016/j.midw.2019.03.022 30953966

[36] Habib M Ludwig S Lange U Prazeres da Costa C The impact of the COVID-19 pandemic on pregnancy, birth and sexual & reproductive health and rights: Perspectives from Germany and Somalia. J Glob Health. 2021;11:03085. 10.7189/jogh.11.03085 34552715 PMC8442510

[37] Topp SM Abimbola S Joshi R Negin J How to assess and prepare health systems in low- and middle-income countries for integration of services-a systematic review. Health Policy Plan. 2018;33:298–312. 10.1093/heapol/czx169 29272396 PMC5886169

[38] The World Bank. The World Bank in Somalia. 2023. Available: https://www.worldbank.org/en/country/somalia/overview. Accessed: 21 December 2023.

[39] Vanda Felbab-Brown. Somalia’s chanllenges in 2023. 2023. Available: https://www.brookings.edu/articles/somalias-challenges-in-2023/. Accessed: 21 December 2023.

[40] Sharma S Rodrigues PRS Zaher S Davies LC Ghazal P Immune-metabolic adaptations in pregnancy: A potential stepping-stone to sepsis. EBioMedicine. 2022;86:104337. 10.1016/j.ebiom.2022.104337 36470829 PMC9782817

[41] Inhorn MC, Smith-Hefner NJ. Waithood: Gender, education, and global delays in marriage and childbearing: Berghahn Books; 2020.

[42] Jones GW Delayed marriage and very low fertility in Pacific Asia. Popul Dev Rev. 2007;33:453–78. 10.1111/j.1728-4457.2007.00180.x

[43] Kang L Jing W Liu J Ma Q Zhang S Liu M The prevalence of barriers to rearing children aged 0-3 years following China’s new three-child policy: a national cross-sectional study. BMC Public Health. 2022;22:489. 10.1186/s12889-022-12880-z 35279114 PMC8917473

[44] Xinhua News Agency. China's three-child policy to improve demographic structure. 2021. Available: https://english.www.gov.cn/statecouncil/ministries/202106/01/content_WS60b61ab7c6d0df57f98da86e.html. Accessed: 30 November 2023.

[45] Molina-García L Hidalgo-Ruiz M Arredondo-López B Colomino-Ceprián S Delgado-Rodríguez M Martínez-Galiano JM Maternal Age and Pregnancy, Childbirth and the Puerperium: Obstetric Results. J Clin Med. 2019;8:672. 10.3390/jcm8050672 31086046 PMC6571680

[46] Saccone G Gragnano E Ilardi B Marrone V Strina I Venturella R Maternal and perinatal complications according to maternal age: A systematic review and meta-analysis. Int J Gynaecol Obstet. 2022;159:43–55. 10.1002/ijgo.14100 35044694 PMC9543904

[47] Kendle AM Salemi JL Tanner JP Louis JM Delivery-associated sepsis: trends in prevalence and mortality. Am J Obstet Gynecol. 2019;220:391.e1–.e16. 10.1016/j.ajog.2019.02.002 30786257

[48] World Health Organization. Universal health coverage (UHC). 2023. Available: https://www.who.int/news-room/fact-sheets/detail/universal-health-coverage-(uhc). Accessed: 30 November 2023.

