# Supplementary material for: Trends and disparities of disease burden in infections among pregnant women in 131 low-income and middle-income countries, 1990–2019
Source: J Glob Health. 2024 Sep 6;14:04130. doi: 10.7189/jogh.14.04130 (PMC11377970; doi:10.7189/jogh.14.04130)
Supplement: Online Supplementary Document [file jogh-14-04130-s001.pdf]

Online Supplementary Document

Qin et al. Trends and disparities of disease burden in infections among pregnant women in 131 low-income and middle-income countries, 1990-2019

CONTENTS

Supplementary Materials **4**

Supplementary Methods **5**

1. GBD Overview 5

2. Definition of Maternal sepsis and other maternal infections (MSMI) 6

3. Estimation of MSMI in GBD 2019 6

3.1 Input data 6

3.2 Data processing 7

3.3 Modelling strategy 8

3.4 Incidence estimation 8

3.5 Uncertainty and model selection 8

4. Data analysis 9

5. Reference 9

Supplementary Tables and Figures **11**

Table S1. 131 LMICs as per Word Bank Classification (2019) † 11

Table S2. The SDI and UHCI of 131 low- and middle-income countries and territories in 2019. 13

Table S3. The global SDI from 1990 to 2019. 17

Table S4. Incidence of MSMI in 131 low- and middle-income countries and territories with EAPC from 1990 to 2019. 18

Table S5. MMR of MSMI in 131 low- and middle-income countries and territories with EAPC from 1990 to 2019. 33

Table S6. DALYs of MSMI in 131 low- and middle-income countries with EAPC from 1990 and 2019. 51

Table S7. Incidence of MSMI in 131 low- and middle-income countries and territories with EAPC in age group of 10-14 years from 1990 to 2019. 70

Table S8. Incidence of MSMI in 131 low- and middle-income countries and territories with EAPC in age group of 15-19 years from 1990 to 2019. 84

Table S9. Incidence of MSMI in 131 low- and middle-income countries and territories with EAPC in age group of 20-24 years from 1990 and 2019. 104

Table S10. Incidence of MSMI in 131 low- and middle-income countries and territories with EAPC in age group of 25-29 years from 1990 and 2019. 119

Table S11. Incidence of MSMI in 131 low- and middle-income countries and territories with EAPC in age group of 30-34 years from 1990 and 2019. 137

Table S12. Incidence of MSMI in 131 low- and middle-income countries and territories with EAPC in age group of 35-39 years from 1990 and 2019. 155

Table S13. Incidence of MSMI in 131 low- and middle-income countries and territories with EAPC in age group of 40-44 years from 1990 and 2019. 172

Table S14. Incidence of MSMI in 131 low- and middle-income countries and territories with EAPC in age group of 45-49 years from 1990 and 2019. 187

Table S15. Incidence of MSMI in 131 low- and middle-income countries and territories with EAPC in age group of 50-54 years from 1990 and 2019. 204

Table S16. MMR of MSMI in 131 low- and middle-income countries and territories with EAPC in age group of 0-14 years from 1990 to 2019....219

Table S17. MMR of MSMI in 131 low- and middle-income countries and territories with EAPC in age group of 15-19 years from 1990 and 2019....234

Table S18. MMR of MSMI in 131 low- and middle-income countries and territories with EAPC in age group of 20-24 years from 1990 and 2019....250

Table S19. MMR of MSMI in 131 low- and middle-income countries and territories with EAPC in age group of 25-29 years from 1990 and 2019....265

Table S20. MMR of MSMI in 131 low- and middle-income countries and territories with EAPC in age group of 30-34 years from 1990 and 2019....280

Table S21. MMR of MSMI in 131 low- and middle-income countries and territories with EAPC in age group of 35-39 years from 1990 and 2019....299

Table S22. MMR of MSMI in 131 low- and middle-income countries and territories with EAPC in age group of 40-44 years from 1990 and 2019....315

Table S23. MMR of MSMI in 131 low- and middle-income countries and territories with EAPC in age group of 45-49 years from 1990 and 2019....330

Table S24. MMR of MSMI in 131 low- and middle-income countries and territories with EAPC in age group of 50-54 years from 1990 and 2019....345

Table S25. DALYs of MSMI in 131 low- and middle-income countries and territories with EAPC in age group of 10-14 years from 1990 and 2019....359

Table S26. DALYs of MSMI in 131 low- and middle-income countries and territories with EAPC in age group of 15-19 years from 1990 and 2019....375

Table S27. DALYs of MSMI in 131 low- and middle-income countries and territories with EAPC in age group of 20-24 years from 1990 and 2019....391

Table S28. DALYs of MSMI in 131 low- and middle-income countries and territories with EAPC in age group of 25-29 years from 1990 and 2019....407

Table S29. DALYs of MSMI in 131 low- and middle-income countries and territories with EAPC in age group of 30-34 years from 1990 and 2019....423

Table S30. DALYs of MSMI in 131 low- and middle-income countries and territories with EAPC in age group of 35-39 years from 1990 and 2019. 439

Table S31. DALYs of MSMI in 131 low- and middle-income countries and territories with EAPC in age group of 40-44 years from 1990 and 2019. 455

Table S32. DALYs of MSMI in 131 low- and middle-income countries and territories with EAPC in age group of 45-49 years from 1990 and 2019. 470

Table S33. DALYs of MSMI in 131 low- and middle-income countries and territories with EAPC in age group of 50-54 years from 1990 and 2019. 486

Figure S1. National trends in the deaths of MSMI in 131 low- and middle-income countries and territories. 504

Figure S2. National trends in the DALYs of MSMI in 131 low- and middle-income countries and territories. 504

Figure S3. ASIR (A), ASMMR (B), and age-standardized DALY rates (C) for MSMSI globally by SDI, 1990–2019. 505

Figure S4. The correlation between the EAPC of ASIR/ASMMR/ age-standardized DALY rates of MSMI and SDI (in 2019) in LICs, LMCs, and UMCs. 506

Figure S5. The correlation between the EAPC of ASIR/ASMMR/ age-standardized DALY rates of MSMI and UHCI (in 2019) in LICs, LMCs, and UMCs. 508

Online Supplementary Document

Supplementary Methods

1. GBD Overview

The Global Burden of Diseases, Injuries, and Risk Factors Study (GBD) provides a systematic scientific assessment of published, publicly available, and contributed data on global descriptive epidemiology. It estimates incidence, prevalence, mortality, years of life lost (YLLs), years lived with disability (YLDs), and disability-adjusted life-years (DALYs) due to 369 diseases and injuries, for two sexes, and for 204 countries and territories in the past decades. The numbers and rates of incidence, prevalence, years lived with disability (YLDs), and disability-adjusted life-years (DALYs) were estimated for the years 1990–2019, while deaths and years of life lost (YLLs) were estimated for 1980–2019. The cause and sequelae list were based on input from the Scientific Council and GBD collaborator network.

GBD Input data were extracted from censuses, household surveys, civil registration and vital statistics, disease registries, health service use, air pollution monitors, satellite imaging, disease notifications, and other sources. Cause-specific death rates and cause fractions were calculated using the Cause of Death Ensemble model and spatiotemporal Gaussian process regression. Cause-specific deaths were adjusted to match the total all-cause deaths calculated as part of the GBD population, fertility, and mortality estimates. Deaths were multiplied by standard life expectancy at each age to calculate YLLs. A Bayesian meta-regression modelling tool, DisMod-MR 2.1, was used to ensure consistency between incidence, prevalence, remission, excess mortality, and cause-specific mortality for most causes. Prevalence estimates were multiplied by disability weights for mutually exclusive sequelae of diseases and injuries to calculate YLDs. We considered results in the context of the Socio-demographic Index (SDI), a composite indicator of income per capita, years of schooling, and fertility rate in females younger than 25 years. Uncertainty intervals (UIs) were generated for every metric using the 25th and 975th ordered 1000 draw values of the posterior distribution.

2. Definition of Maternal sepsis and other maternal infections (MSMI)

The GBD cause list is organized hierarchically. Levels 1 and 2 aggregate causes into general groupings. There are four cause groups at level 1: Communicable, maternal, neonatal, and nutritional diseases. The "maternal disorders" group which includes disability due to maternal causes, is at Level 3 under level 2 of "Maternal and neonatal disorders." Level 4 includes 7 groups including 1) Abortion and miscarriage; 2) Ectopic pregnancy; 3) Obstructed labour and uterine rupture;4) Maternal haemorrhage; 5) Maternal sepsis and other maternal infections; 6) Maternal hypertensive disorders; and 7) Other [direct] maternal disorders. Indirect maternal disorders, late maternal deaths, and maternal deaths aggravated by HIV/AIDS did not have any estimated disability based on the premise that it is captured in the respective underlying causes.

Maternal sepsis and other maternal infections：

a. Maternal sepsis is defined as a temperature <36°C or >38°C and clinical signs of shock including systolic blood pressure <90 mmHg and tachycardia >120 bpm;

b. Other maternal infections are defined as any maternal infections excluding HIV, sexually transmitted infections, or are not believed to have epidemiologic relationship with pregnancy. Examples include urinary tract infections, mastitis, candidiasis, and bacterial vaginosis during pregnancy.

3. Estimation of MSMI in GBD 2019

3.1 Input data

Systematic literature reviews have been completed on May 10, 2019. In addition, GBD searched ministry of health websites for pregnancy complication data and used Confidential Enquiry and other sources used in our maternal mortality analyses when they presented data on pregnancy complications. GBD also performed snowball searches for abortion reporting and surveillance data systems, finding multiple such systems throughout high-income countries and several geographies in Central and Eastern Europe. Inpatient and outpatient data were used, as were claims data from Taiwan and Singapore as well as MarketScan in the United States.All data were either extracted as incidence ratio

(number of events / live birth) or, if data were only available with population as the denominator, they were converted to incidence ratio using GBD 2019 age-specific fertility rate (number of live births / population). All data were extracted in standard fashion, and were uploaded and stored on a centralised SQL database.

In locations with low-quality, or no vital registration, maternal mortality metrics can be found in surveillance, surveys, census, and sibling history data sources. The best data have death counts due to maternal causes and the total number of deaths for women within the reproductive ages of 10–54 by year. If a data source is missing these components, creating a complete cause list is necessary by using live births and all-cause mortality deaths. Though death counts are the preferred metric, maternal mortality is often measured by using the maternal mortality ratio (MMR), which is easily converted to deaths by using live births. The China Maternal and Child Surveillance data is adjusted by scaling data from the strata to the province level.

3.2 Data processing

The first step of data processing was age-sex splitting. For any datum that did not entirely fit within a GBD age group or was for both sexes combined, the observation was split to be multiple age specific and sex-specific data points based on the age and sex pattern predicted by GBD 2017 DisModMR 2.1 models.

The second step was crosswalking all data from alternate to reference definitions. For all other models, we adjusted data to the reference category for each cause by age using Meta-Regression-Bayesian, Regularized, Trimmed (MR-BRT), a meta-analytic tool developed for GBD 2019. In accordance with GBD 2019 principles for data processing, to make data comparable, staffs of GBD began by evaluating the number of observations of each alternate definition that matched with a corresponding observation from the reference definition. They excluded some alternative definitions from this process, e.g. studies reporting chronic hypertension and studies reporting severe diagnoses of maternal disorders except for sepsis and eclampsia. The standard error of the ratio was calculated using the delta method. The details of each of the crosswalks are described below. All data sources that only reported event rates for severe maternal morbidity or "near miss" were excluded as a reliable crosswalk model could not be developed.

More specifically, puerperal sepsis cases reported in literature studies were the reference category. We crosswalked claims data to inpatient data by age. After this adjustment we crosswalked all of the clinical data to the literature data by age. The age pattern for the claims to inpatient crosswalk was significant with an increase with age until age 40. The age pattern of clinical to literature was slightly decreasing with age. Inpatient hospital data were the reference for other maternal infections. We crosswalked claims data to inpatient hospital data by age. The age pattern shows a steep increase in the ratio from ages 10 to 35.

3.3 Modelling strategy

Incidence ratio for each age-sex-location-year in the GBD 2019 location hierarchy were estimated using DisMod-MR 2.1. A series of country covariates were chosen to help drive the magnitude of estimates in areas of sparse or absent data. GBD staffs included the respective log transformed maternal mortality ratio (MMR) for each maternal disorder that was estimated as a country level covariate for almost every model. Puerperal sepsis used the log transformed age standardized death rate (LN-ASDR) as a covariate, instead of MMR. No specific age or slope priors were used. All models were run with a time window of five years.

3.4 Incidence estimation

All age-specific ratios were then converted to incidence rates by multiplying by live births per population. Maternal sepsis was assigned a duration of five days (+/-2) and, based on the same data identified in our review of pelvic inflammatory disease, 9% (7.7% - 10%) of incident cases of puerperal sepsis were estimated to continue on to have secondary infertility due to maternal sepsis. GBD staffs apply this proportion to the incidence results of puerperal sepsis and use them as input data for a full compartment DisMod-MR 2.1 model. Other maternal infections were assigned a wide potential duration of 15 to 45 days (mean 30).

3.5 Uncertainty and model selection

For all maternal disorders, uncertainty bounds include uncertainty due to input data, crosswalks from non-reference definitions, uncertainty in numerical solutions (posteriors) of each DisMod-MR 2.1 model. In consultation with GBD researchers and

collaborators, final models were selected on a combination of qualitative and quantitative goodness of fit to input data, plausibility of geographic and temporal trends, consistency of age pattern, and, when available, comparison with other published studies on the epidemiology of pregnancy complications. Directionality, magnitude, and plausibility of study-level and country-level covariates were also considered in the process of model development. Of note, due to the nature of statistical modelling, final results do not always cover the values reported in input data.

4. Data analysis

ASIRs were calculated on the basis of the following formula:

$$ASIR = \frac{\sum_{i=1}^{A} a_i w_i}{\sum_{i=1}^{A} w_i} \times 100{,}000$$

The ASIR (per 100,000 population) is equal to the sum of the product of the specific age ratio (ai) in age group i and the number (or weight) (wi) of the selected reference standard population group i divided by the sum of number (or weight) of the standard population, i.e.

Meanwhile, EAPC, which is approximately equal to the annual change for a specified range, was calculated using the following regression model to assess the trends in ASIR: $Y=\alpha+\beta X+\varepsilon$, where y refers to ln(ASIR), x represents calendar year, ε means error term, and β determines the positive or negative trends in ASIR. The EAPC could be given by 100*(exp(β)-1), as well as its 95% confidence interval (CI).

Disability adjusted life-years were also defined as years of healthy life lost, estimated by the sum of the YLLs and the YLDs. YLLs, the years of life lost due to premature mortality, are the multiplication of deaths and a standard life expectancy at each age of death. In each population greater than 5 million, the standard life expectancy was estimated from a life table which includes the lowest observed mortality at each age group. YLDs were defined as the years lived with any short-term or long-term health loss weighted for severity by the disability weights, calculated by multiplying prevalence estimate and the disability weight of each mutually exclusive sequela for prostate cancer, such as disability due to incontinence and impotence from prostatectomy.

5. Reference

GBD 2019 Diseases and Injuries Collaborators. Global burden of 369 diseases and injuries in 204 countries and territories, 1990-2019: a systematic analysis for the Global Burden of Disease Study 2019. Lancet 2020; 396(10258): 1204-22.

Supplementary Tables and Figures

Table S1. 131 LMICs as per Word Bank Classification (2019) †

Word Bank income levels	Number	Countries
Low-income countries	29	Afghanistan; Burkina Faso; Burundi; Central African Republic; Chad; Democratic People's Republic of Korea; Democratic Republic of the Congo; Eritrea; Ethiopia; Gambia; Guinea; Guinea-Bissau; Haiti; Liberia; Madagascar; Malawi; Mali; Mozambique; Niger; Rwanda; Sierra Leone; Somalia; South Sudan; Sudan; Syrian Arab Republic; Tajikistan; Togo; Uganda; Yemen
Lower-middle-income countries	48	Algeria; Angola; Bangladesh; Benin; Bhutan; Bolivia (Plurinational State of); Cabo Verde; Cambodia; Cameroon; Comoros; Congo; Côte d'Ivoire; Djibouti; Egypt; El Salvador; Eswatini; Ghana; Honduras; India; Kenya; Kiribati; Kyrgyzstan; Lao People's Democratic Republic; Lesotho; Mauritania; Micronesia (Federated States of); Mongolia; Morocco; Myanmar; Nepal; Nicaragua; Nigeria; Pakistan; Papua New Guinea; Philippines; Republic of Moldova; Senegal; Solomon Islands; Sri Lanka; Timor-Leste; Tunisia; Ukraine; United Republic of Tanzania; Uzbekistan; Vanuatu; Viet Nam; Zambia; Zimbabwe
Upper-middle-income countries	54	Albania; American Samoa; Armenia; Azerbaijan; Belarus; Belize; Bosnia and Herzegovina; Botswana; Brazil; Bulgaria; China; Colombia; Costa Rica; Cuba; Dominica; Dominican Republic; Ecuador; Equatorial Guinea; Fiji; Gabon; Georgia; Grenada; Guatemala; Guyana; Indonesia; Iran (Islamic Republic of); Iraq; Jamaica; Jordan; Kazakhstan; Lebanon; Libya; Malaysia; Maldives; Marshall Islands; Mexico; Montenegro; Namibia; North Macedonia; Paraguay; Peru; Russian Federation; Saint Lucia; Saint Vincent and the Grenadines; Samoa; Serbia; South Africa; Suriname; Thailand; Tonga; Turkey; Turkmenistan; Tuvalu; Venezuela (Bolivarian Republic of)

Note: LMICs – low- and middle-income countries.

† According to World Bank Group country classifications by income level (https://datahelpdesk.worldbank.org/knowledgebase/articles/906519-world-bank-country-and-lending-groups), three economies shift from low-income countries (2019) to lower-middle-income countries (2022), two economies shift from lower-middle-income countries (2019) to upper-middle income countries (2022), two economies shift from upper-middle-income countries (2019) to high-income countries (2022), and four economies shift from upper-middle-income countries (2019) to lower-middle-income countries (2022), and Venezuela (Bolivarian Republic of) has no data for 2022.

Table S2. The SDI and UHCI of 131 low- and middle-income countries and territories in 2019.

Order	Location	SDI in 2019	UHCI in 2019
1	Afghanistan	0.343	39.2945
2	Albania	0.681	69.6255
3	Algeria	0.652	64.8553
4	American Samoa	0.712	53.1971
5	Angola	0.47	39.1577
6	Armenia	0.689	62.4351
7	Azerbaijan	0.683	48.1814
8	Bangladesh	0.483	53.8827
9	Belarus	0.745	70.4622
10	Belize	0.603	54.2797
11	Benin	0.352	44.6243
12	Bhutan	0.455	51.3014
13	Bolivia (Plurinational State of)	0.566	52.3988
14	Bosnia and Herzegovina	0.718	64.1839
15	Botswana	0.634	57.5176
16	Brazil	0.64	64.8276
17	Bulgaria	0.764	62.5567
18	Burkina Faso	0.257	41.7968
19	Burundi	0.284	49.94
20	Cabo Verde	0.525	62.188
21	Cambodia	0.469	57.0794
22	Cameroon	0.49	42.29
23	Central African Republic	0.274	22.2999
24	Chad	0.238	31.3724
25	China	0.686	69.7123
26	Colombia	0.633	74.3969
27	Comoros	0.455	48.1389
28	Congo	0.568	43.9038
29	Costa Rica	0.68	79.015
30	Cuba	0.668	72.5922
31	Côte d'Ivoire	0.408	43.0401
32	Democratic People's Republic of Korea	0.878	52.8378

33	Democratic Republic of the Congo	0.382	45.1687
34	Djibouti	0.459	45.2867
35	Dominica	0.729	51.8089
36	Dominican Republic	0.592	52.4998
37	Ecuador	0.64	64.4544
38	Egypt	0.658	54.7965
39	El Salvador	0.573	61.6782
40	Equatorial Guinea	0.685	49.9937
41	Eritrea	0.396	42.275
42	Eswatini	0.577	53.3968
43	Ethiopia	0.343	46.5216
44	Fiji	0.664	45.1761
45	Gabon	0.656	53.0498
46	Gambia	0.399	48.065
47	Georgia	0.841	55.9534
48	Ghana	0.557	49.1393
49	Grenada	0.669	50.4838
50	Guatemala	0.526	52.0988
51	Guinea	0.325	32.3346
52	Guinea-Bissau	0.355	35.7073
53	Guyana	0.618	40.6219
54	Haiti	0.432	35.8119
55	Honduras	0.496	54.2843
56	India	0.566	46.8261
57	Indonesia	0.66	48.7277
58	Iran (Islamic Republic of)	0.67	69.5146
59	Iraq	0.671	57.7248
60	Jamaica	0.684	56.8395
61	Jordan	0.731	69.9667
62	Kazakhstan	0.723	59.2368
63	Kenya	0.508	51.6469
64	Kiribati	0.527	35.7356
65	Kyrgyzstan	0.596	52.9515
66	Lao People's Democratic Republic	0.49	43.8545
67	Lebanon	0.708	74.5332
68	Lesotho	0.507	38.7373
69	Liberia	0.37	47.5997

70	Libya	0.709	66.3277
71	Madagascar	0.396	39.6908
72	Malawi	0.384	55.5208
73	Malaysia	0.737	66.5736
74	Maldives	0.562	66.8566
75	Mali	0.263	40.6607
76	Marshall Islands	0.544	44.0043
77	Mauritania	0.496	53.2779
78	Mexico	0.649	61.4368
79	Micronesia (Federated States of)	0.58	34.4743
80	Mongolia	0.606	47.907
81	Montenegro	0.791	65.9585
82	Morocco	0.548	58.0323
83	Mozambique	0.307	44.044
84	Myanmar	0.521	46.9541
85	Namibia	0.612	62.169
86	Nepal	0.422	47.2804
87	Nicaragua	0.517	57.1586
88	Niger	0.162	35.0256
89	Nigeria	0.515	38.339
90	North Macedonia	0.744	60.7461
91	Pakistan	0.449	39.1679
92	Papua New Guinea	0.394	37.7673
93	Paraguay	0.638	63.3513
94	Peru	0.648	75.7594
95	Philippines	0.623	54.7117
96	Republic of Moldova	0.696	62.1936
97	Russian Federation	0.805	68.9736
98	Rwanda	0.429	59.3591
99	Saint Lucia	0.67	59.1403
100	Saint Vincent and the Grenadines	0.627	49.4939
101	Samoa	0.641	49.7959
102	Senegal	0.389	49.6099
103	Serbia	0.767	63.3494
104	Sierra Leone	0.347	42.1196
105	Solomon Islands	0.407	39.3331
106	Somalia	0.081	23.9397

107	South Africa	0.678	59.7274
108	South Sudan	0.363	41.6941
109	Sri Lanka	0.69	65.5627
110	Sudan	0.515	51.8347
111	Suriname	0.636	50.1342
112	Syrian Arab Republic	0.619	57.5646
113	Tajikistan	0.539	47.8775
114	Thailand	0.687	71.6003
115	Timor-Leste	0.514	45.9543
116	Togo	0.417	42.8086
117	Tonga	0.636	52.4167
118	Tunisia	0.672	68.1057
119	Turkey	0.748	69.2104
120	Turkmenistan	0.67	44.0134
121	Tuvalu	0.589	39.5697
122	Uganda	0.404	52.7479
123	Ukraine	0.736	56.7521
124	United Republic of Tanzania	0.423	55.2493
125	Uzbekistan	0.631	42.1848
126	Vanuatu	0.485	34.0835
127	Venezuela (Bolivarian Republic of)	0.607	60.9675
128	Viet Nam	0.617	59.7068
129	Yemen	0.412	49.0458
130	Zambia	0.505	52.6937
131	Zimbabwe	0.476	54.4611

SDI – sociodemographic index, UHCI – universal health coverage effective coverage index.

Table S3. The global SDI from 1990 to 2019.

Year	Global SDI
1990	0.511
1991	0.516
1992	0.521
1993	0.525
1994	0.529
1995	0.534
1996	0.538
1997	0.542
1998	0.547
1999	0.551
2000	0.556
2001	0.561
2002	0.566
2003	0.571
2004	0.576
2005	0.581
2006	0.586
2007	0.591
2008	0.596
2009	0.601
2010	0.607
2011	0.612
2012	0.616
2013	0.621
2014	0.626
2015	0.631
2016	0.635
2017	0.641
2018	0.647
2019	0.651

SDI – sociodemographic index.

Table S4. Incidence of MSMI in 131 low- and middle-income countries and territories with EAPC from 1990 to 2019.

Location	Incident cases in 1990 (95% UI)	Incident cases in 2019 (95% UI)	Relative change (%, 95 CI)	ASIR in 1990 (95% UI)	ASIR in 2019 (95% UI)	EAPC (%, 95 CI)
Global	23029128 (17399801 to 29084560)	20569889 (15688621 to 25972496)	-10.68% (-14.28 to -5.76)	787.04 (595.71 to 990.76)	534.21 (407.34 to 673.73)	-1.17% (-1.23 to -1.11)
Afghanistan	96159 (70688 to 122486)	257526 (185543 to 332541)	167.81% (134.66 to 206.13)	1680.09 (1252.69 to 2140.27)	1240.48 (900.04 to 1586.22)	-1.09% (-1.23 to -0.95)
Albania	15158 (11634 to 18547)	6645 (5050 to 8072)	-56.17% (-61.68 to -50.35)	792.97 (611.18 to 969.26)	519.15 (395.49 to 630.14)	-1.72% (-1.97 to -1.45)
Algeria	114483 (85698 to 146583)	128879 (94553 to 170734)	12.57% (-6.19 to 34.15)	879.16 (664.37 to 1123.48)	560.74 (414.08 to 737.29)	-1.02% (-1.71 to -0.32)
American Samoa	243 (179 to 321)	153 (112 to 199)	-37.14% (-44.17 to -27.92)	876.46 (655.06 to 1148.12)	547.76 (405.63 to 717.46)	-1.65% (-1.71 to -1.6)
Angola	80633 (61351 to 104904)	169586 (124823 to 217002)	110.32% (83.01 to 141.65)	1572.32 (1216.94 to 2004.13)	1088.55 (807.68 to 1396.47)	-1.18% (-1.26 to -1.1)
Armenia	17644 (13206 to 21932)	7392 (5583 to 9381)	-58.11% (-63.79 to -51.93)	951.57 (706.41 to 1179.18)	550.74 (411.85 to 690.29)	-1.6% (-2.06 to -1.13)
Azerbaijan	42361 (30649 to 53007)	31425 (22954 to 39352)	-25.82% (-35.74 to -14.69)	931.96 (678.49 to 1161.3)	622.4 (453.64 to 772.59)	-0.94% (-1.16 to -0.71)

Location	Incident cases in 1990 (95% UI)	Incident cases in 2019 (95% UI)	Relative change (%, 95 CI)	ASIR in 1990 (95% UI)	ASIR in 2019 (95% UI)	EAPC (%, 95 CI)
Bangladesh	592233 (435008 to 791267)	448305 (331656 to 592764)	-24.3% (-35.22 to -13.84)	970.69 (722.93 to 1267.5)	468.8 (348.61 to 617.42)	-2.61% (-2.66 to -2.56)
Belarus	38857 (29552 to 47881)	26155 (19392 to 33357)	-32.69% (-42.24 to -19.93)	786.75 (596.21 to 962.73)	666.62 (501.31 to 837.63)	0.48% (0.01 to 0.95)
Belize	1171 (861 to 1536)	1360 (1003 to 1744)	16.22% (2.72 to 33.74)	1153.83 (855.1 to 1504.64)	551.96 (408.55 to 705.06)	-2.49% (-2.55 to -2.42)
Benin	26373 (20074 to 34594)	55897 (42170 to 71909)	111.95% (83.95 to 146.8)	1106.15 (846.3 to 1431.85)	849.9 (649.71 to 1093.19)	-0.69% (-0.76 to -0.62)
Bhutan	3787 (2815 to 4972)	2218 (1597 to 2889)	-41.43% (-49.16 to -33.37)	1142.88 (843.14 to 1466.29)	500.99 (362.32 to 650.1)	-3.05% (-3.16 to -2.95)
Bolivia (Plurinational State of)	42830 (32320 to 55485)	54529 (41252 to 69851)	27.31% (11.73 to 45.23)	1282.95 (975.53 to 1645.61)	851.53 (647.34 to 1085.3)	-1.45% (-1.58 to -1.31)
Bosnia and Herzegovina	13746 (10670 to 16192)	4090 (3229 to 5060)	-70.25% (-73.95 to -65.49)	566.17 (439.92 to 665.74)	307.62 (243.25 to 380.35)	-2.25% (-2.67 to -1.83)
Botswana	6637 (5037 to 8510)	8147 (6227 to 10366)	22.74% (4.85 to 44.04)	903.88 (694.47 to 1135.14)	592.69 (454.66 to 754.93)	-1.25% (-1.37 to -1.13)
Brazil	502182 (386807 to 638708)	649700 (520633 to 786245)	29.38% (8.15 to 47.21)	583.13 (451.95 to 734.85)	584.32 (466.5 to 704.77)	0.72% (0.45 to 0.99)

Location	Incident cases in 1990 (95% UI)	Incident cases in 2019 (95% UI)	Relative change (%, 95 CI)	ASIR in 1990 (95% UI)	ASIR in 2019 (95% UI)	EAPC (%, 95 CI)
Bulgaria	23311 (17231 to 27484)	9066 (7020 to 11144)	-61.11% (-66.51 to -53.97)	637.46 (470.95 to 751.48)	411.92 (315.98 to 498.01)	-0.84% (-1.18 to -0.51)
Burkina Faso	53492 (40481 to 69401)	102828 (78486 to 132583)	92.23% (64.28 to 130.85)	1187.98 (896.87 to 1521.28)	893.42 (687.57 to 1138.2)	-1.05% (-1.1 to -1)
Burundi	37373 (27988 to 48260)	66462 (49783 to 84724)	77.84% (56.05 to 100.97)	1381.16 (1047.59 to 1753.44)	1130.43 (865.68 to 1435.09)	-0.51% (-0.57 to -0.45)
Cabo Verde	1316 (973 to 1729)	1130 (847 to 1447)	-14.08% (-27.84 to 0.12)	748.99 (568.18 to 954.58)	361.93 (273.07 to 462.34)	-2.58% (-2.67 to -2.49)
Cambodia	69962 (51783 to 91883)	56109 (40355 to 74832)	-19.8% (-30.11 to -8.05)	1275.22 (948.88 to 1665.71)	596.21 (432.32 to 792.42)	-2.8% (-2.95 to -2.66)
Cameroon	53293 (40347 to 68314)	104476 (78934 to 135023)	96.04% (66.4 to 127.58)	1017.77 (776.99 to 1278.8)	652.57 (497.72 to 845.58)	-1.22% (-1.42 to -1.01)
Central African Republic	20097 (14835 to 25765)	31795 (24018 to 41111)	58.2% (41.16 to 81.97)	1382.05 (1037 to 1738.2)	1067.56 (818.65 to 1368.09)	-0.86% (-0.88 to -0.83)
Chad	36814 (28140 to 47377)	91446 (68586 to 118897)	148.4% (115.77 to 189.38)	1256.83 (954.61 to 1603.2)	1153.12 (863.12 to 1481.97)	-0.32% (-0.37 to -0.28)
China	3811058 (2674283 to 5163364)	1600299 (1169381 to 2118217)	-58.01% (-63.08 to -50.77)	509.67 (362.01 to 682.17)	246.03 (178.04 to 324.51)	-1.49% (-1.92 to -1.06)

Location	Incident cases in 1990 (95% UI)	Incident cases in 2019 (95% UI)	Relative change (%, 95 CI)	ASIR in 1990 (95% UI)	ASIR in 2019 (95% UI)	EAPC (%, 95 CI)
Colombia	162997 (120770 to 214679)	137623 (103193 to 176745)	-15.57% (-26.21 to -3.86)	825.6 (619.26 to 1075.24)	547.78 (410.68 to 702.61)	-1.58% (-1.65 to -1.5)
Comoros	3575 (2689 to 4586)	2543 (1910 to 3279)	-28.87% (-38.07 to -16.58)	1564.78 (1194.96 to 1985.66)	660.22 (500.75 to 850.14)	-3.02% (-3.11 to -2.93)
Congo	15952 (12078 to 20665)	20525 (15480 to 26464)	28.67% (12.47 to 49.08)	1242.49 (958.87 to 1601.55)	722.11 (542.62 to 925.42)	-1.51% (-1.67 to -1.36)
Costa Rica	12607 (9143 to 16687)	10368 (7586 to 13262)	-17.76% (-29.36 to -5)	721.66 (527.97 to 946.39)	408.6 (297.72 to 521.25)	-1.96% (-2.13 to -1.79)
Côte d'Ivoire	65921 (49263 to 84421)	100963 (74847 to 130568)	53.16% (30.9 to 75.71)	1093.19 (837.18 to 1380.34)	737.87 (550.71 to 941.11)	-1.33% (-1.37 to -1.29)
Cuba	33295 (24420 to 43901)	18381 (13653 to 23674)	-44.79% (-53.26 to -36.2)	491.27 (364.03 to 643.02)	407.11 (303.2 to 528.69)	-0.06% (-0.31 to 0.19)
Democratic People's Republic of Korea	104335 (74651 to 140514)	43495 (29764 to 59840)	-58.31% (-64.05 to -51.57)	862.7 (621.76 to 1162.06)	330.26 (226.88 to 452.38)	-3.39% (-3.43 to -3.35)
Democratic Republic of the Congo	295458 (222944 to 376429)	431127 (323999 to 566664)	45.92% (24.17 to 69.17)	1569.18 (1198.87 to 1976.28)	958.44 (726.98 to 1240.27)	-1.5% (-1.68 to -1.31)
Djibouti	3197 (2376 to 4115)	5067 (3926 to 6389)	58.48% (35.66 to 85.97)	1363.25 (1026.12 to 1727.73)	806.52 (627.09 to 1019.47)	-1.85% (-2.01 to -1.7)

Location	Incident cases in 1990 (95% UI)	Incident cases in 2019 (95% UI)	Relative change (%, 95 CI)	ASIR in 1990 (95% UI)	ASIR in 2019 (95% UI)	EAPC (%, 95 CI)
Dominica	308 (226 to 401)	134 (99 to 175)	-56.5% (-62.82 to -49.25)	781.25 (581.18 to 1004.68)	405.76 (300.97 to 530.1)	-2.48% (-2.57 to -2.39)
Dominican Republic	43939 (31370 to 58454)	43021 (31421 to 55993)	-2.09% (-14.53 to 12.51)	969.87 (702.82 to 1273.64)	718.99 (525.66 to 931.75)	-1.24% (-1.35 to -1.13)
Ecuador	55480 (40987 to 71300)	66809 (50158 to 84744)	20.42% (5.78 to 36.73)	975.52 (727.18 to 1247.66)	692.4 (519.8 to 875.97)	-1.16% (-1.23 to -1.09)
Egypt	337842 (246111 to 431423)	347533 (253094 to 444638)	2.87% (-10.13 to 17.61)	1153.4 (843.97 to 1470.05)	649.64 (473.99 to 830.67)	-1.58% (-1.71 to -1.45)
El Salvador	27438 (20126 to 35939)	17118 (12456 to 22483)	-37.61% (-45.38 to -28.36)	898.69 (672.33 to 1164.15)	466.53 (343.86 to 608.03)	-2.55% (-2.72 to -2.37)
Equatorial Guinea	3672 (2789 to 4720)	5773 (4304 to 7548)	57.23% (37.29 to 81.44)	1686.84 (1285.29 to 2129.04)	752.57 (568.3 to 973.74)	-2.82% (-2.98 to -2.66)
Eritrea	19822 (14833 to 25812)	28392 (21472 to 37002)	43.23% (24.24 to 65.86)	1337.16 (1013.34 to 1712.82)	787.72 (600.22 to 1010.17)	-1.76% (-1.87 to -1.64)
Eswatini	5218 (3921 to 6734)	4662 (3460 to 6109)	-10.65% (-21.69 to 1.96)	1154.91 (883.32 to 1467.07)	671.79 (502.5 to 872.47)	-1.64% (-1.7 to -1.58)
Ethiopia	401188 (303584 to 512903)	554579 (415660 to 719553)	38.23% (29.24 to 49.8)	1582.79 (1211.11 to 1987.72)	959.27 (730.38 to 1221.4)	-1.77% (-1.85 to -1.7)

Location	Incident cases in 1990 (95% UI)	Incident cases in 2019 (95% UI)	Relative change (%, 95 CI)	ASIR in 1990 (95% UI)	ASIR in 2019 (95% UI)	EAPC (%, 95 CI)
Fiji	2838 (2043 to 3802)	2352 (1699 to 3093)	-17.12% (-28.68 to -3.62)	655.82 (476.24 to 870.47)	510.93 (369.16 to 672.27)	-0.83% (-0.9 to -0.77)
Gabon	6592 (4901 to 8672)	6174 (4642 to 8021)	-6.35% (-18.61 to 7.29)	1304.74 (986.36 to 1673.07)	593.97 (450.32 to 768.78)	-2.64% (-2.68 to -2.61)
Gambia	5619 (4215 to 7258)	8174 (6219 to 10442)	45.49% (25.52 to 68.44)	1136.42 (865.63 to 1448.24)	670.92 (513.76 to 850.61)	-1.8% (-1.9 to -1.69)
Georgia	17687 (13047 to 22023)	8996 (6721 to 11251)	-49.14% (-55.34 to -41.2)	642.48 (470.24 to 795.72)	635.36 (482.49 to 794.8)	0.91% (0.4 to 1.41)
Ghana	67188 (51135 to 86845)	94428 (70748 to 120078)	40.54% (21.19 to 63.64)	899.77 (690.99 to 1144.37)	518.59 (393.41 to 657.36)	-1.79% (-1.9 to -1.67)
Grenada	386 (284 to 498)	233 (174 to 299)	-39.62% (-47.1 to -28.92)	863.17 (641.62 to 1105.43)	442.18 (331.6 to 564.99)	-2.07% (-2.36 to -1.78)
Guatemala	60395 (44938 to 78878)	63479 (46619 to 82750)	5.11% (-9.33 to 22.29)	1509.54 (1134.71 to 1945.47)	583.9 (429.69 to 755.26)	-3.55% (-3.78 to -3.32)
Guinea	35273 (26411 to 45120)	55201 (41924 to 70821)	56.49% (32.59 to 86.53)	1163.36 (889.37 to 1471.15)	809.26 (619.17 to 1023.75)	-1.28% (-1.36 to -1.2)
Guinea-Bissau	5302 (4079 to 6770)	7101 (5421 to 9038)	33.92% (15.33 to 54.57)	1046.62 (802.76 to 1316.5)	677.35 (522.8 to 845.73)	-1.54% (-1.61 to -1.47)

Location	Incident cases in 1990 (95% UI)	Incident cases in 2019 (95% UI)	Relative change (%, 95 CI)	ASIR in 1990 (95% UI)	ASIR in 2019 (95% UI)	EAPC (%, 95 CI)
Guyana	4256 (3107 to 5568)	2532 (1868 to 3318)	-40.5% (-47.9 to -32.06)	871.1 (642.04 to 1134.88)	556.84 (415.94 to 721.35)	-1.49% (-1.53 to -1.45)
Haiti	39966 (29783 to 50738)	52308 (39058 to 67613)	30.88% (14.34 to 48.08)	1211.84 (909.02 to 1528.95)	724.07 (541.26 to 930.58)	-1.8% (-1.83 to -1.77)
Honduras	29354 (21693 to 38158)	39252 (28626 to 51017)	33.72% (14.73 to 54.29)	1222.04 (912.79 to 1562.3)	650.11 (485.4 to 840.38)	-2.51% (-2.69 to -2.33)
India	4631691 (3426648 to 5907578)	3762909 (2747461 to 4894066)	-18.76% (-22.89 to -13.98)	1039.77 (778.79 to 1321.85)	487.69 (355.59 to 633.34)	-2.62% (-2.72 to -2.52)
Indonesia	787376 (585840 to 1019506)	616291 (461524 to 806604)	-21.73% (-29.03 to -13.61)	731.55 (545.12 to 945.27)	448.98 (336.01 to 589.37)	-1.61% (-1.73 to -1.5)
Iran (Islamic Republic of)	319668 (241651 to 392962)	182721 (137252 to 232582)	-42.84% (-48.68 to -33.6)	1044.67 (800.6 to 1285.74)	413.9 (310.93 to 523.13)	-2.38% (-2.71 to -2.05)
Iraq	119477 (89880 to 152922)	150711 (112825 to 191554)	26.14% (11.8 to 43.14)	1375.45 (1031.19 to 1761.95)	605.7 (455.3 to 768.4)	-2.92% (-3.06 to -2.78)
Jamaica	10446 (7605 to 13677)	5812 (4378 to 7487)	-44.36% (-52.9 to -34.35)	737.29 (541.06 to 957.97)	364.3 (275.6 to 466.8)	-2.58% (-2.64 to -2.52)
Jordan	18556 (13566 to 24502)	35661 (27884 to 43478)	92.18% (61.69 to 133.97)	960.28 (707.45 to 1260.68)	580.7 (459.6 to 705.4)	-1.49% (-1.64 to -1.34)

Location	Incident cases in 1990 (95% UI)	Incident cases in 2019 (95% UI)	Relative change (%, 95 CI)	ASIR in 1990 (95% UI)	ASIR in 2019 (95% UI)	EAPC (%, 95 CI)
Kazakhstan	73853 (54814 to 91758)	63170 (46429 to 79553)	-14.47% (-25.62 to -2.09)	843.56 (627.08 to 1045.58)	700.8 (509.7 to 877.4)	0.04% (-0.34 to 0.42)
Kenya	175780 (131888 to 225613)	215388 (160312 to 278256)	22.53% (19.11 to 25.72)	1461.41 (1116.65 to 1840.36)	729.8 (545.3 to 934.2)	-2.27% (-2.34 to -2.21)
Kiribati	343 (249 to 456)	388 (287 to 515)	13.01% (-1.6 to 31.28)	815.48 (598.01 to 1073.35)	585.8 (435.4 to 776.1)	-1.14% (-1.21 to -1.07)
Kyrgyzstan	24657 (18255 to 30827)	27221 (20435 to 34390)	10.4% (-2.45 to 24.75)	1019.53 (758.21 to 1274.09)	756.1 (570.8 to 952.7)	-0.25% (-0.69 to 0.2)
Lao People's Democratic Republic	25209 (18514 to 33263)	28708 (20465 to 37783)	13.88% (-1.61 to 28.65)	1175.09 (869.9 to 1533.28)	684.6 (491.9 to 896.1)	-2.43% (-2.63 to -2.22)
Lebanon	20229 (15055 to 25926)	13556 (10002 to 17660)	-32.99% (-43.13 to -21.38)	1219.99 (906.27 to 1565.61)	545.2 (405.7 to 693.6)	-2.81% (-2.86 to -2.76)
Lesotho	10457 (7924 to 13413)	7660 (5729 to 9920)	-26.75% (-35.78 to -16.52)	1090.8 (834.81 to 1378.82)	617.4 (465.8 to 793.3)	-1.71% (-1.83 to -1.59)
Liberia	10188 (7740 to 13086)	15754 (11973 to 20134)	54.63% (33.05 to 79.64)	1097.04 (824.46 to 1401.22)	595.2 (452.9 to 755)	-2.08% (-2.24 to -1.92)
Libya	19524 (14566 to 24860)	10556 (7702 to 13803)	-45.93% (-56.48 to -31.64)	976.37 (732.33 to 1234.95)	267.9 (197.1 to 349.9)	-3.88% (-4.2 to -3.57)

Location	Incident cases in 1990 (95% UI)	Incident cases in 2019 (95% UI)	Relative change (%, 95 CI)	ASIR in 1990 (95% UI)	ASIR in 2019 (95% UI)	EAPC (%, 95 CI)
Madagascar	78044 (57980 to 101451)	135290 (99182 to 178565)	73.35% (52 to 98.91)	1278.37 (959.63 to 1646.61)	905.7 (666.2 to 1176.8)	-1.27% (-1.38 to -1.16)
Malawi	73943 (55388 to 96047)	88847 (66091 to 116081)	20.16% (5.85 to 39.32)	1479.47 (1118.22 to 1886.37)	836 (627.9 to 1091.4)	-1.99% (-2.21 to -1.78)
Malaysia	69533 (50991 to 92016)	66721 (48923 to 89388)	-4.04% (-17.09 to 12.03)	717.14 (529.45 to 942.11)	379.9 (278.1 to 506.2)	-2.69% (-2.88 to -2.5)
Maldives	1139 (825 to 1509)	1035 (742 to 1365)	-9.19% (-23.04 to 7.07)	1051.13 (772.25 to 1370.94)	432.9 (312.8 to 566.7)	-2.68% (-3.01 to -2.34)
Mali	52835 (40751 to 68399)	108828 (82454 to 141345)	105.97% (77.68 to 137.72)	1285.49 (996 to 1638.77)	995.3 (759.4 to 1276.5)	-0.87% (-0.9 to -0.84)
Marshall Islands	227 (166 to 302)	195 (143 to 254)	-14.1% (-26.45 to -0.97)	974.52 (712.72 to 1289.23)	642.6 (470 to 836.7)	-1.33% (-1.39 to -1.27)
Mauritania	11260 (8816 to 14355)	13513 (10267 to 17306)	20.01% (3.13 to 39.54)	1130.52 (890.91 to 1424.05)	645.6 (491.1 to 825.7)	-1.87% (-1.99 to -1.75)
Mexico	428971 (318594 to 556759)	437296 (332874 to 562585)	1.94% (-8.08 to 12.85)	861.95 (646.55 to 1105.69)	648.7 (494.1 to 834.6)	-0.49% (-0.95 to -0.03)
Micronesia (Federated States of)	486 (361 to 627)	287 (209 to 370)	-40.86% (-47.93 to -32.67)	979.91 (739.15 to 1262.32)	533.9 (389.7 to 686.8)	-2.13% (-2.29 to -1.97)

Location	Incident cases in 1990 (95% UI)	Incident cases in 2019 (95% UI)	Relative change (%, 95 CI)	ASIR in 1990 (95% UI)	ASIR in 2019 (95% UI)	EAPC (%, 95 CI)
Mongolia	15386 (11432 to 19113)	15317 (11518 to 19281)	-0.45% (-13.11 to 14.61)	1252.51 (936.21 to 1551.72)	892.9 (671.3 to 1114.1)	-0.51% (-1.03 to 0.01)
Montenegro	1674 (1242 to 2013)	874 (656 to 1107)	-47.75% (-54.85 to -38.14)	531.24 (393.63 to 638.44)	332.1 (249 to 417.4)	-1.87% (-1.95 to -1.78)
Morocco	124777 (94094 to 157218)	87253 (65942 to 111706)	-30.07% (-39.92 to -19.06)	888.77 (676.4 to 1120.68)	454.6 (343.3 to 582.5)	-2.15% (-2.31 to -2)
Mozambique	93126 (69057 to 119177)	169834 (126949 to 220545)	82.37% (59.26 to 110.48)	1379.64 (1035.91 to 1744.54)	1041.7 (793.6 to 1328.1)	-0.88% (-1.01 to -0.75)
Myanmar	206907 (150460 to 271261)	161277 (118723 to 211624)	-22.05% (-31.72 to -11.28)	914.78 (677.67 to 1191.03)	537.9 (396.1 to 705.9)	-1.81% (-1.83 to -1.79)
Namibia	7631 (5752 to 9847)	9322 (6954 to 12214)	22.15% (6.18 to 40.48)	1027.14 (785.7 to 1306.33)	674.3 (508.3 to 880.3)	-1.15% (-1.28 to -1.03)
Nepal	127011 (93872 to 169518)	109078 (77836 to 146102)	-14.12% (-26.75 to -2.16)	1215.14 (910.36 to 1599.07)	550.8 (397.7 to 734.2)	-3.07% (-3.2 to -2.94)
Nicaragua	24567 (17313 to 32767)	21037 (15463 to 27724)	-14.37% (-26.89 to -2.4)	1136.28 (817.75 to 1488.26)	562 (414.6 to 737.7)	-2.58% (-2.8 to -2.36)
Niger	52936 (40730 to 67879)	138836 (104830 to 179870)	162.27% (128.49 to 208.72)	1379.8 (1077.5 to 1750.58)	1296.4 (996.6 to 1627.8)	-0.17% (-0.2 to -0.15)

Location	Incident cases in 1990 (95% UI)	Incident cases in 2019 (95% UI)	Relative change (%, 95 CI)	ASIR in 1990 (95% UI)	ASIR in 2019 (95% UI)	EAPC (%, 95 CI)
Nigeria	555433 (435752 to 694984)	999426 (782527 to 1241539)	79.94% (72.36 to 87.5)	1282.41 (1017.16 to 1579.69)	895.6 (707.1 to 1108.4)	-1.46% (-1.72 to -1.19)
North Macedonia	6947 (5197 to 8258)	2853 (2161 to 3544)	-58.94% (-64.9 to -51.44)	670.26 (500.57 to 796.05)	300.7 (226.3 to 370.8)	-3.02% (-3.26 to -2.78)
Pakistan	865250 (648553 to 1096370)	1172759 (874062 to 1498524)	35.54% (25.66 to 46.38)	1633.53 (1238.76 to 2050.01)	949.1 (712.9 to 1204.2)	-1.97% (-2.08 to -1.86)
Papua New Guinea	21825 (16014 to 28051)	47466 (35130 to 62178)	117.49% (92.09 to 149.19)	1024.79 (770.64 to 1303.97)	892.5 (661.6 to 1163.4)	-0.48% (-0.5 to -0.46)
Paraguay	21268 (15882 to 27183)	20003 (14831 to 25773)	-5.95% (-18.24 to 8.41)	1013.81 (760.2 to 1275.41)	516.4 (384.4 to 661.6)	-2.35% (-2.4 to -2.3)
Peru	122629 (90878 to 157182)	101076 (74056 to 130598)	-17.58% (-27.78 to -6.02)	1006.59 (753.73 to 1276.78)	570.2 (416.9 to 737.4)	-2.04% (-2.08 to -1.99)
Philippines	347831 (258098 to 451778)	458545 (339518 to 592456)	31.83% (27.97 to 35.8)	1017.22 (762.3 to 1314.62)	761.7 (565.7 to 979.6)	-1.06% (-1.14 to -0.97)
Republic of Moldova	22040 (17379 to 27097)	8583 (6583 to 10782)	-61.05% (-65.48 to -55.6)	1011.06 (787.73 to 1235.26)	528.3 (411.7 to 655.3)	-1.89% (-2.16 to -1.62)
Russian Federation	524961 (402882 to 646904)	427068 (319432 to 537251)	-18.65% (-27.43 to -6.76)	751.6 (572.17 to 922.55)	683.2 (520.1 to 855.9)	0.71% (0.17 to 1.25)

Location	Incident cases in 1990 (95% UI)	Incident cases in 2019 (95% UI)	Relative change (%, 95 CI)	ASIR in 1990 (95% UI)	ASIR in 2019 (95% UI)	EAPC (%, 95 CI)
Rwanda	44989 (33551 to 57001)	48504 (36165 to 62151)	7.81% (-6.68 to 22.85)	1315.68 (998.95 to 1644.39)	699 (524.7 to 889.4)	-2.39% (-2.51 to -2.27)
Saint Lucia	633 (462 to 827)	285 (211 to 369)	-55.07% (-61.84 to -48.09)	779.49 (577.96 to 1014.07)	322.9 (240.3 to 420.4)	-3% (-3.2 to -2.8)
Saint Vincent and the Grenadines	481 (358 to 631)	261 (192 to 333)	-45.81% (-52.99 to -37.35)	755.01 (564.29 to 981.06)	479.5 (353.7 to 614.5)	-1.53% (-1.63 to -1.43)
Samoa	416 (300 to 555)	262 (195 to 337)	-36.88% (-45.33 to -27.15)	516.92 (374.37 to 685.26)	255.6 (191.3 to 331.1)	-2.47% (-2.62 to -2.33)
Senegal	38054 (28928 to 48710)	50260 (37611 to 64776)	32.08% (12.93 to 52.12)	1040.94 (797.12 to 1325.87)	648.8 (485.4 to 835.7)	-1.4% (-1.52 to -1.28)
Serbia	19163 (13199 to 25460)	7912 (5753 to 10455)	-58.71% (-64.88 to -51.68)	461.87 (314.5 to 617.18)	227.3 (164.6 to 302.8)	-2.31% (-2.54 to -2.08)
Sierra Leone	19200 (14554 to 24730)	33380 (25445 to 43250)	73.86% (47.37 to 103.81)	1025.35 (789.2 to 1304.52)	716.6 (549.5 to 913.9)	-1.23% (-1.32 to -1.15)
Solomon Islands	2131 (1555 to 2809)	3147 (2327 to 4102)	47.69% (29.16 to 69.19)	1266.78 (939.07 to 1641.01)	909.3 (673.4 to 1185.9)	-1.22% (-1.26 to -1.17)
Somalia	51508 (39525 to 65306)	130590 (97995 to 167955)	153.53% (123.88 to 190.09)	1626.55 (1242.45 to 2059.78)	1328.4 (1006 to 1679.6)	-0.61% (-0.65 to -0.57)

Location	Incident cases in 1990 (95% UI)	Incident cases in 2019 (95% UI)	Relative change (%, 95 CI)	ASIR in 1990 (95% UI)	ASIR in 2019 (95% UI)	EAPC (%, 95 CI)
South Africa	161548 (121407 to 207190)	168010 (127792 to 214289)	4% (-4.66 to 14.16)	734.8 (563.22 to 925.8)	543.2 (413.7 to 691.2)	-0.9% (-0.96 to -0.84)
South Sudan	41300 (30814 to 53823)	59082 (44730 to 76305)	43.05% (24.54 to 65.96)	1404.93 (1054.28 to 1795.98)	1249.8 (962.7 to 1598.3)	-0.38% (-0.41 to -0.35)
Sri Lanka	54298 (39154 to 71372)	40025 (29094 to 54452)	-26.29% (-37.79 to -13.56)	552.48 (401.57 to 728.41)	371.9 (270.3 to 507)	-1.08% (-1.28 to -0.88)
Sudan	195168 (147149 to 245131)	199631 (151717 to 249270)	2.29% (-9.68 to 17.2)	1779.1 (1344.46 to 2217.15)	848.5 (647.7 to 1061.2)	-2.6% (-2.79 to -2.4)
Suriname	1670 (1204 to 2216)	1500 (1111 to 1970)	-10.2% (-22.47 to 3.76)	758.08 (554.2 to 992.47)	528.5 (391.6 to 692.8)	-1.15% (-1.27 to -1.02)
Syrian Arab Republic	82390 (61115 to 106325)	34901 (26248 to 44432)	-57.64% (-62.75 to -51.95)	1244.45 (938.34 to 1597.51)	448.9 (337.3 to 571.2)	-3% (-3.34 to -2.66)
Tajikistan	42489 (31822 to 52968)	55630 (41797 to 70143)	30.93% (14.84 to 48.03)	1443.43 (1094.19 to 1797.7)	1019.5 (768.2 to 1282.2)	-1.15% (-1.34 to -0.96)
Thailand	169176 (121301 to 225127)	95061 (71609 to 122494)	-43.81% (-51.47 to -35.41)	479.39 (344.87 to 635.46)	305.2 (228.8 to 394.6)	-1.47% (-1.55 to -1.39)
Timor-Leste	5607 (4112 to 7438)	5899 (4296 to 7768)	5.22% (-9.2 to 17.71)	1367.16 (1011.72 to 1792.85)	858 (632.5 to 1126.2)	-1.85% (-2.07 to -1.62)

Location	Incident cases in 1990 (95% UI)	Incident cases in 2019 (95% UI)	Relative change (%, 95 CI)	ASIR in 1990 (95% UI)	ASIR in 2019 (95% UI)	EAPC (%, 95 CI)
Togo	17956 (13651 to 22696)	26696 (19771 to 33992)	48.67% (26.37 to 70.91)	997.09 (765.36 to 1248.58)	629.3 (469.9 to 800.2)	-1.34% (-1.45 to -1.22)
Tonga	339 (250 to 443)	294 (218 to 380)	-13.12% (-25.68 to 0.45)	733.8 (546.68 to 963.11)	588.8 (433 to 758.7)	-0.67% (-0.72 to -0.62)
Tunisia	39535 (28574 to 50548)	20323 (14993 to 26644)	-48.59% (-57.37 to -35.49)	842.92 (614.96 to 1071.11)	334.2 (244.8 to 434.4)	-3.12% (-3.28 to -2.96)
Turkey	377830 (298819 to 459517)	231569 (179771 to 291870)	-38.71% (-46.15 to -28.82)	1123.43 (898.76 to 1374.98)	536.5 (419.2 to 675)	-2.34% (-2.41 to -2.28)
Turkmenistan	24574 (18540 to 30921)	20342 (15163 to 25910)	-17.22% (-28.16 to -5)	1174.8 (891.87 to 1465.77)	801.9 (592.5 to 1015.2)	-1.08% (-1.45 to -0.7)
Tuvalu	38 (28 to 49)	29 (22 to 39)	-22.47% (-30.71 to -11.84)	745.96 (550.71 to 977.46)	496.6 (367.9 to 653.2)	-1.43% (-1.46 to -1.41)
Uganda	155270 (114301 to 200387)	236965 (174863 to 314531)	52.61% (33.42 to 74.64)	1709.5 (1281.62 to 2156.23)	1052.3 (783.5 to 1386.5)	-1.79% (-1.93 to -1.65)
Ukraine	188316 (144023 to 233210)	107579 (79988 to 135293)	-42.87% (-51.02 to -31.52)	806.64 (613.77 to 996.44)	588.8 (438.7 to 737.5)	-0.23% (-0.75 to 0.29)
United Republic of Tanzania	192915 (144628 to 252888)	320361 (238243 to 423149)	66.06% (46.46 to 89.57)	1414.43 (1082.04 to 1819.59)	1023.4 (769.6 to 1328.7)	-0.98% (-1.05 to -0.91)

Location	Incident cases in 1990 (95% UI)	Incident cases in 2019 (95% UI)	Relative change (%, 95 CI)	ASIR in 1990 (95% UI)	ASIR in 2019 (95% UI)	EAPC (%, 95 CI)
Uzbekistan	150829 (113178 to 186618)	153955 (114066 to 191729)	2.07% (-10.01 to 16.51)	1274.18 (957.96 to 1572.48)	816.3 (605.4 to 1014.6)	-1.47% (-1.78 to -1.16)
Vanuatu	787 (583 to 1030)	1073 (781 to 1441)	36.27% (20.21 to 55.51)	1001.83 (748.61 to 1294.83)	662.2 (485.8 to 884.2)	-1.54% (-1.59 to -1.48)
Venezuela (Bolivarian Republic of)	87434 (64399 to 115384)	84263 (62393 to 109763)	-3.63% (-17.83 to 11.8)	802.17 (593.83 to 1052.1)	604.3 (445.4 to 792.5)	-0.53% (-0.78 to -0.29)
Viet Nam	279626 (199204 to 379182)	200655 (145169 to 264128)	-28.24% (-38.31 to -17.7)	698.01 (504.07 to 937.13)	393.7 (285.6 to 518.1)	-1.73% (-1.82 to -1.64)
Yemen	120321 (90813 to 150650)	160281 (118568 to 201630)	33.21% (14.68 to 50.57)	1924.21 (1454.26 to 2402.84)	939.5 (696.9 to 1183.1)	-2.5% (-2.69 to -2.32)
Zambia	62566 (46357 to 81258)	89494 (66407 to 119127)	43.04% (23.44 to 66.48)	1463.17 (1096.95 to 1858.4)	855.7 (641.2 to 1118.4)	-1.8% (-1.97 to -1.63)
Zimbabwe	64969 (48807 to 83784)	72678 (53412 to 95463)	11.87% (-2.37 to 26.59)	1172.79 (897.77 to 1485.43)	830.1 (613.2 to 1085.8)	-0.8% (-0.94 to -0.66)

ASIR – age-standardized incidence rate (per 100,000 population), CI – confidence interval, EAPC – estimated annual percentage change, MSMI – maternal sepsis and other maternal infections, UI – uncertain interval..

Table S5. MMR of MSMI in 131 low- and middle-income countries and territories with EAPC from 1990 to 2019.

Location	Deaths in 1990 (95% UI)	Deaths in 2019 (95% UI)	Relative change (%, 95 CI)	ASMMR in 1990 (95% UI)	ASMMR in 2019 (95% UI)	EAPC (%, 95 CI)
Global	38027 (33015 to 43506)	16840 (14225 to 19634)	-55.72% (-63.78 to -47.52)	27.53 (23.9 to 31.49)	12.44 (10.51 to 14.51)	-2.83% (-3.22 to -2.43)
Afghanistan	274 (198 to 379)	324 (220 to 469)	18% (-20.06 to 81.64)	51.99 (37.45 to 71.78)	21.65 (14.7 to 31.34)	-3.14% (-4.21 to -2.06)
Albania	0 (0 to 1)	0 (0 to 0)	-76.79% (-86.18 to -62.43)	0.53 (0.39 to 0.7)	0.29 (0.18 to 0.44)	0.74% (-1.03 to 2.53)
Algeria	321 (229 to 446)	29 (20 to 41)	-90.99% (-94.21 to -85.71)	41.69 (29.78 to 57.97)	3.27 (2.22 to 4.61)	-9.05% (-9.27 to -8.83)
American Samoa	0 (0 to 0)	0 (0 to 0)	-63.11% (-77.56 to -38.41)	6.21 (4.45 to 8.58)	3.79 (2.54 to 5.37)	-1.96% (-2.26 to -1.65)
Angola	162 (109 to 226)	146 (90 to 211)	-9.92% (-48.07 to 44.59)	31.64 (21.28 to 44.11)	13.25 (8.1 to 19.13)	-2.91% (-3.18 to -2.63)
Armenia	1 (1 to 2)	0 (0 to 0)	-81.95% (-88.05 to -72.72)	1.66 (1.23 to 2.17)	0.62 (0.45 to 0.86)	-4.27% (-5.47 to -3.05)

Location	Deaths in 1990 (95% UI)	Deaths in 2019 (95% UI)	Relative change (%, 95 CI)	ASMMR in 1990 (95% UI)	ASMMR in 2019 (95% UI)	EAPC (%, 95 CI)
Azerbaijan	6 (5 to 8)	1 (0 to 1)	-90.14% (-93.71 to -85.16)	2.96 (2.22 to 3.9)	0.39 (0.26 to 0.55)	-9.71% (-10.9 to -8.51)
Bangladesh	479 (340 to 644)	17 (12 to 24)	-96.37% (-97.71 to -94.33)	12.65 (8.98 to 17)	0.65 (0.46 to 0.9)	-9.56% (-10.92 to -8.19)
Belarus	1 (1 to 2)	0 (0 to 0)	-87.9% (-92.13 to -81.51)	1.01 (0.79 to 1.27)	0.17 (0.11 to 0.25)	-7.53% (-8.19 to -6.87)
Belize	0 (0 to 0)	0 (0 to 1)	61.67% (10.84 to 135.63)	5.11 (3.88 to 6.45)	6.41 (4.86 to 8.22)	1.34% (1 to 1.67)
Benin	132 (98 to 174)	114 (72 to 170)	-14.11% (-47.48 to 33.4)	55.79 (41.31 to 73.61)	22.47 (14.34 to 33.71)	-2.67% (-2.98 to -2.36)
Bhutan	2 (1 to 3)	0 (0 to 0)	-93.33% (-96.47 to -86.67)	10.28 (5.25 to 15.16)	1.14 (0.66 to 1.77)	-7.77% (-8.06 to -7.47)
Bolivia (Plurinational State of)	135 (102 to 179)	45 (27 to 64)	-66.61% (-80.57 to -47.58)	54.85 (41.71 to 72.93)	13.75 (8.21 to 19.61)	-5.21% (-5.45 to -4.98)

Location	Deaths in 1990 (95% UI)	Deaths in 2019 (95% UI)	Relative change (%, 95 CI)	ASMMR in 1990 (95% UI)	ASMMR in 2019 (95% UI)	EAPC (%, 95 CI)
Bosnia and Herzegovina	3 (2 to 4)	0 (0 to 0)	-99.8% (-99.88 to -99.69)	4.37 (3.3 to 5.72)	0.02 (0.01 to 0.03)	-21.25% (-22.88 to -19.58)
Botswana	13 (8 to 19)	4 (2 to 6)	-68.54% (-83.64 to -44.52)	31.79 (20.37 to 46.69)	8.39 (4.55 to 13.07)	-4.88% (-5.77 to -3.97)
Brazil	443 (369 to 528)	156 (130 to 188)	-64.76% (-72.9 to -54.06)	12.93 (10.76 to 15.4)	5.01 (4.16 to 6.03)	-2.35% (-2.87 to -1.82)
Bulgaria	4 (3 to 5)	0 (0 to 0)	-91.28% (-94.35 to -87.23)	3.66 (2.95 to 4.57)	0.53 (0.36 to 0.75)	-8.5% (-9.52 to -7.46)
Burkina Faso	210 (145 to 296)	181 (112 to 261)	-14% (-47.39 to 32.84)	46.01 (31.79 to 64.78)	19.29 (12 to 27.89)	-3.62% (-3.99 to -3.25)
Burundi	235 (163 to 326)	308 (215 to 411)	30.7% (-15.04 to 96.18)	88.76 (61.45 to 123.11)	65.97 (46.13 to 88.17)	-1.57% (-2.07 to -1.06)
Cabo Verde	1 (1 to 2)	0 (0 to 0)	-92.77% (-95.65 to -88.93)	12.48 (9.31 to 16.55)	0.97 (0.64 to 1.42)	-9.24% (-9.56 to -8.92)

Location	Deaths in 1990 (95% UI)	Deaths in 2019 (95% UI)	Relative change (%, 95 CI)	ASMMR in 1990 (95% UI)	ASMMR in 2019 (95% UI)	EAPC (%, 95 CI)
Cambodia	229 (162 to 324)	41 (28 to 59)	-81.91% (-88.68 to -70.62)	51.57 (36.62 to 72.94)	11.21 (7.64 to 15.85)	-6.46% (-7.63 to -5.28)
Cameroon	198 (144 to 255)	222 (130 to 321)	11.92% (-34.24 to 72.12)	42.65 (30.9 to 54.92)	24.54 (14.39 to 35.52)	-1.97% (-2.31 to -1.64)
Central African Republic	118 (85 to 157)	242 (162 to 354)	104.59% (33.29 to 218.48)	92.6 (66.57 to 123.07)	122.36 (81.78 to 179.27)	0.99% (0.84 to 1.14)
Chad	260 (195 to 338)	367 (251 to 501)	41.48% (-7.06 to 109.56)	81.09 (61.03 to 105.49)	46.63 (31.82 to 63.59)	-1.89% (-2.26 to -1.53)
China	1841 (1435 to 2319)	20 (15 to 25)	-98.94% (-99.26 to -98.5)	7.56 (5.9 to 9.52)	0.13 (0.1 to 0.17)	-13.7% (-14.44 to -12.96)
Colombia	77 (62 to 96)	26 (18 to 36)	-66.27% (-79.08 to -48.99)	8.17 (6.58 to 10.15)	3.22 (2.21 to 4.48)	-2.87% (-3.43 to -2.3)
Comoros	5 (1 to 8)	3 (2 to 5)	-32.65% (-66.23 to 222.95)	22.71 (4.77 to 37.67)	18.49 (9.4 to 27.9)	-1.35% (-1.97 to -0.73)

Location	Deaths in 1990 (95% UI)	Deaths in 2019 (95% UI)	Relative change (%, 95 CI)	ASMMR in 1990 (95% UI)	ASMMR in 2019 (95% UI)	EAPC (%, 95 CI)
Congo	35 (25 to 48)	30 (19 to 45)	-15.21% (-49.51 to 38.52)	34.73 (24.81 to 47.33)	20.65 (13.23 to 30.95)	-1.82% (-2.51 to -1.12)
Costa Rica	4 (3 to 5)	1 (0 to 1)	-81.77% (-88.16 to -72.73)	4.64 (3.72 to 5.64)	1.03 (0.7 to 1.46)	-5.83% (-7.06 to -4.59)
Côte d'Ivoire	141 (97 to 191)	149 (91 to 218)	5.76% (-37.43 to 65.34)	24.45 (16.96 to 33.16)	16.66 (10.25 to 24.41)	-1.01% (-1.42 to -0.6)
Cuba	12 (10 to 15)	2 (2 to 3)	-79.1% (-85.9 to -70.02)	6.62 (5.29 to 8.14)	2.39 (1.7 to 3.25)	-3.92% (-4.33 to -3.52)
Democratic People's Republic of Korea	48 (29 to 73)	38 (23 to 56)	-22.1% (-57.74 to 46.59)	6.26 (3.79 to 9.37)	10.76 (6.54 to 16)	2.61% (1.31 to 3.92)
Democratic Republic of the Congo	1076 (733 to 1477)	2770 (2026 to 3675)	157.43% (74.22 to 286.29)	56.91 (38.75 to 78.11)	94.36 (69.02 to 125.17)	3.19% (2.14 to 4.24)
Djibouti	11 (7 to 17)	17 (9 to 27)	49.62% (-26.28 to 165.78)	51.51 (32.07 to 74.44)	48.24 (24.04 to 77.32)	-0.01% (-0.38 to 0.35)
Dominica	0 (0 to 0)	0 (0 to 0)	-39.24% (-63.87 to -3.21)	15.31 (11.21 to 20.5)	19.38 (13.46 to 27.39)	1.34% (1.04 to 1.64)

Location	Deaths in 1990 (95% UI)	Deaths in 2019 (95% UI)	Relative change (%, 95 CI)	ASMMR in 1990 (95% UI)	ASMMR in 2019 (95% UI)	EAPC (%, 95 CI)
Dominican Republic	15 (11 to 19)	10 (6 to 14)	-35.33% (-61.1 to 4.1)	6.04 (4.63 to 7.8)	4.18 (2.78 to 6.18)	-0.42% (-1.15 to 0.31)
Ecuador	48 (39 to 59)	15 (11 to 22)	-67.82% (-78.4 to -52.43)	15.85 (12.75 to 19.33)	4.42 (3.1 to 6.29)	-3.53% (-4.53 to -2.53)
Egypt	332 (264 to 411)	24 (15 to 35)	-92.83% (-95.58 to -88.97)	17.01 (13.54 to 21.03)	1.13 (0.73 to 1.67)	-10.18% (-10.7 to -9.65)
El Salvador	66 (53 to 81)	3 (2 to 5)	-95.37% (-97.19 to -92.71)	39.73 (31.88 to 48.7)	2.72 (1.67 to 4.06)	-8.46% (-9.68 to -7.22)
Equatorial Guinea	22 (15 to 30)	2 (1 to 3)	-90.66% (-95.31 to -82.38)	97.48 (66.44 to 132.11)	5.46 (2.78 to 9.08)	-10.79% (-11.61 to -9.96)
Eritrea	180 (115 to 259)	152 (100 to 229)	-15.56% (-47.87 to 39.09)	132.56 (84.42 to 190.25)	76.45 (50.2 to 114.98)	-0.93% (-1.6 to -0.25)
Eswatini	5 (3 to 7)	2 (1 to 4)	-54.64% (-77.6 to -15.33)	14.86 (10.23 to 20.6)	7.25 (3.54 to 12.45)	-1.64% (-2.76 to -0.5)
Ethiopia	3575 (2569 to 5011)	1176 (824 to 1646)	-67.11% (-79.84 to -49.4)	144.9 (104.11 to 203.09)	32 (22.42 to 44.79)	-5.46% (-6.2 to -4.71)

Location	Deaths in 1990 (95% UI)	Deaths in 2019 (95% UI)	Relative change (%, 95 CI)	ASMMR in 1990 (95% UI)	ASMMR in 2019 (95% UI)	EAPC (%, 95 CI)
Fiji	1 (1 to 2)	1 (0 to 1)	-51.26% (-71.08 to -16.64)	6.95 (4.75 to 9.85)	3.66 (2.4 to 5.4)	-2.73% (-3 to -2.47)
Gabon	10 (7 to 13)	3 (2 to 5)	-63.54% (-78.81 to -42.17)	23.79 (17.41 to 31.43)	8.15 (4.96 to 12.33)	-3.97% (-4.68 to -3.25)
Gambia	21 (13 to 29)	28 (19 to 39)	37.42% (-10.23 to 118.75)	40.99 (26.93 to 57.64)	39.17 (26.88 to 54.03)	-0.39% (-0.68 to -0.09)
Georgia	2 (1 to 2)	1 (1 to 2)	-31.68% (-56.45 to 7.57)	2.03 (1.54 to 2.7)	2.45 (1.78 to 3.39)	2.42% (0.34 to 4.53)
Ghana	152 (103 to 208)	66 (42 to 95)	-56.8% (-73.75 to -33.51)	25.04 (16.96 to 34.23)	7.68 (4.93 to 11.09)	-3.6% (-3.81 to -3.39)
Grenada	0 (0 to 0)	0 (0 to 0)	-80.44% (-86.98 to -71.35)	2.11 (1.6 to 2.72)	0.58 (0.42 to 0.77)	-3.47% (-4.28 to -2.65)
Guatemala	79 (60 to 101)	26 (18 to 37)	-66.73% (-78.52 to -50.16)	23.17 (17.6 to 29.52)	6.43 (4.49 to 8.97)	-5.57% (-6.59 to -4.53)

Location	Deaths in 1990 (95% UI)	Deaths in 2019 (95% UI)	Relative change (%, 95 CI)	ASMMR in 1990 (95% UI)	ASMMR in 2019 (95% UI)	EAPC (%, 95 CI)
Guinea	221 (164 to 286)	231 (164 to 317)	4.23% (-28.29 to 51.32)	71.7 (53.16 to 92.71)	48.31 (34.29 to 66.48)	-0.84% (-1 to -0.67)
Guinea-Bissau	21 (15 to 28)	12 (8 to 17)	-41.26% (-64 to -9.54)	44.3 (31.21 to 60.82)	19.33 (12.93 to 27.39)	-2.49% (-2.74 to -2.23)
Guyana	2 (2 to 3)	1 (1 to 1)	-67.08% (-80.03 to -46.43)	10.45 (7.6 to 13.82)	5.46 (3.54 to 7.92)	-2.14% (-2.54 to -1.73)
Haiti	207 (143 to 298)	334 (221 to 471)	61.48% (8.77 to 141.5)	83.3 (57.76 to 120.02)	100.21 (66.27 to 141.19)	1.4% (1.06 to 1.74)
Honduras	115 (88 to 146)	16 (9 to 25)	-86.19% (-92.36 to -77.79)	63.89 (48.67 to 80.74)	6.83 (3.85 to 10.7)	-7.97% (-8.27 to -7.66)
India	14407 (11250 to 18005)	1919 (1344 to 2641)	-86.68% (-91.06 to -80.62)	54.79 (42.78 to 68.47)	8.18 (5.73 to 11.26)	-6.91% (-7.65 to -6.15)
Indonesia	1173 (880 to 1548)	156 (113 to 209)	-86.67% (-91.2 to -80.08)	24.59 (18.44 to 32.45)	4.07 (2.94 to 5.45)	-5.98% (-6.28 to -5.67)
Iran (Islamic Republic of)	134 (110 to 167)	12 (9 to 15)	-91.32% (-93.63 to -87.97)	7.67 (6.29 to 9.57)	0.86 (0.68 to 1.1)	-8.03% (-8.37 to -7.69)

Location	Deaths in 1990 (95% UI)	Deaths in 2019 (95% UI)	Relative change (%, 95 CI)	ASMMR in 1990 (95% UI)	ASMMR in 2019 (95% UI)	EAPC (%, 95 CI)
Iraq	8 (5 to 11)	4 (2 to 6)	-50.19% (-71.39 to -10.9)	1.07 (0.73 to 1.51)	0.41 (0.26 to 0.63)	-2.79% (-3.7 to -1.86)
Jamaica	2 (2 to 3)	0 (0 to 1)	-80.54% (-87.66 to -68.66)	4.18 (3.2 to 5.24)	1.26 (0.83 to 1.88)	-6.21% (-7.56 to -4.84)
Jordan	13 (9 to 17)	4 (3 to 6)	-68.83% (-81.04 to -47.04)	9.29 (6.65 to 12.67)	1.66 (1.12 to 2.44)	-7.6% (-8.27 to -6.92)
Kazakhstan	1 (1 to 2)	3 (2 to 3)	83.18% (23.93 to 165.2)	0.4 (0.31 to 0.51)	0.74 (0.55 to 0.97)	1.69% (0.7 to 2.69)
Kenya	261 (193 to 342)	258 (181 to 365)	-1.02% (-34.65 to 46.55)	25.64 (19.02 to 33.65)	19.21 (13.45 to 27.21)	-0.67% (-1.18 to -0.16)
Kiribati	1 (0 to 1)	1 (0 to 1)	-0.69% (-39.91 to 63.98)	22.87 (15.71 to 31.75)	19.53 (13.13 to 27.92)	-0.77% (-1.04 to -0.5)
Kyrgyzstan	3 (3 to 4)	4 (3 to 5)	21.73% (-13.73 to 67.31)	2.68 (2.1 to 3.42)	2.8 (2.14 to 3.56)	1.18% (0.06 to 2.3)
Lao People's Democratic Republic	110 (76 to 158)	10 (6 to 14)	-91.15% (-94.8 to -86.21)	66.27 (46.07 to 95.42)	5.59 (3.55 to 7.91)	-8.19% (-8.84 to -7.54)

Location	Deaths in 1990 (95% UI)	Deaths in 2019 (95% UI)	Relative change (%, 95 CI)	ASMMR in 1990 (95% UI)	ASMMR in 2019 (95% UI)	EAPC (%, 95 CI)
Lebanon	3 (2 to 5)	0 (0 to 1)	-86.35% (-91.78 to -76.43)	2.91 (1.97 to 4.13)	0.42 (0.29 to 0.64)	-7% (-7.46 to -6.54)
Lesotho	34 (23 to 47)	20 (11 to 32)	-40.83% (-68.26 to 0.74)	55.08 (36.61 to 74.71)	43.53 (24.49 to 68.3)	1.16% (0.28 to 2.05)
Liberia	79 (57 to 104)	130 (86 to 181)	65.11% (4.43 to 143.63)	88.91 (64.36 to 117.64)	96.17 (63.14 to 133.92)	-0.93% (-1.74 to -0.1)
Libya	3 (2 to 4)	1 (1 to 2)	-63.92% (-79.79 to -37.37)	1.91 (1.29 to 2.83)	1.32 (0.83 to 2.03)	-1.11% (-2.07 to -0.15)
Madagascar	284 (216 to 358)	361 (250 to 488)	27.18% (-10.84 to 77.14)	52.51 (39.93 to 66.27)	41.86 (29.06 to 56.59)	-0.74% (-1.07 to -0.42)
Malawi	165 (116 to 218)	130 (82 to 189)	-21.2% (-53.4 to 27.37)	35.09 (24.75 to 46.5)	23.83 (15.05 to 34.71)	-1.09% (-1.39 to -0.79)
Malaysia	56 (44 to 72)	10 (7 to 15)	-81.85% (-88.82 to -70.45)	11.37 (8.86 to 14.49)	1.89 (1.26 to 2.76)	-6.13% (-6.41 to -5.86)
Maldives	1 (0 to 1)	0 (0 to 0)	-92.45% (-95.12 to -87.99)	7.66 (5.37 to 10.85)	0.6 (0.43 to 0.81)	-9.16% (-9.87 to -8.45)

Location	Deaths in 1990 (95% UI)	Deaths in 2019 (95% UI)	Relative change (%, 95 CI)	ASMMR in 1990 (95% UI)	ASMMR in 2019 (95% UI)	EAPC (%, 95 CI)
Mali	596 (471 to 746)	404 (262 to 584)	-32.21% (-56.41 to 2.68)	131.95 (104.26 to 165.04)	42.06 (27.31 to 60.71)	-4.24% (-4.56 to -3.93)
Marshall Islands	0 (0 to 0)	0 (0 to 0)	-20.98% (-55.51 to 27.85)	15.33 (10.97 to 20.61)	14.71 (8.43 to 22.36)	-0.58% (-1.2 to 0.05)
Mauritania	128 (101 to 162)	32 (20 to 48)	-74.66% (-84.24 to -61)	141.46 (111.64 to 178.9)	29.77 (18.23 to 43.99)	-5.96% (-6.25 to -5.67)
Mexico	107 (87 to 129)	34 (25 to 45)	-68.69% (-78.59 to -55.66)	4.28 (3.5 to 5.14)	1.6 (1.19 to 2.13)	-3% (-3.25 to -2.74)
Micronesia (Federated States of)	1 (1 to 1)	0 (0 to 0)	-72.49% (-91.43 to -51)	26.53 (17.89 to 39.51)	13.2 (4.36 to 22.05)	-2.76% (-2.9 to -2.61)
Mongolia	23 (16 to 31)	5 (3 to 7)	-79.39% (-87.78 to -65.1)	30.52 (21.48 to 40.97)	5.65 (3.62 to 8.83)	-7.7% (-8.57 to -6.81)
Montenegro	0 (0 to 0)	0 (0 to 0)	-66.12% (-79.92 to -43.88)	0.04 (0.03 to 0.06)	0.02 (0.01 to 0.03)	-3.11% (-4.16 to -2.05)
Morocco	291 (212 to 382)	20 (13 to 32)	-93.05% (-95.75 to -88.58)	36.08 (26.27 to 47.31)	3.33 (2.15 to 5.3)	-8.17% (-8.49 to -7.85)

Location	Deaths in 1990 (95% UI)	Deaths in 2019 (95% UI)	Relative change (%, 95 CI)	ASMMR in 1990 (95% UI)	ASMMR in 2019 (95% UI)	EAPC (%, 95 CI)
Mozambique	303 (216 to 411)	138 (85 to 202)	-54.44% (-73.63 to -30.04)	50.16 (35.86 to 68.03)	12.26 (7.6 to 17.96)	-4.37% (-4.94 to -3.81)
Myanmar	441 (296 to 663)	115 (74 to 167)	-73.89% (-85.53 to -54.86)	32.77 (21.96 to 49.27)	10.91 (6.97 to 15.81)	-4.35% (-4.94 to -3.75)
Namibia	14 (8 to 19)	5 (3 to 8)	-64.92% (-81.57 to -28.5)	28.42 (15.95 to 40.06)	7.65 (4.3 to 12.93)	-4.3% (-4.96 to -3.64)
Nepal	463 (333 to 630)	39 (25 to 55)	-91.63% (-94.99 to -87.17)	55.78 (40.08 to 75.79)	6.28 (4.11 to 8.84)	-7.21% (-7.46 to -6.96)
Nicaragua	17 (14 to 22)	2 (1 to 3)	-87.63% (-92.23 to -81.11)	12.07 (9.53 to 15.04)	1.65 (1.14 to 2.26)	-7.5% (-7.89 to -7.11)
Niger	366 (264 to 484)	573 (378 to 810)	56.43% (-0.54 to 134.87)	82.33 (59.45 to 108.8)	50.14 (33.08 to 70.9)	-1.92% (-2.14 to -1.69)
Nigeria	790 (471 to 1251)	833 (489 to 1380)	5.43% (-43.71 to 86.39)	18.48 (11.01 to 29.26)	10.98 (6.44 to 18.18)	-2.6% (-3.32 to -1.88)
North Macedonia	0 (0 to 1)	0 (0 to 0)	-94.3% (-96.58 to -90.21)	1.16 (0.82 to 1.57)	0.11 (0.07 to 0.16)	-9.86% (-10.56 to -9.16)

Location	Deaths in 1990 (95% UI)	Deaths in 2019 (95% UI)	Relative change (%, 95 CI)	ASMMR in 1990 (95% UI)	ASMMR in 2019 (95% UI)	EAPC (%, 95 CI)
Pakistan	1496 (1174 to 1872)	823 (575 to 1127)	-45% (-64.93 to -17.82)	30.6 (24.02 to 38.3)	12.3 (8.6 to 16.86)	-3.38% (-3.72 to -3.04)
Papua New Guinea	77 (53 to 104)	80 (55 to 112)	4.46% (-32.04 to 59.38)	51.1 (35.31 to 69.24)	24.12 (16.68 to 33.87)	-1.92% (-2.35 to -1.49)
Paraguay	29 (22 to 36)	5 (3 to 7)	-82.19% (-88.73 to -72.05)	22.72 (17.59 to 28.41)	4.09 (2.69 to 5.97)	-6.54% (-7.2 to -5.88)
Peru	198 (152 to 253)	22 (14 to 34)	-88.81% (-92.89 to -82.09)	28.95 (22.23 to 37.11)	3.38 (2.09 to 5.14)	-8.27% (-8.77 to -7.78)
Philippines	143 (112 to 183)	57 (38 to 80)	-60.17% (-74.49 to -40.04)	6.83 (5.38 to 8.79)	2.13 (1.41 to 3)	-3.67% (-3.86 to -3.48)
Republic of Moldova	3 (2 to 3)	0 (0 to 0)	-97.25% (-98.1 to -96.07)	3.37 (2.67 to 4.17)	0.22 (0.16 to 0.3)	-10.27% (-11.12 to -9.41)
Russian Federation	49 (40 to 60)	7 (5 to 9)	-86.42% (-91.1 to -80.32)	2.57 (2.08 to 3.16)	0.4 (0.28 to 0.55)	-8.5% (-9.41 to -7.58)

Location	Deaths in 1990 (95% UI)	Deaths in 2019 (95% UI)	Relative change (%, 95 CI)	ASMMR in 1990 (95% UI)	ASMMR in 2019 (95% UI)	EAPC (%, 95 CI)
Rwanda	478 (360 to 633)	167 (109 to 243)	-65% (-81.05 to -43.81)	149.93 (113.01 to 198.68)	47.47 (30.89 to 68.89)	-4.42% (-5.26 to -3.57)
Saint Lucia	0 (0 to 0)	0 (0 to 0)	-68.48% (-78.43 to -53.96)	2.64 (2.08 to 3.38)	1.79 (1.28 to 2.44)	0.42% (-0.83 to 1.67)
Saint Vincent and the Grenadines	0 (0 to 0)	0 (0 to 0)	-40.82% (-60.22 to -13.67)	0.58 (0.45 to 0.74)	0.6 (0.45 to 0.81)	0.38% (-0.32 to 1.09)
Samoa	0 (0 to 1)	0 (0 to 0)	-77.09% (-90.3 to -56.39)	12.26 (7.73 to 18.35)	2.49 (1.21 to 4.02)	-6.85% (-7.57 to -6.12)
Senegal	166 (118 to 219)	131 (83 to 184)	-21.45% (-53.14 to 19.17)	48.29 (34.4 to 63.58)	28 (17.86 to 39.38)	-1.75% (-2.09 to -1.4)
Serbia	1 (1 to 2)	0 (0 to 0)	-89.46% (-93.86 to -82.63)	0.8 (0.58 to 1.1)	0.15 (0.1 to 0.21)	-7.31% (-8.13 to -6.49)
Sierra Leone	76 (52 to 106)	140 (93 to 194)	82.75% (13.35 to 189.14)	46.32 (31.54 to 64.24)	49.72 (33.08 to 68.95)	0.38% (-0.24 to 1.01)
Solomon Islands	7 (4 to 10)	10 (6 to 14)	46.23% (-12.45 to 144.79)	44.3 (27.84 to 69)	44.89 (27.63 to 63.75)	0.68% (0.11 to 1.25)

Location	Deaths in 1990 (95% UI)	Deaths in 2019 (95% UI)	Relative change (%, 95 CI)	ASMMR in 1990 (95% UI)	ASMMR in 2019 (95% UI)	EAPC (%, 95 CI)
Somalia	372 (234 to 559)	996 (661 to 1426)	167.69% (79.66 to 300.85)	110.92 (69.88 to 166.7)	114.28 (75.88 to 163.72)	0.33% (0.22 to 0.43)
South Africa	249 (194 to 310)	64 (38 to 97)	-74.13% (-85.41 to -58.03)	25.73 (20.05 to 32.05)	6.23 (3.65 to 9.4)	-4.22% (-6.2 to -2.2)
South Sudan	65 (40 to 98)	72 (40 to 124)	11.42% (-37.69 to 100.67)	25.65 (15.81 to 38.79)	19.81 (10.92 to 33.95)	-1% (-1.3 to -0.69)
Sri Lanka	13 (10 to 18)	2 (1 to 2)	-87.64% (-92.54 to -80.06)	3.73 (2.76 to 5.02)	0.56 (0.37 to 0.81)	-6.72% (-6.94 to -6.49)
Sudan	787 (575 to 1073)	242 (135 to 379)	-69.25% (-83.16 to -48.76)	74.43 (54.4 to 101.49)	20.05 (11.21 to 31.4)	-4.35% (-4.71 to -3.99)
Suriname	0 (0 to 1)	0 (0 to 1)	-15.19% (-47.69 to 48.4)	4.79 (2.9 to 6.56)	4.27 (2.91 to 5.98)	-0.36% (-1.01 to 0.3)
Syrian Arab Republic	29 (19 to 41)	3 (2 to 4)	-90% (-94.1 to -82.89)	5.57 (3.76 to 7.91)	1.24 (0.81 to 1.79)	-7.17% (-8.41 to -5.92)

Location	Deaths in 1990 (95% UI)	Deaths in 2019 (95% UI)	Relative change (%, 95 CI)	ASMMR in 1990 (95% UI)	ASMMR in 2019 (95% UI)	EAPC (%, 95 CI)
Tajikistan	3 (2 to 4)	1 (1 to 2)	-61.73% (-76.32 to -39.75)	1.6 (1.18 to 2.14)	0.5 (0.33 to 0.75)	-5.7% (-6.61 to -4.77)
Thailand	21 (15 to 29)	10 (6 to 16)	-51.58% (-72.99 to -16.07)	1.99 (1.38 to 2.77)	1.76 (1.06 to 2.66)	0.22% (-0.56 to 1)
Timor-Leste	22 (14 to 32)	8 (2 to 11)	-65.7% (-91.92 to -37.69)	57.53 (35.49 to 83.9)	19.39 (4.86 to 28.9)	-5.06% (-5.78 to -4.34)
Togo	72 (53 to 96)	66 (41 to 94)	-8.83% (-45.46 to 38.06)	44.34 (32.62 to 58.58)	27.76 (17.32 to 39.59)	-1.44% (-1.75 to -1.13)
Tonga	0 (0 to 0)	0 (0 to 0)	-58.61% (-76.7 to -33.03)	11.56 (8.25 to 15.39)	5.34 (3.26 to 7.96)	-2.94% (-3.11 to -2.78)
Tunisia	20 (14 to 27)	2 (1 to 3)	-89.83% (-94.21 to -82.41)	8.2 (5.85 to 11.21)	1.2 (0.73 to 1.85)	-6.08% (-6.42 to -5.75)
Turkey	231 (167 to 305)	13 (9 to 18)	-94.49% (-96.42 to -91.21)	13.72 (9.91 to 18.08)	1.3 (0.9 to 1.83)	-9.36% (-10.3 to -8.41)

Location	Deaths in 1990 (95% UI)	Deaths in 2019 (95% UI)	Relative change (%, 95 CI)	ASMMR in 1990 (95% UI)	ASMMR in 2019 (95% UI)	EAPC (%, 95 CI)
Turkmenistan	1 (1 to 2)	1 (1 to 1)	-42.04% (-62.58 to -14.11)	0.98 (0.75 to 1.26)	0.68 (0.46 to 0.98)	-2.01% (-2.77 to -1.24)
Tuvalu	0 (0 to 0)	0 (0 to 0)	-83.61% (-90.39 to -72.69)	34.54 (22.24 to 49.75)	7.87 (4.83 to 12)	-4.82% (-4.91 to -4.72)
Uganda	177 (111 to 264)	220 (143 to 330)	24.02% (-28.31 to 116.07)	18.89 (11.77 to 28.1)	13.8 (8.97 to 20.68)	-1.25% (-1.76 to -0.74)
Ukraine	7 (5 to 9)	2 (1 to 3)	-73.3% (-82.79 to -58.8)	1.12 (0.8 to 1.47)	0.49 (0.31 to 0.73)	-4.48% (-5.31 to -3.64)
United Republic of Tanzania	421 (299 to 558)	338 (229 to 479)	-19.76% (-49.95 to 26.29)	34.03 (24.17 to 45.11)	16.18 (10.98 to 22.93)	-1.8% (-2.13 to -1.46)
Uzbekistan	23 (18 to 28)	14 (10 to 20)	-38.11% (-58.07 to -10.69)	3.21 (2.51 to 3.93)	1.98 (1.42 to 2.73)	-1.26% (-2.31 to -0.21)
Vanuatu	1 (0 to 1)	1 (0 to 1)	28.51% (-37.95 to 133.81)	12.91 (7.39 to 19.77)	12.54 (6.35 to 19.55)	-0.84% (-1.22 to -0.46)

Location	Deaths in 1990 (95% UI)	Deaths in 2019 (95% UI)	Relative change (%, 95 CI)	ASMMR in 1990 (95% UI)	ASMMR in 2019 (95% UI)	EAPC (%, 95 CI)
Venezuela (Bolivarian Republic of)	89 (74 to 105)	40 (27 to 57)	-54.61% (-69.68 to -31.74)	15.93 (13.23 to 18.74)	8.46 (5.75 to 12.01)	-2.66% (-3.78 to -1.54)
Viet Nam	57 (34 to 88)	2 (1 to 2)	-97.05% (-98.35 to -94.79)	3.15 (1.87 to 4.87)	0.12 (0.08 to 0.18)	-11.32% (-11.58 to -11.06)
Yemen	294 (151 to 463)	340 (179 to 525)	15.88% (-41.72 to 128.25)	43.08 (22.17 to 67.97)	35.87 (18.83 to 55.28)	-1.47% (-1.86 to -1.09)
Zambia	101 (72 to 137)	57 (36 to 81)	-43.26% (-66.15 to -12.4)	25.67 (18.42 to 34.95)	9.28 (5.8 to 13.09)	-4.08% (-4.7 to -3.46)
Zimbabwe	114 (83 to 149)	228 (154 to 328)	100.37% (32.79 to 210.21)	29.21 (21.41 to 38.17)	50.94 (34.42 to 73.15)	5.21% (3.68 to 6.76)

ASMMR – age-standardized maternal mortality ratio (per 100,000 livebirths), CI – confidence interval, EAPC – estimated annual percentage change, MSMI – maternal sepsis and other maternal infections, UI – uncertain interval..

Table S6. DALYs of MSMI in 131 low- and middle-income countries with EAPC from 1990 and 2019.

Location	DALYs in 1990 (95% UI)	DALYs in 2019 (95% UI)	Relative change (%, 95 CI)	Age-standardized DALYs rate in 1990 (95% UI)	Age-standardized DALYs rate in 2019 (95% UI)	EAPC (%, 95 CI)
Global	2370496 (2053615 to 2710598)	1064670 (895636 to 1256185)	-55.09% (-63.27 to -47.24)	84.83 (73.76 to 96.71)	27.34 (22.97 to 32.3)	-3.89% (-4.18 to -3.59)
Afghanistan	16418 (11923 to 22391)	20019 (13873 to 28883)	21.93% (-16.02 to 84.73)	338.44 (241.57 to 464.01)	115.38 (80.29 to 165.56)	-4.24% (-5.36 to -3.1)
Albania	89 (53 to 140)	33 (18 to 56)	-62.74% (-72 to -53.06)	4.76 (2.89 to 7.49)	2.57 (1.36 to 4.42)	-1.75% (-2.06 to -1.43)
Algeria	20041 (14358 to 27853)	2230 (1591 to 2995)	-88.87% (-92.74 to -83.09)	161.57 (116.6 to 226.29)	9.83 (7.01 to 13.21)	-9.43% (-9.84 to -9.02)
American Samoa	7 (5 to 10)	3 (2 to 4)	-59.52% (-73.44 to -37.76)	29.86 (22.12 to 40)	10.65 (7.5 to 14.51)	-3.96% (-4.16 to -3.76)
Angola	9690 (6601 to 13404)	9081 (5826 to 12553)	-6.29% (-44.11 to 49.12)	213.18 (145.24 to 294.39)	65.06 (41.54 to 91.54)	-3.93% (-4.3 to -3.56)
Armenia	153 (106 to 218)	44 (27 to 72)	-71.19% (-78.97 to -62.87)	8.06 (5.58 to 11.56)	3.16 (1.89 to 5.19)	-3.28% (-3.76 to -2.79)

Location	DALYs in 1990 (95% UI)	DALYs in 2019 (95% UI)	Relative change (%, 95 CI)	Age-standardized DALYs rate in 1990 (95% UI)	Age-standardized DALYs rate in 2019 (95% UI)	EAPC (%, 95 CI)
Azerbaijan	527 (384 to 706)	161 (84 to 263)	-69.42% (-81.12 to -56.74)	12.38 (9.16 to 16.4)	3.12 (1.61 to 5.22)	-5.75% (-6.09 to -5.4)
Bangladesh	31551 (22915 to 41462)	2976 (1819 to 4800)	-90.57% (-94.63 to -84.53)	59.4 (42.99 to 78.49)	3.19 (1.97 to 5.08)	-9.43% (-10.56 to -8.29)
Belarus	240 (151 to 378)	115 (56 to 210)	-51.98% (-66.18 to -37.59)	4.75 (2.97 to 7.62)	2.92 (1.43 to 5.33)	-1.02% (-1.45 to -0.58)
Belize	23 (18 to 30)	35 (27 to 44)	49.37% (8.69 to 106.7)	24.73 (19.43 to 31.03)	14.71 (11.23 to 18.65)	-1.32% (-1.59 to -1.05)
Benin	7674 (5736 to 10128)	6740 (4337 to 10105)	-12.17% (-45.18 to 34.38)	357.69 (268.86 to 470.06)	114.66 (74.31 to 169.32)	-3.3% (-3.63 to -2.97)
Bhutan	159 (93 to 227)	19 (12 to 29)	-88.16% (-93.08 to -78.83)	53.32 (30.61 to 75.91)	4.38 (2.75 to 6.65)	-8.64% (-8.92 to -8.36)
Bolivia (Plurinational State of)	7999 (6158 to 10425)	2750 (1728 to 3827)	-65.62% (-78.67 to -47.39)	260.73 (199.78 to 344.61)	44.6 (27.99 to 61.93)	-6.35% (-6.66 to -6.03)

Location	DALYs in 1990 (95% UI)	DALYs in 2019 (95% UI)	Relative change (%, 95 CI)	Age-standardized DALYs rate in 1990 (95% UI)	Age-standardized DALYs rate in 2019 (95% UI)	EAPC (%, 95 CI)
Bosnia and Herzegovina	227 (172 to 294)	17 (8 to 32)	-92.45% (-96.32 to -87.77)	9.28 (7.06 to 12.03)	1.28 (0.57 to 2.39)	-8.26% (-9.72 to -6.78)
Botswana	821 (542 to 1187)	283 (170 to 427)	-65.49% (-80.59 to -41.43)	116.78 (75.02 to 169.43)	20.54 (12.38 to 30.86)	-6.14% (-6.91 to -5.36)
Brazil	28214 (23637 to 33265)	12030 (9714 to 14919)	-57.36% (-66.81 to -45.63)	34.96 (29.27 to 41.14)	10.59 (8.53 to 13.17)	-2.96% (-3.49 to -2.43)
Bulgaria	300 (228 to 389)	54 (31 to 88)	-82.05% (-87.95 to -75.33)	7.6 (5.69 to 10.08)	2.24 (1.25 to 3.75)	-4.36% (-4.77 to -3.95)
Burkina Faso	12501 (8693 to 17516)	11125 (7012 to 16036)	-11.01% (-45.21 to 37.53)	296.54 (207.15 to 412.01)	100.42 (63.73 to 145.07)	-4.39% (-4.77 to -4.01)
Burundi	13647 (9456 to 18941)	17993 (12645 to 23801)	31.85% (-13.18 to 95.13)	548.82 (380.14 to 761.25)	331.87 (233.11 to 441.11)	-2.1% (-2.6 to -1.59)
Cabo Verde	94 (71 to 123)	11 (8 to 17)	-87.83% (-92.21 to -80.8)	54.91 (42.11 to 71.05)	3.74 (2.54 to 5.4)	-9.66% (-9.92 to -9.41)

Location	DALYs in 1990 (95% UI)	DALYs in 2019 (95% UI)	Relative change (%, 95 CI)	Age-standardized DALYs rate in 1990 (95% UI)	Age-standardized DALYs rate in 2019 (95% UI)	EAPC (%, 95 CI)
Cambodia	13510 (9580 to 19042)	2515 (1719 to 3445)	-81.38% (-88.02 to -70.48)	268.44 (190.82 to 375.99)	27.98 (19.43 to 38.28)	-8.8% (-9.76 to - 7.82)
Cameroon	11767 (8584 to 14959)	13453 (8052 to 19339)	14.33% (-32.03 to 75.06)	249.38 (182.89 to 319.44)	90.66 (54.64 to 130.62)	-3.31% (-3.79 to - 2.82)
Central African Republic	6911 (5005 to 9217)	14023 (9463 to 20549)	102.91% (33.75 to 212.99)	546.49 (393.01 to 724.64)	542.47 (362.86 to 796.05)	0.08% (-0.1 to 0.26)
Chad	15316 (11693 to 19969)	22041 (14941 to 29996)	43.91% (-4.44 to 112.72)	577.46 (436.81 to 750.32)	310.73 (216.71 to 425.41)	-2.11% (-2.51 to - 1.71)
China	127359 (99679 to 159590)	7692 (3983 to 13595)	-93.96% (-96.75 to -90.21)	18.18 (14.33 to 22.78)	1.17 (0.61 to 2.04)	-9.05% (-9.76 to - 8.33)
Colombia	5324 (4329 to 6574)	2111 (1477 to 2905)	-60.35% (-73 to -44.91)	28.52 (23.13 to 35.01)	8.38 (5.86 to 11.54)	-4% (-4.45 to - 3.55)
Comoros	275 (66 to 450)	184 (98 to 278)	-32.98% (-65.19 to 184.65)	141.66 (34.69 to 230.77)	50.93 (27.46 to 76.54)	-4.15% (-4.73 to - 3.57)

Location	DALYs in 1990 (95% UI)	DALYs in 2019 (95% UI)	Relative change (%, 95 CI)	Age-standardized DALYs rate in 1990 (95% UI)	Age-standardized DALYs rate in 2019 (95% UI)	EAPC (%, 95 CI)
Congo	2121 (1518 to 2882)	1782 (1162 to 2612)	-15.99% (-48.45 to 36.44)	192.74 (139.79 to 262.94)	65.09 (42.59 to 95.25)	-3.42% (-4.22 to -2.62)
Costa Rica	269 (212 to 335)	82 (54 to 124)	-69.58% (-79.07 to -58.14)	16.25 (12.85 to 20.22)	3.14 (2.05 to 4.75)	-5.85% (-6.83 to -4.86)
Côte d'Ivoire	8454 (5952 to 11448)	8988 (5585 to 12974)	6.32% (-35.67 to 62.56)	157.05 (110.86 to 211.11)	69.91 (44.16 to 100.63)	-2.41% (-2.86 to -1.97)
Cuba	849 (683 to 1044)	221 (156 to 304)	-73.97% (-81.49 to -64.93)	13.48 (10.84 to 16.49)	4.64 (3.22 to 6.44)	-3.23% (-3.61 to -2.84)
Democratic People's Republic of Korea	3303 (2076 to 4831)	2361 (1495 to 3486)	-28.51% (-57.98 to 27.52)	29.21 (18.36 to 42.48)	18.22 (11.56 to 26.75)	-1.29% (-2.21 to -0.36)
Democratic Republic of the Congo	63316 (43670 to 85897)	158985 (116905 to 209294)	151.1% (72 to 275.69)	379.05 (258.96 to 518.46)	402.31 (294.94 to 534.31)	1.75% (0.55 to 2.96)
Djibouti	694 (430 to 1002)	1013 (525 to 1595)	45.92% (-26.74 to 156.21)	322.19 (203.9 to 457.85)	159.1 (83.01 to 250.86)	-2.17% (-2.69 to -1.65)

Location	DALYs in 1990 (95% UI)	DALYs in 2019 (95% UI)	Relative change (%, 95 CI)	Age-standardized DALYs rate in 1990 (95% UI)	Age-standardized DALYs rate in 2019 (95% UI)	EAPC (%, 95 CI)
Dominica	17 (13 to 23)	10 (7 to 14)	-41.08% (-63.85 to -7.53)	48.04 (35.62 to 63.82)	31.08 (21.77 to 43.53)	-1.23% (-1.51 to - 0.95)
Dominican Republic	1090 (845 to 1392)	753 (516 to 1066)	-30.89% (-53.98 to 2.33)	26.21 (20.36 to 33.42)	12.77 (8.78 to 18.05)	-1.9% (-2.53 to - 1.26)
Ecuador	3113 (2517 to 3804)	1192 (830 to 1642)	-61.72% (-73.92 to -46.89)	58.65 (47.59 to 71.12)	12.57 (8.8 to 17.29)	-4.53% (-5.42 to - 3.64)
Egypt	21047 (16953 to 25905)	2824 (1833 to 4185)	-86.58% (-91.4 to -79.66)	77.98 (62.95 to 95.46)	5.37 (3.51 to 7.93)	-9.48% (-9.97 to - 8.97)
El Salvador	4129 (3329 to 5057)	252 (171 to 363)	-93.9% (-96.09 to -90.78)	148.76 (120.79 to 181.25)	7 (4.75 to 10.05)	-9.88% (-11.19 to - 8.55)
Equatorial Guinea	1304 (906 to 1763)	148 (84 to 232)	-88.68% (-93.68 to -80.14)	675.48 (458.79 to 911.09)	21.6 (12.2 to 34.09)	-12.56% (-13.26 to - 11.86)
Eritrea	10665 (6822 to 15272)	9088 (5979 to 13672)	-14.78% (-47.28 to 40.63)	780.92 (501.98 to 1104.81)	264.56 (174.46 to 397.22)	-2.76% (-3.34 to - 2.19)

Location	DALYs in 1990 (95% UI)	DALYs in 2019 (95% UI)	Relative change (%, 95 CI)	Age-standardized DALYs rate in 1990 (95% UI)	Age-standardized DALYs rate in 2019 (95% UI)	EAPC (%, 95 CI)
Eswatini	296 (211 to 403)	144 (82 to 237)	-51.34% (-72.91 to -15.64)	79.33 (55.2 to 108.4)	22.83 (12.85 to 37.79)	-3.3% (-4.39 to - 2.19)
Ethiopia	203449 (147655 to 284786)	69416 (48951 to 96432)	-65.88% (-78.82 to -47.48)	940.11 (672.73 to 1307.74)	137.26 (97.59 to 190.67)	-6.85% (-7.68 to - 6.02)
Fiji	87 (61 to 121)	46 (32 to 65)	-46.93% (-66.09 to -18.02)	21.96 (15.49 to 30.48)	9.99 (6.93 to 14.14)	-2.94% (-3.08 to - 2.81)
Gabon	588 (432 to 775)	227 (143 to 327)	-61.44% (-75.84 to -40.6)	136.03 (99.94 to 178.62)	23.26 (14.78 to 33.76)	-6.31% (-6.96 to - 5.65)
Gambia	1207 (796 to 1693)	1630 (1117 to 2239)	34.99% (-10.69 to 112.2)	276.16 (184 to 386.51)	149.02 (102 to 204.81)	-2.37% (-2.72 to - 2.01)
Georgia	170 (119 to 238)	102 (73 to 139)	-40.05% (-55.51 to -16.63)	5.97 (4.18 to 8.37)	6.65 (4.79 to 9.11)	2.28% (1.23 to 3.35)
Ghana	9248 (6322 to 12507)	4288 (2918 to 6053)	-53.63% (-71.22 to -30.02)	127.35 (87.01 to 172.12)	23.77 (16.17 to 33.42)	-5.19% (-5.44 to - 4.94)

Location	DALYs in 1990 (95% UI)	DALYs in 2019 (95% UI)	Relative change (%, 95 CI)	Age-standardized DALYs rate in 1990 (95% UI)	Age-standardized DALYs rate in 2019 (95% UI)	EAPC (%, 95 CI)
Grenada	4 (3 to 6)	1 (1 to 2)	-64.62% (-74.63 to -52.99)	9.47 (6.81 to 12.93)	2.73 (1.68 to 4.46)	-3.71% (-4.21 to -3.21)
Guatemala	4975 (3761 to 6276)	1819 (1317 to 2488)	-63.44% (-74.86 to -48.04)	133.72 (102.12 to 169.21)	17.46 (12.65 to 23.89)	-8.11% (-9.01 to -7.2)
Guinea	12843 (9640 to 16569)	13626 (9756 to 18557)	6.1% (-26.17 to 53.78)	454.45 (338.69 to 585.73)	219.38 (156.81 to 301.85)	-1.99% (-2.16 to -1.82)
Guinea-Bissau	1216 (867 to 1660)	728 (492 to 1022)	-40.15% (-62.93 to -7.9)	260.18 (184.38 to 356.49)	73.77 (50.32 to 103.96)	-3.92% (-4.29 to -3.56)
Guyana	161 (120 to 209)	58 (40 to 80)	-64.21% (-76.99 to -45.72)	36.57 (27.42 to 47.1)	13.21 (9.12 to 18.3)	-3.28% (-3.62 to -2.94)
Haiti	12181 (8468 to 17318)	19227 (12865 to 26844)	57.84% (7.02 to 132.82)	391.84 (271.94 to 562.8)	272.11 (181.28 to 382.37)	-0.51% (-0.86 to -0.15)
Honduras	7151 (5504 to 9105)	1100 (669 to 1612)	-84.62% (-90.77 to -75.89)	320.21 (247.83 to 404.98)	19.55 (11.92 to 28.79)	-9.89% (-10.27 to -9.5)

Location	DALYs in 1990 (95% UI)	DALYs in 2019 (95% UI)	Relative change (%, 95 CI)	Age-standardized DALYs rate in 1990 (95% UI)	Age-standardized DALYs rate in 2019 (95% UI)	EAPC (%, 95 CI)
India	915482 (718062 to 1145799)	133547 (96103 to 179525)	-85.41% (-89.99 to -79.19)	210.37 (165.39 to 261.59)	17.59 (12.64 to 23.41)	-8.8% (-9.51 to - 8.08)
Indonesia	72646 (55033 to 96608)	11730 (8829 to 15597)	-83.85% (-89 to -76.56)	73.09 (55.54 to 96.16)	8.46 (6.36 to 11.28)	-7.01% (-7.28 to - 6.73)
Iran (Islamic Republic of)	9172 (7482 to 11344)	1411 (981 to 2047)	-84.62% (-89.02 to -78.39)	34.25 (28.13 to 42.29)	3.08 (2.1 to 4.5)	-8.01% (-8.52 to - 7.49)
Iraq	935 (601 to 1397)	836 (471 to 1381)	-10.63% (-38.53 to 16.76)	11.71 (7.71 to 17.06)	3.48 (2.01 to 5.68)	-4.25% (-4.7 to -3.79)
Jamaica	180 (136 to 229)	50 (33 to 75)	-72.01% (-80.72 to -60.83)	14.21 (10.86 to 18)	3.15 (2.08 to 4.66)	-6.52% (-7.46 to - 5.57)
Jordan	824 (603 to 1108)	381 (267 to 543)	-53.8% (-70.3 to -28.97)	51.33 (37.39 to 69.07)	6.42 (4.54 to 9.13)	-8.26% (-8.78 to - 7.74)
Kazakhstan	382 (212 to 647)	403 (250 to 635)	5.74% (-14.1 to 37.35)	4.36 (2.43 to 7.38)	4.35 (2.68 to 6.88)	0.7% (0.37 to 1.03)
Kenya	16262 (12119 to 21138)	15793 (11299 to 21693)	-2.88% (-34.76 to 43.54)	157.16 (118.16 to 202.78)	60.57 (43.15 to 83.75)	-2.9% (-3.48 to - 2.32)

Location	DALYs in 1990 (95% UI)	DALYs in 2019 (95% UI)	Relative change (%, 95 CI)	Age-standardized DALYs rate in 1990 (95% UI)	Age-standardized DALYs rate in 2019 (95% UI)	EAPC (%, 95 CI)
Kiribati	35 (24 to 48)	35 (24 to 48)	-0.89% (-40.41 to 59.37)	93.72 (65.57 to 128.62)	55.39 (38.15 to 77.75)	-1.96% (-2.16 to -1.76)
Kyrgyzstan	301 (228 to 399)	348 (263 to 465)	15.69% (-10.04 to 47.65)	12.84 (9.84 to 16.92)	9.83 (7.46 to 13.04)	0.62% (-0.11 to 1.35)
Lao People's Democratic Republic	6808 (4769 to 9772)	696 (454 to 939)	-89.77% (-93.75 to -84.54)	330.9 (231.13 to 475.61)	17.09 (11.18 to 23.05)	-10.05% (-10.65 to -9.46)
Lebanon	277 (196 to 386)	83 (48 to 130)	-70.19% (-80.48 to -56.68)	17.6 (12.54 to 24.27)	3.24 (1.87 to 5.2)	-5.7% (-6.06 to -5.35)
Lesotho	2049 (1381 to 2769)	1207 (704 to 1865)	-41.08% (-68.09 to -0.97)	245.12 (163.22 to 330.28)	105.85 (61.51 to 164.62)	-0.98% (-1.84 to -0.11)
Liberia	4506 (3306 to 5991)	7373 (4851 to 10188)	63.63% (3.12 to 140.11)	532.69 (385.18 to 701.72)	305.28 (201.09 to 423.45)	-3.15% (-4.13 to -2.16)
Libya	245 (168 to 344)	100 (65 to 151)	-59.05% (-73.2 to -40.87)	14.8 (10.33 to 20.7)	2.46 (1.58 to 3.71)	-5.76% (-6.44 to -5.06)

Location	DALYs in 1990 (95% UI)	DALYs in 2019 (95% UI)	Relative change (%, 95 CI)	Age-standardized DALYs rate in 1990 (95% UI)	Age-standardized DALYs rate in 2019 (95% UI)	EAPC (%, 95 CI)
Madagascar	17335 (13079 to 22026)	21512 (15228 to 28924)	24.1% (-12.84 to 71.48)	311.04 (238.45 to 392.37)	161.69 (113.08 to 216.44)	-2.23% (-2.63 to -1.82)
Malawi	9541 (6784 to 12668)	7787 (4971 to 11136)	-18.39% (-50.42 to 31.41)	234.04 (166.45 to 307.82)	88.4 (56.38 to 126.81)	-3.22% (-3.74 to -2.69)
Malaysia	3478 (2748 to 4411)	843 (579 to 1190)	-75.75% (-84.13 to -64.15)	38.28 (30.22 to 48.39)	4.91 (3.4 to 6.89)	-7.28% (-7.76 to -6.79)
Maldives	43 (31 to 60)	7 (4 to 11)	-83.64% (-90.48 to -74.14)	48.12 (34.93 to 66.84)	2.95 (1.83 to 4.5)	-9.26% (-10.17 to -8.34)
Mali	34101 (26930 to 42722)	23806 (15524 to 34543)	-30.19% (-55.19 to 3.77)	911.78 (720.04 to 1135.41)	247.57 (163.71 to 354.59)	-4.78% (-5.09 to -4.47)
Marshall Islands	14 (10 to 19)	11 (7 to 16)	-23.06% (-54.83 to 19.89)	74.96 (54 to 100.72)	36.6 (22.24 to 54.3)	-2.69% (-3.29 to -2.09)
Mauritania	7251 (5743 to 9068)	1882 (1169 to 2758)	-74.04% (-83.73 to -60.37)	813.38 (641.15 to 1032.65)	97.87 (62.26 to 142.85)	-7.66% (-7.9 to -7.42)

Location	DALYs in 1990 (95% UI)	DALYs in 2019 (95% UI)	Relative change (%, 95 CI)	Age-standardized DALYs rate in 1990 (95% UI)	Age-standardized DALYs rate in 2019 (95% UI)	EAPC (%, 95 CI)
Mexico	8236 (6559 to 10212)	3762 (2573 to 5468)	-54.32% (-67.54 to -40.05)	17.62 (14.19 to 21.61)	5.56 (3.79 to 8.1)	-3.57% (-3.93 to - 3.21)
Micronesia (Federated States of)	55 (37 to 82)	16 (6 to 25)	-71.4% (-89.11 to -50.96)	125.29 (85.44 to 183.54)	30.79 (11.93 to 50.39)	-5.1% (-5.26 to - 4.94)
Mongolia	1393 (995 to 1875)	327 (219 to 473)	-76.55% (-85.55 to -62.72)	136.71 (97.47 to 181.55)	17.44 (11.78 to 24.94)	-7.99% (-8.41 to - 7.56)
Montenegro	7 (3 to 13)	4 (2 to 7)	-48.22% (-63.18 to -28.59)	2.23 (0.98 to 4.07)	1.38 (0.6 to 2.51)	-1.93% (-2.03 to - 1.83)
Morocco	17686 (13073 to 23272)	1532 (1026 to 2289)	-91.34% (-94.48 to -86.6)	137.23 (100.92 to 179.68)	7.94 (5.33 to 11.87)	-9.53% (-9.8 to -9.26)
Mozambique	18186 (13008 to 24419)	8966 (5806 to 12805)	-50.7% (-69.83 to -25.12)	293.26 (211.15 to 396.59)	61.33 (38.99 to 87.79)	-4.9% (-5.37 to - 4.43)
Myanmar	27813 (18927 to 41437)	7420 (4954 to 10400)	-73.32% (-84.16 to -54.81)	125.64 (85.19 to 188.08)	24.71 (16.49 to 34.65)	-6.04% (-6.55 to - 5.53)
Namibia	826 (484 to 1154)	316 (187 to 505)	-61.75% (-78.57 to -25.54)	125.17 (74.77 to 173.22)	24.22 (14.45 to 38.59)	-5.33% (-5.9 to -4.76)

Location	DALYs in 1990 (95% UI)	DALYs in 2019 (95% UI)	Relative change (%, 95 CI)	Age-standardized DALYs rate in 1990 (95% UI)	Age-standardized DALYs rate in 2019 (95% UI)	EAPC (%, 95 CI)
Nepal	28259 (20404 to 38232)	2704 (1847 to 3717)	-90.43% (-94.01 to -85.64)	296.82 (214.68 to 400.59)	14.95 (10.35 to 20.63)	-10.1% (-10.31 to -9.9)
Nicaragua	1174 (933 to 1447)	217 (150 to 307)	-81.53% (-87.72 to -73.61)	59.28 (47.52 to 73.4)	5.87 (4.08 to 8.27)	-8.33% (-8.9 to -7.76)
Niger	21446 (15450 to 28138)	34010 (22406 to 47752)	58.58% (1.06 to 139.55)	631.46 (456.52 to 832.42)	365.43 (242.41 to 513.78)	-2.14% (-2.39 to -1.89)
Nigeria	49826 (31146 to 77785)	54068 (33555 to 87114)	8.51% (-39.59 to 85.36)	121.76 (75.61 to 190.46)	50.43 (31.62 to 80.55)	-3.8% (-4.54 to -3.05)
North Macedonia	52 (35 to 77)	13 (6 to 23)	-75.28% (-84.44 to -66.23)	5.03 (3.36 to 7.43)	1.36 (0.65 to 2.43)	-5.14% (-5.55 to -4.73)
Pakistan	90857 (70886 to 114389)	52795 (38422 to 71374)	-41.89% (-61.85 to -14.36)	195.07 (154.15 to 245.58)	47.47 (34.32 to 65.06)	-5.14% (-5.59 to -4.68)
Papua New Guinea	4639 (3185 to 6298)	4887 (3400 to 6782)	5.35% (-30.87 to 59.1)	233.08 (162.31 to 315.44)	94.97 (66.19 to 131.73)	-2.48% (-2.91 to -2.06)

Location	DALYs in 1990 (95% UI)	DALYs in 2019 (95% UI)	Relative change (%, 95 CI)	Age-standardized DALYs rate in 1990 (95% UI)	Age-standardized DALYs rate in 2019 (95% UI)	EAPC (%, 95 CI)
Paraguay	1806 (1421 to 2249)	387 (269 to 545)	-78.55% (-85.71 to -67.96)	92.35 (73.09 to 114.26)	10.18 (7.09 to 14.31)	-7.96% (-8.47 to -7.44)
Peru	12150 (9438 to 15352)	1695 (1122 to 2427)	-86.05% (-90.86 to -79.32)	109.35 (85.12 to 139.23)	9.48 (6.23 to 13.49)	-9.13% (-9.61 to -8.65)
Philippines	9780 (7674 to 12290)	5160 (3591 to 7123)	-47.24% (-62.63 to -26.59)	30.72 (24.14 to 38.83)	8.81 (6.21 to 12.15)	-3.98% (-4.13 to -3.83)
Republic of Moldova	243 (183 to 331)	39 (19 to 71)	-83.98% (-90.8 to -76.41)	10.88 (8.17 to 14.79)	2.4 (1.17 to 4.39)	-5.45% (-6.09 to -4.8)
Russian Federation	5106 (3759 to 6988)	2118 (1169 to 3628)	-58.52% (-71.55 to -44.69)	7.11 (5.2 to 9.82)	3.38 (1.85 to 5.73)	-2.52% (-2.78 to -2.27)
Rwanda	26547 (20119 to 35087)	9314 (6099 to 13461)	-64.91% (-81.13 to -44.25)	914.1 (690.76 to 1217.8)	150.13 (98.3 to 215.9)	-6.66% (-7.53 to -5.78)
Saint Lucia	9 (7 to 11)	3 (2 to 4)	-64.92% (-73.65 to -54.34)	11.64 (8.97 to 14.95)	3.38 (2.31 to 4.81)	-3.12% (-3.98 to -2.26)

Location	DALYs in 1990 (95% UI)	DALYs in 2019 (95% UI)	Relative change (%, 95 CI)	Age-standardized DALYs rate in 1990 (95% UI)	Age-standardized DALYs rate in 2019 (95% UI)	EAPC (%, 95 CI)
Saint Vincent and the Grenadines	3 (2 to 5)	2 (1 to 3)	-44.41% (-55.5 to -29.01)	4.73 (2.93 to 7.41)	2.94 (1.82 to 4.74)	-1.54% (-1.84 to -1.24)
Samoa	25 (16 to 36)	6 (3 to 10)	-74.01% (-87.63 to -53.55)	33.34 (21.66 to 48.76)	6.37 (3.45 to 9.65)	-6.66% (-7.19 to -6.12)
Senegal	9833 (6980 to 13019)	7716 (4976 to 10772)	-21.53% (-52.98 to 17.83)	291.8 (210.41 to 384.12)	105.08 (68.12 to 146.66)	-3.18% (-3.52 to -2.85)
Serbia	144 (93 to 216)	39 (20 to 68)	-72.88% (-82.77 to -62.2)	3.38 (2.12 to 5.1)	1.11 (0.56 to 1.95)	-4.13% (-4.48 to -3.79)
Sierra Leone	4503 (3063 to 6222)	8346 (5585 to 11596)	85.32% (14.7 to 192.26)	258.38 (177.06 to 356.92)	193.8 (129.47 to 266.66)	-0.83% (-1.61 to -0.04)
Solomon Islands	384 (245 to 593)	549 (340 to 774)	42.77% (-13.76 to 133.02)	273.33 (173.21 to 418.86)	169.47 (105.5 to 238.71)	-1.16% (-1.79 to -0.54)
Somalia	21025 (13198 to 31532)	57209 (38264 to 81642)	172.1% (85.6 to 307.08)	711.11 (448.81 to 1060.03)	656.45 (433.86 to 934.27)	-0.09% (-0.33 to 0.14)
South Africa	15445 (12139 to 19210)	4478 (2876 to 6526)	-71.01% (-82.29 to -54.44)	75.53 (59.63 to 93.86)	14.05 (9.11 to 20.29)	-4.87% (-6.7 to -3)

Location	DALYs in 1990 (95% UI)	DALYs in 2019 (95% UI)	Relative change (%, 95 CI)	Age-standardized DALYs rate in 1990 (95% UI)	Age-standardized DALYs rate in 2019 (95% UI)	EAPC (%, 95 CI)
South Sudan	3940<br>(2487 to 5855)	4381<br>(2516 to 7329)	11.18%<br>(-35.75 to 94.49)	157.26<br>(99.42 to 234.99)	102.75<br>(58.52 to 173.31)	-1.6%<br>(-1.81 to -1.4)
Sri Lanka	1031<br>(775 to 1370)	262<br>(161 to 412)	-74.62%<br>(-83.72 to -62.18)	10.65<br>(8.02 to 14.15)	2.41<br>(1.47 to 3.82)	-4.86%<br>(-5.03 to -<br>4.69)
Sudan	45698<br>(33584 to 62126)	14354<br>(8353 to 21683)	-68.59%<br>(-82 to -49.19)	507.64<br>(372.68 to 691.06)	69.41<br>(40.29 to 105.9)	-6.67%<br>(-7.2 to -6.14)
Suriname	35<br>(22 to 47)	29<br>(21 to 39)	-15.75%<br>(-43.4 to 34.14)	16.87<br>(10.97 to 22.7)	10.23<br>(7.29 to 13.7)	-1.56%<br>(-2.11 to -<br>1.01)
Syrian Arab Republic	2070<br>(1471 to 2854)	305<br>(199 to 451)	-85.27%<br>(-90.7 to -77.83)	35.75<br>(25.44 to 49.43)	3.94<br>(2.57 to 5.79)	-8.33%<br>(-8.91 to -<br>7.75)
Tajikistan	360<br>(249 to 523)	295<br>(162 to 492)	-17.99%<br>(-42.48 to 4.83)	14.43<br>(10.41 to 20.07)	5.56<br>(3.09 to 9.13)	-3.97%<br>(-4.28 to -<br>3.65)
Thailand	1936<br>(1341 to 2723)	948<br>(610 to 1350)	-51.06%<br>(-66.87 to -29.52)	5.72<br>(3.99 to 7.98)	2.75<br>(1.76 to 3.97)	-2.16%<br>(-2.63 to -<br>1.69)
Timor-Leste	1280<br>(783 to 1865)	448<br>(126 to 648)	-64.99%<br>(-90.07 to -35.84)	340.39<br>(212.82 to 491.68)	73.3<br>(19.78 to 108.43)	-6.59%<br>(-7.35 to -<br>5.84)

Location	DALYs in 1990 (95% UI)	DALYs in 2019 (95% UI)	Relative change (%, 95 CI)	Age-standardized DALYs rate in 1990 (95% UI)	Age-standardized DALYs rate in 2019 (95% UI)	EAPC (%, 95 CI)
Togo	4207 (3106 to 5592)	3809 (2417 to 5365)	-9.47% (-44.5 to 34.57)	255.96 (189.77 to 333.9)	93.36 (58.77 to 131.32)	-3.08% (-3.51 to -2.64)
Tonga	17 (13 to 23)	8 (5 to 11)	-54.24% (-72.31 to -29.63)	43.44 (31.27 to 57.39)	16.18 (10.45 to 23.3)	-3.6% (-3.73 to -3.47)
Tunisia	1349 (992 to 1785)	201 (128 to 300)	-85.11% (-90.78 to -77.12)	31.07 (23.05 to 41.3)	3.29 (2.09 to 4.94)	-6.98% (-7.48 to -6.48)
Turkey	15240 (11092 to 19804)	1687 (1081 to 2550)	-88.93% (-93.07 to -82.87)	50 (36.43 to 64.58)	3.84 (2.45 to 5.83)	-9.76% (-10.55 to -8.96)
Turkmenistan	175 (115 to 266)	125 (74 to 202)	-28.34% (-44.34 to -10.62)	9.1 (6.07 to 13.55)	4.94 (2.95 to 7.97)	-2.09% (-2.26 to -1.91)
Tuvalu	6 (4 to 8)	1 (1 to 2)	-81.92% (-89.12 to -70.77)	118.28 (77.46 to 168.78)	19.08 (12.17 to 28.89)	-5.89% (-6.08 to -5.7)
Uganda	11206 (7174 to 16367)	14231 (9379 to 20996)	26.99% (-24.52 to 117.94)	144.2 (93.41 to 205.97)	70.36 (47.4 to 102.33)	-2.7% (-3.3 to -2.09)
Ukraine	1207 (751 to 1869)	548 (300 to 942)	-54.57% (-66.17 to -41.17)	5.12 (3.16 to 8)	3 (1.63 to 5.14)	-1.55% (-1.89 to -1.2)

Location	DALYs in 1990 (95% UI)	DALYs in 2019 (95% UI)	Relative change (%, 95 CI)	Age-standardized DALYs rate in 1990 (95% UI)	Age-standardized DALYs rate in 2019 (95% UI)	EAPC (%, 95 CI)
United Republic of Tanzania	25194 (17962 to 33239)	20820 (14171 to 29063)	-17.36% (-47.74 to 28.36)	217.49 (155.2 to 286.17)	74.73 (51.42 to 104.18)	-2.85% (-3.24 to -2.46)
Uzbekistan	1957 (1492 to 2561)	1465 (1004 to 2079)	-25.11% (-43.69 to -3.96)	18.46 (14.32 to 23.76)	7.75 (5.34 to 10.98)	-2.46% (-3.03 to -1.9)
Vanuatu	46 (27 to 69)	59 (32 to 91)	28.86% (-35.43 to 120.9)	65.95 (38.52 to 98.69)	39.89 (21.62 to 60.25)	-2.5% (-2.87 to -2.13)
Venezuela (Bolivarian Republic of)	5670 (4687 to 6676)	2784 (1930 to 3774)	-50.9% (-66.05 to -29.06)	55.28 (45.94 to 64.65)	19.46 (13.48 to 26.31)	-3.53% (-4.52 to -2.53)
Viet Nam	4574 (2928 to 6670)	931 (458 to 1712)	-79.65% (-90.08 to -65.6)	11.94 (7.78 to 17.45)	1.82 (0.89 to 3.4)	-6.24% (-6.7 to -5.78)
Yemen	17551 (9043 to 27611)	20472 (11011 to 31330)	16.64% (-40.62 to 133.12)	317.8 (169.73 to 493.82)	127.58 (69.25 to 194.66)	-3.93% (-4.2 to -3.65)
Zambia	6330 (4570 to 8594)	3835 (2511 to 5317)	-39.42% (-61.7 to -9)	169.54 (122.98 to 227)	39.57 (26.07 to 54.41)	-5.57% (-6.28 to -4.86)
Zimbabwe	7014 (5152 to 9279)	13729 (9441 to 19646)	95.75% (31.05 to 199.64)	146.06 (108.54 to 190.63)	170.19 (115.63 to 242.25)	4.02% (2.44 to 5.62)

CI – confidence interval, DALYs – disability-adjusted life years (per 100,000 population), EAPC – estimated annual percentage change, MSMI – maternal sepsis and other maternal infections, UI – uncertain interval.

Table S7. Incidence of MSMI in 131 low- and middle-income countries and territories with EAPC in age group of 10-14 years from 1990 to 2019.

Location	Incident cases in 1990 (95% UI)	Incident cases in 2019 (95% UI)	Relative change (%, 95 CI)	ASIR in 1990 (95% UI)	ASIR in 2019 (95% UI)	EAPC (%, 95 CI)
Afghanistan	105 (74 to 132)	292 (204 to 366)	177.13% (145.75 to 219.25)	4.3 (3 to 5.4)	3.5 (2.4 to 4.4)	0.01% (-0.31 to 0.33)
Albania	5 (4 to 6)	2 (1 to 2)	-60.14% (-64.99 to -54.54)	0.9 (0.7 to 1.1)	0.8 (0.6 to 1)	0.1% (-0.1 to 0.3)
Algeria	72 (50 to 90)	45 (33 to 56)	-37% (-44.49 to -27.84)	1.4 (1 to 1.7)	0.8 (0.6 to 1)	-2.16% (-2.46 to -1.85)
American Samoa	1 (0 to 1)	1 (0 to 1)	-6.69% (-19.51 to 9.11)	6.9 (4.7 to 8.8)	7.3 (5 to 9.4)	0.42% (0.31 to 0.53)
Angola	369 (254 to 468)	938 (646 to 1213)	154.42% (122.51 to 192.73)	15.6 (10.8 to 19.8)	13.4 (9.2 to 17.3)	-0.7% (-0.8 to -0.61)
Armenia	4 (3 to 5)	1 (1 to 2)	-66.42% (-70.35 to -61.53)	0.7 (0.5 to 0.9)	0.4 (0.3 to 0.6)	-2.57% (-2.86 to -2.27)
Azerbaijan	1 (1 to 2)	1 (1 to 2)	1.05% (-12.89 to 15.84)	0.1 (0.1 to 0.1)	0.1 (0.1 to 0.2)	0.25% (-0.17 to 0.66)
Bangladesh	2469 (1639 to 3256)	1915 (1330 to 2458)	-22.43% (-32.24 to -9.09)	10.5 (7 to 13.8)	8.8 (6.1 to 11.3)	-0.65% (-0.71 to -0.6)

Location	Incident cases in 1990 (95% UI)	Incident cases in 2019 (95% UI)	Relative change (%, 95 CI)	ASIR in 1990 (95% UI)	ASIR in 2019 (95% UI)	EAPC (%, 95 CI)
Belarus	3 (2 to 4)	1 (1 to 1)	-60.19% (-65.29 to -54.36)	0.3 (0.2 to 0.3)	0.2 (0.1 to 0.2)	-2.36% (-2.84 to -1.88)
Belize	6 (4 to 7)	7 (5 to 8)	18.35% (2 to 37.28)	14.1 (9.7 to 17.9)	11 (7.7 to 14)	-0.93% (-1 to -0.86)
Benin	541 (347 to 759)	1258 (808 to 1783)	132.53% (95.79 to 171.1)	46.1 (29.6 to 64.6)	44.2 (28.3 to 62.6)	-0.05% (-0.1 to 0)
Bhutan	8 (6 to 10)	4 (3 to 5)	-49.71% (-55.6 to -42.71)	6.6 (4.5 to 8.3)	4.2 (2.9 to 5.3)	-1.8% (-2 to -1.6)
Bolivia (Plurinational State of)	297 (209 to 378)	326 (227 to 410)	9.69% (-3.55 to 23.83)	22.1 (15.5 to 28.1)	16.9 (11.8 to 21.3)	-0.9% (-1.04 to -0.75)
Bosnia and Herzegovina	6 (5 to 7)	1 (1 to 2)	-75.67% (-78.53 to -72.41)	1.1 (0.9 to 1.3)	0.6 (0.5 to 0.8)	-2.32% (-2.53 to -2.11)
Botswana	20 (14 to 25)	18 (13 to 22)	-10.53% (-21.98 to 3.26)	7 (4.9 to 8.7)	5.2 (3.7 to 6.4)	-1.11% (-1.18 to -1.04)
Brazil	4869 (3644 to 5896)	4071 (3045 to 4779)	-16.39% (-22.06 to -9.29)	18.9 (14.1 to 22.8)	17.4 (13 to 20.4)	-0.13% (-0.31 to 0.06)
Bulgaria	102 (75 to 122)	40 (29 to 48)	-60.61% (-65.02 to -55.08)	12.1 (8.9 to 14.4)	8.4 (6.1 to 10)	-1.58% (-1.8 to -1.35)
Burkina Faso	458 (294 to 634)	817 (522 to 1170)	78.37% (47.71 to 111.87)	19.7 (12.6 to 27.3)	16 (10.2 to 22.9)	-0.86% (-0.91 to -0.8)

Location	Incident cases in 1990 (95% UI)	Incident cases in 2019 (95% UI)	Relative change (%, 95 CI)	ASIR in 1990 (95% UI)	ASIR in 2019 (95% UI)	EAPC (%, 95 CI)
Burundi	163 (114 to 207)	302 (205 to 386)	85.3% (59.33 to 111.22)	12.3 (8.6 to 15.6)	11.1 (7.5 to 14.2)	-0.5% (-0.67 to -0.33)
Cabo Verde	11 (7 to 16)	7 (5 to 10)	-37.43% (-46.51 to -26.04)	14.7 (9.2 to 20.8)	9 (5.9 to 12.4)	-1.95% (-2.22 to -1.68)
Cambodia	96 (65 to 123)	74 (51 to 95)	-22.67% (-32 to -10.44)	4.1 (2.8 to 5.2)	3 (2.1 to 3.9)	-1.2% (-1.31 to -1.09)
Cameroon	451 (291 to 630)	937 (613 to 1281)	107.57% (78.86 to 147.77)	19 (12.3 to 26.5)	15.8 (10.3 to 21.6)	-0.67% (-0.76 to -0.58)
Central African Republic	88 (62 to 111)	158 (110 to 204)	80.19% (55.96 to 106.49)	14.5 (10.2 to 18.3)	14.2 (9.9 to 18.3)	-0.03% (-0.08 to 0.01)
Chad	310 (199 to 436)	837 (534 to 1178)	170.39% (128.18 to 220.5)	21.2 (13.6 to 29.9)	20.3 (13 to 28.6)	-0.18% (-0.23 to -0.14)
China	1765 (1219 to 2250)	738 (516 to 936)	-58.21% (-59.48 to -56.76)	1.1 (0.8 to 1.5)	0.7 (0.5 to 0.9)	-0.6% (-0.86 to -0.33)
Colombia	768 (555 to 949)	671 (473 to 827)	-12.62% (-24.42 to 0.63)	13.3 (9.6 to 16.4)	12.5 (8.8 to 15.4)	-0.19% (-0.34 to -0.04)
Comoros	19 (14 to 23)	13 (9 to 16)	-33.39% (-43.38 to -23.51)	18.2 (13 to 22.3)	11.1 (7.7 to 13.8)	-1.64% (-1.71 to -1.56)
Congo	81 (56 to 103)	109 (74 to 143)	34.77% (14.04 to 56.73)	14.7 (10.1 to 18.7)	11 (7.5 to 14.4)	-1.17% (-1.27 to -1.07)

Location	Incident cases in 1990 (95% UI)	Incident cases in 2019 (95% UI)	Relative change (%, 95 CI)	ASIR in 1990 (95% UI)	ASIR in 2019 (95% UI)	EAPC (%, 95 CI)
Costa Rica	77 (55 to 97)	63 (46 to 79)	-17.55% (-28.08 to -1.72)	14 (10 to 17.6)	12.2 (8.8 to 15.2)	-0.61% (-0.82 to -0.41)
Côte d'Ivoire	691 (446 to 968)	1078 (703 to 1525)	56.03% (29.6 to 85.98)	24.6 (15.9 to 34.4)	20.8 (13.6 to 29.5)	-0.75% (-0.83 to -0.66)
Cuba	220 (155 to 277)	131 (92 to 165)	-40.46% (-47.43 to -32.61)	18 (12.7 to 22.8)	15 (10.6 to 18.9)	-0.15% (-0.4 to 0.1)
Democratic People's Republic of Korea	40 (27 to 51)	14 (9 to 18)	-64.75% (-69.49 to -58.77)	1.2 (0.8 to 1.6)	0.6 (0.4 to 0.8)	-2.27% (-2.49 to -2.05)
Democratic Republic of the Congo	1235 (874 to 1568)	2039 (1416 to 2631)	65.16% (43.06 to 90.4)	13.7 (9.7 to 17.4)	10.9 (7.6 to 14.1)	-0.81% (-0.94 to -0.68)
Djibouti	17 (12 to 23)	23 (16 to 29)	32.07% (13.69 to 57.3)	16.8 (11.6 to 22.2)	11.8 (8.2 to 14.7)	-1.72% (-1.97 to -1.48)
Dominica	2 (2 to 3)	1 (1 to 1)	-57.8% (-63.34 to -51.25)	21 (14.8 to 26.4)	14.8 (10.5 to 18.8)	-1.28% (-1.36 to -1.2)
Dominican Republic	133 (93 to 171)	132 (94 to 165)	-0.58% (-12.47 to 15.28)	9.7 (6.8 to 12.5)	8.9 (6.3 to 11.1)	-0.3% (-0.4 to -0.2)
Ecuador	172 (121 to 215)	215 (152 to 270)	24.56% (9.1 to 42.36)	8.9 (6.3 to 11.1)	8.7 (6.2 to 10.9)	0.02% (-0.06 to 0.1)
Egypt	398 (276 to 500)	423 (299 to 532)	6.32% (-7.24 to 20.41)	3.7 (2.6 to 4.6)	2.7 (1.9 to 3.4)	-1.27% (-1.39 to -1.14)

Location	Incident cases in 1990 (95% UI)	Incident cases in 2019 (95% UI)	Relative change (%, 95 CI)	ASIR in 1990 (95% UI)	ASIR in 2019 (95% UI)	EAPC (%, 95 CI)
El Salvador	146 (105 to 184)	86 (59 to 107)	-41.52% (-49.95 to -33.08)	14 (10.1 to 17.6)	10.3 (7.1 to 12.9)	-0.71% (-0.9 to -0.52)
Equatorial Guinea	17 (12 to 21)	33 (23 to 43)	101.83% (76.47 to 130.88)	16.5 (11.6 to 21.1)	13.2 (9.2 to 17)	-0.89% (-0.98 to -0.81)
Eritrea	113 (78 to 144)	147 (103 to 190)	30.92% (12.63 to 48.57)	16.4 (11.3 to 20.9)	11.3 (7.9 to 14.6)	-1.18% (-1.26 to -1.1)
Eswatini	27 (19 to 35)	21 (15 to 27)	-22.16% (-31.33 to -10.9)	14.1 (9.8 to 17.9)	10.7 (7.6 to 13.6)	-0.95% (-1.07 to -0.82)
Ethiopia	2248 (1578 to 2856)	3042 (2103 to 3913)	35.3% (24.14 to 45.04)	18.7 (13.1 to 23.8)	13.5 (9.4 to 17.4)	-1.27% (-1.33 to -1.2)
Fiji	1 (1 to 1)	1 (1 to 1)	-29.94% (-39.23 to -20.52)	0.8 (0.5 to 1)	0.6 (0.4 to 0.7)	-1.38% (-1.48 to -1.28)
Gabon	33 (23 to 42)	30 (20 to 38)	-10.21% (-23 to 5.94)	15.7 (10.8 to 19.7)	10 (6.9 to 13)	-1.38% (-1.47 to -1.29)
Gambia	42 (28 to 59)	62 (40 to 86)	45.8% (23.31 to 72.61)	18 (11.8 to 25.1)	13.4 (8.7 to 18.7)	-1.18% (-1.25 to -1.11)
Georgia	20 (14 to 25)	8 (6 to 10)	-59.65% (-64.85 to -53.57)	3 (2.1 to 3.7)	2.3 (1.6 to 2.9)	-0.89% (-1.03 to -0.75)
Ghana	364 (236 to 509)	450 (292 to 631)	23.81% (4.01 to 43.2)	11 (7.1 to 15.4)	8.1 (5.3 to 11.4)	-0.94% (-1.06 to -0.83)

Location	Incident cases in 1990 (95% UI)	Incident cases in 2019 (95% UI)	Relative change (%, 95 CI)	ASIR in 1990 (95% UI)	ASIR in 2019 (95% UI)	EAPC (%, 95 CI)
Global	57887 (41262 to 72280)	62939 (44439 to 79300)	8.73% (5.98 to 11.06)	6.8 (4.8 to 8.5)	6.6 (4.7 to 8.4)	-0.07% (-0.14 to 0)
Grenada	2 (1 to 2)	1 (1 to 1)	-51.67% (-57.82 to -43.9)	12.3 (8.5 to 15.5)	8.7 (6.1 to 11)	-1.08% (-1.23 to -0.92)
Guatemala	319 (230 to 396)	389 (278 to 482)	21.87% (5.03 to 38.61)	17.7 (12.8 to 22)	13.6 (9.7 to 16.9)	-0.79% (-0.89 to -0.68)
Guinea	444 (280 to 626)	827 (521 to 1153)	86.45% (56.97 to 123.67)	31.9 (20.2 to 45)	29.3 (18.5 to 40.9)	-0.36% (-0.44 to -0.28)
Guinea-Bissau	44 (28 to 62)	56 (36 to 79)	27.45% (10.08 to 50.04)	18.8 (11.9 to 26.2)	14.4 (9.2 to 20)	-1.08% (-1.17 to -0.98)
Guyana	33 (23 to 42)	17 (12 to 21)	-49.77% (-56.16 to -42.68)	23.2 (16.3 to 29.5)	16.1 (11.4 to 20.3)	-0.69% (-0.96 to -0.42)
Haiti	188 (132 to 239)	251 (177 to 317)	33.66% (14.85 to 52.16)	13.8 (9.7 to 17.5)	11.8 (8.3 to 14.9)	-0.79% (-0.9 to -0.68)
Honduras	187 (133 to 236)	239 (169 to 294)	27.4% (11.73 to 49.58)	17.3 (12.2 to 21.8)	14.8 (10.5 to 18.2)	-0.66% (-0.71 to -0.62)
India	8621 (6181 to 10753)	4941 (3536 to 6198)	-42.68% (-44.73 to -40.57)	5.4 (3.9 to 6.8)	2.8 (2 to 3.5)	-2.38% (-2.72 to -2.04)
Indonesia	1586 (1097 to 2025)	880 (619 to 1113)	-44.53% (-46.79 to -41.83)	4.8 (3.3 to 6.1)	2.8 (2 to 3.5)	-2.11% (-2.2 to -2.02)

Location	Incident cases in 1990 (95% UI)	Incident cases in 2019 (95% UI)	Relative change (%, 95 CI)	ASIR in 1990 (95% UI)	ASIR in 2019 (95% UI)	EAPC (%, 95 CI)
Iran (Islamic Republic of)	1012 (721 to 1257)	433 (306 to 547)	-57.21% (-59.47 to -55.34)	8 (5.7 to 9.9)	4.4 (3.1 to 5.5)	-2.14% (-2.36 to -1.92)
Iraq	329 (232 to 417)	426 (300 to 537)	29.61% (14.82 to 47.78)	8.3 (5.9 to 10.6)	6.4 (4.5 to 8)	-0.63% (-0.85 to -0.41)
Jamaica	65 (45 to 84)	31 (22 to 39)	-53.15% (-58.92 to -45.15)	15.8 (11 to 20.2)	10.1 (7.1 to 12.7)	-1.55% (-1.63 to -1.47)
Jordan	8 (5 to 11)	23 (16 to 29)	178.77% (138.61 to 239.39)	1 (0.7 to 1.3)	1.2 (0.9 to 1.6)	0.88% (0.73 to 1.04)
Kazakhstan	16 (11 to 20)	10 (8 to 13)	-33.89% (-42.29 to -23.56)	0.6 (0.4 to 0.8)	0.4 (0.3 to 0.5)	-1.8% (-2.05 to -1.55)
Kenya	965 (688 to 1217)	1178 (833 to 1486)	22.05% (18.78 to 25.03)	17.3 (12.3 to 21.8)	12.5 (8.8 to 15.8)	-1.2% (-1.27 to -1.13)
Kiribati	0 (0 to 1)	0 (0 to 1)	22.49% (6.62 to 41.04)	2.8 (1.9 to 3.7)	2.4 (1.7 to 3.1)	-0.79% (-1.06 to -0.53)
Kyrgyzstan	1 (1 to 2)	1 (1 to 1)	-3.7% (-15.84 to 8.68)	0.2 (0.1 to 0.2)	0.1 (0.1 to 0.1)	-0.75% (-0.89 to -0.6)
Lao People's Democratic Republic	42 (28 to 54)	43 (30 to 55)	4.37% (-9.86 to 23.83)	4.6 (3.1 to 6)	3.9 (2.7 to 5)	-0.54% (-0.74 to -0.34)

Location	Incident cases in 1990 (95% UI)	Incident cases in 2019 (95% UI)	Relative change (%, 95 CI)	ASIR in 1990 (95% UI)	ASIR in 2019 (95% UI)	EAPC (%, 95 CI)
Lebanon	24 (17 to 30)	19 (13 to 24)	-23.54% (-33.1 to -12.56)	4.1 (2.9 to 5.1)	2.8 (2 to 3.6)	-1.86% (-2.27 to -1.44)
Lesotho	49 (34 to 62)	32 (23 to 41)	-33.32% (-43.13 to -22.92)	12.8 (9 to 16.3)	9.8 (6.9 to 12.6)	-0.91% (-0.95 to -0.88)
Liberia	54 (35 to 76)	83 (53 to 116)	52.91% (31.29 to 79.3)	12 (7.8 to 16.9)	9 (5.8 to 12.6)	-1.22% (-1.37 to -1.07)
Libya	30 (21 to 38)	9 (7 to 12)	-69.22% (-73.08 to -64.9)	3.2 (2.2 to 4)	1.3 (0.9 to 1.6)	-2.93% (-3.06 to -2.79)
Madagascar	529 (366 to 695)	974 (661 to 1256)	84.08% (57.6 to 114.24)	19.4 (13.4 to 25.4)	18.1 (12.3 to 23.3)	-0.27% (-0.32 to -0.21)
Malawi	554 (380 to 711)	867 (583 to 1136)	56.5% (34.07 to 78.84)	25 (17.1 to 32)	21.9 (14.7 to 28.7)	-0.59% (-0.65 to -0.52)
Malaysia	93 (64 to 120)	67 (45 to 89)	-27.74% (-39.22 to -16.59)	2.9 (2 to 3.7)	1.8 (1.2 to 2.4)	-1.43% (-1.67 to -1.18)
Maldives	1 (1 to 1)	0 (0 to 1)	-45.72% (-52.8 to -37.61)	1.7 (1.2 to 2.2)	0.9 (0.6 to 1.1)	-2.41% (-2.71 to -2.11)
Mali	439 (288 to 611)	1001 (629 to 1412)	128.19% (97.12 to 170.36)	21.6 (14.2 to 30.1)	19.7 (12.3 to 27.7)	-0.36% (-0.39 to -0.33)
Marshall Islands	1 (1 to 1)	1 (0 to 1)	-25.36% (-34.59 to -12.36)	8.6 (5.9 to 10.9)	7.7 (5.4 to 9.8)	-0.72% (-0.84 to -0.6)

Location	Incident cases in 1990 (95% UI)	Incident cases in 2019 (95% UI)	Relative change (%, 95 CI)	ASIR in 1990 (95% UI)	ASIR in 2019 (95% UI)	EAPC (%, 95 CI)
Mauritania	85 (55 to 114)	113 (74 to 153)	32.56% (12.01 to 56.76)	18.5 (11.9 to 24.7)	14.1 (9.3 to 19.2)	-0.98% (-1.02 to -0.94)
Mexico	2278 (1604 to 2897)	1819 (1223 to 2385)	-20.18% (-26.36 to -15.06)	13.7 (9.7 to 17.4)	11.5 (7.7 to 15.1)	-0.71% (-0.79 to -0.63)
Micronesia (Federated States of)	4 (3 to 6)	3 (2 to 3)	-42.18% (-49.99 to -33.48)	19.2 (13.1 to 24.7)	16.4 (11.7 to 20.8)	-0.5% (-0.53 to -0.47)
Mongolia	13 (10 to 17)	11 (8 to 14)	-16.19% (-26.68 to -5.91)	3 (2.2 to 3.7)	2.3 (1.6 to 2.8)	-1.3% (-1.53 to -1.06)
Montenegro	2 (1 to 2)	1 (0 to 1)	-58.82% (-63.85 to -53.88)	2 (1.5 to 2.5)	1.3 (0.9 to 1.5)	-2.08% (-2.24 to -1.92)
Morocco	163 (115 to 203)	123 (87 to 153)	-24.65% (-32.58 to -14.14)	3.4 (2.4 to 4.2)	2.7 (1.9 to 3.3)	-0.89% (-1.05 to -0.73)
Mozambique	658 (448 to 853)	1227 (839 to 1599)	86.38% (60.98 to 115.42)	21.6 (14.7 to 28)	17.8 (12.2 to 23.2)	-0.54% (-0.68 to -0.41)
Myanmar	293 (200 to 374)	225 (156 to 291)	-23.48% (-32.89 to -11.39)	3.8 (2.6 to 4.8)	3 (2.1 to 3.9)	-0.73% (-0.77 to -0.69)
Namibia	99 (70 to 125)	115 (80 to 148)	15.73% (-0.17 to 31.05)	33.2 (23.4 to 42)	27.3 (19.1 to 35.1)	-0.46% (-0.61 to -0.3)
Nepal	309 (207 to 404)	295 (207 to 383)	-4.4% (-17.04 to 11.46)	7.4 (5 to 9.7)	6.6 (4.6 to 8.5)	-0.39% (-0.52 to -0.26)

Location	Incident cases in 1990 (95% UI)	Incident cases in 2019 (95% UI)	Relative change (%, 95 CI)	ASIR in 1990 (95% UI)	ASIR in 2019 (95% UI)	EAPC (%, 95 CI)
Nicaragua	151 (106 to 190)	138 (97 to 172)	-8.54% (-21.39 to 4.22)	16.7 (11.7 to 21)	14.1 (9.9 to 17.6)	-0.73% (-0.8 to -0.66)
Niger	457 (296 to 632)	1218 (795 to 1687)	166.58% (123.02 to 217.86)	22.7 (14.7 to 31.4)	20.5 (13.4 to 28.5)	-0.46% (-0.63 to -0.29)
Nigeria	4071 (2765 to 5424)	7745 (5368 to 10226)	90.24% (82.79 to 98.49)	19.9 (13.5 to 26.5)	16.4 (11.4 to 21.7)	-1.13% (-1.54 to -0.72)
North Macedonia	13 (9 to 15)	4 (3 to 5)	-66.29% (-71.17 to -61.59)	4.9 (3.6 to 5.9)	2.6 (1.9 to 3.2)	-2.48% (-2.6 to -2.36)
Pakistan	1408 (1011 to 1759)	2029 (1431 to 2564)	44.16% (34.58 to 55.72)	5.8 (4.1 to 7.2)	4.8 (3.4 to 6.1)	-0.72% (-0.81 to -0.64)
Papua New Guinea	42 (29 to 53)	76 (53 to 97)	80.56% (54.37 to 108.3)	5.3 (3.7 to 6.6)	4.3 (3 to 5.5)	-0.52% (-0.62 to -0.43)
Paraguay	48 (36 to 60)	46 (33 to 58)	-4.84% (-20.18 to 9.64)	5.9 (4.4 to 7.3)	4.8 (3.4 to 6.1)	-0.76% (-0.83 to -0.68)
Peru	289 (205 to 365)	234 (162 to 300)	-19.17% (-31.73 to -8.09)	7 (4.9 to 8.8)	5.3 (3.7 to 6.8)	-1.04% (-1.17 to -0.91)
Philippines	233 (162 to 296)	319 (221 to 405)	37.04% (33.78 to 41.07)	1.9 (1.3 to 2.4)	1.9 (1.3 to 2.4)	0.09% (-0.04 to 0.21)

Location	Incident cases in 1990 (95% UI)	Incident cases in 2019 (95% UI)	Relative change (%, 95 CI)	ASIR in 1990 (95% UI)	ASIR in 2019 (95% UI)	EAPC (%, 95 CI)
Republic of Moldova	5 (3 to 6)	2 (1 to 2)	-67.52% (-71.29 to -63.14)	0.8 (0.6 to 0.9)	0.5 (0.4 to 0.7)	-2.01% (-2.33 to -1.69)
Russian Federation	86 (62 to 104)	38 (27 to 46)	-56.01% (-57.19 to -55.2)	0.5 (0.4 to 0.6)	0.3 (0.2 to 0.3)	-2.62% (-2.91 to -2.33)
Rwanda	192 (133 to 244)	197 (138 to 251)	2.73% (-9.88 to 19.48)	11.3 (7.8 to 14.3)	8.2 (5.7 to 10.4)	-1.75% (-2.05 to -1.46)
Saint Lucia	3 (2 to 4)	1 (1 to 2)	-61.44% (-66.97 to -56.2)	13.2 (9.3 to 16.8)	8.5 (5.9 to 10.9)	-1.42% (-1.51 to -1.32)
Saint Vincent and the Grenadines	4 (3 to 5)	2 (1 to 2)	-52.79% (-58.71 to -45.53)	18.4 (12.7 to 23.2)	14.6 (10.4 to 18.4)	-0.73% (-0.81 to -0.65)
Samoa	0 (0 to 0)	0 (0 to 0)	-20.49% (-30.96 to -7.64)	1 (0.7 to 1.3)	0.7 (0.5 to 0.9)	-1.28% (-1.38 to -1.19)
Senegal	314 (203 to 439)	370 (233 to 525)	17.89% (-1.17 to 40.19)	17.1 (11.1 to 23.9)	12.5 (7.9 to 17.7)	-1.15% (-1.21 to -1.09)
Serbia	34 (22 to 45)	13 (8 to 17)	-62.18% (-67.03 to -56.17)	3.1 (2 to 4.1)	1.8 (1.2 to 2.5)	-1.78% (-1.84 to -1.72)
Sierra Leone	154 (98 to 213)	284 (181 to 393)	84.27% (54.52 to 116.15)	19.2 (12.3 to 26.7)	17 (10.8 to 23.6)	-0.54% (-0.63 to -0.45)
Solomon Islands	5 (3 to 6)	6 (5 to 8)	35.55% (17.46 to 57.78)	6.2 (4.2 to 8.1)	5.1 (3.6 to 6.5)	-0.82% (-0.94 to -0.71)

Location	Incident cases in 1990 (95% UI)	Incident cases in 2019 (95% UI)	Relative change (%, 95 CI)	ASIR in 1990 (95% UI)	ASIR in 2019 (95% UI)	EAPC (%, 95 CI)
Somalia	320 (219 to 408)	738 (512 to 946)	130.99% (100.34 to 166.11)	19.7 (13.5 to 25.2)	16 (11.1 to 20.5)	-0.63% (-0.8 to -0.46)
South Africa	449 (320 to 568)	376 (269 to 473)	-16.22% (-20.51 to -11.22)	6.8 (4.8 to 8.5)	5 (3.6 to 6.4)	-1.14% (-1.29 to -1)
South Sudan	234 (164 to 294)	380 (267 to 481)	62.58% (39.1 to 88.38)	18.6 (13.1 to 23.4)	19 (13.3 to 24.1)	-0.07% (-0.22 to 0.08)
Sri Lanka	44 (30 to 56)	27 (19 to 35)	-38.19% (-46.89 to -27.73)	1.6 (1.1 to 2)	1.1 (0.7 to 1.4)	-1.62% (-1.73 to -1.51)
Sudan	245 (174 to 302)	292 (209 to 362)	19.32% (5.02 to 36.07)	5.6 (4 to 6.9)	3.8 (2.7 to 4.7)	-1.43% (-1.51 to -1.36)
Suriname	23 (16 to 29)	22 (16 to 28)	-3.22% (-15.06 to 10.71)	35.6 (24.9 to 45.1)	31.4 (22.1 to 40)	-0.6% (-0.77 to -0.44)
Syrian Arab Republic	118 (83 to 148)	60 (42 to 76)	-49.22% (-56.31 to -42.47)	4 (2.8 to 5)	3 (2.1 to 3.8)	-0.92% (-1.09 to -0.75)
Tajikistan	8 (5 to 9)	9 (6 to 11)	12.6% (-0.82 to 26.35)	0.7 (0.5 to 0.8)	0.5 (0.4 to 0.7)	-0.93% (-1.14 to -0.71)
Thailand	303 (205 to 393)	167 (115 to 210)	-44.96% (-51.87 to -33.8)	3.6 (2.5 to 4.7)	3.2 (2.2 to 4)	-0.22% (-0.37 to -0.07)

Location	Incident cases in 1990 (95% UI)	Incident cases in 2019 (95% UI)	Relative change (%, 95 CI)	ASIR in 1990 (95% UI)	ASIR in 2019 (95% UI)	EAPC (%, 95 CI)
Timor-Leste	6 (4 to 8)	7 (5 to 9)	13.87% (-1.05 to 29.99)	3.7 (2.5 to 4.8)	2.7 (1.8 to 3.6)	-1.14% (-1.24 to -1.04)
Togo	138 (89 to 199)	182 (117 to 250)	31.28% (13.18 to 57.04)	15.7 (10.1 to 22.6)	11.8 (7.6 to 16.3)	-1.12% (-1.22 to -1.02)
Tonga	1 (1 to 1)	1 (0 to 1)	-29.57% (-38.06 to -20.13)	4.4 (3 to 5.6)	3.4 (2.4 to 4.3)	-0.86% (-0.91 to -0.82)
Tunisia	183 (129 to 230)	55 (39 to 69)	-70% (-73.96 to -66.56)	11.8 (8.3 to 14.8)	4.3 (3.1 to 5.4)	-4.21% (-4.54 to -3.87)
Turkey	395 (305 to 454)	165 (125 to 191)	-58.39% (-63.96 to -53.05)	3.8 (2.9 to 4.3)	2.1 (1.6 to 2.5)	-1.52% (-1.66 to -1.37)
Turkmenistan	3 (2 to 4)	3 (2 to 3)	-14.44% (-26.06 to -1.22)	0.4 (0.3 to 0.5)	0.4 (0.3 to 0.4)	-0.48% (-0.61 to -0.34)
Tuvalu	0 (0 to 0)	0 (0 to 0)	4.06% (-9.23 to 19.06)	3.3 (2.2 to 4.2)	3.3 (2.3 to 4.2)	0.1% (0.07 to 0.13)
Uganda	905 (637 to 1150)	1500 (1018 to 1954)	65.71% (38.86 to 92.18)	21.5 (15.1 to 27.3)	16 (10.9 to 20.9)	-1.08% (-1.18 to -0.99)
Ukraine	27 (19 to 33)	10 (7 to 13)	-62.53% (-67.1 to -57.6)	0.5 (0.3 to 0.6)	0.3 (0.2 to 0.4)	-2.4% (-2.64 to -2.16)
United Republic of Tanzania	1129 (779 to 1442)	2057 (1383 to 2646)	82.21% (55.39 to 111.23)	18.7 (12.9 to 23.8)	16.8 (11.3 to 21.6)	-0.23% (-0.3 to -0.16)

Location	Incident cases in 1990 (95% UI)	Incident cases in 2019 (95% UI)	Relative change (%, 95 CI)	ASIR in 1990 (95% UI)	ASIR in 2019 (95% UI)	EAPC (%, 95 CI)
Uzbekistan	15 (10 to 18)	14 (10 to 17)	-3.7% (-14.91 to 9.6)	0.3 (0.2 to 0.4)	0.3 (0.2 to 0.4)	-0.89% (-0.95 to -0.82)
Vanuatu	2 (1 to 2)	2 (2 to 3)	44.79% (24.08 to 67.95)	5.1 (3.6 to 6.6)	4.4 (3.1 to 5.8)	-0.65% (-0.73 to -0.57)
Venezuela (Bolivarian Republic of)	884 (612 to 1127)	960 (681 to 1191)	8.65% (-6 to 29.44)	25.3 (17.5 to 32.2)	27.9 (19.8 to 34.6)	0.53% (0.22 to 0.84)
Viet Nam	395 (269 to 515)	232 (159 to 301)	-41.21% (-48.37 to -32.03)	3.1 (2.1 to 4)	2.3 (1.6 to 2.9)	-1.32% (-1.65 to -0.98)
Yemen	166 (119 to 206)	224 (160 to 282)	34.88% (16.63 to 53.19)	4.8 (3.5 to 6)	3.5 (2.5 to 4.5)	-1.54% (-1.75 to -1.34)
Zambia	392 (276 to 495)	594 (399 to 776)	51.61% (31.12 to 74.15)	20.7 (14.6 to 26.2)	15.3 (10.3 to 20)	-0.9% (-1 to -0.8)
Zimbabwe	205 (145 to 259)	220 (157 to 280)	7.24% (-7.3 to 23.36)	8.5 (6 to 10.7)	7.4 (5.3 to 9.4)	-0.58% (-0.65 to -0.5)

ASIR – age-standardized incidence rate (per 100,000 population), CI – confidence interval, EAPC – estimated annual percentage change, MSMI – maternal sepsis and other maternal infections, UI – uncertain interval.

Table S8. Incidence of MSMI in 131 low- and middle-income countries and territories with EAPC in age group of 15-19 years from 1990 to 2019.

Location	Incident cases in 1990 (95% UI)	Incident cases in 2019 (95% UI)	Relative change (%, 95 CI)	ASIR in 1990 (95% UI)	ASIR in 2019 (95% UI)	EAPC (%, 95 CI)
Afghanistan	26191 (17630 to 33895)	51026 (33765 to 66996)	94.82% (71.2 to 126)	3754.7 (2527.5 to 4859.1)	2420.5 (1601.7 to 3178)	-1.69% (-1.88 to -1.49)
Albania	1666 (1259 to 1939)	828 (615 to 973)	-50.28% (-56.77 to -44.53)	1028.4 (777.6 to 1197.2)	898.5 (667 to 1055.1)	-0.88% (-1.24 to -0.53)
Algeria	10679 (7357 to 13936)	4179 (2863 to 5343)	-60.86% (-65.74 to -55.08)	778.4 (536.2 to 1015.7)	280.7 (192.3 to 358.8)	-3.07% (-4.14 to -1.99)
American Samoa	28 (19 to 39)	24 (17 to 34)	-14.07% (-24.07 to -1.72)	1204.2 (820.1 to 1646.4)	827.7 (563.9 to 1140)	-1.31% (-1.44 to -1.18)
Angola	22264 (14895 to 30340)	43487 (29605 to 59513)	95.32% (74.46 to 126.68)	4270.9 (2857.4 to 5820.1)	2776.9 (1890.5 to 3800.4)	-1.34% (-1.45 to -1.24)
Armenia	3682 (2639 to 4547)	729 (526 to 904)	-80.21% (-82.83 to -77.59)	2682.2 (1922.6 to 3312.5)	937.4 (676.2 to 1163)	-3.9% (-4.67 to -3.12)

Location	Incident cases in 1990 (95% UI)	Incident cases in 2019 (95% UI)	Relative change (%, 95 CI)	ASIR in 1990 (95% UI)	ASIR in 2019 (95% UI)	EAPC (%, 95 CI)
Azerbaijan	6317 (4359 to 7841)	6125 (4340 to 7591)	-3.04% (-15.12 to 11.54)	1884.2 (1300.2 to 2338.9)	1944.7 (1377.9 to 2410.1)	0.43% (0.19 to 0.66)
Bangladesh	187132 (125616 to 256415)	118840 (81070 to 162410)	-36.49% (-45.13 to -27.02)	3162.3 (2122.8 to 4333.1)	1500.2 (1023.4 to 2050.3)	-2.69% (-2.75 to -2.62)
Belarus	5246 (3745 to 6207)	1209 (881 to 1434)	-76.96% (-79.52 to -73.8)	1438.1 (1026.7 to 1701.5)	563 (410.1 to 667.6)	-3.12% (-3.32 to -2.92)
Belize	320 (219 to 432)	312 (211 to 426)	-2.62% (-14.34 to 11.66)	3085.9 (2109.6 to 4163.1)	1383.1 (938.4 to 1889.2)	-2.88% (-2.94 to -2.81)
Benin	5075 (3394 to 7044)	11318 (7544 to 15503)	123.03% (93.61 to 159.88)	2205.7 (1475.2 to 3061.4)	1652.3 (1101.3 to 2263.3)	-0.73% (-0.83 to -0.63)
Bhutan	842 (583 to 1149)	274 (189 to 365)	-67.43% (-71.49 to -62.51)	2520.7 (1743.7 to 3437.8)	793.6 (547.2 to 1055.2)	-4.09% (-4.28 to -3.91)
Bolivia (Plurinational State of)	9207 (6233 to 12408)	9279 (6172 to 12556)	0.79% (-12.94 to 14.76)	2776.3 (1879.5 to 3741.7)	1754 (1166.7 to 2373.3)	-1.69% (-1.83 to -1.55)

Location	Incident cases in 1990 (95% UI)	Incident cases in 2019 (95% UI)	Relative change (%, 95 CI)	ASIR in 1990 (95% UI)	ASIR in 2019 (95% UI)	EAPC (%, 95 CI)
Bosnia and Herzegovina	2384 (1805 to 2728)	361 (274 to 414)	-84.85% (-86.5 to -82.92)	1253.2 (948.6 to 1434.1)	395.1 (299.3 to 452.6)	-4.2% (-4.69 to -3.71)
Botswana	1793 (1261 to 2436)	1364 (958 to 1846)	-23.93% (-33.62 to -14.35)	2293.8 (1612.9 to 3117.1)	1285.6 (903 to 1740)	-1.73% (-1.82 to -1.64)
Brazil	119678 (86127 to 158446)	122004 (90781 to 141944)	1.94% (-15.66 to 20.11)	1555.2 (1119.2 to 2059)	1504 (1119.1 to 1749.8)	0.51% (0.24 to 0.78)
Bulgaria	7262 (5404 to 8470)	2023 (1499 to 2368)	-72.15% (-76.05 to -68.28)	2372.2 (1765.1 to 2766.7)	1321.2 (979.4 to 1546.7)	-1.54% (-1.91 to -1.17)
Burkina Faso	12678 (8348 to 17554)	20566 (13875 to 28414)	62.22% (40.18 to 87.32)	2636.2 (1735.8 to 3650.1)	1711.2 (1154.5 to 2364.2)	-1.57% (-1.61 to -1.52)
Burundi	4841 (3240 to 6574)	7727 (5207 to 10477)	59.63% (38.69 to 81.61)	1776.3 (1189.1 to 2412.4)	1180.7 (795.6 to 1600.8)	-1.21% (-1.28 to -1.14)
Cabo Verde	263 (176 to 361)	105 (71 to 145)	-59.88% (-65.86 to -53.34)	1414.6 (946.1 to 1943.2)	424.9 (286.7 to 584.7)	-4.17% (-4.34 to -4.01)

Location	Incident cases in 1990 (95% UI)	Incident cases in 2019 (95% UI)	Relative change (%, 95 CI)	ASIR in 1990 (95% UI)	ASIR in 2019 (95% UI)	EAPC (%, 95 CI)
Cambodia	11464 (7788 to 15735)	7081 (4790 to 9769)	-38.23% (-46.52 to -27.86)	2222.7 (1510.1 to 3050.8)	979.7 (662.7 to 1351.5)	-2.88% (-3.17 to -2.59)
Cameroon	13822 (9156 to 19218)	21513 (14532 to 29545)	55.65% (32.86 to 80.29)	2562.3 (1697.4 to 3562.6)	1336.3 (902.6 to 1835.1)	-1.82% (-2.09 to -1.55)
Central African Republic	5371 (3700 to 7347)	8828 (6120 to 12169)	64.38% (45.9 to 88.48)	3853.7 (2654.8 to 5271.8)	2975.5 (2062.7 to 4101.7)	-0.8% (-0.83 to -0.77)
Chad	10394 (7107 to 14282)	26179 (17759 to 36000)	151.87% (118.81 to 196.08)	3364.3 (2300.4 to 4622.7)	2891.4 (1961.4 to 3976.1)	-0.58% (-0.63 to -0.53)
China	195986 (136346 to 268719)	48816 (33699 to 66814)	-75.09% (-75.95 to -74.26)	317.5 (220.9 to 435.4)	139.3 (96.2 to 190.7)	-0.88% (-1.95 to 0.2)
Colombia	41796 (29113 to 57038)	35631 (24207 to 47925)	-14.75% (-25.73 to -2)	2413.6 (1681.2 to 3293.7)	1828 (1241.9 to 2458.7)	-1.18% (-1.37 to -0.99)
Comoros	765 (531 to 1042)	325 (223 to 436)	-57.47% (-63.33 to -49.85)	2967.2 (2059.6 to 4041.4)	910.8 (623.8 to 1220.8)	-3.97% (-4.15 to -3.78)

Location	Incident cases in 1990 (95% UI)	Incident cases in 2019 (95% UI)	Relative change (%, 95 CI)	ASIR in 1990 (95% UI)	ASIR in 2019 (95% UI)	EAPC (%, 95 CI)
Congo	4662 (3190 to 6393)	4404 (3022 to 6176)	-5.55% (-18.23 to 8.78)	3367.8 (2304 to 4617.7)	1699.7 (1166.5 to 2383.8)	-1.92% (-2.2 to -1.64)
Costa Rica	2987 (2086 to 4129)	2260 (1536 to 3100)	-24.35% (-34.23 to - 13.28)	2041.9 (1425.9 to 2822.3)	1249.7 (849.3 to 1714.6)	-1.78% (-1.91 to -1.66)
Côte d'Ivoire	16289 (10840 to 22853)	21045 (14486 to 28756)	29.2% (8.73 to 51.37)	2656 (1767.5 to 3726.1)	1651.3 (1136.6 to 2256.3)	-1.52% (-1.58 to -1.47)
Cuba	9618 (6587 to 13181)	3773 (2582 to 5037)	-60.78% (-66.22 to - 54.74)	1733.3 (1187 to 2375.4)	1141.2 (781 to 1523.5)	-0.89% (-1.28 to -0.49)
Democratic People's Republic of Korea	4690 (3190 to 6445)	725 (494 to 997)	-84.55% (-86.22 to - 82.42)	460.8 (313.5 to 633.2)	81.8 (55.7 to 112.6)	-6.13% (-6.28 to -5.98)
Democratic Republic of the Congo	68890 (48114 to 92604)	84101 (57433 to 114966)	22.08% (5.64 to 39.29)	3525.8 (2462.4 to 4739.4)	1765.9 (1206 to 2414)	-2.14% (-2.42 to -1.85)
Djibouti	725 (494 to 1007)	643 (439 to 875)	-11.34% (-21.91 to 3.3)	2979.1 (2030.9 to 4139.6)	1410.3 (962.9 to 1919.6)	-2.53% (-2.8 to -2.25)

Location	Incident cases in 1990 (95% UI)	Incident cases in 2019 (95% UI)	Relative change (%, 95 CI)	ASIR in 1990 (95% UI)	ASIR in 2019 (95% UI)	EAPC (%, 95 CI)
Dominica	96 (65 to 131)	29 (19 to 39)	-69.92% (-73.97 to -65.38)	2549 (1710.3 to 3471.3)	1042 (693.8 to 1406.8)	-3.4% (-3.54 to -3.27)
Dominican Republic	13561 (9411 to 18885)	12748 (8929 to 16929)	-5.99% (-17.71 to 9.29)	3243.4 (2250.8 to 4516.8)	2644.7 (1852.4 to 3512.2)	-1.11% (-1.28 to -0.93)
Ecuador	13929 (9412 to 18646)	18333 (12460 to 24260)	31.62% (13.31 to 51.02)	2531 (1710.2 to 3388.1)	2247.8 (1527.6 to 2974.4)	-0.33% (-0.38 to -0.27)
Egypt	72459 (49920 to 94712)	54613 (38004 to 71437)	-24.63% (-34.05 to -14.73)	2636.7 (1816.5 to 3446.4)	1230 (855.9 to 1608.9)	-2.13% (-2.31 to -1.95)
El Salvador	8555 (5925 to 11643)	4612 (3201 to 6243)	-46.09% (-52.75 to -38.08)	2821.6 (1954.2 to 3840.2)	1586.1 (1100.9 to 2147)	-2.36% (-2.52 to -2.19)
Equatorial Guinea	1157 (792 to 1602)	1694 (1157 to 2321)	46.44% (27.4 to 69.43)	5332.9 (3652.3 to 7383.6)	2257.9 (1541.5 to 3092.2)	-2.97% (-3.2 to -2.74)
Eritrea	4213 (2881 to 5885)	3995 (2733 to 5526)	-5.18% (-17.45 to 9.03)	2667.8 (1824.6 to 3727.1)	1070.4 (732.3 to 1480.7)	-3.08% (-3.28 to -2.88)

Location	Incident cases in 1990 (95% UI)	Incident cases in 2019 (95% UI)	Relative change (%, 95 CI)	ASIR in 1990 (95% UI)	ASIR in 2019 (95% UI)	EAPC (%, 95 CI)
Eswatini	1584 (1089 to 2177)	1038 (724 to 1398)	-34.51% (-42.52 to -24.8)	3344.9 (2299.1 to 4596)	1725.5 (1203.8 to 2325.1)	-2.09% (-2.16 to -2.03)
Ethiopia	92281 (63647 to 125288)	97353 (65849 to 134148)	5.5% (-1.98 to 13.62)	3583.9 (2471.8 to 4865.8)	1578.3 (1067.6 to 2174.8)	-2.92% (-3.02 to -2.81)
Fiji	428 (291 to 591)	238 (167 to 327)	-44.55% (-51.46 to -36.8)	1129.9 (766.5 to 1558.8)	637.5 (448.9 to 878.2)	-2.06% (-2.16 to -1.95)
Gabon	2003 (1364 to 2747)	1202 (824 to 1643)	-39.98% (-47.63 to -31.18)	3879.1 (2641.6 to 5321.6)	1295.2 (887.8 to 1770.5)	-3.66% (-3.77 to -3.55)
Gambia	1217 (817 to 1664)	1287 (897 to 1761)	5.76% (-8.65 to 21.65)	2364.1 (1587.5 to 3231.9)	970 (675.8 to 1327)	-3.06% (-3.24 to -2.88)
Georgia	3934 (2800 to 4798)	1311 (926 to 1618)	-66.67% (-71.12 to -61.98)	1909.6 (1359.3 to 2328.9)	1429.9 (1009.7 to 1764.7)	-0.32% (-0.95 to 0.32)
Ghana	12938 (8828 to 17716)	12205 (8139 to 16525)	-5.67% (-18.4 to 10.69)	1687.2 (1151.2 to 2310.3)	745.8 (497.4 to 1009.8)	-2.56% (-2.76 to -2.37)

Location	Incident cases in 1990 (95% UI)	Incident cases in 2019 (95% UI)	Relative change (%, 95 CI)	ASIR in 1990 (95% UI)	ASIR in 2019 (95% UI)	EAPC (%, 95 CI)
Global	3988690 (2833027 to 5210443)	2887793 (2034645 to 3779060)	-27.6% (-28.77 to -26.2)	1561 (1108.7 to 2039.1)	957 (674.3 to 1252.3)	-1.72% (-1.79 to -1.65)
Grenada	113 (77 to 155)	45 (31 to 62)	-60.57% (-65.71 to -54.6)	2630.9 (1792 to 3594.5)	1096.4 (750.3 to 1513.3)	-2.69% (-3.1 to -2.28)
Guatemala	14867 (10097 to 20428)	15490 (10448 to 21380)	4.19% (-11.4 to 22.08)	3595.7 (2442.1 to 4940.6)	1658.5 (1118.6 to 2289.2)	-2.78% (-2.91 to -2.65)
Guinea	9033 (6038 to 12514)	14080 (9368 to 19077)	55.88% (31.1 to 86.13)	3139.6 (2098.6 to 4349.7)	2058.8 (1369.9 to 2789.5)	-1.5% (-1.62 to -1.38)
Guinea-Bissau	1275 (883 to 1765)	1277 (853 to 1751)	0.17% (-13 to 16.11)	2344.4 (1623.9 to 3244.5)	1222.9 (816.8 to 1675.9)	-2.27% (-2.39 to -2.15)
Guyana	1276 (880 to 1726)	520 (357 to 692)	-59.25% (-64.25 to -53.28)	2883.7 (1989.6 to 3901.1)	1448.1 (994.5 to 1928)	-2.43% (-2.46 to -2.4)
Haiti	6959 (4651 to 9457)	8467 (5796 to 11498)	21.66% (7.3 to 37.67)	2129.2 (1422.9 to 2893.4)	1374.5 (940.9 to 1866.6)	-1.56% (-1.62 to -1.5)

Location	Incident cases in 1990 (95% UI)	Incident cases in 2019 (95% UI)	Relative change (%, 95 CI)	ASIR in 1990 (95% UI)	ASIR in 2019 (95% UI)	EAPC (%, 95 CI)
Honduras	8696 (5855 to 11952)	10903 (7485 to 14861)	25.38% (7.56 to 45.21)	3417.7 (2301.1 to 4697.4)	2048.3 (1406.1 to 2791.9)	-2.14% (-2.29 to -1.99)
India	713942 (497339 to 948962)	222096 (155639 to 298200)	-68.89% (-69.96 to -67.8)	1781.6 (1241.1 to 2368.1)	345.7 (242.3 to 464.2)	-5.51% (-6.09 to -4.92)
Indonesia	178984 (122827 to 244418)	61836 (42116 to 84782)	-65.45% (-67.03 to -63.89)	1807.8 (1240.6 to 2468.7)	550.7 (375.1 to 755.1)	-4.22% (-4.33 to -4.12)
Iran (Islamic Republic of)	65869 (45533 to 83433)	16783 (11476 to 21856)	-74.52% (-75.8 to -73.13)	2201.5 (1521.8 to 2788.5)	630.1 (430.9 to 820.6)	-3.03% (-3.47 to -2.58)
Iraq	28849 (20337 to 38089)	29573 (19735 to 39071)	2.51% (-10.86 to 16.03)	3025.9 (2133.1 to 3995.1)	1376.8 (918.8 to 1819)	-2.76% (-3 to -2.51)
Jamaica	3207 (2188 to 4411)	1045 (718 to 1397)	-67.41% (-71.63 to -61.5)	2476 (1689.3 to 3406.3)	871.1 (598.9 to 1164.7)	-3.91% (-4.03 to -3.79)
Jordan	2760 (1873 to 3787)	8455 (6246 to 10614)	206.32% (155.89 to 280.93)	1254.1 (851.1 to 1720.6)	1498 (1106.6 to 1880.6)	1.2% (0.91 to 1.5)

Location	Incident cases in 1990 (95% UI)	Incident cases in 2019 (95% UI)	Relative change (%, 95 CI)	ASIR in 1990 (95% UI)	ASIR in 2019 (95% UI)	EAPC (%, 95 CI)
Kazakhstan	11083 (7633 to 13691)	5020 (3620 to 6216)	-54.7% (-59.42 to -48.23)	1567 (1079.3 to 1935.8)	856.7 (617.8 to 1060.8)	-1.86% (-2.34 to -1.37)
Kenya	45111 (31176 to 60958)	40897 (28299 to 55049)	-9.34% (-11.63 to -7.06)	3388.5 (2341.8 to 4578.9)	1409.1 (975 to 1896.7)	-2.9% (-2.94 to -2.85)
Kiribati	39 (27 to 54)	35 (24 to 48)	-11.41% (-22.32 to 0.44)	1125.2 (781.8 to 1537.1)	629.1 (436.3 to 864.7)	-2.09% (-2.24 to -1.94)
Kyrgyzstan	3449 (2417 to 4292)	2670 (1887 to 3305)	-22.6% (-31.17 to -13.57)	1617.8 (1133.6 to 2013.1)	1031.1 (728.7 to 1276.3)	-0.94% (-1.68 to -0.19)
Lao People's Democratic Republic	5557 (3677 to 7824)	5391 (3574 to 7425)	-2.98% (-16.34 to 10.66)	2525.3 (1671.2 to 3555.7)	1538.7 (1020.1 to 2119.2)	-1.99% (-2.18 to -1.8)
Lebanon	5341 (3709 to 6993)	2504 (1661 to 3301)	-53.12% (-59.23 to -45.81)	3531.8 (2452.2 to 4624)	1558.5 (1033.9 to 2054.3)	-2.89% (-3.11 to -2.68)
Lesotho	2798 (1900 to 3812)	1667 (1169 to 2268)	-40.45% (-48.25 to -30.95)	2785.3 (1891.5 to 3794.2)	1526.9 (1071.4 to 2078.5)	-1.6% (-1.77 to -1.42)

Location	Incident cases in 1990 (95% UI)	Incident cases in 2019 (95% UI)	Relative change (%, 95 CI)	ASIR in 1990 (95% UI)	ASIR in 2019 (95% UI)	EAPC (%, 95 CI)
Liberia	2947<br>(1995 to 4036)	3475<br>(2321 to 4790)	17.93%<br>(0.67 to 38.28)	2810.9<br>(1902.6 to 3849.6)	1306.8<br>(872.6 to 1801.3)	-2.54%<br>(-2.74 to -2.34)
Libya	4338<br>(3032 to 5701)	642<br>(448 to 826)	-85.21%<br>(-86.96 to -82.89)	1867.7<br>(1305.6 to 2454.7)	223.7<br>(156.1 to 288.2)	-6.67%<br>(-7.01 to -6.34)
Madagascar	21431<br>(14622 to 29669)	36060<br>(24383 to 50210)	68.26%<br>(45.75 to 93.12)	3344.1<br>(2281.6 to 4629.4)	2463.1<br>(1665.5 to 3429.5)	-1.16%<br>(-1.32 to -0.99)
Malawi	20155<br>(13411 to 27675)	24511<br>(16722 to 33592)	21.62%<br>(5.33 to 40.15)	3944.6<br>(2624.8 to 5416.5)	2097.3<br>(1430.8 to 2874.3)	-2.22%<br>(-2.55 to -1.89)
Malaysia	5653<br>(3832 to 7826)	3050<br>(2048 to 4246)	-46.05%<br>(-53.24 to -38.02)	644.5<br>(436.9 to 892.3)	229.6<br>(154.2 to 319.7)	-3.45%<br>(-3.95 to -2.96)
Maldives	168<br>(113 to 231)	52<br>(35 to 73)	-68.99%<br>(-72.91 to -64.68)	1438<br>(965 to 1981.3)	373.2<br>(253.8 to 523.1)	-4.25%<br>(-4.57 to -3.94)
Mali	13299<br>(9031 to 17875)	28347<br>(18829 to 39014)	113.16%<br>(83.19 to 147.65)	3225<br>(2190.1 to 4334.8)	2306.7<br>(1532.2 to 3174.6)	-1.18%<br>(-1.23 to -1.13)

Location	Incident cases in 1990 (95% UI)	Incident cases in 2019 (95% UI)	Relative change (%, 95 CI)	ASIR in 1990 (95% UI)	ASIR in 2019 (95% UI)	EAPC (%, 95 CI)
Marshall Islands	57 (40 to 78)	43 (29 to 58)	-25.19% (-34.23 to -13.22)	2400.9 (1668 to 3289.6)	1576.3 (1080.5 to 2157.7)	-1.31% (-1.38 to -1.24)
Mauritania	2433 (1649 to 3341)	2221 (1502 to 3053)	-8.7% (-22.86 to 5.48)	2292.7 (1553.7 to 3148.3)	963.2 (651.1 to 1323.6)	-2.79% (-2.96 to -2.62)
Mexico	101163 (69020 to 135202)	79558 (55055 to 102741)	-21.36% (-28.1 to -14.26)	2007.4 (1369.6 to 2682.9)	1462.7 (1012.2 to 1889)	-0.84% (-1.02 to -0.67)
Micronesia (Federated States of)	75 (51 to 104)	40 (27 to 55)	-46.18% (-53.07 to -39.3)	1318.3 (902.1 to 1819.3)	748.3 (505.7 to 1027.1)	-1.87% (-1.9 to -1.83)
Mongolia	2254 (1586 to 2756)	1535 (1090 to 1897)	-31.92% (-40.39 to -21.61)	1952.6 (1374.1 to 2387.4)	1431.4 (1016.6 to 1769.8)	-1.06% (-1.66 to -0.47)
Montenegro	230 (169 to 270)	66 (49 to 79)	-71.1% (-75.12 to -67.37)	891.2 (655.2 to 1047.1)	339.2 (248.7 to 403.5)	-3.96% (-4.17 to -3.74)
Morocco	21957 (15264 to 28900)	13508 (9315 to 17621)	-38.48% (-46.91 to -29.24)	1622.7 (1128.1 to 2135.8)	885 (610.3 to 1154.5)	-1.9% (-2.18 to -1.61)

Location	Incident cases in 1990 (95% UI)	Incident cases in 2019 (95% UI)	Relative change (%, 95 CI)	ASIR in 1990 (95% UI)	ASIR in 2019 (95% UI)	EAPC (%, 95 CI)
Mozambique	28882 (19104 to 39866)	50275 (33910 to 69340)	74.07% (52.86 to 100.45)	4081.3 (2699.7 to 5633.5)	2952.5 (1991.4 to 4072)	-1.04% (-1.2 to -0.88)
Myanmar	34339 (22839 to 47391)	21820 (14578 to 30644)	-36.46% (-44.54 to -25.8)	1610.8 (1071.4 to 2223.2)	879.5 (587.6 to 1235.2)	-2.04% (-2.06 to -2.02)
Namibia	1626 (1118 to 2184)	1612 (1128 to 2211)	-0.84% (-14.12 to 14.34)	1990.9 (1368.9 to 2674.9)	1328.8 (930.1 to 1822.6)	-0.97% (-1.13 to -0.81)
Nepal	35687 (24449 to 49190)	31689 (21387 to 43598)	-11.2% (-23.31 to 2.06)	3671.3 (2515.3 to 5060.6)	1894.9 (1278.9 to 2607.1)	-2.48% (-2.64 to -2.31)
Nicaragua	7646 (5332 to 10569)	6394 (4294 to 8716)	-16.38% (-27.01 to -4.78)	3514.7 (2450.9 to 4858)	2077.2 (1395.1 to 2831.8)	-1.89% (-2.1 to -1.69)
Niger	15383 (10424 to 21079)	38006 (25717 to 52198)	147.06% (111.89 to 194.51)	3743.9 (2536.9 to 5130)	2925.8 (1979.8 to 4018.5)	-0.82% (-0.85 to -0.78)
Nigeria	123093 (85458 to 164224)	173785 (120815 to 229834)	41.18% (35.75 to 47.07)	2473.3 (1717.1 to 3299.8)	1378.8 (958.6 to 1823.5)	-2.35% (-2.69 to -2.01)

Location	Incident cases in 1990 (95% UI)	Incident cases in 2019 (95% UI)	Relative change (%, 95 CI)	ASIR in 1990 (95% UI)	ASIR in 2019 (95% UI)	EAPC (%, 95 CI)
North Macedonia	1235 (921 to 1443)	266 (189 to 322)	-78.44% (-81.64 to -75.51)	1477.4 (1101.5 to 1726)	451.7 (321.2 to 545.4)	-4.53% (-4.91 to -4.14)
Pakistan	235773 (164263 to 315001)	279160 (194352 to 374558)	18.4% (9.61 to 28.21)	4180.6 (2912.6 to 5585.4)	2330.7 (1622.7 to 3127.2)	-2.14% (-2.21 to -2.06)
Papua New Guinea	3504 (2445 to 4773)	5929 (4055 to 8082)	69.2% (47.64 to 92.83)	1696.2 (1183.5 to 2310.3)	1317.4 (901.1 to 1795.8)	-0.87% (-0.93 to -0.81)
Paraguay	5208 (3580 to 7023)	4355 (2999 to 5890)	-16.37% (-27.17 to -4.28)	2604 (1790.2 to 3511.5)	1374.9 (946.7 to 1859.6)	-2.08% (-2.2 to -1.96)
Peru	28178 (19005 to 37563)	19126 (12715 to 26881)	-32.12% (-41.13 to -22.11)	2394.9 (1615.3 to 3192.6)	1411.7 (938.5 to 1984.1)	-1.99% (-2.05 to -1.93)
Philippines	51810 (35614 to 71163)	73748 (50116 to 102278)	42.34% (38.42 to 46.75)	1517.3 (1043 to 2084.1)	1407 (956.1 to 1951.3)	-0.13% (-0.26 to 0.01)
Republic of Moldova	3223 (2333 to 3791)	734 (540 to 857)	-77.24% (-79.85 to -74.31)	1910.9 (1383.2 to 2247.6)	771.6 (568 to 901.8)	-3.43% (-3.71 to -3.15)

Location	Incident cases in 1990 (95% UI)	Incident cases in 2019 (95% UI)	Relative change (%, 95 CI)	ASIR in 1990 (95% UI)	ASIR in 2019 (95% UI)	EAPC (%, 95 CI)
Russian Federation	87416 (62663 to 105411)	22775 (16282 to 27520)	-73.95% (-74.44 to -73.48)	1691.5 (1212.5 to 2039.7)	632 (451.9 to 763.7)	-2.83% (-3.31 to -2.35)
Rwanda	5231 (3526 to 7091)	3757 (2602 to 5183)	-28.18% (-37.26 to -17.92)	1381.1 (930.9 to 1872.2)	528.6 (366.2 to 729.3)	-3.88% (-4.14 to -3.62)
Saint Lucia	198 (133 to 268)	54 (37 to 74)	-72.58% (-76.66 to -68.73)	2569.4 (1725.9 to 3485.3)	820.8 (560.2 to 1126.5)	-3.87% (-4.14 to -3.6)
Saint Vincent and the Grenadines	153 (104 to 207)	66 (45 to 90)	-57.07% (-62.54 to -50.91)	2474.4 (1686.9 to 3347.4)	1480.5 (1014.8 to 2031.5)	-1.67% (-1.76 to -1.58)
Samoa	38 (26 to 51)	20 (14 to 28)	-45.64% (-52.85 to -37.78)	400.5 (273.9 to 548.5)	178.6 (122.6 to 244)	-2.87% (-2.96 to -2.77)
Senegal	8451 (5613 to 11609)	7374 (4828 to 10278)	-12.75% (-24.28 to -0.15)	2046.8 (1359.4 to 2811.4)	917.4 (600.6 to 1278.7)	-2.52% (-2.65 to -2.38)
Serbia	3712 (2521 to 5015)	1057 (707 to 1448)	-71.54% (-74.7 to -67.59)	1072.9 (728.5 to 1449.5)	417.3 (279.2 to 571.6)	-3.47% (-3.66 to -3.29)

Location	Incident cases in 1990 (95% UI)	Incident cases in 2019 (95% UI)	Relative change (%, 95 CI)	ASIR in 1990 (95% UI)	ASIR in 2019 (95% UI)	EAPC (%, 95 CI)
Sierra Leone	4724 (3212 to 6673)	7703 (5172 to 10480)	63.05% (39.66 to 89.03)	2614.8 (1777.7 to 3693.4)	1625.5 (1091.6 to 2211.8)	-1.65% (-1.77 to -1.52)
Solomon Islands	449 (309 to 628)	505 (351 to 708)	12.48% (-1.59 to 28.39)	2365.7 (1628.8 to 3310.3)	1552.4 (1078.6 to 2177.1)	-1.52% (-1.56 to -1.47)
Somalia	12878 (8679 to 17797)	27691 (18618 to 37738)	115.03% (89.2 to 146.01)	3503.9 (2361.4 to 4842.1)	2509.7 (1687.4 to 3420.3)	-1.05% (-1.11 to -0.98)
South Africa	42013 (29325 to 56475)	27037 (18694 to 36181)	-35.64% (-38.95 to -32.26)	2023.2 (1412.2 to 2719.7)	1210.5 (837 to 1619.9)	-1.66% (-1.69 to -1.62)
South Sudan	10010 (6854 to 13794)	15142 (10338 to 20866)	51.27% (30.39 to 72.88)	3185 (2180.8 to 4389.1)	2805.6 (1915.6 to 3866.2)	-0.41% (-0.44 to -0.39)
Sri Lanka	6236 (4276 to 8926)	2782 (1863 to 3888)	-55.39% (-61.52 to -48.37)	738 (506 to 1056.3)	321.4 (215.3 to 449.2)	-2.77% (-2.98 to -2.56)
Sudan	62602 (44598 to 80066)	48716 (33950 to 62300)	-22.18% (-31.17 to -10.22)	5859.3 (4174.2 to 7493.7)	2262.6 (1576.8 to 2893.5)	-3.3% (-3.53 to -3.07)

Location	Incident cases in 1990 (95% UI)	Incident cases in 2019 (95% UI)	Relative change (%, 95 CI)	ASIR in 1990 (95% UI)	ASIR in 2019 (95% UI)	EAPC (%, 95 CI)
Suriname	372 (247 to 512)	301 (207 to 418)	-19.01% (-29.48 to -9.27)	1937.3 (1288 to 2668.6)	1319 (906.7 to 1830.3)	-1.31% (-1.5 to -1.11)
Syrian Arab Republic	21509 (14870 to 28205)	8462 (5934 to 10958)	-60.66% (-65.91 to -55.22)	3044.5 (2104.8 to 3992.2)	924.4 (648.3 to 1197.1)	-3.77% (-4.25 to -3.29)
Tajikistan	7181 (5063 to 8757)	6420 (4554 to 7911)	-10.6% (-22.17 to 1.44)	2602.5 (1834.7 to 3173.6)	1526.1 (1082.4 to 1880.5)	-2.13% (-2.39 to -1.87)
Thailand	31962 (21785 to 45132)	16396 (11482 to 22344)	-48.7% (-55.41 to -41.35)	1084.6 (739.3 to 1531.6)	777.2 (544.3 to 1059.2)	-0.96% (-1.1 to -0.82)
Timor-Leste	818 (554 to 1131)	593 (403 to 824)	-27.47% (-36.95 to -16.94)	2360.8 (1599.6 to 3263.8)	757.8 (515 to 1052.8)	-4.27% (-4.42 to -4.12)
Togo	3502 (2357 to 4784)	3286 (2205 to 4496)	-6.15% (-19.72 to 10.04)	1796.3 (1209.3 to 2454.1)	842.7 (565.4 to 1153)	-2.33% (-2.56 to -2.11)
Tonga	31 (21 to 43)	19 (13 to 26)	-39.53% (-47.4 to -30.49)	563.2 (386 to 773.3)	367.6 (253.5 to 503.2)	-1.31% (-1.4 to -1.22)

Location	Incident cases in 1990 (95% UI)	Incident cases in 2019 (95% UI)	Relative change (%, 95 CI)	ASIR in 1990 (95% UI)	ASIR in 2019 (95% UI)	EAPC (%, 95 CI)
Tunisia	5574 (3851 to 7267)	679 (471 to 881)	-87.81% (-89.36 to -86.02)	1280.6 (884.9 to 1669.8)	173.3 (120.3 to 224.8)	-7.44% (-7.69 to -7.18)
Turkey	84043 (62286 to 96446)	24147 (18139 to 27811)	-71.27% (-74.73 to -67.92)	2676.7 (1983.8 to 3071.7)	760.6 (571.4 to 876)	-4.03% (-4.23 to -3.83)
Turkmenistan	1769 (1256 to 2165)	1300 (918 to 1594)	-26.48% (-35.61 to -17.04)	948.1 (673.3 to 1160.8)	686.6 (484.9 to 841.4)	-0.82% (-1.26 to -0.38)
Tuvalu	3 (2 to 4)	3 (2 to 4)	-13.69% (-24.58 to -3.08)	819.5 (559.8 to 1116.2)	518.3 (354.6 to 701)	-1.5% (-1.54 to -1.46)
Uganda	46218 (31512 to 63041)	54133 (37107 to 75843)	17.12% (2.27 to 33.25)	4760.1 (3245.5 to 6492.7)	2237.4 (1533.7 to 3134.7)	-2.88% (-3.07 to -2.68)
Ukraine	34462 (24824 to 42604)	7447 (5245 to 9183)	-78.39% (-81.14 to -75.54)	1909.8 (1375.7 to 2361)	747.2 (526.3 to 921.4)	-3.21% (-3.73 to -2.69)
United Republic of Tanzania	48709 (32993 to 67322)	80049 (53745 to 110112)	64.34% (42.83 to 88.09)	3294.5 (2231.5 to 4553.4)	2545.3 (1708.9 to 3501.2)	-0.63% (-0.73 to -0.53)

Location	Incident cases in 1990 (95% UI)	Incident cases in 2019 (95% UI)	Relative change (%, 95 CI)	ASIR in 1990 (95% UI)	ASIR in 2019 (95% UI)	EAPC (%, 95 CI)
Uzbekistan	20072 (14399 to 24868)	15340 (10940 to 18896)	-23.58% (-33.14 to -12.91)	1899.2 (1362.3 to 2352.9)	1101.2 (785.3 to 1356.4)	-1.96% (-2.58 to -1.34)
Vanuatu	134 (93 to 185)	157 (107 to 218)	17.47% (4.68 to 34.8)	1771.8 (1223.5 to 2443.3)	1112.1 (757.8 to 1542)	-1.74% (-1.82 to -1.65)
Venezuela (Bolivarian Republic of)	22673 (15586 to 31905)	26453 (18016 to 36386)	16.67% (-0.82 to 36.41)	2298.2 (1579.9 to 3233.9)	2365.2 (1610.9 to 3253.4)	0.61% (0.3 to 0.92)
Viet Nam	38345 (25995 to 53688)	16901 (11316 to 23757)	-55.92% (-61.61 to -48.97)	1063 (720.6 to 1488.3)	515.3 (345 to 724.4)	-2.41% (-2.52 to -2.29)
Yemen	32591 (22545 to 41735)	34260 (23703 to 44463)	5.12% (-9.06 to 21.46)	5157 (3567.5 to 6604)	2082.4 (1440.7 to 2702.6)	-3.22% (-3.47 to -2.97)
Zambia	18773 (12963 to 25628)	21352 (14717 to 29729)	13.73% (-1.57 to 32.31)	3844.1 (2654.4 to 5247.6)	2029.8 (1399.1 to 2826.1)	-2.14% (-2.39 to -1.89)
Zimbabwe	18004 (12525 to 24696)	18006 (12434 to 24738)	0.01% (-14.01 to 14.78)	2937.1 (2043.3 to 4028.7)	2141.8 (1479.1 to 2942.6)	-0.75% (-0.88 to -0.61)

ASIR – age-standardized incidence rate (per 100,000 population), CI – confidence interval, EAPC – estimated annual percentage change, MSMI – maternal sepsis and other maternal infections, UI – uncertain interval.

Table S9. Incidence of MSMI in 131 low- and middle-income countries and territories with EAPC in age group of 20-24 years from 1990 and 2019.

Location	Incident cases in 1990 (95% UI)	Incident cases in 2019 (95% UI)	Relative change (%, 95 CI)	ASIR in 1990 (95% UI)	ASIR in 2019 (95% UI)	EAPC (%, 95 CI)
Afghanistan	39530 (24937 to 53176)	108188 (67274 to 145659)	173.68% (132.16 to 226.85)	7730.2 (4876.4 to 10398.6)	5890.2 (3662.7 to 7930.3)	-0.98% (-1.11 to -0.85)
Albania	7563 (5470 to 8870)	3172 (2167 to 3761)	-58.05% (-64.38 to -52.09)	5007.7 (3621.6 to 5873.3)	3053.3 (2085.3 to 3620)	-2.01% (-2.25 to -1.77)
Algeria	47279 (30079 to 64758)	31956 (21008 to 42192)	-32.41% (-42.95 to -19.94)	3809.7 (2423.8 to 5218.2)	2003.3 (1317 to 2645)	-1.87% (-2.78 to -0.94)
American Samoa	96 (59 to 141)	56 (36 to 79)	-41.96% (-49.74 to -30.93)	3880.8 (2389.3 to 5703)	2214.7 (1426.3 to 3150.7)	-1.99% (-2.1 to -1.88)
Angola	23543 (14782 to 34901)	49226 (29976 to 71637)	109.09% (77.45 to 151.35)	5331.2 (3347.4 to 7903.4)	3692.3 (2248.5 to 5373.4)	-1.16% (-1.21 to -1.1)
Armenia	8501 (5726 to 10643)	3162 (2123 to 4017)	-62.8% (-68.48 to -56.57)	6003.7 (4044.3 to 7516.8)	3443.5 (2311.9 to 4374.7)	-1.8% (-2.16 to -1.45)
Azerbaijan	20103 (13303 to 25334)	14310 (9460 to 17959)	-28.82% (-40.15 to -16.38)	5542.9 (3668 to 6985)	3704.8 (2449.3 to 4649.5)	-1.06% (-1.24 to -0.88)
Bangladesh	228955 (144919 to 332186)	184320 (119177 to 266638)	-19.5% (-32.79 to -4.68)	4401.3 (2785.9 to 6385.8)	2376.4 (1536.5 to 3437.7)	-2.18% (-2.23 to -2.13)

Location	Incident cases in 1990 (95% UI)	Incident cases in 2019 (95% UI)	Relative change (%, 95 CI)	ASIR in 1990 (95% UI)	ASIR in 2019 (95% UI)	EAPC (%, 95 CI)
Belarus	18188 (12906 to 21786)	5820 (4144 to 6980)	-68% (-71.64 to -63.4)	5031 (3569.9 to 6026.3)	2594.9 (1847.5 to 3111.8)	-1.5% (-1.78 to -1.23)
Belize	444 (275 to 639)	518 (329 to 742)	16.71% (0.91 to 37.98)	5090.6 (3159.8 to 7325.9)	2558.8 (1626.2 to 3665.5)	-2.44% (-2.51 to -2.37)
Benin	6926 (4167 to 10544)	15510 (9285 to 23218)	123.94% (86.31 to 175.48)	3165.5 (1904.8 to 4819.2)	2562.9 (1534.2 to 3836.7)	-0.53% (-0.6 to -0.46)
Bhutan	1620 (1032 to 2310)	1049 (655 to 1481)	-35.24% (-45.21 to -23.94)	5523 (3519.9 to 7876.6)	2921.1 (1823.5 to 4124)	-2.28% (-2.42 to -2.14)
Bolivia (Plurinational State of)	14406 (9155 to 20504)	18792 (12117 to 26514)	30.44% (11.25 to 51.87)	5072.6 (3223.6 to 7219.8)	3557.5 (2294 to 5019.5)	-1.25% (-1.34 to -1.16)
Bosnia and Herzegovina	7045 (5171 to 8145)	1568 (1168 to 1816)	-77.75% (-81.12 to -74.24)	3691.4 (2709.2 to 4267.7)	1506.5 (1121.9 to 1745.4)	-3.28% (-3.6 to -2.96)
Botswana	2193 (1403 to 3136)	2615 (1678 to 3734)	19.22% (0.17 to 40.63)	3399.8 (2174.4 to 4861.8)	2495.2 (1601.5 to 3563.7)	-0.85% (-0.94 to -0.76)
Brazil	183839 (118628 to 255765)	221950 (157947 to 263536)	20.73% (-6.03 to 51)	2595.5 (1674.8 to 3611)	2554.6 (1817.9 to 3033.2)	0.76% (0.44 to 1.09)
Bulgaria	12131 (8452 to 14357)	2772 (1922 to 3271)	-77.15% (-80.97 to -73.31)	4267.5 (2973.5 to 5050.7)	1876.4 (1301.3 to 2214.4)	-2.27% (-2.63 to -1.9)

Location	Incident cases in 1990 (95% UI)	Incident cases in 2019 (95% UI)	Relative change (%, 95 CI)	ASIR in 1990 (95% UI)	ASIR in 2019 (95% UI)	EAPC (%, 95 CI)
Burkina Faso	13665 (8250 to 20727)	27403 (16816 to 40904)	100.53% (68.96 to 146.57)	3413.7 (2060.9 to 5177.8)	2679.5 (1644.3 to 3999.7)	-0.82% (-0.86 to -0.77)
Burundi	11652 (7048 to 16980)	21130 (12940 to 30410)	81.34% (53.53 to 113.1)	4786.6 (2895.2 to 6975)	3847.9 (2356.5 to 5537.9)	-0.51% (-0.58 to -0.44)
Cabo Verde	421 (252 to 633)	370 (225 to 546)	-12.06% (-30.13 to 7.12)	2513.6 (1506.7 to 3780.2)	1475.9 (898.2 to 2179.9)	-1.84% (-1.91 to -1.76)
Cambodia	22891 (14401 to 33132)	19309 (11734 to 28884)	-15.65% (-29.73 to 2.45)	4788.8 (3012.7 to 6931.4)	2591.4 (1574.8 to 3876.4)	-2.26% (-2.31 to -2.21)
Cameroon	14488 (8992 to 21463)	29612 (18240 to 42886)	104.38% (66.11 to 146.14)	3035.9 (1884.2 to 4497.2)	2125.9 (1309.5 to 3078.9)	-0.96% (-1.09 to -0.82)
Central African Republic	6491 (4010 to 9413)	10399 (6506 to 15351)	60.2% (37.46 to 89.76)	4953.6 (3060.3 to 7183.3)	3939 (2464.3 to 5814.8)	-0.76% (-0.8 to -0.73)
Chad	9575 (5816 to 14403)	23976 (14521 to 36386)	150.4% (103.79 to 209.41)	3774.6 (2292.8 to 5677.8)	3375.9 (2044.5 to 5123.2)	-0.46% (-0.51 to -0.41)
China	2087630 (1302187 to 3020908)	443402 (278275 to 642366)	-78.76% (-79.6 to -77.93)	3226.1 (2012.3 to 4668.4)	1134 (711.7 to 1642.9)	-2.89% (-3.22 to -2.57)
Colombia	60325 (37810 to 87347)	44988 (27432 to 64370)	-25.42% (-37.85 to -11.23)	3605.1 (2259.6 to 5219.9)	2202.2 (1342.9 to 3151)	-1.86% (-1.96 to -1.76)

Location	Incident cases in 1990 (95% UI)	Incident cases in 2019 (95% UI)	Relative change (%, 95 CI)	ASIR in 1990 (95% UI)	ASIR in 2019 (95% UI)	EAPC (%, 95 CI)
Comoros	1113 (690 to 1603)	813 (498 to 1184)	-26.95% (-39.6 to -10.62)	5303.2 (3289.3 to 7636.9)	2477 (1517.9 to 3606.4)	-2.6% (-2.66 to -2.54)
Congo	4913 (3082 to 7131)	5967 (3671 to 8835)	21.46% (1.9 to 44.3)	4292.9 (2692.8 to 6230.6)	2654.1 (1632.6 to 3929.4)	-1.36% (-1.48 to -1.24)
Costa Rica	4379 (2721 to 6387)	3289 (1959 to 4800)	-24.88% (-37.4 to -10.21)	3015.6 (1873.9 to 4398)	1634.3 (973.4 to 2384.8)	-2.14% (-2.27 to -2.02)
Côte d'Ivoire	17869 (11179 to 26408)	26916 (16288 to 39929)	50.63% (20.27 to 82.32)	3091.6 (1934.2 to 4568.8)	2312.2 (1399.2 to 3430)	-0.95% (-1.01 to -0.89)
Cuba	13049 (8074 to 18761)	6370 (4024 to 8937)	-51.18% (-58.87 to -41.46)	2305.6 (1426.6 to 3315)	1857.6 (1173.4 to 2606.1)	0.03% (-0.25 to 0.31)
Democratic People's Republic of Korea	46539 (28792 to 66873)	14656 (8944 to 21825)	-68.51% (-73.08 to -63.11)	4423.1 (2736.4 to 6355.6)	1470.7 (897.5 to 2190.1)	-3.9% (-4.08 to -3.72)
Democratic Republic of the Congo	92210 (57978 to 133852)	135749 (85379 to 205303)	47.22% (21.85 to 72.99)	5487.7 (3450.4 to 7965.9)	3383.2 (2127.8 to 5116.6)	-1.47% (-1.62 to -1.32)
Djibouti	1030 (637 to 1526)	1365 (874 to 1992)	32.48% (12.95 to 57.03)	4539.1 (2807.8 to 6724.4)	2960.4 (1895 to 4320.1)	-1.48% (-1.59 to -1.37)

Location	Incident cases in 1990 (95% UI)	Incident cases in 2019 (95% UI)	Relative change (%, 95 CI)	ASIR in 1990 (95% UI)	ASIR in 2019 (95% UI)	EAPC (%, 95 CI)
Dominica	107 (67 to 154)	39 (24 to 55)	-63.65% (-69.22 to -56.56)	3227.7 (2029.8 to 4637.4)	1528.8 (941.4 to 2171.3)	-2.8% (-2.9 to -2.71)
Dominican Republic	16727 (10421 to 24495)	15890 (10050 to 22043)	-5.01% (-19.55 to 13.79)	4331.2 (2698.3 to 6342.5)	3249.6 (2055.3 to 4508)	-1.18% (-1.27 to -1.09)
Ecuador	19683 (12241 to 27947)	21522 (13663 to 29543)	9.35% (-7.46 to 28.75)	4060.3 (2525.2 to 5765.2)	2708.6 (1719.5 to 3718.1)	-1.42% (-1.46 to -1.39)
Egypt	141743 (90347 to 189659)	148975 (95020 to 202839)	5.1% (-11.25 to 24.4)	5881.6 (3749 to 7869.9)	3456.6 (2204.7 to 4706.4)	-1.47% (-1.58 to -1.35)
El Salvador	9457 (5865 to 13628)	5524 (3287 to 8188)	-41.59% (-50.07 to -29.76)	3630.9 (2251.7 to 5232.5)	1735.7 (1032.8 to 2572.9)	-2.86% (-3.03 to -2.69)
Equatorial Guinea	1005 (631 to 1478)	1700 (1056 to 2515)	69.2% (42.15 to 104.89)	5372 (3374.8 to 7903.2)	2584 (1604.5 to 3821.7)	-2.51% (-2.6 to -2.42)
Eritrea	6088 (3716 to 9140)	9810 (6171 to 14650)	61.12% (35.3 to 93.19)	4600.3 (2808.1 to 6906.3)	2995 (1884.1 to 4472.8)	-1.38% (-1.47 to -1.3)
Eswatini	1602 (996 to 2310)	1630 (1040 to 2397)	1.8% (-15.36 to 19.98)	4010 (2492.5 to 5783.9)	2807.5 (1790.9 to 4127.7)	-0.94% (-1.02 to -0.86)
Ethiopia	121577 (74809 to 174505)	186779 (115411 to 270954)	53.63% (39.88 to 67.79)	5284 (3251.3 to 7584.4)	3518.9 (2174.3 to 5104.7)	-1.4% (-1.45 to -1.35)
Fiji	1253 (772 to 1856)	816 (508 to 1198)	-34.88% (-44.87 to -23.47)	3553.1 (2188.3 to 5262.5)	2237.3 (1391.4 to 3283.3)	-1.65% (-1.72 to -1.59)

Location	Incident cases in 1990 (95% UI)	Incident cases in 2019 (95% UI)	Relative change (%, 95 CI)	ASIR in 1990 (95% UI)	ASIR in 2019 (95% UI)	EAPC (%, 95 CI)
Gabon	2023 (1256 to 2983)	1954 (1224 to 2865)	-3.41% (-18.39 to 14.37)	4516.5 (2803.7 to 6660.1)	2209.8 (1384.8 to 3239.8)	-2.35% (-2.39 to -2.31)
Gambia	1470 (920 to 2184)	2238 (1416 to 3374)	52.2% (26.4 to 85.07)	3127.1 (1955.7 to 4644.4)	1962.2 (1241.3 to 2958.3)	-1.56% (-1.63 to -1.5)
Georgia	8115 (5317 to 10150)	3432 (2284 to 4298)	-57.71% (-63.62 to -50.48)	3853.6 (2525.1 to 4820.3)	3413 (2271.4 to 4273.9)	0.6% (0.08 to 1.12)
Ghana	17076 (10465 to 25967)	25043 (14936 to 37278)	46.66% (19.94 to 82.98)	2464 (1510.1 to 3746.9)	1568.3 (935.4 to 2334.6)	-1.41% (-1.49 to -1.33)
Global	9364130 (6135124 to 12799640)	7004614 (4621514 to 9559767)	-25.2% (-26.5 to -24.02)	3832 (2510.6 to 5237.9)	2368.2 (1562.5 to 3232.1)	-1.51% (-1.55 to -1.47)
Grenada	128 (79 to 185)	69 (43 to 100)	-46.64% (-54.54 to -35.36)	3505.2 (2156.1 to 5047.9)	1512.3 (943.6 to 2200.9)	-2.7% (-2.98 to -2.43)
Guatemala	20473 (12503 to 30552)	20419 (12218 to 30704)	-0.26% (-19.01 to 21.17)	5932.7 (3623 to 8853.2)	2201.4 (1317.2 to 3310.2)	-3.6% (-3.78 to -3.42)
Guinea	8518 (5165 to 12789)	14591 (8938 to 21554)	71.31% (37.23 to 121.54)	3263.7 (1979.3 to 4900.5)	2444.3 (1497.3 to 3610.7)	-0.99% (-1.08 to -0.89)
Guinea-Bissau	1285 (764 to 1896)	1836 (1120 to 2714)	42.83% (18.08 to 73.71)	2793.8 (1661.5 to 4122.3)	1943.5 (1185.7 to 2873.1)	-1.25% (-1.3 to -1.21)

Location	Incident cases in 1990 (95% UI)	Incident cases in 2019 (95% UI)	Relative change (%, 95 CI)	ASIR in 1990 (95% UI)	ASIR in 2019 (95% UI)	EAPC (%, 95 CI)
Guyana	1739 (1093 to 2455)	1066 (681 to 1523)	-38.66% (-47.09 to -28.49)	4267.1 (2681.6 to 6025.9)	2655.8 (1695.5 to 3792.3)	-1.67% (-1.69 to -1.64)
Haiti	11988 (7288 to 17419)	14474 (9005 to 20868)	20.74% (3.91 to 41.05)	4222.6 (2567.2 to 6135.5)	2530.7 (1574.4 to 3648.7)	-1.83% (-1.88 to -1.79)
Honduras	9143 (5719 to 13536)	13498 (8322 to 20079)	47.63% (21.88 to 76.97)	4523.2 (2829 to 6696.2)	2621 (1615.9 to 3898.8)	-2.22% (-2.35 to -2.09)
India	2014745 (1293061 to 2829155)	1691578 (1086589 to 2379992)	-16.04% (-19.81 to -12.85)	5406.6 (3470 to 7592.2)	2698.4 (1733.3 to 3796.5)	-2.21% (-2.43 to -1.99)
Indonesia	275405 (171979 to 394394)	182131 (114603 to 260105)	-33.87% (-37.53 to -30.72)	3025.5 (1889.3 to 4332.7)	1697.4 (1068.1 to 2424.1)	-1.83% (-1.97 to -1.69)
Iran (Islamic Republic of)	138189 (90431 to 179875)	48965 (31635 to 65136)	-64.57% (-66.68 to -62.31)	5069.3 (3317.3 to 6598.5)	1721.3 (1112.1 to 2289.8)	-2.92% (-3.1 to -2.73)
Iraq	44364 (28224 to 60753)	54783 (35145 to 75217)	23.49% (3.79 to 42.73)	6131.5 (3900.8 to 8396.7)	2604.6 (1670.9 to 3576.1)	-2.94% (-3.17 to -2.71)
Jamaica	3853 (2397 to 5595)	1876 (1195 to 2651)	-51.32% (-58.44 to -40.84)	3223 (2005.1 to 4680)	1418.5 (903.4 to 2004.3)	-3.03% (-3.12 to -2.95)
Jordan	7720 (4822 to 11026)	11113 (7597 to 14384)	43.95% (16.03 to 88.62)	4212.3 (2630.8 to 6016)	2228.1 (1523.1 to 2883.9)	-1.98% (-2.11 to -1.85)

Location	Incident cases in 1990 (95% UI)	Incident cases in 2019 (95% UI)	Relative change (%, 95 CI)	ASIR in 1990 (95% UI)	ASIR in 2019 (95% UI)	EAPC (%, 95 CI)
Kazakhstan	33558 (22039 to 42172)	24191 (15786 to 30218)	-27.91% (-37.12 to -15.26)	5160.7 (3389.3 to 6485.4)	3873.5 (2527.7 to 4838.6)	-0.51% (-0.85 to -0.16)
Kenya	60463 (38168 to 86746)	86909 (54996 to 123613)	43.74% (39.53 to 47.78)	5486.3 (3463.3 to 7871.1)	3466.4 (2193.5 to 4930.3)	-1.45% (-1.51 to -1.4)
Kiribati	128 (82 to 187)	113 (70 to 164)	-12.08% (-25.13 to 3.85)	3428.8 (2193.7 to 5003.7)	2093.4 (1302.3 to 3052.4)	-1.73% (-1.81 to -1.65)
Kyrgyzstan	11054 (7330 to 13986)	11934 (8101 to 15056)	7.96% (-6.18 to 24.19)	5874.7 (3895.4 to 7433)	4229.3 (2870.8 to 5335.8)	-0.54% (-0.97 to -0.1)
Lao People's Democratic Republic	8283 (5104 to 12305)	10236 (6176 to 15022)	23.58% (2.43 to 42.43)	4511.9 (2780.3 to 6703.1)	2983.7 (1800.4 to 4378.9)	-1.87% (-2.06 to -1.68)
Lebanon	7672 (4835 to 10424)	3869 (2500 to 5292)	-49.58% (-57.71 to -40.31)	5798.3 (3654.2 to 7877.7)	2328.1 (1504.3 to 3184.7)	-3.21% (-3.36 to -3.07)
Lesotho	3414 (2099 to 4903)	2791 (1757 to 4007)	-18.26% (-30.4 to -1.03)	4111.5 (2528.1 to 5904)	2686.6 (1690.9 to 3857.7)	-1.19% (-1.3 to -1.08)
Liberia	2131 (1292 to 3236)	4501 (2718 to 6761)	111.22% (74.64 to 158.16)	3109.1 (1884.4 to 4721.8)	1953.8 (1179.8 to 2934.9)	-1.53% (-1.62 to -1.44)
Libya	7098 (4605 to 9657)	1508 (983 to 2035)	-78.76% (-81.82 to -74.69)	3887.1 (2521.7 to 5288.4)	527.1 (343.7 to 711.6)	-6.13% (-6.58 to -5.68)

Location	Incident cases in 1990 (95% UI)	Incident cases in 2019 (95% UI)	Relative change (%, 95 CI)	ASIR in 1990 (95% UI)	ASIR in 2019 (95% UI)	EAPC (%, 95 CI)
Madagascar	24371 (14437 to 36167)	46174 (27710 to 69463)	89.46% (59.23 to 125.94)	4570 (2707.2 to 6781.9)	3623.3 (2174.4 to 5450.7)	-0.87% (-0.96 to -0.78)
Malawi	23844 (14384 to 34461)	30864 (18627 to 45498)	29.45% (10.21 to 55.31)	5240.8 (3161.6 to 7574.7)	3397.9 (2050.7 to 5009)	-1.47% (-1.63 to -1.31)
Malaysia	20465 (12789 to 29924)	12416 (7660 to 18489)	-39.33% (-48.76 to -28.18)	2519.7 (1574.6 to 3684.2)	894 (551.6 to 1331.3)	-4.43% (-4.78 to -4.08)
Maldives	449 (274 to 656)	266 (165 to 391)	-40.91% (-50.58 to -30.73)	4328.3 (2639.9 to 6319.3)	1685.8 (1045 to 2479.7)	-2.74% (-3.03 to -2.44)
Mali	12982 (7962 to 19577)	28952 (17436 to 43370)	123.01% (84.25 to 171.55)	3548.8 (2176.4 to 5351.4)	2836.6 (1708.3 to 4249.2)	-0.75% (-0.77 to -0.73)
Marshall Islands	89 (55 to 132)	73 (45 to 107)	-17.55% (-30.61 to -1.23)	4681.4 (2904.2 to 6929.9)	3108.5 (1910.9 to 4543.3)	-1.39% (-1.43 to -1.34)
Mauritania	2756 (1713 to 4091)	3568 (2195 to 5285)	29.48% (4.5 to 55.81)	3006.2 (1868.4 to 4463.2)	1841.6 (1132.8 to 2728)	-1.64% (-1.7 to -1.59)
Mexico	152997 (95853 to 214042)	142518 (94974 to 187198)	-6.85% (-17.34 to 5.79)	3513.5 (2201.2 to 4915.4)	2642.6 (1761 to 3471.1)	-0.33% (-0.78 to 0.12)
Micronesia (Federated States of)	153 (95 to 225)	92 (56 to 136)	-39.74% (-49.42 to -30.15)	3613.7 (2238.4 to 5317.9)	1983.4 (1210.5 to 2923.6)	-2.12% (-2.28 to -1.97)

Location	Incident cases in 1990 (95% UI)	Incident cases in 2019 (95% UI)	Relative change (%, 95 CI)	ASIR in 1990 (95% UI)	ASIR in 2019 (95% UI)	EAPC (%, 95 CI)
Mongolia	7047 (4676 to 8734)	6037 (3985 to 7535)	-14.34% (-26.04 to 1.17)	6852.1 (4546.4 to 8492.1)	5007 (3305.1 to 6249.5)	-0.74% (-1.08 to -0.39)
Montenegro	815 (559 to 968)	306 (205 to 367)	-62.48% (-69.4 to -56.22)	3328.4 (2285.4 to 3954.5)	1547 (1037.6 to 1858.4)	-2.99% (-3.14 to -2.84)
Morocco	47842 (30485 to 64567)	25661 (16246 to 34814)	-46.36% (-55.11 to -37.14)	3918.6 (2496.9 to 5288.4)	1730.8 (1095.8 to 2348.2)	-2.57% (-2.82 to -2.32)
Mozambique	26793 (16442 to 38872)	55392 (33744 to 80655)	106.74% (75.16 to 144.71)	4811.6 (2952.7 to 6980.9)	3799.6 (2314.7 to 5532.5)	-0.75% (-0.83 to -0.66)
Myanmar	75704 (46010 to 110264)	51611 (31919 to 76288)	-31.82% (-42.04 to -19.1)	3872.7 (2353.7 to 5640.7)	2172.7 (1343.7 to 3211.5)	-1.95% (-1.98 to -1.93)
Namibia	2408 (1512 to 3495)	2780 (1701 to 4047)	15.46% (-3.28 to 37.48)	3470.5 (2179.5 to 5037)	2389.4 (1461.6 to 3478.2)	-0.99% (-1.09 to -0.88)
Nepal	48313 (29996 to 71007)	48764 (30796 to 70777)	0.93% (-15.44 to 20.04)	5515.1 (3424.1 to 8105.6)	2993.6 (1890.6 to 4345)	-2.32% (-2.46 to -2.19)
Nicaragua	9238 (5446 to 13610)	7176 (4329 to 10545)	-22.32% (-35.12 to -7.57)	5074.7 (2991.7 to 7476.5)	2357.6 (1422.1 to 3464.5)	-2.94% (-3.07 to -2.81)
Niger	13538 (8453 to 20340)	38954 (23543 to 58000)	187.74% (133.72 to 263.81)	3973.4 (2481 to 5969.7)	3805 (2299.6 to 5665.3)	-0.09% (-0.11 to -0.06)

Location	Incident cases in 1990 (95% UI)	Incident cases in 2019 (95% UI)	Relative change (%, 95 CI)	ASIR in 1990 (95% UI)	ASIR in 2019 (95% UI)	EAPC (%, 95 CI)
Nigeria	148837 (94941 to 214162)	264237 (171818 to 376847)	77.53% (69.9 to 86.49)	3557.8 (2269.5 to 5119.3)	2523.2 (1640.7 to 3598.6)	-1.51% (-1.96 to -1.06)
North Macedonia	3702 (2550 to 4362)	1012 (670 to 1231)	-72.65% (-77.72 to -67.94)	4512.5 (3108 to 5316.9)	1471.1 (973.4 to 1788.3)	-4.26% (-4.5 to -4.03)
Pakistan	298636 (191936 to 415616)	460122 (293136 to 648567)	54.07% (39.87 to 69.9)	6556.8 (4214.1 to 9125.1)	4438.3 (2827.5 to 6256)	-1.42% (-1.5 to -1.34)
Papua New Guinea	7918 (4930 to 11503)	16953 (10701 to 24894)	114.11% (81.02 to 155.12)	4211.4 (2622.1 to 6118)	3813.9 (2407.3 to 5600.3)	-0.31% (-0.35 to -0.27)
Paraguay	7419 (4467 to 10584)	6476 (4066 to 9174)	-12.71% (-26.48 to 3.3)	4222.3 (2542.3 to 6023.6)	2060.9 (1293.9 to 2919.6)	-2.56% (-2.6 to -2.51)
Peru	44893 (28144 to 63529)	32855 (19886 to 47773)	-26.82% (-37.5 to -14.15)	4215.6 (2642.8 to 5965.5)	2366 (1432 to 3440.3)	-2.12% (-2.16 to -2.07)
Philippines	124558 (78324 to 177534)	153588 (97040 to 219273)	23.31% (19.02 to 27.37)	4132.4 (2598.5 to 5890)	3067.5 (1938.1 to 4379.3)	-1.03% (-1.15 to -0.91)
Republic of Moldova	8882 (6448 to 10571)	2355 (1733 to 2807)	-73.48% (-76.76 to -70.03)	5815.5 (4222 to 6921.2)	2298.5 (1690.7 to 2739.2)	-3% (-3.23 to -2.77)
Russian Federation	207295 (140851 to 254401)	83984 (57237 to 103176)	-59.49% (-60.3 to -58.77)	4246.7 (2885.5 to 5211.7)	2415 (1645.9 to 2966.9)	-1.32% (-1.7 to -0.94)
Rwanda	14175 (8711 to 20731)	16232 (9879 to 23530)	14.51% (-2.08 to 33.4)	4508.4 (2770.4 to 6593.4)	2620.6 (1594.9 to 3798.8)	-2.01% (-2.13 to -1.9)

Location	Incident cases in 1990 (95% UI)	Incident cases in 2019 (95% UI)	Relative change (%, 95 CI)	ASIR in 1990 (95% UI)	ASIR in 2019 (95% UI)	EAPC (%, 95 CI)
Saint Lucia	240 (152 to 342)	88 (53 to 128)	-63.46% (-69.78 to -56.64)	3500.1 (2212.1 to 4986.5)	1259.5 (760.4 to 1836.1)	-3.41% (-3.65 to -3.16)
Saint Vincent and the Grenadines	170 (107 to 244)	80 (49 to 115)	-53.27% (-60.32 to -44.43)	3216.8 (2024.8 to 4607.4)	1836.5 (1124.3 to 2651.5)	-1.91% (-1.99 to -1.84)
Samoa	169 (104 to 250)	89 (55 to 129)	-47.5% (-55.03 to -37.59)	2359.1 (1454.9 to 3481.5)	1000.4 (619.6 to 1448.7)	-3% (-3.11 to -2.9)
Senegal	9629 (5915 to 14410)	12982 (8141 to 19234)	34.82% (11.7 to 59.53)	2809.4 (1725.6 to 4204.2)	1847.4 (1158.5 to 2737.1)	-1.25% (-1.35 to -1.14)
Serbia	9559 (5871 to 13364)	2812 (1733 to 4052)	-70.58% (-75.06 to -65.61)	3057.5 (1878 to 4274.7)	1091.8 (673 to 1573.4)	-3.98% (-4.2 to -3.76)
Sierra Leone	4904 (2928 to 7305)	9438 (5767 to 13973)	92.45% (56.77 to 135.35)	2940.6 (1755.7 to 4380.2)	2223.4 (1358.6 to 3291.5)	-0.94% (-1 to -0.88)
Solomon Islands	764 (462 to 1133)	1130 (699 to 1663)	47.81% (26.3 to 74.94)	4917.8 (2973.4 to 7290.2)	3915 (2420.9 to 5763.3)	-0.8% (-0.85 to -0.76)
Somalia	13016 (8035 to 18802)	41184 (25429 to 60396)	216.41% (171.44 to 274.07)	5729.8 (3537 to 8276.8)	4557.7 (2814.2 to 6683.8)	-0.65% (-0.69 to -0.61)
South Africa	52038 (33072 to 73726)	46026 (29486 to 65419)	-11.55% (-17.58 to -5.81)	2726.4 (1732.8 to 3862.7)	2010 (1287.7 to 2856.9)	-0.85% (-0.93 to -0.77)

Location	Incident cases in 1990 (95% UI)	Incident cases in 2019 (95% UI)	Relative change (%, 95 CI)	ASIR in 1990 (95% UI)	ASIR in 2019 (95% UI)	EAPC (%, 95 CI)
South Sudan	14497 (9038 to 21369)	19570 (12202 to 28691)	34.99% (13.26 to 59.91)	5347.9 (3334.2 to 7883.1)	4713.5 (2938.8 to 6910.4)	-0.39% (-0.44 to -0.34)
Sri Lanka	18626 (11606 to 27392)	9142 (5720 to 13255)	-50.92% (-59.18 to -41.31)	2307.7 (1437.9 to 3393.8)	1125.4 (704.1 to 1631.7)	-2.12% (-2.32 to -1.92)
Sudan	67511 (43588 to 89812)	68379 (44125 to 90364)	1.29% (-13.18 to 21.09)	7205.2 (4652 to 9585.3)	3517 (2269.5 to 4647.8)	-2.38% (-2.58 to -2.19)
Suriname	668 (412 to 955)	476 (301 to 686)	-28.77% (-39.4 to -18.11)	3512.3 (2168.2 to 5020.8)	2113 (1335.9 to 3047.6)	-1.72% (-1.84 to -1.6)
Syrian Arab Republic	31968 (20642 to 43774)	13530 (8670 to 18351)	-57.68% (-64.38 to -50.7)	5608.6 (3621.6 to 7680)	1803.7 (1155.8 to 2446.4)	-3.35% (-3.63 to -3.06)
Tajikistan	18326 (12147 to 22843)	23545 (15565 to 29558)	28.48% (9.91 to 49.36)	7444.1 (4934.1 to 9278.8)	5427 (3587.5 to 6812.9)	-1% (-1.09 to -0.92)
Thailand	68344 (41004 to 99719)	28615 (18279 to 41124)	-58.13% (-64.37 to -50.18)	2344.5 (1406.6 to 3420.9)	1178.9 (753.1 to 1694.2)	-2.29% (-2.36 to -2.21)
Timor-Leste	1761 (1114 to 2547)	2010 (1251 to 2937)	14.16% (-3.69 to 36.2)	4859.6 (3073.3 to 7028.5)	3036.5 (1889.5 to 4435.9)	-1.75% (-1.97 to -1.53)
Togo	4652 (2892 to 6863)	6696 (4201 to 9956)	43.93% (18.9 to 74.61)	2760 (1715.9 to 4071.7)	1907.7 (1196.7 to 2836.4)	-1.03% (-1.12 to -0.94)
Tonga	122 (75 to 178)	81 (51 to 119)	-33.4% (-44.54 to -20.2)	2809.3 (1731.3 to 4095.8)	1872.4 (1171.6 to 2747.1)	-1.28% (-1.34 to -1.23)

Location	Incident cases in 1990 (95% UI)	Incident cases in 2019 (95% UI)	Relative change (%, 95 CI)	ASIR in 1990 (95% UI)	ASIR in 2019 (95% UI)	EAPC (%, 95 CI)
Tunisia	17372 (11007 to 23417)	4329 (2798 to 5795)	-75.08% (-78.73 to -70.6)	4312.8 (2732.5 to 5813.5)	1064.1 (687.9 to 1424.5)	-4.86% (-4.97 to -4.76)
Turkey	169024 (123229 to 199370)	74962 (53311 to 88430)	-55.65% (-61.63 to -49.54)	6164 (4494 to 7270.7)	2297.9 (1634.2 to 2710.8)	-3.28% (-3.41 to -3.15)
Turkmenistan	10247 (6915 to 12791)	8312 (5519 to 10448)	-18.89% (-30.92 to -5.93)	6007.2 (4053.9 to 7498.1)	4325.5 (2871.9 to 5437.4)	-0.97% (-1.29 to -0.65)
Tuvalu	13 (8 to 19)	10 (6 to 14)	-24.96% (-35.62 to -12.42)	3288.7 (2044.5 to 4718.6)	1986.7 (1229.5 to 2863.8)	-1.74% (-1.77 to -1.71)
Uganda	51678 (31855 to 75134)	85072 (51872 to 126539)	64.62% (38.92 to 95.31)	6234.7 (3843.2 to 9064.6)	4224.3 (2575.7 to 6283.3)	-1.38% (-1.48 to -1.28)
Ukraine	80847 (56041 to 101145)	26060 (17763 to 32634)	-67.77% (-72.1 to -62.61)	4752.1 (3294 to 5945.2)	2405.5 (1639.6 to 3012.4)	-1.69% (-2.09 to -1.29)
United Republic of Tanzania	64025 (39051 to 96956)	105284 (65442 to 155068)	64.44% (39.27 to 93.25)	5174 (3155.8 to 7835.1)	3859.8 (2399.1 to 5684.9)	-0.91% (-0.96 to -0.86)
Uzbekistan	74704 (51478 to 92805)	82995 (57635 to 103061)	11.1% (-4.47 to 29.62)	7853.7 (5411.9 to 9756.7)	5665.1 (3934.1 to 7034.8)	-1.13% (-1.33 to -0.94)
Vanuatu	287 (178 to 420)	425 (264 to 618)	48.25% (26.39 to 74.36)	4264.5 (2646.8 to 6245.4)	3119.2 (1937.3 to 4538.3)	-1.1% (-1.17 to -1.04)

Location	Incident cases in 1990 (95% UI)	Incident cases in 2019 (95% UI)	Relative change (%, 95 CI)	ASIR in 1990 (95% UI)	ASIR in 2019 (95% UI)	EAPC (%, 95 CI)
Venezuela (Bolivarian Republic of)	29989 (17829 to 45401)	24496 (15003 to 35368)	-18.31% (-32.59 to -0.63)	3328.1 (1978.7 to 5038.5)	2329.8 (1427 to 3363.8)	-0.74% (-1.04 to -0.45)
Viet Nam	118212 (74089 to 172266)	66461 (41077 to 97376)	-43.78% (-52.9 to -32.92)	3553.9 (2227.4 to 5178.9)	1862 (1150.8 to 2728.1)	-2.09% (-2.21 to -1.98)
Yemen	41634 (26653 to 55529)	64154 (40778 to 85294)	54.09% (27.21 to 81.73)	8354.6 (5348.5 to 11143)	4765 (3028.8 to 6335.2)	-1.92% (-2.01 to -1.82)
Zambia	20349 (12528 to 29616)	30312 (18461 to 44634)	48.96% (24.9 to 78.77)	5110.9 (3146.7 to 7438.5)	3274.9 (1994.6 to 4822.3)	-1.48% (-1.58 to -1.37)
Zimbabwe	21955 (13825 to 32217)	24894 (15398 to 36550)	13.38% (-7.75 to 32.55)	4438.5 (2794.9 to 6512.9)	3397.3 (2101.4 to 4988.1)	-0.59% (-0.7 to -0.49)

ASIR – age-standardized incidence rate (per 100,000 population), CI – confidence interval, EAPC – estimated annual percentage change, MSMI – maternal sepsis and other maternal infections, UI – uncertain interval.

Table S10. Incidence of MSMI in 131 low- and middle-income countries and territories with EAPC in age group of 25-29 years from 1990 and 2019.

Location	Incident cases in 1990 (95% UI)	Incident cases in 2019 (95% UI)	Relative change (%, 95 CI)	ASIR in 1990 (95% UI)	ASIR in 2019 (95% UI)	EAPC (%, 95 CI)
Afghanistan	17550 (12198 to 24384)	64257 (43581 to 87699)	266.14% (212.58 to 324.96)	5095.5 (3541.6 to 7079.8)	4146.1 (2812 to 5658.6)	-0.7% (-0.82 to -0.58)
Albania	4397 (3297 to 5838)	1989 (1480 to 2606)	-54.77% (-61.13 to -47.57)	2930.8 (2197.8 to 3891.3)	1962.1 (1460 to 2571.4)	-1.55% (-1.75 to -1.35)
Algeria	30025 (20832 to 41078)	40146 (28009 to 53847)	33.71% (11.54 to 55.77)	3053.6 (2118.7 to 4177.8)	2236.3 (1560.2 to 2999.5)	-0.72% (-1.31 to -0.12)
American Samoa	69 (48 to 98)	38 (26 to 55)	-44.77% (-51.93 to -35.56)	3166.4 (2171.8 to 4459.4)	1964 (1317.3 to 2805.8)	-1.67% (-1.75 to -1.59)
Angola	14631 (9923 to 21056)	32365 (21123 to 46112)	121.21% (81.95 to 166.5)	3734.1 (2532.5 to 5373.9)	2767.1 (1806 to 3942.5)	-0.98% (-1.05 to -0.91)
Armenia	3993 (2781 to 5601)	2409 (1665 to 3331)	-39.67% (-47.9 to -29.81)	2403 (1673.5 to 3370.9)	1865.4 (1289.3 to 2579.2)	0.12% (-0.32 to 0.57)
Azerbaijan	11897 (8293 to 16400)	8280 (5799 to 11490)	-30.4% (-41.56 to -18.04)	3090.2 (2154.1 to 4259.6)	1666.1 (1166.9 to 2311.9)	-1.46% (-1.73 to -1.19)

Location	Incident cases in 1990 (95% UI)	Incident cases in 2019 (95% UI)	Relative change (%, 95 CI)	ASIR in 1990 (95% UI)	ASIR in 2019 (95% UI)	EAPC (%, 95 CI)
Bangladesh	105236 (72698 to 147168)	92933 (63919 to 127856)	-11.69% (-26.73 to 3.38)	2476.7 (1710.9 to 3463.6)	1273.6 (876 to 1752.1)	-2.47% (-2.61 to -2.33)
Belarus	10032 (6639 to 13787)	9005 (6039 to 12284)	-10.23% (-21.34 to 2.55)	2349.5 (1554.9 to 3229.2)	2942.8 (1973.6 to 4014.2)	2.4% (1.79 to 3.01)
Belize	227 (155 to 318)	286 (192 to 404)	25.75% (5.48 to 47.21)	3137 (2139.7 to 4393.4)	1589.9 (1068.1 to 2248.4)	-2.25% (-2.35 to -2.16)
Benin	5703 (3755 to 8347)	11868 (7564 to 17457)	108.1% (67.68 to 160.19)	2843.1 (1872 to 4161.5)	2341.8 (1492.5 to 3444.8)	-0.44% (-0.52 to -0.37)
Bhutan	772 (533 to 1073)	491 (335 to 676)	-36.36% (-46.2 to -25.09)	3347.6 (2312.7 to 4650.7)	1419.1 (968.4 to 1951.5)	-3.53% (-3.75 to -3.31)
Bolivia (Plurinational State of)	8887 (6023 to 12630)	11755 (7866 to 16841)	32.27% (12.51 to 57.78)	3618.1 (2452.1 to 5141.6)	2325.5 (1556 to 3331.6)	-1.66% (-1.9 to -1.42)
Bosnia and Herzegovina	3247 (2410 to 4240)	1261 (957 to 1658)	-61.15% (-66.4 to -55.44)	1684.4 (1250.5 to 2200)	1276.6 (968.9 to 1678.2)	-1.06% (-1.53 to -0.59)
Botswana	1265 (857 to 1788)	1935 (1338 to 2724)	52.91% (27.82 to 82.73)	2333.3 (1580.9 to 3296.7)	1741.6 (1204.7 to 2451.9)	-0.77% (-1.05 to -0.49)

Location	Incident cases in 1990 (95% UI)	Incident cases in 2019 (95% UI)	Relative change (%, 95 CI)	ASIR in 1990 (95% UI)	ASIR in 2019 (95% UI)	EAPC (%, 95 CI)
Brazil	98928 (69964 to 134588)	148110 (108581 to 199908)	49.72% (32.95 to 66.66)	1505.9 (1065 to 2048.7)	1709.1 (1253 to 2306.8)	1.1% (0.82 to 1.39)
Bulgaria	3037 (2233 to 4021)	2440 (1799 to 3237)	-19.66% (-29.92 to -7.52)	1082 (795.4 to 1432.7)	1281.6 (944.8 to 1700.5)	1.31% (1.07 to 1.55)
Burkina Faso	9830 (6370 to 14892)	20409 (13428 to 30177)	107.63% (67.06 to 171.22)	2820.8 (1827.9 to 4273.4)	2317.8 (1524.9 to 3427)	-0.73% (-0.77 to -0.68)
Burundi	8821 (5774 to 12564)	15177 (10260 to 21817)	72.06% (45.71 to 103.02)	3948.4 (2584.4 to 5624.2)	3285.4 (2221.1 to 4722.8)	-0.46% (-0.52 to -0.4)
Cabo Verde	282 (182 to 411)	246 (162 to 355)	-12.86% (-28.2 to 6.79)	1944.9 (1257 to 2833.9)	972.5 (641.6 to 1408)	-2.72% (-2.85 to -2.59)
Cambodia	17228 (11500 to 25041)	16004 (10234 to 22986)	-7.11% (-20 to 8.73)	4024.7 (2686.5 to 5849.9)	2110.3 (1349.5 to 3030.9)	-2.41% (-2.63 to -2.19)
Cameroon	10339 (6811 to 14954)	21198 (13687 to 31430)	105.03% (69.32 to 150.5)	2514.2 (1656.3 to 3636.5)	1702.3 (1099.2 to 2524.1)	-1.01% (-1.25 to -0.77)
Central African Republic	3747 (2539 to 5292)	5523 (3712 to 7991)	47.41% (25.24 to 76.6)	3350.9 (2271 to 4732.9)	2620.9 (1761.5 to 3792.2)	-0.85% (-0.88 to -0.81)

Location	Incident cases in 1990 (95% UI)	Incident cases in 2019 (95% UI)	Relative change (%, 95 CI)	ASIR in 1990 (95% UI)	ASIR in 2019 (95% UI)	EAPC (%, 95 CI)
Chad	6708 (4378 to 10023)	16535 (10640 to 24307)	146.48% (99.32 to 204.95)	3102 (2024.4 to 4634.7)	2879.5 (1852.9 to 4232.9)	-0.31% (-0.35 to -0.27)
China	1102815 (742367 to 1571939)	631225 (424963 to 902998)	-42.76% (-45.1 to -40.52)	2055.8 (1383.9 to 2930.4)	1161.2 (781.7 to 1661.1)	-1.05% (-1.45 to -0.65)
Colombia	31729 (21360 to 46620)	28680 (19150 to 41094)	-9.61% (-23.43 to 7.23)	2066.5 (1391.1 to 3036.3)	1431.8 (956 to 2051.6)	-1.39% (-1.46 to -1.32)
Comoros	725 (488 to 1040)	551 (370 to 784)	-23.99% (-36.74 to -7.75)	4226.6 (2846.3 to 6066.5)	1847.6 (1241.8 to 2628.7)	-3.17% (-3.28 to -3.06)
Congo	2803 (1907 to 3967)	3787 (2527 to 5383)	35.12% (14.27 to 61.36)	3000.8 (2041.9 to 4247.3)	1789.1 (1193.6 to 2543.2)	-1.31% (-1.51 to -1.12)
Costa Rica	2778 (1826 to 4022)	2301 (1548 to 3301)	-17.17% (-31.59 to 0.14)	1974.7 (1297.9 to 2858.6)	1106 (744 to 1586.1)	-1.98% (-2.18 to -1.78)
Côte d'Ivoire	13046 (8257 to 18961)	19876 (12913 to 29520)	52.36% (22.65 to 87.06)	2620.7 (1658.7 to 3808.9)	1820.2 (1182.5 to 2703.3)	-1.24% (-1.29 to -1.19)
Cuba	7373 (5035 to 10375)	4582 (3095 to 6476)	-37.86% (-47.76 to -27.61)	1350.6 (922.3 to 1900.4)	1234.3 (833.8 to 1744.7)	0.14% (-0.11 to 0.39)

Location	Incident cases in 1990 (95% UI)	Incident cases in 2019 (95% UI)	Relative change (%, 95 CI)	ASIR in 1990 (95% UI)	ASIR in 2019 (95% UI)	EAPC (%, 95 CI)
Democratic People's Republic of Korea	34685 (23169 to 49984)	19123 (12390 to 27833)	-44.87% (-53.64 to -35.25)	3791.4 (2532.5 to 5463.7)	1807.2 (1170.9 to 2630.2)	-2.72% (-2.85 to -2.6)
Democratic Republic of the Congo	56976 (38071 to 80932)	88069 (57759 to 127382)	54.57% (23.03 to 87.3)	3947.9 (2638 to 5607.9)	2587.3 (1696.8 to 3742.2)	-1.22% (-1.4 to -1.05)
Djibouti	627 (406 to 893)	992 (674 to 1413)	58.16% (31.83 to 92.66)	3628.7 (2351.2 to 5170.2)	2020.6 (1372.7 to 2880.4)	-2.07% (-2.28 to -1.86)
Dominica	58 (39 to 83)	31 (20 to 44)	-47.33% (-55.99 to -38)	2089.4 (1392.2 to 2992.2)	1222 (815.7 to 1747.9)	-2.13% (-2.24 to -2.02)
Dominican Republic	8350 (5680 to 11843)	9310 (6243 to 13139)	11.5% (-6.68 to 29.86)	2562.1 (1743.1 to 3634)	1996.7 (1338.9 to 2817.9)	-1% (-1.19 to -0.82)
Ecuador	10771 (7235 to 15422)	13416 (9080 to 19090)	24.56% (5.07 to 46.79)	2551.6 (1713.9 to 3653.4)	1848.5 (1251 to 2630.3)	-1.15% (-1.26 to -1.04)
Egypt	77513 (53177 to 107167)	92573 (64015 to 126821)	19.43% (3.62 to 39.19)	3650 (2504 to 5046.4)	2284.4 (1579.7 to 3129.6)	-1.35% (-1.49 to -1.21)
El Salvador	5201 (3484 to 7475)	3768 (2455 to 5392)	-27.54% (-39.18 to -15.02)	2422.4 (1623 to 3481.9)	1303.3 (849.2 to 1865)	-2.34% (-2.48 to -2.2)

Location	Incident cases in 1990 (95% UI)	Incident cases in 2019 (95% UI)	Relative change (%, 95 CI)	ASIR in 1990 (95% UI)	ASIR in 2019 (95% UI)	EAPC (%, 95 CI)
Equatorial Guinea	638 (427 to 888)	1035 (686 to 1463)	62.25% (37.6 to 94.53)	4043.6 (2708.5 to 5628.4)	1821.3 (1208.2 to 2575.1)	-2.75% (-2.98 to -2.51)
Eritrea	3905 (2577 to 5577)	5349 (3586 to 7640)	36.99% (15.03 to 65.2)	3458.3 (2282.4 to 4939.4)	1918 (1285.8 to 2739.3)	-1.91% (-2.09 to -1.73)
Eswatini	1005 (690 to 1430)	895 (602 to 1268)	-10.92% (-24.99 to 3.78)	3053.4 (2097 to 4344.5)	1626.1 (1094 to 2302.9)	-1.9% (-1.98 to -1.82)
Ethiopia	80384 (54295 to 112708)	112405 (76220 to 159422)	39.84% (28.04 to 53.61)	4095.6 (2766.4 to 5742.5)	2589.2 (1755.7 to 3672.2)	-1.6% (-1.7 to -1.5)
Fiji	697 (472 to 969)	692 (462 to 962)	-0.73% (-17.4 to 15.37)	2096.5 (1419.1 to 2914.6)	1988.8 (1329.1 to 2767.5)	-0.01% (-0.07 to 0.05)
Gabon	1206 (776 to 1732)	1309 (888 to 1832)	8.54% (-8.26 to 30.47)	3140.9 (2020.7 to 4510.8)	1613.9 (1094.6 to 2259)	-2.48% (-2.65 to -2.31)
Gambia	1198 (794 to 1814)	1648 (1071 to 2448)	37.5% (10.71 to 70.68)	2825.5 (1870.9 to 4276)	1694.7 (1101.6 to 2517.8)	-1.69% (-1.84 to -1.54)
Georgia	3879 (2702 to 5312)	2496 (1756 to 3432)	-35.66% (-43.9 to -25.94)	1591.3 (1108.4 to 2179.1)	1996.9 (1404.7 to 2746)	1.73% (1.33 to 2.12)

Location	Incident cases in 1990 (95% UI)	Incident cases in 2019 (95% UI)	Relative change (%, 95 CI)	ASIR in 1990 (95% UI)	ASIR in 2019 (95% UI)	EAPC (%, 95 CI)
Ghana	14202 (9177 to 21084)	20639 (13479 to 30502)	45.32% (18.26 to 79.28)	2297 (1484.3 to 3410.1)	1391.3 (908.6 to 2056.1)	-1.78% (-1.92 to -1.65)
Global	5606513 (3939975 to 7684054)	5490967 (3864321 to 7512201)	-2.06% (-3.64 to -0.49)	2547.1 (1790 to 3491)	1826.1 (1285.1 to 2498.3)	-0.93% (-1 to -0.85)
Grenada	66 (44 to 93)	54 (37 to 77)	-18.4% (-30.96 to -2.9)	2033.4 (1355.6 to 2873.4)	1233.2 (837.9 to 1757.8)	-1.58% (-1.82 to -1.34)
Guatemala	11535 (7728 to 16398)	13728 (8869 to 19751)	19.01% (-3.7 to 44.63)	4040 (2706.7 to 5743.3)	1622.7 (1048.4 to 2334.7)	-3.56% (-3.93 to -3.19)
Guinea	6538 (4231 to 9744)	10431 (6927 to 15189)	59.55% (26.88 to 103.23)	2741.6 (1774.2 to 4086.1)	1971.4 (1309.2 to 2870.5)	-1.16% (-1.24 to -1.07)
Guinea-Bissau	991 (653 to 1438)	1345 (887 to 1998)	35.72% (10.64 to 65.99)	2505.4 (1650.4 to 3635.5)	1532 (1009.7 to 2275.1)	-1.68% (-1.78 to -1.57)
Guyana	757 (507 to 1037)	549 (360 to 775)	-27.52% (-38.89 to -14.88)	2131.4 (1428.3 to 2920.5)	1574.3 (1032.5 to 2223)	-0.78% (-0.89 to -0.68)
Haiti	9176 (6131 to 12971)	11861 (7952 to 16927)	29.25% (9.52 to 53.3)	3589.9 (2398.6 to 5074.2)	2086.7 (1399.1 to 2978.1)	-1.82% (-1.93 to -1.7)

Location	Incident cases in 1990 (95% UI)	Incident cases in 2019 (95% UI)	Relative change (%, 95 CI)	ASIR in 1990 (95% UI)	ASIR in 2019 (95% UI)	EAPC (%, 95 CI)
Honduras	5443 (3628 to 7628)	7587 (5114 to 10805)	39.4% (14.94 to 66.4)	3167.2 (2111.2 to 4438.7)	1671.2 (1126.5 to 2379.9)	-2.54% (-2.82 to -2.26)
India	1156734 (805391 to 1583095)	1208623 (842819 to 1670091)	4.49% (-0.3 to 8.41)	3482.3 (2424.6 to 4765.9)	2083.9 (1453.2 to 2879.6)	-1.89% (-1.95 to -1.82)
Indonesia	177855 (121102 to 254368)	177429 (120041 to 251017)	-0.24% (-4.85 to 4.59)	2166.8 (1475.4 to 3098.9)	1739.7 (1177 to 2461.2)	-0.75% (-0.89 to -0.61)
Iran (Islamic Republic of)	60539 (42368 to 81664)	55155 (38786 to 74729)	-8.89% (-13.6 to -3.69)	2755.7 (1928.6 to 3717.3)	1585.3 (1114.8 to 2147.9)	-1.78% (-2.2 to -1.35)
Iraq	25440 (17577 to 35321)	37349 (26258 to 50932)	46.81% (28.1 to 68.42)	4223.1 (2917.8 to 5863.4)	1913.1 (1345 to 2608.9)	-3.06% (-3.21 to -2.91)
Jamaica	1890 (1251 to 2665)	1364 (925 to 1945)	-27.83% (-38.38 to -15.2)	1796.6 (1189.1 to 2533.2)	1049.4 (711.9 to 1496.8)	-1.95% (-2.03 to -1.88)
Jordan	5207 (3609 to 7247)	10407 (7867 to 13224)	99.88% (62.87 to 149.16)	3834.1 (2657.7 to 5336.9)	2273.3 (1718.5 to 2888.7)	-1.48% (-1.71 to -1.25)
Kazakhstan	18900 (13052 to 26340)	19921 (13537 to 27913)	5.4% (-9.29 to 20.07)	2515.6 (1737.3 to 3506)	2536.6 (1723.7 to 3554.2)	0.88% (0.54 to 1.22)

Location	Incident cases in 1990 (95% UI)	Incident cases in 2019 (95% UI)	Relative change (%, 95 CI)	ASIR in 1990 (95% UI)	ASIR in 2019 (95% UI)	EAPC (%, 95 CI)
Kenya	35073 (24153 to 49140)	40915 (28079 to 57260)	16.66% (13.38 to 20)	3938.7 (2712.4 to 5518.5)	1911.8 (1312 to 2675.5)	-2.37% (-2.4 to -2.34)
Kiribati	91 (61 to 129)	121 (82 to 172)	32.92% (12.73 to 58)	2717.1 (1805.7 to 3832.9)	2288.3 (1539.2 to 3241.2)	-0.43% (-0.48 to -0.38)
Kyrgyzstan	6599 (4619 to 9066)	7762 (5428 to 10791)	17.64% (2.33 to 35.82)	3326.3 (2328.4 to 4569.9)	2604.8 (1821.5 to 3621.2)	0.12% (-0.28 to 0.52)
Lao People's Democratic Republic	5855 (3874 to 8387)	7248 (4665 to 10299)	23.81% (3.74 to 45.14)	3701 (2449.3 to 5301.7)	2198.9 (1415 to 3124.4)	-2.54% (-2.82 to -2.26)
Lebanon	4538 (3140 to 6283)	4171 (2877 to 5789)	-8.09% (-22.55 to 7.85)	3636.3 (2515.8 to 5034.5)	1834.3 (1265 to 2545.7)	-2.44% (-2.65 to -2.23)
Lesotho	2050 (1389 to 2829)	1450 (963 to 2036)	-29.25% (-41.4 to -16.14)	2885.3 (1955.9 to 3982.2)	1498.8 (994.8 to 2103.6)	-1.99% (-2.13 to -1.85)
Liberia	1795 (1182 to 2705)	2628 (1706 to 3803)	46.37% (18.87 to 83.05)	2564.5 (1688.6 to 3864)	1337.9 (868.5 to 1936)	-2.14% (-2.37 to -1.92)
Libya	4227 (2881 to 5724)	3424 (2384 to 4688)	-18.99% (-30.27 to -6.59)	3006.2 (2049 to 4071.5)	1174 (817.4 to 1607.4)	-2.7% (-2.99 to -2.41)
Madagascar	14499 (9517 to 20588)	24649 (16448 to 34851)	70% (42.02 to 101.61)	3207.7 (2105.5 to 4554.8)	2280.3 (1521.6 to 3224)	-1.21% (-1.33 to -1.09)

Location	Incident cases in 1990 (95% UI)	Incident cases in 2019 (95% UI)	Relative change (%, 95 CI)	ASIR in 1990 (95% UI)	ASIR in 2019 (95% UI)	EAPC (%, 95 CI)
Malawi	13400 (8935 to 19261)	14676 (9496 to 20671)	9.53% (-7.26 to 31.01)	3494.3 (2330.1 to 5022.8)	2011.5 (1301.6 to 2833.2)	-1.91% (-2.12 to -1.69)
Malaysia	21769 (14550 to 31601)	22523 (14964 to 32396)	3.46% (-13.81 to 21.34)	2845.5 (1901.9 to 4130.6)	1621.8 (1077.5 to 2332.7)	-2.59% (-2.8 to -2.38)
Maldives	280 (183 to 406)	342 (229 to 495)	22.07% (4.23 to 44.96)	3334 (2178.4 to 4829.8)	1744.5 (1167.3 to 2525.1)	-1.67% (-2.04 to -1.31)
Mali	9638 (6343 to 13985)	19918 (12961 to 29776)	106.66% (68.22 to 153.32)	3011.2 (1981.7 to 4369.4)	2409.6 (1567.9 to 3602.1)	-0.76% (-0.79 to -0.73)
Marshall Islands	48 (32 to 67)	39 (26 to 55)	-19.83% (-32.59 to -5.36)	2938.7 (1953.8 to 4082.3)	1740 (1155.3 to 2462.9)	-1.87% (-1.94 to -1.81)
Mauritania	2185 (1458 to 3158)	2470 (1586 to 3613)	13.05% (-7.67 to 39.34)	2797 (1866.8 to 4042.6)	1527.7 (980.9 to 2234.5)	-2.05% (-2.21 to -1.89)
Mexico	86630 (57900 to 126522)	104226 (69593 to 151667)	20.31% (9.4 to 32.12)	2425.7 (1621.2 to 3542.6)	2023 (1350.8 to 2943.8)	-0.03% (-0.58 to 0.53)

Location	Incident cases in 1990 (95% UI)	Incident cases in 2019 (95% UI)	Relative change (%, 95 CI)	ASIR in 1990 (95% UI)	ASIR in 2019 (95% UI)	EAPC (%, 95 CI)
Micronesia (Federated States of)	121 (81 to 175)	75 (51 to 106)	-38.32% (-47.8 to -27.74)	3260.6 (2187 to 4725.3)	1869.6 (1267.1 to 2652.1)	-1.87% (-2.07 to -1.67)
Mongolia	4024 (2769 to 5674)	4023 (2753 to 5625)	-0.02% (-14.32 to 17.81)	4176 (2873.3 to 5888.7)	2681.6 (1835.1 to 3749.6)	-0.5% (-1.14 to 0.14)
Montenegro	446 (331 to 580)	298 (212 to 398)	-33.27% (-43.89 to -23.59)	1818.1 (1347.7 to 2361.8)	1504.1 (1071.6 to 2010.9)	-0.74% (-0.82 to -0.66)
Morocco	28307 (19446 to 38418)	21765 (14943 to 30098)	-23.11% (-35.42 to -11.03)	2659.7 (1827.1 to 3609.7)	1454.4 (998.6 to 2011.3)	-1.95% (-2.07 to -1.83)
Mozambique	14757 (9869 to 21227)	29538 (19281 to 41850)	100.16% (65.79 to 144.21)	3211.1 (2147.5 to 4618.9)	2516.5 (1642.7 to 3565.4)	-0.76% (-0.87 to -0.64)
Myanmar	47936 (31764 to 69050)	40335 (26357 to 58768)	-15.86% (-28.14 to -1.53)	2752.3 (1823.8 to 3964.6)	1775.2 (1160 to 2586.4)	-1.29% (-1.38 to -1.2)
Namibia	1471 (981 to 2096)	2342 (1558 to 3447)	59.21% (32.46 to 92.6)	2588.3 (1725.6 to 3689.7)	2140 (1424.1 to 3150)	-0.45% (-0.65 to -0.26)
Nepal	23945 (16130 to 33478)	19143 (12894 to 26349)	-20.06% (-34.12 to -6.44)	3134.9 (2111.7 to 4382.9)	1320.7 (889.6 to 1817.9)	-3.63% (-3.89 to -3.37)

Location	Incident cases in 1990 (95% UI)	Incident cases in 2019 (95% UI)	Relative change (%, 95 CI)	ASIR in 1990 (95% UI)	ASIR in 2019 (95% UI)	EAPC (%, 95 CI)
Nicaragua	4212 (2798 to 5977)	3834 (2515 to 5381)	-8.96% (-24.94 to 8.26)	2821.2 (1874.4 to 4003.9)	1311 (860 to 1839.7)	-2.82% (-3.23 to -2.4)
Niger	9412 (6146 to 13627)	24701 (16310 to 36184)	162.44% (114 to 227)	3259.5 (2128.3 to 4719.4)	3227.3 (2131.1 to 4727.7)	0% (-0.03 to 0.02)
Nigeria	113023 (75923 to 160747)	214544 (146447 to 306046)	89.82% (81.47 to 99.53)	3336.5 (2241.3 to 4745.4)	2492.6 (1701.4 to 3555.6)	-1.26% (-1.58 to -0.94)
North Macedonia	1572 (1154 to 2042)	989 (720 to 1301)	-37.07% (-45.88 to -27.49)	1975.7 (1451 to 2566.7)	1257.9 (915.4 to 1654.1)	-1.59% (-1.73 to -1.45)
Pakistan	165300 (115305 to 226379)	249343 (173783 to 343768)	50.84% (38.54 to 63.75)	4551.8 (3175.1 to 6233.7)	2867 (1998.2 to 3952.7)	-1.67% (-1.81 to -1.54)
Papua New Guinea	5079 (3438 to 7021)	11347 (7643 to 16278)	123.43% (90.12 to 160.54)	3058.5 (2070.6 to 4228.4)	2713.6 (1827.6 to 3892.5)	-0.39% (-0.45 to -0.33)
Paraguay	4472 (3095 to 6163)	4779 (3221 to 6598)	6.85% (-9.16 to 25.09)	2838.5 (1964.7 to 3911.8)	1571.5 (1059.3 to 2169.9)	-1.95% (-2.01 to -1.89)

Location	Incident cases in 1990 (95% UI)	Incident cases in 2019 (95% UI)	Relative change (%, 95 CI)	ASIR in 1990 (95% UI)	ASIR in 2019 (95% UI)	EAPC (%, 95 CI)
Peru	23809 (15859 to 33127)	22439 (15023 to 31209)	-5.76% (-20.91 to 12.48)	2643.9 (1761.1 to 3678.6)	1582.3 (1059.4 to 2200.7)	-1.68% (-1.75 to -1.61)
Philippines	86983 (58271 to 123653)	116294 (76449 to 165378)	33.7% (27.89 to 39.23)	3371.9 (2258.9 to 4793.4)	2484.2 (1633.1 to 3532.7)	-1.2% (-1.28 to -1.13)
Republic of Moldova	5952 (4201 to 8076)	2743 (1851 to 3705)	-53.92% (-59.54 to -47.64)	3183.3 (2246.9 to 4319.5)	2080.9 (1404.5 to 2811.1)	-1.01% (-1.26 to -0.76)
Russian Federation	134184 (90804 to 180605)	129023 (87830 to 172468)	-3.85% (-6.2 to -1.33)	2215.9 (1499.5 to 2982.4)	2865.6 (1950.7 to 3830.6)	2.15% (1.68 to 2.63)
Rwanda	10857 (7295 to 15281)	10634 (7161 to 15109)	-2.05% (-18.48 to 16.55)	4001.8 (2688.8 to 5632.5)	2118.9 (1426.8 to 3010.3)	-2.37% (-2.51 to -2.23)
Saint Lucia	103 (69 to 147)	65 (44 to 92)	-36.79% (-46.13 to -26.07)	1760.5 (1179.6 to 2512.5)	945.9 (637.8 to 1335.6)	-2.2% (-2.31 to -2.09)
Saint Vincent and the Grenadines	88 (58 to 125)	54 (36 to 76)	-38.8% (-48.1 to -26.88)	1925.5 (1277.9 to 2729.2)	1329.2 (882.8 to 1863.7)	-1.27% (-1.38 to -1.15)
Samoa	126 (85 to 177)	78 (53 to 108)	-38.51% (-47.81 to -26.5)	2122.3 (1429.2 to 2984.4)	1012.9 (688.8 to 1410.4)	-2.61% (-2.83 to -2.4)

Location	Incident cases in 1990 (95% UI)	Incident cases in 2019 (95% UI)	Relative change (%, 95 CI)	ASIR in 1990 (95% UI)	ASIR in 2019 (95% UI)	EAPC (%, 95 CI)
Senegal	7400 (4961 to 10928)	10507 (6709 to 15834)	41.99% (14.53 to 72.65)	2590.3 (1736.6 to 3825.3)	1684 (1075.3 to 2537.8)	-1.25% (-1.37 to -1.14)
Serbia	4386 (3081 to 5956)	2425 (1699 to 3330)	-44.72% (-52.53 to -36.51)	1326.4 (931.8 to 1801.1)	880.6 (616.8 to 1209.3)	-1.15% (-1.38 to -0.92)
Sierra Leone	3629 (2378 to 5416)	6152 (3974 to 9122)	69.52% (35.93 to 111.82)	2427.9 (1591.3 to 3623.7)	1679.4 (1084.9 to 2490.3)	-1.21% (-1.32 to -1.1)
Solomon Islands	464 (318 to 660)	721 (487 to 996)	55.54% (31.53 to 80.3)	3847.1 (2636 to 5476.3)	2820.1 (1905.1 to 3895.8)	-1.11% (-1.18 to -1.03)
Somalia	9063 (6007 to 12762)	25698 (16758 to 36605)	183.54% (137.05 to 240.63)	4187.3 (2775.2 to 5895.8)	3618.4 (2359.5 to 5153.9)	-0.43% (-0.47 to -0.4)
South Africa	31864 (21948 to 44597)	48205 (33373 to 67171)	51.28% (40.16 to 60.9)	1893.8 (1304.5 to 2650.5)	1860.2 (1287.8 to 2592)	0.06% (0 to 0.13)
South Sudan	7704 (5042 to 11164)	8866 (5990 to 12709)	15.08% (-4.41 to 36.48)	3523.8 (2306.3 to 5106.1)	3259.2 (2202.1 to 4671.7)	-0.24% (-0.28 to -0.2)
Sri Lanka	15738 (10162 to 22829)	12945 (8434 to 19199)	-17.75% (-30.37 to -2.38)	2070.3 (1336.8 to 3003.1)	1619.3 (1055 to 2401.6)	-0.55% (-0.76 to -0.35)

Location	Incident cases in 1990 (95% UI)	Incident cases in 2019 (95% UI)	Relative change (%, 95 CI)	ASIR in 1990 (95% UI)	ASIR in 2019 (95% UI)	EAPC (%, 95 CI)
Sudan	36020 (25416 to 49120)	42939 (29991 to 57496)	19.21% (1.13 to 37.41)	4769.8 (3365.7 to 6504.5)	2441.6 (1705.4 to 3269.4)	-2.5% (-2.76 to -2.23)
Suriname	380 (252 to 545)	342 (223 to 492)	-10.03% (-24.04 to 5.8)	2305.6 (1530.8 to 3308.2)	1602.4 (1045 to 2306.2)	-1.07% (-1.18 to -0.95)
Syrian Arab Republic	17769 (12520 to 24791)	5994 (4143 to 8153)	-66.27% (-71.03 to -60.63)	3881.6 (2735 to 5415.6)	1650.2 (1140.7 to 2244.7)	-2.27% (-2.64 to -1.9)
Tajikistan	11219 (7668 to 15494)	17846 (12379 to 24804)	59.07% (35.08 to 82.5)	4816.9 (3292.2 to 6652.5)	4083.1 (2832.4 to 5675.2)	-0.55% (-0.87 to -0.23)
Thailand	38774 (25583 to 55235)	25065 (16814 to 35617)	-35.36% (-44.78 to -23.6)	1424.3 (939.8 to 2029)	1032.4 (692.5 to 1467)	-1.09% (-1.17 to -1.02)
Timor-Leste	1448 (961 to 2087)	1609 (1047 to 2284)	11.11% (-6.83 to 29.48)	4253.7 (2822.9 to 6129.6)	2982.2 (1940.7 to 4233.2)	-1.54% (-1.82 to -1.26)
Togo	3643 (2363 to 5322)	5550 (3698 to 8211)	52.35% (23.97 to 88.26)	2482.8 (1610.8 to 3627.4)	1659.6 (1105.8 to 2455.3)	-1.09% (-1.24 to -0.93)

Location	Incident cases in 1990 (95% UI)	Incident cases in 2019 (95% UI)	Relative change (%, 95 CI)	ASIR in 1990 (95% UI)	ASIR in 2019 (95% UI)	EAPC (%, 95 CI)
Tonga	91 (62 to 129)	89 (60 to 127)	-2.1% (-18.28 to 15.12)	2652.5 (1808.4 to 3780)	2389.2 (1601.1 to 3403.8)	-0.3% (-0.33 to -0.27)
Tunisia	9286 (6240 to 12723)	6989 (4876 to 9490)	-24.73% (-35.85 to -12.08)	2630.2 (1767.5 to 3603.8)	1504.5 (1049.7 to 2042.8)	-1.95% (-2.07 to -1.82)
Turkey	83662 (59086 to 117822)	74493 (52866 to 102360)	-10.96% (-21.42 to 1.67)	3467.2 (2448.7 to 4882.8)	2207.1 (1566.3 to 3032.8)	-1.24% (-1.36 to -1.11)
Turkmenistan	8315 (5940 to 11450)	6965 (4802 to 9641)	-16.23% (-29.41 to -1.83)	4861.3 (3473.1 to 6694.5)	3392.8 (2339 to 4695.9)	-0.97% (-1.34 to -0.59)
Tuvalu	9 (6 to 13)	8 (5 to 12)	-11.29% (-24.03 to 4.39)	2362.9 (1578.9 to 3349.3)	1802.5 (1219.6 to 2579.7)	-0.93% (-0.96 to -0.9)
Uganda	28284 (19259 to 39851)	48971 (32172 to 69717)	73.14% (47.34 to 107.32)	4194.7 (2856.2 to 5910.1)	2984 (1960.4 to 4248.1)	-1.26% (-1.37 to -1.15)
Ukraine	45153 (29784 to 60873)	33961 (22855 to 45753)	-24.79% (-34.97 to -12.72)	2293.7 (1513 to 3092.2)	2362.5 (1589.9 to 3182.8)	1.27% (0.74 to 1.81)

Location	Incident cases in 1990 (95% UI)	Incident cases in 2019 (95% UI)	Relative change (%, 95 CI)	ASIR in 1990 (95% UI)	ASIR in 2019 (95% UI)	EAPC (%, 95 CI)
United Republic of Tanzania	37575 (25534 to 53912)	60777 (39339 to 87976)	61.75% (36.99 to 91.4)	3658.4 (2486.1 to 5249)	2639 (1708.2 to 3820.1)	-1.06% (-1.12 to -0.99)
Uzbekistan	38878 (27100 to 52738)	37666 (26101 to 53002)	-3.12% (-16.52 to 11.34)	4130.1 (2878.9 to 5602.4)	2439.4 (1690.4 to 3432.7)	-1.64% (-2.01 to -1.28)
Vanuatu	191 (126 to 271)	238 (160 to 336)	24.67% (8.03 to 47.78)	3125.3 (2057.8 to 4435)	1854.5 (1242.7 to 2612.9)	-2.01% (-2.15 to -1.87)
Venezuela (Bolivarian Republic of)	17828 (11753 to 25488)	16101 (10594 to 23163)	-9.69% (-25.74 to 8.69)	2162.9 (1425.8 to 3092.2)	1476.6 (971.6 to 2124.2)	-0.97% (-1.15 to -0.78)
Viet Nam	75593 (50282 to 110597)	66939 (45107 to 96574)	-11.45% (-25.03 to 4.13)	2400.8 (1596.9 to 3512.5)	1541.8 (1038.9 to 2224.3)	-1.06% (-1.23 to -0.9)
Yemen	24596 (16934 to 33638)	31686 (22152 to 42805)	28.83% (7.97 to 49.94)	5342.2 (3678.1 to 7306.4)	2500.7 (1748.3 to 3378.2)	-2.55% (-2.8 to -2.3)
Zambia	10946 (7136 to 15687)	17250 (11210 to 25109)	57.59% (29.69 to 90.36)	3634.2 (2369.4 to 5208.3)	2204.3 (1432.5 to 3208.5)	-1.67% (-1.84 to -1.49)

Location	Incident cases in 1990 (95% UI)	Incident cases in 2019 (95% UI)	Relative change (%, 95 CI)	ASIR in 1990 (95% UI)	ASIR in 2019 (95% UI)	EAPC (%, 95 CI)
Zimbabwe	11689 (7952 to 16124)	13966 (9195 to 19728)	19.49% (1.06 to 41.14)	2983.9 (2029.9 to 4116.1)	2224.2 (1464.3 to 3141.8)	-0.58% (-0.75 to -0.42)

ASIR – age-standardized incidence rate (per 100,000 population), CI – confidence interval, EAPC – estimated annual percentage change, MSMI – maternal sepsis and other maternal infections, UI – uncertain interval.

Table S11. Incidence of MSMI in 131 low- and middle-income countries and territories with EAPC in age group of 30-34 years from 1990 and 2019.

Location	Incident cases in 1990 (95% UI)	Incident cases in 2019 (95% UI)	Relative change (%, 95 CI)	ASIR in 1990 (95% UI)	ASIR in 2019 (95% UI)	EAPC (%, 95 CI)
Afghanistan	5816 (3528 to 9167)	20272 (11734 to 31685)	248.54% (178.08 to 343.94)	2677.2 (1623.8 to 4219.8)	1964.9 (1137.4 to 3071.3)	-1.02% (-1.16 to -0.88)
Albania	1143 (638 to 1841)	481 (277 to 793)	-57.94% (-68.02 to -43.67)	835.3 (466.4 to 1345)	544.8 (313.7 to 899)	-1.62% (-2.08 to -1.16)
Algeria	14165 (8221 to 22931)	28937 (17141 to 46235)	104.29% (61.27 to 163.48)	1837.1 (1066.2 to 2974.1)	1545.2 (915.3 to 2468.9)	-0.01% (-0.48 to 0.46)
American Samoa	30 (19 to 47)	19 (12 to 30)	-37.85% (-48.98 to -24.38)	1779.4 (1094 to 2729)	1213.7 (738.1 to 1900.1)	-1.38% (-1.43 to -1.34)
Angola	9074 (5787 to 13996)	21404 (13047 to 32311)	135.88% (86.29 to 198.03)	2829.3 (1804.4 to 4364)	2142.5 (1305.9 to 3234.2)	-1% (-1.06 to -0.94)
Armenia	1102 (667 to 1758)	757 (440 to 1227)	-31.29% (-44.8 to -13.88)	688.9 (416.9 to 1098.5)	553 (321.3 to 896.3)	0.1% (-0.52 to 0.74)
Azerbaijan	3092 (1845 to 4884)	2009 (1229 to 3162)	-35.02% (-47.93 to -19.74)	975.3 (582.1 to 1541)	407.9 (249.6 to 642.1)	-2.16% (-2.55 to -1.77)

Location	Incident cases in 1990 (95% UI)	Incident cases in 2019 (95% UI)	Relative change (%, 95 CI)	ASIR in 1990 (95% UI)	ASIR in 2019 (95% UI)	EAPC (%, 95 CI)
Bangladesh	39686 (25387 to 60654)	32071 (19965 to 49423)	-19.19% (-36.62 to -1.2)	1196.7 (765.5 to 1829)	486.9 (303.1 to 750.3)	-3.34% (-3.48 to -3.2)
Belarus	4094 (2544 to 5906)	7118 (4439 to 10073)	73.84% (50.08 to 106.42)	930 (577.9 to 1341.4)	1857.7 (1158.5 to 2629)	4.95% (3.99 to 5.91)
Belize	101 (63 to 156)	143 (87 to 215)	41.5% (15.8 to 70.56)	1765.9 (1099.2 to 2738.9)	894.5 (546.2 to 1347.6)	-2% (-2.19 to -1.8)
Benin	3894 (2412 to 5888)	7492 (4761 to 11199)	92.4% (53.36 to 146.69)	2441.6 (1512.3 to 3692)	1853.8 (1178.1 to 2771.1)	-0.7% (-0.78 to -0.63)
Bhutan	292 (185 to 453)	240 (143 to 377)	-17.82% (-33.75 to 3.31)	1582.1 (999.9 to 2450.6)	742.8 (442.7 to 1165.2)	-2.82% (-3.01 to -2.63)
Bolivia (Plurinational State of)	4889 (2998 to 7728)	7440 (4584 to 11609)	52.16% (23.2 to 87.43)	2299.3 (1409.9 to 3634.3)	1667.4 (1027.3 to 2601.9)	-1.07% (-1.21 to -0.94)
Bosnia and Herzegovina	785 (435 to 1281)	639 (359 to 1036)	-18.61% (-38.09 to 8.07)	422.5 (233.9 to 689.3)	591.7 (332.4 to 958.9)	1.06% (0.67 to 1.46)
Botswana	647 (394 to 974)	1158 (758 to 1740)	79.07% (46.8 to 125.47)	1469.5 (896.2 to 2212.3)	1049.2 (686.8 to 1576.3)	-1% (-1.1 to -0.89)

Location	Incident cases in 1990 (95% UI)	Incident cases in 2019 (95% UI)	Relative change (%, 95 CI)	ASIR in 1990 (95% UI)	ASIR in 2019 (95% UI)	EAPC (%, 95 CI)
Brazil	61313 (39885 to 88640)	104808 (65766 to 158257)	70.94% (49.33 to 90.88)	1078.5 (701.6 to 1559.2)	1172.8 (735.9 to 1771)	0.94% (0.54 to 1.35)
Bulgaria	586 (344 to 946)	1180 (660 to 1898)	101.32% (50.29 to 161.54)	198.5 (116.4 to 320.5)	509.7 (285 to 819.9)	4.91% (4.36 to 5.47)
Burkina Faso	6996 (4356 to 10725)	14645 (8914 to 22280)	109.34% (65.84 to 176.01)	2412.6 (1502.3 to 3698.5)	1914.4 (1165.2 to 2912.5)	-0.88% (-0.94 to -0.83)
Burundi	5692 (3546 to 8851)	10146 (6347 to 15362)	78.26% (44.54 to 119.48)	3010.5 (1875.7 to 4681.2)	2553.4 (1597.4 to 3865.8)	-0.45% (-0.5 to -0.4)
Cabo Verde	159 (100 to 241)	204 (128 to 305)	28.33% (2.46 to 58.64)	1418.6 (894.7 to 2145.3)	830.7 (520.3 to 1241.3)	-1.81% (-1.94 to -1.69)
Cambodia	10017 (6107 to 15576)	8543 (5104 to 13120)	-14.71% (-29.46 to 2.41)	2743.6 (1672.8 to 4266.4)	1209.4 (722.5 to 1857.4)	-3% (-3.17 to -2.82)
Cameroon	6573 (4177 to 10115)	16701 (10662 to 25633)	154.1% (107.02 to 214.49)	2020.2 (1283.9 to 3109)	1554.3 (992.3 to 2385.6)	-0.67% (-0.84 to -0.49)
Central African Republic	2166 (1364 to 3346)	3367 (2098 to 5157)	55.45% (25.44 to 93.46)	2405.3 (1514.7 to 3716)	1877.9 (1170.2 to 2876.3)	-0.91% (-0.96 to -0.86)

Location	Incident cases in 1990 (95% UI)	Incident cases in 2019 (95% UI)	Relative change (%, 95 CI)	ASIR in 1990 (95% UI)	ASIR in 2019 (95% UI)	EAPC (%, 95 CI)
Chad	4590 (2834 to 6964)	11317 (7069 to 17431)	146.55% (97.3 to 202.41)	2555.6 (1578.1 to 3877.1)	2417.3 (1509.8 to 3723)	-0.23% (-0.29 to -0.16)
China	291456 (181636 to 445307)	324105 (202879 to 496897)	11.2% (5.21 to 17.59)	688.1 (428.8 to 1051.3)	508 (318 to 778.8)	0.86% (0.3 to 1.43)
Colombia	15776 (9812 to 25321)	15723 (9695 to 24772)	-0.33% (-20.66 to 24.01)	1230.3 (765.2 to 1974.6)	868.9 (535.8 to 1369)	-1.32% (-1.39 to -1.24)
Comoros	424 (260 to 666)	426 (261 to 638)	0.52% (-18.74 to 28.26)	3111.6 (1913.2 to 4895.4)	1641.2 (1005.8 to 2457.6)	-2.17% (-2.26 to -2.09)
Congo	1555 (1006 to 2401)	3025 (1878 to 4756)	94.56% (55.81 to 140.86)	2094.7 (1355.4 to 3235)	1491.4 (925.9 to 2345.1)	-0.85% (-0.96 to -0.73)
Costa Rica	1403 (856 to 2216)	1460 (887 to 2269)	4.07% (-19.64 to 32.85)	1163.4 (709.5 to 1837.7)	706 (428.7 to 1096.9)	-1.55% (-1.85 to -1.25)
Côte d'Ivoire	8314 (5128 to 12670)	15174 (9413 to 23292)	82.51% (44.14 to 126.01)	2163.2 (1334.1 to 3296.4)	1533.8 (951.4 to 2354.4)	-1.24% (-1.3 to -1.17)
Cuba	2157 (1329 to 3299)	2353 (1458 to 3682)	9.12% (-12.08 to 36.69)	556.6 (343.1 to 851.6)	604.9 (374.9 to 946.4)	0.61% (0.43 to 0.8)

Location	Incident cases in 1990 (95% UI)	Incident cases in 2019 (95% UI)	Relative change (%, 95 CI)	ASIR in 1990 (95% UI)	ASIR in 2019 (95% UI)	EAPC (%, 95 CI)
Democratic People's Republic of Korea	12582 (7773 to 20033)	6981 (4205 to 10811)	-44.52% (-54.47 to -31.12)	1699.4 (1049.9 to 2705.8)	743.1 (447.6 to 1150.7)	-2.66% (-2.77 to -2.55)
Democratic Republic of the Congo	35644 (22962 to 54281)	60847 (37547 to 93232)	70.71% (31.23 to 117.13)	3031.2 (1952.7 to 4616.1)	2131.7 (1315.4 to 3266.3)	-1.1% (-1.22 to -0.99)
Djibouti	374 (231 to 580)	1026 (645 to 1546)	173.92% (119.07 to 251.94)	2740.9 (1688.7 to 4243.8)	1902.7 (1195.9 to 2868.4)	-1.37% (-1.45 to -1.29)
Dominica	28 (17 to 44)	21 (13 to 33)	-24.55% (-39.78 to -6.35)	1250.9 (752.9 to 2002.6)	824.4 (502.9 to 1298)	-1.58% (-1.63 to -1.54)
Dominican Republic	3039 (1901 to 4594)	3418 (2127 to 5260)	12.48% (-12.1 to 37.4)	1172.4 (733.4 to 1772.4)	820.6 (510.7 to 1262.8)	-1.32% (-1.47 to -1.16)
Ecuador	5677 (3500 to 8973)	7600 (4743 to 11722)	33.86% (7.06 to 66.63)	1610.8 (993 to 2546)	1154.7 (720.6 to 1780.9)	-1.06% (-1.22 to -0.89)
Egypt	29173 (16855 to 46752)	36324 (21385 to 56863)	24.51% (-0.52 to 58.82)	1618 (934.8 to 2593)	958.5 (564.3 to 1500.5)	-1.28% (-1.5 to -1.06)
El Salvador	2060 (1270 to 3208)	1716 (1043 to 2685)	-16.71% (-33.75 to 2.91)	1201.5 (740.6 to 1871.3)	725.3 (440.8 to 1134.9)	-1.88% (-2.13 to -1.62)

Location	Incident cases in 1990 (95% UI)	Incident cases in 2019 (95% UI)	Relative change (%, 95 CI)	ASIR in 1990 (95% UI)	ASIR in 2019 (95% UI)	EAPC (%, 95 CI)
Equatorial Guinea	377 (247 to 582)	755 (463 to 1146)	100.47% (65.48 to 152.93)	2873.3 (1888.3 to 4439.1)	1560.2 (957.5 to 2368.8)	-2.22% (-2.28 to -2.16)
Eritrea	2483 (1527 to 3760)	4047 (2453 to 6197)	62.98% (32.84 to 104.08)	2617.7 (1609.7 to 3963.3)	1731.5 (1049.5 to 2651.3)	-1.38% (-1.42 to -1.33)
Eswatini	504 (320 to 754)	615 (399 to 948)	21.96% (-0.35 to 47.66)	1970.4 (1252.6 to 2947.8)	1261.6 (818.3 to 1946.1)	-1.4% (-1.44 to -1.37)
Ethiopia	49028 (31370 to 73072)	74042 (47342 to 112779)	51.02% (37 to 72.51)	3145.4 (2012.6 to 4687.9)	2077.5 (1328.3 to 3164.4)	-1.53% (-1.59 to -1.46)
Fiji	300 (189 to 451)	364 (225 to 551)	21.28% (-4.7 to 47.29)	1032.3 (649.3 to 1552.4)	1078.2 (665.8 to 1632.3)	0.17% (0.09 to 0.25)
Gabon	660 (412 to 1023)	890 (561 to 1329)	34.72% (10.35 to 64.95)	2260.8 (1410 to 3501.9)	1279.5 (806.7 to 1911.7)	-1.8% (-1.88 to -1.72)
Gambia	796 (501 to 1227)	1332 (829 to 2108)	67.45% (34.6 to 111.74)	2414.3 (1520.9 to 3724.7)	1646 (1024 to 2605.3)	-1.33% (-1.37 to -1.3)
Georgia	1298 (783 to 2052)	1103 (670 to 1781)	-15.02% (-31.51 to 5.52)	575.5 (347.1 to 910)	829.9 (503.9 to 1340.4)	2.58% (2.11 to 3.05)

Location	Incident cases in 1990 (95% UI)	Incident cases in 2019 (95% UI)	Relative change (%, 95 CI)	ASIR in 1990 (95% UI)	ASIR in 2019 (95% UI)	EAPC (%, 95 CI)
Ghana	9477 (5884 to 14447)	16756 (10628 to 25486)	76.8% (40.92 to 120.21)	1865.9 (1158.5 to 2844.4)	1298.8 (823.8 to 1975.5)	-1.14% (-1.25 to -1.03)
Global	2350276 (1530057 to 3494310)	3034188 (1969911 to 4504000)	29.1% (26.29 to 31.85)	1235.9 (804.6 to 1837.5)	1016.3 (659.8 to 1508.6)	-0.32% (-0.47 to -0.18)
Grenada	45 (28 to 71)	35 (21 to 56)	-22.41% (-37.73 to -2.81)	1556.5 (947.6 to 2424.4)	978.8 (591.6 to 1549.8)	-1.45% (-1.66 to -1.24)
Guatemala	6309 (3965 to 9716)	7028 (4286 to 11290)	11.4% (-14.43 to 39.67)	2568.3 (1614.3 to 3955.3)	996.6 (607.7 to 1601)	-3.74% (-4.12 to -3.37)
Guinea	4793 (2986 to 7432)	7272 (4664 to 11156)	51.74% (18.85 to 94.57)	2286.5 (1424.5 to 3545.5)	1606.7 (1030.4 to 2464.7)	-1.3% (-1.38 to -1.23)
Guinea-Bissau	724 (462 to 1068)	1156 (715 to 1761)	59.73% (30.26 to 98.85)	2162.8 (1380.9 to 3193.2)	1527.5 (944.7 to 2326.7)	-1.22% (-1.25 to -1.19)
Guyana	269 (165 to 413)	230 (139 to 349)	-14.64% (-30.24 to 5.95)	935.5 (573.1 to 1434)	855 (517.9 to 1299.3)	-0.18% (-0.27 to -0.08)

Location	Incident cases in 1990 (95% UI)	Incident cases in 2019 (95% UI)	Relative change (%, 95 CI)	ASIR in 1990 (95% UI)	ASIR in 2019 (95% UI)	EAPC (%, 95 CI)
Haiti	5457 (3314 to 8453)	8421 (5169 to 13107)	54.31% (26.2 to 88.62)	2458.2 (1492.8 to 3807.6)	1544.1 (947.9 to 2403.5)	-1.75% (-1.81 to -1.68)
Honduras	2879 (1720 to 4442)	3948 (2419 to 5966)	37.14% (10.69 to 74.1)	2020.2 (1206.8 to 3117.5)	1039.7 (637 to 1571.3)	-2.45% (-2.67 to -2.22)
India	412923 (267063 to 621122)	433622 (273508 to 663612)	5.01% (-2.29 to 11.47)	1451.7 (938.9 to 2183.7)	799.2 (504.1 to 1223)	-2.39% (-2.64 to -2.15)
Indonesia	93378 (59020 to 141153)	109959 (69625 to 165588)	17.76% (11.25 to 25.83)	1349.6 (853 to 2040.1)	1097.5 (694.9 to 1652.7)	-0.64% (-0.79 to -0.49)
Iran (Islamic Republic of)	29337 (17887 to 46200)	37362 (23050 to 58065)	27.35% (18.69 to 38.49)	1628.1 (992.7 to 2564)	855.3 (527.6 to 1329.2)	-1.32% (-1.97 to -0.67)
Iraq	11835 (6899 to 19036)	19242 (11545 to 29909)	62.59% (30.54 to 103.92)	2309.5 (1346.3 to 3714.7)	1191.6 (714.9 to 1852.1)	-2.27% (-2.32 to -2.21)
Jamaica	901 (570 to 1423)	783 (477 to 1221)	-13.15% (-28.4 to 5.68)	1063.7 (672.5 to 1678.9)	677.6 (412.7 to 1056.3)	-1.74% (-1.87 to -1.62)
Jordan	1649 (941 to 2623)	3596 (2388 to 5364)	118.11% (69.33 to 196.68)	1693.3 (966.1 to 2693.3)	934.1 (620.2 to 1393.2)	-2.11% (-2.22 to -2)

Location	Incident cases in 1990 (95% UI)	Incident cases in 2019 (95% UI)	Relative change (%, 95 CI)	ASIR in 1990 (95% UI)	ASIR in 2019 (95% UI)	EAPC (%, 95 CI)
Kazakhstan	7050 (4293 to 11255)	9312 (5462 to 14902)	32.09% (6.76 to 62.62)	1022 (622.4 to 1631.8)	1147.6 (673.1 to 1836.6)	1.61% (1.14 to 2.08)
Kenya	17451 (11186 to 26260)	26931 (17451 to 40266)	54.32% (49.33 to 59.3)	2639.1 (1691.6 to 3971.3)	1440.5 (933.4 to 2153.8)	-1.97% (-2.12 to -1.83)
Kiribati	49 (29 to 73)	72 (44 to 111)	47.92% (17.39 to 83.01)	1778.7 (1053.7 to 2673.7)	1568.3 (965.7 to 2426.2)	-0.5% (-0.59 to -0.41)
Kyrgyzstan	2503 (1488 to 4001)	3391 (2127 to 5419)	35.46% (11.84 to 64.02)	1472.2 (875.1 to 2352.8)	1245.1 (781 to 1989.8)	0.36% (-0.12 to 0.84)
Lao People's Democratic Republic	2934 (1808 to 4585)	3547 (2119 to 5406)	20.89% (-2.98 to 47.05)	2156.8 (1328.8 to 3370.8)	1202.9 (718.6 to 1833.6)	-2.75% (-3.01 to -2.5)
Lebanon	1635 (956 to 2629)	2148 (1249 to 3459)	31.39% (3.78 to 66.88)	1605.9 (938.8 to 2582.3)	870.4 (506.1 to 1401.8)	-1.86% (-1.95 to -1.77)
Lesotho	995 (653 to 1512)	896 (559 to 1361)	-9.9% (-28.27 to 14.37)	1758.1 (1154.5 to 2672.8)	1067.1 (665.7 to 1620.3)	-1.64% (-1.73 to -1.54)
Liberia	1347 (827 to 2061)	2268 (1461 to 3376)	68.4% (35.78 to 113.93)	2123.8 (1303.2 to 3249.8)	1330 (856.7 to 1979.3)	-1.65% (-1.75 to -1.56)

Location	Incident cases in 1990 (95% UI)	Incident cases in 2019 (95% UI)	Relative change (%, 95 CI)	ASIR in 1990 (95% UI)	ASIR in 2019 (95% UI)	EAPC (%, 95 CI)
Libya	2106 (1228 to 3210)	2788 (1621 to 4540)	32.4% (4.91 to 65.1)	1906.5 (1112.1 to 2906.4)	931.9 (541.8 to 1517.7)	-2.06% (-2.28 to -1.84)
Madagascar	8342 (5020 to 12751)	14148 (8773 to 21611)	69.6% (35.46 to 117.53)	2212.9 (1331.7 to 3382.4)	1559.3 (966.9 to 2381.9)	-1.32% (-1.4 to -1.24)
Malawi	6903 (4139 to 10751)	8877 (5289 to 13757)	28.59% (3.71 to 59.94)	2404.4 (1441.5 to 3744.6)	1385 (825.2 to 2146.4)	-2.04% (-2.23 to -1.85)
Malaysia	12978 (7938 to 20249)	17845 (10813 to 28085)	37.5% (10.61 to 69.5)	1908.1 (1167 to 2977.1)	1353.3 (820 to 2129.9)	-1.48% (-1.59 to -1.38)
Maldives	134 (82 to 210)	234 (143 to 351)	73.84% (45.9 to 111.36)	2183.4 (1332.1 to 3404)	1097.2 (670.6 to 1647.9)	-2.3% (-2.66 to -1.94)
Mali	7017 (4430 to 10543)	13108 (8239 to 19936)	86.8% (49.91 to 130.21)	2585 (1632.1 to 3883.7)	1978.1 (1243.3 to 3008.3)	-0.92% (-0.95 to -0.89)
Marshall Islands	20 (12 to 30)	24 (15 to 37)	21.54% (-1.46 to 51.89)	1420.8 (851.6 to 2168.2)	1102.4 (668.3 to 1699.5)	-0.52% (-0.71 to -0.33)
Mauritania	1605 (1019 to 2451)	2307 (1420 to 3489)	43.77% (16.26 to 78.41)	2466.9 (1566.7 to 3767)	1689.5 (1039.4 to 2554.6)	-1.29% (-1.39 to -1.2)

Location	Incident cases in 1990 (95% UI)	Incident cases in 2019 (95% UI)	Relative change (%, 95 CI)	ASIR in 1990 (95% UI)	ASIR in 2019 (95% UI)	EAPC (%, 95 CI)
Mexico	48120 (30398 to 75683)	67188 (41344 to 103548)	39.63% (28.76 to 54.1)	1618.3 (1022.3 to 2545.3)	1372 (844.3 to 2114.5)	0.06% (-0.64 to 0.75)
Micronesia (Federated States of)	65 (41 to 102)	43 (27 to 67)	-34.43% (-46.59 to -18.32)	1933.2 (1218.9 to 3024)	1244.5 (792.7 to 1962)	-1.63% (-1.76 to -1.49)
Mongolia	1381 (855 to 2265)	2366 (1386 to 3803)	71.3% (38.79 to 115.6)	1834.5 (1135.4 to 3009)	1455.8 (852.9 to 2340.2)	0.57% (-0.16 to 1.29)
Montenegro	127 (73 to 202)	134 (77 to 225)	6.18% (-20.36 to 37.34)	530 (305.3 to 847.3)	633.8 (364.3 to 1059)	0.81% (0.71 to 0.9)
Morocco	14243 (8615 to 22789)	14264 (8502 to 22660)	0.14% (-22.01 to 24.72)	1623.7 (982.1 to 2597.9)	994.7 (592.9 to 1580.3)	-1.55% (-1.62 to -1.48)
Mozambique	9574 (5902 to 14649)	15339 (9087 to 23599)	60.22% (25.2 to 104.08)	2267.3 (1397.8 to 3469.2)	1716.2 (1016.7 to 2640.2)	-0.83% (-0.96 to -0.69)
Myanmar	26006 (15540 to 41101)	26392 (15913 to 40645)	1.49% (-16.65 to 23.79)	1757.3 (1050.1 to 2777.3)	1208.5 (728.7 to 1861.1)	-1.45% (-1.51 to -1.4)
Namibia	899 (546 to 1342)	1255 (795 to 1929)	39.59% (11.5 to 76.24)	1999.6 (1214.1 to 2983.5)	1317.4 (833.9 to 2023.9)	-1.12% (-1.26 to -0.98)

Location	Incident cases in 1990 (95% UI)	Incident cases in 2019 (95% UI)	Relative change (%, 95 CI)	ASIR in 1990 (95% UI)	ASIR in 2019 (95% UI)	EAPC (%, 95 CI)
Nepal	10142 (6183 to 15597)	6416 (3834 to 9958)	-36.74% (-51.89 to -20.1)	1610.5 (981.9 to 2476.8)	508.9 (304.1 to 789.8)	-4.66% (-5.06 to -4.26)
Nicaragua	1788 (1144 to 2692)	2112 (1291 to 3213)	18.09% (-5.65 to 46.05)	1474.1 (943.4 to 2219.3)	799.7 (489 to 1217)	-2.05% (-2.42 to -1.69)
Niger	6672 (4161 to 10160)	15458 (9864 to 22933)	131.7% (86.69 to 186.33)	2728.1 (1701.6 to 4154.5)	2672.7 (1705.5 to 3965)	-0.07% (-0.13 to -0.01)
Nigeria	76996 (50731 to 111408)	157907 (105144 to 229437)	105.09% (96.56 to 116.81)	2866.9 (1888.9 to 4148.2)	2241 (1492.2 to 3256.2)	-1.09% (-1.35 to -0.83)
North Macedonia	320 (185 to 512)	411 (239 to 675)	28.35% (-0.47 to 72.05)	411.6 (238 to 657.9)	501 (291.9 to 822.4)	0.91% (0.68 to 1.13)
Pakistan	87079 (55905 to 128840)	112621 (73572 to 168314)	29.33% (15.76 to 43.06)	2936.1 (1885 to 4344.2)	1507.2 (984.6 to 2252.6)	-2.41% (-2.58 to -2.24)
Papua New Guinea	2705 (1674 to 4046)	6752 (4044 to 10278)	149.59% (98.8 to 207.14)	2010.9 (1244.5 to 3007.8)	1818.3 (1089.1 to 2767.9)	-0.39% (-0.41 to -0.37)
Paraguay	2198 (1350 to 3328)	2691 (1618 to 4132)	22.39% (0.02 to 54.16)	1645.2 (1010.2 to 2490.7)	963.2 (579 to 1479.1)	-1.95% (-1.99 to -1.91)

Location	Incident cases in 1990 (95% UI)	Incident cases in 2019 (95% UI)	Relative change (%, 95 CI)	ASIR in 1990 (95% UI)	ASIR in 2019 (95% UI)	EAPC (%, 95 CI)
Peru	12658 (7960 to 19725)	14050 (8611 to 21266)	11% (-10.78 to 38.59)	1692.6 (1064.3 to 2637.5)	1059.5 (649.3 to 1603.7)	-1.7% (-1.78 to -1.61)
Philippines	46298 (28903 to 69959)	64330 (39861 to 97233)	38.95% (32.19 to 45.77)	2130 (1329.7 to 3218.5)	1608 (996.4 to 2430.5)	-1.15% (-1.24 to -1.07)
Republic of Moldova	2896 (1865 to 4063)	1953 (1239 to 2753)	-32.54% (-42.23 to -20.98)	1486.8 (957.7 to 2085.7)	1210 (767.4 to 1705.2)	0.37% (-0.08 to 0.83)
Russian Federation	68344 (44029 to 95222)	125700 (81744 to 173362)	83.92% (78.43 to 89.6)	1033.8 (666 to 1440.3)	2014.8 (1310.2 to 2778.7)	4.25% (3.56 to 4.93)
Rwanda	6999 (4420 to 10787)	8830 (5486 to 13312)	26.17% (-0.1 to 56.48)	2943.5 (1859 to 4537)	1774.3 (1102.3 to 2674.8)	-1.83% (-1.9 to -1.76)
Saint Lucia	54 (33 to 83)	40 (24 to 62)	-26.32% (-40.13 to -9.49)	1137 (697.3 to 1757.8)	618.2 (376.1 to 966.3)	-2.23% (-2.33 to -2.12)
Saint Vincent and the Grenadines	42 (26 to 67)	31 (19 to 48)	-25.6% (-38.4 to -7.69)	1119 (695.7 to 1776.3)	804.2 (488.5 to 1225.9)	-1.18% (-1.33 to -1.04)
Samoa	52 (31 to 79)	44 (27 to 68)	-14.66% (-31.11 to 5.33)	1093.6 (653.1 to 1675.3)	644.4 (401.9 to 999)	-1.89% (-2.01 to -1.76)

Location	Incident cases in 1990 (95% UI)	Incident cases in 2019 (95% UI)	Relative change (%, 95 CI)	ASIR in 1990 (95% UI)	ASIR in 2019 (95% UI)	EAPC (%, 95 CI)
Senegal	5223 (3254 to 7929)	8599 (5295 to 13293)	64.64% (29.82 to 102.59)	2253.8 (1404.1 to 3421.9)	1634.7 (1006.7 to 2527)	-0.85% (-0.96 to -0.74)
Serbia	1080 (602 to 1770)	1085 (612 to 1760)	0.43% (-22.13 to 27.38)	299.6 (167 to 491.1)	374.2 (211.1 to 607.1)	2.26% (1.69 to 2.82)
Sierra Leone	2526 (1584 to 3870)	4828 (3137 to 7352)	91.11% (49.62 to 138.89)	1981.3 (1242.6 to 3034.9)	1585 (1029.7 to 2413.4)	-0.79% (-0.84 to -0.75)
Solomon Islands	237 (144 to 367)	410 (251 to 629)	73.04% (37.93 to 109.65)	2530.3 (1540.9 to 3917.6)	1772.4 (1083.8 to 2715.4)	-1.38% (-1.45 to -1.31)
Somalia	6662 (4161 to 10246)	14044 (8288 to 21346)	110.83% (67.61 to 167.16)	3047.6 (1903.8 to 4687.4)	2573.7 (1518.9 to 3911.7)	-0.56% (-0.6 to -0.51)
South Africa	17972 (11623 to 26674)	26228 (16765 to 38985)	45.94% (33.47 to 56.37)	1277.5 (826.2 to 1896)	1025.5 (655.5 to 1524.4)	-0.57% (-0.63 to -0.5)
South Sudan	4201 (2586 to 6599)	6395 (4031 to 9946)	52.21% (24.59 to 91.73)	2450.5 (1508.6 to 3849.2)	2221.7 (1400.6 to 3455.5)	-0.32% (-0.37 to -0.28)
Sri Lanka	8580 (5072 to 13101)	9086 (5372 to 14375)	5.9% (-12.27 to 27.02)	1258.6 (743.9 to 1921.7)	1143.8 (676.3 to 1809.4)	-0.06% (-0.29 to 0.17)

Location	Incident cases in 1990 (95% UI)	Incident cases in 2019 (95% UI)	Relative change (%, 95 CI)	ASIR in 1990 (95% UI)	ASIR in 2019 (95% UI)	EAPC (%, 95 CI)
Sudan	16597 (9652 to 26636)	24532 (14219 to 37668)	47.81% (19.74 to 85.36)	2586.7 (1504.3 to 4151.4)	1576.6 (913.8 to 2420.7)	-1.83% (-1.9 to -1.75)
Suriname	150 (89 to 230)	213 (126 to 339)	41.94% (12.68 to 74.8)	1142.3 (681.8 to 1755.8)	986.1 (584.2 to 1573.3)	-0.23% (-0.34 to -0.12)
Syrian Arab Republic	6651 (3884 to 10498)	3582 (2098 to 5637)	-46.14% (-57.14 to -30.93)	1880.2 (1098.1 to 2967.9)	873.5 (511.5 to 1374.5)	-2.12% (-2.54 to -1.7)
Tajikistan	3734 (2210 to 6008)	5466 (3262 to 8832)	46.38% (16.54 to 80.57)	2082.3 (1232.4 to 3350.1)	1350.7 (805.9 to 2182.5)	-1.36% (-1.51 to -1.21)
Thailand	18906 (11510 to 29165)	15067 (9253 to 23024)	-20.3% (-33.91 to -1.45)	770.9 (469.3 to 1189.2)	612.7 (376.2 to 936.2)	-0.74% (-0.85 to -0.62)
Timor-Leste	818 (509 to 1271)	920 (554 to 1408)	12.47% (-7.08 to 36.21)	2929 (1821 to 4548.7)	2256.4 (1358.1 to 3452.8)	-1.22% (-1.41 to -1.04)
Togo	2493 (1546 to 3827)	5146 (3266 to 7797)	106.42% (67.64 to 162.1)	2107.3 (1307.1 to 3235)	1628 (1033.2 to 2466.8)	-0.61% (-0.7 to -0.51)

Location	Incident cases in 1990 (95% UI)	Incident cases in 2019 (95% UI)	Relative change (%, 95 CI)	ASIR in 1990 (95% UI)	ASIR in 2019 (95% UI)	EAPC (%, 95 CI)
Tonga	47 (29 to 73)	57 (35 to 87)	19.32% (-4.46 to 47.52)	1714.9 (1039.3 to 2622.1)	1682.9 (1032.5 to 2577.9)	-0.02% (-0.13 to 0.08)
Tunisia	4610 (2582 to 7406)	4832 (2915 to 7482)	4.8% (-14.64 to 38.88)	1568.4 (878.6 to 2519.5)	994.6 (600.1 to 1540.2)	-1.09% (-1.29 to -0.89)
Turkey	27988 (16323 to 44948)	38533 (22972 to 60178)	37.67% (16.44 to 67.8)	1340.3 (781.7 to 2152.6)	1144.1 (682.1 to 1786.7)	-0.32% (-0.44 to -0.21)
Turkmenistan	2908 (1798 to 4601)	2761 (1655 to 4338)	-5.05% (-24 to 20.24)	2093.9 (1294.6 to 3312.4)	1429.6 (856.6 to 2245.8)	-0.99% (-1.38 to -0.59)
Tuvalu	6 (4 to 10)	5 (3 to 8)	-24.59% (-39.44 to -6.92)	1682.5 (1032.5 to 2593.9)	1219 (754 to 1931.6)	-1.18% (-1.21 to -1.14)
Uganda	13862 (8811 to 20911)	24981 (15540 to 38842)	80.22% (47.17 to 124.37)	2851.3 (1812.3 to 4301.4)	1922.2 (1195.7 to 2988.7)	-1.48% (-1.63 to -1.33)
Ukraine	20079 (12855 to 27703)	26608 (17336 to 36715)	32.51% (13.93 to 53.52)	974.6 (623.9 to 1344.5)	1455.6 (948.3 to 2008.5)	3.19% (2.46 to 3.93)
United Republic of Tanzania	19752 (12841 to 30854)	34789 (21084 to 53177)	76.13% (44.88 to 116.07)	2519.6 (1638 to 3935.9)	1840.3 (1115.3 to 2813)	-1% (-1.08 to -0.91)

Location	Incident cases in 1990 (95% UI)	Incident cases in 2019 (95% UI)	Relative change (%, 95 CI)	ASIR in 1990 (95% UI)	ASIR in 2019 (95% UI)	EAPC (%, 95 CI)
Uzbekistan	12645 (7595 to 19492)	14215 (8297 to 22601)	12.42% (-10.68 to 39.12)	1674.4 (1005.7 to 2581)	975.2 (569.2 to 1550.5)	-1.67% (-2.06 to -1.27)
Vanuatu	94 (56 to 149)	147 (92 to 228)	56.19% (31.7 to 97.52)	1858.1 (1097.9 to 2938.1)	1357.9 (845.7 to 2107.2)	-1.18% (-1.3 to -1.06)
Venezuela (Bolivarian Republic of)	9085 (5570 to 14179)	9345 (5732 to 14346)	2.86% (-18.64 to 28.58)	1263 (774.4 to 1971.2)	835.6 (512.5 to 1282.8)	-1.1% (-1.3 to -0.91)
Viet Nam	31427 (19370 to 49376)	34058 (21025 to 52087)	8.37% (-11.84 to 29.26)	1178.3 (726.2 to 1851.2)	796.8 (491.9 to 1218.6)	-1.01% (-1.17 to -0.85)
Yemen	11969 (6924 to 19895)	16639 (9837 to 25734)	39.01% (10.61 to 73.65)	2906.9 (1681.6 to 4831.6)	1386.3 (819.6 to 2144.1)	-2.7% (-2.92 to -2.49)
Zambia	5766 (3484 to 8936)	10584 (6483 to 16445)	83.58% (43.02 to 130.41)	2568.3 (1552.1 to 3980.5)	1663.9 (1019.1 to 2585.3)	-1.53% (-1.67 to -1.4)
Zimbabwe	6334 (4058 to 9288)	8475 (5362 to 12904)	33.81% (8.84 to 62.37)	2017.3 (1292.5 to 2958.1)	1499.9 (948.9 to 2283.7)	-0.58% (-0.81 to -0.35)

ASIR – age-standardized incidence rate (per 100,000 population), CI – confidence interval, EAPC – estimated annual percentage change, MSMI – maternal sepsis and other maternal infections, UI – uncertain interval.

Table S12. Incidence of MSMI in 131 low- and middle-income countries and territories with EAPC in age group of 35-39 years from 1990 and 2019.

Location	Incident cases in 1990 (95% UI)	Incident cases in 2019 (95% UI)	Relative change (%, 95 CI)	ASIR in 1990 (95% UI)	ASIR in 2019 (95% UI)	EAPC (%, 95 CI)
Afghanistan	4213 (2721 to 6500)	7791 (4590 to 11828)	84.95% (41.16 to 143.2)	1559.9 (1007.5 to 2406.8)	983.9 (579.6 to 1493.6)	-1.56% (-1.75 to -1.38)
Albania	287 (164 to 459)	140 (84 to 219)	-51.27% (-64.18 to -30.46)	290.9 (166 to 464.7)	172.6 (103.2 to 270)	-1.72% (-2.29 to -1.16)
Algeria	8183 (5322 to 12424)	15520 (9776 to 24341)	89.66% (47.28 to 146.47)	1322.5 (860.1 to 2007.8)	887.9 (559.3 to 1392.6)	-0.26% (-0.82 to 0.31)
American Samoa	14 (9 to 21)	11 (7 to 17)	-16.87% (-32.59 to 2.74)	995.6 (644 to 1479)	734.8 (460.2 to 1099.8)	-1% (-1.02 to -0.98)
Angola	6374 (4008 to 9676)	13203 (7896 to 20646)	107.14% (64.54 to 158.04)	2333.7 (1467.5 to 3542.7)	1661.9 (993.8 to 2598.8)	-1.08% (-1.17 to -0.99)
Armenia	302 (183 to 465)	274 (168 to 426)	-9.04% (-28.57 to 16.26)	247.8 (150.3 to 381.7)	222.4 (136.6 to 345.5)	0.39% (-0.48 to 1.28)
Azerbaijan	777 (487 to 1201)	554 (340 to 870)	-28.76% (-42.52 to -11.01)	349.7 (219.3 to 540.2)	136 (83.4 to 213.6)	-2.39% (-2.83 to -1.94)
Bangladesh	19633 (12509 to 29559)	12392 (7826 to 18462)	-36.88% (-51.28 to -21.67)	762.8 (486 to 1148.5)	216.5 (136.7 to 322.6)	-4.62% (-4.8 to -4.44)

Location	Incident cases in 1990 (95% UI)	Incident cases in 2019 (95% UI)	Relative change (%, 95 CI)	ASIR in 1990 (95% UI)	ASIR in 2019 (95% UI)	EAPC (%, 95 CI)
Belarus	1070 (670 to 1662)	2519 (1591 to 3785)	135.33% (100.35 to 180.42)	280.5 (175.5 to 435.6)	677.3 (427.7 to 1017.7)	6.23% (5.1 to 7.38)
Belize	48 (29 to 73)	72 (43 to 111)	50.78% (23.68 to 84.76)	1101.3 (671.2 to 1686.9)	503.3 (299.3 to 775.2)	-2.24% (-2.46 to -2.03)
Benin	2495 (1463 to 3825)	4734 (2801 to 7379)	89.74% (54.3 to 129.3)	1996.6 (1171.1 to 3061.3)	1404.5 (831 to 2189.3)	-1.03% (-1.09 to -0.97)
Bhutan	160 (101 to 243)	110 (68 to 165)	-31.45% (-44.85 to -14.71)	1072.3 (675.5 to 1624.7)	392.9 (244.1 to 590.5)	-3.68% (-3.98 to -3.39)
Bolivia (Plurinational State of)	3036 (1871 to 4769)	4502 (2636 to 7015)	48.28% (18.08 to 86.71)	1631.8 (1005.5 to 2563.2)	1132 (662.8 to 1764.1)	-1.15% (-1.28 to -1.02)
Bosnia and Herzegovina	216 (128 to 328)	207 (122 to 316)	-3.9% (-29.01 to 30.09)	127.5 (75.8 to 194)	185.9 (109.5 to 283.5)	0.96% (0.63 to 1.29)
Botswana	409 (258 to 622)	701 (427 to 1062)	71.4% (39.18 to 110.57)	1168.6 (738.5 to 1779.6)	707.4 (431.3 to 1072.1)	-1.64% (-1.73 to -1.56)
Brazil	19080 (11936 to 29023)	34999 (22900 to 52620)	83.43% (54.29 to 110.51)	393.2 (246 to 598.2)	390.8 (255.7 to 587.5)	0.79% (0.11 to 1.48)
Bulgaria	151 (89 to 241)	444 (256 to 701)	193.15% (114.49 to 295.4)	48.9 (28.7 to 77.9)	190.7 (110.1 to 301.1)	6.51% (5.86 to 7.16)

Location	Incident cases in 1990 (95% UI)	Incident cases in 2019 (95% UI)	Relative change (%, 95 CI)	ASIR in 1990 (95% UI)	ASIR in 2019 (95% UI)	EAPC (%, 95 CI)
Burkina Faso	5115 (3115 to 7906)	10405 (6140 to 15696)	103.42% (55.84 to 155.99)	2169.6 (1321.4 to 3353.4)	1628.9 (961.1 to 2457.2)	-1.12% (-1.19 to -1.05)
Burundi	3525 (2190 to 5359)	6837 (4271 to 10371)	93.95% (56.09 to 135.21)	2460.7 (1528.6 to 3740.6)	2151.3 (1344 to 3263.4)	-0.29% (-0.36 to -0.22)
Cabo Verde	101 (60 to 162)	124 (76 to 194)	22.95% (-2.58 to 55.69)	1262.3 (754 to 2026.2)	624.1 (381.8 to 979.2)	-2.34% (-2.47 to -2.2)
Cambodia	5677 (3533 to 8725)	3930 (2488 to 5928)	-30.78% (-43 to -15.92)	1945.9 (1211 to 2990.7)	655.9 (415.3 to 989.4)	-4.08% (-4.28 to -3.88)
Cameroon	4163 (2510 to 6539)	9458 (5570 to 14698)	127.19% (82.84 to 187.3)	1643.1 (990.8 to 2580.9)	1111.1 (654.3 to 1726.6)	-1.02% (-1.22 to -0.82)
Central African Republic	1247 (777 to 1886)	2055 (1253 to 3169)	64.72% (32.87 to 102.86)	1839.4 (1145.5 to 2780.9)	1368 (833.9 to 2109.7)	-0.97% (-1.01 to -0.94)
Chad	3203 (1888 to 5056)	7372 (4325 to 11442)	130.14% (88.09 to 188.46)	2028 (1195.3 to 3200.9)	1941.2 (1138.9 to 3012.9)	-0.16% (-0.24 to -0.09)
China	99534 (63294 to 148017)	88960 (56790 to 131768)	-10.62% (-15.54 to -5.3)	225.3 (143.2 to 335)	179.7 (114.7 to 266.2)	2.05% (1.07 to 3.04)

Location	Incident cases in 1990 (95% UI)	Incident cases in 2019 (95% UI)	Relative change (%, 95 CI)	ASIR in 1990 (95% UI)	ASIR in 2019 (95% UI)	EAPC (%, 95 CI)
Colombia	7687 (4654 to 12040)	8346 (4835 to 13052)	8.57% (-15.12 to 35.81)	743.2 (449.9 to 1164)	469.9 (272.3 to 734.9)	-1.76% (-1.9 to -1.62)
Comoros	288 (180 to 445)	259 (155 to 401)	-10.13% (-30.03 to 14.17)	2606.6 (1629.7 to 4023.5)	1143.2 (682.6 to 1767.7)	-2.85% (-2.96 to -2.74)
Congo	1152 (724 to 1747)	2094 (1297 to 3260)	81.8% (46.97 to 123.44)	1846.8 (1160.2 to 2801.4)	1125.2 (697.1 to 1752.1)	-1.41% (-1.52 to -1.3)
Costa Rica	673 (401 to 1047)	716 (420 to 1111)	6.38% (-17.24 to 34.83)	705.4 (420.1 to 1098.2)	382.1 (224 to 593.2)	-1.93% (-2.4 to -1.45)
Côte d'Ivoire	5287 (3166 to 8004)	9990 (5929 to 15578)	88.97% (50.69 to 131.11)	1909.1 (1143.4 to 2890.2)	1241.7 (736.9 to 1936.2)	-1.51% (-1.54 to -1.49)
Cuba	686 (396 to 1059)	872 (527 to 1343)	27.11% (0.49 to 56.52)	193 (111.4 to 298)	267.3 (161.5 to 412)	1.67% (1.42 to 1.92)
Democratic People's Republic of Korea	4294 (2608 to 6628)	1666 (1038 to 2490)	-61.19% (-67.75 to -51.6)	690.6 (419.4 to 1066)	188.6 (117.4 to 281.7)	-4.01% (-4.29 to -3.72)
Democratic Republic of the Congo	23099 (14048 to 35048)	38145 (24117 to 57373)	65.14% (31.94 to 115.8)	2405 (1462.6 to 3649.1)	1648.9 (1042.5 to 2480.1)	-1.11% (-1.27 to -0.95)

Location	Incident cases in 1990 (95% UI)	Incident cases in 2019 (95% UI)	Relative change (%, 95 CI)	ASIR in 1990 (95% UI)	ASIR in 2019 (95% UI)	EAPC (%, 95 CI)
Djibouti	237 (139 to 371)	604 (365 to 914)	155.26% (103.39 to 232.59)	2007 (1181.3 to 3144.4)	1267.6 (765.5 to 1918.9)	-1.7% (-1.79 to -1.62)
Dominica	12 (7 to 19)	10 (6 to 16)	-16.65% (-34.27 to 4.11)	630.4 (382.8 to 992.3)	450.4 (273.5 to 711.4)	-1.23% (-1.36 to -1.09)
Dominican Republic	1185 (709 to 1872)	1187 (719 to 1866)	0.15% (-19.65 to 24.76)	562.9 (336.7 to 889.1)	320.2 (193.9 to 503.4)	-1.97% (-2.18 to -1.75)
Ecuador	3150 (1917 to 4742)	4102 (2350 to 6466)	30.23% (3.45 to 61.83)	1075.3 (654.6 to 1618.9)	669.2 (383.5 to 1055)	-1.54% (-1.8 to -1.28)
Egypt	11811 (7189 to 18498)	12322 (7798 to 19119)	4.32% (-19.26 to 34.48)	740 (450.5 to 1159)	361.9 (229 to 561.6)	-1.73% (-2.05 to -1.41)
El Salvador	1130 (648 to 1777)	906 (520 to 1464)	-19.83% (-35.21 to 3.1)	790.7 (453.7 to 1243.9)	410.3 (235.5 to 663.3)	-2.42% (-2.71 to -2.13)
Equatorial Guinea	280 (171 to 428)	376 (235 to 572)	34.4% (8.96 to 69.4)	2224 (1360.7 to 3403.4)	965.5 (605.1 to 1468.8)	-3% (-3.11 to -2.88)
Eritrea	1722 (1075 to 2610)	3048 (1846 to 4579)	77% (44.27 to 121.06)	2200.1 (1373.6 to 3333.5)	1562.3 (946.2 to 2346.6)	-1.18% (-1.24 to -1.11)

Location	Incident cases in 1990 (95% UI)	Incident cases in 2019 (95% UI)	Relative change (%, 95 CI)	ASIR in 1990 (95% UI)	ASIR in 2019 (95% UI)	EAPC (%, 95 CI)
Eswatini	295 (186 to 446)	321 (205 to 487)	8.81% (-11.18 to 37.8)	1470.1 (926.1 to 2221.3)	828.4 (527.4 to 1255.9)	-1.88% (-1.93 to -1.82)
Ethiopia	34378 (21884 to 52435)	51602 (32555 to 77505)	50.1% (34.1 to 68.67)	2558.9 (1628.9 to 3902.9)	1706 (1076.3 to 2562.4)	-1.48% (-1.54 to -1.41)
Fiji	115 (75 to 170)	188 (118 to 280)	64% (32.08 to 100.31)	479 (312.1 to 707.7)	572.7 (359.3 to 850.9)	0.68% (0.57 to 0.8)
Gabon	425 (261 to 651)	535 (328 to 820)	25.69% (1.19 to 55.13)	1770 (1087.3 to 2707.1)	900.5 (552.1 to 1381.3)	-2.11% (-2.2 to -2.03)
Gambia	500 (310 to 778)	964 (575 to 1463)	92.73% (57.98 to 139.53)	2132.8 (1322 to 3319.5)	1468.9 (876.7 to 2230.6)	-1.3% (-1.39 to -1.22)
Georgia	351 (218 to 531)	475 (288 to 733)	35.46% (10.52 to 68.79)	178.4 (111.1 to 270)	371.5 (225.6 to 573)	3.27% (2.94 to 3.59)
Ghana	6388 (3919 to 9858)	10581 (6377 to 16166)	65.64% (29.34 to 104.27)	1597.1 (979.7 to 2464.8)	973.3 (586.6 to 1487)	-1.63% (-1.73 to -1.52)
Global	1069279 (698567 to 1582333)	1390759 (905238 to 2057087)	30.07% (26.84 to 33.14)	615.5 (402.1 to 910.8)	518.1 (337.2 to 766.3)	-0.34% (-0.54 to -0.15)
Grenada	23 (14 to 35)	19 (11 to 29)	-17.37% (-33.6 to 2.44)	958.1 (587 to 1477.3)	623.2 (372 to 982.1)	-1.35% (-1.52 to -1.18)

Location	Incident cases in 1990 (95% UI)	Incident cases in 2019 (95% UI)	Relative change (%, 95 CI)	ASIR in 1990 (95% UI)	ASIR in 2019 (95% UI)	EAPC (%, 95 CI)
Guatemala	4019 (2331 to 6308)	4250 (2466 to 6650)	5.75% (-16.07 to 33.6)	1899.4 (1101.6 to 2981.4)	670.5 (389 to 1049.2)	-4.11% (-4.46 to -3.76)
Guinea	3178 (1877 to 4794)	4541 (2744 to 6959)	42.88% (15.35 to 79.02)	1878.2 (1109.4 to 2832.8)	1269 (766.7 to 1944.7)	-1.38% (-1.46 to -1.3)
Guinea-Bissau	509 (307 to 773)	761 (478 to 1173)	49.48% (19.72 to 85.56)	1857.4 (1118.1 to 2818.8)	1288.3 (808.4 to 1984.6)	-1.38% (-1.44 to -1.32)
Guyana	111 (66 to 174)	110 (66 to 169)	-1.2% (-20.33 to 23.06)	474.3 (282.9 to 741.9)	439.1 (264.3 to 674.3)	0.06% (-0.15 to 0.27)
Haiti	3671 (2270 to 5706)	5753 (3513 to 8926)	56.69% (27.72 to 95.65)	1903.5 (1176.7 to 2958.6)	1212 (740 to 1880.4)	-1.57% (-1.61 to -1.53)
Honduras	1814 (1055 to 2823)	2125 (1265 to 3272)	17.16% (-8.53 to 48.92)	1518.3 (882.9 to 2363.1)	650.1 (387 to 1001)	-3.21% (-3.47 to -2.94)
India	195478 (128324 to 285042)	139740 (91078 to 204360)	-28.51% (-34.06 to -23.08)	768.4 (504.5 to 1120.5)	281.8 (183.7 to 412.1)	-3.84% (-4.07 to -3.61)
Indonesia	41245 (26549 to 61647)	60788 (38837 to 90689)	47.38% (39 to 57.57)	748.6 (481.8 to 1118.9)	599.4 (382.9 to 894.2)	-0.64% (-0.84 to -0.44)

Location	Incident cases in 1990 (95% UI)	Incident cases in 2019 (95% UI)	Relative change (%, 95 CI)	ASIR in 1990 (95% UI)	ASIR in 2019 (95% UI)	EAPC (%, 95 CI)
Iran (Islamic Republic of)	14916 (9672 to 22436)	19238 (12449 to 28519)	28.98% (20.65 to 40.85)	1056.5 (685 to 1589.1)	464.1 (300.3 to 688)	-1.14% (-2.3 to 0.03)
Iraq	5869 (3608 to 9032)	7475 (4759 to 11423)	27.37% (1.18 to 66.12)	1383.7 (850.8 to 2129.6)	601.4 (382.9 to 919)	-2.9% (-3.02 to -2.78)
Jamaica	376 (224 to 570)	467 (285 to 711)	24.3% (1.73 to 55.57)	572.9 (342.1 to 869.7)	454.7 (277 to 691.6)	-0.66% (-0.81 to -0.5)
Jordan	799 (514 to 1228)	1560 (1052 to 2268)	95.15% (48.5 to 155.94)	990.5 (636.6 to 1521.3)	417.9 (281.9 to 607.5)	-2.92% (-3.06 to -2.79)
Kazakhstan	2640 (1626 to 4091)	3596 (2171 to 5597)	36.22% (8.48 to 70.72)	452 (278.5 to 700.5)	528.4 (319 to 822.6)	2.26% (1.62 to 2.89)
Kenya	10049 (6424 to 15239)	13681 (8796 to 20323)	36.14% (31.77 to 41)	2028.3 (1296.6 to 3075.9)	879 (565.1 to 1305.7)	-2.81% (-3 to -2.62)
Kiribati	26 (16 to 39)	38 (24 to 58)	49.8% (21.12 to 85.98)	1144.3 (719.8 to 1719.2)	936.3 (579.8 to 1413.6)	-0.8% (-0.95 to -0.64)
Kyrgyzstan	768 (473 to 1176)	1059 (649 to 1672)	37.92% (7.33 to 67.43)	578.8 (356.7 to 887.1)	501 (307.2 to 791.2)	0.81% (0.32 to 1.31)
Lao People's Democratic Republic	1649 (1019 to 2551)	1584 (959 to 2355)	-3.93% (-22.75 to 16.97)	1530 (945.2 to 2366.4)	649.9 (393.5 to 965.9)	-3.8% (-4.1 to -3.49)

Location	Incident cases in 1990 (95% UI)	Incident cases in 2019 (95% UI)	Relative change (%, 95 CI)	ASIR in 1990 (95% UI)	ASIR in 2019 (95% UI)	EAPC (%, 95 CI)
Lebanon	711 (447 to 1077)	723 (447 to 1115)	1.56% (-19.79 to 32.57)	747.8 (470.1 to 1131.9)	341.7 (211.6 to 527.3)	-2.13% (-2.38 to -1.89)
Lesotho	628 (393 to 958)	513 (326 to 778)	-18.31% (-32.59 to 2.19)	1388.3 (868.6 to 2116.5)	709.6 (450.9 to 1076)	-2.4% (-2.45 to -2.35)
Liberia	1057 (636 to 1633)	1623 (977 to 2597)	53.62% (25.41 to 91.6)	1847.4 (1112.9 to 2856)	1062.2 (639.5 to 1699.8)	-2.03% (-2.19 to -1.87)
Libya	938 (578 to 1435)	1311 (805 to 1987)	39.78% (8.43 to 76.47)	1068 (658.3 to 1633.8)	442.9 (272.1 to 671.3)	-2.92% (-3.09 to -2.76)
Madagascar	5236 (3160 to 7842)	8454 (5219 to 13010)	61.48% (28.06 to 102.21)	1798.2 (1085.2 to 2693.3)	1099.9 (679 to 1692.7)	-1.79% (-1.89 to -1.7)
Malawi	4939 (2995 to 7579)	5133 (3065 to 7964)	3.91% (-17.6 to 32.96)	2115.8 (1282.7 to 3246.3)	976.6 (583.2 to 1515.4)	-2.76% (-2.95 to -2.56)
Malaysia	6176 (3877 to 9381)	8297 (5097 to 12635)	34.34% (11.4 to 64.32)	1085.5 (681.5 to 1648.9)	742.2 (456 to 1130.2)	-1.56% (-1.72 to -1.39)
Maldives	68 (43 to 104)	105 (65 to 159)	53.05% (25.22 to 84.55)	1598 (1011.1 to 2443)	573.5 (358.8 to 873.4)	-3.48% (-3.87 to -3.09)

Location	Incident cases in 1990 (95% UI)	Incident cases in 2019 (95% UI)	Relative change (%, 95 CI)	ASIR in 1990 (95% UI)	ASIR in 2019 (95% UI)	EAPC (%, 95 CI)
Mali	5185 (3160 to 7978)	9651 (5704 to 15203)	86.13% (53.62 to 133.46)	2363.2 (1440.3 to 3636.2)	1820.5 (1075.9 to 2867.7)	-0.89% (-0.93 to -0.86)
Marshall Islands	9 (6 to 13)	12 (7 to 18)	36.25% (6.89 to 71.93)	747 (477.6 to 1117.5)	569.6 (353.4 to 846)	-0.3% (-0.59 to -0.01)
Mauritania	1216 (717 to 1902)	1651 (1013 to 2618)	35.83% (12.23 to 72.92)	2192.4 (1293 to 3429.7)	1443.9 (885.8 to 2289.3)	-1.4% (-1.52 to -1.27)
Mexico	25078 (15502 to 39358)	33274 (20313 to 52200)	32.68% (21.28 to 45.66)	1025.7 (634.1 to 1609.8)	717.2 (437.8 to 1125.1)	-0.66% (-1.48 to 0.16)
Micronesia (Federated States of)	44 (28 to 68)	26 (17 to 39)	-40.72% (-52.35 to -26.67)	1629.7 (1021.2 to 2516.3)	860.2 (544.8 to 1292.7)	-2.31% (-2.47 to -2.14)
Mongolia	440 (271 to 678)	1008 (606 to 1593)	129.22% (81.99 to 207.04)	859.9 (530.3 to 1325.3)	745.2 (448.2 to 1177.5)	1.31% (0.24 to 2.4)
Montenegro	41 (25 to 64)	54 (33 to 84)	33.36% (-0.99 to 80.56)	182 (113.1 to 288.7)	240 (144.5 to 372.5)	1.29% (1.16 to 1.41)
Morocco	7163 (4484 to 10893)	7580 (4721 to 11873)	5.82% (-19.31 to 34.78)	970 (607.3 to 1475.2)	560.4 (349 to 877.8)	-1.76% (-1.81 to -1.71)

Location	Incident cases in 1990 (95% UI)	Incident cases in 2019 (95% UI)	Relative change (%, 95 CI)	ASIR in 1990 (95% UI)	ASIR in 2019 (95% UI)	EAPC (%, 95 CI)
Mozambique	6684 (4121 to 10049)	9658 (6049 to 14731)	44.49% (15.11 to 76.65)	1781.8 (1098.6 to 2678.9)	1271.8 (796.5 to 1939.9)	-1.01% (-1.15 to -0.87)
Myanmar	14600 (9243 to 22549)	15115 (9430 to 22758)	3.52% (-15.11 to 23.68)	1175.1 (744 to 1815)	736.2 (459.3 to 1108.5)	-1.82% (-1.91 to -1.73)
Namibia	625 (384 to 947)	781 (496 to 1201)	25.05% (0.86 to 56.64)	1742.8 (1071.1 to 2641.7)	982.2 (623 to 1509.7)	-1.56% (-1.77 to -1.35)
Nepal	5474 (3334 to 8089)	1910 (1141 to 2961)	-65.1% (-73.06 to -56.34)	1029.9 (627.3 to 1521.9)	174.4 (104.1 to 270.3)	-7.01% (-7.4 to -6.62)
Nicaragua	997 (592 to 1538)	1016 (597 to 1574)	1.9% (-19 to 29.4)	1022.1 (606.7 to 1576.8)	434.8 (255.4 to 673.8)	-2.96% (-3.4 to -2.52)
Niger	3758 (2330 to 5795)	10542 (6229 to 16360)	180.54% (126.37 to 249.34)	2153.3 (1335.4 to 3320.8)	2146.6 (1268.3 to 3331.1)	0.03% (0 to 0.06)
Nigeria	49361 (31350 to 73049)	100423 (63996 to 149997)	103.44% (94.54 to 113.12)	2398.9 (1523.6 to 3550.1)	1727.1 (1100.6 to 2579.7)	-1.3% (-1.45 to -1.15)
North Macedonia	82 (48 to 128)	140 (86 to 217)	69.69% (28.47 to 130.77)	112.8 (65.5 to 174.5)	170 (104.4 to 264.1)	1.98% (1.66 to 2.31)

Location	Incident cases in 1990 (95% UI)	Incident cases in 2019 (95% UI)	Relative change (%, 95 CI)	ASIR in 1990 (95% UI)	ASIR in 2019 (95% UI)	EAPC (%, 95 CI)
Pakistan	47777 (31214 to 69594)	39903 (26420 to 58392)	-16.48% (-25.3 to -7.25)	1845.9 (1206 to 2688.8)	613.6 (406.3 to 897.9)	-3.91% (-4.2 to -3.61)
Papua New Guinea	1666 (1026 to 2469)	4055 (2553 to 6087)	143.48% (95.17 to 201.61)	1539.7 (948.4 to 2282.1)	1268.7 (798.6 to 1904.5)	-0.76% (-0.81 to -0.71)
Paraguay	1090 (635 to 1710)	1086 (643 to 1692)	-0.34% (-21.76 to 29.03)	958.1 (558.1 to 1503.5)	446.3 (264.1 to 695.1)	-2.79% (-2.86 to -2.71)
Peru	7286 (4470 to 11201)	8372 (5044 to 12919)	14.9% (-9.05 to 44.16)	1168.2 (716.7 to 1795.9)	664 (400.1 to 1024.7)	-2.07% (-2.18 to -1.95)
Philippines	25469 (16450 to 38488)	36400 (23301 to 54752)	42.92% (36.28 to 50.66)	1425.9 (920.9 to 2154.7)	1004.7 (643.1 to 1511.2)	-1.35% (-1.4 to -1.3)
Republic of Moldova	920 (572 to 1421)	661 (409 to 1018)	-28.12% (-39.46 to -14.46)	501.9 (312.3 to 775.6)	434.8 (269.2 to 669.1)	1.16% (0.52 to 1.81)
Russian Federation	23272 (14948 to 34636)	53649 (34455 to 79600)	130.53% (123.88 to 136.43)	381.2 (244.8 to 567.3)	892.7 (573.3 to 1324.5)	5.52% (4.55 to 6.5)
Rwanda	4353 (2705 to 6684)	5595 (3463 to 8654)	28.54% (3.32 to 61.25)	2300.7 (1429.8 to 3532.7)	1338.9 (828.7 to 2070.8)	-2.04% (-2.19 to -1.9)
Saint Lucia	24 (14 to 38)	24 (14 to 37)	-1.07% (-21.16 to 22.71)	653.1 (389 to 1036.3)	351.1 (208 to 545.8)	-2.31% (-2.43 to -2.2)

Location	Incident cases in 1990 (95% UI)	Incident cases in 2019 (95% UI)	Relative change (%, 95 CI)	ASIR in 1990 (95% UI)	ASIR in 2019 (95% UI)	EAPC (%, 95 CI)
Saint Vincent and the Grenadines	17 (11 to 27)	20 (12 to 31)	14.16% (-12.9 to 41.35)	623.9 (380.6 to 969)	499.2 (301.9 to 779.2)	-0.78% (-0.89 to -0.67)
Samoa	24 (16 to 37)	22 (14 to 32)	-11.02% (-27.96 to 11.54)	630.3 (401.3 to 950.1)	379.4 (239.6 to 567.2)	-1.74% (-1.82 to -1.65)
Senegal	3813 (2247 to 5989)	6020 (3600 to 9410)	57.85% (28.04 to 101.51)	2085.5 (1228.8 to 3274.9)	1409 (842.5 to 2202.6)	-1.03% (-1.2 to -0.86)
Serbia	295 (177 to 450)	409 (255 to 610)	38.38% (5.48 to 77.03)	87.1 (52.3 to 132.6)	135.9 (84.6 to 202.8)	3.69% (2.95 to 4.43)
Sierra Leone	1730 (1017 to 2694)	2857 (1725 to 4427)	65.11% (29.4 to 105.89)	1691.8 (993.9 to 2633.8)	1211.5 (731.6 to 1877.2)	-1.24% (-1.32 to -1.16)
Solomon Islands	140 (91 to 207)	243 (153 to 364)	73.21% (38.43 to 109.85)	1873.1 (1209 to 2768.9)	1176 (741.8 to 1764.7)	-1.83% (-1.94 to -1.72)
Somalia	5328 (3234 to 8164)	11092 (6824 to 16756)	108.2% (67.64 to 161.46)	2463 (1494.9 to 3774.5)	2141.3 (1317.4 to 3234.7)	-0.43% (-0.47 to -0.4)
South Africa	10175 (6638 to 14891)	14081 (9097 to 20956)	38.39% (26.95 to 48.76)	900 (587.1 to 1317.2)	652 (421.2 to 970.3)	-0.93% (-0.99 to -0.88)

Location	Incident cases in 1990 (95% UI)	Incident cases in 2019 (95% UI)	Relative change (%, 95 CI)	ASIR in 1990 (95% UI)	ASIR in 2019 (95% UI)	EAPC (%, 95 CI)
South Sudan	2516 (1521 to 3814)	4763 (2928 to 7125)	89.36% (53.31 to 144.08)	1989.8 (1203 to 3017.3)	1748.3 (1074.6 to 2614.9)	-0.46% (-0.5 to -0.42)
Sri Lanka	3947 (2459 to 6046)	4831 (3058 to 7511)	22.4% (-0.61 to 45.8)	656.6 (409 to 1005.7)	576.2 (364.7 to 895.8)	-0.18% (-0.43 to 0.08)
Sudan	7772 (4897 to 11637)	10209 (6732 to 15150)	31.36% (5.42 to 70.38)	1478.6 (931.6 to 2214)	768.1 (506.5 to 1139.8)	-2.52% (-2.65 to -2.39)
Suriname	53 (32 to 84)	106 (62 to 164)	98% (60.76 to 146.55)	513 (307.7 to 804.8)	529.9 (312.6 to 820.8)	0.23% (0.08 to 0.38)
Syrian Arab Republic	2733 (1714 to 4302)	2289 (1435 to 3549)	-16.24% (-33.7 to 6)	979.4 (614.2 to 1541.8)	404.5 (253.6 to 627.2)	-2.43% (-3.26 to -1.58)
Tajikistan	1472 (914 to 2283)	1743 (1072 to 2700)	18.39% (-4.98 to 44)	1126.5 (699.7 to 1746.9)	578.8 (356 to 896.9)	-1.86% (-2.09 to -1.62)
Thailand	7734 (4618 to 11678)	7024 (4303 to 10471)	-9.18% (-27.25 to 11.55)	383 (228.7 to 578.3)	257 (157.4 to 383.2)	-1.25% (-1.39 to -1.12)
Timor-Leste	505 (315 to 774)	512 (321 to 771)	1.38% (-16.88 to 21.82)	2316.3 (1447.2 to 3555.2)	1618.4 (1015.8 to 2437.7)	-1.48% (-1.75 to -1.21)

Location	Incident cases in 1990 (95% UI)	Incident cases in 2019 (95% UI)	Relative change (%, 95 CI)	ASIR in 1990 (95% UI)	ASIR in 2019 (95% UI)	EAPC (%, 95 CI)
Togo	1857 (1083 to 2867)	3364 (2019 to 5169)	81.17% (46.92 to 121.48)	1972.8 (1150.2 to 3045.9)	1262.9 (758.1 to 1940.4)	-1.31% (-1.4 to -1.23)
Tonga	31 (19 to 46)	35 (22 to 53)	14.45% (-8.2 to 41.77)	1320.4 (819.4 to 1998)	1108.9 (686.2 to 1672.4)	-0.39% (-0.49 to -0.29)
Tunisia	1776 (1103 to 2754)	2484 (1540 to 3864)	39.83% (11.94 to 83.89)	773.8 (480.5 to 1199.7)	498.2 (308.9 to 775.1)	-0.8% (-1.1 to -0.5)
Turkey	8702 (5348 to 13750)	14600 (8855 to 22578)	67.78% (40.18 to 101.81)	500.3 (307.5 to 790.5)	444.3 (269.5 to 687.1)	0.32% (0 to 0.63)
Turkmenistan	1020 (654 to 1544)	794 (495 to 1218)	-22.15% (-38.28 to -2.06)	958.6 (614.7 to 1451.7)	474.3 (295.5 to 727.9)	-2.08% (-2.58 to -1.57)
Tuvalu	4 (2 to 6)	3 (2 to 4)	-28.65% (-41.86 to -13.16)	1108.5 (687.7 to 1692.1)	798 (496 to 1217.5)	-1.24% (-1.29 to -1.19)
Uganda	8407 (5255 to 12903)	13935 (8654 to 21446)	65.75% (34.51 to 104.25)	2262 (1414 to 3471.6)	1383.4 (859.2 to 2129.1)	-1.74% (-1.92 to -1.56)
Ukraine	6449 (4088 to 9600)	10950 (6818 to 16375)	69.81% (44.02 to 97.55)	325.1 (206.1 to 484)	602.4 (375.1 to 900.8)	4.44% (3.46 to 5.42)
United Republic of Tanzania	12236 (7163 to 18396)	22298 (13483 to 34598)	82.22% (50.43 to 126.77)	1969.7 (1153 to 2961.2)	1397.3 (844.9 to 2168.1)	-0.94% (-1.05 to -0.83)

Location	Incident cases in 1990 (95% UI)	Incident cases in 2019 (95% UI)	Relative change (%, 95 CI)	ASIR in 1990 (95% UI)	ASIR in 2019 (95% UI)	EAPC (%, 95 CI)
Uzbekistan	3570 (2144 to 5542)	3068 (1894 to 4683)	-14.04% (-31.36 to 9.67)	594.6 (357.1 to 923.1)	252.8 (156.1 to 385.8)	-2.73% (-3.24 to -2.21)
Vanuatu	54 (34 to 83)	71 (45 to 106)	31.15% (7.28 to 60.32)	1298.8 (823.8 to 1984.3)	803.5 (511.8 to 1200.2)	-1.82% (-1.99 to -1.64)
Venezuela (Bolivarian Republic of)	4569 (2684 to 6978)	4763 (2828 to 7332)	4.24% (-17.39 to 30.04)	745.4 (438 to 1138.5)	435.8 (258.8 to 670.9)	-1.44% (-1.65 to -1.23)
Viet Nam	11666 (7326 to 17465)	13024 (8031 to 19308)	11.64% (-8.77 to 34.67)	580.1 (364.3 to 868.5)	326.8 (201.5 to 484.5)	-1.59% (-1.9 to -1.27)
Yemen	6380 (3978 to 9695)	8761 (5494 to 13762)	37.32% (7.3 to 75.08)	1896.8 (1182.7 to 2882.4)	854 (535.6 to 1341.6)	-2.94% (-3.26 to -2.62)
Zambia	3457 (2074 to 5311)	6108 (3709 to 9582)	76.68% (39.05 to 125.76)	2056.1 (1233.3 to 3158.7)	1193.9 (725 to 1872.9)	-1.82% (-2 to -1.65)
Zimbabwe	4126 (2632 to 6258)	5119 (3163 to 7680)	24.06% (-0.35 to 59.81)	1605.1 (1023.9 to 2434.7)	1027.1 (634.7 to 1541)	-1.04% (-1.33 to -0.75)

ASIR – age-standardized incidence rate (per 100,000 population), CI – confidence interval, EAPC – estimated annual percentage change, MSMI – maternal sepsis and other maternal infections, UI – uncertain interval.

Table S13. Incidence of MSMI in 131 low- and middle-income countries and territories with EAPC in age group of 40-44 years from 1990 and 2019.

Location	Incident cases in 1990 (95% UI)	Incident cases in 2019 (95% UI)	Relative change (%, 95 CI)	ASIR in 1990 (95% UI)	ASIR in 2019 (95% UI)	EAPC (%, 95 CI)
Afghanistan	1823 (1185 to 2713)	3446 (2192 to 5002)	89.07% (51.39 to 139.95)	738.7 (480.5 to 1099.5)	516.6 (328.5 to 749.7)	-1.32% (-1.39 to -1.24)
Albania	85 (54 to 127)	29 (19 to 43)	-65.5% (-72.75 to -55.48)	117.9 (74.3 to 176.3)	36.2 (23.8 to 52.8)	-4.05% (-4.62 to -3.47)
Algeria	2905 (1860 to 4158)	6877 (4375 to 10333)	136.7% (89.4 to 195.67)	646.9 (414.1 to 925.9)	482 (306.6 to 724.2)	0.32% (-0.33 to 0.96)
American Samoa	4 (3 to 6)	3 (2 to 5)	-21.37% (-34.64 to -5.72)	398.7 (271.9 to 568.6)	209.2 (139.2 to 306.5)	-2.18% (-2.29 to -2.07)
Angola	3216 (2207 to 4422)	7560 (5130 to 10819)	135.07% (98.09 to 179.19)	1671.3 (1147.1 to 2298)	1083.7 (735.3 to 1550.7)	-1.32% (-1.43 to -1.2)
Armenia	49 (33 to 71)	48 (31 to 68)	-2.29% (-18.55 to 17.94)	62.3 (41.6 to 90.9)	46.8 (30.9 to 67.1)	-0.38% (-1.16 to 0.4)
Azerbaijan	156 (103 to 229)	132 (87 to 195)	-15.31% (-28.78 to 1.04)	113.4 (75 to 166.6)	39.4 (25.9 to 58.1)	-2.88% (-3.27 to -2.48)
Bangladesh	6507 (4456 to 9235)	3667 (2501 to 5139)	-43.65% (-53.8 to -32.11)	323.5 (221.5 to 459.1)	70.8 (48.3 to 99.2)	-5.12% (-5.42 to -4.82)

Location	Incident cases in 1990 (95% UI)	Incident cases in 2019 (95% UI)	Relative change (%, 95 CI)	ASIR in 1990 (95% UI)	ASIR in 2019 (95% UI)	EAPC (%, 95 CI)
Belarus	213 (141 to 316)	459 (304 to 675)	114.9% (85.89 to 148.65)	70.1 (46.5 to 103.7)	134.1 (88.8 to 197.2)	4.96% (3.84 to 6.09)
Belize	22 (15 to 31)	20 (13 to 27)	-9.96% (-23.33 to 3.86)	674.7 (468.7 to 948.9)	158.2 (105.9 to 217.5)	-4.42% (-4.67 to -4.17)
Benin	1190 (796 to 1660)	2677 (1830 to 3688)	124.98% (97.03 to 165.31)	1363.9 (912.6 to 1903)	962.6 (657.9 to 1326.2)	-1.08% (-1.12 to -1.04)
Bhutan	64 (44 to 90)	33 (23 to 45)	-48.64% (-56.54 to -38.5)	564.7 (392.2 to 798.6)	151 (104.5 to 210.2)	-4.59% (-4.89 to -4.29)
Bolivia (Plurinational State of)	1574 (1079 to 2191)	1915 (1277 to 2716)	21.62% (2.85 to 42.03)	1065.6 (730.4 to 1483.1)	546.3 (364.3 to 775)	-2.15% (-2.27 to -2.02)
Bosnia and Herzegovina	54 (35 to 79)	47 (30 to 70)	-12.81% (-31.79 to 12.95)	40.4 (26.2 to 58.9)	38.6 (24.2 to 56.7)	0.08% (-0.06 to 0.22)
Botswana	193 (131 to 268)	291 (194 to 407)	50.88% (28.88 to 76.68)	765.2 (520.7 to 1061.3)	385.5 (256 to 538.2)	-2.26% (-2.32 to -2.2)
Brazil	9774 (7542 to 12865)	11605 (8521 to 15846)	18.74% (4.69 to 31.09)	248.7 (191.9 to 327.3)	143.8 (105.6 to 196.3)	-1.28% (-2.05 to -0.51)
Bulgaria	38 (25 to 56)	143 (93 to 207)	279.83% (202.93 to 377.13)	11.6 (7.6 to 17)	55.3 (35.8 to 80)	6.11% (5.48 to 6.75)

Location	Incident cases in 1990 (95% UI)	Incident cases in 2019 (95% UI)	Relative change (%, 95 CI)	ASIR in 1990 (95% UI)	ASIR in 2019 (95% UI)	EAPC (%, 95 CI)
Burkina Faso	3167 (2171 to 4383)	6267 (4148 to 8797)	97.86% (64.28 to 129.47)	1632.9 (1119.2 to 2259.8)	1223 (809.4 to 1716.8)	-1.11% (-1.18 to -1.04)
Burundi	2016 (1328 to 2809)	4201 (2858 to 5917)	108.36% (75.11 to 142.45)	1930.9 (1271.9 to 2689.9)	1818.6 (1237.4 to 2561.6)	-0.06% (-0.11 to -0.01)
Cabo Verde	58 (40 to 80)	68 (47 to 93)	16.22% (-1.09 to 35.8)	1094.7 (745.3 to 1499.4)	450.8 (310 to 622.5)	-3.14% (-3.26 to -3.03)
Cambodia	2099 (1379 to 3055)	940 (607 to 1374)	-55.24% (-61.81 to -46.28)	912.6 (599.7 to 1328.1)	202.4 (130.8 to 295.9)	-5.8% (-6.06 to -5.54)
Cameroon	2515 (1750 to 3535)	3943 (2616 to 5452)	56.78% (33.11 to 83.66)	1249.6 (869.4 to 1756.1)	612.1 (406.1 to 846.5)	-2.16% (-2.41 to -1.91)
Central African Republic	704 (485 to 981)	1139 (756 to 1608)	61.83% (38.93 to 89.67)	1258.3 (867.6 to 1753.8)	922.2 (611.7 to 1301.4)	-1.01% (-1.05 to -0.96)
Chad	1457 (977 to 2061)	3829 (2567 to 5352)	162.84% (125.96 to 210.23)	1261.9 (846.5 to 1785.6)	1267.3 (849.5 to 1771.3)	0.21% (0.12 to 0.31)
China	23798 (15631 to 34193)	34837 (23011 to 49784)	46.39% (38.95 to 54.42)	74.4 (48.9 to 106.9)	70 (46.2 to 100)	3.33% (1.84 to 4.84)
Colombia	3760 (2679 to 5170)	3039 (2132 to 4231)	-19.15% (-30.93 to -4.91)	465 (331.3 to 639.5)	195.3 (137 to 271.9)	-3.33% (-3.53 to -3.12)

Location	Incident cases in 1990 (95% UI)	Incident cases in 2019 (95% UI)	Relative change (%, 95 CI)	ASIR in 1990 (95% UI)	ASIR in 2019 (95% UI)	EAPC (%, 95 CI)
Comoros	162 (110 to 231)	128 (85 to 181)	-21.32% (-35.84 to -4.31)	1788 (1211.1 to 2541.5)	627.6 (418.8 to 889.3)	-3.66% (-3.76 to -3.55)
Congo	550 (368 to 766)	984 (666 to 1386)	78.9% (52.01 to 112.98)	1261.4 (844.1 to 1756.8)	635.7 (430 to 895.2)	-2.06% (-2.18 to -1.95)
Costa Rica	272 (190 to 376)	255 (180 to 347)	-6% (-19.23 to 8.28)	364.4 (254.8 to 504)	158 (111.3 to 214.6)	-3.02% (-3.64 to -2.4)
Côte d'Ivoire	2988 (2001 to 4167)	4914 (3354 to 6948)	64.46% (40.44 to 90.97)	1393.1 (932.9 to 1942.8)	834.4 (569.6 to 1179.8)	-1.79% (-1.81 to -1.76)
Cuba	160 (109 to 224)	282 (195 to 401)	76.89% (50.5 to 106.09)	46.3 (31.5 to 65.1)	79.9 (55.1 to 113.5)	2.64% (2.38 to 2.9)
Democratic People's Republic of Korea	1159 (737 to 1679)	284 (181 to 414)	-75.52% (-79.26 to -70.58)	195.6 (124.4 to 283.4)	33.7 (21.5 to 49.2)	-6.94% (-7.39 to -6.48)
Democratic Republic of the Congo	12190 (8106 to 17103)	17891 (11937 to 25183)	46.77% (24.86 to 78.16)	1717.3 (1142 to 2409.4)	979.8 (653.7 to 1379.1)	-1.66% (-1.9 to -1.42)
Djibouti	125 (84 to 182)	295 (200 to 412)	135.53% (94.92 to 192.48)	1418.9 (950.4 to 2063.1)	788.7 (533.5 to 1100.6)	-2.17% (-2.35 to -1.99)
Dominica	3 (2 to 5)	3 (2 to 4)	-10.09% (-24.35 to 6.51)	219.4 (149.2 to 310.9)	163 (109.8 to 228.2)	-0.51% (-0.97 to -0.06)

Location	Incident cases in 1990 (95% UI)	Incident cases in 2019 (95% UI)	Relative change (%, 95 CI)	ASIR in 1990 (95% UI)	ASIR in 2019 (95% UI)	EAPC (%, 95 CI)
Dominican Republic	813 (552 to 1142)	280 (188 to 391)	-65.52% (-69.79 to -59.73)	500 (339.1 to 702.1)	84.4 (56.7 to 117.6)	-5.69% (-6.21 to -5.17)
Ecuador	1684 (1157 to 2326)	1427 (952 to 2014)	-15.25% (-27.93 to -1.09)	722.1 (496.1 to 997.2)	264.1 (176.1 to 372.5)	-3.42% (-3.63 to -3.21)
Egypt	3920 (2525 to 5690)	1873 (1234 to 2772)	-52.21% (-61.64 to -38.67)	297.9 (191.9 to 432.4)	68 (44.8 to 100.7)	-4.36% (-4.61 to -4.11)
El Salvador	693 (490 to 974)	461 (323 to 626)	-33.5% (-42.2 to -22)	593.8 (419.7 to 833.8)	214.2 (149.9 to 290.9)	-4.09% (-4.57 to -3.6)
Equatorial Guinea	135 (93 to 188)	162 (111 to 228)	19.63% (2.86 to 40.96)	1647.5 (1129.5 to 2293.4)	555.8 (381.5 to 781.8)	-3.84% (-4.08 to -3.6)
Eritrea	847 (562 to 1206)	1463 (952 to 2101)	72.59% (45.81 to 106.07)	1529.5 (1015 to 2177.2)	924 (601.2 to 1327.2)	-1.72% (-1.88 to -1.56)
Eswatini	151 (102 to 212)	123 (86 to 169)	-18.18% (-29.87 to -0.7)	934.3 (635.4 to 1316.2)	431.5 (300.7 to 591.9)	-2.39% (-2.5 to -2.27)
Ethiopia	15145 (10275 to 21294)	23209 (15774 to 32525)	53.25% (40.31 to 68.07)	1661.5 (1127.2 to 2336)	986.3 (670.3 to 1382.1)	-1.92% (-2.03 to -1.8)
Fiji	37 (25 to 53)	49 (34 to 70)	32.59% (11.62 to 57.48)	186.5 (124.8 to 267.1)	176.1 (121.2 to 250.5)	-0.06% (-0.27 to 0.14)
Gabon	168 (112 to 236)	237 (159 to 333)	41.14% (19.5 to 63.93)	1147.6 (766.7 to 1614.2)	470.7 (316.4 to 662.5)	-2.88% (-3.04 to -2.72)

Location	Incident cases in 1990 (95% UI)	Incident cases in 2019 (95% UI)	Relative change (%, 95 CI)	ASIR in 1990 (95% UI)	ASIR in 2019 (95% UI)	EAPC (%, 95 CI)
Gambia	285 (194 to 399)	515 (357 to 714)	80.4% (56.3 to 109.9)	1650.4 (1123.8 to 2306.7)	1016.8 (704.9 to 1410.9)	-1.67% (-1.83 to -1.51)
Georgia	80 (54 to 114)	145 (97 to 210)	81.46% (51.72 to 117.1)	52.9 (35.5 to 75.5)	119.7 (79.9 to 172.9)	3.47% (3.28 to 3.67)
Ghana	4639 (3211 to 6461)	6885 (4674 to 9452)	48.42% (26.63 to 75.14)	1523.7 (1054.7 to 2122)	787.8 (534.8 to 1081.6)	-2.15% (-2.21 to -2.09)
Global	431230 (300232 to 598296)	528481 (365267 to 735798)	22.55% (19.94 to 25.23)	307.8 (214.3 to 427)	216 (149.3 to 300.7)	-0.99% (-1.19 to -0.79)
Grenada	7 (5 to 10)	10 (7 to 15)	52.3% (30.69 to 77.77)	434.9 (296.9 to 608.4)	339.1 (230.4 to 475.2)	0.07% (-0.3 to 0.45)
Guatemala	2260 (1616 to 3155)	1808 (1277 to 2481)	-19.97% (-32.16 to -6.61)	1390.7 (994.3 to 1941.8)	345.9 (244.2 to 474.6)	-4.9% (-5.07 to -4.72)
Guinea	1900 (1312 to 2623)	2340 (1563 to 3281)	23.13% (5.73 to 45.4)	1377.8 (951.2 to 1902)	859.4 (574 to 1205.1)	-1.64% (-1.7 to -1.58)
Guinea-Bissau	304 (205 to 424)	428 (290 to 602)	40.74% (20.06 to 63.15)	1496.5 (1011.3 to 2088.8)	987.8 (670.8 to 1391.3)	-1.59% (-1.71 to -1.48)
Guyana	58 (40 to 81)	35 (23 to 49)	-39.95% (-48.59 to -29.9)	328.3 (224.7 to 459.5)	141.4 (95.4 to 199.4)	-2.62% (-2.8 to -2.45)
Haiti	1937 (1322 to 2695)	2527 (1747 to 3480)	30.49% (13.99 to 52.88)	1397 (953.3 to 1944.1)	674.2 (466 to 928.4)	-2.4% (-2.56 to -2.24)

Location	Incident cases in 1990 (95% UI)	Incident cases in 2019 (95% UI)	Relative change (%, 95 CI)	ASIR in 1990 (95% UI)	ASIR in 2019 (95% UI)	EAPC (%, 95 CI)
Honduras	966 (672 to 1329)	808 (575 to 1115)	-16.36% (-28.88 to -1.06)	1075.6 (748.9 to 1479.7)	288.9 (205.6 to 398.7)	-5.25% (-5.53 to -4.97)
India	89891 (62793 to 124498)	43654 (30388 to 61625)	-51.44% (-54.91 to -48.19)	439 (306.7 to 608.1)	102.6 (71.4 to 144.8)	-4.91% (-5.03 to -4.79)
Indonesia	14229 (9562 to 20192)	20598 (13805 to 29335)	44.76% (37.64 to 52.65)	320.9 (215.7 to 455.4)	213.7 (143.2 to 304.4)	-1.3% (-1.37 to -1.23)
Iran (Islamic Republic of)	6990 (4676 to 10203)	4409 (2933 to 6465)	-36.93% (-40.71 to -30.99)	642.8 (430 to 938.3)	140.8 (93.6 to 206.4)	-3.05% (-4.75 to -1.33)
Iraq	2158 (1429 to 3132)	1543 (1018 to 2237)	-28.48% (-42.3 to -8.75)	641 (424.6 to 930.3)	136.3 (89.9 to 197.5)	-5.45% (-5.85 to -5.05)
Jamaica	132 (91 to 181)	212 (144 to 296)	60.6% (37.74 to 87.31)	266.4 (183.4 to 365.6)	239 (161.8 to 333.5)	0.19% (-0.14 to 0.51)
Jordan	328 (213 to 478)	469 (322 to 654)	43.32% (12.8 to 84.44)	508.3 (330.7 to 742)	149.6 (102.5 to 208.5)	-4.46% (-4.7 to -4.23)
Kazakhstan	547 (352 to 791)	1031 (658 to 1485)	88.5% (57.85 to 132.1)	133.4 (85.7 to 192.8)	163.8 (104.5 to 235.9)	2.72% (1.9 to 3.56)
Kenya	4674 (3197 to 6547)	4055 (2776 to 5684)	-13.24% (-15.64 to -10.69)	1216 (831.7 to 1703.6)	329.6 (225.7 to 462.1)	-4.43% (-4.58 to -4.29)
Kiribati	8 (5 to 11)	7 (5 to 10)	-8.18% (-22.31 to 7.79)	450.8 (301 to 634.9)	205 (140.3 to 292.9)	-2.69% (-2.87 to -2.51)

Location	Incident cases in 1990 (95% UI)	Incident cases in 2019 (95% UI)	Relative change (%, 95 CI)	ASIR in 1990 (95% UI)	ASIR in 2019 (95% UI)	EAPC (%, 95 CI)
Kyrgyzstan	232 (154 to 338)	332 (216 to 472)	42.8% (15.46 to 71.97)	276.4 (183.1 to 402)	178.8 (116.4 to 254.6)	0.47% (-0.21 to 1.16)
Lao People's Democratic Republic	630 (412 to 917)	472 (308 to 688)	-25.07% (-37.66 to -10.22)	728.1 (476.4 to 1059.9)	234.9 (153.3 to 342.3)	-4.68% (-5.13 to -4.23)
Lebanon	202 (131 to 297)	96 (63 to 139)	-52.61% (-61.68 to -40.26)	232.2 (150.5 to 341.8)	54.5 (36 to 79.6)	-5.46% (-5.7 to -5.21)
Lesotho	397 (272 to 552)	275 (189 to 386)	-30.64% (-40.5 to -18.95)	1063.3 (729 to 1477.8)	501.5 (344.7 to 703.3)	-2.47% (-2.68 to -2.27)
Liberia	592 (399 to 828)	936 (641 to 1326)	58.28% (36.89 to 87.03)	1503.4 (1014.2 to 2104.9)	738.6 (505.5 to 1046.2)	-2.47% (-2.7 to -2.24)
Libya	570 (371 to 839)	791 (508 to 1186)	38.71% (10.38 to 71.64)	833 (541.6 to 1225.8)	284 (182.5 to 425.6)	-3.31% (-3.61 to -3.01)
Madagascar	2756 (1811 to 3889)	4124 (2781 to 5924)	49.66% (26.18 to 80.69)	1258.1 (826.9 to 1775.7)	643.5 (434 to 924.4)	-2.43% (-2.53 to -2.32)
Malawi	2686 (1787 to 3847)	2391 (1585 to 3357)	-10.98% (-24.85 to 5.43)	1422.8 (946.7 to 2037.9)	615.5 (408 to 864.2)	-2.94% (-3.19 to -2.7)
Malaysia	2148 (1420 to 3066)	2283 (1470 to 3332)	6.26% (-9.92 to 27.76)	475.1 (313.9 to 677.9)	242.1 (156 to 353.4)	-2.67% (-2.88 to -2.46)

Location	Incident cases in 1990 (95% UI)	Incident cases in 2019 (95% UI)	Relative change (%, 95 CI)	ASIR in 1990 (95% UI)	ASIR in 2019 (95% UI)	EAPC (%, 95 CI)
Maldives	26 (16 to 38)	33 (21 to 50)	25.06% (2.93 to 49.12)	780.8 (487.1 to 1137.7)	240.5 (156.9 to 363.6)	-3.82% (-4.39 to -3.25)
Mali	3040 (2066 to 4231)	5602 (3791 to 7838)	84.25% (59.79 to 114.71)	1685.5 (1145.5 to 2345.5)	1345.1 (910.4 to 1882.1)	-0.75% (-0.79 to -0.72)
Marshall Islands	3 (2 to 4)	3 (2 to 4)	13.59% (-5.23 to 36.79)	318.7 (212.7 to 458.5)	168.4 (112.2 to 242.8)	-1.56% (-1.85 to -1.27)
Mauritania	672 (455 to 936)	889 (615 to 1256)	32.25% (14.68 to 55.98)	1648.4 (1115.7 to 2295.6)	931.3 (644.3 to 1316.6)	-1.96% (-2.11 to -1.81)
Mexico	10301 (7125 to 14576)	7892 (5262 to 11524)	-23.39% (-30.1 to -17.42)	529.2 (366.1 to 748.8)	180.8 (120.5 to 264)	-3.7% (-4.22 to -3.17)
Micronesia (Federated States of)	16 (11 to 24)	6 (4 to 9)	-61.26% (-67.92 to -53.84)	842 (561 to 1226.7)	222.4 (148.4 to 320.4)	-4.99% (-5.46 to -4.51)
Mongolia	159 (101 to 227)	309 (205 to 445)	93.92% (59.84 to 139.26)	446.2 (284.3 to 637.5)	250.9 (166.4 to 361.6)	-0.42% (-1.58 to 0.75)
Montenegro	13 (8 to 19)	14 (9 to 20)	7.26% (-14.5 to 36.52)	69.9 (44.2 to 101.7)	64.3 (42.6 to 92.2)	0.25% (0.07 to 0.43)
Morocco	3638 (2366 to 5377)	3813 (2436 to 5668)	4.8% (-16.31 to 28.85)	646 (420.2 to 954.6)	311.9 (199.2 to 463.6)	-2.85% (-3.19 to -2.51)

Location	Incident cases in 1990 (95% UI)	Incident cases in 2019 (95% UI)	Relative change (%, 95 CI)	ASIR in 1990 (95% UI)	ASIR in 2019 (95% UI)	EAPC (%, 95 CI)
Mozambique	3894 (2604 to 5622)	5707 (3870 to 8239)	46.57% (22.93 to 71.01)	1294.7 (865.9 to 1869.4)	930.7 (631.1 to 1343.5)	-1.12% (-1.28 to -0.96)
Myanmar	6541 (4173 to 9470)	4957 (3275 to 7255)	-24.22% (-35.36 to -11.17)	736.1 (469.6 to 1065.7)	262.5 (173.4 to 384.1)	-3.61% (-3.7 to -3.52)
Namibia	350 (245 to 497)	358 (244 to 502)	2.49% (-12.57 to 20.2)	1283.4 (900.7 to 1822.8)	540.6 (368.3 to 757.1)	-2.75% (-2.92 to -2.58)
Nepal	2245 (1530 to 3138)	595 (402 to 847)	-73.5% (-78.42 to -68.14)	519.6 (354.2 to 726.6)	64.1 (43.3 to 91.4)	-7.81% (-8.19 to -7.44)
Nicaragua	428 (302 to 578)	326 (226 to 450)	-23.78% (-34.44 to -11.13)	583.7 (412.4 to 788.8)	163.9 (113.8 to 226.3)	-4.31% (-4.69 to -3.93)
Niger	2581 (1815 to 3581)	6649 (4477 to 9233)	157.57% (120.92 to 198.32)	1636.1 (1150.2 to 2269.5)	1637.6 (1102.6 to 2273.9)	0.07% (0 to 0.15)
Nigeria	26015 (18247 to 35292)	50959 (36066 to 68979)	95.88% (89.36 to 103.25)	1698.8 (1191.5 to 2304.5)	1058.6 (749.2 to 1432.9)	-1.56% (-1.65 to -1.46)
North Macedonia	20 (14 to 30)	27 (18 to 39)	30.19% (5.6 to 68.96)	31.8 (21.2 to 46.2)	34.9 (23.2 to 50.8)	1.05% (0.67 to 1.43)
Pakistan	19418 (13550 to 26951)	20315 (14358 to 28026)	4.62% (-4.11 to 14.7)	851.2 (594 to 1181.5)	363.4 (256.8 to 501.3)	-2.99% (-3.15 to -2.82)

Location	Incident cases in 1990 (95% UI)	Incident cases in 2019 (95% UI)	Relative change (%, 95 CI)	ASIR in 1990 (95% UI)	ASIR in 2019 (95% UI)	EAPC (%, 95 CI)
Papua New Guinea	708 (474 to 1024)	1860 (1226 to 2699)	162.81% (117.44 to 211.65)	798.4 (535 to 1155.3)	685.8 (452.1 to 994.8)	-0.58% (-0.61 to -0.55)
Paraguay	669 (455 to 925)	540 (375 to 727)	-19.33% (-32.21 to -3.3)	786.7 (535.8 to 1088.2)	269.6 (187.5 to 363.1)	-4.08% (-4.28 to -3.88)
Peru	4397 (3095 to 6104)	3454 (2332 to 4862)	-21.46% (-34 to -8.52)	863.7 (608 to 1198.9)	299.2 (202 to 421.1)	-3.61% (-3.71 to -3.51)
Philippines	10494 (6937 to 15007)	12076 (8055 to 17286)	15.08% (10.05 to 20.08)	735.2 (486.1 to 1051.4)	379.2 (252.9 to 542.7)	-2.19% (-2.37 to -2.01)
Republic of Moldova	153 (99 to 226)	129 (85 to 191)	-15.92% (-28.23 to -1.99)	123.8 (80.3 to 183.1)	92.5 (61.2 to 137.3)	0.53% (-0.15 to 1.22)
Russian Federation	4204 (2813 to 6055)	11185 (7435 to 16045)	166.05% (158.79 to 172.61)	87.9 (58.8 to 126.7)	203 (134.9 to 291.2)	5.47% (4.32 to 6.63)
Rwanda	2243 (1477 to 3158)	2868 (1900 to 4084)	27.85% (8.01 to 50.11)	1849 (1217.8 to 2603)	893.6 (592 to 1272.5)	-2.78% (-2.93 to -2.63)
Saint Lucia	10 (7 to 14)	12 (8 to 17)	24.62% (6.01 to 46)	335.1 (230.3 to 470.4)	187 (126.2 to 260.2)	-1.37% (-1.7 to -1.04)
Saint Vincent and the Grenadines	5 (4 to 8)	8 (5 to 11)	47.66% (23.37 to 74.45)	266 (184 to 367.9)	208.1 (138.1 to 294.9)	-0.58% (-0.96 to -0.2)

Location	Incident cases in 1990 (95% UI)	Incident cases in 2019 (95% UI)	Relative change (%, 95 CI)	ASIR in 1990 (95% UI)	ASIR in 2019 (95% UI)	EAPC (%, 95 CI)
Samoa	5<br>(3 to 7)	9<br>(6 to 12)	72.1%<br>(43.95 to 109.88)	165.4<br>(111.1 to 232.5)	172.1<br>(115.4 to 246.2)	0.46%<br>(0.34 to 0.57)
Senegal	2331<br>(1562 to 3271)	3613<br>(2453 to 5081)	54.99%<br>(32 to 84.38)	1565.9<br>(1049.6 to 2197.5)	1037<br>(704.2 to 1458.4)	-1.12%<br>(-1.33 to -0.91)
Serbia	79<br>(53 to 114)	101<br>(67 to 146)	26.65%<br>(4.03 to 56.64)	28<br>(18.5 to 40.2)	33.2<br>(22.2 to 48.1)	2.87%<br>(2.1 to 3.65)
Sierra Leone	1056<br>(702 to 1463)	1521<br>(1041 to 2135)	44.06%<br>(23.03 to 69.06)	1316.6<br>(875.7 to 1825.3)	824.3<br>(564 to 1157.3)	-1.59%<br>(-1.76 to -1.43)
Solomon Islands	54<br>(36 to 78)	107<br>(71 to 155)	96.88%<br>(61.76 to 131.03)	901.6<br>(603 to 1291.5)	587.7<br>(390 to 850)	-1.6%<br>(-1.65 to -1.54)
Somalia	2874<br>(1947 to 4114)	6942<br>(4591 to 9966)	141.56%<br>(105.06 to 188.35)	1752.8<br>(1187.3 to 2509.2)	1483.9<br>(981.4 to 2130.4)	-0.49%<br>(-0.53 to -0.44)
South Africa	4675<br>(3211 to 6432)	5345<br>(3684 to 7389)	14.33%<br>(5.66 to 21.54)	530.6<br>(364.5 to 730)	306<br>(210.9 to 423)	-1.86%<br>(-1.9 to -1.83)
South Sudan	1549<br>(1035 to 2156)	2833<br>(1917 to 3902)	82.85%<br>(54.59 to 119.34)	1412.3<br>(943.8 to 1965.2)	1224.1<br>(828.5 to 1685.9)	-0.49%<br>(-0.52 to -0.46)
Sri Lanka	953<br>(620 to 1378)	1138<br>(728 to 1655)	19.36%<br>(1.28 to 39.06)	189.4<br>(123.1 to 273.8)	147.6<br>(94.5 to 214.8)	-0.73%<br>(-1.15 to -0.32)

Location	Incident cases in 1990 (95% UI)	Incident cases in 2019 (95% UI)	Relative change (%, 95 CI)	ASIR in 1990 (95% UI)	ASIR in 2019 (95% UI)	EAPC (%, 95 CI)
Sudan	3214 (2133 to 4770)	3544 (2385 to 5064)	10.26% (-10.07 to 38.2)	791 (524.9 to 1174.1)	341.6 (229.9 to 488.2)	-2.67% (-3.04 to -2.29)
Suriname	20 (14 to 27)	37 (25 to 52)	90.6% (61.85 to 121.81)	204.4 (141.3 to 283.4)	202.4 (136.6 to 285.2)	-0.03% (-0.23 to 0.16)
Syrian Arab Republic	1193 (785 to 1731)	832 (545 to 1220)	-30.28% (-44.34 to -14.12)	554.7 (364.7 to 804.7)	163.8 (107.4 to 240.2)	-4.5% (-5.69 to -3.3)
Tajikistan	422 (277 to 601)	495 (318 to 721)	17.3% (-1.46 to 36.64)	537.5 (352.7 to 765.9)	204.4 (131.3 to 298.1)	-2.65% (-3.08 to -2.23)
Thailand	2421 (1539 to 3501)	2493 (1610 to 3613)	2.98% (-15.67 to 22.56)	152.5 (97 to 220.6)	87.2 (56.4 to 126.5)	-1.94% (-2.32 to -1.56)
Timor-Leste	198 (127 to 291)	191 (125 to 277)	-3.78% (-18.11 to 13.26)	1121.9 (717.6 to 1648.6)	651.7 (426.4 to 948.5)	-2.01% (-2.53 to -1.5)
Togo	1130 (749 to 1560)	1704 (1179 to 2403)	50.8% (30.09 to 72.28)	1534.2 (1016.7 to 2118.9)	795.7 (550.6 to 1122.6)	-2.1% (-2.2 to -2)
Tonga	13 (9 to 19)	12 (8 to 18)	-9.6% (-26.4 to 8.08)	646.9 (437.8 to 931.4)	436.4 (280.5 to 634.8)	-1.43% (-1.53 to -1.34)
Tunisia	592 (386 to 880)	882 (561 to 1332)	49.04% (20.58 to 87.44)	325.5 (212 to 483.7)	197.7 (125.8 to 298.7)	-0.95% (-1.49 to -0.41)
Turkey	3470 (2263 to 5024)	4264 (2676 to 6113)	22.89% (4.2 to 45.08)	244.7 (159.6 to 354.4)	136.3 (85.5 to 195.4)	-1.24% (-1.69 to -0.79)

Location	Incident cases in 1990 (95% UI)	Incident cases in 2019 (95% UI)	Relative change (%, 95 CI)	ASIR in 1990 (95% UI)	ASIR in 2019 (95% UI)	EAPC (%, 95 CI)
Turkmenistan	271<br>(179 to 392)	172<br>(115 to 246)	-36.82%<br>(-47.17 to -23.04)	474.7<br>(313.7 to 684.8)	112.4<br>(75.2 to 161.1)	-4.69%<br>(-5.38 to -3.99)
Tuvalu	2<br>(1 to 2)	1<br>(0 to 1)	-55.49%<br>(-62.31 to -47.57)	539.5<br>(361.1 to 788)	222.1<br>(146.1 to 314.1)	-3.43%<br>(-3.57 to -3.29)
Uganda	4103<br>(2816 to 5790)	6875<br>(4659 to 9642)	67.56%<br>(42.92 to 98.26)	1472.1<br>(1010.3 to 2077.6)	861<br>(583.5 to 1207.6)	-1.79%<br>(-2.03 to -1.55)
Ukraine	1229<br>(824 to 1779)	2321<br>(1516 to 3379)	88.89%<br>(62.93 to 117.19)	77<br>(51.7 to 111.6)	137.5<br>(89.8 to 200.3)	4.18%<br>(3.09 to 5.29)
United Republic of Tanzania	6306<br>(4192 to 8802)	12105<br>(7850 to 17366)	91.96%<br>(65.02 to 125.89)	1375.7<br>(914.5 to 1920.2)	906.5<br>(587.9 to 1300.5)	-1.22%<br>(-1.33 to -1.1)
Uzbekistan	715<br>(464 to 1049)	575<br>(384 to 823)	-19.56%<br>(-32.45 to -2.21)	253.2<br>(164.2 to 371.2)	55.3<br>(36.9 to 79.1)	-4.97%<br>(-5.51 to -4.43)
Vanuatu	20<br>(13 to 28)	25<br>(17 to 37)	29.21%<br>(8.61 to 54.41)	663.2<br>(446 to 964.2)	348.3<br>(237.8 to 511.2)	-2.34%<br>(-2.44 to -2.23)
Venezuela (Bolivarian Republic of)	1946<br>(1357 to 2671)	1861<br>(1345 to 2559)	-4.39%<br>(-19.23 to 12.95)	419.1<br>(292.3 to 575.2)	183.8<br>(132.8 to 252.8)	-2.43%<br>(-2.68 to -2.17)
Viet Nam	3557<br>(2290 to 5182)	2915<br>(1860 to 4257)	-18.05%<br>(-32.87 to -2.15)	288.8<br>(185.9 to 420.7)	83.2<br>(53.1 to 121.5)	-4.43%<br>(-4.98 to -3.88)

Location	Incident cases in 1990 (95% UI)	Incident cases in 2019 (95% UI)	Relative change (%, 95 CI)	ASIR in 1990 (95% UI)	ASIR in 2019 (95% UI)	EAPC (%, 95 CI)
Yemen	2105 (1340 to 3140)	3213 (2100 to 4693)	52.66% (20.25 to 92.89)	965.9 (614.8 to 1440.9)	418 (273.3 to 610.5)	-2.94% (-3.04 to -2.85)
Zambia	2039 (1383 to 2929)	2754 (1846 to 3895)	35.03% (14.31 to 60.66)	1460.6 (990.3 to 2097.5)	696.8 (467.2 to 985.6)	-2.34% (-2.55 to -2.14)
Zimbabwe	1976 (1366 to 2770)	1655 (1133 to 2311)	-16.24% (-29.56 to -0.13)	1077.7 (745.1 to 1511.2)	425.5 (291.4 to 594.2)	-2.7% (-2.88 to -2.52)

ASIR – age-standardized incidence rate (per 100,000 population), CI – confidence interval, EAPC – estimated annual percentage change, MSMI – maternal sepsis and other maternal infections, UI – uncertain interval.

Table S14. Incidence of MSMI in 131 low- and middle-income countries and territories with EAPC in age group of 45-49 years from 1990 and 2019.

Location	Incident cases in 1990 (95% UI)	Incident cases in 2019 (95% UI)	Relative change (%, 95 CI)	ASIR in 1990 (95% UI)	ASIR in 2019 (95% UI)	EAPC (%, 95 CI)
Afghanistan	906 (513 to 1415)	2207 (1314 to 3407)	143.65% (100.83 to 204.03)	374.4 (212.2 to 585)	269.4 (160.4 to 415.8)	-1.21% (-1.36 to -1.06)
Albania	12 (7 to 18)	3 (2 to 5)	-72.92% (-78.09 to -66.91)	20 (11.4 to 30.4)	3.7 (2.1 to 5.8)	-5.83% (-6.66 to -4.98)
Algeria	1142 (650 to 1781)	1187 (699 to 1820)	3.98% (-14.04 to 27.39)	295.8 (168.4 to 461.3)	97.5 (57.4 to 149.5)	-3.75% (-4.99 to -2.49)
American Samoa	1 (0 to 1)	0 (0 to 0)	-54.45% (-61.84 to -45.71)	75.5 (47.7 to 112.8)	18.6 (11.7 to 28.3)	-4.92% (-5.02 to -4.81)
Angola	1140 (698 to 1601)	1375 (867 to 1969)	20.68% (1.77 to 40.51)	678.7 (415.7 to 953.4)	262.7 (165.6 to 376.1)	-3.02% (-3.25 to -2.79)
Armenia	12 (7 to 17)	11 (7 to 16)	-3.64% (-19.13 to 14.91)	19 (11 to 28.1)	12.6 (7.7 to 18.2)	-0.96% (-1.79 to -0.13)
Azerbaijan	17 (10 to 25)	14 (8 to 21)	-19.71% (-32.38 to -3.8)	14.5 (8.5 to 21.5)	4.2 (2.5 to 6.3)	-3.22% (-4.05 to -2.37)
Bangladesh	2560 (1623 to 3841)	2121 (1288 to 3127)	-17.15% (-30.53 to -2.54)	163.9 (103.9 to 246)	47.9 (29.1 to 70.6)	-2.99% (-3.41 to -2.56)

Location	Incident cases in 1990 (95% UI)	Incident cases in 2019 (95% UI)	Relative change (%, 95 CI)	ASIR in 1990 (95% UI)	ASIR in 2019 (95% UI)	EAPC (%, 95 CI)
Belarus	10 (7 to 13)	24 (17 to 30)	138.69% (108.1 to 176.27)	3.8 (2.7 to 4.8)	7.1 (5.1 to 8.9)	2.66% (1.91 to 3.42)
Belize	3 (2 to 4)	4 (2 to 5)	16.4% (-0.38 to 35.09)	123.3 (80.6 to 162.6)	33.2 (21.6 to 43.6)	-4.38% (-4.53 to -4.23)
Benin	540 (342 to 758)	1022 (659 to 1427)	89.28% (62.17 to 120.91)	730.6 (462.8 to 1025.6)	483.6 (311.7 to 675.3)	-1.15% (-1.27 to -1.03)
Bhutan	28 (18 to 42)	17 (10 to 24)	-41.79% (-51.53 to -30.73)	307.4 (190.1 to 456)	92.7 (57.5 to 134.9)	-4.15% (-4.41 to -3.89)
Bolivia (Plurinational State of)	523 (358 to 677)	510 (334 to 666)	-2.5% (-15.2 to 13.44)	438.4 (300 to 568)	176 (115.2 to 229.9)	-2.91% (-3.19 to -2.64)
Bosnia and Herzegovina	8 (5 to 12)	4 (2 to 6)	-50.12% (-58.95 to -39.22)	6.9 (4.2 to 10.6)	3.5 (2.1 to 5.3)	-1.85% (-2.22 to -1.49)
Botswana	115 (74 to 163)	65 (41 to 88)	-43.92% (-52.07 to -33.6)	553 (354.9 to 781.1)	111 (71 to 152.2)	-5.09% (-5.33 to -4.85)
Brazil	4605 (3497 to 5448)	2106 (1471 to 2719)	-54.26% (-60.08 to -48.32)	147.6 (112.1 to 174.6)	29.9 (20.9 to 38.6)	-4.83% (-5.14 to -4.52)

Location	Incident cases in 1990 (95% UI)	Incident cases in 2019 (95% UI)	Relative change (%, 95 CI)	ASIR in 1990 (95% UI)	ASIR in 2019 (95% UI)	EAPC (%, 95 CI)
Bulgaria	3 (2 to 5)	24 (14 to 38)	703.33% (560.12 to 869.69)	1.1 (0.7 to 1.8)	9.6 (5.6 to 15.1)	8.83% (7.18 to 10.5)
Burkina Faso	1553 (978 to 2125)	2273 (1435 to 3168)	46.41% (22.34 to 74.76)	947.3 (597 to 1296.5)	565.7 (357.1 to 788.4)	-2.04% (-2.13 to -1.95)
Burundi	649 (394 to 921)	924 (575 to 1324)	42.35% (17.55 to 68.85)	787.3 (477.8 to 1117.1)	532 (331 to 762.6)	-1.12% (-1.26 to -0.98)
Cabo Verde	20 (13 to 28)	6 (4 to 9)	-68.92% (-73.87 to -63.05)	413.7 (258.3 to 579.1)	47.9 (30.9 to 65.5)	-7.25% (-7.77 to -6.72)
Cambodia	480 (303 to 748)	223 (136 to 345)	-53.57% (-61.02 to -44.68)	270.8 (170.9 to 421.8)	54.7 (33.5 to 84.8)	-6.12% (-6.39 to -5.85)
Cameroon	922 (583 to 1282)	1094 (699 to 1462)	18.55% (1.92 to 41.02)	544 (343.9 to 755.8)	220.9 (141.2 to 295.4)	-2.67% (-3.1 to -2.25)
Central African Republic	278 (176 to 396)	319 (200 to 451)	14.96% (-1.21 to 36.96)	568 (359.3 to 809.8)	323 (202.7 to 456.3)	-1.84% (-1.98 to -1.71)
Chad	566 (359 to 788)	1377 (888 to 1915)	143.26% (108.04 to 183.95)	581.8 (369.1 to 810)	588.8 (379.8 to 819.1)	0.38% (0.23 to 0.53)

Location	Incident cases in 1990 (95% UI)	Incident cases in 2019 (95% UI)	Relative change (%, 95 CI)	ASIR in 1990 (95% UI)	ASIR in 2019 (95% UI)	EAPC (%, 95 CI)
China	7889 (4900 to 12084)	27491 (16700 to 41896)	248.45% (229.52 to 269.38)	32.3 (20.1 to 49.5)	46.2 (28.1 to 70.4)	4.69% (3.02 to 6.39)
Colombia	1135 (821 to 1383)	533 (391 to 649)	-53.07% (-59.15 to -45.33)	179.9 (130.2 to 219.3)	36.1 (26.5 to 44)	-5.38% (-5.65 to -5.12)
Comoros	77 (52 to 104)	28 (17 to 38)	-64.18% (-70.05 to -57.29)	1012.7 (675.7 to 1354.7)	156.9 (98.3 to 215.9)	-6.07% (-6.66 to -5.47)
Congo	231 (145 to 330)	152 (95 to 219)	-34.28% (-44.3 to -24.67)	604.2 (379.4 to 863.1)	126 (79 to 181.8)	-4.93% (-5.38 to -4.48)
Costa Rica	37 (26 to 46)	22 (16 to 27)	-39.97% (-47.59 to -30.16)	68.2 (47.4 to 84.9)	15.6 (11.3 to 19.1)	-5.07% (-5.61 to -4.53)
Côte d'Ivoire	1410 (891 to 1972)	1932 (1214 to 2663)	37.07% (17.11 to 64.89)	822.2 (519.6 to 1150.4)	441 (277.1 to 607.8)	-2.18% (-2.28 to -2.08)
Cuba	32 (22 to 42)	18 (12 to 23)	-44.37% (-52.77 to -35.7)	10.7 (7.2 to 13.9)	3.7 (2.4 to 4.8)	-2.67% (-3.16 to -2.17)
Democratic People's Republic of Korea	337 (205 to 514)	46 (28 to 70)	-86.45% (-88.42 to -84.02)	52 (31.6 to 79.2)	4.5 (2.7 to 7)	-8.4% (-8.65 to -8.15)

Location	Incident cases in 1990 (95% UI)	Incident cases in 2019 (95% UI)	Relative change (%, 95 CI)	ASIR in 1990 (95% UI)	ASIR in 2019 (95% UI)	EAPC (%, 95 CI)
Democratic Republic of the Congo	5102 (3191 to 7243)	4200 (2558 to 6107)	-17.69% (-30.26 to -1.98)	826.3 (516.7 to 1173)	281.6 (171.5 to 409.5)	-3.35% (-3.85 to -2.84)
Djibouti	60 (38 to 86)	118 (75 to 165)	95.78% (62.99 to 135.05)	904.1 (570.7 to 1295.7)	426.3 (271.1 to 594.1)	-2.5% (-2.85 to -2.16)
Dominica	0 (0 to 1)	0 (0 to 0)	-36.12% (-45.21 to -24.28)	32.3 (20.3 to 42.5)	14.3 (9.3 to 19.1)	-2.43% (-2.58 to -2.28)
Dominican Republic	128 (85 to 169)	55 (36 to 72)	-57.32% (-62.46 to -49.97)	101.7 (67.7 to 134.3)	18.5 (12.3 to 24.2)	-5.57% (-6.14 to -5)
Ecuador	406 (277 to 519)	191 (131 to 241)	-53.03% (-59.43 to -44.21)	221.3 (150.9 to 283.1)	40.3 (27.7 to 51)	-6.26% (-6.43 to -6.09)
Egypt	806 (492 to 1268)	420 (260 to 641)	-47.92% (-56.55 to -36.01)	72.6 (44.3 to 114.2)	18.7 (11.6 to 28.6)	-3.92% (-4.4 to -3.45)
El Salvador	193 (135 to 241)	45 (31 to 57)	-76.7% (-79.72 to -72.82)	195.2 (136.7 to 243)	23.8 (16.4 to 29.9)	-7.42% (-8.1 to -6.74)
Equatorial Guinea	63 (38 to 89)	18 (11 to 26)	-71.73% (-75.62 to -66.98)	755.3 (459.4 to 1071)	77 (47.5 to 112.1)	-7.78% (-8.4 to -7.16)

Location	Incident cases in 1990 (95% UI)	Incident cases in 2019 (95% UI)	Relative change (%, 95 CI)	ASIR in 1990 (95% UI)	ASIR in 2019 (95% UI)	EAPC (%, 95 CI)
Eritrea	442 (275 to 630)	522 (325 to 755)	18.12% (-2.33 to 38.55)	857.5 (532.7 to 1221.4)	414.6 (258 to 599.6)	-2.32% (-2.6 to -2.04)
Eswatini	50 (30 to 73)	19 (12 to 26)	-62.55% (-67.85 to -54.98)	384.8 (235.5 to 564)	84.4 (54.9 to 117.7)	-4.78% (-4.93 to -4.63)
Ethiopia	6030 (3824 to 8576)	6040 (3811 to 8711)	0.17% (-9.11 to 8.48)	767 (486.5 to 1090.9)	363.5 (229.3 to 524.3)	-2.41% (-2.66 to -2.16)
Fiji	6 (4 to 10)	4 (3 to 7)	-31.19% (-41.39 to -18.88)	39.6 (25 to 60.9)	18.2 (11.5 to 27.3)	-2.88% (-3.01 to -2.75)
Gabon	72 (45 to 103)	18 (11 to 25)	-75.35% (-79.15 to -71.13)	444.9 (277 to 631.1)	41.6 (25.5 to 59.3)	-7.58% (-7.9 to -7.25)
Gambia	108 (66 to 153)	127 (84 to 175)	18.27% (2.45 to 41.53)	858.5 (528.2 to 1218.6)	347.6 (229.1 to 477.6)	-2.95% (-3.28 to -2.63)
Georgia	10 (6 to 15)	25 (15 to 37)	142% (97.84 to 189)	7.4 (4.5 to 11)	21 (12.6 to 31.2)	3.79% (3.14 to 4.45)
Ghana	2063 (1286 to 2857)	1834 (1156 to 2511)	-11.1% (-24.08 to 4.68)	844.1 (526.3 to 1169.3)	262.9 (165.8 to 360)	-3.54% (-3.9 to -3.19)

Location	Incident cases in 1990 (95% UI)	Incident cases in 2019 (95% UI)	Relative change (%, 95 CI)	ASIR in 1990 (95% UI)	ASIR in 2019 (95% UI)	EAPC (%, 95 CI)
Global	157796 (102029 to 225593)	166584 (107500 to 236628)	5.57% (3.15 to 8.09)	138.6 (89.6 to 198.1)	70.8 (45.7 to 100.5)	-1.87% (-2.12 to -1.63)
Grenada	1 (1 to 1)	0 (0 to 0)	-59.8% (-65.27 to -53.25)	64 (43.6 to 83.3)	10.3 (6.8 to 13.5)	-5.99% (-6.59 to -5.38)
Guatemala	599 (441 to 728)	360 (256 to 446)	-39.95% (-48.88 to -29.83)	470.3 (345.9 to 571.8)	89.6 (63.8 to 111.1)	-6.37% (-6.85 to -5.88)
Guinea	854 (542 to 1178)	1098 (711 to 1524)	28.48% (10.46 to 50.51)	789.1 (500.9 to 1088.1)	515.6 (334 to 716)	-1.51% (-1.57 to -1.44)
Guinea-Bissau	166 (106 to 229)	237 (149 to 325)	42.43% (22.01 to 68.82)	1020.5 (652.5 to 1404.9)	730.9 (460.2 to 1004)	-1.16% (-1.28 to -1.03)
Guyana	13 (9 to 17)	6 (4 to 8)	-54.43% (-61.2 to -46.32)	92.5 (61.7 to 122.2)	26.6 (16.9 to 35.1)	-4.8% (-5 to -4.59)
Haiti	578 (375 to 764)	545 (360 to 714)	-5.67% (-19.54 to 8.44)	507.2 (329.6 to 670.6)	191.9 (126.8 to 251.6)	-3.05% (-3.24 to -2.86)
Honduras	223 (156 to 275)	142 (103 to 172)	-36.41% (-45.94 to -24.1)	303.6 (212.4 to 374.8)	62.9 (45.8 to 76.5)	-5.94% (-6.19 to -5.69)
India	38542 (24739 to 57014)	18250 (11724 to 27068)	-52.65% (-55.55 to -49.65)	229.1 (147 to 338.9)	49.3 (31.6 to 73.1)	-5.27% (-5.53 to -5.01)

Location	Incident cases in 1990 (95% UI)	Incident cases in 2019 (95% UI)	Relative change (%, 95 CI)	ASIR in 1990 (95% UI)	ASIR in 2019 (95% UI)	EAPC (%, 95 CI)
Indonesia	4600<br>(2925 to 6881)	2616<br>(1668 to 3888)	-43.13%<br>(-46.37 to -39.93)	119.4<br>(75.9 to 178.6)	30.6<br>(19.5 to 45.4)	-3.81%<br>(-4.38 to -3.24)
Iran (Islamic Republic of)	2743<br>(1648 to 4199)	368<br>(222 to 566)	-86.6%<br>(-87.45 to -85.61)	336.8<br>(202.3 to 515.6)	14.3<br>(8.6 to 22)	-8.23%<br>(-9.63 to -6.82)
Iraq	619<br>(365 to 967)	311<br>(191 to 476)	-49.76%<br>(-58.83 to -36.98)	234.1<br>(138.1 to 365.6)	30.5<br>(18.7 to 46.6)	-7.27%<br>(-7.62 to -6.91)
Jamaica	21<br>(13 to 27)	34<br>(22 to 45)	65.6%<br>(42.51 to 88.18)	49.7<br>(32.4 to 65.4)	40.7<br>(26.3 to 53.3)	0.71%<br>(-0.09 to 1.52)
Jordan	83<br>(51 to 125)	37<br>(23 to 56)	-55.06%<br>(-63.23 to -43.39)	154.3<br>(94.9 to 233.7)	13.4<br>(8.3 to 20.1)	-8.22%<br>(-8.4 to -8.03)
Kazakhstan	58<br>(35 to 86)	87<br>(55 to 127)	50.68%<br>(24.88 to 81.53)	17.6<br>(10.7 to 26.3)	15.1<br>(9.4 to 21.9)	1.15%<br>(0.15 to 2.17)
Kenya	1956<br>(1254 to 2748)	807<br>(519 to 1125)	-58.73%<br>(-60.37 to -56.97)	629.2<br>(403.5 to 884)	84.3<br>(54.2 to 117.5)	-6.81%<br>(-6.91 to -6.7)
Kiribati	2<br>(1 to 3)	1<br>(1 to 2)	-47.21%<br>(-54.97 to -38.23)	137.3<br>(85.1 to 202.8)	37.5<br>(24.3 to 55.9)	-4.63%<br>(-4.79 to -4.46)
Kyrgyzstan	49<br>(29 to 72)	71<br>(43 to 105)	44.37%<br>(21.23 to 73.29)	78.1<br>(46.5 to 115.2)	41<br>(25.2 to 61.1)	-1.15%<br>(-1.71 to -0.58)

Location	Incident cases in 1990 (95% UI)	Incident cases in 2019 (95% UI)	Relative change (%, 95 CI)	ASIR in 1990 (95% UI)	ASIR in 2019 (95% UI)	EAPC (%, 95 CI)
Lao People's Democratic Republic	255 (161 to 401)	183 (111 to 277)	-28.29% (-40.76 to -14.97)	337.4 (212.8 to 531.4)	106 (64.4 to 160.7)	-4.72% (-5.28 to -4.15)
Lebanon	102 (61 to 157)	27 (16 to 43)	-73.5% (-78.56 to -67.46)	140.5 (84.5 to 217.1)	16.6 (10.1 to 26.3)	-7.49% (-7.74 to -7.24)
Lesotho	123 (77 to 175)	34 (22 to 48)	-72.39% (-76.51 to -68.03)	407.2 (255.4 to 577.6)	80.8 (51.8 to 113.5)	-5.25% (-5.48 to -5.02)
Liberia	261 (162 to 364)	236 (150 to 322)	-9.4% (-21.83 to 6.21)	803.3 (499.2 to 1122.9)	229.5 (146.1 to 313.3)	-4.22% (-4.65 to -3.79)
Libya	212 (123 to 332)	82 (48 to 124)	-61.36% (-68.1 to -53.37)	371.9 (215.8 to 581.3)	34.9 (20.6 to 52.9)	-6.92% (-7.63 to -6.2)
Madagascar	861 (525 to 1272)	693 (432 to 997)	-19.52% (-31.9 to -2.92)	487.5 (297.3 to 720.3)	133.4 (83.2 to 191.9)	-4.72% (-4.98 to -4.47)
Malawi	1433 (887 to 2135)	1500 (894 to 2228)	4.61% (-11.05 to 22.55)	995.8 (616 to 1483.2)	511.9 (305.3 to 760.6)	-2.19% (-2.48 to -1.9)
Malaysia	244 (149 to 373)	234 (143 to 358)	-4.18% (-18.09 to 13.96)	75.4 (45.8 to 115)	28.7 (17.5 to 43.9)	-3.74% (-4.08 to -3.39)
Maldives	11 (7 to 17)	3 (2 to 5)	-73.74% (-78.31 to -68.97)	338.7 (205.9 to 516.7)	27.3 (16.8 to 41.6)	-7.94% (-8.4 to -7.47)

Location	Incident cases in 1990 (95% UI)	Incident cases in 2019 (95% UI)	Relative change (%, 95 CI)	ASIR in 1990 (95% UI)	ASIR in 2019 (95% UI)	EAPC (%, 95 CI)
Mali	1210 (766 to 1667)	2205 (1382 to 3068)	82.24% (51.73 to 114.39)	826.4 (523.1 to 1138.3)	682.4 (427.7 to 949.5)	-0.54% (-0.63 to -0.45)
Marshall Islands	0 (0 to 1)	1 (0 to 1)	12.24% (-6.11 to 35.86)	85.9 (52.9 to 129.1)	35.3 (22.2 to 54.4)	-2.95% (-3.05 to -2.86)
Mauritania	304 (202 to 403)	289 (190 to 381)	-4.86% (-17.67 to 14.11)	928.6 (616.7 to 1233.1)	370.3 (243.6 to 487.8)	-2.82% (-3.18 to -2.45)
Mexico	2359 (1659 to 3071)	807 (569 to 1055)	-65.79% (-68.35 to -63.14)	150.8 (106 to 196.3)	19.9 (14 to 26)	-7.47% (-7.79 to -7.16)
Micronesia (Federated States of)	7 (4 to 11)	3 (2 to 4)	-63.9% (-70.22 to -56.79)	479.4 (292.3 to 724.4)	94 (58.3 to 144)	-5.89% (-6.03 to -5.76)
Mongolia	66 (38 to 96)	28 (17 to 40)	-57.62% (-63.93 to -49.87)	191.2 (111.8 to 280.1)	25.8 (15.7 to 37.5)	-6.35% (-7.04 to -5.66)
Montenegro	1 (1 to 1)	1 (1 to 2)	18.98% (-2.11 to 46.96)	5.4 (3.3 to 8.7)	4.9 (2.9 to 7.6)	0.7% (0.16 to 1.24)
Morocco	1426 (847 to 2219)	526 (309 to 805)	-63.14% (-70.05 to -55.19)	294.7 (174.9 to 458.5)	48.9 (28.7 to 74.8)	-6.01% (-6.23 to -5.78)

Location	Incident cases in 1990 (95% UI)	Incident cases in 2019 (95% UI)	Relative change (%, 95 CI)	ASIR in 1990 (95% UI)	ASIR in 2019 (95% UI)	EAPC (%, 95 CI)
Mozambique	1846 (1162 to 2738)	2649 (1658 to 3909)	43.5% (18.54 to 68.3)	755.6 (475.5 to 1120.8)	529.6 (331.5 to 781.7)	-1.05% (-1.35 to -0.76)
Myanmar	1455 (868 to 2271)	804 (487 to 1208)	-44.74% (-51.93 to -35.39)	191.2 (114.1 to 298.3)	46.5 (28.2 to 69.9)	-4.76% (-5.01 to -4.51)
Namibia	151 (98 to 218)	76 (48 to 108)	-49.44% (-57.36 to -39.43)	655.5 (424 to 946.6)	139.4 (87.4 to 197.1)	-5.06% (-5.32 to -4.8)
Nepal	878 (561 to 1336)	260 (161 to 389)	-70.43% (-75.42 to -64.84)	245.1 (156.5 to 372.9)	33.6 (20.9 to 50.3)	-7.23% (-7.44 to -7.02)
Nicaragua	105 (72 to 132)	41 (29 to 51)	-60.95% (-66.53 to -53.53)	186.4 (127 to 234.2)	24.6 (17.4 to 30.4)	-6.96% (-7.14 to -6.78)
Niger	1115 (718 to 1528)	3242 (2065 to 4404)	190.66% (148.93 to 243.25)	921.2 (592.7 to 1262.2)	1025 (653 to 1392.5)	0.52% (0.44 to 0.61)
Nigeria	13778 (9074 to 18815)	29250 (19774 to 39460)	112.3% (104.2 to 122.76)	1028.2 (677.2 to 1404.1)	720.5 (487.1 to 972)	-1.02% (-1.2 to -0.83)
North Macedonia	3 (2 to 4)	3 (2 to 4)	3.75% (-14.34 to 26.46)	5.1 (3 to 7.7)	3.8 (2.3 to 5.9)	-0.13% (-1.18 to 0.94)

Location	Incident cases in 1990 (95% UI)	Incident cases in 2019 (95% UI)	Relative change (%, 95 CI)	ASIR in 1990 (95% UI)	ASIR in 2019 (95% UI)	EAPC (%, 95 CI)
Pakistan	9661 (6337 to 14318)	9088 (5988 to 13299)	-5.94% (-13.98 to 3.19)	482.1 (316.2 to 714.4)	196.5 (129.5 to 287.6)	-3.16% (-3.3 to -3.03)
Papua New Guinea	199 (123 to 303)	483 (301 to 753)	143.25% (104.37 to 187.53)	282.7 (174.4 to 431.1)	222.4 (138.6 to 346.5)	-1.07% (-1.21 to -0.94)
Paraguay	161 (110 to 204)	30 (21 to 39)	-81.05% (-84.19 to -77.75)	217 (148.9 to 275.5)	18 (12.5 to 23)	-8.73% (-8.92 to -8.54)
Peru	1096 (699 to 1423)	535 (344 to 726)	-51.13% (-59.34 to -43.18)	268.5 (171.2 to 348.8)	53 (34.1 to 71.9)	-5.83% (-6.1 to -5.55)
Philippines	1947 (1199 to 2967)	1754 (1092 to 2648)	-9.92% (-14.12 to -6.22)	169 (104.1 to 257.5)	62.1 (38.7 to 93.7)	-3.27% (-3.41 to -3.13)
Republic of Moldova	9 (7 to 12)	7 (5 to 9)	-25.68% (-36.09 to -13.83)	8 (5.7 to 10)	5.5 (4 to 6.9)	-1.13% (-2.17 to -0.08)
Russian Federation	154 (102 to 196)	701 (466 to 894)	355.94% (341.41 to 371.54)	4.4 (2.9 to 5.7)	13.7 (9.1 to 17.5)	5.16% (4.26 to 6.07)
Rwanda	918 (575 to 1329)	382 (243 to 548)	-58.36% (-64.87 to -52.27)	904.4 (566.6 to 1309.7)	155.7 (99 to 223.3)	-6.68% (-7.11 to -6.24)

Location	Incident cases in 1990 (95% UI)	Incident cases in 2019 (95% UI)	Relative change (%, 95 CI)	ASIR in 1990 (95% UI)	ASIR in 2019 (95% UI)	EAPC (%, 95 CI)
Saint Lucia	2 (1 to 2)	1 (0 to 1)	-55.72% (-62.77 to -48.79)	65.7 (43.2 to 87.4)	10.3 (6.8 to 13.9)	-6.62% (-7.18 to -6.05)
Saint Vincent and the Grenadines	1 (1 to 1)	1 (0 to 1)	-40.43% (-48.29 to -31.2)	57.3 (38.5 to 75.5)	15.9 (10.7 to 20.8)	-4.35% (-4.48 to -4.21)
Samoa	1 (1 to 2)	1 (0 to 1)	-32.71% (-43.22 to -18.5)	43.9 (27.3 to 66.4)	16.3 (10.3 to 24.5)	-3.74% (-4.28 to -3.19)
Senegal	875 (546 to 1226)	781 (488 to 1078)	-10.69% (-25.16 to 9.38)	761.8 (475.2 to 1067.4)	275 (171.6 to 379.6)	-3.83% (-4.06 to -3.61)
Serbia	17 (10 to 26)	11 (7 to 17)	-33.13% (-43.39 to -19.4)	5.8 (3.3 to 8.9)	4 (2.4 to 6.1)	1.56% (0.13 to 3.01)
Sierra Leone	467 (298 to 634)	587 (384 to 788)	25.77% (8.96 to 47.22)	768.3 (490.1 to 1042.8)	424.1 (277.7 to 569)	-1.98% (-2.25 to -1.7)
Solomon Islands	17 (11 to 26)	24 (15 to 37)	40.93% (16.66 to 65.95)	337 (215.9 to 518.2)	162.4 (100.4 to 248.9)	-2.57% (-2.6 to -2.55)
Somalia	1342 (809 to 1913)	3156 (1886 to 4536)	135.07% (99.21 to 176.19)	1059.9 (638.4 to 1510.4)	959.3 (573.4 to 1379)	-0.23% (-0.27 to -0.19)

Location	Incident cases in 1990 (95% UI)	Incident cases in 2019 (95% UI)	Relative change (%, 95 CI)	ASIR in 1990 (95% UI)	ASIR in 2019 (95% UI)	EAPC (%, 95 CI)
South Africa	2315 (1472 to 3295)	696 (452 to 955)	-69.94% (-72.4 to -67.56)	318.7 (202.6 to 453.7)	44.6 (28.9 to 61.2)	-6.91% (-7.16 to -6.65)
South Sudan	579 (362 to 817)	1112 (717 to 1545)	92.1% (60.26 to 127.97)	753.9 (471.8 to 1063.9)	605.7 (390.5 to 841.1)	-0.71% (-0.76 to -0.67)
Sri Lanka	170 (102 to 266)	72 (45 to 110)	-57.87% (-64.18 to -50.01)	43.3 (26 to 67.8)	10.1 (6.3 to 15.5)	-4.94% (-5.32 to -4.55)
Sudan	1179 (733 to 1812)	997 (609 to 1514)	-15.45% (-32.2 to 5.11)	347.9 (216.3 to 534.7)	125.7 (76.7 to 190.9)	-3.45% (-3.92 to -2.98)
Suriname	4 (3 to 6)	3 (2 to 4)	-31.22% (-41.49 to -18.09)	51 (33.5 to 67.2)	16.4 (10.7 to 21.6)	-3.81% (-3.89 to -3.74)
Syrian Arab Republic	438 (263 to 661)	149 (88 to 233)	-66.1% (-72.07 to -58.65)	241 (144.9 to 363.8)	31.3 (18.6 to 49.2)	-5.64% (-7.09 to -4.18)
Tajikistan	123 (74 to 180)	104 (62 to 156)	-15.34% (-30.59 to 2.9)	192.8 (116.7 to 283.9)	47.5 (28.3 to 71.1)	-4.28% (-4.7 to -3.85)
Thailand	718 (440 to 1076)	228 (141 to 355)	-68.19% (-73.35 to -62.14)	54 (33.1 to 80.9)	7.7 (4.7 to 12)	-7.08% (-8.08 to -6.06)
Timor-Leste	52 (32 to 82)	56 (35 to 87)	8.75% (-5.88 to 29.86)	372.1 (226.5 to 586.7)	213 (130.6 to 326.7)	-1.83% (-2.32 to -1.35)

Location	Incident cases in 1990 (95% UI)	Incident cases in 2019 (95% UI)	Relative change (%, 95 CI)	ASIR in 1990 (95% UI)	ASIR in 2019 (95% UI)	EAPC (%, 95 CI)
Togo	532 (329 to 753)	754 (486 to 1026)	41.86% (23.57 to 69.93)	936.7 (578.8 to 1325.6)	439.1 (282.9 to 597.4)	-2.33% (-2.72 to -1.95)
Tonga	2 (1 to 4)	1 (1 to 1)	-62.35% (-68.85 to -55.75)	122.8 (76.1 to 188.1)	34.5 (21.3 to 52)	-4.59% (-4.81 to -4.38)
Tunisia	138 (84 to 209)	72 (41 to 112)	-48% (-57.66 to -36.62)	91 (55.4 to 137.6)	19.2 (11 to 29.9)	-4.52% (-5.39 to -3.64)
Turkey	533 (329 to 808)	396 (244 to 607)	-25.66% (-36.49 to -13.13)	45.9 (28.3 to 69.5)	15.3 (9.4 to 23.4)	-2.21% (-3.23 to -1.18)
Turkmenistan	40 (24 to 60)	34 (21 to 50)	-14.35% (-29.46 to 2.61)	73.4 (44.7 to 109.6)	24.5 (14.6 to 35.9)	-3.72% (-3.97 to -3.47)
Tuvalu	0 (0 to 0)	0 (0 to 0)	-72.19% (-76.41 to -67.14)	99.9 (61.5 to 151.3)	23.7 (14.6 to 36.4)	-4.99% (-5.12 to -4.86)
Uganda	1777 (1131 to 2521)	1469 (929 to 2089)	-17.34% (-30.14 to -3.67)	742.4 (472.3 to 1053)	233.7 (147.8 to 332.4)	-4.4% (-4.85 to -3.95)
Ukraine	67 (46 to 87)	217 (147 to 278)	222.42% (182.42 to 268.21)	4.4 (3 to 5.7)	13.4 (9 to 17.1)	5.73% (4.14 to 7.34)

Location	Incident cases in 1990 (95% UI)	Incident cases in 2019 (95% UI)	Relative change (%, 95 CI)	ASIR in 1990 (95% UI)	ASIR in 2019 (95% UI)	EAPC (%, 95 CI)
United Republic of Tanzania	3116 (1917 to 4461)	2946 (1798 to 4285)	-5.46% (-21.27 to 12.26)	793.5 (488.2 to 1135.9)	277 (169.1 to 402.8)	-3.7% (-3.93 to -3.47)
Uzbekistan	224 (133 to 333)	80 (48 to 118)	-64.49% (-70.35 to -57.22)	71.6 (42.6 to 106.6)	8.7 (5.3 to 13)	-7.37% (-7.81 to -6.93)
Vanuatu	6 (3 to 9)	6 (4 to 10)	6.65% (-9.41 to 25.38)	235.6 (140.8 to 360.1)	101.8 (62.3 to 159.1)	-3.04% (-3.17 to -2.91)
Venezuela (Bolivarian Republic of)	451 (310 to 571)	279 (199 to 336)	-38.23% (-47.41 to -23.79)	134.9 (92.7 to 170.6)	31.7 (22.6 to 38.2)	-4.65% (-4.89 to -4.4)
Viet Nam	422 (262 to 662)	122 (76 to 184)	-71.16% (-76.06 to -65.25)	38.3 (23.8 to 60.2)	3.7 (2.3 to 5.6)	-8.2% (-8.56 to -7.83)
Yemen	861 (496 to 1342)	1315 (782 to 2020)	52.65% (25.82 to 89.94)	470.8 (271.2 to 733.6)	246.4 (146.6 to 378.7)	-2.2% (-2.3 to -2.09)
Zambia	827 (509 to 1192)	529 (325 to 784)	-35.97% (-45.04 to -25.1)	697.7 (429.3 to 1005.9)	181.7 (111.5 to 269)	-4.58% (-4.99 to -4.15)
Zimbabwe	666 (416 to 935)	337 (208 to 478)	-49.41% (-57.2 to -41.78)	452.5 (282.6 to 635.4)	117.7 (72.6 to 166.9)	-4.03% (-4.27 to -3.79)

ASIR – age-standardized incidence rate (per 100,000 population), CI – confidence interval, EAPC – estimated annual percentage change, MSMI – maternal sepsis and other maternal infections, UI – uncertain interval.

Table S15. Incidence of MSMI in 131 low- and middle-income countries and territories with EAPC in age group of 50-54 years from 1990 and 2019.

Location	Incident cases in 1990 (95% UI)	Incident cases in 2019 (95% UI)	Relative change (%, 95 CI)	ASIR in 1990 (95% UI)	ASIR in 2019 (95% UI)	EAPC (%, 95 CI)
Afghanistan	25 (18 to 33)	47 (34 to 63)	87.72% (64.19 to 117.04)	11.5 (8.5 to 15.4)	8.2 (6 to 11.1)	-1.23% (-1.37 to -1.08)
Albania	0 (0 to 0)	0 (0 to 0)	-70.08% (-74.27 to -65.63)	0.6 (0.4 to 0.8)	0.1 (0.1 to 0.2)	-5.79% (-6.62 to -4.95)
Algeria	33 (24 to 44)	31 (22 to 41)	-7.75% (-19.07 to 6.04)	9.1 (6.7 to 12)	3 (2.2 to 4)	-3.78% (-5.02 to -2.53)
American Samoa	0 (0 to 0)	0 (0 to 0)	-40.93% (-48.85 to -32.4)	2 (1.4 to 2.7)	0.5 (0.3 to 0.7)	-4.9% (-5.01 to -4.8)
Angola	22 (16 to 29)	28 (20 to 37)	24.79% (8.75 to 40.42)	16.7 (11.8 to 22)	6.5 (4.6 to 8.6)	-3.03% (-3.26 to -2.79)
Armenia	0 (0 to 1)	0 (0 to 0)	-35.83% (-44.06 to -24.91)	0.5 (0.3 to 0.7)	0.3 (0.2 to 0.4)	-0.94% (-1.76 to -0.11)
Azerbaijan	1 (0 to 1)	0 (0 to 1)	-46.04% (-52.79 to -37.78)	0.4 (0.3 to 0.5)	0.1 (0.1 to 0.1)	-3.2% (-4.03 to -2.36)
Bangladesh	54 (40 to 73)	46 (34 to 62)	-13.85% (-25.13 to -0.87)	4.4 (3.3 to 6)	1.3 (0.9 to 1.7)	-3.01% (-3.43 to -2.58)

Location	Incident cases in 1990 (95% UI)	Incident cases in 2019 (95% UI)	Relative change (%, 95 CI)	ASIR in 1990 (95% UI)	ASIR in 2019 (95% UI)	EAPC (%, 95 CI)
Belarus	0 (0 to 0)	1 (0 to 1)	68.59% (49.88 to 90.4)	0.1 (0.1 to 0.1)	0.2 (0.1 to 0.2)	2.43% (1.67 to 3.2)
Belize	0 (0 to 0)	0 (0 to 0)	9.37% (-2.75 to 23.94)	3 (2.2 to 3.7)	0.8 (0.6 to 1)	-4.38% (-4.53 to -4.23)
Benin	10 (7 to 13)	19 (13 to 25)	87.4% (64.91 to 114.29)	17.8 (12.8 to 23.5)	11.7 (8.3 to 15.5)	-1.18% (-1.29 to -1.07)
Bhutan	1 (0 to 1)	0 (0 to 0)	-41.7% (-49.05 to -33.97)	8.2 (6 to 11)	2.5 (1.8 to 3.3)	-4.17% (-4.43 to -3.91)
Bolivia (Plurinational State of)	10 (7 to 12)	10 (8 to 13)	5.69% (-7 to 19.92)	10.4 (7.7 to 13)	4.2 (3.1 to 5.2)	-2.9% (-3.18 to -2.63)
Bosnia and Herzegovina	0 (0 to 0)	0 (0 to 0)	-56.73% (-62.13 to -50.77)	0.2 (0.1 to 0.3)	0.1 (0.1 to 0.1)	-1.85% (-2.21 to -1.49)
Botswana	2 (2 to 3)	1 (1 to 2)	-48.27% (-54.01 to -40.57)	13.7 (10 to 18.2)	2.7 (2 to 3.5)	-5.17% (-5.41 to -4.93)
Brazil	96 (79 to 110)	48 (36 to 58)	-50.54% (-55.94 to -45.35)	3.6 (3 to 4.2)	0.7 (0.5 to 0.9)	-4.98% (-5.21 to -4.75)
Bulgaria	0 (0 to 0)	1 (0 to 1)	634.04% (540.52 to 745.64)	0 (0 to 0)	0.3 (0.2 to 0.4)	8.69% (7.06 to 10.35)

Location	Incident cases in 1990 (95% UI)	Incident cases in 2019 (95% UI)	Relative change (%, 95 CI)	ASIR in 1990 (95% UI)	ASIR in 2019 (95% UI)	EAPC (%, 95 CI)
Burkina Faso	31 (22 to 41)	44 (31 to 57)	40.17% (20.98 to 61.73)	23 (16.1 to 30.1)	13.7 (9.8 to 18)	-2.06% (-2.15 to -1.97)
Burundi	14 (10 to 18)	19 (13 to 25)	33.85% (15.86 to 52.01)	19.4 (13.5 to 25.7)	13 (9.2 to 17.5)	-1.16% (-1.31 to -1.02)
Cabo Verde	1 (0 to 1)	0 (0 to 0)	-75.48% (-78.75 to -71.5)	10.1 (7.1 to 13.3)	1.2 (0.8 to 1.5)	-7.28% (-7.79 to -6.75)
Cambodia	10 (7 to 15)	5 (4 to 8)	-47.75% (-54.99 to -39.89)	6.6 (4.6 to 9.3)	1.3 (0.9 to 1.9)	-6.12% (-6.39 to -5.85)
Cameroon	19 (14 to 26)	21 (15 to 27)	7.34% (-5.32 to 22.17)	13.3 (9.5 to 17.4)	5.3 (3.8 to 6.8)	-2.72% (-3.14 to -2.3)
Central African Republic	6 (4 to 8)	6 (5 to 8)	11.04% (-1.76 to 26.61)	13.9 (10 to 18.5)	7.9 (5.8 to 10.4)	-1.84% (-1.98 to -1.7)
Chad	11 (8 to 14)	24 (17 to 32)	118.32% (92.96 to 148.43)	14.2 (9.9 to 18.5)	14.2 (10.2 to 18.6)	0.35% (0.2 to 0.5)
China	184 (130 to 255)	725 (506 to 1012)	294.44% (276.96 to 312.2)	0.8 (0.6 to 1.1)	1.2 (0.8 to 1.6)	4.66% (2.99 to 6.35)
Colombia	23 (17 to 27)	13 (10 to 15)	-43.94% (-49.75 to -35.77)	4.3 (3.3 to 5)	0.9 (0.7 to 1)	-5.37% (-5.63 to -5.11)
Comoros	2 (1 to 2)	1 (0 to 1)	-63.57% (-68.36 to -57.91)	24.3 (17.6 to 30.9)	3.8 (2.7 to 5)	-6.01% (-6.6 to -5.42)

Location	Incident cases in 1990 (95% UI)	Incident cases in 2019 (95% UI)	Relative change (%, 95 CI)	ASIR in 1990 (95% UI)	ASIR in 2019 (95% UI)	EAPC (%, 95 CI)
Congo	5<br>(4 to 7)	3<br>(2 to 4)	-45.07%<br>(-51.79 to -38.44)	14.9<br>(10.6 to 20)	3.1<br>(2.2 to 4.2)	-4.93%<br>(-5.37 to -4.48)
Costa Rica	1<br>(1 to 1)	1<br>(0 to 1)	-28.9%<br>(-37.27 to -18.87)	1.6<br>(1.2 to 1.9)	0.4<br>(0.3 to 0.4)	-5.07%<br>(-5.6 to -4.53)
Côte d'Ivoire	27<br>(19 to 35)	36<br>(26 to 47)	34.85%<br>(19.01 to 54.41)	20<br>(14.2 to 26.5)	10.6<br>(7.6 to 13.7)	-2.21%<br>(-2.3 to -2.12)
Cuba	1<br>(0 to 1)	0<br>(0 to 1)	-27.8%<br>(-36.67 to -18.35)	0.3<br>(0.2 to 0.3)	0.1<br>(0.1 to 0.1)	-2.65%<br>(-3.13 to -2.16)
Democratic People's Republic of Korea	9<br>(6 to 12)	1<br>(1 to 2)	-86.04%<br>(-87.74 to -83.97)	1.3<br>(0.9 to 1.8)	0.1<br>(0.1 to 0.2)	-8.38%<br>(-8.63 to -8.13)
Democratic Republic of the Congo	113<br>(80 to 150)	86<br>(60 to 116)	-23.39%<br>(-32.32 to -12.47)	20.3<br>(14.4 to 27)	6.9<br>(4.8 to 9.3)	-3.33%<br>(-3.84 to -2.82)
Djibouti	1<br>(1 to 2)	2<br>(1 to 3)	80.4%<br>(57.56 to 109.5)	22.4<br>(15.7 to 30.2)	10.4<br>(7.4 to 13.7)	-2.56%<br>(-2.91 to -2.21)
Dominica	0<br>(0 to 0)	0<br>(0 to 0)	-28.96%<br>(-37.5 to -18.66)	0.8<br>(0.6 to 1)	0.3<br>(0.3 to 0.4)	-2.41%<br>(-2.56 to -2.26)
Dominican Republic	3<br>(2 to 3)	1<br>(1 to 1)	-55.03%<br>(-59.89 to -48.14)	2.4<br>(1.8 to 3.1)	0.4<br>(0.3 to 0.6)	-5.57%<br>(-6.14 to -4.99)

Location	Incident cases in 1990 (95% UI)	Incident cases in 2019 (95% UI)	Relative change (%, 95 CI)	ASIR in 1990 (95% UI)	ASIR in 2019 (95% UI)	EAPC (%, 95 CI)
Ecuador	8 (6 to 10)	4 (3 to 5)	-50.18% (-56.06 to -42.76)	5.2 (3.9 to 6.5)	1 (0.7 to 1.2)	-6.27% (-6.44 to -6.1)
Egypt	20 (15 to 27)	11 (8 to 15)	-43.54% (-50.15 to -35.12)	2.2 (1.7 to 3)	0.6 (0.4 to 0.8)	-3.94% (-4.42 to -3.46)
El Salvador	4 (3 to 5)	1 (1 to 1)	-75.33% (-78.11 to -72.09)	4.7 (3.6 to 5.6)	0.6 (0.4 to 0.7)	-7.4% (-8.07 to -6.72)
Equatorial Guinea	1 (1 to 2)	0 (0 to 0)	-73.02% (-76.38 to -69.11)	18.5 (13.1 to 24.6)	1.9 (1.4 to 2.6)	-7.77% (-8.39 to -7.14)
Eritrea	9 (6 to 12)	10 (7 to 14)	18.51% (2 to 36.36)	21.2 (15 to 28.5)	10.2 (7.2 to 13.8)	-2.35% (-2.63 to -2.08)
Eswatini	1 (1 to 1)	0 (0 to 1)	-59.08% (-63.84 to -52.48)	9.6 (6.8 to 12.8)	2.1 (1.5 to 2.8)	-4.82% (-4.96 to -4.67)
Ethiopia	117 (85 to 155)	108 (77 to 145)	-7.14% (-14.69 to -1.23)	18.5 (13.4 to 24.5)	8.8 (6.3 to 11.8)	-2.42% (-2.66 to -2.17)
Fiji	0 (0 to 0)	0 (0 to 0)	-13.27% (-24.31 to -1.45)	1 (0.7 to 1.4)	0.5 (0.3 to 0.7)	-2.86% (-3 to -2.72)
Gabon	2 (1 to 2)	0 (0 to 0)	-79.74% (-82.29 to -76.85)	10.9 (7.7 to 14.5)	1 (0.7 to 1.4)	-7.55% (-7.87 to -7.22)
Gambia	2 (1 to 3)	2 (2 to 3)	17.1% (3.29 to 36.39)	20.9 (14.7 to 27.7)	8.4 (6.1 to 10.9)	-3% (-3.32 to -2.68)

Location	Incident cases in 1990 (95% UI)	Incident cases in 2019 (95% UI)	Relative change (%, 95 CI)	ASIR in 1990 (95% UI)	ASIR in 2019 (95% UI)	EAPC (%, 95 CI)
Georgia	0<br>(0 to 0)	1<br>(0 to 1)	77.84%<br>(51.81 to 103.29)	0.2<br>(0.1 to 0.3)	0.5<br>(0.4 to 0.7)	3.79%<br>(3.14 to 4.44)
Ghana	41<br>(29 to 54)	35<br>(25 to 45)	-13.74%<br>(-24.53 to -0.98)	20.6<br>(14.4 to 26.9)	6.3<br>(4.5 to 8.2)	-3.58%<br>(-3.92 to -3.23)
Global	3326<br>(2479 to 4365)	3566<br>(2619 to 4661)	7.19%<br>(5.04 to 9.29)	3.2<br>(2.4 to 4.2)	1.6<br>(1.2 to 2.1)	-2.07%<br>(-2.27 to -1.86)
Grenada	0<br>(0 to 0)	0<br>(0 to 0)	-56.27%<br>(-61.33 to -50.69)	1.5<br>(1.1 to 1.9)	0.2<br>(0.2 to 0.3)	-5.96%<br>(-6.56 to -5.35)
Guatemala	13<br>(10 to 16)	7<br>(5 to 8)	-48.74%<br>(-55.35 to -41.51)	11.2<br>(8.8 to 13.1)	2.1<br>(1.6 to 2.6)	-6.35%<br>(-6.83 to -5.87)
Guinea	16<br>(12 to 21)	20<br>(15 to 26)	22.97%<br>(8.87 to 38.41)	19.2<br>(13.8 to 25.2)	12.5<br>(9.1 to 16.3)	-1.54%<br>(-1.6 to -1.47)
Guinea-Bissau	3<br>(2 to 4)	4<br>(3 to 6)	37.79%<br>(21.84 to 58.02)	24.9<br>(17.7 to 32.6)	17.6<br>(12.5 to 22.8)	-1.19%<br>(-1.32 to -1.06)
Guyana	0<br>(0 to 0)	0<br>(0 to 0)	-47.63%<br>(-54.68 to -40.78)	2.2<br>(1.6 to 2.8)	0.6<br>(0.5 to 0.8)	-4.79%<br>(-4.99 to -4.59)
Haiti	12<br>(9 to 15)	10<br>(8 to 13)	-11.87%<br>(-22.4 to -0.82)	12.3<br>(8.9 to 15.4)	4.6<br>(3.4 to 5.8)	-3.06%<br>(-3.25 to -2.87)
Honduras	4<br>(3 to 5)	3<br>(2 to 3)	-38.75%<br>(-46.38 to -29.49)	7.3<br>(5.5 to 8.7)	1.5<br>(1.2 to 1.8)	-5.95%<br>(-6.2 to -5.7)

Location	Incident cases in 1990 (95% UI)	Incident cases in 2019 (95% UI)	Relative change (%, 95 CI)	ASIR in 1990 (95% UI)	ASIR in 2019 (95% UI)	EAPC (%, 95 CI)
India	814 (610 to 1097)	406 (305 to 547)	-50.1% (-52.4 to -47.58)	5.9 (4.4 to 8)	1.3 (1 to 1.7)	-5.23% (-5.5 to -4.96)
Indonesia	96 (67 to 135)	54 (38 to 76)	-43.32% (-46.13 to -40.46)	2.9 (2 to 4)	0.7 (0.5 to 1)	-3.79% (-4.37 to -3.21)
Iran (Islamic Republic of)	73 (54 to 98)	9 (7 to 12)	-87.3% (-87.91 to -86.58)	9.7 (7.1 to 12.9)	0.4 (0.3 to 0.6)	-9.12% (-10.21 to -8.02)
Iraq	16 (12 to 22)	7 (5 to 9)	-55.55% (-61.38 to -49.11)	7.2 (5.3 to 9.9)	0.9 (0.7 to 1.3)	-7.28% (-7.64 to -6.93)
Jamaica	0 (0 to 1)	1 (1 to 1)	69.69% (50.28 to 91.59)	1.2 (0.9 to 1.5)	1 (0.7 to 1.2)	0.7% (-0.1 to 1.5)
Jordan	2 (2 to 3)	1 (1 to 1)	-57.55% (-63.26 to -50.91)	4.8 (3.6 to 6.4)	0.4 (0.3 to 0.6)	-8.07% (-8.24 to -7.89)
Kazakhstan	2 (1 to 3)	2 (1 to 3)	-4.47% (-17.6 to 10.18)	0.5 (0.3 to 0.6)	0.4 (0.3 to 0.5)	1.14% (0.13 to 2.17)
Kenya	38 (27 to 50)	15 (11 to 20)	-60.23% (-61.55 to -58.8)	15.2 (11.1 to 20)	2 (1.5 to 2.7)	-6.81% (-6.91 to -6.71)
Kiribati	0 (0 to 0)	0 (0 to 0)	-41.7% (-48.9 to -33.75)	3.6 (2.6 to 4.9)	1 (0.7 to 1.3)	-4.61% (-4.77 to -4.44)
Kyrgyzstan	2 (1 to 2)	2 (1 to 2)	-10.41% (-21.17 to 3)	2 (1.4 to 2.7)	1 (0.7 to 1.4)	-1.14% (-1.7 to -0.58)

Location	Incident cases in 1990 (95% UI)	Incident cases in 2019 (95% UI)	Relative change (%, 95 CI)	ASIR in 1990 (95% UI)	ASIR in 2019 (95% UI)	EAPC (%, 95 CI)
Lao People's Democratic Republic	5<br>(4 to 8)	4<br>(3 to 5)	-29.47%<br>(-39.41 to -18.97)	8.2<br>(5.6 to 11.9)	2.6<br>(1.8 to 3.6)	-4.7%<br>(-5.28 to -4.13)
Lebanon	3<br>(2 to 4)	1<br>(1 to 1)	-75.96%<br>(-79.12 to -72.88)	4.3<br>(3.2 to 5.8)	0.5<br>(0.4 to 0.7)	-7.46%<br>(-7.71 to -7.21)
Lesotho	3<br>(2 to 4)	1<br>(1 to 1)	-71.3%<br>(-74.72 to -67.56)	10.1<br>(7.2 to 13.5)	2<br>(1.4 to 2.6)	-5.28%<br>(-5.51 to -5.05)
Liberia	5<br>(4 to 7)	3<br>(3 to 4)	-33.57%<br>(-41.4 to -24.06)	19.6<br>(14 to 25.7)	5.5<br>(4 to 7.2)	-4.27%<br>(-4.69 to -3.84)
Libya	6<br>(4 to 7)	2<br>(1 to 3)	-65.68%<br>(-69.7 to -60.95)	11.4<br>(8.4 to 15.3)	1.1<br>(0.8 to 1.4)	-6.94%<br>(-7.65 to -6.23)
Madagascar	18<br>(13 to 25)	13<br>(10 to 18)	-27.16%<br>(-36.14 to -16.1)	12.1<br>(8.6 to 16.5)	3.3<br>(2.3 to 4.4)	-4.75%<br>(-5 to -4.5)
Malawi	29<br>(20 to 39)	27<br>(19 to 37)	-4.51%<br>(-15.98 to 8.84)	24.7<br>(17.4 to 33.9)	12.6<br>(8.8 to 17.2)	-2.21%<br>(-2.5 to -1.92)
Malaysia	6<br>(4 to 8)	5<br>(4 to 7)	-5.02%<br>(-16.08 to 9.72)	1.8<br>(1.3 to 2.6)	0.7<br>(0.5 to 1)	-3.72%<br>(-4.07 to -3.37)
Maldives	0<br>(0 to 0)	0<br>(0 to 0)	-75.9%<br>(-79.15 to -72.38)	8.3<br>(5.6 to 11.4)	0.7<br>(0.5 to 0.9)	-7.91%<br>(-8.37 to -7.45)

Location	Incident cases in 1990 (95% UI)	Incident cases in 2019 (95% UI)	Relative change (%, 95 CI)	ASIR in 1990 (95% UI)	ASIR in 2019 (95% UI)	EAPC (%, 95 CI)
Mali	24 (17 to 32)	43 (30 to 56)	75.01% (51.16 to 97.49)	20.1 (14.2 to 26.3)	16.5 (11.7 to 21.6)	-0.56% (-0.65 to -0.47)
Marshall Islands	0 (0 to 0)	0 (0 to 0)	21.62% (6.12 to 40.66)	2.2 (1.6 to 3.1)	0.9 (0.7 to 1.3)	-2.94% (-3.03 to -2.85)
Mauritania	6 (4 to 7)	6 (4 to 7)	-7.13% (-17.59 to 7.02)	22.3 (16.3 to 28.1)	8.8 (6.4 to 11.1)	-2.82% (-3.18 to -2.45)
Mexico	44 (34 to 55)	15 (11 to 19)	-66.31% (-68.61 to -64.17)	3.5 (2.6 to 4.3)	0.4 (0.3 to 0.5)	-7.81% (-8.14 to -7.47)
Micronesia (Federated States of)	0 (0 to 0)	0 (0 to 0)	-58.65% (-64.35 to -52.51)	12.5 (9 to 17.1)	2.4 (1.7 to 3.4)	-5.89% (-6.03 to -5.76)
Mongolia	1 (1 to 2)	1 (0 to 1)	-53.49% (-58.83 to -46.4)	4.9 (3.5 to 6.6)	0.7 (0.5 to 0.9)	-6.37% (-7.05 to -5.68)
Montenegro	0 (0 to 0)	0 (0 to 0)	-1.95% (-14.47 to 15.28)	0.2 (0.1 to 0.2)	0.1 (0.1 to 0.2)	0.7% (0.16 to 1.23)
Morocco	36 (27 to 49)	14 (11 to 19)	-60.72% (-65.67 to -55.25)	9 (6.7 to 12.2)	1.5 (1.1 to 2)	-6.02% (-6.25 to -5.78)
Mozambique	38 (27 to 52)	49 (34 to 67)	28.35% (11.24 to 46.01)	18.7 (13.4 to 25.4)	13.1 (9.1 to 17.7)	-1.06% (-1.35 to -0.77)

Location	Incident cases in 1990 (95% UI)	Incident cases in 2019 (95% UI)	Relative change (%, 95 CI)	ASIR in 1990 (95% UI)	ASIR in 2019 (95% UI)	EAPC (%, 95 CI)
Myanmar	32 (22 to 45)	18 (12 to 24)	-44.7% (-51.75 to -37.2)	4.6 (3.1 to 6.6)	1.1 (0.8 to 1.6)	-4.73% (-4.99 to -4.47)
Namibia	3 (2 to 4)	2 (1 to 2)	-49.02% (-55.38 to -41.2)	16.3 (11.9 to 21.9)	3.4 (2.5 to 4.5)	-5.09% (-5.35 to -4.84)
Nepal	19 (14 to 26)	6 (4 to 8)	-69.66% (-73.55 to -65.25)	6.6 (4.9 to 8.9)	0.9 (0.7 to 1.2)	-7.25% (-7.46 to -7.05)
Nicaragua	2 (2 to 2)	1 (1 to 1)	-58.69% (-63.31 to -52.3)	4.5 (3.3 to 5.4)	0.6 (0.4 to 0.7)	-6.95% (-7.12 to -6.77)
Niger	19 (14 to 25)	65 (47 to 85)	240.08% (201.95 to 289.14)	22.4 (16.2 to 29.4)	24.6 (17.5 to 31.9)	0.48% (0.39 to 0.56)
Nigeria	259 (189 to 334)	577 (426 to 733)	122.49% (115.65 to 131.04)	24.6 (17.9 to 31.6)	17.1 (12.6 to 21.7)	-1.05% (-1.25 to -0.85)
North Macedonia	0 (0 to 0)	0 (0 to 0)	3.88% (-9.69 to 20.6)	0.2 (0.1 to 0.2)	0.1 (0.1 to 0.2)	-0.1% (-1.15 to 0.95)
Pakistan	196 (147 to 268)	176 (132 to 235)	-10.07% (-15.82 to -4.1)	12.4 (9.3 to 16.9)	5.1 (3.8 to 6.7)	-3.15% (-3.28 to -3.01)
Papua New Guinea	4 (3 to 6)	10 (7 to 14)	135.42% (105.09 to 169)	7.3 (5.3 to 10)	5.8 (4.1 to 8.1)	-1.07% (-1.2 to -0.93)

Location	Incident cases in 1990 (95% UI)	Incident cases in 2019 (95% UI)	Relative change (%, 95 CI)	ASIR in 1990 (95% UI)	ASIR in 2019 (95% UI)	EAPC (%, 95 CI)
Paraguay	3<br>(2 to 4)	1<br>(1 to 1)	-78.96%<br>(-81.44 to -76.06)	5.4<br>(4.1 to 6.6)	0.4<br>(0.3 to 0.5)	-8.72%<br>(-8.9 to -8.54)
Peru	21<br>(15 to 27)	11<br>(8 to 14)	-47.5%<br>(-54.99 to -40.04)	6.4<br>(4.5 to 8)	1.3<br>(0.9 to 1.6)	-5.78%<br>(-6.06 to -5.51)
Philippines	39<br>(27 to 55)	37<br>(25 to 51)	-6.32%<br>(-10.64 to -2.9)	4<br>(2.8 to 5.7)	1.5<br>(1 to 2.1)	-3.32%<br>(-3.45 to -3.18)
Republic of Moldova	0<br>(0 to 0)	0<br>(0 to 0)	-34.5%<br>(-42.27 to -25.96)	0.2<br>(0.1 to 0.2)	0.1<br>(0.1 to 0.1)	-1.14%<br>(-2.17 to -0.09)
Russian Federation	5<br>(4 to 7)	14<br>(10 to 17)	154.84%<br>(146.58 to 162.39)	0.1<br>(0.1 to 0.1)	0.3<br>(0.2 to 0.4)	5.12%<br>(4.23 to 6.01)
Rwanda	21<br>(15 to 29)	8<br>(6 to 10)	-63.32%<br>(-67.8 to -58.47)	22.3<br>(15.7 to 30)	3.8<br>(2.8 to 5.2)	-6.7%<br>(-7.13 to -6.27)
Saint Lucia	0<br>(0 to 0)	0<br>(0 to 0)	-50.96%<br>(-56.87 to -44.67)	1.6<br>(1.1 to 2)	0.3<br>(0.2 to 0.3)	-6.6%<br>(-7.17 to -6.03)
Saint Vincent and the Grenadines	0<br>(0 to 0)	0<br>(0 to 0)	-39.38%<br>(-45.88 to -31.27)	1.4<br>(1 to 1.7)	0.4<br>(0.3 to 0.5)	-4.34%<br>(-4.48 to -4.2)
Samoa	0<br>(0 to 0)	0<br>(0 to 0)	-34.66%<br>(-42.65 to -24.63)	1.1<br>(0.8 to 1.6)	0.4<br>(0.3 to 0.6)	-3.72%<br>(-4.25 to -3.18)

Location	Incident cases in 1990 (95% UI)	Incident cases in 2019 (95% UI)	Relative change (%, 95 CI)	ASIR in 1990 (95% UI)	ASIR in 2019 (95% UI)	EAPC (%, 95 CI)
Senegal	18 (13 to 24)	16 (11 to 20)	-12.61% (-24.52 to 2.57)	18.7 (13.1 to 24.7)	6.6 (4.7 to 8.7)	-3.88% (-4.11 to -3.65)
Serbia	1 (0 to 1)	0 (0 to 0)	-40.18% (-47 to -32.56)	0.2 (0.1 to 0.2)	0.1 (0.1 to 0.2)	1.07% (-0.34 to 2.5)
Sierra Leone	9 (7 to 12)	11 (8 to 14)	14.33% (2.54 to 31.03)	18.7 (13.4 to 24.2)	10.2 (7.4 to 12.9)	-2.03% (-2.3 to -1.75)
Solomon Islands	0 (0 to 1)	0 (0 to 1)	20.27% (3.56 to 38.78)	8.7 (6.3 to 12.1)	4.2 (3 to 5.8)	-2.56% (-2.59 to -2.53)
Somalia	25 (18 to 34)	44 (31 to 59)	75.32% (52.46 to 101.28)	26 (18.2 to 34.7)	23.6 (16.6 to 31.6)	-0.25% (-0.29 to -0.21)
South Africa	47 (34 to 63)	15 (11 to 19)	-68.75% (-70.85 to -66.77)	7.8 (5.7 to 10.4)	1.1 (0.8 to 1.4)	-6.96% (-7.21 to -6.7)
South Sudan	11 (8 to 14)	21 (15 to 27)	95.45% (69.08 to 122.94)	18.5 (13.2 to 24.4)	14.8 (10.6 to 19.4)	-0.73% (-0.78 to -0.67)
Sri Lanka	3 (2 to 5)	2 (1 to 2)	-48.75% (-55.21 to -40.85)	1.1 (0.7 to 1.5)	0.3 (0.2 to 0.4)	-4.91% (-5.28 to -4.53)
Sudan	28 (21 to 38)	23 (18 to 30)	-18.18% (-29.34 to -5.87)	10.7 (8.2 to 14.5)	3.8 (2.9 to 5.1)	-3.45% (-3.92 to -2.98)
Suriname	0 (0 to 0)	0 (0 to 0)	-22.66% (-31.87 to -10.45)	1.2 (0.9 to 1.5)	0.4 (0.3 to 0.5)	-3.81% (-3.88 to -3.73)

Location	Incident cases in 1990 (95% UI)	Incident cases in 2019 (95% UI)	Relative change (%, 95 CI)	ASIR in 1990 (95% UI)	ASIR in 2019 (95% UI)	EAPC (%, 95 CI)
Syrian Arab Republic	11 (8 to 15)	4 (3 to 6)	-64.36% (-68.95 to -58.48)	7.4 (5.5 to 9.8)	1 (0.7 to 1.3)	-5.66% (-7.1 to -4.19)
Tajikistan	4 (3 to 6)	2 (2 to 3)	-40.6% (-49.01 to -30.1)	4.9 (3.4 to 6.6)	1.2 (0.9 to 1.7)	-4.26% (-4.69 to -3.83)
Thailand	16 (11 to 22)	6 (4 to 8)	-64.53% (-69.5 to -58.76)	1.3 (0.9 to 1.8)	0.2 (0.1 to 0.3)	-7.06% (-8.05 to -6.06)
Timor-Leste	1 (1 to 1)	1 (1 to 1)	5.22% (-7.11 to 23.02)	9 (6.2 to 12.9)	5.2 (3.5 to 7.4)	-1.83% (-2.32 to -1.33)
Togo	10 (7 to 13)	14 (10 to 18)	45.14% (28.61 to 69.53)	22.9 (16.1 to 30.3)	10.6 (7.7 to 13.7)	-2.38% (-2.77 to -2)
Tonga	0 (0 to 0)	0 (0 to 0)	-63.09% (-67.79 to -58.42)	3.2 (2.3 to 4.5)	0.9 (0.6 to 1.3)	-4.58% (-4.8 to -4.36)
Tunisia	4 (3 to 5)	2 (2 to 3)	-47.4% (-54.13 to -39.99)	2.8 (2.1 to 3.7)	0.6 (0.4 to 0.8)	-4.52% (-5.38 to -3.66)
Turkey	13 (9 to 18)	10 (7 to 14)	-23.44% (-31.94 to -14.4)	1.2 (0.9 to 1.7)	0.4 (0.3 to 0.6)	-2.57% (-3.47 to -1.65)
Turkmenistan	1 (1 to 1)	1 (1 to 1)	-23.86% (-34.03 to -11.73)	1.9 (1.3 to 2.6)	0.6 (0.4 to 0.8)	-3.71% (-3.95 to -3.46)
Tuvalu	0 (0 to 0)	0 (0 to 0)	-67.67% (-71.69 to -63.17)	2.6 (1.8 to 3.6)	0.6 (0.4 to 0.9)	-4.98% (-5.11 to -4.85)

Location	Incident cases in 1990 (95% UI)	Incident cases in 2019 (95% UI)	Relative change (%, 95 CI)	ASIR in 1990 (95% UI)	ASIR in 2019 (95% UI)	EAPC (%, 95 CI)
Uganda	36 (26 to 48)	28 (20 to 38)	-21.66% (-31.82 to -11.35)	18.3 (13.1 to 24.1)	5.8 (4.2 to 7.8)	-4.38% (-4.82 to -3.93)
Ukraine	2 (1 to 3)	5 (3 to 6)	124.51% (99.65 to 154.68)	0.1 (0.1 to 0.1)	0.3 (0.2 to 0.4)	5.62% (4.07 to 7.2)
United Republic of Tanzania	66 (46 to 87)	56 (39 to 76)	-14.92% (-26.47 to -2.98)	19.6 (13.7 to 26)	6.8 (4.8 to 9.2)	-3.71% (-3.94 to -3.48)
Uzbekistan	6 (4 to 8)	2 (1 to 2)	-69.68% (-73.98 to -64.76)	1.8 (1.3 to 2.5)	0.2 (0.2 to 0.3)	-7.35% (-7.79 to -6.91)
Vanuatu	0 (0 to 0)	0 (0 to 0)	17.31% (2.6 to 32.88)	6.1 (4.4 to 8.6)	2.7 (1.9 to 3.7)	-3.04% (-3.17 to -2.91)
Venezuela (Bolivarian Republic of)	9 (7 to 11)	6 (5 to 7)	-33.27% (-41.8 to -20.04)	3.2 (2.4 to 3.9)	0.8 (0.6 to 0.9)	-4.67% (-4.91 to -4.42)
Viet Nam	10 (7 to 14)	3 (2 to 4)	-72.7% (-76.63 to -68.71)	0.9 (0.6 to 1.3)	0.1 (0.1 to 0.1)	-8.15% (-8.52 to -7.79)
Yemen	20 (15 to 27)	31 (23 to 40)	52.08% (33.12 to 74.73)	14.3 (10.4 to 19.2)	7.5 (5.6 to 9.9)	-2.17% (-2.27 to -2.06)
Zambia	17 (12 to 22)	10 (7 to 13)	-40.72% (-47.61 to -32.99)	17.2 (12.1 to 23.2)	4.5 (3.1 to 6.1)	-4.58% (-4.99 to -4.16)

Location	Incident cases in 1990 (95% UI)	Incident cases in 2019 (95% UI)	Relative change (%, 95 CI)	ASIR in 1990 (95% UI)	ASIR in 2019 (95% UI)	EAPC (%, 95 CI)
Zimbabwe	14 (10 to 19)	6 (5 to 8)	-55.06% (-60.89 to -49.69)	11.2 (8.1 to 14.8)	2.9 (2.1 to 3.8)	-4.04% (-4.27 to -3.81)

ASIR – age-standardized incidence rate (per 100,000 population), CI – confidence interval, EAPC – estimated annual percentage change, MSMI – maternal sepsis and other maternal infections, UI – uncertain interval.

Table S16. MMR of MSMI in 131 low- and middle-income countries and territories with EAPC in age group of 0-14 years from 1990 to 2019.

Location	Deaths in 1990 (95% UI)	Deaths in 2019 (95% UI)	Relative change (%, 95 CI)	ASMMR in 1990 (95% UI)	ASMMR in 2019 (95% UI)	EAPC (%, 95 CI)
Afghanistan	1<br>(0 to 4)	1<br>(0 to 3)	-2.59%<br>(-78.9 to 346.9)	352.1<br>(98.4 to 908.5)	139.8<br>(38.7 to 365.4)	-3.98%<br>(-4.96 to -3)
Albania	0<br>(0 to 0)	0<br>(0 to 0)	-61.02%<br>(-82.74 to -16.38)	2.5<br>(1.4 to 4.2)	2.7<br>(1.4 to 4.5)	0.6%<br>(-0.33 to 1.53)
Algeria	4<br>(1 to 9)	0<br>(0 to 1)	-94.76%<br>(-98.96 to -74.05)	1688.7<br>(437 to 4244)	166.2<br>(43.9 to 458.7)	-7.64%<br>(-8.07 to -7.2)
American Samoa	0<br>(0 to 0)	0<br>(0 to 0)	-48.29%<br>(-89.98 to 144.05)	3.7<br>(1.2 to 9.7)	2<br>(0.5 to 5.8)	-2.35%<br>(-2.62 to -2.07)
Angola	0<br>(0 to 1)	1<br>(0 to 2)	42.88%<br>(-72.16 to 674.93)	36<br>(7.8 to 105.4)	21.1<br>(5.4 to 54.3)	-1.51%<br>(-1.99 to -1.02)
Armenia	0<br>(0 to 0)	0<br>(0 to 0)	-87.24%<br>(-94.11 to -74.49)	3.4<br>(1.9 to 5.7)	1.4<br>(0.7 to 2.3)	-3.96%<br>(-5.15 to -2.75)
Azerbaijan	0<br>(0 to 0)	0<br>(0 to 0)	-82.82%<br>(-93.53 to -53.88)	77.9<br>(34 to 150.1)	13.5<br>(5.9 to 28.6)	-8.3%<br>(-9.29 to -7.3)
Bangladesh	4<br>(1 to 9)	0<br>(0 to 0)	-95.88%<br>(-99.05 to -82.41)	52<br>(16 to 117.3)	2.8<br>(0.8 to 7.6)	-8.36%<br>(-9.69 to -7)

Location	Deaths in 1990 (95% UI)	Deaths in 2019 (95% UI)	Relative change (%, 95 CI)	ASMMR in 1990 (95% UI)	ASMMR in 2019 (95% UI)	EAPC (%, 95 CI)
Belarus	0<br>(0 to 0)	0<br>(0 to 0)	-84.65%<br>(-92.43 to -68.61)	9.5<br>(5.3 to 15.8)	4.3<br>(2.2 to 7.6)	-3.13%<br>(-3.7 to -2.56)
Belize	0<br>(0 to 0)	0<br>(0 to 0)	-45.48%<br>(-73.16 to 15.46)	11<br>(6.3 to 17.6)	4.8<br>(2.7 to 7.9)	-2.58%<br>(-3.31 to -1.85)
Benin	0<br>(0 to 1)	0<br>(0 to 1)	-8.33%<br>(-81.55 to 361.38)	28.9<br>(7.2 to 78.8)	10.8<br>(2.5 to 29.9)	-2.51%<br>(-3.04 to -1.97)
Bhutan	0<br>(0 to 0)	0<br>(0 to 0)	-82.76%<br>(-96.82 to 63.44)	16.8<br>(2.1 to 48.7)	5.9<br>(1.5 to 16.4)	-3.49%<br>(-4.19 to -2.77)
Bolivia (Plurinational State of)	1<br>(0 to 2)	0<br>(0 to 0)	-81.24%<br>(-95.73 to -8.82)	86.4<br>(24.3 to 218.6)	15<br>(3.6 to 39.3)	-6.53%<br>(-6.73 to -6.33)
Bosnia and Herzegovina	0<br>(0 to 0)	0<br>(0 to 0)	-98.11%<br>(-99.38 to -93.34)	56.9<br>(17 to 146.5)	4.7<br>(2.7 to 7.7)	-10.28%<br>(-11.87 to -8.66)
Botswana	0<br>(0 to 0)	0<br>(0 to 0)	-70.92%<br>(-93.06 to 34.03)	231.2<br>(64.3 to 615.2)	80.2<br>(21.6 to 209.5)	-3.57%<br>(-4.37 to -2.77)
Brazil	3<br>(2 to 5)	2<br>(1 to 2)	-52.41%<br>(-74.66 to -11.25)	17.8<br>(11.3 to 26.1)	11.6<br>(7.1 to 17.9)	-0.44%<br>(-0.86 to -0.02)
Bulgaria	0<br>(0 to 0)	0<br>(0 to 0)	-95.01%<br>(-97.73 to -88.69)	1<br>(0.5 to 1.7)	0.1<br>(0.1 to 0.2)	-7.9%<br>(-8.8 to -6.98)

Location	Deaths in 1990 (95% UI)	Deaths in 2019 (95% UI)	Relative change (%, 95 CI)	ASMMR in 1990 (95% UI)	ASMMR in 2019 (95% UI)	EAPC (%, 95 CI)
Burkina Faso	1<br>(0 to 2)	1<br>(0 to 2)	39.39%<br>(-73.91 to 643.44)	43.9<br>(11 to 112.1)	32.6<br>(8.2 to 88.3)	-1.3%<br>(-1.56 to -1.04)
Burundi	0<br>(0 to 1)	1<br>(0 to 3)	161.87%<br>(-52.37 to 1157.81)	71.3<br>(16.9 to 196.6)	100.4<br>(26.7 to 282.6)	2.46%<br>(1.12 to 3.82)
Cabo Verde	0<br>(0 to 0)	0<br>(0 to 0)	-94.22%<br>(-98.8 to -69.19)	11.9<br>(3 to 31.1)	1.1<br>(0.3 to 3)	-9.24%<br>(-10.11 to -8.37)
Cambodia	0<br>(0 to 1)	0<br>(0 to 0)	-91.62%<br>(-98.34 to -55.23)	97.3<br>(24.4 to 266.4)	10<br>(2.7 to 27.4)	-8.67%<br>(-9.86 to -7.47)
Cameroon	1<br>(0 to 3)	1<br>(0 to 3)	27.45%<br>(-74.31 to 528.8)	63.3<br>(17.1 to 167.4)	40<br>(9.7 to 103.4)	-1.62%<br>(-2.08 to -1.17)
Central African Republic	0<br>(0 to 1)	1<br>(0 to 2)	121.86%<br>(-51.16 to 1047.6)	148.6<br>(34.3 to 404.9)	179.3<br>(50 to 444.9)	0.86%<br>(0.68 to 1.05)
Chad	2<br>(0 to 5)	3<br>(1 to 9)	85.23%<br>(-60.37 to 787.61)	173.1<br>(43.2 to 447)	117.8<br>(32.6 to 297.2)	-1.11%<br>(-1.43 to -0.79)
China	8<br>(6 to 11)	0<br>(0 to 0)	-99.16%<br>(-99.44 to -98.74)	127.7<br>(93.1 to 173.7)	1.8<br>(1.4 to 2.4)	-14.67%<br>(-15.35 to -13.99)
Colombia	1<br>(0 to 1)	0<br>(0 to 0)	-72.72%<br>(-86.84 to -46.06)	24.2<br>(14.5 to 37)	8.1<br>(4.5 to 13.2)	-3.34%<br>(-3.81 to -2.88)
Comoros	0<br>(0 to 0)	0<br>(0 to 0)	-35.11%<br>(-88.16 to 691.22)	9.1<br>(0.8 to 28.4)	9<br>(2.2 to 23.6)	-0.97%<br>(-1.71 to -0.22)

Location	Deaths in 1990 (95% UI)	Deaths in 2019 (95% UI)	Relative change (%, 95 CI)	ASMMR in 1990 (95% UI)	ASMMR in 2019 (95% UI)	EAPC (%, 95 CI)
Congo	0<br>(0 to 0)	0<br>(0 to 0)	-6.85%<br>(-81.32 to 428.01)	34.2<br>(6.7 to 100.8)	24.2<br>(6.4 to 62.9)	-0.61%<br>(-1.58 to 0.37)
Costa Rica	0<br>(0 to 0)	0<br>(0 to 0)	-88.92%<br>(-95.13 to -76.89)	4.1<br>(2.3 to 7)	0.6<br>(0.3 to 0.9)	-7.04%<br>(-8.4 to -5.66)
Côte d'Ivoire	1<br>(0 to 2)	1<br>(0 to 1)	-11%<br>(-81.27 to 389.13)	23.2<br>(5.3 to 60.7)	13.2<br>(3.5 to 38.2)	-1.42%<br>(-1.86 to -0.98)
Cuba	0<br>(0 to 0)	0<br>(0 to 0)	-78.08%<br>(-89.52 to -52.79)	0.7<br>(0.4 to 1.1)	0.3<br>(0.2 to 0.5)	-3.01%<br>(-3.55 to -2.48)
Democratic People's Republic of Korea	0<br>(0 to 1)	0<br>(0 to 0)	-69.02%<br>(-93.99 to 66.9)	153.7<br>(34.6 to 420.3)	143.8<br>(37.9 to 370.9)	0.42%<br>(-0.52 to 1.37)
Democratic Republic of the Congo	4<br>(1 to 12)	15<br>(4 to 39)	230.52%<br>(-32.9 to 1655.5)	108<br>(24.9 to 292.6)	215.7<br>(59.4 to 573.2)	4.5%<br>(3.16 to 5.86)
Djibouti	0<br>(0 to 0)	0<br>(0 to 0)	72.96%<br>(-63.64 to 727.79)	103.7<br>(24.5 to 295.8)	152.1<br>(36 to 391.4)	1.71%<br>(1.17 to 2.26)
Dominica	0<br>(0 to 0)	0<br>(0 to 0)	-21.9%<br>(-72.2 to 113.65)	17.9<br>(8.5 to 33)	33.1<br>(15.2 to 65.4)	4.77%<br>(3.63 to 5.91)
Dominican Republic	0<br>(0 to 0)	0<br>(0 to 0)	-56.33%<br>(-89.84 to 54.77)	18<br>(7.4 to 38)	8.2<br>(2.2 to 20.9)	-1.94%<br>(-3.16 to -0.71)

Location	Deaths in 1990 (95% UI)	Deaths in 2019 (95% UI)	Relative change (%, 95 CI)	ASMMR in 1990 (95% UI)	ASMMR in 2019 (95% UI)	EAPC (%, 95 CI)
Ecuador	0 (0 to 0)	0 (0 to 0)	-75.33% (-89.67 to -37.52)	43.7 (22.3 to 73.2)	8.7 (4 to 16.5)	-5.23% (-5.87 to -4.58)
Egypt	0 (0 to 1)	0 (0 to 0)	-53.76% (-91.11 to 109.73)	19.2 (5.6 to 47.1)	9.1 (2.1 to 25.7)	-2.53% (-4.07 to -0.97)
El Salvador	1 (0 to 2)	0 (0 to 0)	-98.25% (-99.67 to -91.72)	162.5 (42.3 to 431.6)	4.8 (1.2 to 13.3)	-10.59% (-12.31 to -8.84)
Equatorial Guinea	0 (0 to 0)	0 (0 to 0)	-87.14% (-97.62 to -22.37)	144.5 (33 to 406)	9 (2.1 to 25.7)	-10.36% (-10.87 to -9.83)
Eritrea	1 (0 to 2)	1 (0 to 2)	13.25% (-78.03 to 443.6)	161.3 (37.8 to 448.7)	142 (37.5 to 370.8)	0.54% (0 to 1.09)
Eswatini	0 (0 to 0)	0 (0 to 0)	-60.16% (-91.15 to 108.21)	20.8 (5.6 to 53)	10.6 (3 to 26.9)	-1.52% (-2.14 to -0.89)
Ethiopia	6 (2 to 14)	3 (1 to 7)	-46.63% (-80.57 to 66.93)	80.7 (30.5 to 173.7)	29.9 (12.7 to 60.8)	-3.66% (-4.55 to -2.76)
Fiji	0 (0 to 0)	0 (0 to 0)	-32.29% (-87.54 to 240.62)	35.2 (9.3 to 93.8)	33.5 (8.4 to 92)	-1.22% (-1.99 to -0.44)
Gabon	0 (0 to 0)	0 (0 to 0)	-72.08% (-94.7 to 45.54)	36.1 (7.4 to 102.7)	11.1 (2.8 to 28.7)	-4.2% (-4.82 to -3.57)
Gambia	0 (0 to 0)	0 (0 to 0)	-8.63% (-80.03 to 347.01)	75.9 (18.8 to 199.4)	48.6 (12.2 to 127.9)	-1.67% (-2.1 to -1.23)

Location	Deaths in 1990 (95% UI)	Deaths in 2019 (95% UI)	Relative change (%, 95 CI)	ASMMR in 1990 (95% UI)	ASMMR in 2019 (95% UI)	EAPC (%, 95 CI)
Georgia	0<br>(0 to 0)	0<br>(0 to 0)	-61.17%<br>(-83.32 to -16.4)	0.6<br>(0.3 to 1)	0.6<br>(0.3 to 1.1)	-1.79%<br>(-3.36 to -0.19)
Ghana	0<br>(0 to 1)	0<br>(0 to 0)	-54.46%<br>(-90.39 to 157.49)	15.2<br>(3.8 to 40.9)	5.8<br>(1.5 to 14.4)	-3.23%<br>(-3.65 to -2.82)
Global	151<br>(114 to 199)	76<br>(55 to 108)	-49.93%<br>(-66.57 to -26.1)	77.4<br>(58.6 to 101.9)	35.7<br>(26.1 to 51.1)	-2.37%<br>(-2.55 to -2.19)
Grenada	0<br>(0 to 0)	0<br>(0 to 0)	-86.34%<br>(-93.31 to -71.37)	2<br>(1.1 to 3.3)	0.6<br>(0.3 to 0.9)	-2.56%<br>(-3.39 to -1.72)
Guatemala	1<br>(0 to 1)	0<br>(0 to 0)	-88.78%<br>(-94.25 to -76.8)	56.9<br>(31.5 to 92.7)	5.1<br>(2.9 to 8.5)	-7.86%<br>(-9.1 to -6.6)
Guinea	1<br>(0 to 3)	1<br>(0 to 3)	4.22%<br>(-79.61 to 454.55)	68.6<br>(16.6 to 180.4)	39.5<br>(9.9 to 106.1)	-1.41%<br>(-1.69 to -1.14)
Guinea-Bissau	0<br>(0 to 0)	0<br>(0 to 0)	-54.07%<br>(-90.07 to 123.06)	96<br>(26.5 to 246.7)	34.9<br>(9.4 to 87.7)	-3.08%<br>(-3.63 to -2.53)
Guyana	0<br>(0 to 0)	0<br>(0 to 0)	-76.32%<br>(-89.56 to -47.6)	8.6<br>(4.6 to 14)	4.2<br>(2.3 to 7.2)	0.06%<br>(-1.16 to 1.29)
Haiti	1<br>(0 to 2)	1<br>(0 to 2)	31.59%<br>(-74.98 to 690.01)	98.7<br>(21.2 to 270.2)	96.5<br>(23.7 to 253.6)	0.7%<br>(0.48 to 0.91)
Honduras	2<br>(0 to 4)	0<br>(0 to 0)	-94.39%<br>(-98.86 to -69.61)	250.4<br>(71.7 to 594)	11<br>(2.7 to 29.4)	-11.16%<br>(-11.5 to -10.81)

Location	Deaths in 1990 (95% UI)	Deaths in 2019 (95% UI)	Relative change (%, 95 CI)	ASMMR in 1990 (95% UI)	ASMMR in 2019 (95% UI)	EAPC (%, 95 CI)
India	60<br>(33 to 100)	4<br>(2 to 9)	-92.48%<br>(-96.62 to -83.16)	226.9<br>(125 to 380.4)	30.3<br>(13.6 to 57.7)	-8.17%<br>(-8.69 to -7.65)
Indonesia	1<br>(0 to 2)	0<br>(0 to 0)	-93.02%<br>(-97.13 to -83.76)	16.6<br>(8 to 30.9)	2.2<br>(1.1 to 3.8)	-6.47%<br>(-6.59 to -6.35)
Iran (Islamic Republic of)	1<br>(0 to 1)	0<br>(0 to 0)	-93.61%<br>(-97.12 to -86.69)	27<br>(15.3 to 44.6)	3.4<br>(1.8 to 5.9)	-7.45%<br>(-7.8 to -7.09)
Iraq	0<br>(0 to 0)	0<br>(0 to 0)	-74.51%<br>(-95.14 to 54.39)	3<br>(0.7 to 8.5)	0.7<br>(0.2 to 1.9)	-4%<br>(-4.83 to -3.16)
Jamaica	0<br>(0 to 0)	0<br>(0 to 0)	-94.11%<br>(-97.36 to -87.09)	2.8<br>(1.6 to 4.5)	0.4<br>(0.2 to 0.6)	-9.69%<br>(-11.49 to -7.86)
Jordan	0<br>(0 to 0)	0<br>(0 to 0)	-52.83%<br>(-90.5 to 122.39)	98.4<br>(26.5 to 271.5)	31.1<br>(7.7 to 82.7)	-5.24%<br>(-5.92 to -4.55)
Kazakhstan	0<br>(0 to 0)	0<br>(0 to 0)	18.38%<br>(-42.78 to 153.7)	2.3<br>(1.2 to 3.7)	4.3<br>(2.4 to 6.9)	2.59%<br>(2.38 to 2.79)
Kenya	1<br>(1 to 2)	1<br>(0 to 1)	-47.93%<br>(-74.85 to 11.85)	37.3<br>(18 to 66.3)	15.7<br>(7.8 to 27.2)	-1.88%<br>(-2.44 to -1.31)
Kiribati	0<br>(0 to 0)	0<br>(0 to 0)	-2.58%<br>(-78.49 to 288.77)	30<br>(8.9 to 75.9)	24.9<br>(6.9 to 66.6)	-0.85%<br>(-1.07 to -0.62)
Kyrgyzstan	0<br>(0 to 0)	0<br>(0 to 0)	12%<br>(-41.49 to 125.83)	21.3<br>(12.2 to 34.1)	24.6<br>(14.7 to 37.3)	1.23%<br>(0.55 to 1.92)

Location	Deaths in 1990 (95% UI)	Deaths in 2019 (95% UI)	Relative change (%, 95 CI)	ASMMR in 1990 (95% UI)	ASMMR in 2019 (95% UI)	EAPC (%, 95 CI)
Lao People's Democratic Republic	0<br>(0 to 1)	0<br>(0 to 0)	-96.77%<br>(-99.37 to -84.82)	468.1<br>(127.6 to 1308.1)	15.1<br>(3.5 to 38.5)	-11.08%<br>(-11.52 to -10.62)
Lebanon	0<br>(0 to 0)	0<br>(0 to 0)	-90.1%<br>(-97.75 to -52.76)	13.7<br>(3.8 to 38.2)	1.6<br>(0.5 to 4.3)	-7.45%<br>(-7.97 to -6.92)
Lesotho	0<br>(0 to 1)	0<br>(0 to 0)	-73.55%<br>(-95.06 to 33.15)	174.5<br>(43.5 to 452.5)	68.7<br>(16.9 to 172.5)	-1.92%<br>(-2.54 to -1.3)
Liberia	1<br>(0 to 1)	1<br>(0 to 1)	-2.57%<br>(-79.02 to 383.95)	248.5<br>(64.3 to 687.7)	157.8<br>(44.5 to 409)	-2.94%<br>(-3.83 to -2.04)
Libya	0<br>(0 to 0)	0<br>(0 to 0)	-87.66%<br>(-96.26 to -48.41)	6.7<br>(1.7 to 18.5)	1.8<br>(0.9 to 4)	-4.51%<br>(-5.49 to -3.52)
Madagascar	2<br>(0 to 4)	1<br>(0 to 4)	-12.89%<br>(-83.44 to 319.34)	81.3<br>(20 to 202.7)	39.9<br>(9.3 to 104.9)	-1.82%<br>(-2.23 to -1.42)
Malawi	1<br>(0 to 2)	1<br>(0 to 1)	1.68%<br>(-78.15 to 409.17)	29.1<br>(6.9 to 85.7)	18.9<br>(4.7 to 49.6)	-1.04%<br>(-1.48 to -0.59)
Malaysia	0<br>(0 to 0)	0<br>(0 to 0)	-84.7%<br>(-95.88 to -30.84)	12.3<br>(3.3 to 31.8)	2.6<br>(1.2 to 5.7)	-6.36%<br>(-7.01 to -5.71)
Maldives	0<br>(0 to 0)	0<br>(0 to 0)	-96.35%<br>(-99.23 to -83.6)	18.2<br>(4.8 to 50.3)	1.2<br>(0.3 to 2.9)	-9.04%<br>(-10.37 to -7.68)

Location	Deaths in 1990 (95% UI)	Deaths in 2019 (95% UI)	Relative change (%, 95 CI)	ASMMR in 1990 (95% UI)	ASMMR in 2019 (95% UI)	EAPC (%, 95 CI)
Mali	4 (1 to 11)	4 (1 to 11)	-2.21% (-81.1 to 365.55)	268 (73.6 to 685)	108.7 (27 to 292.9)	-3.09% (-3.31 to -2.87)
Marshall Islands	0 (0 to 0)	0 (0 to 0)	-57.88% (-90.81 to 106.21)	6.1 (1.5 to 16.2)	3.4 (1.1 to 9.5)	-1.93% (-2.49 to -1.37)
Mauritania	0 (0 to 1)	0 (0 to 0)	-70.34% (-94.05 to 70.68)	147.4 (38.5 to 384.6)	33.7 (8.1 to 96.1)	-5.79% (-6.19 to -5.39)
Mexico	1 (0 to 1)	0 (0 to 0)	-74.16% (-87.06 to -47.03)	8.7 (5.3 to 13.3)	2.8 (1.7 to 4.5)	-3.22% (-4.44 to -1.99)
Micronesia (Federated States of)	0 (0 to 0)	0 (0 to 0)	-81.28% (-97.3 to 2.3)	4.2 (1.1 to 11.5)	1.4 (0.2 to 4.1)	-3.92% (-4.1 to -3.75)
Mongolia	0 (0 to 0)	0 (0 to 0)	-86.18% (-95.74 to -37.91)	14 (3.7 to 37.8)	2.5 (1.2 to 5.4)	-7.16% (-7.96 to -6.36)
Montenegro	0 (0 to 0)	0 (0 to 0)	-64.29% (-84.67 to -13.46)	0.2 (0.1 to 0.3)	0.2 (0.1 to 0.3)	-0.55% (-1.02 to -0.09)
Morocco	1 (0 to 2)	0 (0 to 0)	-91.55% (-98.32 to -54.03)	132.6 (30 to 361.6)	15.5 (3.6 to 41.7)	-7.24% (-7.52 to -6.97)
Mozambique	1 (0 to 4)	1 (0 to 2)	-54.26% (-91.01 to 128.8)	55.7 (12.7 to 156.2)	13.4 (3.4 to 37.6)	-4.03% (-4.55 to -3.51)

Location	Deaths in 1990 (95% UI)	Deaths in 2019 (95% UI)	Relative change (%, 95 CI)	ASMMR in 1990 (95% UI)	ASMMR in 2019 (95% UI)	EAPC (%, 95 CI)
Myanmar	1 (0 to 2)	0 (0 to 1)	-76.37% (-94.57 to 21.85)	118.9 (30.4 to 309.1)	37.5 (11.9 to 96.6)	-4.22% (-4.8 to -3.64)
Namibia	0 (0 to 0)	0 (0 to 0)	-67.03% (-94.04 to 110.17)	27.4 (4.9 to 75.4)	7.9 (1.8 to 22.2)	-3.16% (-3.65 to -2.67)
Nepal	1 (0 to 2)	0 (0 to 0)	-96.11% (-99.2 to -82.46)	78.2 (22.4 to 201.9)	3.2 (0.7 to 9.3)	-10.72% (-11.02 to -10.43)
Nicaragua	0 (0 to 1)	0 (0 to 0)	-92.81% (-98.24 to -73.17)	32.4 (11.7 to 70.7)	2.6 (0.8 to 6.2)	-9.15% (-9.45 to -8.85)
Niger	2 (1 to 5)	4 (1 to 11)	85.18% (-61.72 to 867.02)	136.6 (35.5 to 364.1)	92.2 (23 to 264.4)	-1.04% (-1.27 to -0.81)
Nigeria	6 (3 to 11)	7 (3 to 14)	21.99% (-53.95 to 202.83)	44.9 (20.7 to 80.6)	30.3 (12.7 to 60)	-1.88% (-2.6 to -1.16)
North Macedonia	0 (0 to 0)	0 (0 to 0)	-84.72% (-93.99 to -57.54)	1.3 (0.5 to 2.4)	0.6 (0.3 to 1)	-3.97% (-4.54 to -3.4)
Pakistan	5 (2 to 12)	3 (1 to 6)	-39.07% (-80.15 to 98.46)	103.8 (36.6 to 246.1)	44 (18 to 90.5)	-2.69% (-2.89 to -2.48)
Papua New Guinea	0 (0 to 1)	0 (0 to 0)	-29.27% (-86.3 to 252.2)	172.5 (47.1 to 496.5)	68.1 (19.5 to 181)	-2.8% (-3.28 to -2.32)
Paraguay	0 (0 to 0)	0 (0 to 0)	-86.93% (-96.89 to -54.38)	77.7 (28.5 to 166.3)	10.4 (2.9 to 25)	-7.47% (-8.54 to -6.38)

Location	Deaths in 1990 (95% UI)	Deaths in 2019 (95% UI)	Relative change (%, 95 CI)	ASMMR in 1990 (95% UI)	ASMMR in 2019 (95% UI)	EAPC (%, 95 CI)
Peru	2<br>(0 to 5)	0<br>(0 to 0)	-92.1%<br>(-98.32 to -61.56)	180.3<br>(47.6 to 479.8)	17.1<br>(4.8 to 47)	-7.97%<br>(-8.36 to -7.59)
Philippines	0<br>(0 to 0)	0<br>(0 to 0)	-73.64%<br>(-87.48 to -44.55)	24.7<br>(13.3 to 40.4)	4.7<br>(2.5 to 7.8)	-4.54%<br>(-5.21 to -3.87)
Republic of Moldova	0<br>(0 to 0)	0<br>(0 to 0)	-91.07%<br>(-95.61 to -81.28)	11.4<br>(6.5 to 18)	3.3<br>(1.9 to 5.3)	-4.76%<br>(-5.69 to -3.82)
Russian Federation	0<br>(0 to 0)	0<br>(0 to 0)	-88.81%<br>(-94.31 to -78.54)	38.3<br>(23.2 to 58.6)	11<br>(6.1 to 17.7)	-5.53%<br>(-6.09 to -4.96)
Rwanda	1<br>(0 to 3)	0<br>(0 to 1)	-63.4%<br>(-91.7 to 65.85)	147.2<br>(42.9 to 384.7)	53.9<br>(15.8 to 140)	-2.71%<br>(-3.95 to -1.45)
Saint Lucia	0<br>(0 to 0)	0<br>(0 to 0)	-69.69%<br>(-85.71 to -31.82)	1.8<br>(1 to 2.8)	1.4<br>(0.8 to 2.4)	0.3%<br>(-0.53 to 1.13)
Saint Vincent and the Grenadines	0<br>(0 to 0)	0<br>(0 to 0)	-61.17%<br>(-80.8 to -18.74)	0.6<br>(0.3 to 1)	0.5<br>(0.3 to 0.8)	0.5%<br>(-0.5 to 1.51)
Samoa	0<br>(0 to 0)	0<br>(0 to 0)	-79.9%<br>(-94.21 to -9.92)	52.1<br>(13.7 to 143.7)	8.5<br>(3.7 to 19.6)	-7.36%<br>(-8.06 to -6.65)
Senegal	1<br>(0 to 2)	0<br>(0 to 1)	-36.76%<br>(-88.85 to 185.94)	72.2<br>(18.6 to 196.1)	39.3<br>(9.7 to 109.6)	-1.58%<br>(-1.93 to -1.23)
Serbia	0<br>(0 to 0)	0<br>(0 to 0)	-72.35%<br>(-92.42 to -8.81)	6<br>(2.3 to 13.5)	4.5<br>(1.9 to 10)	-1.01%<br>(-1.79 to -0.21)

Location	Deaths in 1990 (95% UI)	Deaths in 2019 (95% UI)	Relative change (%, 95 CI)	ASMMR in 1990 (95% UI)	ASMMR in 2019 (95% UI)	EAPC (%, 95 CI)
Sierra Leone	1 (0 to 2)	1 (0 to 2)	58.82% (-64.51 to 651.43)	114.9 (30 to 313.2)	97.5 (25.1 to 250.6)	-0.76% (-1.37 to -0.15)
Solomon Islands	0 (0 to 0)	0 (0 to 0)	29.78% (-75.54 to 507.43)	21.2 (5.9 to 55.1)	21.6 (5.1 to 58.1)	0.76% (-0.06 to 1.58)
Somalia	1 (0 to 2)	3 (1 to 8)	381.33% (-13.28 to 2501.28)	55.5 (11.8 to 158.9)	115.5 (28.9 to 295)	2.97% (2.54 to 3.41)
South Africa	1 (0 to 1)	0 (0 to 0)	-93.95% (-98.19 to -82.85)	24.6 (12.4 to 44)	1.9 (0.6 to 4.3)	-6.28% (-9.21 to -3.26)
South Sudan	0 (0 to 0)	0 (0 to 1)	81.02% (-64.04 to 784.37)	15.5 (3.7 to 42.7)	17.2 (4 to 47.7)	-0.1% (-0.45 to 0.24)
Sri Lanka	0 (0 to 0)	0 (0 to 0)	-89.66% (-97.73 to -45.84)	16.2 (4.2 to 47.9)	2.8 (0.9 to 7.2)	-5.75% (-6.33 to -5.17)
Sudan	2 (1 to 6)	1 (0 to 3)	-48.72% (-90.28 to 165.97)	290.4 (74.5 to 771.6)	135.5 (35.3 to 358.5)	-2.22% (-2.64 to -1.79)
Suriname	0 (0 to 0)	0 (0 to 0)	-37.51% (-86.76 to 210.38)	3 (0.7 to 8.5)	2 (0.5 to 5.1)	0.81% (-0.53 to 2.16)
Syrian Arab Republic	0 (0 to 0)	0 (0 to 0)	-91.23% (-97.05 to -75.61)	27 (12 to 54.7)	4.6 (1.9 to 9.3)	-7.63% (-8.85 to -6.39)
Tajikistan	0 (0 to 0)	0 (0 to 0)	-17.35% (-65.38 to 118.17)	6.5 (2.4 to 14)	5.1 (2.6 to 8.7)	-2.54% (-3.37 to -1.7)

Location	Deaths in 1990 (95% UI)	Deaths in 2019 (95% UI)	Relative change (%, 95 CI)	ASMMR in 1990 (95% UI)	ASMMR in 2019 (95% UI)	EAPC (%, 95 CI)
Thailand	0<br>(0 to 0)	0<br>(0 to 0)	-82.72%<br>(-96.09 to -15.68)	1.6<br>(0.4 to 4.4)	0.5<br>(0.2 to 1.3)	-2.5%<br>(-3.21 to -1.78)
Timor-Leste	0<br>(0 to 0)	0<br>(0 to 0)	-73.18%<br>(-94.84 to 45.83)	256.5<br>(60.1 to 745.4)	60.5<br>(14 to 170)	-6.4%<br>(-7.36 to -5.44)
Togo	0<br>(0 to 1)	0<br>(0 to 0)	-34.8%<br>(-85.52 to 228.42)	53.5<br>(14.1 to 140.1)	28.1<br>(7.2 to 70.3)	-1.89%<br>(-2.23 to -1.54)
Tonga	0<br>(0 to 0)	0<br>(0 to 0)	-60.43%<br>(-92.68 to 76.3)	9<br>(2.3 to 24.8)	5.3<br>(1.4 to 15.1)	-2.62%<br>(-3.57 to -1.66)
Tunisia	0<br>(0 to 0)	0<br>(0 to 0)	-93.92%<br>(-98.65 to -71.91)	21.1<br>(5.6 to 55)	4.7<br>(1.2 to 12.6)	-3.82%<br>(-4.62 to -3.01)
Turkey	0<br>(0 to 0)	0<br>(0 to 0)	-90.7%<br>(-97.54 to -49.95)	10.7<br>(2.4 to 28.3)	2.6<br>(1.2 to 6.3)	-5.17%<br>(-5.72 to -4.62)
Turkmenistan	0<br>(0 to 0)	0<br>(0 to 0)	-35.38%<br>(-65.17 to 20.84)	3.9<br>(2.2 to 6.2)	3<br>(1.7 to 4.8)	-1.67%<br>(-2.42 to -0.91)
Tuvalu	0<br>(0 to 0)	0<br>(0 to 0)	-81.03%<br>(-95.87 to -11.66)	29.8<br>(8.1 to 74.7)	5.7<br>(1.8 to 15.4)	-5.29%<br>(-5.41 to -5.17)
Uganda	1<br>(0 to 2)	1<br>(0 to 3)	73.04%<br>(-61.69 to 878.31)	20.4<br>(4.1 to 56.4)	20.8<br>(6 to 52.6)	0.54%<br>(-0.16 to 1.24)
Ukraine	0<br>(0 to 0)	0<br>(0 to 0)	-4.82%<br>(-50.37 to 90.03)	4.7<br>(2.5 to 7.8)	12.5<br>(6.5 to 20.2)	4.17%<br>(3.6 to 4.74)

Location	Deaths in 1990 (95% UI)	Deaths in 2019 (95% UI)	Relative change (%, 95 CI)	ASMMR in 1990 (95% UI)	ASMMR in 2019 (95% UI)	EAPC (%, 95 CI)
United Republic of Tanzania	2<br>(0 to 4)	2<br>(0 to 5)	15.71%<br>(-77.09 to 427.48)	38.8<br>(10.3 to 99.6)	25<br>(6.2 to 64.7)	0.19%<br>(-0.48 to 0.87)
Uzbekistan	0<br>(0 to 0)	0<br>(0 to 0)	-62.85%<br>(-82.93 to -22.02)	18.8<br>(10.6 to 30.6)	7.4<br>(3.8 to 13)	-2.96%<br>(-3.72 to -2.19)
Vanuatu	0<br>(0 to 0)	0<br>(0 to 0)	58.11%<br>(-65.14 to 675.91)	9.5<br>(2.4 to 25.3)	10.8<br>(3 to 29.1)	-0.23%<br>(-0.61 to 0.15)
Venezuela (Bolivarian Republic of)	1<br>(0 to 1)	0<br>(0 to 0)	-68.57%<br>(-83.85 to -37.17)	19.2<br>(12.2 to 27.7)	5.7<br>(3.3 to 9.1)	-4.28%<br>(-4.98 to -3.58)
Viet Nam	0<br>(0 to 0)	0<br>(0 to 0)	-97.33%<br>(-99.43 to -84.75)	8.9<br>(2.1 to 27)	0.4<br>(0.1 to 1.2)	-10.28%<br>(-10.5 to -10.06)
Yemen	2<br>(1 to 6)	2<br>(0 to 5)	-27.87%<br>(-85.92 to 271.4)	476.9<br>(107 to 1266.5)	258<br>(63.8 to 711.4)	-2.96%<br>(-3.34 to -2.59)
Zambia	0<br>(0 to 1)	0<br>(0 to 1)	-36.97%<br>(-88.41 to 250.78)	17.8<br>(4.5 to 51.3)	7.5<br>(2 to 19.8)	-2.69%<br>(-3.42 to -1.96)
Zimbabwe	1<br>(0 to 2)	1<br>(0 to 3)	42.34%<br>(-67.24 to 636.04)	108.2<br>(28.6 to 274)	140<br>(37.9 to 362.2)	4.45%<br>(2.25 to 6.7)

ASMMR – age-standardized maternal mortality ratio (per 100,000 livebirths), CI – confidence interval, EAPC – estimated annual percentage change, MSMI – maternal sepsis and other maternal infections, UI – uncertain interval.

Table S17. MMR of MSMI in 131 low- and middle-income countries and territories with EAPC in age group of 15-19 years from 1990 and 2019.

Location	Deaths in 1990 (95% UI)	Deaths in 2019 (95% UI)	Relative change (%, 95 CI)	ASMMR in 1990 (95% UI)	ASMMR in 2019 (95% UI)	EAPC (%, 95 CI)
Afghanistan	35 (18 to 62)	35 (17 to 63)	-0.44% (-57.8 to 133)	38.8 (19.4 to 69.1)	20 (9.6 to 36.3)	-2.36% (-3.35 to -1.37)
Albania	0 (0 to 0)	0 (0 to 0)	-88.42% (-95.95 to -68.93)	0.3 (0.1 to 0.5)	0.1 (0 to 0.2)	-3.76% (-4.42 to -3.1)
Algeria	45 (22 to 76)	3 (1 to 5)	-93.27% (-97.17 to -84.02)	122.6 (59.7 to 206.3)	25.3 (12 to 45.7)	-6.3% (-6.72 to -5.87)
American Samoa	0 (0 to 0)	0 (0 to 0)	-48.72% (-82.75 to 49.31)	5.2 (2.3 to 10.1)	3.3 (1.3 to 7.2)	-1.97% (-2.29 to -1.66)
Angola	13 (5 to 24)	14 (6 to 27)	5.92% (-60.53 to 170.63)	14.2 (6 to 26.1)	7.5 (3.3 to 14.6)	-1.9% (-2.2 to -1.6)
Armenia	0 (0 to 0)	0 (0 to 0)	-91.07% (-96.2 to -80.64)	0.5 (0.3 to 0.9)	0.2 (0.1 to 0.4)	-2.37% (-3.49 to -1.24)
Azerbaijan	0 (0 to 0)	0 (0 to 0)	-86.88% (-94.55 to -69.55)	1.3 (0.7 to 2.4)	0.2 (0.1 to 0.3)	-9.06% (-9.94 to -8.18)
Bangladesh	96 (55 to 163)	3 (1 to 5)	-97.05% (-98.76 to -92.71)	12.3 (7.1 to 20.9)	0.6 (0.3 to 1.1)	-10.08% (-11.47 to -8.68)

Location	Deaths in 1990 (95% UI)	Deaths in 2019 (95% UI)	Relative change (%, 95 CI)	ASMMR in 1990 (95% UI)	ASMMR in 2019 (95% UI)	EAPC (%, 95 CI)
Belarus	0<br>(0 to 0)	0<br>(0 to 0)	-91.05%<br>(-95.5 to -82.54)	0.5<br>(0.3 to 0.9)	0.2<br>(0.1 to 0.4)	-4.19%<br>(-4.86 to -3.52)
Belize	0<br>(0 to 0)	0<br>(0 to 0)	55.83%<br>(-21.8 to 227.66)	3.2<br>(1.9 to 4.9)	4.8<br>(2.7 to 7.9)	3.58%<br>(2.96 to 4.21)
Benin	9<br>(5 to 16)	8<br>(4 to 15)	-10.53%<br>(-62.53 to 93.67)	29.6<br>(16 to 51.7)	11.8<br>(5.3 to 21.4)	-2.77%<br>(-3.11 to -2.43)
Bhutan	0<br>(0 to 1)	0<br>(0 to 0)	-94.08%<br>(-97.73 to -84.07)	10.6<br>(4.4 to 19)	1.9<br>(0.9 to 3.9)	-6.17%<br>(-6.56 to -5.78)
Bolivia (Plurinational State of)	11<br>(6 to 18)	3<br>(1 to 6)	-72.46%<br>(-89.05 to -36.24)	30.8<br>(16.7 to 53.3)	8.2<br>(3.6 to 15.4)	-5.2%<br>(-5.42 to -4.97)
Bosnia and Herzegovina	0<br>(0 to 0)	0<br>(0 to 0)	-99.84%<br>(-99.94 to -99.57)	2.6<br>(1.3 to 4.2)	0<br>(0 to 0.1)	-18.71%<br>(-20.36 to -17.01)
Botswana	2<br>(1 to 3)	1<br>(0 to 1)	-67.95%<br>(-87.19 to -18.36)	27<br>(13.1 to 48.8)	12.4<br>(5.5 to 23.7)	-3.21%<br>(-3.94 to -2.46)
Brazil	55<br>(34 to 80)	22<br>(13 to 32)	-60.58%<br>(-78.63 to -25.38)	9.9<br>(6.2 to 14.5)	5.7<br>(3.5 to 8.4)	-0.7%<br>(-1.2 to -0.19)
Bulgaria	0<br>(0 to 0)	0<br>(0 to 0)	-95.19%<br>(-97.73 to -89.57)	1.2<br>(0.7 to 1.9)	0.2<br>(0.1 to 0.3)	-6.94%<br>(-7.78 to -6.1)

Location	Deaths in 1990 (95% UI)	Deaths in 2019 (95% UI)	Relative change (%, 95 CI)	ASMMR in 1990 (95% UI)	ASMMR in 2019 (95% UI)	EAPC (%, 95 CI)
Burkina Faso	20 (10 to 35)	20 (9 to 36)	-0.16% (-58.36 to 131.17)	26.5 (13.7 to 46)	15.3 (6.6 to 27.8)	-2.41% (-2.86 to -1.95)
Burundi	11 (5 to 20)	20 (9 to 34)	86.79% (-24.12 to 331.93)	52 (23.7 to 95.3)	61.4 (27.8 to 103.5)	0.69% (0.18 to 1.21)
Cabo Verde	0 (0 to 0)	0 (0 to 0)	-94.43% (-97.97 to -85.91)	11.7 (5.7 to 20.4)	1.5 (0.7 to 3.1)	-7.85% (-8.34 to -7.36)
Cambodia	11 (5 to 20)	1 (1 to 2)	-87.55% (-95.37 to -66.44)	22.9 (9.8 to 44.2)	4.4 (2.1 to 8.3)	-7.12% (-8.3 to -5.92)
Cameroon	20 (11 to 33)	24 (11 to 44)	17.07% (-49.51 to 158.39)	23.7 (13.2 to 39.5)	18.3 (8.3 to 34.5)	-1.23% (-1.53 to -0.92)
Central African Republic	10 (5 to 17)	21 (10 to 38)	122.83% (1.95 to 425.4)	42.5 (21.1 to 74.4)	56.6 (27.8 to 99.7)	1.02% (0.89 to 1.15)
Chad	32 (19 to 52)	48 (25 to 79)	46.82% (-25.68 to 188.81)	51.2 (29.3 to 82.4)	29.8 (15.8 to 49.6)	-1.85% (-2.22 to -1.47)
China	159 (126 to 205)	1 (1 to 1)	-99.36% (-99.55 to -99.12)	20.7 (16.3 to 26.6)	0.3 (0.2 to 0.3)	-14.7% (-15.64 to -13.75)

Location	Deaths in 1990 (95% UI)	Deaths in 2019 (95% UI)	Relative change (%, 95 CI)	ASMMR in 1990 (95% UI)	ASMMR in 2019 (95% UI)	EAPC (%, 95 CI)
Colombia	12<br>(6 to 19)	4<br>(2 to 7)	-63.9%<br>(-82.83 to -20.96)	7.2<br>(3.9 to 11.6)	3.6<br>(1.9 to 6)	-2.19%<br>(-2.96 to -1.42)
Comoros	0<br>(0 to 1)	0<br>(0 to 0)	-47.62%<br>(-87.91 to 783.78)	6.7<br>(0.4 to 18)	7.7<br>(1.8 to 17.4)	-1.4%<br>(-3.13 to 0.37)
Congo	3<br>(1 to 6)	3<br>(1 to 5)	-23.94%<br>(-68.87 to 123.29)	16.8<br>(6.3 to 31)	13.1<br>(6.1 to 24.2)	-1.12%<br>(-1.71 to -0.52)
Costa Rica	0<br>(0 to 0)	0<br>(0 to 0)	-88.65%<br>(-95.02 to -74.92)	1.8<br>(1.1 to 2.9)	0.3<br>(0.1 to 0.5)	-7.3%<br>(-8.51 to -6.08)
Côte d'Ivoire	14<br>(7 to 24)	13<br>(5 to 23)	-8.1%<br>(-63.7 to 121.27)	13.6<br>(6.6 to 23.8)	9.7<br>(4.3 to 17.8)	-0.93%<br>(-1.38 to -0.47)
Cuba	1<br>(0 to 1)	0<br>(0 to 0)	-83.31%<br>(-91.84 to -64.57)	2.1<br>(1.2 to 3.3)	0.9<br>(0.5 to 1.5)	-3.79%<br>(-4.74 to -2.83)
Democratic People's Republic of Korea	5<br>(2 to 10)	3<br>(1 to 5)	-47.26%<br>(-80.5 to 55.28)	23.8<br>(9 to 46.5)	77.9<br>(34.8 to 147.6)	5.15%<br>(4.16 to 6.14)
Democratic Republic of the Congo	108<br>(49 to 191)	269<br>(144 to 422)	150.32%<br>(23.54 to 434.17)	37.5<br>(17.2 to 66.7)	74.4<br>(39.8 to 116.7)	3.9%<br>(2.91 to 4.9)

Location	Deaths in 1990 (95% UI)	Deaths in 2019 (95% UI)	Relative change (%, 95 CI)	ASMMR in 1990 (95% UI)	ASMMR in 2019 (95% UI)	EAPC (%, 95 CI)
Djibouti	1 (1 to 2)	2 (1 to 3)	16.74% (-56.97 to 171.62)	42 (19.9 to 70.8)	59 (23.8 to 108.9)	1.39% (0.89 to 1.88)
Dominica	0 (0 to 0)	0 (0 to 0)	-36.43% (-73.63 to 51.09)	10.7 (5.4 to 19)	21.7 (10.8 to 38.2)	4.19% (3.43 to 4.94)
Dominican Republic	2 (1 to 4)	1 (0 to 2)	-61.73% (-85.02 to -6.27)	4.1 (2.3 to 6.7)	1.7 (0.8 to 3.3)	-2.01% (-3.11 to -0.9)
Ecuador	7 (4 to 10)	2 (1 to 3)	-73.21% (-88.33 to -40.06)	12.8 (7.4 to 19.9)	2.7 (1.3 to 4.8)	-4.9% (-5.66 to -4.14)
Egypt	40 (21 to 67)	3 (1 to 5)	-93.44% (-97.29 to -84.88)	15.8 (8.5 to 26.9)	1.4 (0.6 to 2.7)	-9.64% (-10.38 to -8.89)
El Salvador	11 (6 to 17)	0 (0 to 1)	-96.93% (-98.73 to -92.98)	29.7 (15.6 to 48.6)	1.6 (0.7 to 3.1)	-9.19% (-10.51 to -7.85)
Equatorial Guinea	2 (1 to 3)	0 (0 to 1)	-85.42% (-94.78 to -65.44)	37.3 (18.1 to 63.1)	3.6 (1.4 to 7.3)	-9.18% (-9.93 to -8.43)
Eritrea	16 (7 to 28)	17 (8 to 31)	5.24% (-53.95 to 171.67)	88.7 (38.6 to 157)	98 (47.5 to 177.7)	1.19% (0.54 to 1.85)
Eswatini	0 (0 to 1)	0 (0 to 0)	-53.85% (-84.59 to 30)	6.7 (3.2 to 12.2)	4.7 (1.9 to 9.8)	-0.73% (-1.74 to 0.29)

Location	Deaths in 1990 (95% UI)	Deaths in 2019 (95% UI)	Relative change (%, 95 CI)	ASMMR in 1990 (95% UI)	ASMMR in 2019 (95% UI)	EAPC (%, 95 CI)
Ethiopia	197<br>(108 to 332)	103<br>(57 to 172)	-47.94%<br>(-75.64 to 4.85)	53.7<br>(29.3 to 90.4)	24.2<br>(13.4 to 40.5)	-2.68%<br>(-3.64 to -1.71)
Fiji	0<br>(0 to 0)	0<br>(0 to 0)	-44.93%<br>(-80.81 to 56.91)	6<br>(2.6 to 12.3)	5.6<br>(2.3 to 11.1)	-0.38%<br>(-0.64 to -0.11)
Gabon	1<br>(0 to 2)	0<br>(0 to 1)	-64.85%<br>(-85.63 to -9.25)	11.3<br>(4.8 to 20.4)	6.2<br>(2.9 to 11.7)	-2.53%<br>(-3.03 to -2.03)
Gambia	2<br>(1 to 3)	2<br>(1 to 4)	22.45%<br>(-45.85 to 165.56)	23.2<br>(11.2 to 40.4)	27.5<br>(12.8 to 47.5)	0.16%<br>(-0.17 to 0.49)
Georgia	0<br>(0 to 0)	0<br>(0 to 0)	-43.43%<br>(-75.26 to 33.16)	0.8<br>(0.4 to 1.3)	1.2<br>(0.6 to 2.1)	3.08%<br>(0.78 to 5.44)
Ghana	11<br>(5 to 21)	5<br>(2 to 9)	-58.2%<br>(-81.88 to 2.02)	14.1<br>(6.7 to 26.9)	6.5<br>(3 to 12)	-2.46%<br>(-2.78 to -2.15)
Global	4773<br>(3619 to 6127)	1713<br>(1355 to 2099)	-64.11%<br>(-73.58 to -51.44)	30.5<br>(23.2 to 39.2)	14.3<br>(11.3 to 17.6)	-2.71%<br>(-3.02 to -2.38)
Grenada	0<br>(0 to 0)	0<br>(0 to 0)	-71.62%<br>(-86.5 to -39.85)	0.7<br>(0.4 to 1.1)	0.5<br>(0.3 to 0.8)	-1.53%<br>(-2.08 to -0.97)
Guatemala	8<br>(5 to 13)	3<br>(2 to 5)	-63.48%<br>(-81.11 to -30.58)	14.7<br>(8.4 to 23.5)	4.7<br>(2.6 to 8)	-5%<br>(-6.08 to -3.91)

Location	Deaths in 1990 (95% UI)	Deaths in 2019 (95% UI)	Relative change (%, 95 CI)	ASMMR in 1990 (95% UI)	ASMMR in 2019 (95% UI)	EAPC (%, 95 CI)
Guinea	20 (11 to 33)	22 (10 to 37)	10.6% (-47.27 to 127.6)	35.2 (19.2 to 58.9)	25.1 (12 to 42.6)	-0.69% (-0.85 to -0.53)
Guinea-Bissau	2 (1 to 4)	1 (0 to 2)	-46.03% (-78.28 to 22.8)	25.6 (12.7 to 44.6)	13.8 (6.2 to 25.7)	-1.84% (-2.09 to -1.6)
Guyana	0 (0 to 0)	0 (0 to 0)	-74.18% (-89.1 to -43.13)	5 (2.7 to 8.4)	3.1 (1.5 to 5.1)	0.07% (-0.81 to 0.97)
Haiti	12 (5 to 21)	16 (8 to 29)	40.18% (-39.19 to 224.52)	42.9 (19.7 to 79)	48.9 (24.7 to 85.5)	1.3% (1.01 to 1.58)
Honduras	15 (9 to 23)	2 (1 to 3)	-88.46% (-95.22 to -74.64)	42.2 (24.2 to 65.7)	3.9 (1.6 to 7.6)	-8.43% (-8.82 to -8.04)
India	2602 (1610 to 3896)	261 (149 to 436)	-89.96% (-94.88 to -80.1)	97.9 (60.5 to 146.5)	30.1 (17.2 to 50.2)	-4.91% (-5.39 to -4.43)
Indonesia	89 (49 to 147)	9 (5 to 15)	-89.56% (-95.17 to -77.97)	11.8 (6.5 to 19.5)	3.6 (2 to 5.8)	-3.92% (-4.06 to -3.79)
Iran (Islamic Republic of)	11 (7 to 16)	1 (0 to 1)	-92.41% (-95.94 to -85.52)	5.9 (3.7 to 8.6)	1.3 (0.7 to 2)	-6.26% (-6.77 to -5.74)
Iraq	1 (0 to 2)	0 (0 to 1)	-63.07% (-86.94 to 7.7)	0.9 (0.4 to 1.7)	0.4 (0.2 to 0.8)	-1.84% (-2.66 to -1.02)

Location	Deaths in 1990 (95% UI)	Deaths in 2019 (95% UI)	Relative change (%, 95 CI)	ASMMR in 1990 (95% UI)	ASMMR in 2019 (95% UI)	EAPC (%, 95 CI)
Jamaica	0<br>(0 to 0)	0<br>(0 to 0)	-90.18%<br>(-95.69 to -78.91)	2.2<br>(1.3 to 3.6)	0.6<br>(0.3 to 1.1)	-4.76%<br>(-6.22 to -3.28)
Jordan	1<br>(0 to 2)	0<br>(0 to 1)	-55.36%<br>(-82.65 to 11.32)	7.7<br>(3.8 to 13.9)	3.4<br>(1.6 to 6.6)	-4.09%<br>(-4.64 to -3.55)
Kazakhstan	0<br>(0 to 0)	0<br>(0 to 0)	-9.01%<br>(-60.1 to 103.14)	0.4<br>(0.2 to 0.6)	0.7<br>(0.3 to 1.1)	0.97%<br>(0.22 to 1.72)
Kenya	31<br>(17 to 48)	24<br>(13 to 39)	-21.39%<br>(-59.17 to 43.87)	17.4<br>(9.9 to 27.2)	14.4<br>(8 to 23.3)	0.08%<br>(-0.33 to 0.5)
Kiribati	0<br>(0 to 0)	0<br>(0 to 0)	15.14%<br>(-56.45 to 201.36)	19.4<br>(9.2 to 35.6)	25.9<br>(11.6 to 50.3)	0.7%<br>(0.42 to 0.98)
Kyrgyzstan	0<br>(0 to 1)	0<br>(0 to 0)	-24.97%<br>(-63.04 to 50.87)	3.5<br>(2 to 5.7)	2.8<br>(1.6 to 4.6)	0.39%<br>(-0.61 to 1.4)
Lao People's Democratic Republic	15<br>(8 to 24)	1<br>(0 to 2)	-93.84%<br>(-97.46 to -85.55)	59.6<br>(32.3 to 98.2)	4<br>(1.8 to 7.6)	-8.85%<br>(-9.37 to -8.33)
Lebanon	0<br>(0 to 1)	0<br>(0 to 0)	-86.66%<br>(-95.68 to -59.5)	1.7<br>(0.7 to 3.4)	0.4<br>(0.2 to 0.8)	-5.23%<br>(-5.84 to -4.62)
Lesotho	4<br>(2 to 7)	2<br>(1 to 3)	-53.36%<br>(-80.52 to 6.64)	37.9<br>(19.2 to 62.3)	29.6<br>(13.4 to 55)	0.51%<br>(-0.15 to 1.18)

Location	Deaths in 1990 (95% UI)	Deaths in 2019 (95% UI)	Relative change (%, 95 CI)	ASMMR in 1990 (95% UI)	ASMMR in 2019 (95% UI)	EAPC (%, 95 CI)
Liberia	8 (5 to 14)	10 (5 to 17)	25.2% (-40.36 to 153.98)	45.4 (25.4 to 75.2)	49.3 (25.7 to 82.8)	-1.09% (-2.01 to -0.17)
Libya	0 (0 to 0)	0 (0 to 0)	-85.06% (-94.8 to -56.7)	0.8 (0.3 to 1.7)	0.4 (0.2 to 0.8)	-2.57% (-3.31 to -1.82)
Madagascar	39 (21 to 66)	37 (18 to 66)	-6.93% (-57.63 to 102.88)	41 (22.3 to 68.9)	23.7 (11.6 to 42.8)	-1.77% (-2.01 to -1.52)
Malawi	14 (7 to 23)	15 (7 to 29)	11.37% (-55.17 to 154.25)	16.3 (8.2 to 27.8)	15.6 (7.1 to 29.8)	0.58% (0.11 to 1.05)
Malaysia	3 (1 to 5)	0 (0 to 1)	-82.52% (-94.33 to -47.88)	10.2 (4.6 to 19.1)	3.1 (1.3 to 6.5)	-4.88% (-5.44 to -4.31)
Maldives	0 (0 to 0)	0 (0 to 0)	-94.76% (-97.88 to -87.45)	4.5 (2.2 to 8.6)	0.6 (0.3 to 1.2)	-6.74% (-7.51 to -5.97)
Mali	54 (31 to 81)	46 (22 to 84)	-14.46% (-59.96 to 82.55)	67.2 (39 to 101.7)	25.9 (12.2 to 47.6)	-3.38% (-3.59 to -3.16)
Marshall Islands	0 (0 to 0)	0 (0 to 0)	-47.44% (-82.55 to 44.34)	5.3 (2.4 to 9.8)	3.9 (1.6 to 8)	-1.23% (-1.65 to -0.8)

Location	Deaths in 1990 (95% UI)	Deaths in 2019 (95% UI)	Relative change (%, 95 CI)	ASMMR in 1990 (95% UI)	ASMMR in 2019 (95% UI)	EAPC (%, 95 CI)
Mauritania	8<br>(4 to 13)	2<br>(1 to 4)	-71.54%<br>(-87.8 to -37.41)	57.9<br>(32.3 to 94.3)	18.4<br>(7.9 to 34.6)	-4.78%<br>(-5.15 to -4.41)
Mexico	15<br>(9 to 24)	4<br>(2 to 6)	-74.52%<br>(-87.37 to -50.22)	3.7<br>(2.3 to 5.8)	1.3<br>(0.8 to 2.1)	-3.04%<br>(-3.31 to -2.76)
Micronesia (Federated States of)	0<br>(0 to 0)	0<br>(0 to 0)	-74.45%<br>(-90.61 to -30.23)	15.7<br>(6.6 to 30)	7.7<br>(3.1 to 15.2)	-2.77%<br>(-2.94 to -2.6)
Mongolia	1<br>(1 to 2)	0<br>(0 to 0)	-88.49%<br>(-95.57 to -69.38)	14.9<br>(7.4 to 26.1)	2.4<br>(1.1 to 4.7)	-7.52%<br>(-8.22 to -6.81)
Montenegro	0<br>(0 to 0)	0<br>(0 to 0)	-77.93%<br>(-93.15 to -27.81)	0<br>(0 to 0)	0<br>(0 to 0)	-2.05%<br>(-3.32 to -0.76)
Morocco	34<br>(17 to 58)	2<br>(1 to 5)	-92.95%<br>(-97 to -83.77)	43.3<br>(21.3 to 73.5)	5.2<br>(2.4 to 10)	-7.45%<br>(-7.74 to -7.16)
Mozambique	39<br>(20 to 65)	21<br>(10 to 39)	-45.17%<br>(-75.77 to 21.56)	31.8<br>(16.4 to 53.2)	9.4<br>(4.4 to 17.3)	-3.78%<br>(-4.24 to -3.32)
Myanmar	53<br>(25 to 94)	10<br>(5 to 20)	-80.7%<br>(-92.4 to -53.18)	35.5<br>(16.7 to 62.7)	11.2<br>(5 to 21.7)	-4.8%<br>(-5.41 to -4.19)
Namibia	2<br>(1 to 3)	1<br>(0 to 1)	-68.19%<br>(-88.29 to -17.65)	25.5<br>(11.8 to 45.6)	8<br>(3.4 to 15.7)	-3.7%<br>(-4.4 to -3)

Location	Deaths in 1990 (95% UI)	Deaths in 2019 (95% UI)	Relative change (%, 95 CI)	ASMMR in 1990 (95% UI)	ASMMR in 2019 (95% UI)	EAPC (%, 95 CI)
Nepal	52<br>(26 to 86)	4<br>(2 to 7)	-92.79%<br>(-97.19 to -83.43)	35.8<br>(18 to 59)	3.1<br>(1.3 to 5.8)	-8.23%<br>(-8.49 to -7.96)
Nicaragua	3<br>(2 to 4)	0<br>(0 to 1)	-86.91%<br>(-94.72 to -68.78)	8.3<br>(4.6 to 13.5)	1.3<br>(0.6 to 2.3)	-7.02%<br>(-7.4 to -6.64)
Niger	40<br>(22 to 67)	66<br>(31 to 113)	63.27%<br>(-24.58 to 265.7)	44<br>(24.4 to 73.6)	29.2<br>(13.7 to 50.4)	-1.67%<br>(-1.85 to -1.49)
Nigeria	91<br>(43 to 162)	92<br>(42 to 179)	0.87%<br>(-56.18 to 105.84)	13.3<br>(6.3 to 23.6)	9.8<br>(4.5 to 19.2)	-1.9%<br>(-2.57 to -1.23)
North Macedonia	0<br>(0 to 0)	0<br>(0 to 0)	-93.26%<br>(-97.44 to -81.64)	0.6<br>(0.3 to 1.1)	0.2<br>(0.1 to 0.3)	-5.72%<br>(-6.86 to -4.57)
Pakistan	151<br>(83 to 247)	88<br>(48 to 145)	-41.57%<br>(-72.5 to 27.26)	17.6<br>(9.6 to 28.9)	8.5<br>(4.7 to 14.1)	-2.58%<br>(-2.82 to -2.33)
Papua New Guinea	8<br>(4 to 15)	7<br>(4 to 13)	-8.06%<br>(-61.26 to 115.07)	55.9<br>(26.3 to 101)	29.7<br>(14.9 to 53.7)	-1.76%<br>(-2.21 to -1.3)
Paraguay	3<br>(2 to 5)	1<br>(0 to 1)	-80.93%<br>(-91.87 to -56.33)	15.8<br>(8.7 to 25.2)	3.5<br>(1.7 to 6.3)	-6.18%<br>(-6.95 to -5.39)
Peru	19<br>(11 to 32)	2<br>(1 to 4)	-87.95%<br>(-94.65 to -74.88)	18.4<br>(10.4 to 30.1)	2.9<br>(1.3 to 5.5)	-6.85%<br>(-7.24 to -6.46)

Location	Deaths in 1990 (95% UI)	Deaths in 2019 (95% UI)	Relative change (%, 95 CI)	ASMMR in 1990 (95% UI)	ASMMR in 2019 (95% UI)	EAPC (%, 95 CI)
Philippines	9<br>(6 to 15)	4<br>(2 to 6)	-62.25%<br>(-80.43 to -25.49)	4.4<br>(2.6 to 6.9)	1.2<br>(0.7 to 1.8)	-3.81%<br>(-4.12 to -3.5)
Republic of Moldova	0<br>(0 to 0)	0<br>(0 to 0)	-97%<br>(-98.63 to -93.66)	2.6<br>(1.5 to 4.3)	0.3<br>(0.2 to 0.5)	-7.42%<br>(-8.2 to -6.63)
Russian Federation	6<br>(4 to 10)	0<br>(0 to 1)	-93.15%<br>(-96.65 to -86.04)	2.3<br>(1.4 to 3.4)	0.6<br>(0.4 to 1)	-6.67%<br>(-7.63 to -5.7)
Rwanda	20<br>(10 to 32)	9<br>(4 to 17)	-54.73%<br>(-81.4 to 6.44)	87.6<br>(46.2 to 145.2)	56.4<br>(25.4 to 110.6)	-0.88%<br>(-2.04 to 0.3)
Saint Lucia	0<br>(0 to 0)	0<br>(0 to 0)	-82.05%<br>(-91.17 to -62.35)	1.7<br>(1 to 2.8)	1.1<br>(0.6 to 1.9)	0.85%<br>(-0.42 to 2.13)
Saint Vincent and the Grenadines	0<br>(0 to 0)	0<br>(0 to 0)	-60.99%<br>(-81.55 to -14.76)	0.3<br>(0.2 to 0.5)	0.3<br>(0.2 to 0.5)	0.48%<br>(-0.15 to 1.12)
Samoa	0<br>(0 to 0)	0<br>(0 to 0)	-77.74%<br>(-92.31 to -39.5)	24.1<br>(10.7 to 46.6)	4.4<br>(1.7 to 9)	-7.34%<br>(-8.13 to -6.56)
Senegal	16<br>(8 to 28)	11<br>(5 to 19)	-32.41%<br>(-71.99 to 50.62)	29.7<br>(15.3 to 53.1)	23.1<br>(10.7 to 42.2)	-0.83%<br>(-1.16 to -0.51)
Serbia	0<br>(0 to 0)	0<br>(0 to 0)	-91.81%<br>(-97.1 to -75.29)	0.6<br>(0.3 to 1.2)	0.2<br>(0.1 to 0.3)	-5.6%<br>(-6.57 to -4.61)

Location	Deaths in 1990 (95% UI)	Deaths in 2019 (95% UI)	Relative change (%, 95 CI)	ASMMR in 1990 (95% UI)	ASMMR in 2019 (95% UI)	EAPC (%, 95 CI)
Sierra Leone	8 (4 to 14)	16 (8 to 27)	96.75% (-11.34 to 299.61)	28.5 (14.2 to 48.4)	35 (18 to 60.1)	0.8% (0.16 to 1.45)
Solomon Islands	0 (0 to 1)	0 (0 to 1)	29.38% (-50.18 to 252.03)	17.6 (7.5 to 36.3)	21.3 (8.8 to 41)	1.06% (0.43 to 1.69)
Somalia	25 (11 to 47)	85 (42 to 154)	239.3% (63.24 to 672.52)	48.6 (21.6 to 91.3)	71.4 (35 to 129.8)	1.74% (1.51 to 1.96)
South Africa	16 (10 to 25)	3 (1 to 6)	-79.72% (-91.46 to -56.18)	9.6 (5.7 to 14.5)	3.1 (1.4 to 5.9)	-1.22% (-2.71 to 0.29)
South Sudan	6 (2 to 12)	7 (3 to 16)	28.74% (-50.08 to 258.33)	14.3 (5.8 to 28.9)	11.9 (4.7 to 25.6)	-0.87% (-1.21 to -0.52)
Sri Lanka	2 (1 to 3)	0 (0 to 0)	-88.93% (-95.78 to -72.09)	6.4 (2.9 to 12.1)	1.7 (0.8 to 3.2)	-5.08% (-5.41 to -4.74)
Sudan	62 (33 to 101)	24 (11 to 45)	-61.96% (-84.73 to -12.1)	32.6 (17.1 to 53)	17.4 (8 to 32.8)	-1.99% (-2.27 to -1.7)
Suriname	0 (0 to 0)	0 (0 to 0)	-25.19% (-71.21 to 143.32)	4.1 (1.5 to 7.8)	3.9 (1.7 to 7.5)	0.8% (-0.63 to 2.26)

Location	Deaths in 1990 (95% UI)	Deaths in 2019 (95% UI)	Relative change (%, 95 CI)	ASMMR in 1990 (95% UI)	ASMMR in 2019 (95% UI)	EAPC (%, 95 CI)
Syrian Arab Republic	3 (2 to 6)	0 (0 to 1)	-90.19% (-95.97 to -73.95)	4.2 (2 to 7.8)	1.1 (0.5 to 2.3)	-6.62% (-7.79 to -5.44)
Tajikistan	0 (0 to 0)	0 (0 to 0)	-66.35% (-86.38 to -16.77)	0.8 (0.4 to 1.4)	0.3 (0.1 to 0.6)	-5.18% (-6.43 to -3.92)
Thailand	2 (1 to 4)	0 (0 to 1)	-78.49% (-91.93 to -40.03)	1.3 (0.5 to 2.6)	0.6 (0.3 to 1.1)	-2.29% (-2.75 to -1.82)
Timor-Leste	1 (0 to 3)	1 (0 to 1)	-53.09% (-83.82 to 58.47)	38 (11.6 to 76)	25.7 (10.5 to 46.5)	-3.11% (-4.19 to -2.02)
Togo	5 (2 to 9)	4 (2 to 7)	-24.55% (-68.71 to 75.55)	22.6 (11.2 to 39.6)	18.8 (8.1 to 35.7)	-0.54% (-0.79 to -0.3)
Tonga	0 (0 to 0)	0 (0 to 0)	-29.4% (-73.27 to 87.7)	10.4 (4.6 to 20.1)	12.6 (5.2 to 25.3)	0.33% (-0.44 to 1.11)
Tunisia	2 (1 to 4)	0 (0 to 0)	-92.37% (-97.16 to -81.36)	11.1 (5.1 to 20.4)	6.7 (2.7 to 13)	-0.79% (-1.56 to -0.02)
Turkey	19 (9 to 37)	1 (0 to 1)	-96.55% (-98.64 to -91.39)	7 (3.2 to 13.3)	0.9 (0.4 to 1.7)	-8.22% (-9.32 to -7.1)
Turkmenistan	0 (0 to 0)	0 (0 to 0)	-37.55% (-68.56 to 34.21)	0.8 (0.4 to 1.4)	0.7 (0.3 to 1.1)	-0.99% (-1.61 to -0.35)
Tuvalu	0 (0 to 0)	0 (0 to 0)	-74.36% (-91.14 to -30.31)	25.9 (11.1 to 48.1)	8.5 (3.4 to 17.6)	-3.53% (-3.64 to -3.42)

Location	Deaths in 1990 (95% UI)	Deaths in 2019 (95% UI)	Relative change (%, 95 CI)	ASMMR in 1990 (95% UI)	ASMMR in 2019 (95% UI)	EAPC (%, 95 CI)
Uganda	23 (8 to 45)	33 (15 to 61)	40.35% (-47.65 to 342.25)	12.3 (4.1 to 23.7)	13.6 (6.3 to 25.1)	0.42% (-0.14 to 0.97)
Ukraine	1 (0 to 1)	0 (0 to 0)	-85.94% (-93.25 to -73.16)	0.9 (0.5 to 1.4)	0.5 (0.2 to 0.9)	-3.58% (-4.84 to -2.3)
United Republic of Tanzania	48 (26 to 82)	37 (18 to 68)	-22.27% (-69.67 to 79.22)	23.4 (12.4 to 39.9)	11 (5.2 to 20)	-2.06% (-2.38 to -1.74)
Uzbekistan	1 (1 to 2)	1 (0 to 1)	-43.39% (-73.27 to 18.68)	1.9 (1.1 to 3)	1.3 (0.7 to 2.2)	-2.33% (-3.41 to -1.24)
Vanuatu	0 (0 to 0)	0 (0 to 0)	63.51% (-40 to 361.32)	6.7 (2.6 to 14.1)	9.5 (3.9 to 18.1)	0.26% (-0.1 to 0.62)
Venezuela (Bolivarian Republic of)	11 (7 to 16)	6 (3 to 9)	-47.86% (-73.11 to -2.24)	11.2 (7.1 to 15.9)	5.5 (3 to 8.9)	-2.88% (-4.13 to -1.61)
Viet Nam	5 (2 to 10)	0 (0 to 0)	-97.17% (-99.02 to -92.13)	3 (1.2 to 6)	0.2 (0.1 to 0.4)	-9.38% (-9.63 to -9.12)
Yemen	18 (4 to 44)	27 (8 to 59)	46.02% (-60.7 to 651.19)	17.9 (3.8 to 43.3)	25.9 (7.7 to 57.3)	0.8% (0.41 to 1.19)
Zambia	13 (7 to 22)	8 (4 to 15)	-35.28% (-71.94 to 44.61)	16.3 (8.6 to 28.5)	8.7 (4.1 to 16.1)	-1.87% (-2.55 to -1.18)

Location	Deaths in 1990 (95% UI)	Deaths in 2019 (95% UI)	Relative change (%, 95 CI)	ASMMR in 1990 (95% UI)	ASMMR in 2019 (95% UI)	EAPC (%, 95 CI)
Zimbabwe	15 (8 to 24)	27 (13 to 50)	83.23% (-16.64 to 298.67)	20.9 (11.9 to 34.7)	36.7 (17.5 to 67.9)	4.99% (3.57 to 6.43)

ASMMR – age-standardized maternal mortality ratio (per 100,000 livebirths), CI – confidence interval, EAPC – estimated annual percentage change, MSMI – maternal sepsis and other maternal infections, UI – uncertain interval.

Table S18. MMR of MSMI in 131 low- and middle-income countries and territories with EAPC in age group of 20-24 years from 1990 and 2019.

Location	Deaths in 1990 (95% UI)	Deaths in 2019 (95% UI)	Relative change (%, 95 CI)	ASMMR in 1990 (95% UI)	ASMMR in 2019 (95% UI)	EAPC (%, 95 CI)
Afghanistan	61 (33 to 103)	68 (34 to 123)	10.77% (-48.21 to 127.45)	36.4 (19.8 to 61.5)	14.4 (7.3 to 26.1)	-3.52% (-4.61 to -2.42)
Albania	0 (0 to 0)	0 (0 to 0)	-83.25% (-94.08 to -55.54)	0.3 (0.1 to 0.5)	0.1 (0.1 to 0.2)	-0.89% (-1.95 to 0.19)
Algeria	73 (37 to 125)	5 (2 to 8)	-93.6% (-97.04 to -86.24)	34.4 (17.7 to 59.2)	4.7 (2.3 to 8.6)	-7.39% (-7.69 to -7.1)
American Samoa	0 (0 to 0)	0 (0 to 0)	-63.26% (-86.48 to -3.22)	3.7 (1.8 to 6.9)	2.4 (1 to 4.7)	-2.02% (-2.32 to -1.71)
Angola	28 (13 to 48)	24 (12 to 43)	-14.76% (-64.66 to 102.37)	21.5 (9.8 to 37.5)	8.6 (4.2 to 15.5)	-2.91% (-3.29 to -2.53)
Armenia	0 (0 to 1)	0 (0 to 0)	-87.5% (-94.34 to -72.67)	1.4 (0.8 to 2.3)	0.5 (0.2 to 0.8)	-4.44% (-5.5 to -3.38)
Azerbaijan	1 (1 to 2)	0 (0 to 0)	-92.06% (-96.73 to -82.17)	1.4 (0.8 to 2.4)	0.2 (0.1 to 0.3)	-10.09% (-11.04 to -9.12)
Bangladesh	119 (66 to 199)	3 (2 to 6)	-97.37% (-98.92 to -93.94)	9.4 (5.3 to 15.7)	0.3 (0.2 to 0.6)	-11.01% (-12.34 to -9.66)

Location	Deaths in 1990 (95% UI)	Deaths in 2019 (95% UI)	Relative change (%, 95 CI)	ASMMR in 1990 (95% UI)	ASMMR in 2019 (95% UI)	EAPC (%, 95 CI)
Belarus	0<br>(0 to 0)	0<br>(0 to 0)	-91.43%<br>(-96.08 to -83.79)	0.5<br>(0.3 to 0.8)	0.1<br>(0.1 to 0.2)	-6.16%<br>(-6.84 to -5.48)
Belize	0<br>(0 to 0)	0<br>(0 to 0)	0.03%<br>(-51.57 to 106.73)	5.2<br>(3 to 8.2)	4.2<br>(2.4 to 6.9)	0.02%<br>(-0.6 to 0.64)
Benin	21<br>(12 to 33)	18<br>(8 to 33)	-12.23%<br>(-60.46 to 78.26)	33.8<br>(19.2 to 53.2)	13.1<br>(6 to 23.5)	-2.82%<br>(-3.1 to -2.53)
Bhutan	1<br>(0 to 1)	0<br>(0 to 0)	-95.21%<br>(-98.25 to -86.65)	7.4<br>(3.4 to 13.4)	0.6<br>(0.2 to 1.1)	-9.22%<br>(-9.58 to -8.87)
Bolivia (Plurinational State of)	23<br>(13 to 36)	6<br>(3 to 12)	-72.15%<br>(-88.5 to -42.23)	33.5<br>(18.8 to 53.5)	7.2<br>(3.5 to 13.1)	-5.69%<br>(-5.9 to -5.48)
Bosnia and Herzegovina	1<br>(0 to 1)	0<br>(0 to 0)	-99.9%<br>(-99.97 to -99.76)	3<br>(1.6 to 5)	0<br>(0 to 0)	-21.99%<br>(-23.64 to -20.31)
Botswana	3<br>(2 to 6)	1<br>(0 to 2)	-71.42%<br>(-88.63 to -33.96)	27.6<br>(14.6 to 48.4)	7.4<br>(3.2 to 14.2)	-4.73%<br>(-5.44 to -4.01)
Brazil	85<br>(54 to 126)	31<br>(19 to 46)	-63.99%<br>(-80.13 to -33.21)	7.5<br>(4.8 to 11.1)	3.9<br>(2.5 to 5.8)	-1.4%<br>(-1.9 to -0.9)
Bulgaria	1<br>(0 to 1)	0<br>(0 to 0)	-95.66%<br>(-98.07 to -90.57)	1.7<br>(1 to 2.7)	0.3<br>(0.2 to 0.6)	-6.82%<br>(-7.82 to -5.82)

Location	Deaths in 1990 (95% UI)	Deaths in 2019 (95% UI)	Relative change (%, 95 CI)	ASMMR in 1990 (95% UI)	ASMMR in 2019 (95% UI)	EAPC (%, 95 CI)
Burkina Faso	40 (22 to 66)	35 (17 to 64)	-12.15% (-61.6 to 82.78)	34 (18.8 to 55.7)	14.2 (6.7 to 25.6)	-3.61% (-4.08 to -3.14)
Burundi	38 (20 to 64)	56 (32 to 88)	45.38% (-25.64 to 185.5)	58.6 (30.9 to 98.2)	47.7 (27 to 75.3)	-0.98% (-1.56 to -0.4)
Cabo Verde	0 (0 to 1)	0 (0 to 0)	-95.05% (-97.93 to -87.67)	10 (4.9 to 16.6)	0.6 (0.3 to 1.1)	-9.93% (-10.27 to -9.59)
Cambodia	39 (20 to 69)	5 (2 to 9)	-87.88% (-95.14 to -72.86)	32.9 (16.7 to 57.9)	4.6 (2.1 to 8.7)	-7.75% (-8.77 to -6.72)
Cameroon	37 (21 to 59)	41 (18 to 77)	10.97% (-51.07 to 132.31)	29.3 (16.5 to 46.2)	16.6 (7.1 to 31)	-1.98% (-2.41 to -1.54)
Central African Republic	19 (10 to 31)	39 (20 to 64)	102.35% (0.31 to 320.26)	53.4 (28.3 to 86.7)	67.5 (35.6 to 111.8)	0.9% (0.75 to 1.05)
Chad	42 (24 to 66)	67 (36 to 110)	58.89% (-17.82 to 192)	49.9 (27.9 to 77.8)	31.9 (17.1 to 52.2)	-1.51% (-1.9 to -1.12)
China	533 (397 to 708)	3 (2 to 4)	-99.47% (-99.65 to -99.18)	5 (3.7 to 6.6)	0.1 (0.1 to 0.1)	-13.21% (-13.75 to -12.67)
Colombia	18 (11 to 30)	5 (3 to 9)	-71.55% (-87.27 to -38.86)	6.1 (3.6 to 9.9)	2.3 (1.2 to 4)	-2.87% (-3.53 to -2.21)

Location	Deaths in 1990 (95% UI)	Deaths in 2019 (95% UI)	Relative change (%, 95 CI)	ASMMR in 1990 (95% UI)	ASMMR in 2019 (95% UI)	EAPC (%, 95 CI)
Comoros	0 (0 to 1)	0 (0 to 0)	-52.47% (-85.54 to 458.1)	6.9 (0.6 to 16.4)	4.1 (1.2 to 8.5)	-3.25% (-4.65 to -1.82)
Congo	6 (3 to 11)	4 (2 to 7)	-37.56% (-72.93 to 79.89)	22.8 (9.6 to 41.1)	11.4 (5.5 to 20.3)	-2.39% (-3.23 to -1.54)
Costa Rica	1 (0 to 1)	0 (0 to 0)	-83.24% (-92.2 to -65.92)	2.1 (1.3 to 3.2)	0.5 (0.3 to 0.9)	-5.73% (-7.07 to -4.37)
Côte d'Ivoire	24 (12 to 41)	22 (9 to 41)	-7.38% (-62.01 to 100.39)	15.2 (8 to 26.2)	9.3 (4 to 17.6)	-1.3% (-1.74 to -0.85)
Cuba	2 (1 to 3)	0 (0 to 1)	-84.03% (-92.44 to -65.91)	3.6 (2.2 to 5.6)	1.2 (0.7 to 2)	-4.14% (-4.67 to -3.62)
Democratic People's Republic of Korea	10 (5 to 21)	7 (3 to 13)	-36.18% (-73.54 to 64.98)	4 (1.7 to 8)	7.7 (3.5 to 14.7)	2.95% (2.12 to 3.8)
Democratic Republic of the Congo	184 (90 to 313)	415 (254 to 655)	125.65% (12.32 to 355.43)	37.1 (18.1 to 63)	54.3 (33.2 to 85.7)	2.79% (1.76 to 3.84)
Djibouti	2 (1 to 4)	3 (1 to 5)	5.11% (-57.6 to 151.03)	40.3 (20 to 67.8)	35 (14 to 65.7)	-0.25% (-0.74 to 0.24)
Dominica	0 (0 to 0)	0 (0 to 0)	-48.95% (-79.23 to 23.65)	14.2 (7.4 to 24.5)	19.1 (9.5 to 34.1)	2.62% (1.66 to 3.6)
Dominican Republic	4 (2 to 6)	2 (1 to 4)	-40.67% (-74.38 to 29.77)	4.3 (2.6 to 7.1)	2.9 (1.4 to 5.1)	-0.26% (-1.3 to 0.79)

Location	Deaths in 1990 (95% UI)	Deaths in 2019 (95% UI)	Relative change (%, 95 CI)	ASMMR in 1990 (95% UI)	ASMMR in 2019 (95% UI)	EAPC (%, 95 CI)
Ecuador	11<br>(7 to 17)	3<br>(2 to 6)	-69.34%<br>(-85.05 to -34.53)	12.1<br>(7.3 to 18.4)	3.6<br>(1.9 to 6.4)	-3.1%<br>(-4.21 to -1.98)
Egypt	65<br>(37 to 103)	4<br>(2 to 9)	-93.38%<br>(-97.3 to -85.33)	10.6<br>(6.1 to 16.9)	0.7<br>(0.3 to 1.4)	-10.02%<br>(-10.44 to -9.6)
El Salvador	16<br>(9 to 24)	1<br>(0 to 1)	-95.83%<br>(-98.24 to -90.93)	29.6<br>(17.5 to 45)	2.1<br>(0.9 to 4)	-8.2%<br>(-9.43 to -6.95)
Equatorial Guinea	4<br>(2 to 6)	0<br>(0 to 1)	-90.28%<br>(-96.41 to -77.52)	67<br>(37.4 to 107.4)	3.7<br>(1.4 to 7.7)	-10.83%<br>(-11.55 to -10.1)
Eritrea	31<br>(15 to 51)	28<br>(15 to 48)	-12.24%<br>(-57.53 to 104.74)	89.5<br>(43.5 to 146.8)	48.9<br>(26.4 to 85.8)	-1.13%<br>(-1.77 to -0.48)
Eswatini	1<br>(0 to 2)	0<br>(0 to 1)	-60.93%<br>(-86.27 to 10.11)	9.5<br>(4.5 to 17.2)	3.7<br>(1.4 to 7.6)	-2.47%<br>(-3.64 to -1.29)
Ethiopia	528<br>(284 to 827)	191<br>(97 to 326)	-63.88%<br>(-82.94 to -28.28)	81.1<br>(43.7 to 127.1)	18<br>(9.2 to 30.8)	-5.37%<br>(-6.24 to -4.49)
Fiji	0<br>(0 to 0)	0<br>(0 to 0)	-59.51%<br>(-84.59 to 4.98)	4<br>(1.9 to 7.3)	2.4<br>(1.1 to 4.5)	-2.55%<br>(-3.07 to -2.03)
Gabon	2<br>(1 to 3)	1<br>(0 to 1)	-70.59%<br>(-87.3 to -30.28)	17.5<br>(8.4 to 29.3)	5<br>(2.2 to 9.2)	-4.66%<br>(-5.35 to -3.96)

Location	Deaths in 1990 (95% UI)	Deaths in 2019 (95% UI)	Relative change (%, 95 CI)	ASMMR in 1990 (95% UI)	ASMMR in 2019 (95% UI)	EAPC (%, 95 CI)
Gambia	4 (2 to 6)	4 (2 to 7)	24.34% (-37.75 to 142.47)	27.6 (14.1 to 46.5)	23.2 (12.1 to 38.8)	-0.83% (-1.21 to -0.44)
Georgia	0 (0 to 1)	0 (0 to 0)	-37.09% (-71.94 to 44.16)	1 (0.5 to 1.6)	1.5 (0.8 to 2.6)	3.54% (1.44 to 5.68)
Ghana	30 (15 to 52)	13 (6 to 23)	-58.24% (-82.01 to -12.35)	19.8 (10 to 34.2)	5.9 (3 to 10.6)	-3.65% (-3.98 to -3.32)
Global	8745 (6784 to 11207)	3026 (2421 to 3637)	-65.4% (-74.39 to -54.7)	19.1 (14.8 to 24.5)	8.5 (6.8 to 10.3)	-3.06% (-3.42 to -2.69)
Grenada	0 (0 to 0)	0 (0 to 0)	-85.07% (-93.13 to -68.3)	1.4 (0.8 to 2.3)	0.5 (0.3 to 0.8)	-2.32% (-3.21 to -1.43)
Guatemala	20 (11 to 31)	6 (3 to 10)	-69.95% (-84.48 to -39.13)	20 (11.4 to 31)	5.1 (2.8 to 8.8)	-5.61% (-6.61 to -4.59)
Guinea	35 (20 to 56)	41 (22 to 70)	16.78% (-40.31 to 122.11)	46.5 (25.9 to 75)	31.7 (17.2 to 54.1)	-0.77% (-0.95 to -0.59)
Guinea-Bissau	4 (2 to 6)	2 (1 to 3)	-45.23% (-73.95 to 10.3)	30.6 (16.8 to 53.7)	12 (5.6 to 21.6)	-2.83% (-3.15 to -2.5)
Guyana	1 (0 to 1)	0 (0 to 0)	-62.53% (-84.05 to -11.72)	6.4 (3.5 to 10.3)	3.9 (2 to 7)	-1.75% (-2.28 to -1.21)
Haiti	36 (17 to 63)	48 (26 to 80)	35.5% (-32.45 to 184.21)	59.6 (28.5 to 104.7)	67.4 (35.9 to 111.5)	1.24% (0.84 to 1.63)

Location	Deaths in 1990 (95% UI)	Deaths in 2019 (95% UI)	Relative change (%, 95 CI)	ASMMR in 1990 (95% UI)	ASMMR in 2019 (95% UI)	EAPC (%, 95 CI)
Honduras	27<br>(16 to 40)	3<br>(1 to 6)	-89.02%<br>(-95.68 to -77.26)	54.4<br>(31.9 to 80.5)	4.2<br>(1.7 to 8.4)	-9.15%<br>(-9.5 to -8.79)
India	4232<br>(2546 to 6448)	486<br>(267 to 836)	-88.52%<br>(-94.19 to -76.8)	45.2<br>(27.2 to 68.9)	6<br>(3.3 to 10.3)	-7.62%<br>(-8.31 to -6.92)
Indonesia	240<br>(136 to 376)	30<br>(16 to 49)	-87.55%<br>(-93.97 to -74.79)	16.3<br>(9.2 to 25.6)	3.3<br>(1.8 to 5.5)	-5.33%<br>(-5.58 to -5.08)
Iran (Islamic Republic of)	23<br>(14 to 35)	1<br>(1 to 2)	-94.32%<br>(-96.95 to -89.03)	3.6<br>(2.2 to 5.5)	0.5<br>(0.3 to 0.7)	-7.16%<br>(-7.56 to -6.77)
Iraq	1<br>(1 to 2)	1<br>(0 to 1)	-56.58%<br>(-83.62 to 21.68)	0.6<br>(0.3 to 1.2)	0.2<br>(0.1 to 0.4)	-3.15%<br>(-3.92 to -2.37)
Jamaica	0<br>(0 to 1)	0<br>(0 to 0)	-80.8%<br>(-91.44 to -57.33)	2.3<br>(1.3 to 3.8)	0.9<br>(0.4 to 1.5)	-5.14%<br>(-6.35 to -3.92)
Jordan	3<br>(1 to 5)	1<br>(0 to 2)	-66.94%<br>(-87.49 to -12.41)	6.2<br>(3.1 to 11.1)	1.8<br>(0.8 to 3.6)	-5.76%<br>(-6.45 to -5.06)
Kazakhstan	0<br>(0 to 1)	0<br>(0 to 1)	16.18%<br>(-46.55 to 165.07)	0.2<br>(0.1 to 0.4)	0.4<br>(0.2 to 0.6)	1.18%<br>(0.04 to 2.33)
Kenya	57<br>(35 to 89)	43<br>(22 to 70)	-24.98%<br>(-60.8 to 44.06)	18.5<br>(11.3 to 28.9)	9.2<br>(4.7 to 15.1)	-2.09%<br>(-2.53 to -1.66)
Kiribati	0<br>(0 to 0)	0<br>(0 to 0)	-11.25%<br>(-67.01 to 111.59)	15.1<br>(7.5 to 26.8)	15.9<br>(7 to 29.2)	0.1%<br>(-0.05 to 0.26)

Location	Deaths in 1990 (95% UI)	Deaths in 2019 (95% UI)	Relative change (%, 95 CI)	ASMMR in 1990 (95% UI)	ASMMR in 2019 (95% UI)	EAPC (%, 95 CI)
Kyrgyzstan	1 (1 to 1)	1 (0 to 1)	-3.31% (-52.93 to 93.47)	2.1 (1.2 to 3.3)	1.9 (1.1 to 2.9)	0.58% (-0.71 to 1.89)
Lao People's Democratic Republic	25 (14 to 42)	2 (1 to 4)	-91.61% (-96.28 to -82.63)	53.1 (29.5 to 89)	4 (2 to 7.1)	-8.55% (-9.18 to -7.93)
Lebanon	1 (0 to 1)	0 (0 to 0)	-87.92% (-95.6 to -70.48)	1.8 (0.9 to 3.4)	0.4 (0.2 to 0.7)	-5.35% (-5.92 to -4.77)
Lesotho	6 (3 to 10)	3 (1 to 6)	-48.24% (-78.54 to 15.09)	33.3 (18.6 to 55.5)	21.6 (9.5 to 39.8)	0.57% (-0.34 to 1.5)
Liberia	9 (5 to 15)	18 (9 to 29)	91.27% (-9.13 to 295.77)	48 (26.2 to 76.8)	45.6 (23.5 to 75.3)	-1.39% (-2.27 to -0.51)
Libya	0 (0 to 1)	0 (0 to 0)	-80.85% (-93.37 to -48.34)	0.7 (0.3 to 1.3)	0.3 (0.1 to 0.6)	-1.67% (-2.84 to -0.49)
Madagascar	63 (38 to 100)	70 (39 to 115)	11.18% (-45.44 to 108.73)	42.6 (25.4 to 67.4)	27.1 (14.9 to 44.6)	-1.57% (-1.91 to -1.24)
Malawi	26 (13 to 44)	22 (10 to 39)	-16.42% (-65.71 to 88.29)	19.7 (10 to 33.7)	13.2 (6.1 to 23.8)	-1.13% (-1.57 to -0.69)
Malaysia	9 (5 to 16)	1 (1 to 2)	-86.29% (-94.63 to -66.16)	8 (4.2 to 14.3)	1.7 (0.8 to 3.1)	-5.45% (-5.77 to -5.13)

Location	Deaths in 1990 (95% UI)	Deaths in 2019 (95% UI)	Relative change (%, 95 CI)	ASMMR in 1990 (95% UI)	ASMMR in 2019 (95% UI)	EAPC (%, 95 CI)
Maldives	0 (0 to 0)	0 (0 to 0)	-95.95% (-98.19 to -91.03)	3.9 (2 to 6.6)	0.2 (0.1 to 0.4)	-9.3% (-10.07 to -8.52)
Mali	87 (54 to 133)	65 (31 to 116)	-24.57% (-63.6 to 41.87)	76.9 (47.5 to 117.7)	25.1 (12.1 to 44.7)	-4.05% (-4.32 to -3.79)
Marshall Islands	0 (0 to 0)	0 (0 to 0)	-45.83% (-78.25 to 30.67)	8.6 (4.2 to 15.7)	5.9 (2.7 to 10.9)	-1.47% (-1.99 to -0.96)
Mauritania	19 (11 to 29)	5 (2 to 9)	-75.08% (-88.46 to -49.29)	86.9 (50.6 to 132.4)	16.9 (8.2 to 31.5)	-6.25% (-6.55 to -5.95)
Mexico	25 (16 to 38)	7 (4 to 11)	-73.43% (-86.83 to -48.08)	3.1 (2 to 4.7)	1.1 (0.7 to 1.8)	-3.01% (-3.25 to -2.77)
Micronesia (Federated States of)	0 (0 to 0)	0 (0 to 0)	-67.32% (-88.44 to -18.05)	16.5 (7.3 to 31.1)	9.6 (3.8 to 18.7)	-2.05% (-2.17 to -1.92)
Mongolia	5 (2 to 8)	1 (0 to 1)	-86.7% (-94.17 to -69.37)	17.3 (9.1 to 29.4)	2.7 (1.2 to 5.1)	-8.07% (-8.77 to -7.37)
Montenegro	0 (0 to 0)	0 (0 to 0)	-72.19% (-90.65 to -20.56)	0 (0 to 0)	0 (0 to 0)	-0.78% (-1.71 to 0.17)
Morocco	51 (27 to 88)	3 (1 to 6)	-94.34% (-97.69 to -86.31)	23.4 (12.2 to 40.3)	2.6 (1.1 to 5.2)	-7.71% (-7.96 to -7.46)

Location	Deaths in 1990 (95% UI)	Deaths in 2019 (95% UI)	Relative change (%, 95 CI)	ASMMR in 1990 (95% UI)	ASMMR in 2019 (95% UI)	EAPC (%, 95 CI)
Mozambique	61<br>(33 to 103)	30<br>(15 to 55)	-50.47%<br>(-77.31 to 10.28)	39.7<br>(21.4 to 66.3)	9.2<br>(4.4 to 16.5)	-4.63%<br>(-5.16 to -4.09)
Myanmar	113<br>(58 to 193)	23<br>(10 to 42)	-79.42%<br>(-91.51 to -54.33)	26.9<br>(14 to 46.2)	8.4<br>(3.8 to 15.2)	-4.47%<br>(-5.04 to -3.89)
Namibia	2<br>(1 to 4)	1<br>(0 to 2)	-68.06%<br>(-87.71 to -17.58)	18<br>(8.4 to 30.6)	4.9<br>(2 to 9.9)	-4.07%<br>(-4.59 to -3.54)
Nepal	109<br>(59 to 178)	7<br>(3 to 13)	-93.45%<br>(-96.87 to -85.6)	42<br>(22.6 to 68.4)	2.9<br>(1.4 to 5.3)	-8.99%<br>(-9.22 to -8.75)
Nicaragua	5<br>(3 to 8)	1<br>(0 to 1)	-89.14%<br>(-95.02 to -76.29)	10.4<br>(5.8 to 16.6)	1.4<br>(0.7 to 2.4)	-7.37%<br>(-7.89 to -6.85)
Niger	57<br>(32 to 91)	105<br>(54 to 173)	82.86%<br>(-10.27 to 257.06)	49.4<br>(27.9 to 78.1)	32<br>(16.5 to 52.6)	-1.74%<br>(-1.97 to -1.5)
Nigeria	162<br>(80 to 284)	157<br>(76 to 292)	-3.12%<br>(-56.73 to 108.22)	14.4<br>(7.1 to 25.1)	8.1<br>(3.9 to 15)	-2.85%<br>(-3.62 to -2.08)
North Macedonia	0<br>(0 to 0)	0<br>(0 to 0)	-95.3%<br>(-98.34 to -86.81)	0.7<br>(0.3 to 1.2)	0.1<br>(0 to 0.2)	-8.06%<br>(-8.83 to -7.27)
Pakistan	288<br>(169 to 447)	150<br>(84 to 254)	-47.92%<br>(-75.54 to 5.15)	20.4<br>(12 to 31.7)	6.8<br>(3.8 to 11.6)	-4.05%<br>(-4.31 to -3.79)
Papua New Guinea	17<br>(9 to 28)	16<br>(8 to 27)	-6.88%<br>(-54.81 to 83.73)	39.2<br>(20.6 to 64.7)	17.3<br>(8.6 to 29.9)	-2.18%<br>(-2.58 to -1.78)

Location	Deaths in 1990 (95% UI)	Deaths in 2019 (95% UI)	Relative change (%, 95 CI)	ASMMR in 1990 (95% UI)	ASMMR in 2019 (95% UI)	EAPC (%, 95 CI)
Paraguay	6 (4 to 9)	1 (0 to 2)	-84.3% (-92.97 to -66.34)	17.3 (10.3 to 26.2)	3.1 (1.6 to 5.6)	-6.38% (-7.04 to -5.71)
Peru	44 (25 to 72)	4 (2 to 7)	-91.06% (-96.07 to -81.73)	20.8 (11.8 to 34.2)	2.3 (1.1 to 4.3)	-8.44% (-8.91 to -7.96)
Philippines	28 (17 to 43)	11 (6 to 17)	-62.55% (-82.41 to -25.54)	4.5 (2.7 to 6.9)	1.4 (0.7 to 2.3)	-3.49% (-3.72 to -3.26)
Republic of Moldova	1 (0 to 1)	0 (0 to 0)	-97.97% (-99.07 to -95.9)	2.1 (1.2 to 3.4)	0.2 (0.1 to 0.3)	-9.65% (-10.62 to -8.67)
Russian Federation	12 (7 to 18)	1 (1 to 2)	-91.56% (-95.93 to -82.56)	1.7 (1 to 2.6)	0.4 (0.2 to 0.6)	-7.5% (-8.42 to -6.56)
Rwanda	63 (37 to 96)	21 (10 to 38)	-66.54% (-85.7 to -31.32)	79.4 (47.1 to 122)	22.9 (10.9 to 41.6)	-4.4% (-5.38 to -3.42)
Saint Lucia	0 (0 to 0)	0 (0 to 0)	-72.74% (-88.27 to -41.87)	1.4 (0.8 to 2.2)	1.1 (0.6 to 1.9)	1.25% (-0.03 to 2.55)
Saint Vincent and the Grenadines	0 (0 to 0)	0 (0 to 0)	-52.65% (-78.4 to -3.13)	0.4 (0.2 to 0.6)	0.4 (0.2 to 0.7)	1.52% (0.3 to 2.76)
Samoa	0 (0 to 0)	0 (0 to 0)	-76.72% (-93.14 to -33.69)	8.7 (4 to 16.6)	2.2 (0.8 to 4.8)	-6.06% (-6.75 to -5.36)

Location	Deaths in 1990 (95% UI)	Deaths in 2019 (95% UI)	Relative change (%, 95 CI)	ASMMR in 1990 (95% UI)	ASMMR in 2019 (95% UI)	EAPC (%, 95 CI)
Senegal	31<br>(17 to 50)	22<br>(11 to 40)	-27.13%<br>(-66.37 to 45.13)	35.1<br>(19.8 to 56.9)	19.3<br>(9.7 to 34.1)	-1.81%<br>(-2.11 to -1.51)
Serbia	0<br>(0 to 0)	0<br>(0 to 0)	-93.4%<br>(-97.8 to -81.33)	0.5<br>(0.2 to 1)	0.1<br>(0 to 0.2)	-6.61%<br>(-7.41 to -5.8)
Sierra Leone	12<br>(6 to 20)	25<br>(13 to 42)	109.36%<br>(-0.48 to 344.32)	28<br>(14.2 to 47.5)	31.7<br>(16.4 to 53.6)	0.55%<br>(-0.1 to 1.21)
Solomon Islands	1<br>(1 to 3)	2<br>(1 to 3)	14.52%<br>(-53.35 to 182.09)	31.7<br>(15.1 to 60.6)	26.2<br>(11.4 to 48.1)	-0.15%<br>(-0.7 to 0.41)
Somalia	45<br>(21 to 77)	164<br>(92 to 273)	265.65%<br>(99.56 to 634.99)	64.3<br>(30.6 to 110.7)	70.6<br>(39.6 to 117.5)	0.58%<br>(0.36 to 0.81)
South Africa	49<br>(30 to 73)	9<br>(4 to 16)	-82.09%<br>(-93.18 to -59.83)	17.6<br>(10.6 to 26.1)	3.8<br>(1.6 to 7)	-4.34%<br>(-6.39 to -2.25)
South Sudan	12<br>(5 to 23)	12<br>(5 to 26)	3.43%<br>(-55.45 to 162.56)	15.8<br>(6.9 to 29.7)	11.8<br>(5.1 to 24.9)	-1.18%<br>(-1.45 to -0.9)
Sri Lanka	3<br>(2 to 6)	0<br>(0 to 1)	-90.26%<br>(-96.11 to -75.97)	3.4<br>(1.7 to 6)	0.7<br>(0.3 to 1.3)	-5.88%<br>(-6.15 to -5.6)
Sudan	127<br>(72 to 206)	35<br>(15 to 66)	-72.14%<br>(-88.93 to -35.49)	45.1<br>(25.6 to 73.1)	12.7<br>(5.5 to 23.7)	-4.28%<br>(-4.66 to -3.89)
Suriname	0<br>(0 to 0)	0<br>(0 to 0)	-44.17%<br>(-77.13 to 39.51)	3.4<br>(1.5 to 6.2)	2.7<br>(1.3 to 4.9)	-1.06%<br>(-1.89 to -0.22)

Location	Deaths in 1990 (95% UI)	Deaths in 2019 (95% UI)	Relative change (%, 95 CI)	ASMMR in 1990 (95% UI)	ASMMR in 2019 (95% UI)	EAPC (%, 95 CI)
Syrian Arab Republic	6 (3 to 11)	0 (0 to 1)	-93.46% (-97.5 to -83.08)	4.3 (2 to 7.7)	0.7 (0.3 to 1.3)	-8.52% (-9.8 to -7.23)
Tajikistan	0 (0 to 1)	0 (0 to 0)	-70.05% (-88.37 to -33.85)	0.5 (0.3 to 0.9)	0.1 (0 to 0.2)	-6.37% (-7.14 to -5.6)
Thailand	4 (2 to 9)	1 (0 to 2)	-75.87% (-90.78 to -31.84)	1.2 (0.5 to 2.4)	0.7 (0.3 to 1.4)	-2.11% (-2.71 to -1.49)
Timor-Leste	4 (2 to 7)	1 (0 to 2)	-65.26% (-93.38 to -7.11)	37.6 (15.6 to 69.6)	12.3 (2.1 to 23.4)	-5.23% (-6.02 to -4.44)
Togo	11 (6 to 19)	8 (4 to 15)	-25.01% (-68.47 to 63.07)	26.1 (13.5 to 44.9)	14.3 (6.6 to 25.8)	-1.8% (-2.19 to -1.41)
Tonga	0 (0 to 0)	0 (0 to 0)	-55.07% (-82.5 to 10.37)	5 (2.3 to 9.1)	3.5 (1.5 to 7.3)	-1.27% (-1.43 to -1.12)
Tunisia	4 (2 to 7)	0 (0 to 1)	-92.12% (-96.91 to -80.94)	5.5 (2.8 to 9.9)	1.7 (0.7 to 3.4)	-3.51% (-3.88 to -3.15)
Turkey	49 (25 to 82)	2 (1 to 4)	-95.61% (-98.24 to -89.63)	8.1 (4.1 to 13.6)	1 (0.5 to 1.8)	-8.72% (-9.85 to -7.58)
Turkmenistan	0 (0 to 0)	0 (0 to 0)	-50.31% (-77.92 to 9.54)	0.5 (0.3 to 0.8)	0.3 (0.1 to 0.5)	-2.47% (-3.09 to -1.85)
Tuvalu	0 (0 to 0)	0 (0 to 0)	-81.9% (-93.54 to -52.23)	19.3 (9 to 34.5)	5 (2 to 10.1)	-4.17% (-4.4 to -3.93)

Location	Deaths in 1990 (95% UI)	Deaths in 2019 (95% UI)	Relative change (%, 95 CI)	ASMMR in 1990 (95% UI)	ASMMR in 2019 (95% UI)	EAPC (%, 95 CI)
Uganda	36 (14 to 67)	48 (22 to 87)	33.02% (-47.77 to 259.84)	13 (4.9 to 24.2)	9.7 (4.5 to 17.5)	-1.38% (-1.97 to -0.78)
Ukraine	2 (1 to 4)	0 (0 to 1)	-83.32% (-92.01 to -67.43)	1 (0.5 to 1.6)	0.5 (0.2 to 0.8)	-4.02% (-4.91 to -3.11)
United Republic of Tanzania	79 (44 to 133)	62 (29 to 104)	-22.25% (-66.96 to 70.27)	22.5 (12.4 to 37.8)	10.4 (4.9 to 17.6)	-1.99% (-2.34 to -1.64)
Uzbekistan	5 (3 to 7)	4 (2 to 6)	-22.17% (-62.47 to 59.67)	1.8 (1.1 to 2.8)	1.2 (0.7 to 2.2)	-1.18% (-2.36 to 0.02)
Vanuatu	0 (0 to 0)	0 (0 to 0)	25.75% (-56.04 to 227.18)	8.1 (3.5 to 15.7)	7.2 (2.9 to 14)	-1.23% (-1.54 to -0.91)
Venezuela (Bolivarian Republic of)	18 (11 to 25)	9 (5 to 15)	-49.86% (-74.67 to 0.26)	10.6 (6.6 to 15.1)	7.4 (4 to 12.5)	-1.86% (-3.24 to -0.46)
Viet Nam	13 (5 to 26)	0 (0 to 1)	-97.72% (-99.15 to -93.65)	2.1 (0.8 to 4)	0.1 (0 to 0.2)	-10.89% (-11.17 to -10.61)
Yemen	47 (15 to 93)	56 (20 to 105)	21.32% (-54.74 to 323.84)	27 (8.5 to 54.2)	20.7 (7.5 to 38.4)	-1.65% (-1.96 to -1.33)
Zambia	23 (13 to 40)	13 (6 to 24)	-45.53% (-76.07 to 20.54)	20.7 (11.2 to 35)	7.2 (3.3 to 13.4)	-4.07% (-4.72 to -3.42)

Location	Deaths in 1990 (95% UI)	Deaths in 2019 (95% UI)	Relative change (%, 95 CI)	ASMMR in 1990 (95% UI)	ASMMR in 2019 (95% UI)	EAPC (%, 95 CI)
Zimbabwe	25 (15 to 39)	44 (23 to 74)	76.24% (-6.89 to 257.33)	21.6 (13 to 34.1)	33.3 (17.6 to 55.7)	5% (3.41 to 6.62)

ASMMR – age-standardized maternal mortality ratio (per 100,000 livebirths), CI – confidence interval, EAPC – estimated annual percentage change, MSMI – maternal sepsis and other maternal infections, UI – uncertain interval.

Table S19. MMR of MSMI in 131 low- and middle-income countries and territories with EAPC in age group of 25-29 years from 1990 and 2019.

Location	Deaths in 1990 (95% UI)	Deaths in 2019 (95% UI)	Relative change (%, 95 CI)	ASMMR in 1990 (95% UI)	ASMMR in 2019 (95% UI)	EAPC (%, 95 CI)
Afghanistan	50<br>(26 to 86)	72<br>(35 to 129)	45.95%<br>(-29.67 to 213.48)	41.5<br>(21.4 to 71.5)	16.1<br>(7.7 to 28.8)	-3.76%<br>(-4.86 to -2.65)
Albania	0<br>(0 to 0)	0<br>(0 to 0)	-82.37%<br>(-93.35 to -55.16)	0.4<br>(0.2 to 0.7)	0.2<br>(0.1 to 0.3)	-1.27%<br>(-2.36 to -0.16)
Algeria	74<br>(39 to 132)	6<br>(3 to 11)	-92.37%<br>(-96.81 to -83.72)	38.2<br>(20 to 67.5)	2.4<br>(1.1 to 4.4)	-9.67%<br>(-9.88 to -9.47)
American Samoa	0<br>(0 to 0)	0<br>(0 to 0)	-71.69%<br>(-90.18 to -15.16)	2.6<br>(1.1 to 4.8)	1.4<br>(0.6 to 2.7)	-2.8%<br>(-3.16 to -2.45)
Angola	33<br>(16 to 62)	25<br>(12 to 43)	-24.55%<br>(-68.88 to 68.46)	29.2<br>(13.6 to 54.2)	9.9<br>(4.8 to 16.9)	-3.57%<br>(-3.89 to -3.26)
Armenia	0<br>(0 to 1)	0<br>(0 to 0)	-82.9%<br>(-92.22 to -63.14)	1.5<br>(0.9 to 2.5)	0.4<br>(0.2 to 0.8)	-5.74%<br>(-7.39 to -4.05)
Azerbaijan	2<br>(1 to 3)	0<br>(0 to 0)	-93.59%<br>(-97.34 to -85.11)	2.6<br>(1.4 to 4.3)	0.2<br>(0.1 to 0.5)	-11.16%<br>(-12.41 to -9.89)
Bangladesh	85<br>(48 to 142)	3<br>(1 to 5)	-97.05%<br>(-98.77 to -92.81)	9.3<br>(5.2 to 15.4)	0.3<br>(0.2 to 0.6)	-10.61%<br>(-12.12 to -9.07)

Location	Deaths in 1990 (95% UI)	Deaths in 2019 (95% UI)	Relative change (%, 95 CI)	ASMMR in 1990 (95% UI)	ASMMR in 2019 (95% UI)	EAPC (%, 95 CI)
Belarus	0 (0 to 0)	0 (0 to 0)	-88.42% (-94.93 to -75.17)	0.8 (0.4 to 1.2)	0.1 (0 to 0.2)	-8.65% (-9.32 to -7.97)
Belize	0 (0 to 0)	0 (0 to 0)	79.37% (-11.17 to 265.89)	4.3 (2.5 to 6.6)	5.9 (3.3 to 9.4)	1.51% (0.94 to 2.08)
Benin	27 (15 to 44)	22 (11 to 41)	-18.18% (-62.47 to 71.53)	44.2 (24.7 to 71.5)	17 (8.4 to 31.3)	-2.83% (-3.09 to -2.57)
Bhutan	1 (0 to 1)	0 (0 to 0)	-94.38% (-97.9 to -86.01)	8.6 (4 to 15)	0.8 (0.3 to 1.5)	-8.01% (-8.25 to -7.77)
Bolivia (Plurinational State of)	30 (16 to 49)	8 (3 to 14)	-73.9% (-89.55 to -46.28)	49.6 (27.4 to 81.2)	9.6 (4 to 17.5)	-5.92% (-6.13 to -5.71)
Bosnia and Herzegovina	1 (0 to 1)	0 (0 to 0)	-99.88% (-99.96 to -99.7)	3.8 (2.1 to 6.3)	0 (0 to 0)	-22.55% (-24.35 to -20.7)
Botswana	3 (1 to 5)	1 (0 to 2)	-69.56% (-88.14 to -23.15)	32.4 (15 to 57.1)	7.4 (3 to 15.2)	-5.32% (-6.39 to -4.24)
Brazil	83 (53 to 126)	31 (19 to 47)	-62.66% (-79.92 to -30.63)	10.6 (6.7 to 16)	4.1 (2.5 to 6.2)	-2.32% (-2.79 to -1.86)
Bulgaria	1 (0 to 1)	0 (0 to 0)	-92.67% (-97.01 to -84.07)	2.9 (1.7 to 4.7)	0.3 (0.1 to 0.5)	-10.37% (-11.45 to -9.27)

Location	Deaths in 1990 (95% UI)	Deaths in 2019 (95% UI)	Relative change (%, 95 CI)	ASMMR in 1990 (95% UI)	ASMMR in 2019 (95% UI)	EAPC (%, 95 CI)
Burkina Faso	44 (23 to 73)	37 (18 to 66)	-14.98% (-60.57 to 78.74)	41.7 (22.2 to 69.7)	16.6 (7.9 to 29.7)	-3.67% (-4.08 to -3.25)
Burundi	53 (28 to 91)	59 (34 to 95)	11.96% (-41.43 to 133.32)	76 (40 to 130.1)	50.2 (29 to 79.9)	-1.84% (-2.31 to -1.36)
Cabo Verde	0 (0 to 1)	0 (0 to 0)	-93.93% (-97.47 to -86.2)	10.9 (5.9 to 18.3)	0.7 (0.3 to 1.4)	-9.35% (-9.76 to -8.95)
Cambodia	60 (29 to 110)	8 (4 to 15)	-86.72% (-94.74 to -69.23)	49.8 (24.5 to 91.6)	7.1 (3.3 to 13.5)	-7.74% (-8.98 to -6.48)
Cameroon	40 (23 to 63)	44 (20 to 79)	11% (-49.39 to 135.09)	35.7 (20.4 to 56.7)	20.1 (9.2 to 35.8)	-2.05% (-2.37 to -1.72)
Central African Republic	26 (14 to 43)	47 (25 to 80)	78.09% (-14.02 to 256.48)	89.7 (48.5 to 145.9)	109.7 (58 to 188.1)	0.75% (0.6 to 0.9)
Chad	50 (29 to 77)	71 (38 to 112)	40.43% (-26.16 to 171.22)	69.4 (39.9 to 106.9)	39.6 (21.1 to 62.7)	-1.9% (-2.34 to -1.46)
China	459 (339 to 603)	4 (3 to 5)	-99.15% (-99.44 to -98.72)	5.2 (3.9 to 6.9)	0.1 (0.1 to 0.1)	-14.84% (-15.73 to -13.94)
Colombia	14 (8 to 23)	4 (2 to 7)	-71.65% (-87.65 to -37.49)	6 (3.3 to 10)	1.9 (0.9 to 3.3)	-3.62% (-4.18 to -3.05)
Comoros	1 (0 to 2)	1 (0 to 1)	-39.08% (-78.43 to 299.68)	24.2 (4.1 to 48)	18.1 (6.8 to 35.1)	-1.53% (-2.24 to -0.82)

Location	Deaths in 1990 (95% UI)	Deaths in 2019 (95% UI)	Relative change (%, 95 CI)	ASMMR in 1990 (95% UI)	ASMMR in 2019 (95% UI)	EAPC (%, 95 CI)
Congo	7 (3 to 13)	5 (2 to 9)	-29.93% (-72.13 to 81.21)	33.1 (13.6 to 58.8)	16.9 (7.5 to 30.6)	-2.47% (-3.23 to -1.7)
Costa Rica	1 (1 to 1)	0 (0 to 0)	-82.63% (-92.02 to -62.67)	3.8 (2.4 to 5.7)	0.8 (0.4 to 1.4)	-5.76% (-7.09 to -4.42)
Côte d'Ivoire	31 (16 to 51)	29 (14 to 54)	-5.27% (-59.78 to 100.7)	22.1 (11.7 to 36.7)	13.8 (6.4 to 25.3)	-1.21% (-1.69 to -0.73)
Cuba	3 (2 to 5)	1 (0 to 1)	-83.18% (-91.86 to -66.22)	6.5 (3.9 to 10)	1.9 (1 to 3.1)	-4.76% (-5.52 to -3.98)
Democratic People's Republic of Korea	10 (4 to 20)	8 (3 to 15)	-24.79% (-70.7 to 90.51)	3.5 (1.5 to 6.9)	4.8 (2 to 9.2)	1.46% (0.32 to 2.62)
Democratic Republic of the Congo	212 (113 to 360)	480 (276 to 739)	126.31% (15.85 to 335.01)	48.3 (25.8 to 82.1)	68.8 (39.6 to 106.1)	2.61% (1.6 to 3.62)
Djibouti	2 (1 to 4)	3 (1 to 6)	31.23% (-45.72 to 193.29)	46.1 (22.8 to 75.7)	42.1 (17.7 to 77.6)	-0.05% (-0.34 to 0.24)
Dominica	0 (0 to 0)	0 (0 to 0)	-45.45% (-76.66 to 23)	15.1 (7.8 to 24.9)	15.2 (7.7 to 27.1)	0.37% (0.01 to 0.73)
Dominican Republic	3 (2 to 6)	2 (1 to 4)	-31.53% (-71.91 to 56.28)	5.7 (3.2 to 9.2)	3.8 (1.7 to 6.7)	-0.64% (-1.21 to -0.06)

Location	Deaths in 1990 (95% UI)	Deaths in 2019 (95% UI)	Relative change (%, 95 CI)	ASMMR in 1990 (95% UI)	ASMMR in 2019 (95% UI)	EAPC (%, 95 CI)
Ecuador	9<br>(5 to 14)	3<br>(1 to 5)	-67.53%<br>(-85.42 to -32.48)	12.7<br>(7.4 to 19.6)	3.5<br>(1.8 to 6.1)	-3.66%<br>(-4.72 to -2.6)
Egypt	74<br>(41 to 116)	5<br>(2 to 10)	-93.1%<br>(-97.09 to -84.17)	13.7<br>(7.7 to 21.7)	0.8<br>(0.3 to 1.5)	-10.21%<br>(-10.66 to -9.76)
El Salvador	13<br>(7 to 20)	1<br>(0 to 1)	-95.25%<br>(-98.08 to -89.34)	33<br>(18.5 to 50.5)	2.1<br>(0.9 to 4.2)	-8.73%<br>(-9.89 to -7.57)
Equatorial Guinea	4<br>(2 to 7)	0<br>(0 to 1)	-91.1%<br>(-96.91 to -77.61)	88.9<br>(48.4 to 143)	4.7<br>(1.8 to 9.8)	-11%<br>(-11.88 to -10.1)
Eritrea	41<br>(22 to 66)	32<br>(16 to 56)	-21.89%<br>(-61.51 to 60.87)	128.1<br>(70 to 207.6)	74.3<br>(38.5 to 130.6)	-0.91%<br>(-1.66 to -0.15)
Eswatini	1<br>(0 to 2)	0<br>(0 to 1)	-55.37%<br>(-85.41 to 18.54)	10.5<br>(5.1 to 20)	5.3<br>(1.7 to 11.8)	-1.19%<br>(-2.35 to -0.01)
Ethiopia	726<br>(420 to 1147)	208<br>(109 to 339)	-71.33%<br>(-86.58 to -44.23)	123.5<br>(71.3 to 194.9)	24.1<br>(12.6 to 39.3)	-5.82%<br>(-6.6 to -5.03)
Fiji	0<br>(0 to 0)	0<br>(0 to 0)	-58.36%<br>(-85.14 to 31.74)	3.4<br>(1.5 to 6.7)	1.4<br>(0.6 to 2.9)	-3.89%<br>(-4.28 to -3.49)
Gabon	2<br>(1 to 4)	1<br>(0 to 1)	-68.41%<br>(-86.38 to -22.41)	22.7<br>(11.1 to 39.3)	6.4<br>(2.8 to 12.2)	-4.35%<br>(-5.13 to -3.57)
Gambia	4<br>(2 to 7)	5<br>(3 to 8)	15.09%<br>(-42.92 to 129.77)	33.2<br>(17.2 to 55.5)	28.6<br>(15.4 to 48)	-0.77%<br>(-1.04 to -0.49)

Location	Deaths in 1990 (95% UI)	Deaths in 2019 (95% UI)	Relative change (%, 95 CI)	ASMMR in 1990 (95% UI)	ASMMR in 2019 (95% UI)	EAPC (%, 95 CI)
Georgia	0<br>(0 to 1)	0<br>(0 to 0)	-45.69%<br>(-77.31 to 22.01)	2.1<br>(1.1 to 3.7)	1.9<br>(1 to 3.2)	1.46%<br>(-0.6 to 3.57)
Ghana	36<br>(19 to 61)	15<br>(7 to 28)	-57.73%<br>(-81.69 to -9.81)	23.3<br>(12 to 39.6)	6.9<br>(3.2 to 12.7)	-3.48%<br>(-3.77 to -3.19)
Global	8127<br>(6573 to 9874)	3231<br>(2643 to 3880)	-60.25%<br>(-69.21 to -48.98)	20.3<br>(16.4 to 24.7)	8.1<br>(6.6 to 9.8)	-3.26%<br>(-3.78 to -2.74)
Grenada	0<br>(0 to 0)	0<br>(0 to 0)	-84.58%<br>(-92.61 to -67.36)	3.4<br>(2 to 5.6)	0.5<br>(0.3 to 0.9)	-4.04%<br>(-5.06 to -3.01)
Guatemala	17<br>(10 to 28)	5<br>(3 to 9)	-70.83%<br>(-85.63 to -43.49)	21.2<br>(11.9 to 34.7)	4.7<br>(2.4 to 8)	-5.95%<br>(-6.93 to -4.97)
Guinea	44<br>(24 to 71)	46<br>(24 to 78)	4.8%<br>(-45.8 to 103.13)	61.5<br>(34.3 to 99.6)	41.1<br>(21.4 to 70.5)	-0.79%<br>(-0.98 to -0.6)
Guinea-Bissau	4<br>(2 to 7)	2<br>(1 to 4)	-42.94%<br>(-74.81 to 25.62)	38<br>(19.6 to 65.3)	16.2<br>(7.5 to 29.4)	-2.49%<br>(-2.73 to -2.26)
Guyana	1<br>(0 to 1)	0<br>(0 to 0)	-61.46%<br>(-82.93 to -17.14)	10.7<br>(5.8 to 17.7)	5.7<br>(2.9 to 10.2)	-2.74%<br>(-3.33 to -2.14)
Haiti	48<br>(24 to 83)	67<br>(36 to 108)	40.22%<br>(-30.75 to 199.64)	74.5<br>(37.3 to 129.3)	81.3<br>(44.1 to 131.7)	0.96%<br>(0.55 to 1.37)
Honduras	27<br>(16 to 42)	3<br>(1 to 7)	-88.35%<br>(-95.33 to -72.84)	65.7<br>(39.2 to 99.6)	5.8<br>(2.4 to 11.9)	-8.38%<br>(-8.72 to -8.04)

Location	Deaths in 1990 (95% UI)	Deaths in 2019 (95% UI)	Relative change (%, 95 CI)	ASMMR in 1990 (95% UI)	ASMMR in 2019 (95% UI)	EAPC (%, 95 CI)
India	3092 (1820 to 4656)	434 (230 to 731)	-85.95% (-92.96 to -71.71)	40.1 (23.6 to 60.3)	5.1 (2.7 to 8.5)	-7.37% (-8.23 to -6.5)
Indonesia	297 (163 to 483)	38 (21 to 64)	-87.24% (-93.82 to -74.58)	24.1 (13.3 to 39.3)	3.2 (1.8 to 5.5)	-6.53% (-6.78 to -6.28)
Iran (Islamic Republic of)	33 (21 to 50)	2 (1 to 4)	-92.71% (-96.04 to -86.51)	7.7 (4.9 to 11.5)	0.6 (0.4 to 0.9)	-8.88% (-9.28 to -8.48)
Iraq	1 (1 to 3)	1 (0 to 1)	-51.49% (-82.53 to 39.34)	0.8 (0.4 to 1.6)	0.3 (0.1 to 0.5)	-3.35% (-4.44 to -2.25)
Jamaica	0 (0 to 1)	0 (0 to 0)	-77.61% (-90.58 to -47.16)	3.4 (1.9 to 5.5)	1 (0.5 to 1.9)	-7.15% (-8.52 to -5.77)
Jordan	3 (2 to 6)	1 (0 to 2)	-75.53% (-90.23 to -38.73)	7.4 (3.8 to 12.7)	0.9 (0.4 to 1.8)	-8.97% (-9.78 to -8.16)
Kazakhstan	0 (0 to 1)	1 (0 to 1)	70.52% (-19.92 to 272.01)	0.3 (0.2 to 0.5)	0.5 (0.3 to 0.7)	0.82% (-0.25 to 1.9)
Kenya	57 (33 to 92)	48 (25 to 81)	-17.23% (-59.49 to 57.52)	22.9 (13.1 to 36.7)	15.1 (8 to 25.7)	-1.22% (-1.7 to -0.73)
Kiribati	0 (0 to 0)	0 (0 to 0)	-19.69% (-70.42 to 113.36)	15.4 (7 to 30.6)	9.6 (4.2 to 18)	-1.97% (-2.22 to -1.71)
Kyrgyzstan	1 (0 to 1)	1 (1 to 1)	25.53% (-40.95 to 156.23)	1.8 (1.1 to 2.9)	1.9 (1.1 to 2.9)	0.8% (-0.34 to 1.96)

Location	Deaths in 1990 (95% UI)	Deaths in 2019 (95% UI)	Relative change (%, 95 CI)	ASMMR in 1990 (95% UI)	ASMMR in 2019 (95% UI)	EAPC (%, 95 CI)
Lao People's Democratic Republic	30 (17 to 48)	2 (1 to 4)	-91.82% (-96.49 to -83.25)	68.3 (40.1 to 110.5)	5 (2.4 to 9.2)	-8.49% (-9.26 to -7.72)
Lebanon	1 (0 to 1)	0 (0 to 0)	-85.8% (-94.75 to -62.97)	2.3 (1 to 4.4)	0.3 (0.1 to 0.6)	-7.23% (-7.55 to -6.91)
Lesotho	7 (3 to 11)	4 (2 to 8)	-35.82% (-74.02 to 40.55)	43 (21.7 to 69.6)	37.8 (16.7 to 73.8)	2.08% (1.01 to 3.15)
Liberia	13 (7 to 21)	21 (11 to 35)	57.93% (-21.36 to 202.14)	68.4 (38.2 to 108.8)	76.8 (41 to 126.7)	-0.87% (-1.75 to 0)
Libya	0 (0 to 1)	0 (0 to 0)	-75.38% (-91.92 to -34.62)	1.2 (0.5 to 2.3)	0.5 (0.2 to 1)	-2.63% (-3.43 to -1.82)
Madagascar	58 (33 to 94)	67 (34 to 114)	14.26% (-42.34 to 128.79)	46.8 (26.2 to 75.4)	34.1 (17.7 to 58.3)	-1.04% (-1.34 to -0.75)
Malawi	29 (15 to 47)	20 (9 to 35)	-29.81% (-69.03 to 54.17)	26.5 (14.1 to 43.7)	17.7 (8.2 to 31.3)	-1.2% (-1.5 to -0.9)
Malaysia	13 (7 to 22)	2 (1 to 4)	-83.34% (-93.34 to -59.21)	7.8 (4.1 to 13.5)	1.2 (0.6 to 2.3)	-6.24% (-6.68 to -5.79)
Maldives	0 (0 to 0)	0 (0 to 0)	-94.16% (-97.46 to -87.01)	7 (3.6 to 12.3)	0.4 (0.2 to 0.7)	-10.39% (-10.99 to -9.79)

Location	Deaths in 1990 (95% UI)	Deaths in 2019 (95% UI)	Relative change (%, 95 CI)	ASMMR in 1990 (95% UI)	ASMMR in 2019 (95% UI)	EAPC (%, 95 CI)
Mali	110<br>(69 to 166)	71<br>(38 to 125)	-35.23%<br>(-66.87 to 20.89)	107.5<br>(67.3 to 162.2)	32.5<br>(17.5 to 57)	-4.31%<br>(-4.6 to -4.02)
Marshall Islands	0<br>(0 to 0)	0<br>(0 to 0)	-38.34%<br>(-81.49 to 51.36)	9.3<br>(4.4 to 17.5)	7.6<br>(2.7 to 15.1)	-0.8%<br>(-1.59 to -0.01)
Mauritania	24<br>(14 to 35)	6<br>(3 to 10)	-76.31%<br>(-88.82 to -54.02)	110.4<br>(66.6 to 165.8)	23.8<br>(11.3 to 42.6)	-5.88%<br>(-6.18 to -5.57)
Mexico	22<br>(14 to 35)	7<br>(4 to 11)	-70.62%<br>(-86.15 to -38.92)	3.6<br>(2.2 to 5.7)	1.2<br>(0.7 to 2)	-3.45%<br>(-3.71 to -3.19)
Micronesia (Federated States of)	0<br>(0 to 0)	0<br>(0 to 0)	-74.05%<br>(-92.89 to -29.43)	16.6<br>(7.3 to 31.6)	7.6<br>(2.2 to 16.7)	-3.03%<br>(-3.3 to -2.75)
Mongolia	6<br>(3 to 10)	1<br>(0 to 2)	-83.6%<br>(-93.37 to -61.04)	24.3<br>(12.3 to 41.9)	4.1<br>(1.8 to 7.9)	-8.25%<br>(-9.19 to -7.3)
Montenegro	0<br>(0 to 0)	0<br>(0 to 0)	-67.87%<br>(-89.8 to -2.5)	0<br>(0 to 0.1)	0<br>(0 to 0)	-3.02%<br>(-4.19 to -1.84)
Morocco	63<br>(34 to 104)	4<br>(2 to 7)	-94.13%<br>(-97.64 to -86.94)	32.4<br>(17.5 to 53.5)	2.5<br>(1.1 to 5.1)	-8.64%<br>(-8.96 to -8.31)
Mozambique	48<br>(25 to 82)	25<br>(11 to 47)	-48.17%<br>(-78.14 to 15.31)	39.8<br>(21 to 67.9)	10<br>(4.5 to 18.8)	-4.31%<br>(-4.84 to -3.78)

Location	Deaths in 1990 (95% UI)	Deaths in 2019 (95% UI)	Relative change (%, 95 CI)	ASMMR in 1990 (95% UI)	ASMMR in 2019 (95% UI)	EAPC (%, 95 CI)
Myanmar	113 (58 to 198)	24 (12 to 44)	-79.18% (-90.83 to -50.77)	32.1 (16.5 to 56.5)	8.4 (4.2 to 15.7)	-5.35% (-6.06 to -4.64)
Namibia	3 (1 to 5)	1 (0 to 2)	-66.21% (-87.94 to -5.37)	22.8 (9.5 to 41.2)	4.7 (1.8 to 10)	-4.89% (-5.62 to -4.16)
Nepal	104 (55 to 170)	7 (3 to 13)	-93.19% (-97.12 to -85.44)	51.1 (26.9 to 83.7)	4.8 (2.3 to 8.7)	-7.55% (-7.91 to -7.19)
Nicaragua	4 (2 to 5)	0 (0 to 1)	-89.85% (-95.89 to -77.16)	10.7 (6.1 to 16.8)	1.1 (0.6 to 2.1)	-8.59% (-8.95 to -8.24)
Niger	71 (39 to 112)	109 (59 to 181)	52.99% (-21.05 to 185.17)	71.7 (39.4 to 112.9)	42.6 (22.9 to 70.9)	-2.05% (-2.3 to -1.8)
Nigeria	182 (90 to 334)	178 (82 to 338)	-1.95% (-58.33 to 113.71)	17.3 (8.6 to 31.9)	9.2 (4.2 to 17.5)	-3.05% (-3.81 to -2.3)
North Macedonia	0 (0 to 0)	0 (0 to 0)	-95.17% (-98.17 to -87.45)	0.8 (0.4 to 1.5)	0.1 (0 to 0.1)	-10.11% (-10.98 to -9.23)
Pakistan	309 (179 to 480)	150 (78 to 251)	-51.43% (-77.63 to -0.57)	26.7 (15.5 to 41.4)	8.3 (4.3 to 13.8)	-4.4% (-4.73 to -4.06)
Papua New Guinea	17 (9 to 29)	16 (8 to 29)	-2.95% (-53.32 to 100.19)	42.8 (23.6 to 72.1)	18.9 (9.6 to 32.9)	-2.06% (-2.51 to -1.62)
Paraguay	6 (4 to 10)	1 (0 to 2)	-82.61% (-92.75 to -61.42)	19.9 (11.6 to 31.5)	3.3 (1.5 to 6.1)	-7.15% (-7.85 to -6.45)

Location	Deaths in 1990 (95% UI)	Deaths in 2019 (95% UI)	Relative change (%, 95 CI)	ASMMR in 1990 (95% UI)	ASMMR in 2019 (95% UI)	EAPC (%, 95 CI)
Peru	36 (20 to 57)	4 (2 to 8)	-88.86% (-95.18 to -74.11)	22.5 (12.5 to 35.9)	2.4 (1.1 to 4.7)	-8.51% (-9.01 to -8.01)
Philippines	34 (20 to 53)	12 (6 to 21)	-64.54% (-84.12 to -23.57)	6 (3.5 to 9.2)	1.6 (0.9 to 2.9)	-4.22% (-4.44 to -3.99)
Republic of Moldova	1 (0 to 1)	0 (0 to 0)	-97.36% (-98.79 to -94.45)	2.7 (1.6 to 4.2)	0.2 (0.1 to 0.3)	-10.99% (-11.98 to -9.98)
Russian Federation	11 (7 to 16)	1 (1 to 3)	-85.86% (-93.35 to -72.51)	2.1 (1.3 to 3.3)	0.3 (0.2 to 0.5)	-8.8% (-9.74 to -7.86)
Rwanda	86 (51 to 135)	23 (11 to 43)	-72.98% (-87.99 to -45.99)	100 (59.9 to 157.4)	28.5 (13.7 to 53.2)	-4.61% (-5.36 to -3.85)
Saint Lucia	0 (0 to 0)	0 (0 to 0)	-71.35% (-86.35 to -38.54)	3.1 (1.8 to 5)	1.7 (0.9 to 2.9)	-0.92% (-2.17 to 0.34)
Saint Vincent and the Grenadines	0 (0 to 0)	0 (0 to 0)	-45.59% (-73.86 to 20.64)	0.7 (0.4 to 1.2)	0.6 (0.3 to 0.9)	-1.17% (-1.96 to -0.36)
Samoa	0 (0 to 0)	0 (0 to 0)	-80.74% (-95.99 to -41.65)	7.4 (3.2 to 14.3)	1.4 (0.3 to 3.1)	-7.05% (-7.78 to -6.31)
Senegal	33 (19 to 54)	24 (12 to 42)	-27.93% (-67.09 to 48.54)	40.6 (22.6 to 65.9)	21 (10.6 to 36.3)	-2.16% (-2.54 to -1.78)
Serbia	0 (0 to 1)	0 (0 to 0)	-89.88% (-96.41 to -72.77)	0.7 (0.3 to 1.3)	0.1 (0.1 to 0.2)	-7.38% (-8.22 to -6.52)

Location	Deaths in 1990 (95% UI)	Deaths in 2019 (95% UI)	Relative change (%, 95 CI)	ASMMR in 1990 (95% UI)	ASMMR in 2019 (95% UI)	EAPC (%, 95 CI)
Sierra Leone	15 (8 to 25)	28 (15 to 47)	90.39% (-11.92 to 318.52)	38.5 (20.4 to 64.6)	44.2 (23.1 to 74.7)	0.68% (0.03 to 1.34)
Solomon Islands	1 (1 to 3)	2 (1 to 3)	23.52% (-52.31 to 202.26)	36.1 (15.1 to 69.3)	30.3 (12.9 to 58.5)	-0.05% (-0.65 to 0.55)
Somalia	66 (33 to 113)	183 (96 to 319)	177.57% (38.18 to 465.08)	93.2 (47.1 to 160.2)	88.5 (46.7 to 154.6)	-0.04% (-0.24 to 0.15)
South Africa	69 (40 to 108)	18 (6 to 35)	-73.94% (-91.91 to -36.45)	30 (17.6 to 46.8)	5.4 (1.8 to 10.7)	-5.68% (-8.07 to -3.23)
South Sudan	14 (6 to 27)	11 (4 to 20)	-25.19% (-70.74 to 90.31)	24.1 (10.1 to 45.9)	15.6 (6.5 to 29.7)	-1.66% (-1.91 to -1.41)
Sri Lanka	3 (1 to 5)	0 (0 to 1)	-89.2% (-95.62 to -75.06)	2.6 (1.3 to 4.5)	0.3 (0.1 to 0.6)	-7.2% (-7.48 to -6.92)
Sudan	143 (83 to 228)	35 (13 to 67)	-75.46% (-90.95 to -45.09)	58.4 (33.8 to 92.8)	11.9 (4.5 to 22.8)	-5.15% (-5.44 to -4.86)
Suriname	0 (0 to 0)	0 (0 to 0)	-19.11% (-70.76 to 129.66)	4 (1.7 to 7.3)	3.6 (1.7 to 6.7)	-0.38% (-1.19 to 0.44)
Syrian Arab Republic	7 (3 to 12)	0 (0 to 1)	-95.94% (-98.52 to -89.55)	4.9 (2.4 to 9)	0.5 (0.2 to 1.1)	-9.4% (-10.52 to -8.27)
Tajikistan	0 (0 to 1)	0 (0 to 0)	-68.66% (-87.8 to -21.31)	0.7 (0.4 to 1.3)	0.2 (0.1 to 0.4)	-5.69% (-6.55 to -4.81)

Location	Deaths in 1990 (95% UI)	Deaths in 2019 (95% UI)	Relative change (%, 95 CI)	ASMMR in 1990 (95% UI)	ASMMR in 2019 (95% UI)	EAPC (%, 95 CI)
Thailand	5<br>(2 to 10)	2<br>(1 to 3)	-69.04%<br>(-89.43 to -8.21)	1.7<br>(0.7 to 3.4)	0.9<br>(0.4 to 1.8)	-2.78%<br>(-4.25 to -1.3)
Timor-Leste	5<br>(2 to 8)	1<br>(0 to 2)	-74.51%<br>(-97.4 to -36.04)	45.1<br>(21.2 to 78.2)	10.6<br>(1.1 to 20.4)	-6.06%<br>(-6.66 to -5.46)
Togo	16<br>(9 to 26)	12<br>(6 to 22)	-23.23%<br>(-64.74 to 57.74)	39<br>(21.5 to 63.7)	21.1<br>(10.1 to 38)	-1.94%<br>(-2.27 to -1.6)
Tonga	0<br>(0 to 0)	0<br>(0 to 0)	-60.39%<br>(-86.11 to 4.55)	4.6<br>(2.2 to 8.6)	1.9<br>(0.8 to 3.8)	-3.04%<br>(-3.24 to -2.85)
Tunisia	5<br>(2 to 8)	0<br>(0 to 1)	-91.52%<br>(-96.85 to -78.85)	7.3<br>(3.7 to 13)	0.8<br>(0.3 to 1.8)	-6.36%<br>(-6.89 to -5.82)
Turkey	55<br>(29 to 95)	3<br>(1 to 5)	-94.85%<br>(-97.83 to -87.83)	11.7<br>(6.1 to 20.3)	0.9<br>(0.4 to 1.7)	-10.01%<br>(-11.01 to -9.01)
Turkmenistan	0<br>(0 to 0)	0<br>(0 to 0)	-52.12%<br>(-78.31 to 6.27)	0.5<br>(0.3 to 0.9)	0.3<br>(0.2 to 0.6)	-2.48%<br>(-3.19 to -1.77)
Tuvalu	0<br>(0 to 0)	0<br>(0 to 0)	-85.44%<br>(-94.13 to -63.85)	21<br>(9.3 to 40.3)	3.8<br>(1.5 to 7.7)	-5.57%<br>(-5.79 to -5.34)
Uganda	42<br>(17 to 76)	47<br>(24 to 87)	11.19%<br>(-48.53 to 209.25)	19.3<br>(7.6 to 34.9)	11.6<br>(5.9 to 21.6)	-2.02%<br>(-2.59 to -1.45)
Ukraine	2<br>(1 to 3)	1<br>(0 to 1)	-66.8%<br>(-84.55 to -30.46)	1<br>(0.5 to 1.6)	0.4<br>(0.2 to 0.8)	-4.5%<br>(-5.52 to -3.48)

Location	Deaths in 1990 (95% UI)	Deaths in 2019 (95% UI)	Relative change (%, 95 CI)	ASMMR in 1990 (95% UI)	ASMMR in 2019 (95% UI)	EAPC (%, 95 CI)
United Republic of Tanzania	82 (45 to 133)	58 (28 to 101)	-28.71% (-68.51 to 58.11)	27.4 (15 to 44.8)	12 (5.8 to 20.9)	-2.16% (-2.48 to -1.84)
Uzbekistan	5 (3 to 8)	4 (2 to 6)	-27.87% (-65.81 to 53.91)	2.4 (1.4 to 3.7)	2 (1 to 3.3)	-0.31% (-1.68 to 1.08)
Vanuatu	0 (0 to 0)	0 (0 to 0)	9.61% (-71.35 to 190)	8.7 (3.7 to 16.5)	7.8 (2.2 to 15.4)	-0.91% (-1.2 to -0.62)
Venezuela (Bolivarian Republic of)	19 (12 to 28)	9 (5 to 16)	-53.98% (-78.34 to -11.15)	13.8 (8.9 to 20)	7.8 (4 to 14.1)	-2.06% (-3.26 to -0.85)
Viet Nam	15 (7 to 31)	0 (0 to 1)	-97.22% (-98.96 to -92.84)	2.8 (1.2 to 5.7)	0.1 (0 to 0.2)	-12.05% (-12.37 to -11.73)
Yemen	68 (31 to 123)	72 (33 to 129)	5.8% (-58.83 to 144.98)	42 (19.1 to 75.8)	32.3 (14.6 to 57.4)	-1.79% (-2.16 to -1.41)
Zambia	24 (13 to 38)	12 (6 to 22)	-49.39% (-77.64 to 1.17)	27.3 (15.1 to 43.7)	8.4 (3.8 to 15)	-4.79% (-5.4 to -4.17)
Zimbabwe	21 (12 to 34)	43 (22 to 76)	108.84% (-0.81 to 320.77)	23.5 (13.6 to 37.8)	41.8 (21.5 to 72.7)	5.52% (3.87 to 7.19)

ASMMR – age-standardized maternal mortality ratio (per 100,000 livebirths), CI – confidence interval, EAPC – estimated annual percentage change, MSMI – maternal sepsis and other maternal infections, UI – uncertain interval.

Table S20. MMR of MSMI in 131 low- and middle-income countries and territories with EAPC in age group of 30-34 years from 1990 and 2019.

Location	Deaths in 1990 (95% UI)	Deaths in 2019 (95% UI)	Relative change (%, 95 CI)	ASMMR in 1990 (95% UI)	ASMMR in 2019 (95% UI)	EAPC (%, 95 CI)
Afghanistan	29 (15 to 51)	47 (22 to 82)	62.11% (-25.16 to 241.98)	46.1 (24.2 to 80.8)	20.7 (9.5 to 36.1)	-3.25% (-4.31 to -2.17)
Albania	0 (0 to 0)	0 (0 to 0)	-81.66% (-93.45 to - 53.63)	0.8 (0.4 to 1.3)	0.3 (0.1 to 0.6)	-0.36% (-1.94 to 1.24)
Algeria	60 (33 to 100)	7 (3 to 12)	-89.14% (-95.21 to - 75.69)	40.1 (22.2 to 66.4)	2.4 (1.2 to 4.6)	-9.6% (-9.91 to -9.29)
American Samoa	0 (0 to 0)	0 (0 to 0)	-73.09% (-91.2 to -21.29)	5.6 (2.6 to 10.5)	2.5 (1.1 to 4.8)	-3.35% (-3.63 to -3.07)
Angola	26 (12 to 47)	22 (11 to 41)	-14.83% (-64.96 to 105.43)	29.7 (13.5 to 52.7)	10.9 (5.2 to 19.9)	-3.28% (-3.57 to -2.98)
Armenia	0 (0 to 1)	0 (0 to 0)	-82.12% (-91.79 to -57.7)	3.3 (1.8 to 5.7)	0.9 (0.4 to 1.5)	-5.92% (-7.42 to -4.41)
Azerbaijan	1 (1 to 2)	0 (0 to 0)	-92.09% (-96.51 to - 83.34)	5.2 (2.8 to 8.6)	0.6 (0.3 to 1.1)	-10.03% (-11.21 to -8.83)

Location	Deaths in 1990 (95% UI)	Deaths in 2019 (95% UI)	Relative change (%, 95 CI)	ASMMR in 1990 (95% UI)	ASMMR in 2019 (95% UI)	EAPC (%, 95 CI)
Bangladesh	60 (31 to 97)	2 (1 to 4)	-96.65% (-98.64 to -92.16)	12.2 (6.4 to 20)	0.6 (0.3 to 1)	-10.2% (-11.52 to -8.86)
Belarus	0 (0 to 1)	0 (0 to 0)	-87.49% (-94.23 to -73.19)	2.1 (1.2 to 3.4)	0.2 (0.1 to 0.3)	-9.61% (-10.34 to -8.88)
Belize	0 (0 to 0)	0 (0 to 0)	134.61% (7.62 to 399.34)	6.4 (3.7 to 10)	9.3 (5.1 to 15)	0.9% (0.18 to 1.62)
Benin	27 (15 to 43)	23 (11 to 42)	-14.5% (-59.32 to 78.12)	60.4 (33.3 to 97.3)	26.2 (13 to 47.7)	-2.47% (-2.72 to -2.21)
Bhutan	0 (0 to 1)	0 (0 to 0)	-92.63% (-97.36 to -81.97)	12.4 (5.5 to 23.1)	1.2 (0.5 to 2.3)	-8.06% (-8.32 to -7.8)
Bolivia (Plurinational State of)	26 (15 to 43)	8 (3 to 15)	-67.96% (-87.34 to -29.97)	60.8 (34.2 to 100.3)	12.7 (5.3 to 23.3)	-5.78% (-6.05 to -5.52)
Bosnia and Herzegovina	1 (0 to 1)	0 (0 to 0)	-99.78% (-99.92 to -99.47)	5.5 (3 to 9)	0 (0 to 0)	-22.97% (-24.56 to -21.34)

Location	Deaths in 1990 (95% UI)	Deaths in 2019 (95% UI)	Relative change (%, 95 CI)	ASMMR in 1990 (95% UI)	ASMMR in 2019 (95% UI)	EAPC (%, 95 CI)
Botswana	2 (1 to 4)	1 (0 to 1)	-66.31% (-87.21 to -13.95)	35.8 (16.6 to 66.2)	7.6 (3.1 to 14.8)	-5.51% (-6.51 to -4.5)
Brazil	86 (54 to 126)	29 (17 to 44)	-66.73% (-82.63 to -32.94)	13.7 (8.6 to 20.1)	3.7 (2.2 to 5.8)	-3.55% (-4.16 to -2.95)
Bulgaria	0 (0 to 0)	0 (0 to 0)	-84.24% (-93.09 to -63.37)	3.4 (1.9 to 5.4)	0.3 (0.1 to 0.5)	-12.63% (-13.98 to -11.26)
Burkina Faso	37 (20 to 63)	34 (17 to 60)	-7% (-58.37 to 97.35)	47.2 (25.6 to 79.9)	20.4 (10.1 to 35.9)	-3.54% (-3.94 to -3.14)
Burundi	52 (27 to 88)	65 (36 to 100)	24.1% (-37.73 to 159.49)	92.5 (48.1 to 157.3)	66.1 (36.8 to 102.5)	-1.56% (-2.03 to -1.09)
Cabo Verde	0 (0 to 1)	0 (0 to 0)	-92.99% (-97.14 to -84.12)	17.3 (9.3 to 29.4)	0.9 (0.4 to 1.7)	-10.32% (-10.75 to -9.89)
Cambodia	47 (23 to 84)	8 (4 to 16)	-82.13% (-92.46 to -59.05)	54.4 (26.2 to 97.4)	11 (5.2 to 20.7)	-6.58% (-7.76 to -5.38)

Location	Deaths in 1990 (95% UI)	Deaths in 2019 (95% UI)	Relative change (%, 95 CI)	ASMMR in 1990 (95% UI)	ASMMR in 2019 (95% UI)	EAPC (%, 95 CI)
Cameroon	36 (20 to 59)	43 (20 to 77)	21.08% (-45.48 to 151.05)	47.1 (26.5 to 77.7)	23.5 (10.7 to 41.9)	-2.36% (-2.76 to -1.95)
Central African Republic	20 (10 to 33)	39 (19 to 68)	97.46% (-3.23 to 308)	93.2 (48.8 to 152.6)	123.3 (60.9 to 212.7)	1.09% (0.93 to 1.25)
Chad	45 (26 to 74)	65 (34 to 108)	44.05% (-26.97 to 182.7)	85.9 (48.9 to 140.5)	50.6 (26.7 to 84.4)	-1.8% (-2.18 to -1.41)
China	323 (250 to 415)	5 (3 to 6)	-98.55% (-99.02 to -97.8)	11.2 (8.7 to 14.4)	0.1 (0.1 to 0.1)	-15.56% (-16.43 to -14.68)
Colombia	14 (8 to 23)	5 (2 to 8)	-66.19% (-85.65 to -23.27)	9.8 (5.5 to 15.9)	3.3 (1.7 to 5.7)	-3.41% (-3.99 to -2.83)
Comoros	1 (0 to 2)	1 (0 to 2)	-23.09% (-70.08 to 319.93)	35.8 (6.4 to 68.2)	25.2 (10.5 to 48.7)	-1.85% (-2.47 to -1.21)
Congo	6 (3 to 11)	5 (2 to 9)	-15.39% (-66.32 to 113.4)	37.6 (16.7 to 69.1)	16.1 (7.5 to 30.1)	-2.94% (-3.8 to -2.07)

Location	Deaths in 1990 (95% UI)	Deaths in 2019 (95% UI)	Relative change (%, 95 CI)	ASMMR in 1990 (95% UI)	ASMMR in 2019 (95% UI)	EAPC (%, 95 CI)
Costa Rica	1 (1 to 2)	0 (0 to 0)	-84.22% (-92.37 to -68.89)	8.6 (5.3 to 12.8)	1.3 (0.7 to 2.2)	-6.73% (-8 to -5.44)
Cuba	3 (2 to 5)	1 (0 to 1)	-76.66% (-88.53 to -53.32)	15.7 (9.5 to 24)	3.6 (2 to 5.8)	-4.7% (-5.33 to -4.07)
Democratic People's Republic of Korea	9 (4 to 17)	8 (3 to 15)	-16.83% (-68.76 to 124.08)	6.6 (3 to 12.1)	9.7 (3.7 to 18.8)	1.42% (0.27 to 2.59)
Democratic Republic of the Congo	168 (85 to 282)	419 (229 to 668)	149.93% (22.05 to 424.76)	48.9 (24.9 to 82.3)	69.8 (38.3 to 111.4)	2.77% (1.67 to 3.88)
Djibouti	2 (1 to 3)	4 (2 to 7)	89.85% (-20.03 to 328.78)	51.3 (26.5 to 87.6)	39.2 (16 to 74.4)	-0.67% (-1.09 to -0.26)
Dominica	0 (0 to 0)	0 (0 to 0)	-30.86% (-71.11 to 57.01)	17.9 (9.6 to 28.7)	18.2 (9.3 to 31.8)	0.24% (-0.14 to 0.63)
Dominican Republic	3 (2 to 5)	2 (1 to 4)	-23.25% (-70.61 to 84.78)	9.5 (5.4 to 16.7)	6.9 (3.2 to 13.9)	-0.22% (-0.86 to 0.41)

Location	Deaths in 1990 (95% UI)	Deaths in 2019 (95% UI)	Relative change (%, 95 CI)	ASMMR in 1990 (95% UI)	ASMMR in 2019 (95% UI)	EAPC (%, 95 CI)
Ecuador	9 (5 to 13)	3 (1 to 6)	-64.64% (-84.13 to -24.8)	17.7 (10.7 to 27)	5 (2.4 to 9.1)	-3.34% (-4.36 to -2.32)
Egypt	55 (31 to 92)	5 (2 to 9)	-91.49% (-96.18 to -81.82)	16.8 (9.5 to 27.9)	1.1 (0.5 to 2.2)	-10.04% (-10.56 to -9.53)
El Salvador	11 (7 to 18)	1 (0 to 1)	-94.72% (-97.86 to -87.93)	54.6 (31.7 to 87.2)	3.4 (1.5 to 6.8)	-8.95% (-10.18 to -7.71)
Equatorial Guinea	4 (2 to 6)	0 (0 to 1)	-91.15% (-97.12 to -77.96)	95.8 (49.3 to 157)	4.1 (1.5 to 8.6)	-11.54% (-12.26 to -10.82)
Eritrea	39 (21 to 66)	31 (16 to 53)	-21.55% (-61.87 to 52.4)	156.3 (83.9 to 263.6)	76.2 (40 to 131.2)	-1.45% (-2.11 to -0.78)
Eswatini	1 (0 to 1)	0 (0 to 1)	-50.11% (-82.68 to 37.61)	17.1 (7.9 to 31.1)	7 (2.6 to 14.7)	-1.85% (-3.07 to -0.62)
Ethiopia	671 (379 to 1094)	207 (110 to 328)	-69.18% (-84.97 to -40.78)	155 (87.5 to 252.7)	30.6 (16.3 to 48.6)	-5.8% (-6.56 to -5.03)

Location	Deaths in 1990 (95% UI)	Deaths in 2019 (95% UI)	Relative change (%, 95 CI)	ASMMR in 1990 (95% UI)	ASMMR in 2019 (95% UI)	EAPC (%, 95 CI)
Fiji	0 (0 to 0)	0 (0 to 0)	-53.34% (-83.54 to 31.78)	6.4 (2.9 to 11.8)	2.5 (1.1 to 4.9)	-3.49% (-3.67 to -3.3)
Gabon	1 (1 to 3)	0 (0 to 1)	-67.53% (-86.58 to -17.29)	22.6 (10.6 to 41.3)	5.3 (2.5 to 10.5)	-5.39% (-6.12 to -4.65)
Gambia	4 (2 to 6)	5 (3 to 9)	33.61% (-38.02 to 193.66)	42 (21.8 to 69.1)	34.7 (17.7 to 61.5)	-0.79% (-1.14 to -0.44)
Georgia	0 (0 to 1)	0 (0 to 0)	-29.5% (-70.4 to 64.91)	3.5 (1.8 to 5.6)	3.1 (1.6 to 5.3)	1.26% (-0.82 to 3.38)
Ghana	33 (17 to 57)	15 (7 to 28)	-54.52% (-79.27 to 1.77)	30.3 (15.4 to 51.9)	8.1 (3.9 to 15.2)	-3.98% (-4.27 to -3.7)
Global	6393 (5265 to 7912)	2935 (2415 to 3555)	-54.1% (-64.43 to -42.02)	29.2 (24.1 to 36.1)	10.1 (8.3 to 12.2)	-3.55% (-3.97 to -3.14)
Grenada	0 (0 to 0)	0 (0 to 0)	-79.22% (-90.49 to -56.26)	3.2 (1.8 to 5.1)	0.6 (0.3 to 1.1)	-4.73% (-5.6 to -3.85)

Location	Deaths in 1990 (95% UI)	Deaths in 2019 (95% UI)	Relative change (%, 95 CI)	ASMMR in 1990 (95% UI)	ASMMR in 2019 (95% UI)	EAPC (%, 95 CI)
Guatemala	13 (7 to 21)	4 (2 to 7)	-65% (-82.31 to -30.94)	23.3 (13.3 to 38.2)	6.5 (3.3 to 10.9)	-5.71% (-6.72 to -4.69)
Guinea	40 (23 to 64)	44 (24 to 75)	10.55% (-41.87 to 111.95)	72.8 (42.2 to 116.3)	53.9 (29.1 to 91.9)	-0.39% (-0.59 to -0.19)
Guinea-Bissau	4 (2 to 6)	2 (1 to 4)	-37.31% (-73.19 to 31.12)	44.7 (23.2 to 74.9)	17.8 (8.7 to 32)	-2.69% (-3.02 to -2.36)
Guyana	1 (0 to 1)	0 (0 to 0)	-75.2% (-89.12 to -39.61)	24.4 (13 to 41.8)	7.4 (3.6 to 13.9)	-3.66% (-4.16 to -3.16)
Haiti	47 (23 to 83)	77 (39 to 130)	62.95% (-22.21 to 244.92)	97.2 (46.9 to 172.6)	102.2 (51.5 to 173.9)	1.03% (0.66 to 1.4)
Honduras	20 (12 to 31)	3 (1 to 6)	-84.57% (-94.12 to -64.72)	72.6 (42.6 to 113.7)	8.4 (3.2 to 17)	-7.64% (-8.03 to -7.25)
India	2057 (1221 to 3254)	315 (164 to 534)	-84.7% (-92.63 to -69.55)	53.9 (32 to 85.2)	7.6 (4 to 13)	-6.7% (-7.67 to -5.72)

Location	Deaths in 1990 (95% UI)	Deaths in 2019 (95% UI)	Relative change (%, 95 CI)	ASMMR in 1990 (95% UI)	ASMMR in 2019 (95% UI)	EAPC (%, 95 CI)
Indonesia	256 (140 to 424)	35 (19 to 59)	-86.18% (-93.44 to -71.25)	32.3 (17.7 to 53.4)	4.1 (2.2 to 6.9)	-6.75% (-7.1 to -6.4)
Iran (Islamic Republic of)	29 (18 to 42)	3 (2 to 5)	-89.76% (-94.49 to -80.52)	11.6 (7.3 to 16.8)	0.9 (0.5 to 1.5)	-9.31% (-9.76 to -8.87)
Iraq	1 (1 to 3)	1 (0 to 1)	-50.73% (-80.8 to 35.4)	1 (0.5 to 1.9)	0.3 (0.1 to 0.6)	-3.82% (-4.82 to -2.81)
Jamaica	1 (0 to 1)	0 (0 to 0)	-75.32% (-90.27 to -42.76)	7.3 (4 to 11.7)	2 (0.9 to 3.5)	-6.24% (-7.81 to -4.65)
Jordan	2 (1 to 4)	1 (0 to 1)	-68.78% (-87.04 to -21.23)	10.2 (5.2 to 18)	1.3 (0.6 to 2.4)	-8.76% (-9.42 to -8.1)
Kazakhstan	0 (0 to 1)	1 (0 to 1)	121.75% (-1.34 to 394.69)	0.5 (0.3 to 0.8)	0.9 (0.5 to 1.5)	2.12% (1.07 to 3.18)
Kenya	45 (26 to 74)	48 (27 to 82)	8.57% (-42.79 to 100.5)	29.2 (16.8 to 48.1)	20.1 (11.1 to 34)	-1.04% (-1.72 to -0.35)

Location	Deaths in 1990 (95% UI)	Deaths in 2019 (95% UI)	Relative change (%, 95 CI)	ASMMR in 1990 (95% UI)	ASMMR in 2019 (95% UI)	EAPC (%, 95 CI)
Kiribati	0 (0 to 0)	0 (0 to 0)	-0.63% (-65.39 to 162.14)	16.3 (7.3 to 31.4)	11.2 (4.6 to 21.1)	-1.31% (-1.5 to -1.13)
Kyrgyzstan	1 (0 to 1)	1 (1 to 2)	21.44% (-39.24 to 143.54)	3.5 (2.1 to 5.7)	3.3 (2 to 5.2)	1.11% (0.06 to 2.18)
Lao People's Democratic Republic	21 (12 to 34)	2 (1 to 4)	-90.8% (-96.17 to -80.53)	78.3 (46.1 to 126.5)	6.4 (3 to 12.1)	-8.22% (-9.01 to -7.42)
Lebanon	1 (0 to 1)	0 (0 to 0)	-84.03% (-93.88 to -60.45)	3.5 (1.6 to 6.4)	0.4 (0.1 to 0.7)	-8.46% (-8.97 to -7.95)
Lesotho	5 (3 to 9)	4 (2 to 7)	-32.18% (-73.05 to 55.79)	58.4 (28.7 to 98.3)	44.6 (18.5 to 86.3)	1.35% (0.37 to 2.35)
Liberia	14 (8 to 23)	23 (13 to 38)	64.69% (-17.76 to 203.01)	91.2 (53.5 to 145.9)	92.6 (52 to 149.7)	-1.12% (-2.08 to -0.15)
Libya	1 (0 to 1)	0 (0 to 0)	-69.63% (-89.09 to -16.01)	2.5 (1.1 to 4.7)	1 (0.4 to 2)	-3.93% (-4.8 to -3.06)

Location	Deaths in 1990 (95% UI)	Deaths in 2019 (95% UI)	Relative change (%, 95 CI)	ASMMR in 1990 (95% UI)	ASMMR in 2019 (95% UI)	EAPC (%, 95 CI)
Madagascar	50 (27 to 80)	61 (31 to 102)	22.29% (-41.25 to 138.38)	55.6 (30.8 to 90.3)	43.8 (22.2 to 73.9)	-0.72% (-1.09 to -0.34)
Malawi	24 (13 to 40)	20 (10 to 37)	-15.95% (-63.09 to 93.4)	34.6 (18.3 to 57.7)	21.8 (10.8 to 39.6)	-1.35% (-1.72 to -0.99)
Malaysia	11 (6 to 20)	2 (1 to 4)	-80.63% (-92.39 to -49.88)	9.6 (5.1 to 16.9)	1.3 (0.6 to 2.6)	-7.17% (-7.57 to -6.76)
Maldives	0 (0 to 0)	0 (0 to 0)	-91.01% (-96 to -80.64)	9.3 (4.9 to 16.2)	0.5 (0.3 to 0.9)	-10.02% (-10.69 to -9.35)
Mali	104 (65 to 152)	68 (35 to 113)	-34.68% (-67.26 to 16.54)	132.6 (82.7 to 194.2)	44.5 (23.2 to 74.3)	-3.99% (-4.29 to -3.69)
Marshall Islands	0 (0 to 0)	0 (0 to 0)	-22.58% (-76.22 to 91.72)	18.1 (8.7 to 32.6)	13.6 (5.2 to 27.6)	-1.63% (-2.51 to -0.75)
Mauritania	23 (15 to 35)	6 (3 to 11)	-74.39% (-87.57 to -51.22)	141.4 (88.1 to 210.8)	25.7 (12.6 to 45.4)	-6.46% (-6.76 to -6.17)

Location	Deaths in 1990 (95% UI)	Deaths in 2019 (95% UI)	Relative change (%, 95 CI)	ASMMR in 1990 (95% UI)	ASMMR in 2019 (95% UI)	EAPC (%, 95 CI)
Mexico	18 (11 to 28)	6 (3 to 10)	-67.18% (-84.87 to -30.51)	4.6 (2.7 to 7.1)	1.4 (0.8 to 2.5)	-3.76% (-3.98 to -3.54)
Micronesia (Federated States of)	0 (0 to 0)	0 (0 to 0)	-74.45% (-96.47 to -31.83)	26.5 (11.7 to 51.9)	11.3 (1.3 to 23.9)	-3.62% (-4.09 to -3.14)
Mongolia	4 (2 to 7)	1 (1 to 2)	-72.94% (-88.8 to -30.56)	36.5 (19.1 to 61.7)	5.9 (2.7 to 11.5)	-8.36% (-9.41 to -7.31)
Montenegro	0 (0 to 0)	0 (0 to 0)	-65.22% (-88.48 to 6.89)	0.1 (0 to 0.1)	0 (0 to 0)	-5.2% (-6.27 to -4.13)
Morocco	56 (30 to 95)	4 (2 to 8)	-93.08% (-96.88 to -84.25)	35.2 (18.9 to 59.6)	2.5 (1.1 to 5.1)	-8.95% (-9.25 to -8.64)
Mozambique	53 (29 to 88)	21 (10 to 39)	-60.34% (-83.13 to -16.66)	54.6 (30.1 to 90.6)	13.3 (6.1 to 24.4)	-4.51% (-5.03 to -3.99)
Myanmar	85 (40 to 159)	23 (11 to 42)	-73.04% (-88.81 to -35.56)	37.5 (17.4 to 69.8)	10.1 (4.7 to 18.6)	-4.86% (-5.43 to -4.28)

Location	Deaths in 1990 (95% UI)	Deaths in 2019 (95% UI)	Relative change (%, 95 CI)	ASMMR in 1990 (95% UI)	ASMMR in 2019 (95% UI)	EAPC (%, 95 CI)
Namibia	2 (1 to 3)	1 (0 to 2)	-61.63% (-87.01 to 12.64)	23.7 (10.2 to 40.6)	6.3 (2.3 to 13.9)	-4.39% (-4.97 to -3.81)
Nepal	83 (45 to 139)	7 (3 to 13)	-91.75% (-96.63 to -82.17)	67.4 (36.1 to 112.4)	9.5 (4.2 to 17.6)	-6.29% (-6.8 to -5.77)
Nicaragua	3 (2 to 5)	0 (0 to 1)	-87.78% (-94.94 to -72.06)	16.7 (9.2 to 27.4)	1.7 (0.8 to 3.3)	-8.31% (-8.72 to -7.91)
Niger	70 (38 to 109)	95 (49 to 159)	35.16% (-33.96 to 156.08)	94.3 (51.3 to 146.2)	56.6 (29.2 to 94.6)	-2.04% (-2.25 to -1.82)
Nigeria	154 (80 to 286)	163 (79 to 302)	5.92% (-52.1 to 137.17)	20.7 (10.7 to 38.5)	11 (5.3 to 20.3)	-2.95% (-3.69 to -2.2)
North Macedonia	0 (0 to 0)	0 (0 to 0)	-94.21% (-97.93 to -84.67)	1.9 (0.9 to 3.4)	0.1 (0 to 0.2)	-12.96% (-14.03 to -11.88)
Pakistan	256 (145 to 395)	142 (72 to 248)	-44.5% (-73.84 to 16.43)	31.7 (17.9 to 48.9)	13.4 (6.8 to 23.4)	-3.33% (-3.63 to -3.03)

Location	Deaths in 1990 (95% UI)	Deaths in 2019 (95% UI)	Relative change (%, 95 CI)	ASMMR in 1990 (95% UI)	ASMMR in 2019 (95% UI)	EAPC (%, 95 CI)
Papua New Guinea	13 (7 to 23)	15 (7 to 27)	11.35% (-52.44 to 137.05)	48.5 (24.3 to 82)	22.2 (10.8 to 40.5)	-1.92% (-2.39 to -1.45)
Paraguay	6 (3 to 9)	1 (1 to 2)	-80.75% (-91.56 to - 55.35)	25.9 (14.9 to 40.6)	4 (1.9 to 7.3)	-6.58% (-7.25 to -5.9)
Peru	38 (22 to 61)	4 (2 to 8)	-89.77% (-95.53 to - 77.93)	34.4 (20.1 to 55.9)	2.9 (1.3 to 5.9)	-9.35% (-9.87 to -8.82)
Philippines	31 (19 to 49)	12 (6 to 20)	-62.82% (-83.01 to - 23.16)	8.3 (5 to 12.9)	2.4 (1.3 to 4.1)	-3.93% (-4.11 to -3.75)
Republic of Moldova	0 (0 to 1)	0 (0 to 0)	-97.22% (-98.77 to - 93.75)	3.8 (2.2 to 6.1)	0.2 (0.1 to 0.3)	-11.73% (-12.8 to -10.66)
Russian Federation	10 (6 to 16)	2 (1 to 3)	-80.88% (-91.08 to -60.1)	3.5 (2.1 to 5.5)	0.4 (0.2 to 0.6)	-9.79% (-10.7 to -8.88)
Rwanda	90 (54 to 134)	29 (14 to 51)	-67.69% (-85.41 to - 35.13)	130.6 (78.8 to 195.1)	33.5 (16.3 to 59)	-5.08% (-5.91 to -4.25)

Location	Deaths in 1990 (95% UI)	Deaths in 2019 (95% UI)	Relative change (%, 95 CI)	ASMMR in 1990 (95% UI)	ASMMR in 2019 (95% UI)	EAPC (%, 95 CI)
Saint Lucia	0 (0 to 0)	0 (0 to 0)	-66.44% (-84.94 to -26.19)	4.5 (2.6 to 7.5)	2.1 (1.1 to 3.6)	-0.38% (-1.72 to 0.97)
Saint Vincent and the Grenadines	0 (0 to 0)	0 (0 to 0)	-40.85% (-71.82 to 28.17)	0.9 (0.5 to 1.5)	0.7 (0.4 to 1.3)	-0.89% (-1.5 to -0.27)
Samoa	0 (0 to 0)	0 (0 to 0)	-76.84% (-95.45 to -31.92)	12.1 (5.3 to 23.6)	1.9 (0.4 to 4.1)	-7.57% (-8.26 to -6.88)
Senegal	31 (18 to 51)	26 (13 to 46)	-17.2% (-57.72 to 65.56)	51.4 (29.4 to 83.6)	26.2 (13.5 to 46)	-2.25% (-2.58 to -1.92)
Serbia	0 (0 to 0)	0 (0 to 0)	-89.62% (-96.43 to -71.56)	1.1 (0.5 to 2.1)	0.1 (0 to 0.2)	-9.5% (-10.45 to -8.55)
Sierra Leone	15 (8 to 25)	28 (15 to 48)	90.58% (-6.62 to 294.27)	51.3 (27.4 to 87.2)	53 (28.1 to 91.1)	0.44% (-0.27 to 1.17)
Solomon Islands	1 (1 to 2)	2 (1 to 3)	47.77% (-43.3 to 262.03)	46.4 (21 to 86.4)	42.2 (18.1 to 80)	0.32% (-0.31 to 0.95)

Location	Deaths in 1990 (95% UI)	Deaths in 2019 (95% UI)	Relative change (%, 95 CI)	ASMMR in 1990 (95% UI)	ASMMR in 2019 (95% UI)	EAPC (%, 95 CI)
Somalia	72 (35 to 123)	161 (87 to 262)	122.56% (15.17 to 347.74)	111.5 (53.9 to 189.6)	115.7 (62.9 to 188.4)	0.26% (0.1 to 0.42)
South Africa	60 (36 to 92)	18 (8 to 35)	-69.33% (-87.73 to -31.55)	37.8 (22.9 to 58.1)	8.6 (3.8 to 16.4)	-5.05% (-7.55 to -2.49)
South Sudan	12 (6 to 24)	13 (6 to 26)	6.94% (-54.64 to 167.72)	30.4 (13.7 to 59.2)	21.7 (9.5 to 42.8)	-1.37% (-1.64 to -1.11)
Sri Lanka	2 (1 to 4)	0 (0 to 1)	-87.56% (-95.07 to -69.1)	3.2 (1.6 to 5.3)	0.4 (0.2 to 0.7)	-7.55% (-7.81 to -7.29)
Sudan	140 (77 to 223)	36 (14 to 68)	-74.19% (-90.41 to -44.62)	77.9 (42.9 to 123.8)	13.2 (5.3 to 24.7)	-5.97% (-6.41 to -5.54)
Suriname	0 (0 to 0)	0 (0 to 0)	10.36% (-56.68 to 193.27)	6 (2.8 to 11.1)	4.7 (2.2 to 9.2)	-1.12% (-2 to -0.24)
Syrian Arab Republic	6 (3 to 10)	0 (0 to 1)	-93.49% (-97.73 to -82.66)	6.7 (3.3 to 11.9)	0.8 (0.3 to 1.6)	-9.54% (-10.74 to -8.34)

Location	Deaths in 1990 (95% UI)	Deaths in 2019 (95% UI)	Relative change (%, 95 CI)	ASMMR in 1990 (95% UI)	ASMMR in 2019 (95% UI)	EAPC (%, 95 CI)
Tajikistan	1 (1 to 2)	0 (0 to 1)	-60.89% (-85.36 to -9.51)	3.5 (1.9 to 5.9)	0.9 (0.4 to 2)	-6.14% (-6.89 to -5.39)
Thailand	4 (2 to 8)	2 (1 to 3)	-59.43% (-86.12 to 13.7)	2.4 (1 to 4.7)	1.3 (0.6 to 2.6)	-1.89% (-3.07 to -0.69)
Timor-Leste	4 (2 to 7)	1 (0 to 2)	-73.73% (-97.04 to -32.42)	56.2 (26.1 to 98.4)	13.7 (1.5 to 26.4)	-6.01% (-6.67 to -5.34)
Togo	13 (7 to 21)	13 (6 to 22)	0.87% (-55.86 to 120.43)	43.8 (23.7 to 72)	23 (11 to 40.7)	-2.06% (-2.42 to -1.7)
Tonga	0 (0 to 0)	0 (0 to 0)	-62.76% (-86.22 to -6.64)	11.2 (5.5 to 19.4)	3.6 (1.5 to 7)	-4.35% (-4.54 to -4.16)
Tunisia	4 (2 to 7)	0 (0 to 1)	-89.99% (-96.48 to -75.7)	8.5 (4.2 to 14.7)	0.8 (0.3 to 1.7)	-7.32% (-7.68 to -6.97)
Turkey	46 (25 to 81)	3 (1 to 5)	-94.59% (-97.69 to -87.07)	22.3 (11.9 to 38.9)	1.1 (0.6 to 2)	-11.62% (-12.69 to -10.54)
Turkmenistan	0 (0 to 1)	0 (0 to 0)	-42.42% (-73.76 to 16.97)	1.2 (0.6 to 2)	0.7 (0.3 to 1.2)	-2.14% (-2.78 to -1.49)

Location	Deaths in 1990 (95% UI)	Deaths in 2019 (95% UI)	Relative change (%, 95 CI)	ASMMR in 1990 (95% UI)	ASMMR in 2019 (95% UI)	EAPC (%, 95 CI)
Tuvalu	0 (0 to 0)	0 (0 to 0)	-85.28% (-94.95 to -58.51)	28.4 (12.9 to 52.3)	5.8 (2.2 to 12.1)	-5.01% (-5.25 to -4.77)
Uganda	28 (11 to 53)	35 (17 to 64)	24.85% (-49.74 to 233.83)	21 (8 to 39.6)	14 (6.9 to 25.4)	-1.69% (-2.18 to -1.19)
Ukraine	1 (1 to 2)	0 (0 to 1)	-58.71% (-82.08 to -11.05)	1.4 (0.7 to 2.3)	0.5 (0.2 to 0.9)	-6.02% (-6.93 to -5.11)
United Republic of Tanzania	64 (35 to 107)	52 (26 to 93)	-18.6% (-62.98 to 81.4)	32.3 (17.6 to 54.3)	15 (7.6 to 26.6)	-2.03% (-2.3 to -1.76)
Uzbekistan	5 (3 to 8)	3 (2 to 5)	-43.53% (-73.23 to 15.33)	5 (2.9 to 7.5)	2.5 (1.3 to 4.1)	-1.21% (-2.32 to -0.08)
Vanuatu	0 (0 to 0)	0 (0 to 0)	26.06% (-65.9 to 238.65)	13.3 (5.2 to 26.4)	11.3 (2.9 to 23)	-1.35% (-1.85 to -0.84)
Venezuela (Bolivarian Republic of)	18 (11 to 26)	7 (4 to 12)	-58.81% (-79.89 to -14.46)	19.8 (12.5 to 28.3)	8.9 (4.8 to 14.8)	-2.9% (-3.92 to -1.88)

Location	Deaths in 1990 (95% UI)	Deaths in 2019 (95% UI)	Relative change (%, 95 CI)	ASMMR in 1990 (95% UI)	ASMMR in 2019 (95% UI)	EAPC (%, 95 CI)
Viet Nam	13 (6 to 26)	0 (0 to 1)	-97.09% (-98.93 to -91.5)	4.6 (2 to 9.2)	0.1 (0.1 to 0.3)	-12.41% (-12.68 to -12.14)
Yemen	62 (26 to 114)	72 (30 to 127)	15.47% (-52.39 to 171.07)	49.5 (20.2 to 90.6)	40 (16.5 to 70.7)	-1.47% (-1.82 to -1.13)
Zambia	18 (9 to 29)	10 (5 to 19)	-41.17% (-73.84 to 30.04)	30.6 (15.8 to 50)	9.5 (4.4 to 16.8)	-4.67% (-5.36 to -3.98)
Zimbabwe	19 (11 to 30)	41 (21 to 74)	121.17% (7.9 to 342.39)	31 (17.6 to 49.6)	52.9 (26.6 to 95.2)	5.26% (3.68 to 6.86)

ASMMR – age-standardized maternal mortality ratio (per 100,000 livebirths), CI – confidence interval, EAPC – estimated annual percentage change, MSMI – maternal sepsis and other maternal infections, UI – uncertain interval.

Table S21. MMR of MSMI in 131 low- and middle-income countries and territories with EAPC in age group of 35-39 years from 1990 and 2019.

Location	Deaths in 1990 (95% UI)	Deaths in 2019 (95% UI)	Relative change (%, 95 CI)	ASMMR in 1990 (95% UI)	ASMMR in 2019 (95% UI)	EAPC (%, 95 CI)
Afghanistan	41 (20 to 73)	42 (20 to 76)	4.24% (-52.25 to 129.09)	70.7 (35.5 to 127.7)	37.6 (18.1 to 67.8)	-2.75% (-3.73 to -1.76)
Albania	0 (0 to 0)	0 (0 to 0)	-70.43% (-89.17 to -24.97)	1.1 (0.5 to 2.1)	0.7 (0.3 to 1.2)	0.44% (-0.94 to 1.83)
Algeria	46 (24 to 80)	6 (3 to 10)	-87.46% (-94.89 to -73.06)	34.4 (18.3 to 60.3)	3 (1.4 to 5.4)	-8.77% (-9.03 to -8.5)
American Samoa	0 (0 to 0)	0 (0 to 0)	-66.61% (-86.83 to -17.44)	16.8 (8.5 to 30.6)	7.1 (3.2 to 13)	-3.39% (-3.66 to -3.12)
Angola	32 (15 to 59)	27 (12 to 51)	-16.23% (-65.85 to 107.77)	54.1 (25.3 to 99.1)	21.9 (10 to 40.8)	-3.08% (-3.35 to -2.81)
Armenia	0 (0 to 0)	0 (0 to 0)	-78.28% (-90.29 to -50.36)	3.2 (1.7 to 5.8)	0.8 (0.4 to 1.5)	-5.49% (-7.22 to -3.74)
Azerbaijan	1 (1 to 2)	0 (0 to 0)	-88.82% (-95.24 to -75.06)	12.7 (6.8 to 21.4)	1.8 (0.8 to 3.6)	-8.87% (-10.08 to -7.64)

Location	Deaths in 1990 (95% UI)	Deaths in 2019 (95% UI)	Relative change (%, 95 CI)	ASMMR in 1990 (95% UI)	ASMMR in 2019 (95% UI)	EAPC (%, 95 CI)
Bangladesh	54 (26 to 99)	2 (1 to 4)	-95.97% (-98.43 to -89.15)	21.8 (10.4 to 39.5)	1.5 (0.7 to 2.9)	-8.87% (-9.9 to -7.83)
Belarus	0 (0 to 0)	0 (0 to 0)	-85.06% (-93.22 to -70)	4.2 (2.5 to 6.8)	0.3 (0.1 to 0.5)	-10.56% (-11.6 to -9.5)
Belize	0 (0 to 0)	0 (0 to 0)	90.32% (-9.79 to 283.38)	8.9 (5.3 to 13.7)	9.7 (5.3 to 15.8)	0.46% (-0.03 to 0.97)
Benin	22 (13 to 36)	19 (10 to 34)	-13.25% (-57.19 to 79.41)	87.9 (50.5 to 144.2)	39.1 (20.1 to 69.6)	-2.31% (-2.61 to -2.01)
Bhutan	0 (0 to 1)	0 (0 to 0)	-91.49% (-96.75 to -77.68)	16.9 (7.1 to 30.5)	2.3 (0.9 to 4.4)	-6.84% (-7.23 to -6.44)
Bolivia (Plurinational State of)	24 (13 to 42)	9 (4 to 16)	-64.21% (-85.39 to -18.46)	90 (46.6 to 154.1)	21.3 (9.4 to 38.6)	-5.49% (-5.8 to -5.17)
Bosnia and Herzegovina	0 (0 to 1)	0 (0 to 0)	-99.7% (-99.89 to -99.19)	10.5 (5.2 to 17.9)	0 (0 to 0.1)	-21.87% (-23.36 to -20.37)
Botswana	2 (1 to 3)	1 (0 to 1)	-64.51% (-87.41 to -7.29)	39.9 (18.1 to 73.7)	9.1 (3.7 to 18)	-5.34% (-6.38 to -4.28)
Brazil	71 (44 to 106)	26 (16 to 40)	-63.5% (-80.53 to -33.42)	32.1 (19.9 to 47.8)	8 (5.1 to 12.3)	-4.01% (-4.52 to -3.5)

Location	Deaths in 1990 (95% UI)	Deaths in 2019 (95% UI)	Relative change (%, 95 CI)	ASMMR in 1990 (95% UI)	ASMMR in 2019 (95% UI)	EAPC (%, 95 CI)
Bulgaria	1 (0 to 1)	0 (0 to 0)	-92.68% (-96.61 to -83.94)	26.3 (14.8 to 42.4)	0.7 (0.4 to 1.3)	-14.31% (-15.63 to -12.96)
Burkina Faso	36 (20 to 59)	31 (15 to 52)	-14.53% (-61.83 to 65.76)	71 (39.3 to 116.2)	28.5 (13.9 to 48.2)	-3.88% (-4.28 to -3.48)
Burundi	42 (21 to 70)	53 (29 to 89)	26.91% (-38.61 to 183.36)	122.5 (62.3 to 203.7)	82.5 (44.9 to 137.6)	-1.94% (-2.44 to -1.45)
Cabo Verde	0 (0 to 0)	0 (0 to 0)	-87.95% (-94.82 to -72.41)	17.2 (9.2 to 29.2)	1.5 (0.7 to 2.8)	-8.85% (-9.12 to -8.57)
Cambodia	39 (18 to 72)	9 (4 to 16)	-77.66% (-91.24 to -46.39)	75.1 (35.2 to 139.4)	22.8 (10.7 to 41.1)	-5.22% (-6.44 to -3.99)
Cameroon	28 (15 to 47)	34 (17 to 62)	19.3% (-45.36 to 154.82)	66.6 (36.3 to 110.3)	36.8 (18.9 to 67.1)	-2.05% (-2.41 to -1.69)
Central African Republic	23 (12 to 38)	49 (25 to 84)	113.77% (5.83 to 330.31)	189.4 (102 to 311)	258.8 (134 to 446.2)	1.04% (0.9 to 1.18)
Chad	43 (24 to 70)	56 (31 to 95)	30.64% (-32.58 to 142.21)	129.8 (73.2 to 212)	75.3 (42.2 to 127)	-1.9% (-2.24 to -1.55)

Location	Deaths in 1990 (95% UI)	Deaths in 2019 (95% UI)	Relative change (%, 95 CI)	ASMMR in 1990 (95% UI)	ASMMR in 2019 (95% UI)	EAPC (%, 95 CI)
China	257 (196 to 329)	4 (3 to 5)	-98.55% (-99.03 to -97.87)	25.8 (19.7 to 32.9)	0.3 (0.2 to 0.3)	-14.99% (-15.61 to -14.38)
Colombia	12 (7 to 18)	4 (2 to 8)	-61.68% (-82.38 to -14.11)	16.4 (9.6 to 25.5)	5.6 (3 to 9.7)	-3.45% (-4.02 to -2.87)
Comoros	1 (0 to 1)	0 (0 to 1)	-35.01% (-77.28 to 318.31)	31.5 (5.3 to 61.6)	20.6 (8.2 to 39.1)	-2.19% (-2.81 to -1.56)
Congo	7 (3 to 12)	7 (3 to 12)	-3.13% (-58.86 to 143.62)	61.4 (26.9 to 109.9)	31.1 (14.4 to 58.9)	-2.35% (-3.15 to -1.55)
Costa Rica	1 (0 to 1)	0 (0 to 0)	-78.22% (-90.1 to -53.99)	10.7 (6.4 to 16.5)	2.3 (1.2 to 4.1)	-5.71% (-6.72 to -4.69)
Cuba	2 (1 to 3)	0 (0 to 1)	-73.14% (-86.99 to -43.64)	27.8 (16.8 to 42.2)	6.1 (3.3 to 10.2)	-5.01% (-5.8 to -4.22)
Democratic People's Republic of Korea	9 (4 to 17)	8 (3 to 15)	-7.13% (-66.62 to 160.63)	16 (7.3 to 32.2)	38.1 (16 to 73)	3% (1.65 to 4.38)
Democratic Republic of the Congo	181 (93 to 308)	458 (250 to 731)	152.78% (28.17 to 422.3)	84.2 (43.3 to 143.1)	126 (68.7 to 200.8)	2.81% (1.83 to 3.81)

Location	Deaths in 1990 (95% UI)	Deaths in 2019 (95% UI)	Relative change (%, 95 CI)	ASMMR in 1990 (95% UI)	ASMMR in 2019 (95% UI)	EAPC (%, 95 CI)
Djibouti	2<br>(1 to 3)	3<br>(1 to 6)	83.88%<br>(-22.68 to 323.26)	72.3<br>(35.5 to 125.4)	57.8<br>(25 to 112.3)	-0.48%<br>(-0.91 to -0.05)
Dominica	0<br>(0 to 0)	0<br>(0 to 0)	-31.3%<br>(-69.96 to 59.15)	20.9<br>(11.4 to 34.8)	21.6<br>(11 to 36.8)	-0.89%<br>(-1.69 to -0.08)
Dominican Republic	2<br>(1 to 3)	1<br>(1 to 3)	-22.24%<br>(-67.58 to 97.51)	15.5<br>(8 to 25.9)	13<br>(6.1 to 25.5)	0.02%<br>(-0.64 to 0.68)
Ecuador	8<br>(5 to 12)	2<br>(1 to 4)	-67.05%<br>(-85.56 to -29.04)	28.1<br>(16.8 to 43.6)	7.4<br>(3.8 to 13.2)	-3.76%<br>(-4.78 to -2.72)
Egypt	61<br>(35 to 98)	4<br>(2 to 8)	-92.98%<br>(-96.88 to -84.79)	36.3<br>(20.7 to 58.5)	2.4<br>(1.1 to 4.5)	-10.64%<br>(-11.2 to -10.07)
El Salvador	9<br>(5 to 15)	1<br>(0 to 1)	-94.47%<br>(-97.74 to -87.28)	82.5<br>(46.2 to 132.8)	5.7<br>(2.4 to 11)	-8.85%<br>(-10.04 to -7.65)
Equatorial Guinea	5<br>(2 to 8)	0<br>(0 to 1)	-92.1%<br>(-97.15 to -79.44)	173.6<br>(85.9 to 290.5)	9.7<br>(3.8 to 19.9)	-10.71%<br>(-11.52 to -9.9)
Eritrea	30<br>(15 to 52)	24<br>(12 to 42)	-20.11%<br>(-63.65 to 74.29)	173.2<br>(87.7 to 298)	80.5<br>(41.8 to 140.8)	-1.63%<br>(-2.27 to -0.99)

Location	Deaths in 1990 (95% UI)	Deaths in 2019 (95% UI)	Relative change (%, 95 CI)	ASMMR in 1990 (95% UI)	ASMMR in 2019 (95% UI)	EAPC (%, 95 CI)
Eswatini	1 (0 to 2)	0 (0 to 1)	-49.5% (-81.42 to 30.63)	32.7 (15.5 to 59.6)	15 (5.9 to 31.5)	-1.62% (-2.83 to -0.4)
Ethiopia	668 (354 to 1067)	202 (112 to 332)	-69.82% (-84.79 to -42.78)	229 (121.2 to 365.5)	44.4 (24.7 to 73.2)	-5.85% (-6.56 to -5.14)
Fiji	0 (0 to 1)	0 (0 to 0)	-46.19% (-80.28 to 39.37)	20.4 (9.5 to 37.7)	7.3 (3.2 to 13.8)	-3.87% (-4.18 to -3.56)
Gabon	2 (1 to 3)	1 (0 to 1)	-61.06% (-84.97 to -8.44)	40.7 (19.5 to 70.6)	11.9 (4.9 to 22.6)	-4.62% (-5.31 to -3.93)
Gambia	3 (2 to 5)	5 (3 to 9)	60.09% (-21.04 to 218.46)	60.4 (31.1 to 102.9)	52.8 (27.4 to 92.8)	-0.64% (-0.9 to -0.38)
Georgia	0 (0 to 0)	0 (0 to 0)	-20.26% (-66.42 to 86.68)	8.6 (4.5 to 14.8)	5.2 (2.7 to 9.3)	0.86% (-1.05 to 2.81)
Ghana	25 (13 to 43)	11 (6 to 20)	-54.81% (-79.75 to -5.78)	38.2 (19.4 to 64.2)	10.9 (5.6 to 19.4)	-3.98% (-4.26 to -3.69)
Global	5182 (4336 to 6211)	2717 (2251 to 3308)	-47.56% (-58.46 to -34.36)	49.7 (41.6 to 59.5)	19.4 (16.1 to 23.7)	-3.25% (-3.6 to -2.9)
Grenada	0 (0 to 0)	0 (0 to 0)	-75.64% (-88.44 to -48.81)	3.6 (2.1 to 5.7)	0.7 (0.4 to 1.2)	-5.78% (-6.64 to -4.92)

Location	Deaths in 1990 (95% UI)	Deaths in 2019 (95% UI)	Relative change (%, 95 CI)	ASMMR in 1990 (95% UI)	ASMMR in 2019 (95% UI)	EAPC (%, 95 CI)
Guatemala	11 (6 to 18)	4 (2 to 7)	-62.06% (-82.02 to -21.23)	30.9 (17.2 to 51.2)	10 (5.4 to 17.8)	-5.4% (-6.54 to -4.25)
Guinea	38 (21 to 59)	38 (20 to 66)	0.12% (-51.1 to 96.32)	116 (64.8 to 180.5)	83.5 (43.4 to 145)	-0.59% (-0.76 to -0.41)
Guinea-Bissau	3 (2 to 6)	2 (1 to 4)	-36.89% (-73.58 to 38.72)	62.7 (31.3 to 108)	27.5 (13.2 to 50.7)	-2.36% (-2.7 to -2.02)
Guyana	0 (0 to 0)	0 (0 to 0)	-67.77% (-87.18 to -21.86)	26.6 (14 to 45.9)	9.2 (4.7 to 16.7)	-3.92% (-4.5 to -3.34)
Haiti	38 (18 to 70)	67 (36 to 114)	75.18% (-18.69 to 280.07)	116.2 (54 to 212.7)	130 (69.7 to 221)	1.24% (0.9 to 1.58)
Honduras	16 (9 to 26)	3 (1 to 6)	-82.76% (-92.98 to -60.95)	92.3 (50.4 to 149)	14.3 (5.9 to 28.3)	-6.86% (-7.15 to -6.56)
India	1366 (771 to 2146)	232 (125 to 405)	-82.99% (-91.77 to -65.61)	74.5 (42 to 117)	17.1 (9.2 to 29.8)	-4.88% (-5.79 to -3.96)
Indonesia	166 (89 to 284)	27 (15 to 47)	-83.9% (-92.61 to -64.34)	44.7 (24 to 76.4)	5.5 (3 to 9.6)	-7.04% (-7.33 to -6.75)
Iran (Islamic Republic of)	22 (14 to 32)	3 (1 to 4)	-88.31% (-94.06 to -77.49)	14.4 (9.2 to 21.1)	1.2 (0.7 to 1.8)	-9.1% (-9.46 to -8.73)

Location	Deaths in 1990 (95% UI)	Deaths in 2019 (95% UI)	Relative change (%, 95 CI)	ASMMR in 1990 (95% UI)	ASMMR in 2019 (95% UI)	EAPC (%, 95 CI)
Iraq	1 (1 to 2)	1 (0 to 1)	-52% (-82.55 to 25.93)	1.4 (0.6 to 2.8)	0.5 (0.2 to 1)	-3.17% (-4.19 to -2.14)
Jamaica	0 (0 to 1)	0 (0 to 0)	-83.53% (-92.82 to -61.01)	12.5 (6.9 to 21.7)	1.8 (0.9 to 3.3)	-9% (-10.49 to - 7.48)
Jordan	2 (1 to 3)	1 (0 to 1)	-65.16% (-86.43 to -12.02)	14.7 (7.5 to 24.2)	2.1 (1 to 4)	-8.33% (-9 to -7.66)
Kazakhstan	0 (0 to 0)	1 (0 to 1)	116.93% (-8.63 to 401.76)	0.9 (0.5 to 1.6)	1.4 (0.8 to 2.5)	1.06% (-0.07 to 2.21)
Kenya	33 (19 to 53)	42 (23 to 72)	28.74% (-30.74 to 148.51)	38.3 (22 to 61.8)	35.8 (19.2 to 60.9)	0.13% (-0.64 to 0.91)
Kiribati	0 (0 to 0)	0 (0 to 0)	17.46% (-52.27 to 198.1)	40.6 (19.8 to 75.6)	33 (15.5 to 61.3)	-0.81% (-0.94 to -0.69)
Kyrgyzstan	0 (0 to 1)	1 (0 to 1)	74.72% (-17.18 to 253.12)	5 (2.8 to 8.2)	7.2 (4 to 11.6)	2.81% (1.7 to 3.92)
Lao People's Democratic Republic	12 (7 to 21)	1 (1 to 2)	-89.32% (-95.53 to -77.41)	76.9 (42.1 to 129)	9.1 (4.2 to 16.5)	-6.92% (-7.68 to -6.16)

Location	Deaths in 1990 (95% UI)	Deaths in 2019 (95% UI)	Relative change (%, 95 CI)	ASMMR in 1990 (95% UI)	ASMMR in 2019 (95% UI)	EAPC (%, 95 CI)
Lebanon	1 (0 to 1)	0 (0 to 0)	-87.19% (-95.21 to -66.9)	6.3 (3.1 to 11.4)	0.6 (0.3 to 1.2)	-8.71% (-9.19 to -8.23)
Lesotho	6 (3 to 9)	3 (2 to 7)	-37.53% (-75.94 to 47.43)	93.9 (47.6 to 155.9)	73.9 (32.4 to 143.4)	1.04% (0.19 to 1.89)
Liberia	15 (8 to 24)	24 (12 to 40)	59.83% (-19.56 to 213.97)	135.3 (75.4 to 216.9)	146.1 (76.8 to 246)	-0.9% (-1.79 to -0.01)
Libya	1 (0 to 1)	0 (0 to 1)	-63.33% (-85.65 to -8.7)	5 (2.4 to 9.1)	3.1 (1.4 to 6.1)	-2.73% (-3.77 to -1.67)
Madagascar	37 (21 to 59)	55 (28 to 98)	50.95% (-31.3 to 234.28)	66.7 (37.5 to 108.5)	68.7 (35 to 121.9)	0.06% (-0.36 to 0.48)
Malawi	25 (14 to 39)	19 (9 to 35)	-22.14% (-66.83 to 74.71)	49.8 (27.5 to 79.2)	36.4 (17.6 to 65.7)	-0.82% (-1.19 to -0.44)
Malaysia	10 (5 to 17)	2 (1 to 4)	-80.29% (-92.48 to -51.98)	17.3 (9 to 29.5)	2.4 (1.1 to 4.6)	-6.5% (-6.92 to -6.08)
Maldives	0 (0 to 0)	0 (0 to 0)	-88.49% (-94.86 to -74.57)	10.3 (4.8 to 18.1)	0.9 (0.4 to 1.6)	-9.34% (-9.98 to -8.69)
Mali	92 (56 to 141)	64 (33 to 113)	-30.33% (-65.74 to 29.2)	180.6 (110.6 to 277.7)	64.5 (33.1 to 113.8)	-3.73% (-4.01 to -3.45)

Location	Deaths in 1990 (95% UI)	Deaths in 2019 (95% UI)	Relative change (%, 95 CI)	ASMMR in 1990 (95% UI)	ASMMR in 2019 (95% UI)	EAPC (%, 95 CI)
Marshall Islands	0 (0 to 0)	0 (0 to 0)	-4.28% (-62.29 to 133.37)	46.9 (22.6 to 82.9)	44.4 (17.1 to 83.9)	-1.14% (-2.17 to -0.1)
Mauritania	24 (14 to 37)	6 (3 to 10)	-75.83% (-88.46 to -53.25)	212.4 (124.2 to 335.6)	38.7 (19.3 to 68.8)	-6.41% (-6.69 to -6.12)
Mexico	16 (9 to 24)	5 (3 to 8)	-69% (-85.38 to -33.32)	8 (4.8 to 12.4)	2.4 (1.3 to 4)	-3.73% (-4.04 to -3.42)
Micronesia (Federated States of)	0 (0 to 0)	0 (0 to 0)	-76.47% (-98.11 to -27.45)	46.3 (21.9 to 84.3)	20.1 (1.7 to 46.9)	-3.58% (-3.92 to -3.23)
Mongolia	5 (3 to 8)	1 (0 to 2)	-77.56% (-90.75 to -49.66)	111.9 (58.2 to 184.2)	11.4 (4.7 to 22)	-9.88% (-11.12 to -8.61)
Montenegro	0 (0 to 0)	0 (0 to 0)	-62.47% (-86.67 to 15)	0.1 (0 to 0.2)	0 (0 to 0.1)	-5.23% (-6.45 to -4)
Morocco	50 (27 to 86)	4 (2 to 8)	-91.99% (-96.75 to -81.16)	50 (26.5 to 85.4)	4.1 (1.8 to 8.3)	-8.51% (-8.92 to -8.11)
Mozambique	48 (25 to 81)	19 (8 to 34)	-61.45% (-84.44 to -14.01)	72.2 (36.7 to 120.9)	19 (8.4 to 35.1)	-4.18% (-4.77 to -3.59)

Location	Deaths in 1990 (95% UI)	Deaths in 2019 (95% UI)	Relative change (%, 95 CI)	ASMMR in 1990 (95% UI)	ASMMR in 2019 (95% UI)	EAPC (%, 95 CI)
Myanmar	56 (27 to 103)	21 (10 to 37)	-63.38% (-84.12 to -13.27)	42.2 (19.8 to 77)	15 (7.3 to 26.7)	-3.99% (-4.53 to -3.46)
Namibia	2 (1 to 4)	1 (0 to 2)	-64.08% (-84.59 to -1.55)	39.4 (18.3 to 68.8)	11.2 (4.8 to 23.2)	-4.37% (-5.07 to -3.67)
Nepal	66 (35 to 111)	6 (3 to 12)	-90.54% (-95.95 to -80.06)	95.3 (49.5 to 158.9)	26.2 (11.6 to 48.2)	-3.85% (-4.32 to -3.37)
Nicaragua	2 (1 to 3)	0 (0 to 1)	-84.1% (-93.45 to -62.39)	21.4 (11.5 to 35.9)	3.3 (1.6 to 5.8)	-7.5% (-7.92 to -7.07)
Niger	56 (31 to 89)	92 (48 to 157)	63.28% (-16.32 to 213.72)	148 (81.8 to 234.9)	90.4 (47.6 to 154.7)	-2.01% (-2.25 to -1.77)
Nigeria	109 (52 to 198)	125 (57 to 234)	14.87% (-49.26 to 168.44)	25.8 (12.2 to 46.8)	15.1 (6.9 to 28.2)	-2.6% (-3.29 to -1.9)
North Macedonia	0 (0 to 0)	0 (0 to 0)	-93.74% (-97.47 to -83.82)	5.5 (2.8 to 9.5)	0.2 (0.1 to 0.4)	-12.9% (-13.62 to -12.18)
Pakistan	254 (148 to 400)	156 (82 to 273)	-38.53% (-72.18 to 31.91)	56.6 (32.9 to 89.3)	42.6 (22.2 to 74.3)	-1.19% (-1.49 to -0.9)

Location	Deaths in 1990 (95% UI)	Deaths in 2019 (95% UI)	Relative change (%, 95 CI)	ASMMR in 1990 (95% UI)	ASMMR in 2019 (95% UI)	EAPC (%, 95 CI)
Papua New Guinea	12 (6 to 21)	15 (7 to 27)	24.52% (-41.6 to 178.31)	66.4 (32 to 117.8)	35.1 (16.7 to 64.2)	-1.46% (-1.9 to -1.02)
Paraguay	5 (3 to 8)	1 (0 to 2)	-81.69% (-92.38 to -58.66)	37.5 (21.7 to 59.9)	6.7 (3.3 to 11.9)	-6.48% (-7.2 to -5.77)
Peru	32 (18 to 52)	4 (2 to 7)	-88.25% (-95.09 to -75.94)	49.2 (28.6 to 81.1)	4.5 (2.1 to 8.5)	-9.07% (-9.64 to -8.49)
Philippines	24 (14 to 36)	10 (5 to 17)	-56.05% (-78.71 to -3.74)	11.2 (6.5 to 17.1)	3.7 (1.9 to 6.1)	-3.45% (-3.65 to -3.25)
Republic of Moldova	0 (0 to 1)	0 (0 to 0)	-97.08% (-98.72 to -93.53)	10.7 (6 to 17.1)	0.4 (0.2 to 0.8)	-12.17% (-13.23 to -11.09)
Russian Federation	7 (4 to 11)	1 (1 to 2)	-81.77% (-91.75 to -63.54)	6.3 (3.9 to 9.8)	0.5 (0.3 to 0.8)	-11.36% (-12.53 to -10.17)
Rwanda	87 (54 to 138)	29 (15 to 50)	-67.04% (-85.21 to -33.05)	207.1 (128.1 to 327.3)	53 (27.3 to 92.5)	-5.18% (-5.94 to -4.41)
Saint Lucia	0 (0 to 0)	0 (0 to 0)	-57.09% (-81.42 to -6.24)	6.1 (3.5 to 10.1)	2.7 (1.4 to 4.5)	-1.15% (-2.63 to 0.36)
Saint Vincent and the Grenadines	0 (0 to 0)	0 (0 to 0)	-14.07% (-60.67 to 79.11)	1.2 (0.7 to 1.9)	0.9 (0.5 to 1.5)	-0.28% (-1.06 to 0.51)

Location	Deaths in 1990 (95% UI)	Deaths in 2019 (95% UI)	Relative change (%, 95 CI)	ASMMR in 1990 (95% UI)	ASMMR in 2019 (95% UI)	EAPC (%, 95 CI)
Samoa	0 (0 to 0)	0 (0 to 0)	-76.43% (-92.76 to -34.07)	25.1 (11.2 to 50.1)	4 (1.3 to 8)	-7.75% (-8.48 to -7.01)
Senegal	26 (14 to 42)	22 (11 to 37)	-15.83% (-61.54 to 65.42)	66.1 (36.4 to 105.8)	35.2 (17.9 to 60.3)	-2.06% (-2.37 to -1.76)
Serbia	0 (0 to 0)	0 (0 to 0)	-86.07% (-94.77 to -63.78)	2.2 (1.1 to 4)	0.2 (0.1 to 0.4)	-9.53% (-10.5 to -8.54)
Sierra Leone	12 (6 to 21)	21 (11 to 36)	71.12% (-23.52 to 241.3)	70.9 (36.3 to 119.2)	76.6 (39.3 to 131)	0.6% (-0.01 to 1.22)
Solomon Islands	1 (1 to 2)	2 (1 to 4)	71.85% (-28.57 to 314.13)	82.8 (36.1 to 154)	89.6 (40.8 to 163.4)	0.92% (0.38 to 1.46)
Somalia	76 (33 to 131)	165 (83 to 276)	117.89% (0.84 to 398.05)	149.3 (65.4 to 258.5)	155.1 (78.1 to 259.6)	0.23% (0.07 to 0.4)
South Africa	34 (20 to 54)	10 (4 to 19)	-70.38% (-87.55 to -32.5)	39.1 (22.4 to 61.9)	9 (3.9 to 16.6)	-4.15% (-5.75 to -2.53)
South Sudan	9 (4 to 18)	13 (6 to 24)	35.86% (-45.71 to 204.29)	39.4 (18.5 to 76)	28.7 (12.6 to 55)	-1.23% (-1.47 to -0.99)
Sri Lanka	2 (1 to 3)	0 (0 to 1)	-84.26% (-94.02 to -60.23)	5.2 (2.6 to 9.1)	0.7 (0.3 to 1.3)	-7.17% (-7.47 to -6.88)

Location	Deaths in 1990 (95% UI)	Deaths in 2019 (95% UI)	Relative change (%, 95 CI)	ASMMR in 1990 (95% UI)	ASMMR in 2019 (95% UI)	EAPC (%, 95 CI)
Sudan	149<br>(87 to 241)	39<br>(17 to 76)	-73.73%<br>(-89.37 to -42.97)	135.7<br>(78.7 to 219)	23.8<br>(10.3 to 46.3)	-5.88%<br>(-6.29 to -5.47)
Suriname	0<br>(0 to 0)	0<br>(0 to 0)	3.61%<br>(-61.31 to 169.32)	12.8<br>(6.4 to 23.2)	7.1<br>(3.2 to 13.3)	-1.69%<br>(-2.47 to -0.9)
Syrian Arab Republic	4<br>(2 to 8)	1<br>(0 to 1)	-83.18%<br>(-93.45 to -57.72)	9.4<br>(4.5 to 16.9)	2.1<br>(0.9 to 3.8)	-7.33%<br>(-8.36 to -6.3)
Tajikistan	1<br>(0 to 1)	0<br>(0 to 0)	-67.55%<br>(-87.27 to -24.12)	3.6<br>(1.8 to 6.3)	1<br>(0.4 to 1.9)	-5.83%<br>(-6.68 to -4.97)
Thailand	3<br>(1 to 5)	2<br>(1 to 4)	-32.39%<br>(-77.05 to 87.92)	4<br>(1.8 to 7.9)	3.1<br>(1.4 to 6)	0.29%<br>(-0.43 to 1.01)
Timor-Leste	4<br>(2 to 6)	1<br>(0 to 2)	-68.33%<br>(-96.47 to -17.77)	75.6<br>(36.5 to 133.9)	24.2<br>(2.7 to 45.4)	-5.24%<br>(-5.92 to -4.56)
Togo	14<br>(7 to 22)	14<br>(7 to 26)	2.14%<br>(-52.1 to 109.26)	73.1<br>(39.5 to 118.6)	43.6<br>(21.9 to 79)	-1.54%<br>(-1.96 to -1.13)
Tonga	0<br>(0 to 0)	0<br>(0 to 0)	-59.12%<br>(-84.02 to -5.04)	22<br>(10.7 to 37.7)	8.1<br>(3.5 to 15.4)	-3.85%<br>(-4.04 to -3.66)
Tunisia	3<br>(2 to 5)	0<br>(0 to 1)	-86.09%<br>(-94.41 to -64.58)	12.4<br>(6.4 to 20.9)	1.2<br>(0.5 to 2.6)	-7.81%<br>(-8.24 to -7.38)

Location	Deaths in 1990 (95% UI)	Deaths in 2019 (95% UI)	Relative change (%, 95 CI)	ASMMR in 1990 (95% UI)	ASMMR in 2019 (95% UI)	EAPC (%, 95 CI)
Turkey	38<br>(19 to 65)	2<br>(1 to 4)	-94.04%<br>(-97.27 to -86.24)	45.7<br>(23.3 to 77.2)	2<br>(1 to 3.6)	-12.14%<br>(-13.11 to -11.16)
Turkmenistan	0<br>(0 to 1)	0<br>(0 to 0)	-48.67%<br>(-75.68 to 9.16)	3.3<br>(1.7 to 5.4)	2<br>(1 to 3.6)	-2.07%<br>(-2.8 to -1.33)
Tuvalu	0<br>(0 to 0)	0<br>(0 to 0)	-85.3%<br>(-94.48 to -66.04)	61.5<br>(29.3 to 113.5)	13.1<br>(5.4 to 26.8)	-4.96%<br>(-5.14 to -4.77)
Uganda	21<br>(9 to 38)	25<br>(12 to 44)	17.51%<br>(-49.2 to 188)	26.8<br>(11.6 to 48.5)	17.9<br>(8.8 to 31.7)	-1.57%<br>(-2 to -1.14)
Ukraine	1<br>(0 to 1)	0<br>(0 to 1)	-64.58%<br>(-83.86 to -28.27)	3<br>(1.5 to 4.9)	0.6<br>(0.3 to 1.2)	-6.89%<br>(-7.78 to -5.99)
United Republic of Tanzania	56<br>(31 to 91)	46<br>(25 to 80)	-17.01%<br>(-62.68 to 77.35)	47.1<br>(26 to 77)	21.5<br>(11.5 to 37.4)	-2.05%<br>(-2.35 to -1.75)
Uzbekistan	4<br>(2 to 7)	2<br>(1 to 3)	-61.36%<br>(-81.69 to -16.99)	10.5<br>(6.1 to 16.3)	4.7<br>(2.5 to 7.9)	-1.68%<br>(-2.54 to -0.8)
Vanuatu	0<br>(0 to 0)	0<br>(0 to 0)	30.51%<br>(-56.35 to 249.17)	26.2<br>(11.2 to 50.6)	27.6<br>(10.3 to 53.8)	-0.58%<br>(-1.14 to -0.02)

Location	Deaths in 1990 (95% UI)	Deaths in 2019 (95% UI)	Relative change (%, 95 CI)	ASMMR in 1990 (95% UI)	ASMMR in 2019 (95% UI)	EAPC (%, 95 CI)
Venezuela (Bolivarian Republic of)	13 (8 to 19)	5 (3 to 9)	-58.66% (-80 to -18.25)	28.8 (18.3 to 41.3)	12.9 (7.2 to 21.1)	-3.51% (-4.27 to -2.73)
Viet Nam	7 (3 to 13)	0 (0 to 0)	-96.34% (-98.59 to -90.27)	6.4 (2.7 to 12.2)	0.2 (0.1 to 0.4)	-11.86% (-12.15 to -11.57)
Yemen	60 (28 to 106)	68 (29 to 125)	13.17% (-55.48 to 160.66)	69.9 (32.5 to 123.3)	58.4 (25 to 107.6)	-1.42% (-1.9 to -0.93)
Zambia	11 (6 to 19)	7 (3 to 13)	-36.96% (-72.94 to 41.53)	32.6 (17.6 to 55.7)	11.3 (5.4 to 21)	-4.56% (-5.23 to -3.88)
Zimbabwe	16 (9 to 26)	37 (19 to 64)	127.72% (7.06 to 382.01)	42.5 (23.6 to 69.2)	80.2 (40.6 to 139.3)	5.39% (3.88 to 6.92)

ASMMR – age-standardized maternal mortality ratio (per 100,000 livebirths), CI – confidence interval, EAPC – estimated annual percentage change, MSMI – maternal sepsis and other maternal infections, UI – uncertain interval.

Table S22. MMR of MSMI in 131 low- and middle-income countries and territories with EAPC in age group of 40-44 years from 1990 and 2019.

Location	Deaths in 1990 (95% UI)	Deaths in 2019 (95% UI)	Relative change (%, 95 CI)	ASMMR in 1990 (95% UI)	ASMMR in 2019 (95% UI)	EAPC (%, 95 CI)
Afghanistan	33 (15 to 61)	32 (14 to 61)	-3.11% (-59.58 to 141.48)	150.2 (68.5 to 279.4)	73.2 (32.6 to 140.6)	-3.2% (-4.14 to -2.25)
Albania	0 (0 to 0)	0 (0 to 0)	-60.12% (-86.27 to 7.32)	2.4 (1.2 to 4.4)	2.2 (1 to 4.5)	1.95% (0.61 to 3.3)
Algeria	16 (7 to 29)	3 (1 to 5)	-83.59% (-93.75 to -58.66)	42.2 (19.3 to 78.7)	3.6 (1.6 to 6.8)	-8.84% (-9.14 to -8.54)
American Samoa	0 (0 to 0)	0 (0 to 0)	-56.45% (-84.64 to 23.76)	34.9 (16 to 64.9)	21.4 (8.9 to 42)	-2.17% (-2.47 to -1.88)
Angola	20 (9 to 40)	24 (11 to 45)	16.15% (-52.22 to 178.78)	89.4 (39.4 to 174.3)	46.5 (21 to 88.8)	-2.4% (-2.58 to -2.21)
Armenia	0 (0 to 0)	0 (0 to 0)	-47.36% (-77.17 to 15.96)	7.9 (4.4 to 13.4)	4.5 (2.3 to 8.1)	-2.61% (-3.99 to -1.2)
Azerbaijan	0 (0 to 1)	0 (0 to 0)	-72.85% (-89.16 to -35.73)	28.5 (14 to 51)	9.2 (4.4 to 17.8)	-5.45% (-6.49 to -4.4)
Bangladesh	24 (12 to 43)	2 (1 to 3)	-93.16% (-97.61 to -81.88)	38 (18.6 to 69.3)	5.1 (2.2 to 9.6)	-6.71% (-7.83 to -5.58)

Location	Deaths in 1990 (95% UI)	Deaths in 2019 (95% UI)	Relative change (%, 95 CI)	ASMMR in 1990 (95% UI)	ASMMR in 2019 (95% UI)	EAPC (%, 95 CI)
Belarus	0 (0 to 0)	0 (0 to 0)	-83.25% (-92.01 to -67.14)	9.6 (5.1 to 15.5)	0.8 (0.4 to 1.6)	-10.07% (-11 to -9.14)
Belize	0 (0 to 0)	0 (0 to 0)	88.8% (-8.88 to 291.73)	9.9 (5.9 to 15.4)	18 (10.1 to 28.7)	2.54% (1.79 to 3.3)
Benin	15 (8 to 26)	14 (7 to 27)	-6.73% (-56.6 to 102.27)	180.3 (97.7 to 301.1)	77.1 (36.6 to 141.9)	-2.44% (-2.76 to -2.11)
Bhutan	0 (0 to 0)	0 (0 to 0)	-89.17% (-96.06 to -71.08)	24.1 (10 to 46.4)	5.2 (2.2 to 10.3)	-5.28% (-5.71 to -4.85)
Bolivia (Plurinational State of)	16 (8 to 26)	8 (3 to 14)	-50.5% (-81.25 to 13.33)	154.6 (83.5 to 264)	61.7 (27.5 to 113.1)	-3.67% (-3.98 to -3.37)
Bosnia and Herzegovina	0 (0 to 0)	0 (0 to 0)	-99.46% (-99.81 to -98.5)	25 (11.7 to 44.1)	0.2 (0.1 to 0.3)	-20.08% (-21.3 to -18.84)
Botswana	1 (0 to 1)	0 (0 to 0)	-66.66% (-88.37 to -9.11)	42.3 (17.5 to 82)	9.6 (3.8 to 20.2)	-5.55% (-6.57 to -4.52)
Brazil	34 (21 to 52)	12 (8 to 19)	-63.87% (-81.22 to -31.62)	49.7 (30 to 74.9)	14.9 (9.4 to 22.7)	-3.58% (-3.83 to -3.33)
Bulgaria	1 (0 to 1)	0 (0 to 0)	-89.71% (-95.31 to -76.84)	118.9 (67.5 to 195.6)	3.9 (2 to 6.8)	-13.64% (-15.04 to -12.22)
Burkina Faso	21 (11 to 38)	16 (7 to 30)	-26.66% (-69.28 to 59.62)	99.3 (50.7 to 177.7)	35.3 (16.7 to 66.6)	-4.34% (-4.8 to -3.88)

Location	Deaths in 1990 (95% UI)	Deaths in 2019 (95% UI)	Relative change (%, 95 CI)	ASMMR in 1990 (95% UI)	ASMMR in 2019 (95% UI)	EAPC (%, 95 CI)
Burundi	26 (12 to 46)	36 (19 to 63)	40.29% (-39.73 to 204.84)	176.2 (84.6 to 315.5)	121.9 (63.1 to 212.3)	-1.78% (-2.25 to -1.32)
Cabo Verde	0 (0 to 0)	0 (0 to 0)	-85.23% (-94.37 to -61.61)	14.3 (7 to 25.5)	1.8 (0.7 to 3.5)	-7.87% (-8.19 to -7.55)
Cambodia	22 (9 to 41)	6 (3 to 12)	-70.95% (-88.77 to -25.46)	129.2 (55.9 to 246.7)	77.1 (33.2 to 147.1)	-2.38% (-3.28 to -1.46)
Cameroon	22 (12 to 36)	22 (10 to 42)	0.46% (-57.12 to 128.98)	126.3 (66.9 to 205.2)	92.2 (40 to 174.1)	-1.16% (-1.4 to -0.92)
Central African Republic	15 (7 to 27)	34 (16 to 63)	126.53% (0.42 to 421.95)	304 (149.7 to 530.8)	486.6 (232.3 to 883.5)	1.63% (1.49 to 1.77)
Chad	31 (16 to 51)	40 (19 to 71)	29.31% (-37.94 to 171.85)	287.1 (150.1 to 472.3)	147.5 (71.4 to 263.5)	-2.41% (-2.77 to -2.05)
China	76 (58 to 98)	2 (2 to 3)	-97.22% (-98.09 to -95.88)	40.1 (30.7 to 51.9)	0.5 (0.4 to 0.7)	-14.81% (-15.63 to -13.99)
Colombia	4 (2 to 7)	2 (1 to 3)	-61.55% (-83.47 to -15.63)	19.1 (10.5 to 31.4)	9 (4.7 to 15.6)	-2.2% (-2.82 to -1.59)

Location	Deaths in 1990 (95% UI)	Deaths in 2019 (95% UI)	Relative change (%, 95 CI)	ASMMR in 1990 (95% UI)	ASMMR in 2019 (95% UI)	EAPC (%, 95 CI)
Comoros	1 (0 to 1)	0 (0 to 1)	-25.16% (-72.91 to 180.12)	53.3 (13.7 to 105.1)	47.8 (21.5 to 94.1)	-0.72% (-1.06 to -0.38)
Congo	4 (2 to 6)	4 (2 to 8)	23.19% (-53.18 to 215.8)	92.3 (42.8 to 166.9)	61.2 (26.1 to 118.2)	-1.15% (-1.66 to -0.63)
Costa Rica	0 (0 to 0)	0 (0 to 0)	-75.62% (-88.57 to -47.08)	15.2 (8.3 to 23.9)	4.2 (2.3 to 7.2)	-5.09% (-5.85 to -4.32)
Cuba	1 (0 to 1)	0 (0 to 0)	-68.09% (-85.42 to -27.36)	52.2 (28.9 to 84.3)	10.9 (5.9 to 18.3)	-6.3% (-6.96 to -5.64)
Democratic People's Republic of Korea	4 (2 to 9)	4 (2 to 8)	0.99% (-65.7 to 174.49)	35.8 (14.8 to 77.3)	135.1 (55.4 to 259.6)	6.51% (5.22 to 7.82)
Democratic Republic of the Congo	146 (71 to 256)	472 (249 to 799)	224.36% (40.98 to 606.69)	173.6 (84.1 to 305.1)	396.9 (209.2 to 671.5)	4.25% (3.3 to 5.22)
Djibouti	1 (0 to 2)	2 (1 to 4)	91.67% (-26.02 to 382.6)	106.7 (46.4 to 199.9)	108.7 (47.2 to 216.3)	0.55% (0.29 to 0.82)
Dominica	0 (0 to 0)	0 (0 to 0)	-27.93% (-70.13 to 68.07)	25.9 (13.2 to 44.9)	33.2 (16.4 to 59.5)	1.75% (1.36 to 2.15)
Dominican Republic	1 (0 to 1)	1 (0 to 1)	-25.75% (-72.03 to 93.21)	13.1 (6.7 to 23)	30.6 (13.6 to 57.8)	3.43% (2.96 to 3.9)

Location	Deaths in 1990 (95% UI)	Deaths in 2019 (95% UI)	Relative change (%, 95 CI)	ASMMR in 1990 (95% UI)	ASMMR in 2019 (95% UI)	EAPC (%, 95 CI)
Ecuador	4 (2 to 7)	1 (1 to 2)	-70.91% (-87.8 to -35.5)	39.9 (22.8 to 64.2)	14.5 (7.2 to 27.7)	-2.6% (-3.45 to -1.74)
Egypt	26 (13 to 42)	2 (1 to 4)	-92.08% (-96.81 to -81.45)	49 (25.4 to 80)	8.4 (3.8 to 15.7)	-7.73% (-8.39 to -7.07)
El Salvador	4 (2 to 8)	0 (0 to 1)	-93.88% (-97.48 to -83.92)	100.1 (50.4 to 175.5)	9.2 (4.1 to 18.2)	-7.43% (-8.62 to -6.23)
Equatorial Guinea	3 (1 to 5)	0 (0 to 1)	-90.48% (-96.61 to -75.08)	289.9 (138.7 to 520.1)	21.2 (8 to 46.2)	-9.64% (-10.6 to -8.67)
Eritrea	17 (8 to 30)	15 (7 to 28)	-9.37% (-58.9 to 114.33)	266.8 (126.9 to 481.7)	161.4 (76 to 299.2)	-0.63% (-1.38 to 0.11)
Eswatini	1 (0 to 1)	0 (0 to 1)	-57.41% (-85.31 to 15.06)	57.1 (26.9 to 105)	29.4 (10.7 to 61.5)	-1.32% (-2.64 to 0.03)
Ethiopia	458 (251 to 738)	163 (91 to 278)	-64.34% (-82.17 to -27.39)	456.9 (250.5 to 737.2)	107.3 (60.1 to 182.9)	-5.23% (-5.88 to -4.58)
Fiji	0 (0 to 0)	0 (0 to 0)	-43.61% (-79.96 to 57.71)	41.8 (19.8 to 82)	19.8 (8.8 to 40.2)	-2.65% (-2.8 to -2.5)
Gabon	1 (0 to 2)	1 (0 to 1)	-43.53% (-77.28 to 33.28)	85.5 (43.2 to 153.7)	32.5 (13.8 to 63.7)	-3.43% (-4.33 to -2.53)
Gambia	2 (1 to 4)	4 (2 to 6)	61.79% (-23.88 to 251.26)	112 (58.7 to 196.6)	118.1 (59.8 to 204.7)	-0.05% (-0.29 to 0.2)

Location	Deaths in 1990 (95% UI)	Deaths in 2019 (95% UI)	Relative change (%, 95 CI)	ASMMR in 1990 (95% UI)	ASMMR in 2019 (95% UI)	EAPC (%, 95 CI)
Georgia	0 (0 to 0)	0 (0 to 0)	28.98% (-43.62 to 204.45)	12.5 (6.6 to 22.4)	10 (5.2 to 17.9)	0.88% (-0.83 to 2.62)
Ghana	11 (6 to 20)	5 (2 to 10)	-52.3% (-80.63 to 11.55)	34.6 (16.7 to 61.7)	11.8 (5.3 to 22)	-3.32% (-3.92 to -2.71)
Global	3019 (2463 to 3596)	2064 (1658 to 2605)	-31.63% (-47.34 to -11.44)	91.5 (74.7 to 109)	50.7 (40.8 to 64.1)	-2.15% (-2.55 to -1.76)
Grenada	0 (0 to 0)	0 (0 to 0)	-61.72% (-82.25 to -15.87)	2.9 (1.6 to 4.8)	0.6 (0.3 to 1)	-5.52% (-6.23 to -4.8)
Guatemala	9 (5 to 14)	3 (2 to 6)	-64.55% (-83.13 to -22.89)	67.8 (37 to 108.2)	28.3 (15 to 51.9)	-4.5% (-5.72 to -3.27)
Guinea	27 (14 to 46)	25 (12 to 44)	-7.71% (-57.32 to 103.8)	195.7 (99.1 to 333.4)	160.1 (75.4 to 284.3)	-0.2% (-0.39 to -0.01)
Guinea-Bissau	2 (1 to 4)	1 (1 to 3)	-37.32% (-75.74 to 44.32)	105.2 (51.1 to 193.4)	52.9 (23.5 to 97.7)	-1.79% (-2.04 to -1.54)
Guyana	0 (0 to 0)	0 (0 to 0)	-59.68% (-83.2 to -6.15)	27.7 (14.4 to 48.9)	19 (9.6 to 32.9)	-0.74% (-1.34 to -0.13)
Haiti	21 (10 to 40)	45 (19 to 83)	115.69% (-15.72 to 445.22)	163.5 (75.6 to 313)	280.1 (119.6 to 519.7)	2.77% (2.35 to 3.18)

Location	Deaths in 1990 (95% UI)	Deaths in 2019 (95% UI)	Relative change (%, 95 CI)	ASMMR in 1990 (95% UI)	ASMMR in 2019 (95% UI)	EAPC (%, 95 CI)
Honduras	7 (3 to 11)	2 (1 to 3)	-75.62% (-90.67 to -43.66)	110 (57.5 to 178.5)	32.1 (13.2 to 67.5)	-4.29% (-4.49 to -4.1)
India	681 (398 to 1042)	123 (66 to 206)	-81.94% (-91.54 to -64.04)	101.2 (59.2 to 155.1)	36.9 (19.9 to 61.8)	-3.76% (-4.28 to -3.24)
Indonesia	88 (49 to 147)	12 (7 to 21)	-85.77% (-93.18 to -69.03)	77.5 (43.3 to 129.8)	8.8 (4.8 to 15)	-7.75% (-8.22 to -7.28)
Iran (Islamic Republic of)	11 (7 to 16)	1 (1 to 2)	-90.12% (-94.74 to -79.9)	17.6 (11 to 26.1)	2.5 (1.5 to 4)	-7.09% (-7.58 to -6.6)
Iraq	1 (0 to 2)	1 (0 to 1)	-35.58% (-76.73 to 70.59)	3.3 (1.4 to 6.3)	3 (1.3 to 5.8)	-0.01% (-0.72 to 0.71)
Jamaica	0 (0 to 0)	0 (0 to 0)	-82.61% (-93.41 to -56.41)	15 (7.8 to 25.3)	1.7 (0.8 to 3.1)	-10.15% (-11.67 to -8.62)
Jordan	1 (1 to 3)	0 (0 to 1)	-70.44% (-89.15 to -22.64)	31.9 (16.3 to 56.6)	5.3 (2.3 to 10.4)	-7.93% (-8.64 to -7.23)
Kazakhstan	0 (0 to 0)	0 (0 to 0)	127.41% (-3.72 to 477.75)	2.3 (1.1 to 4.2)	2.8 (1.5 to 4.6)	-0.69% (-2.1 to 0.74)
Kenya	23 (12 to 37)	33 (17 to 55)	44.59% (-25.99 to 177.22)	73.4 (40.2 to 119.5)	115.9 (61.7 to 194.5)	1.99% (1.32 to 2.66)
Kiribati	0 (0 to 0)	0 (0 to 0)	27.85% (-54 to 244.41)	73.1 (34.9 to 130.9)	110.4 (50.5 to 221.2)	1.43% (1.28 to 1.57)

Location	Deaths in 1990 (95% UI)	Deaths in 2019 (95% UI)	Relative change (%, 95 CI)	ASMMR in 1990 (95% UI)	ASMMR in 2019 (95% UI)	EAPC (%, 95 CI)
Kyrgyzstan	0 (0 to 0)	0 (0 to 0)	72.41% (-13.82 to 259.24)	8.3 (4.7 to 13.6)	11.3 (6.6 to 18.6)	1.9% (0.74 to 3.08)
Lao People's Democratic Republic	5 (3 to 9)	1 (0 to 1)	-85.75% (-94.6 to -64.77)	89.9 (46.5 to 162.4)	18.4 (7.8 to 37.1)	-5.37% (-5.79 to -4.95)
Lebanon	0 (0 to 1)	0 (0 to 0)	-87.45% (-95.45 to -63.6)	12.9 (5.8 to 25.6)	3.1 (1.3 to 6.6)	-5.33% (-5.95 to -4.71)
Lesotho	4 (2 to 7)	3 (1 to 5)	-38.09% (-77.51 to 62.64)	151.4 (74.8 to 264.3)	141.7 (56.5 to 275.7)	1.88% (0.82 to 2.95)
Liberia	11 (6 to 17)	21 (11 to 33)	93.55% (-6.29 to 304.16)	255.5 (136.5 to 408)	364.9 (192.7 to 591.4)	0.09% (-0.76 to 0.95)
Libya	1 (0 to 1)	0 (0 to 1)	-36.29% (-78.66 to 65.74)	11.2 (5.2 to 22)	8.3 (3.4 to 15.9)	-0.41% (-0.94 to 0.14)
Madagascar	23 (13 to 38)	43 (21 to 76)	92.31% (-14.1 to 298.29)	106.2 (59.6 to 176.5)	154.1 (73.3 to 268.7)	1.26% (0.92 to 1.6)
Malawi	23 (12 to 39)	17 (8 to 31)	-25.22% (-68.69 to 65.41)	117.9 (62.2 to 203.2)	110.5 (52.8 to 200.4)	-0.06% (-0.25 to 0.13)
Malaysia	7 (3 to 13)	1 (1 to 3)	-81.54% (-93.18 to -52.4)	39.3 (18.8 to 69.6)	6.3 (2.8 to 12)	-6.09% (-6.37 to -5.8)

Location	Deaths in 1990 (95% UI)	Deaths in 2019 (95% UI)	Relative change (%, 95 CI)	ASMMR in 1990 (95% UI)	ASMMR in 2019 (95% UI)	EAPC (%, 95 CI)
Maldives	0 (0 to 0)	0 (0 to 0)	-88.8% (-95.44 to -71.19)	24.2 (11.5 to 43.8)	2.8 (1.3 to 5.3)	-7.44% (-8.29 to -6.59)
Mali	90 (53 to 134)	55 (28 to 96)	-39.14% (-69.85 to 12.61)	429.9 (255.2 to 638.4)	143.7 (73.7 to 252.7)	-4.3% (-4.7 to -3.89)
Marshall Islands	0 (0 to 0)	0 (0 to 0)	-1.23% (-64.49 to 173.1)	108.7 (48.9 to 199.3)	109.5 (44.4 to 210.9)	-0.71% (-1.51 to 0.1)
Mauritania	17 (10 to 28)	5 (2 to 8)	-73.48% (-87.92 to -46.36)	390.2 (222.4 to 632.1)	85.8 (41.8 to 156.5)	-5.71% (-5.98 to -5.44)
Mexico	8 (4 to 12)	3 (2 to 4)	-64.08% (-82.75 to -26.91)	12.5 (7.3 to 20.2)	6.6 (3.7 to 10.5)	-1.97% (-2.36 to -1.58)
Micronesia (Federated States of)	0 (0 to 0)	0 (0 to 0)	-75.24% (-98.06 to -13.53)	77 (32.9 to 152.7)	53.9 (4.6 to 136.1)	-0.81% (-1.33 to -0.28)
Mongolia	2 (1 to 4)	1 (0 to 1)	-67.36% (-87.92 to -11.9)	157 (70.9 to 281.7)	27 (11.6 to 52.1)	-7.83% (-8.94 to -6.71)
Montenegro	0 (0 to 0)	0 (0 to 0)	-53.13% (-84.8 to 35.87)	0.2 (0.1 to 0.3)	0.1 (0 to 0.1)	-4.28% (-5.44 to -3.1)
Morocco	28 (14 to 49)	3 (1 to 5)	-91.04% (-96.52 to -78.75)	63.5 (31.6 to 110.6)	5.7 (2.6 to 11.3)	-7.94% (-8.26 to -7.62)
Mozambique	38 (21 to 64)	15 (7 to 28)	-60.77% (-83.75 to -10.02)	131 (71.2 to 221.2)	35 (15.5 to 66.4)	-4.08% (-4.8 to -3.37)

Location	Deaths in 1990 (95% UI)	Deaths in 2019 (95% UI)	Relative change (%, 95 CI)	ASMMR in 1990 (95% UI)	ASMMR in 2019 (95% UI)	EAPC (%, 95 CI)
Myanmar	17 (8 to 30)	11 (5 to 20)	-36.38% (-75.03 to 53.03)	30.7 (14.5 to 56)	27.4 (12.8 to 51.1)	-0.8% (-1.38 to -0.21)
Namibia	2 (1 to 3)	1 (0 to 1)	-62.87% (-86.45 to 1.78)	72.9 (32 to 128.3)	24.1 (9.4 to 50.3)	-3.81% (-4.51 to -3.1)
Nepal	33 (16 to 60)	4 (2 to 8)	-86.51% (-94.89 to -68.38)	153.7 (75.8 to 282)	73.6 (32.8 to 140.5)	-1.98% (-2.59 to -1.37)
Nicaragua	1 (0 to 2)	0 (0 to 0)	-82.66% (-93.12 to -53.41)	30.4 (15.1 to 51.5)	7.5 (3.5 to 14.5)	-5.34% (-5.64 to -5.03)
Niger	44 (23 to 75)	64 (31 to 110)	46.35% (-35.09 to 210.01)	234.2 (120.6 to 400.3)	142.3 (69.7 to 243.5)	-2.06% (-2.25 to -1.86)
Nigeria	73 (35 to 130)	90 (43 to 163)	24.64% (-45.36 to 179.06)	42.1 (20 to 75.7)	30.3 (14.5 to 54.5)	-2.02% (-2.64 to -1.39)
North Macedonia	0 (0 to 0)	0 (0 to 0)	-91.79% (-96.93 to -79.77)	8.4 (4.3 to 14.9)	0.5 (0.2 to 1)	-11.72% (-12.58 to -10.86)
Pakistan	142 (79 to 240)	92 (46 to 162)	-35.58% (-71.86 to 44.16)	97.8 (54.3 to 165)	59.8 (29.7 to 105.3)	-2% (-2.47 to -1.54)
Papua New Guinea	6 (3 to 11)	7 (4 to 14)	21.21% (-49.8 to 193.99)	103.4 (47 to 185.4)	46.4 (21.9 to 88.4)	-2.1% (-2.54 to -1.66)

Location	Deaths in 1990 (95% UI)	Deaths in 2019 (95% UI)	Relative change (%, 95 CI)	ASMMR in 1990 (95% UI)	ASMMR in 2019 (95% UI)	EAPC (%, 95 CI)
Paraguay	2 (1 to 4)	0 (0 to 1)	-84.54% (-93.72 to -64.58)	43.9 (25 to 71.1)	8.1 (3.6 to 14.8)	-5.83% (-6.55 to -5.11)
Peru	19 (10 to 33)	3 (1 to 5)	-86.26% (-94.39 to -68.29)	68.7 (36.7 to 116.5)	11.2 (5.1 to 22.1)	-7.73% (-8.48 to -6.96)
Philippines	11 (7 to 18)	6 (3 to 10)	-46.71% (-74.87 to 5.24)	14.1 (8.6 to 22.8)	7 (3.8 to 11.6)	-2.26% (-2.38 to -2.13)
Republic of Moldova	0 (0 to 0)	0 (0 to 0)	-95.54% (-98.11 to -89.29)	21.7 (11.8 to 35.7)	1.3 (0.7 to 2.4)	-11.08% (-12 to -10.16)
Russian Federation	2 (1 to 4)	0 (0 to 1)	-82.38% (-91.54 to -61.9)	11.5 (6.6 to 18.1)	0.8 (0.5 to 1.4)	-11.81% (-13.18 to -10.42)
Rwanda	68 (41 to 107)	29 (15 to 50)	-57.36% (-80.48 to -13.76)	433.7 (258.6 to 677.3)	147.9 (74 to 251.4)	-4.1% (-4.91 to -3.28)
Saint Lucia	0 (0 to 0)	0 (0 to 0)	-49.28% (-77.88 to 5.36)	6.6 (3.8 to 10.6)	3.9 (2 to 6.8)	-0.68% (-1.5 to 0.14)
Saint Vincent and the Grenadines	0 (0 to 0)	0 (0 to 0)	14.45% (-46.29 to 141.88)	2.1 (1.2 to 3.6)	1.4 (0.8 to 2.3)	-0.41% (-1.37 to 0.55)
Samoa	0 (0 to 0)	0 (0 to 0)	-73.85% (-91.35 to -23.69)	51.7 (21.7 to 105.5)	7.9 (2.9 to 16.1)	-8.26% (-9.07 to -7.44)
Senegal	18 (9 to 30)	16 (7 to 28)	-13.4% (-61.97 to 87.61)	108.2 (56.4 to 177.7)	65.7 (31.3 to 116.8)	-1.55% (-1.99 to -1.11)

Location	Deaths in 1990 (95% UI)	Deaths in 2019 (95% UI)	Relative change (%, 95 CI)	ASMMR in 1990 (95% UI)	ASMMR in 2019 (95% UI)	EAPC (%, 95 CI)
Serbia	0 (0 to 0)	0 (0 to 0)	-76.58% (-92.37 to -33.83)	2.2 (1.1 to 4.4)	0.4 (0.2 to 0.8)	-8.02% (-9.25 to -6.78)
Sierra Leone	9 (5 to 16)	14 (6 to 27)	55.85% (-32.66 to 256.48)	122.6 (62.1 to 216.2)	153.5 (71.3 to 292.8)	0.93% (0.46 to 1.41)
Solomon Islands	1 (0 to 1)	1 (1 to 2)	87.25% (-26.83 to 428.9)	126.5 (50 to 261.4)	130.6 (60.7 to 250.1)	0.61% (0.08 to 1.15)
Somalia	55 (24 to 103)	153 (73 to 273)	177.96% (22.99 to 524.66)	269.4 (117 to 506.7)	309.3 (147.9 to 553.2)	0.59% (0.39 to 0.8)
South Africa	11 (7 to 18)	4 (2 to 7)	-66.26% (-85.04 to -29.4)	36.6 (21.7 to 57.4)	10.8 (5.4 to 18.7)	-2.37% (-3.33 to -1.4)
South Sudan	7 (3 to 15)	10 (4 to 21)	34.69% (-46.11 to 216.95)	69 (29.1 to 138.3)	52.3 (20.6 to 108.2)	-1.09% (-1.35 to -0.84)
Sri Lanka	1 (0 to 2)	0 (0 to 0)	-81.02% (-93.09 to -47.56)	11.8 (5.7 to 21.4)	1.8 (0.8 to 3.5)	-6.14% (-6.38 to -5.9)
Sudan	115 (65 to 184)	42 (18 to 82)	-63.58% (-85.06 to -14.8)	300.3 (169.5 to 480.7)	89.1 (38 to 174.9)	-4.29% (-4.52 to -4.06)
Suriname	0 (0 to 0)	0 (0 to 0)	16.1% (-57.29 to 217.26)	16.8 (8 to 30.3)	11.6 (5.4 to 21.3)	-0.98% (-1.5 to -0.45)

Location	Deaths in 1990 (95% UI)	Deaths in 2019 (95% UI)	Relative change (%, 95 CI)	ASMMR in 1990 (95% UI)	ASMMR in 2019 (95% UI)	EAPC (%, 95 CI)
Syrian Arab Republic	2 (1 to 3)	0 (0 to 1)	-70.8% (-89.17 to -21.68)	9.5 (4.4 to 16.7)	4.5 (1.8 to 9.2)	-3.49% (-4.08 to -2.89)
Tajikistan	1 (0 to 1)	0 (0 to 1)	-48.55% (-78.3 to 18.09)	18.9 (9.7 to 33.7)	8.9 (4.1 to 17.3)	-3.81% (-4.81 to -2.8)
Thailand	2 (1 to 4)	2 (1 to 3)	-18.67% (-70.97 to 128.84)	10 (4.4 to 20.3)	8.8 (3.9 to 17.5)	1.18% (0.4 to 1.96)
Timor-Leste	3 (1 to 5)	1 (0 to 2)	-52.89% (-92.17 to 19.87)	160.9 (69.6 to 287.5)	87.9 (13.4 to 164.5)	-3.56% (-4.22 to -2.89)
Togo	9 (5 to 15)	10 (4 to 17)	6.79% (-52.66 to 138.56)	113.1 (58.6 to 193.2)	90.7 (41.8 to 166)	-0.39% (-0.65 to -0.13)
Tonga	0 (0 to 0)	0 (0 to 0)	-59.28% (-84.71 to 3.97)	34.8 (16.4 to 65)	17.3 (7.4 to 34)	-2.75% (-3.05 to -2.45)
Tunisia	1 (1 to 2)	0 (0 to 0)	-82.06% (-93.21 to -55.94)	16.5 (7.6 to 30)	2 (0.8 to 3.8)	-7.64% (-7.82 to -7.47)
Turkey	19 (9 to 34)	2 (1 to 3)	-90.83% (-96.32 to -76.61)	51 (23.3 to 94.2)	4.6 (2.2 to 8.6)	-8.96% (-9.69 to -8.22)
Turkmenistan	0 (0 to 0)	0 (0 to 0)	-7.02% (-59.49 to 102.14)	7.3 (3.6 to 12.3)	6.5 (3 to 12.1)	-0.75% (-1.38 to -0.12)

Location	Deaths in 1990 (95% UI)	Deaths in 2019 (95% UI)	Relative change (%, 95 CI)	ASMMR in 1990 (95% UI)	ASMMR in 2019 (95% UI)	EAPC (%, 95 CI)
Tuvalu	0 (0 to 0)	0 (0 to 0)	-83.48% (-93.91 to -55.42)	94.9 (41.6 to 186.4)	38 (16 to 77.1)	-2.55% (-2.75 to -2.35)
Uganda	15 (6 to 27)	18 (9 to 33)	24.98% (-48.32 to 219.13)	51.6 (22.4 to 94.3)	35.5 (17.6 to 65.3)	-1.35% (-1.69 to -1.01)
Ukraine	0 (0 to 0)	0 (0 to 0)	-66.67% (-84.31 to -33.41)	4.5 (2.5 to 7.8)	0.9 (0.5 to 1.7)	-8.16% (-9.34 to -6.95)
United Republic of Tanzania	45 (24 to 73)	46 (22 to 80)	2.41% (-54.23 to 136.01)	99.7 (53 to 163.9)	54.1 (26.3 to 95.3)	-1.12% (-1.53 to -0.71)
Uzbekistan	2 (1 to 3)	1 (0 to 1)	-50.14% (-75.7 to 0.93)	26.6 (15.3 to 41.5)	13.6 (7.3 to 22.9)	-0.78% (-1.64 to 0.09)
Vanuatu	0 (0 to 0)	0 (0 to 0)	40.41% (-52.2 to 300.41)	42.1 (16.4 to 86.4)	47.7 (18.7 to 97)	-0.24% (-0.54 to 0.07)
Venezuela (Bolivarian Republic of)	8 (5 to 11)	3 (2 to 5)	-62.16% (-81.99 to -23.58)	58.7 (37.9 to 86.7)	24.5 (13.2 to 41.8)	-3.68% (-4.42 to -2.93)
Viet Nam	2 (1 to 4)	0 (0 to 0)	-93.92% (-98.19 to -83.16)	6.4 (2.6 to 13.7)	0.5 (0.2 to 1)	-8.67% (-9.14 to -8.2)

Location	Deaths in 1990 (95% UI)	Deaths in 2019 (95% UI)	Relative change (%, 95 CI)	ASMMR in 1990 (95% UI)	ASMMR in 2019 (95% UI)	EAPC (%, 95 CI)
Yemen	28 (12 to 54)	36 (16 to 69)	27.97% (-50.85 to 255.88)	114.1 (48.5 to 215.6)	90.6 (41.1 to 172.1)	-1.76% (-2.12 to -1.4)
Zambia	7 (3 to 12)	4 (2 to 8)	-37.77% (-77.09 to 46.45)	46.2 (22.9 to 81.9)	20.9 (9 to 40.3)	-3.98% (-4.57 to -3.39)
Zimbabwe	10 (6 to 17)	23 (11 to 41)	122.03% (1.37 to 377.17)	79.4 (43.1 to 130.9)	197 (92.6 to 353.6)	6.16% (4.75 to 7.6)

ASMMR – age-standardized maternal mortality ratio (per 100,000 livebirths), CI – confidence interval, EAPC – estimated annual percentage change, MSMI – maternal sepsis and other maternal infections, UI – uncertain interval.

Table S23. MMR of MSMI in 131 low- and middle-income countries and territories with EAPC in age group of 45-49 years from 1990 and 2019.

Location	Deaths in 1990 (95% UI)	Deaths in 2019 (95% UI)	Relative change (%, 95 CI)	ASMMR in 1990 (95% UI)	ASMMR in 2019 (95% UI)	EAPC (%, 95 CI)
Afghanistan	23<br>(9 to 51)	25<br>(10 to 52)	7.08%<br>(-64.76 to 229.37)	312.8<br>(119.7 to 684.7)	137.2<br>(54.6 to 286.8)	-4.1%<br>(-5.33 to -2.85)
Albania	0<br>(0 to 0)	0<br>(0 to 0)	-24.93%<br>(-77.55 to 136.6)	5.5<br>(2.4 to 10.8)	11.1<br>(4.1 to 23.1)	5.39%<br>(3.62 to 7.19)
Algeria	4<br>(1 to 7)	1<br>(0 to 1)	-85.44%<br>(-94.91 to -55.46)	51.9<br>(19.8 to 104.3)	6.9<br>(2.7 to 14.7)	-6.71%<br>(-7.08 to -6.34)
American Samoa	0<br>(0 to 0)	0<br>(0 to 0)	-40.77%<br>(-81.59 to 92.5)	213.4<br>(79.9 to 453.2)	285.1<br>(110.5 to 593.4)	0.7%<br>(-0.26 to 1.67)
Angola	8<br>(3 to 18)	9<br>(4 to 21)	12.74%<br>(-63.04 to 296.2)	165.3<br>(56.8 to 361.3)	129.4<br>(50.4 to 293.5)	-0.93%<br>(-1.05 to -0.8)
Armenia	0<br>(0 to 0)	0<br>(0 to 0)	152.35%<br>(23.37 to 455.46)	15.1<br>(7.6 to 25.5)	35<br>(20.5 to 55.6)	4.43%<br>(2.68 to 6.21)
Azerbaijan	0<br>(0 to 0)	0<br>(0 to 0)	-51.02%<br>(-84.05 to 41.24)	72.3<br>(31.2 to 139)	36.3<br>(14 to 72.1)	-3.41%<br>(-4.09 to -2.74)
Bangladesh	12<br>(5 to 23)	1<br>(0 to 2)	-90.92%<br>(-96.83 to -70.83)	69.1<br>(29.3 to 136.8)	8.3<br>(3.3 to 17.7)	-8.69%<br>(-9.89 to -7.49)

Location	Deaths in 1990 (95% UI)	Deaths in 2019 (95% UI)	Relative change (%, 95 CI)	ASMMR in 1990 (95% UI)	ASMMR in 2019 (95% UI)	EAPC (%, 95 CI)
Belarus	0<br>(0 to 0)	0<br>(0 to 0)	-79.4%<br>(-90.65 to -55.82)	20.7<br>(10.5 to 35.8)	1.6<br>(0.7 to 3)	-7.99%<br>(-9.18 to -6.78)
Belize	0<br>(0 to 0)	0<br>(0 to 0)	159.83%<br>(31.81 to 459.95)	24.2<br>(13.4 to 38.8)	59.4<br>(33.3 to 98.3)	5.45%<br>(4.34 to 6.57)
Benin	10<br>(5 to 18)	7<br>(3 to 15)	-22.79%<br>(-69.35 to 98.27)	394<br>(189.4 to 734.3)	153.7<br>(63.6 to 313.6)	-2.68%<br>(-3.19 to -2.16)
Bhutan	0<br>(0 to 0)	0<br>(0 to 0)	-87.73%<br>(-96.55 to -57.78)	28.7<br>(10.1 to 66.2)	6<br>(2.2 to 13.1)	-5.41%<br>(-5.83 to -4.99)
Bolivia (Plurinational State of)	5<br>(2 to 9)	3<br>(1 to 6)	-36.22%<br>(-78.49 to 86.48)	218.5<br>(96.4 to 420.4)	144<br>(50.9 to 313.8)	-2.1%<br>(-2.37 to -1.82)
Bosnia and Herzegovina	0<br>(0 to 0)	0<br>(0 to 0)	-99.59%<br>(-99.87 to -98.79)	152.7<br>(66.1 to 292.8)	1.1<br>(0.4 to 2.5)	-19.36%<br>(-20.53 to -18.16)
Botswana	0<br>(0 to 0)	0<br>(0 to 0)	-70.46%<br>(-92.02 to -4.13)	26.2<br>(8.7 to 61.2)	17.6<br>(6 to 40.6)	-2.67%<br>(-3.29 to -2.05)
Brazil	25<br>(16 to 38)	4<br>(3 to 6)	-82.93%<br>(-91.1 to -66.98)	144.9<br>(92 to 216.5)	47.3<br>(29.4 to 70.1)	-3.29%<br>(-4.31 to -2.25)
Bulgaria	0<br>(0 to 0)	0<br>(0 to 0)	-77.49%<br>(-89.88 to -48.2)	970.4<br>(518 to 1617.1)	21<br>(10.6 to 37.6)	-13.26%<br>(-15.25 to -11.22)
Burkina Faso	11<br>(5 to 21)	7<br>(3 to 14)	-41.06%<br>(-80.52 to 51.56)	162.5<br>(70.4 to 298.5)	60.9<br>(23.2 to 124)	-4.56%<br>(-5.58 to -3.53)

Location	Deaths in 1990 (95% UI)	Deaths in 2019 (95% UI)	Relative change (%, 95 CI)	ASMMR in 1990 (95% UI)	ASMMR in 2019 (95% UI)	EAPC (%, 95 CI)
Burundi	12 (5 to 24)	16 (7 to 31)	34.22% (-50.87 to 287.72)	371 (147.1 to 756.5)	337.2 (147.3 to 674)	-0.94% (-1.32 to -0.56)
Cabo Verde	0 (0 to 0)	0 (0 to 0)	-77.08% (-92.41 to -30.44)	24.3 (9.9 to 50)	11.9 (4.5 to 26.1)	-3.78% (-4.51 to -3.05)
Cambodia	11 (4 to 25)	4 (1 to 8)	-65.52% (-89.09 to 8.68)	391.2 (145.3 to 840.8)	294.7 (110 to 629.8)	-1.28% (-2.42 to -0.11)
Cameroon	13 (6 to 24)	12 (4 to 26)	-11% (-70.85 to 138.4)	319.7 (148.4 to 571.7)	221.9 (81.1 to 483.1)	-1.18% (-1.37 to -0.99)
Central African Republic	5 (2 to 9)	11 (4 to 25)	137.56% (-20.38 to 578.23)	357.7 (141.9 to 705.9)	661.4 (239.4 to 1495.7)	2.2% (2.05 to 2.36)
Chad	13 (6 to 26)	17 (7 to 35)	27.28% (-53.93 to 249.22)	541.7 (232 to 1045.3)	276.2 (112.1 to 568.8)	-2.62% (-2.98 to -2.26)
China	19 (14 to 26)	1 (1 to 1)	-95.68% (-97.27 to -93.13)	44.2 (32.2 to 59.6)	0.6 (0.4 to 0.8)	-16.24% (-17.34 to -15.12)
Colombia	2 (1 to 3)	1 (1 to 3)	-31.06% (-68.79 to 61.74)	51.2 (28.9 to 82.7)	55.7 (28 to 100.1)	0.73% (-0.1 to 1.57)
Comoros	0 (0 to 1)	0 (0 to 1)	-22.56% (-76.14 to 229.84)	91.6 (25 to 208.3)	156.2 (57.2 to 355.8)	0.95% (0.35 to 1.55)
Congo	2 (1 to 4)	2 (1 to 5)	16.77% (-68.14 to 253.46)	176.3 (66.8 to 389.5)	258 (91.4 to 541.7)	1.62% (1.24 to 2.01)

Location	Deaths in 1990 (95% UI)	Deaths in 2019 (95% UI)	Relative change (%, 95 CI)	ASMMR in 1990 (95% UI)	ASMMR in 2019 (95% UI)	EAPC (%, 95 CI)
Costa Rica	0 (0 to 0)	0 (0 to 0)	-7.04% (-61.83 to 129.32)	16.1 (7.4 to 29.2)	25.8 (13.4 to 44.7)	2.8% (1.82 to 3.79)
Cuba	0 (0 to 0)	0 (0 to 0)	-23.58% (-66.54 to 90.36)	91.8 (42.9 to 168.9)	68.9 (37.7 to 118.1)	-1.58% (-2.16 to -1.01)
Democratic People's Republic of Korea	1 (0 to 2)	1 (0 to 2)	25.99% (-63.77 to 392.25)	37.5 (12 to 90)	345.2 (119.9 to 782.9)	8.47% (7.91 to 9.03)
Democratic Republic of the Congo	71 (29 to 138)	232 (91 to 462)	228.29% (8.77 to 808.93)	304.7 (123.4 to 595.9)	1003 (395.7 to 2000.3)	5.81% (4.94 to 6.68)
Djibouti	0 (0 to 1)	1 (0 to 1)	81.58% (-41.98 to 479.06)	108 (41.6 to 235.9)	88 (30.7 to 197.7)	-0.29% (-0.48 to -0.11)
Dominica	0 (0 to 0)	0 (0 to 0)	4.73% (-62.59 to 188.64)	46.5 (20.8 to 86.1)	277.8 (116.3 to 537.9)	9% (7.87 to 10.14)
Dominican Republic	0 (0 to 0)	0 (0 to 0)	-14.92% (-71.76 to 157.76)	30.3 (14 to 59.5)	63.8 (23 to 134.6)	3% (2.34 to 3.66)
Ecuador	1 (0 to 1)	1 (0 to 1)	-23.8% (-70.93 to 90.51)	43.1 (21.5 to 74.9)	73.7 (33.9 to 136)	3.65% (2.26 to 5.05)
Egypt	9 (4 to 16)	1 (0 to 1)	-93.32% (-97.73 to -80.46)	119.1 (56.7 to 221)	16 (6.3 to 35.7)	-8.91% (-9.98 to -7.83)
El Salvador	1 (0 to 1)	0 (0 to 0)	-92.33% (-97.88 to -75.12)	95.7 (38.7 to 194.8)	30.7 (11.2 to 68.9)	-3.25% (-4.29 to -2.2)

Location	Deaths in 1990 (95% UI)	Deaths in 2019 (95% UI)	Relative change (%, 95 CI)	ASMMR in 1990 (95% UI)	ASMMR in 2019 (95% UI)	EAPC (%, 95 CI)
Equatorial Guinea	1 (1 to 3)	0 (0 to 0)	-91.64% (-97.48 to -70.14)	541.6 (197.4 to 1140.4)	113.5 (40.1 to 271.5)	-6.45% (-7.64 to -5.24)
Eritrea	5 (2 to 11)	5 (2 to 12)	-1.44% (-70.38 to 207.69)	245.1 (90.4 to 526.8)	173.1 (64.1 to 384)	-0.11% (-0.83 to 0.62)
Eswatini	0 (0 to 1)	0 (0 to 0)	-57.97% (-88.88 to 54.37)	142.8 (50.9 to 307.2)	146.1 (43.5 to 346.3)	1.04% (-0.09 to 2.18)
Ethiopia	289 (152 to 484)	90 (47 to 157)	-68.75% (-84.65 to -35)	1048.2 (550.7 to 1754.6)	261.7 (135.2 to 453.9)	-5.31% (-5.89 to -4.73)
Fiji	0 (0 to 0)	0 (0 to 0)	-45.49% (-83.15 to 83.27)	251.3 (95.4 to 532.8)	214.5 (79 to 464.6)	-0.68% (-0.95 to -0.4)
Gabon	1 (0 to 1)	0 (0 to 1)	-49.73% (-83.02 to 43.15)	182.2 (80.4 to 352.9)	284.5 (105.3 to 631.7)	0.78% (0.23 to 1.34)
Gambia	2 (1 to 3)	3 (1 to 6)	75.86% (-34.61 to 367.4)	341.1 (147.1 to 611.6)	432.4 (182.6 to 858.4)	0.59% (0.31 to 0.88)
Georgia	0 (0 to 0)	0 (0 to 0)	49.04% (-40.68 to 246.23)	22.5 (11.1 to 39.8)	13.2 (6.4 to 23.4)	1.59% (-0.35 to 3.56)
Ghana	4 (2 to 9)	1 (0 to 2)	-77.22% (-92.09 to -35.03)	44.5 (17.9 to 94.7)	10.9 (4.1 to 22.8)	-6.16% (-7.91 to -4.37)

Location	Deaths in 1990 (95% UI)	Deaths in 2019 (95% UI)	Relative change (%, 95 CI)	ASMMR in 1990 (95% UI)	ASMMR in 2019 (95% UI)	EAPC (%, 95 CI)
Global	1419 (1170 to 1721)	985 (748 to 1300)	-30.62% (-48.96 to -7.21)	171.8 (141.6 to 208.4)	112.5 (85.4 to 148.5)	-1.53% (-1.94 to -1.12)
Grenada	0 (0 to 0)	0 (0 to 0)	-57.34% (-80.73 to -1.45)	14.9 (7.8 to 26.5)	24.4 (12.8 to 42)	3.64% (2.71 to 4.58)
Guatemala	1 (1 to 2)	1 (0 to 1)	-43.7% (-73.86 to 21.27)	47.5 (23.6 to 83.1)	43.3 (21.2 to 78.6)	-1.12% (-2.33 to 0.1)
Guinea	16 (7 to 30)	14 (6 to 29)	-16.99% (-69.51 to 121.35)	433.5 (193.4 to 804.2)	259.8 (106 to 550.1)	-1.14% (-1.36 to -0.93)
Guinea-Bissau	2 (1 to 3)	1 (0 to 2)	-44.5% (-81.97 to 57.78)	214.7 (84 to 430.1)	80.9 (27.8 to 168)	-3.03% (-3.3 to -2.75)
Guyana	0 (0 to 0)	0 (0 to 0)	-41.89% (-77.01 to 34.22)	43 (22 to 78)	54.3 (26.6 to 97.7)	2.8% (1.96 to 3.64)
Haiti	5 (2 to 10)	12 (5 to 28)	157% (-16.91 to 659.72)	198.8 (78.8 to 410.6)	535.3 (197 to 1236.1)	4.32% (3.92 to 4.72)
Honduras	1 (0 to 2)	0 (0 to 1)	-59.49% (-87.33 to 13.97)	104.1 (47 to 198.4)	64 (22 to 144.2)	-2.15% (-2.74 to -1.56)
India	248 (141 to 399)	41 (22 to 71)	-83.26% (-91.97 to -66.14)	118 (67.1 to 190.2)	40.6 (21.7 to 69.9)	-3.37% (-3.93 to -2.8)
Indonesia	36 (20 to 63)	5 (2 to 8)	-87.26% (-94.19 to -72.42)	127.3 (70.7 to 223.8)	30.6 (16.5 to 50.9)	-6.16% (-6.61 to -5.71)

Location	Deaths in 1990 (95% UI)	Deaths in 2019 (95% UI)	Relative change (%, 95 CI)	ASMMR in 1990 (95% UI)	ASMMR in 2019 (95% UI)	EAPC (%, 95 CI)
Iran (Islamic Republic of)	3 (2 to 5)	0 (0 to 1)	-90.83% (-95.2 to -83.04)	21.4 (13.8 to 31.5)	7.3 (4.3 to 11.6)	-3.29% (-3.71 to -2.86)
Iraq	1 (0 to 1)	0 (0 to 1)	-39.53% (-80.33 to 92.19)	11.2 (4.2 to 24.4)	13.9 (5.8 to 27.8)	1.41% (0.64 to 2.18)
Jamaica	0 (0 to 0)	0 (0 to 0)	-76.83% (-90.64 to -42.9)	62.1 (35.8 to 103.2)	9.2 (4.3 to 17.7)	-6.59% (-8.47 to -4.67)
Jordan	0 (0 to 1)	0 (0 to 0)	-67.87% (-89.82 to -5.02)	42.8 (16.8 to 88)	31.5 (11.8 to 68.4)	-2.69% (-3.6 to -1.77)
Kazakhstan	0 (0 to 0)	0 (0 to 0)	519.54% (152.35 to 1540.51)	7.7 (3.3 to 15)	25.9 (13.8 to 42.9)	4.32% (3.06 to 5.6)
Kenya	13 (7 to 21)	18 (9 to 30)	38.91% (-28.08 to 159.46)	156.2 (84.5 to 251.8)	409.6 (206.7 to 680.5)	4% (3.17 to 4.83)
Kiribati	0 (0 to 0)	0 (0 to 0)	-15.22% (-72.78 to 175.12)	460.2 (187.4 to 922.4)	779 (276.2 to 1667.8)	1.66% (1.48 to 1.83)
Kyrgyzstan	0 (0 to 0)	0 (0 to 0)	391.29% (135.5 to 957.93)	5.5 (3 to 9.8)	13.9 (8 to 21.8)	5.36% (4.17 to 6.57)
Lao People's Democratic Republic	1 (1 to 3)	0 (0 to 1)	-78.37% (-93.31 to -32.99)	79.4 (32.8 to 157)	25.8 (9.3 to 55.5)	-4.31% (-4.65 to -3.97)
Lebanon	0 (0 to 0)	0 (0 to 0)	-85.4% (-95.84 to -46.14)	9.2 (3.3 to 20)	3.1 (1.1 to 7.1)	-4.38% (-5.01 to -3.74)

Location	Deaths in 1990 (95% UI)	Deaths in 2019 (95% UI)	Relative change (%, 95 CI)	ASMMR in 1990 (95% UI)	ASMMR in 2019 (95% UI)	EAPC (%, 95 CI)
Lesotho	2 (1 to 4)	1 (0 to 3)	-42.75% (-81.69 to 75.66)	389.5 (162.9 to 737.3)	864.3 (306.8 to 1915)	5.04% (4.05 to 6.03)
Liberia	8 (4 to 14)	13 (6 to 25)	67.69% (-35.76 to 329.29)	671.7 (327.5 to 1198.7)	1012.7 (448.6 to 2001)	0.28% (-0.5 to 1.06)
Libya	0 (0 to 0)	0 (0 to 0)	-36.05% (-81.52 to 127.74)	7.9 (3 to 17.4)	24.5 (8.6 to 57.6)	3.67% (2.55 to 4.81)
Madagascar	12 (6 to 22)	25 (9 to 54)	111.41% (-30.26 to 426.82)	266.4 (128.6 to 486.9)	648.4 (241 to 1413.3)	3.26% (2.84 to 3.68)
Malawi	23 (12 to 41)	15 (7 to 30)	-35.07% (-75.58 to 60.06)	334.1 (167.7 to 593.7)	218.8 (98.8 to 439.9)	-1.27% (-1.61 to -0.93)
Malaysia	3 (1 to 7)	1 (0 to 2)	-73.16% (-90.99 to -12)	200 (82.1 to 432.5)	64.8 (26.9 to 139.2)	-3.9% (-4.62 to -3.17)
Maldives	0 (0 to 0)	0 (0 to 0)	-92.9% (-97.6 to -77.87)	85.4 (30.9 to 184.1)	23.9 (9.6 to 49.8)	-4.38% (-5.24 to -3.51)
Mali	53 (29 to 92)	29 (12 to 56)	-45.4% (-79.64 to 33.29)	1008.1 (552.3 to 1739)	285.5 (118.2 to 550.6)	-5.21% (-5.8 to -4.62)
Marshall Islands	0 (0 to 0)	0 (0 to 0)	19.59% (-65.46 to 287.49)	384.6 (150.9 to 828)	470.8 (164.2 to 1067.4)	0.22% (-0.39 to 0.82)

Location	Deaths in 1990 (95% UI)	Deaths in 2019 (95% UI)	Relative change (%, 95 CI)	ASMMR in 1990 (95% UI)	ASMMR in 2019 (95% UI)	EAPC (%, 95 CI)
Mauritania	12 (6 to 22)	3 (1 to 7)	-72.69% (-90.09 to -31.91)	996.8 (469.2 to 1748.7)	249.6 (104 to 483)	-5.49% (-5.97 to -5)
Mexico	2 (1 to 4)	3 (2 to 4)	19.75% (-36.32 to 134.01)	22.5 (13.3 to 35.5)	81.8 (52.5 to 123.3)	7.03% (5.61 to 8.47)
Micronesia (Federated States of)	0 (0 to 0)	0 (0 to 0)	-55.38% (-95.23 to 76.69)	122.2 (44.6 to 263.7)	166.2 (18 to 396)	1.35% (1.19 to 1.52)
Mongolia	0 (0 to 0)	0 (0 to 0)	-42.26% (-83.08 to 101.76)	37.5 (14 to 79)	46.8 (16.6 to 102.3)	-0.25% (-1.09 to 0.61)
Montenegro	0 (0 to 0)	0 (0 to 0)	-57.26% (-87.72 to 63.75)	1.1 (0.4 to 2.5)	0.4 (0.1 to 0.8)	-5.4% (-6.64 to -4.15)
Morocco	8 (4 to 14)	1 (0 to 2)	-90.56% (-96.98 to -74.41)	62.6 (30.1 to 116.1)	17.9 (6.6 to 38.5)	-4.24% (-4.97 to -3.51)
Mozambique	13 (6 to 23)	6 (2 to 12)	-55.83% (-86.62 to 16.42)	138.4 (67 to 257.5)	39 (14.2 to 83.1)	-4.24% (-5.11 to -3.35)
Myanmar	3 (1 to 5)	3 (1 to 7)	31.72% (-52.71 to 255.57)	28.8 (13.2 to 56.3)	71.3 (29.6 to 146.9)	2.64% (2.25 to 3.04)
Namibia	1 (0 to 2)	0 (0 to 1)	-60.93% (-86.38 to 16.2)	161.8 (71.1 to 296.9)	122.3 (47.1 to 269.9)	-1.11% (-1.71 to -0.51)

Location	Deaths in 1990 (95% UI)	Deaths in 2019 (95% UI)	Relative change (%, 95 CI)	ASMMR in 1990 (95% UI)	ASMMR in 2019 (95% UI)	EAPC (%, 95 CI)
Nepal	11 (4 to 24)	3 (1 to 6)	-76.79% (-92.79 to -31.09)	191.4 (75.3 to 426.1)	155.8 (55.1 to 341.3)	1.18% (0.31 to 2.06)
Nicaragua	0 (0 to 0)	0 (0 to 0)	-49.03% (-83.52 to 49.11)	19 (8.3 to 36.3)	23 (9.2 to 47.3)	0.28% (-0.07 to 0.63)
Niger	24 (11 to 46)	37 (14 to 72)	52.53% (-41.48 to 280.73)	479.1 (212.4 to 919.9)	260.8 (100.5 to 515.2)	-2.48% (-2.68 to -2.29)
Nigeria	12 (5 to 22)	17 (8 to 34)	47.62% (-37.52 to 255.18)	19.7 (8.8 to 38.1)	13.2 (5.9 to 26)	-1.97% (-2.58 to -1.36)
North Macedonia	0 (0 to 0)	0 (0 to 0)	-86.43% (-95.46 to -57.22)	19.5 (8.3 to 38)	2.2 (0.9 to 4.5)	-9.33% (-10.53 to -8.12)
Pakistan	58 (30 to 108)	29 (13 to 58)	-50.52% (-81.96 to 23.04)	109.3 (55.9 to 203)	57 (26 to 115.4)	-2.5% (-3.17 to -1.84)
Papua New Guinea	3 (1 to 6)	3 (1 to 6)	2.86% (-68.54 to 220.06)	215.1 (81.5 to 460.7)	93.6 (34 to 201)	-2.71% (-2.97 to -2.45)
Paraguay	0 (0 to 1)	0 (0 to 0)	-66.76% (-88.14 to -11.55)	66.2 (31.9 to 119.9)	112.4 (48.4 to 231.2)	1.95% (1.54 to 2.37)
Peru	8 (4 to 15)	1 (1 to 3)	-81.88% (-94.4 to -50.96)	179.1 (81.7 to 322.9)	63.6 (22.3 to 140.2)	-4.17% (-4.9 to -3.42)
Philippines	4 (3 to 7)	2 (1 to 4)	-43.1% (-73.51 to 20.14)	37.2 (21.8 to 57.3)	25.5 (13.6 to 43.4)	-1.51% (-1.74 to -1.29)

Location	Deaths in 1990 (95% UI)	Deaths in 2019 (95% UI)	Relative change (%, 95 CI)	ASMMR in 1990 (95% UI)	ASMMR in 2019 (95% UI)	EAPC (%, 95 CI)
Republic of Moldova	0<br>(0 to 0)	0<br>(0 to 0)	-95.87%<br>(-98.35 to -89.84)	91<br>(45.2 to 157.2)	4.5<br>(2.1 to 8.3)	-10.15%<br>(-11.03 to -9.26)
Russian Federation	0<br>(0 to 0)	0<br>(0 to 0)	-75.15%<br>(-88.38 to -46.52)	39.1<br>(22.5 to 60.3)	1.3<br>(0.7 to 2.1)	-12.77%<br>(-14.39 to -11.12)
Rwanda	57<br>(30 to 97)	25<br>(11 to 47)	-56.62%<br>(-84.2 to 4.69)	1334.5<br>(705.7 to 2280.6)	1084<br>(461.9 to 2062.1)	-0.44%<br>(-1.01 to 0.14)
Saint Lucia	0<br>(0 to 0)	0<br>(0 to 0)	-40.64%<br>(-76.11 to 36.06)	16.5<br>(8.8 to 27.5)	30.2<br>(13.9 to 53.5)	4.71%<br>(3.55 to 5.88)
Saint Vincent and the Grenadines	0<br>(0 to 0)	0<br>(0 to 0)	52.31%<br>(-28.02 to 213.87)	3.8<br>(2 to 6.5)	9.1<br>(5.1 to 14.8)	3.59%<br>(2.54 to 4.65)
Samoa	0<br>(0 to 0)	0<br>(0 to 0)	-63.09%<br>(-89.21 to 34.8)	96.8<br>(33.6 to 213.1)	26.6<br>(8.5 to 62.7)	-6.45%<br>(-7.29 to -5.59)
Senegal	10<br>(4 to 19)	9<br>(4 to 19)	-7.22%<br>(-64.95 to 146.4)	250.1<br>(108.8 to 465.8)	239.6<br>(104.6 to 487.5)	0.55%<br>(0.21 to 0.89)
Serbia	0<br>(0 to 0)	0<br>(0 to 0)	-41.24%<br>(-80.85 to 98.71)	3<br>(1.2 to 6.2)	3<br>(1.2 to 6.5)	-2.15%<br>(-3.72 to -0.56)
Sierra Leone	5<br>(2 to 10)	7<br>(3 to 14)	33.07%<br>(-47.93 to 259.23)	254.6<br>(109.8 to 501.6)	242.8<br>(97.7 to 493.3)	-0.1%<br>(-0.55 to 0.35)

Location	Deaths in 1990 (95% UI)	Deaths in 2019 (95% UI)	Relative change (%, 95 CI)	ASMMR in 1990 (95% UI)	ASMMR in 2019 (95% UI)	EAPC (%, 95 CI)
Solomon Islands	0 (0 to 1)	1 (0 to 1)	96.9% (-41.72 to 626.21)	277.2 (97.5 to 660.5)	416.3 (140.4 to 932.1)	1.78% (1.28 to 2.29)
Somalia	30 (11 to 64)	77 (27 to 161)	156.61% (-16.59 to 624.56)	472.4 (176.8 to 1002.6)	481.3 (168.6 to 1006.4)	0.33% (0.04 to 0.61)
South Africa	9 (5 to 14)	2 (1 to 3)	-78.39% (-91.28 to -54.1)	83.3 (47.5 to 134.4)	67.3 (32.7 to 121.2)	1.6% (-0.64 to 3.89)
South Sudan	4 (1 to 8)	5 (2 to 12)	50.38% (-54.56 to 357.81)	138.8 (48.7 to 300.9)	107.6 (35.4 to 245.2)	-1.02% (-1.25 to -0.78)
Sri Lanka	0 (0 to 0)	0 (0 to 0)	-74.31% (-92.56 to -21.94)	21 (8.6 to 44.4)	7.2 (2.7 to 15.3)	-3.18% (-3.64 to -2.72)
Sudan	45 (22 to 84)	27 (11 to 57)	-40.76% (-79.37 to 62.69)	452 (220 to 846.8)	254.2 (102.7 to 545.6)	-1.65% (-2.47 to -0.82)
Suriname	0 (0 to 0)	0 (0 to 0)	32.41% (-62.18 to 325.46)	30.4 (12.3 to 60.4)	60.9 (21.6 to 126.4)	2.63% (2.33 to 2.93)
Syrian Arab Republic	0 (0 to 1)	0 (0 to 0)	-44.66% (-84.55 to 82.26)	13.8 (5.5 to 28.6)	35.3 (13 to 78.4)	2.47% (1.33 to 3.62)
Tajikistan	0 (0 to 0)	0 (0 to 0)	-46.84% (-81.66 to 46.82)	5.1 (2.4 to 9.7)	2.9 (1.1 to 6.2)	-2.93% (-3.88 to -1.97)

Location	Deaths in 1990 (95% UI)	Deaths in 2019 (95% UI)	Relative change (%, 95 CI)	ASMMR in 1990 (95% UI)	ASMMR in 2019 (95% UI)	EAPC (%, 95 CI)
Thailand	1<br>(0 to 3)	2<br>(1 to 4)	68.57%<br>(-49.32 to 482.19)	27.1<br>(9.5 to 62.7)	169.5<br>(66.1 to 345.7)	9.74%<br>(8.05 to 11.47)
Timor-Leste	2<br>(1 to 3)	1<br>(0 to 2)	-46.12%<br>(-85.13 to 61.81)	441.8<br>(175.7 to 868.1)	246.6<br>(83.8 to 512.9)	-3.85%<br>(-4.59 to -3.11)
Togo	5<br>(2 to 9)	5<br>(2 to 11)	5.99%<br>(-64.43 to 198.13)	195.4<br>(89 to 360.3)	135.7<br>(47.9 to 287.3)	-1.22%<br>(-1.43 to -1.01)
Tonga	0<br>(0 to 0)	0<br>(0 to 0)	-62.31%<br>(-89.36 to 22.95)	217.4<br>(90.5 to 470.6)	237<br>(83.5 to 544.5)	0.13%<br>(-0.16 to 0.42)
Tunisia	0<br>(0 to 0)	0<br>(0 to 0)	-76.46%<br>(-93.47 to -23.26)	17.3<br>(6.9 to 38.1)	7.6<br>(2.6 to 18.3)	-3.43%<br>(-3.75 to -3.1)
Turkey	5<br>(2 to 11)	1<br>(0 to 1)	-88.07%<br>(-96.58 to -60.39)	66.5<br>(25.1 to 147.6)	18.7<br>(7.7 to 41.5)	-3.96%<br>(-4.23 to -3.69)
Turkmenistan	0<br>(0 to 0)	0<br>(0 to 0)	45.42%<br>(-36.05 to 225.85)	4.9<br>(2.6 to 8.5)	8<br>(3.7 to 15.9)	1.05%<br>(0.36 to 1.74)
Tuvalu	0<br>(0 to 0)	0<br>(0 to 0)	-79.21%<br>(-93.69 to -26.63)	444.9<br>(154.2 to 971)	382.6<br>(122.6 to 862.8)	-0.28%<br>(-0.54 to -0.03)
Uganda	10<br>(4 to 20)	12<br>(5 to 24)	14.6%<br>(-56.6 to 221.05)	127<br>(53.1 to 243.8)	147.5<br>(61.2 to 294.7)	1.27%<br>(0.96 to 1.59)
Ukraine	0<br>(0 to 0)	0<br>(0 to 0)	-58.48%<br>(-81.87 to -7.03)	7.3<br>(3.8 to 12.5)	1<br>(0.4 to 1.9)	-8.59%<br>(-10.02 to -7.13)

Location	Deaths in 1990 (95% UI)	Deaths in 2019 (95% UI)	Relative change (%, 95 CI)	ASMMR in 1990 (95% UI)	ASMMR in 2019 (95% UI)	EAPC (%, 95 CI)
United Republic of Tanzania	44 (22 to 78)	33 (14 to 65)	-25.43% (-71.93 to 90.79)	293.1 (147.8 to 519.6)	212.2 (93.7 to 423.1)	0.37% (-0.21 to 0.96)
Uzbekistan	0 (0 to 0)	0 (0 to 1)	121.17% (1.29 to 373.96)	20.1 (11.3 to 33.7)	103.6 (57.5 to 169.4)	7.95% (6.82 to 9.09)
Vanuatu	0 (0 to 0)	0 (0 to 0)	42.98% (-56.81 to 416.79)	106.4 (35.3 to 237.8)	155.1 (56.2 to 361.7)	0.53% (0.25 to 0.82)
Venezuela (Bolivarian Republic of)	2 (1 to 3)	1 (1 to 2)	-42.42% (-72.1 to 21.42)	93.5 (55.9 to 141.9)	106.2 (58.2 to 176.6)	-0.12% (-0.91 to 0.68)
Viet Nam	0 (0 to 1)	0 (0 to 0)	-92.63% (-97.9 to -73.18)	16.6 (5.4 to 40.3)	4.3 (1.5 to 9.4)	-4.52% (-4.87 to -4.16)
Yemen	7 (2 to 15)	6 (2 to 14)	-8.51% (-71.53 to 199.76)	98.4 (34.7 to 215.7)	56.2 (19.6 to 122.8)	-3.23% (-3.73 to -2.73)
Zambia	4 (2 to 8)	2 (1 to 4)	-53.76% (-85.05 to 27.27)	111.4 (51.3 to 205.7)	64.2 (24.7 to 132.5)	-3.25% (-3.86 to -2.63)
Zimbabwe	7 (3 to 12)	11 (4 to 21)	56.12% (-43.47 to 287.9)	228.6 (112.3 to 392.7)	684.3 (265.5 to 1355)	6.57% (5.17 to 7.99)

ASMMR – age-standardized maternal mortality ratio (per 100,000 livebirths), CI – confidence interval, EAPC – estimated annual percentage change, MSMI – maternal sepsis and other maternal infections, UI – uncertain interval.

Table S24. MMR of MSMI in 131 low- and middle-income countries and territories with EAPC in age group of 50-54 years from 1990 and 2019.

Location	Deaths in 1990 (95% UI)	Deaths in 2019 (95% UI)	Relative change (%, 95 CI)	ASMMR in 1990 (95% UI)	ASMMR in 2019 (95% UI)	EAPC (%, 95 CI)
Afghanistan	2 (1 to 5)	2 (0 to 4)	-12.54% (-78.29 to 206.99)	819.9 (274.8 to 2038.7)	318 (96 to 748.8)	-4.14% (-5.3 to -2.96)
Albania	0 (0 to 0)	0 (0 to 0)	318.87% (17.5 to 1158.37)	39.2 (19.3 to 72.9)	396.3 (143.3 to 863.9)	18.29% (11 to 26.06)
Algeria	0 (0 to 1)	0 (0 to 0)	-83.92% (-96.24 to -34.91)	139.9 (43.4 to 335.8)	22.8 (6.7 to 54)	-5.93% (-6.23 to -5.64)
American Samoa	0 (0 to 0)	0 (0 to 0)	-51.9% (-88.63 to 81.76)	628.9 (203.6 to 1555)	513.2 (178.2 to 1228)	-2.51% (-3.57 to -1.43)
Angola	0 (0 to 1)	0 (0 to 1)	23.1% (-69.39 to 436.47)	202.1 (52 to 504.4)	185.5 (57.2 to 453.8)	-0.45% (-0.72 to -0.18)
Armenia	0 (0 to 0)	0 (0 to 0)	-92.94% (-96.76 to -84.97)	13.7 (7.3 to 23.3)	1.6 (0.8 to 2.9)	-8.96% (-10.45 to -7.46)
Azerbaijan	0 (0 to 0)	0 (0 to 0)	-68.01% (-89.65 to -7.35)	47.1 (18.1 to 94.8)	26.8 (9.8 to 58.8)	-4.02% (-4.79 to -3.24)
Bangladesh	25 (11 to 46)	2 (1 to 4)	-92.52% (-97.61 to -75.98)	5325.6 (2416.6 to 9952)	502.9 (175.6 to 1149.2)	-6.2% (-8.51 to -3.83)
Belarus	0 (0 to 0)	0 (0 to 0)	-78.07% (-89.76 to -52.25)	33.9 (17.9 to 58.8)	4.6 (2.2 to 8.4)	-7.13% (-8.42 to -5.83)

Location	Deaths in 1990 (95% UI)	Deaths in 2019 (95% UI)	Relative change (%, 95 CI)	ASMMR in 1990 (95% UI)	ASMMR in 2019 (95% UI)	EAPC (%, 95 CI)
Belize	0 (0 to 0)	0 (0 to 0)	7.84% (-49 to 125.81)	62.5 (35.8 to 102.5)	66.3 (36.1 to 111.9)	0.7% (-0.25 to 1.67)
Benin	0 (0 to 1)	0 (0 to 1)	-23.13% (-82.72 to 204.46)	621.2 (193.2 to 1615.9)	234.6 (70.2 to 557.9)	-2.52% (-3.1 to -1.94)
Bhutan	0 (0 to 0)	0 (0 to 0)	-83.47% (-96.11 to -28.33)	457.7 (124.9 to 1192.4)	127.9 (38.3 to 347.9)	-4.39% (-4.68 to -4.1)
Bolivia (Plurinational State of)	0 (0 to 0)	0 (0 to 0)	-20.57% (-81.34 to 255.95)	131.7 (35.3 to 326.9)	102.9 (28.7 to 253.5)	-1.78% (-2.15 to -1.42)
Bosnia and Herzegovina	0 (0 to 0)	0 (0 to 0)	-99.59% (-99.87 to -98.67)	208.6 (86.1 to 413.9)	2 (0.8 to 4.3)	-19.31% (-20.52 to -18.08)
Botswana	0 (0 to 0)	0 (0 to 0)	-64.24% (-91.53 to 57.81)	43.8 (12 to 106.7)	39.3 (11.2 to 98.4)	-0.86% (-1.19 to -0.54)
Brazil	0 (0 to 1)	0 (0 to 0)	-58.12% (-76.14 to -23.35)	90.9 (54.9 to 138.4)	67.4 (45.2 to 96)	-1.02% (-2.01 to -0.02)
Bulgaria	0 (0 to 0)	0 (0 to 0)	-92.6% (-96.12 to -87.68)	8038.7 (6569.9 to 9899.3)	55.7 (29.5 to 89.9)	-17.52% (-20.24 to -14.72)
Burkina Faso	0 (0 to 1)	0 (0 to 0)	-35.52% (-83.6 to 187.11)	135.1 (40.1 to 315.8)	58.6 (18.6 to 141.9)	-3.74% (-4.14 to -3.33)
Burundi	1 (0 to 3)	1 (0 to 3)	15% (-72.3 to 374.3)	1193.6 (353.5 to 3028.7)	973 (280.3 to 2360)	-1.67% (-2.2 to -1.12)

Location	Deaths in 1990 (95% UI)	Deaths in 2019 (95% UI)	Relative change (%, 95 CI)	ASMMR in 1990 (95% UI)	ASMMR in 2019 (95% UI)	EAPC (%, 95 CI)
Cabo Verde	0 (0 to 0)	0 (0 to 0)	-76.44% (-94.63 to -0.75)	31.1 (10 to 74.5)	18.5 (5.7 to 46.1)	-3.34% (-4.42 to -2.25)
Cambodia	0 (0 to 1)	0 (0 to 0)	-52.34% (-88.58 to 130.59)	306.2 (87 to 794.9)	290.5 (87.9 to 727.2)	-0.55% (-1.5 to 0.41)
Cameroon	1 (0 to 1)	0 (0 to 1)	-19.38% (-81.05 to 218.29)	439 (132.6 to 1056.6)	305.7 (86.7 to 776.7)	-1.28% (-1.45 to -1.1)
Central African Republic	0 (0 to 1)	0 (0 to 1)	119.75% (-51.59 to 808.14)	530.4 (140.5 to 1456.7)	911.6 (271.4 to 2240.5)	1.95% (1.8 to 2.1)
Chad	1 (0 to 2)	1 (0 to 2)	12.96% (-71.23 to 343.56)	881.5 (268.2 to 2254.9)	445.4 (140.2 to 1094.6)	-2.64% (-3.1 to -2.19)
China	7 (5 to 10)	0 (0 to 0)	-95.27% (-97.14 to -92.22)	507.7 (356.1 to 723.8)	6.2 (4.3 to 9)	-16.03% (-16.94 to -15.12)
Colombia	0 (0 to 0)	0 (0 to 0)	-60.4% (-82.41 to -14.53)	113.3 (68.7 to 180.1)	59.1 (29.4 to 99.8)	-3.47% (-4.6 to -2.32)
Comoros	0 (0 to 0)	0 (0 to 0)	-22.59% (-81.86 to 254.65)	280.6 (64.4 to 730.5)	466.3 (140.9 to 1226.1)	0.98% (0.38 to 1.58)
Congo	0 (0 to 0)	0 (0 to 0)	0.2% (-74.89 to 344.1)	223.6 (59.9 to 562.1)	334.3 (101.9 to 809.5)	1.72% (1.33 to 2.11)
Costa Rica	0 (0 to 0)	0 (0 to 0)	-7.41% (-63.08 to 161.47)	2.2 (1 to 4.4)	3.1 (1.5 to 5.6)	0.35% (-0.98 to 1.7)

Location	Deaths in 1990 (95% UI)	Deaths in 2019 (95% UI)	Relative change (%, 95 CI)	ASMMR in 1990 (95% UI)	ASMMR in 2019 (95% UI)	EAPC (%, 95 CI)
Cuba	0<br>(0 to 0)	0<br>(0 to 0)	-41.7%<br>(-77.25 to 55.68)	45.8<br>(21 to 84.9)	19.8<br>(9.9 to 36.2)	-4.07%<br>(-5.7 to -2.42)
Democratic People's Republic of Korea	0<br>(0 to 0)	0<br>(0 to 0)	66.26%<br>(-62.21 to 597.44)	83.6<br>(22.7 to 229)	898.4<br>(239.5 to 2465.5)	9.3%<br>(8.42 to 10.19)
Democratic Republic of the Congo	3<br>(1 to 8)	10<br>(3 to 24)	233.52%<br>(-23.64 to 1305.7)	437.1<br>(129.7 to 1115.9)	1567.5<br>(497.2 to 3616.4)	5.99%<br>(5.01 to 6.97)
Djibouti	0<br>(0 to 0)	0<br>(0 to 0)	73.24%<br>(-58.77 to 654.6)	344.5<br>(91.5 to 902.4)	296.5<br>(82.9 to 782.3)	-0.26%<br>(-0.51 to 0)
Dominica	0<br>(0 to 0)	0<br>(0 to 0)	-8.97%<br>(-68.42 to 181.28)	84.3<br>(36.1 to 157.9)	370.7<br>(149.9 to 732.5)	5.88%<br>(4.68 to 7.1)
Dominican Republic	0<br>(0 to 0)	0<br>(0 to 0)	-33.34%<br>(-83.77 to 160.55)	129.5<br>(38.3 to 321.3)	202.9<br>(64.4 to 485.9)	1.97%<br>(1.37 to 2.57)
Ecuador	0<br>(0 to 0)	0<br>(0 to 0)	-59.89%<br>(-86.05 to 13.46)	28.1<br>(13.4 to 51.7)	24<br>(10.1 to 50.6)	0.24%<br>(-0.71 to 1.21)
Egypt	4<br>(1 to 8)	0<br>(0 to 0)	-95.76%<br>(-98.94 to -84.89)	1787.5<br>(672.8 to 3794.8)	140<br>(42.3 to 330.7)	-10.49%<br>(-11.46 to -9.5)
El Salvador	0<br>(0 to 0)	0<br>(0 to 0)	-91.36%<br>(-97.97 to -61.36)	93.3<br>(26.5 to 240.4)	32.5<br>(10.1 to 81.6)	-3.26%<br>(-4.09 to -2.43)
Equatorial Guinea	0<br>(0 to 0)	0<br>(0 to 0)	-92%<br>(-98.3 to -63.23)	742.3<br>(211.6 to 1956)	159.7<br>(41.4 to 413.2)	-6.49%<br>(-7.74 to -5.23)

Location	Deaths in 1990 (95% UI)	Deaths in 2019 (95% UI)	Relative change (%, 95 CI)	ASMMR in 1990 (95% UI)	ASMMR in 2019 (95% UI)	EAPC (%, 95 CI)
Eritrea	1 (0 to 2)	0 (0 to 1)	-25.15% (-81.29 to 221.71)	966.8 (261.6 to 2549.9)	520.8 (156 to 1290)	-1.19% (-2.01 to -0.37)
Eswatini	0 (0 to 0)	0 (0 to 0)	-54.06% (-88.58 to 91.66)	279.5 (81.1 to 680.6)	296.8 (83.5 to 751.1)	1.16% (0.18 to 2.16)
Ethiopia	31 (14 to 59)	9 (4 to 16)	-71.12% (-87.88 to -27.82)	4003 (1781.2 to 7652.1)	996.9 (490 to 1830.2)	-5.18% (-5.87 to -4.48)
Fiji	0 (0 to 0)	0 (0 to 0)	-36.25% (-85.39 to 218.42)	639.9 (180.5 to 1610.3)	537.3 (171.9 to 1240.1)	-0.86% (-1.61 to -0.1)
Gabon	0 (0 to 0)	0 (0 to 0)	-58.55% (-90.96 to 73.77)	253.4 (80 to 619.8)	405 (121.7 to 1001.6)	0.96% (0.44 to 1.49)
Gambia	0 (0 to 0)	0 (0 to 0)	45.33% (-65.97 to 477.82)	623.5 (193.9 to 1654.7)	674.1 (206.7 to 1638.5)	-0.34% (-0.74 to 0.06)
Georgia	0 (0 to 0)	0 (0 to 0)	4.73% (-54.47 to 141.83)	23.6 (12 to 40.6)	16.8 (8.2 to 30.6)	0.83% (-0.7 to 2.37)
Ghana	0 (0 to 0)	0 (0 to 0)	-41.42% (-87.02 to 145.69)	23.7 (7.6 to 59.1)	15 (4.2 to 37.3)	-1.52% (-2.3 to -0.73)
Global	218 (164 to 295)	94 (72 to 124)	-56.89% (-70.43 to -38.82)	912.1 (685.7 to 1232.9)	361.3 (274.7 to 475.5)	-3.22% (-3.52 to -2.92)
Grenada	0 (0 to 0)	0 (0 to 0)	-86.85% (-93.92 to -71.7)	39.9 (22 to 64.5)	18.2 (9.8 to 30.8)	-2% (-3.13 to -0.86)

Location	Deaths in 1990 (95% UI)	Deaths in 2019 (95% UI)	Relative change (%, 95 CI)	ASMMR in 1990 (95% UI)	ASMMR in 2019 (95% UI)	EAPC (%, 95 CI)
Guatemala	0 (0 to 0)	0 (0 to 0)	9.28% (-46.34 to 136.56)	49.4 (24.5 to 87.6)	95.3 (55.1 to 159.8)	1.82% (0.86 to 2.78)
Guinea	1 (0 to 2)	1 (0 to 1)	-16.74% (-80.34 to 207.36)	651.4 (199.5 to 1474.1)	414.9 (124.3 to 1060.6)	-1% (-1.23 to -0.76)
Guinea-Bissau	0 (0 to 0)	0 (0 to 0)	-49.45% (-88.39 to 96.59)	292.9 (92.4 to 746.6)	104.5 (32.1 to 270.1)	-3.35% (-3.55 to -3.15)
Guyana	0 (0 to 0)	0 (0 to 0)	-67.19% (-86.78 to -22.61)	103.9 (54.9 to 169.6)	66.5 (35.2 to 115.8)	-1.01% (-2.21 to 0.21)
Haiti	0 (0 to 1)	1 (0 to 2)	197.87% (-34.29 to 1365.84)	288 (71.7 to 760)	947.9 (271.7 to 2443.3)	4.96% (4.62 to 5.3)
Honduras	0 (0 to 0)	0 (0 to 0)	-54.51% (-90.5 to 89.22)	180.8 (58.3 to 472.6)	129.8 (36.5 to 337.9)	-1.34% (-1.82 to -0.87)
India	70 (37 to 120)	21 (11 to 38)	-69.92% (-86.56 to -31.93)	1187.6 (626.1 to 2017)	693.5 (343.8 to 1246.3)	-1.83% (-2.68 to -0.97)
Indonesia	0 (0 to 1)	0 (0 to 0)	-94.95% (-97.74 to -87.27)	54.9 (27.4 to 98.3)	5.4 (2.7 to 9.2)	-9.73% (-10.84 to -8.61)
Iran (Islamic Republic of)	1 (0 to 1)	0 (0 to 0)	-86.74% (-92.51 to -75.6)	141.9 (88.3 to 206.6)	71.3 (48.2 to 102.8)	-1.79% (-3.4 to -0.16)
Iraq	0 (0 to 0)	0 (0 to 0)	10.66% (-72.49 to 357.43)	47.2 (14.1 to 110)	118.4 (42.6 to 261.8)	4.32% (3.44 to 5.21)

Location	Deaths in 1990 (95% UI)	Deaths in 2019 (95% UI)	Relative change (%, 95 CI)	ASMMR in 1990 (95% UI)	ASMMR in 2019 (95% UI)	EAPC (%, 95 CI)
Jamaica	0<br>(0 to 0)	0<br>(0 to 0)	-98.35%<br>(-99.36 to -96.02)	105.1<br>(57.5 to 174.7)	1.1<br>(0.5 to 2)	-19.2%<br>(-21.74 to -16.57)
Jordan	0<br>(0 to 0)	0<br>(0 to 0)	-70.94%<br>(-92.62 to 23.24)	78.4<br>(24.1 to 188.9)	60.8<br>(20.1 to 149.3)	-2.98%<br>(-4.08 to -1.88)
Kazakhstan	0<br>(0 to 0)	0<br>(0 to 0)	155.21%<br>(9.74 to 612.41)	2.2<br>(1 to 4)	6.7<br>(3.6 to 11.6)	5.34%<br>(4.01 to 6.69)
Kenya	1<br>(1 to 2)	1<br>(1 to 2)	18.87%<br>(-45.05 to 158.42)	443.2<br>(241 to 801.8)	1034.3<br>(503.7 to 1810.5)	3.68%<br>(3.38 to 3.97)
Kiribati	0<br>(0 to 0)	0<br>(0 to 0)	21.18%<br>(-74.75 to 434.27)	1788.4<br>(543.2 to 4572.4)	4150<br>(1295.2 to 10410.4)	2.73%<br>(2.56 to 2.89)
Kyrgyzstan	0<br>(0 to 0)	0<br>(0 to 0)	-29.53%<br>(-73.07 to 86.08)	1.3<br>(0.6 to 2.4)	1<br>(0.5 to 1.9)	-1.98%<br>(-3.51 to -0.42)
Lao People's Democratic Republic	0<br>(0 to 0)	0<br>(0 to 0)	-81.17%<br>(-95.81 to -18.04)	125.8<br>(36.5 to 340.3)	36.7<br>(10.6 to 92.9)	-4.62%<br>(-4.99 to -4.26)
Lebanon	0<br>(0 to 0)	0<br>(0 to 0)	-83.26%<br>(-95.62 to -35.61)	20.5<br>(6.7 to 47.2)	9<br>(2.7 to 22.9)	-3.38%<br>(-4.04 to -2.72)
Lesotho	0<br>(0 to 0)	0<br>(0 to 0)	-44.54%<br>(-86.65 to 121.48)	959.9<br>(303.7 to 2298.3)	2014.1<br>(575.6 to 4990.6)	4.55%<br>(3.68 to 5.43)
Liberia	0<br>(0 to 1)	0<br>(0 to 1)	12.6%<br>(-73.48 to 321.63)	1188<br>(373.2 to 2743.6)	1755.8<br>(515.9 to 4244.6)	0.01%<br>(-0.84 to 0.88)

Location	Deaths in 1990 (95% UI)	Deaths in 2019 (95% UI)	Relative change (%, 95 CI)	ASMMR in 1990 (95% UI)	ASMMR in 2019 (95% UI)	EAPC (%, 95 CI)
Libya	0 (0 to 0)	0 (0 to 0)	-34.62% (-83.55 to 176.83)	3.8 (1.2 to 9.5)	13.1 (4.2 to 31.3)	3.4% (1.78 to 5.05)
Madagascar	1 (0 to 2)	2 (1 to 5)	106.51% (-50.77 to 731.38)	758.2 (231.9 to 1818)	2018.5 (555.7 to 5050.6)	3.59% (3.11 to 4.07)
Malawi	1 (0 to 3)	1 (0 to 2)	-36.16% (-83.36 to 144.83)	742.8 (232.9 to 1706.3)	513.3 (177.3 to 1216.1)	-1.06% (-1.21 to -0.92)
Malaysia	0 (0 to 1)	0 (0 to 0)	-75.48% (-94.18 to 0.6)	518.8 (167.5 to 1294.5)	142.1 (42.6 to 343.1)	-4.81% (-5.33 to -4.3)
Maldives	0 (0 to 0)	0 (0 to 0)	-95.37% (-98.76 to -80.38)	87.4 (26.1 to 209.2)	18.8 (6.6 to 42.9)	-5.44% (-6.45 to -4.42)
Mali	3 (1 to 7)	2 (1 to 5)	-31.4% (-82.27 to 208.28)	1907.4 (573.8 to 4359)	698.2 (210.1 to 1851.6)	-3.72% (-4.03 to -3.41)
Marshall Islands	0 (0 to 0)	0 (0 to 0)	49.65% (-62.49 to 535.23)	1054.4 (294 to 2678.1)	1531.5 (425.4 to 3955.7)	0.95% (0.41 to 1.49)
Mauritania	1 (0 to 2)	0 (0 to 0)	-78.67% (-94.81 to -15.88)	2082.8 (579.1 to 4857.5)	429.1 (119 to 1048)	-6.32% (-6.85 to -5.79)
Mexico	0 (0 to 0)	0 (0 to 0)	-71.76% (-85.66 to -43.42)	33.7 (19.7 to 53.7)	27.5 (16.5 to 42.6)	-2.39% (-4 to -0.75)
Micronesia (Federated States of)	0 (0 to 0)	0 (0 to 0)	-26.56% (-83.51 to 211.91)	299.8 (81.1 to 781.3)	621.5 (173.4 to 1641.4)	2.55% (2.36 to 2.75)

Location	Deaths in 1990 (95% UI)	Deaths in 2019 (95% UI)	Relative change (%, 95 CI)	ASMMR in 1990 (95% UI)	ASMMR in 2019 (95% UI)	EAPC (%, 95 CI)
Mongolia	0 (0 to 0)	0 (0 to 0)	-34.63% (-83.57 to 160.37)	72.2 (22.1 to 183.3)	94.9 (26.6 to 233.5)	0.39% (-0.85 to 1.66)
Montenegro	0 (0 to 0)	0 (0 to 0)	-47.95% (-82.48 to 71.59)	2 (0.7 to 4.2)	1.1 (0.5 to 2.1)	-3.71% (-4.31 to -3.11)
Morocco	0 (0 to 1)	0 (0 to 0)	-90.29% (-97.78 to -52.6)	136.2 (40.2 to 351.9)	38.6 (12 to 98.3)	-4.73% (-5.55 to -3.91)
Mozambique	1 (0 to 2)	0 (0 to 1)	-51.62% (-88.88 to 83.75)	267.9 (86 to 633.9)	93.7 (26 to 225.8)	-2.8% (-3.76 to -1.83)
Myanmar	0 (0 to 0)	0 (0 to 0)	57.65% (-57.44 to 536.46)	46.7 (12.5 to 122.1)	137.3 (45.3 to 318.1)	3.52% (2.76 to 4.28)
Namibia	0 (0 to 0)	0 (0 to 0)	-58.21% (-91.08 to 79.31)	348.4 (110.2 to 902.7)	284.4 (81.6 to 805.6)	-0.69% (-1.18 to -0.19)
Nepal	4 (1 to 9)	1 (0 to 2)	-83.19% (-95.79 to -32.63)	2522.8 (713.9 to 5955.3)	1438.6 (450.4 to 3563.7)	-1.45% (-1.79 to -1.11)
Nicaragua	0 (0 to 0)	0 (0 to 0)	-80.04% (-94.87 to -30.36)	6.4 (2.4 to 14.3)	2.9 (0.9 to 6.7)	-3.39% (-4.13 to -2.64)
Niger	1 (0 to 2)	2 (1 to 5)	96.61% (-56.89 to 674.61)	817.5 (230.1 to 1970.2)	464.3 (137.7 to 1167.4)	-2.14% (-2.48 to -1.79)
Nigeria	2 (1 to 4)	3 (1 to 5)	38.76% (-42.23 to 236.57)	109.8 (46.5 to 219.2)	66.2 (28 to 132.4)	-2.48% (-3.32 to -1.64)

Location	Deaths in 1990 (95% UI)	Deaths in 2019 (95% UI)	Relative change (%, 95 CI)	ASMMR in 1990 (95% UI)	ASMMR in 2019 (95% UI)	EAPC (%, 95 CI)
North Macedonia	0 (0 to 0)	0 (0 to 0)	-72.14% (-91.69 to -12.25)	37.4 (16.6 to 71.7)	9.1 (3.4 to 19.4)	-3.51% (-4.34 to -2.68)
Pakistan	32 (16 to 65)	12 (5 to 26)	-60.9% (-86.96 to 9.82)	2140.4 (1077.3 to 4332.1)	921.6 (401.9 to 1921.7)	-2.91% (-3.67 to -2.14)
Papua New Guinea	0 (0 to 1)	0 (0 to 1)	2.42% (-73.8 to 327.06)	970.9 (302.1 to 2431.2)	443.1 (141.6 to 1087.8)	-2.51% (-2.83 to -2.18)
Paraguay	0 (0 to 0)	0 (0 to 0)	-91.41% (-97.67 to -67.82)	60.3 (22.7 to 128.2)	24.6 (8.3 to 56.5)	-5.3% (-6.36 to -4.23)
Peru	0 (0 to 0)	0 (0 to 0)	-76.99% (-94.05 to -10.04)	90.1 (27.9 to 225.9)	38.7 (11.8 to 97.3)	-3.57% (-4.16 to -2.97)
Philippines	0 (0 to 1)	0 (0 to 0)	-60.06% (-81.32 to -21.86)	146.9 (88.7 to 229.6)	65.9 (37.9 to 108.6)	-3.18% (-4.07 to -2.29)
Republic of Moldova	0 (0 to 0)	0 (0 to 0)	-90.02% (-96.01 to -76.37)	10.9 (5.6 to 19.5)	1.8 (0.9 to 3.3)	-5.37% (-7.88 to -2.79)
Russian Federation	0 (0 to 0)	0 (0 to 0)	-69.89% (-86.05 to -35.68)	86.1 (52 to 133.5)	8.7 (4.5 to 15.3)	-8.95% (-9.99 to -7.91)
Rwanda	6 (2 to 15)	2 (1 to 5)	-65.74% (-91.33 to 34.85)	4684.3 (1644.3 to 11272.5)	3413.3 (1138.9 to 8422.5)	-1.04% (-1.48 to -0.59)

Location	Deaths in 1990 (95% UI)	Deaths in 2019 (95% UI)	Relative change (%, 95 CI)	ASMMR in 1990 (95% UI)	ASMMR in 2019 (95% UI)	EAPC (%, 95 CI)
Saint Lucia	0 (0 to 0)	0 (0 to 0)	-75.39% (-89.67 to -42.55)	39.3 (20.9 to 65.7)	26.4 (13 to 47.9)	-1.34% (-2.85 to 0.2)
Saint Vincent and the Grenadines	0 (0 to 0)	0 (0 to 0)	-22.8% (-63.15 to 55.64)	7.6 (4.1 to 12.4)	8.9 (5.1 to 14.6)	0.44% (-0.66 to 1.54)
Samoa	0 (0 to 0)	0 (0 to 0)	-59.78% (-90.23 to 74.29)	317.2 (85.9 to 862.9)	96.1 (29.7 to 241.3)	-6.05% (-6.92 to -5.18)
Senegal	0 (0 to 1)	0 (0 to 1)	-5.62% (-77.96 to 271.11)	338.1 (101.5 to 884.3)	350.3 (99.5 to 843.9)	0.89% (0.44 to 1.34)
Serbia	0 (0 to 0)	0 (0 to 0)	3.5% (-67.65 to 252.4)	1.3 (0.5 to 3)	2.7 (1.1 to 5.5)	4.48% (2.8 to 6.18)
Sierra Leone	0 (0 to 0)	0 (0 to 1)	23.13% (-67.66 to 392.58)	329.5 (104.3 to 776.2)	327.3 (106.1 to 771.6)	-0.07% (-0.6 to 0.46)
Solomon Islands	0 (0 to 0)	0 (0 to 0)	130.54% (-48.53 to 897.96)	651.8 (176.9 to 1751.7)	1301.9 (363.2 to 3260.2)	2.92% (2.35 to 3.49)
Somalia	3 (1 to 7)	6 (2 to 13)	102.48% (-50.6 to 798.63)	1584.5 (447.6 to 3753.7)	1741 (481.1 to 4195.5)	0.95% (0.63 to 1.27)
South Africa	0 (0 to 0)	0 (0 to 0)	-32.55% (-71.02 to 53.61)	84.5 (43.4 to 147.4)	214.8 (108.9 to 370.3)	7.48% (5.4 to 9.6)
South Sudan	0 (0 to 1)	1 (0 to 1)	65.04% (-62.79 to 606.59)	453.7 (122.6 to 1212.4)	379.1 (100.8 to 988.2)	-0.78% (-0.94 to -0.63)

Location	Deaths in 1990 (95% UI)	Deaths in 2019 (95% UI)	Relative change (%, 95 CI)	ASMMR in 1990 (95% UI)	ASMMR in 2019 (95% UI)	EAPC (%, 95 CI)
Sri Lanka	0 (0 to 0)	0 (0 to 0)	-62.26% (-91.11 to 80.21)	36.8 (10.6 to 95)	15.3 (5.3 to 38.1)	-2.46% (-3.12 to -1.8)
Sudan	2 (1 to 6)	3 (1 to 6)	3.86% (-73.79 to 331.41)	922.1 (296.9 to 2233.3)	916.7 (288.1 to 2269.4)	0.25% (-0.87 to 1.39)
Suriname	0 (0 to 0)	0 (0 to 0)	-8.48% (-74.02 to 252.99)	82.8 (29.1 to 181.6)	99.3 (32.4 to 238.5)	0.77% (-0.2 to 1.74)
Syrian Arab Republic	0 (0 to 0)	0 (0 to 0)	-35.23% (-81.08 to 116.08)	49.2 (17.4 to 102.9)	139.9 (53.4 to 283.6)	2.3% (1.42 to 3.19)
Tajikistan	0 (0 to 0)	0 (0 to 0)	-66.76% (-90.45 to 20.75)	12.5 (5.2 to 26)	7.5 (2.4 to 18.5)	-3.67% (-4.84 to -2.48)
Thailand	0 (0 to 0)	0 (0 to 0)	-32.95% (-83.97 to 217.27)	14.5 (4.4 to 37.6)	32.9 (10.8 to 77.2)	4.36% (3.37 to 5.35)
Timor-Leste	0 (0 to 0)	0 (0 to 0)	-49.87% (-88.58 to 134.84)	586.4 (156.4 to 1546.6)	268.6 (79 to 661.7)	-4.15% (-5.05 to -3.25)
Togo	0 (0 to 0)	0 (0 to 0)	10.88% (-72.46 to 378.4)	264.3 (72.5 to 664.6)	192.3 (56.5 to 472.7)	-0.97% (-1.18 to -0.76)
Tonga	0 (0 to 0)	0 (0 to 0)	-69.08% (-92.04 to 18.02)	715.4 (213.2 to 1751.3)	640 (194.8 to 1536.5)	-0.84% (-1.13 to -0.54)
Tunisia	0 (0 to 0)	0 (0 to 0)	-72.88% (-93.95 to 21.06)	39.6 (11.9 to 102.7)	20.1 (6.2 to 51.5)	-2.45% (-2.83 to -2.06)

Location	Deaths in 1990 (95% UI)	Deaths in 2019 (95% UI)	Relative change (%, 95 CI)	ASMMR in 1990 (95% UI)	ASMMR in 2019 (95% UI)	EAPC (%, 95 CI)
Turkey	0<br>(0 to 0)	0<br>(0 to 0)	-81.16%<br>(-95.5 to -18.56)	86<br>(23.4 to 224.2)	40<br>(14.3 to 87.2)	-2.15%<br>(-2.54 to -1.75)
Turkmenistan	0<br>(0 to 0)	0<br>(0 to 0)	-14.36%<br>(-64.41 to 89)	42.8<br>(20.2 to 81.2)	46.9<br>(22.5 to 85.3)	-0.02%<br>(-0.5 to 0.46)
Tuvalu	0<br>(0 to 0)	0<br>(0 to 0)	-71.48%<br>(-94.1 to 28.08)	1375.9<br>(413.3 to 3643.5)	1392.2<br>(395.1 to 3733.6)	0.37%<br>(0.18 to 0.56)
Uganda	1<br>(0 to 2)	1<br>(0 to 2)	21%<br>(-67.58 to 420.71)	357.7<br>(110 to 886.3)	474.5<br>(160.8 to 1089.9)	1.46%<br>(1.23 to 1.69)
Ukraine	0<br>(0 to 0)	0<br>(0 to 0)	-72.3%<br>(-86.79 to -39.27)	8.2<br>(4.3 to 14.4)	1.2<br>(0.7 to 2.2)	-8.69%<br>(-10.3 to -7.04)
United Republic of Tanzania	2<br>(1 to 6)	2<br>(1 to 5)	-10.82%<br>(-79.79 to 311.86)	544<br>(166.7 to 1266.9)	518.3<br>(157.4 to 1244)	1.39%<br>(0.73 to 2.06)
Uzbekistan	0<br>(0 to 0)	0<br>(0 to 0)	-70.92%<br>(-87.91 to -30.97)	24<br>(12.4 to 42.3)	19.4<br>(9.9 to 34.4)	-1.21%<br>(-2.45 to 0.05)
Vanuatu	0<br>(0 to 0)	0<br>(0 to 0)	55.53%<br>(-63.69 to 580.82)	268.4<br>(75 to 691.5)	390<br>(110.5 to 993.1)	0.47%<br>(0.02 to 0.91)
Venezuela (Bolivarian Republic of)	0<br>(0 to 0)	0<br>(0 to 0)	-20.67%<br>(-62.86 to 75.1)	115.9<br>(64 to 172.4)	166.1<br>(89.5 to 283.8)	0.42%<br>(-0.48 to 1.32)
Viet Nam	0<br>(0 to 0)	0<br>(0 to 0)	-93.95%<br>(-98.64 to -71.45)	12.6<br>(3.5 to 31.6)	2.8<br>(0.8 to 7.1)	-5.24%<br>(-5.37 to -5.1)

Location	Deaths in 1990 (95% UI)	Deaths in 2019 (95% UI)	Relative change (%, 95 CI)	ASMMR in 1990 (95% UI)	ASMMR in 2019 (95% UI)	EAPC (%, 95 CI)
Yemen	0<br>(0 to 1)	0<br>(0 to 1)	48.88%<br>(-65.33 to 627.39)	139<br>(38.9 to 354.5)	131.9<br>(38.2 to 334.6)	-1.5%<br>(-1.95 to -1.05)
Zambia	0<br>(0 to 1)	0<br>(0 to 0)	-50.08%<br>(-87.63 to 114.25)	222.8<br>(66.1 to 551.5)	153.8<br>(48 to 355.9)	-1.52%<br>(-2.06 to -0.99)
Zimbabwe	0<br>(0 to 1)	1<br>(0 to 2)	53.41%<br>(-63.98 to 520.54)	487.2<br>(156.2 to 1158.5)	1630.1<br>(498.5 to 3918.9)	7.61%<br>(6.08 to 9.16)

ASMMR – age-standardized maternal mortality ratio (per 100,000 livebirths), CI – confidence interval, EAPC – estimated annual percentage change, MSMI – maternal sepsis and other maternal infections, UI – uncertain interval.

Table S25. DALYs of MSMI in 131 low- and middle-income countries and territories with EAPC in age group of 10-14 years from 1990 and 2019.

Location	DALYs in 1990 (95% UI)	DALYs in 2019 (95% UI)	Relative change (%, 95 CI)	Age-standardized DALYs rate in 1990 (95% UI)	Age-standardized DALYs rate in 2019 (95% UI)	EAPC (%, 95 CI)
Afghanistan	105 (30 to 270)	103 (29 to 266)	-1.86% (-78.26 to 345.73)	4.3 (1.2 to 11)	1.2 (0.4 to 3.2)	-3.95% (-4.9 to -2.99)
Albania	0 (0 to 0)	0 (0 to 0)	-60.59% (-74.64 to -40.72)	0 (0 to 0)	0 (0 to 0)	0.36% (-0.34 to 1.06)
Algeria	276 (72 to 694)	15 (4 to 40)	-94.7% (-98.93 to -73.94)	5.3 (1.4 to 13.2)	0.3 (0.1 to 0.7)	-9.93% (-10.28 to -9.58)
American Samoa	0 (0 to 0)	0 (0 to 0)	-35.87% (-77.99 to 74.17)	0.1 (0 to 0.2)	0.1 (0 to 0.1)	-1% (-1.14 to -0.85)
Angola	37 (9 to 105)	54 (16 to 132)	47.46% (-66.99 to 615.91)	1.6 (0.4 to 4.4)	0.8 (0.2 to 1.9)	-2.32% (-2.82 to -1.82)
Armenia	0 (0 to 0)	0 (0 to 0)	-80.41% (-87.96 to -70.29)	0 (0 to 0)	0 (0 to 0)	-4.83% (-5.85 to -3.81)
Azerbaijan	0 (0 to 1)	0 (0 to 0)	-81.41% (-92.48 to -52.39)	0 (0 to 0.1)	0 (0 to 0)	-7.47% (-8.58 to -6.33)
Bangladesh	325 (107 to 719)	21 (10 to 43)	-93.59% (-97.85 to -77.29)	1.4 (0.5 to 3.1)	0.1 (0 to 0.2)	-7.93% (-9.15 to -6.7)

Location	DALYs in 1990 (95% UI)	DALYs in 2019 (95% UI)	Relative change (%, 95 CI)	Age-standardized DALYs rate in 1990 (95% UI)	Age-standardized DALYs rate in 2019 (95% UI)	EAPC (%, 95 CI)
Belarus	0 (0 to 0)	0 (0 to 0)	-81.43% (-89.59 to -66.97)	0 (0 to 0)	0 (0 to 0)	-5.19% (-5.7 to -4.69)
Belize	0 (0 to 0)	0 (0 to 0)	-37.03% (-63.86 to 17.41)	0.4 (0.3 to 0.7)	0.2 (0.1 to 0.3)	-2.92% (-3.48 to -2.36)
Benin	38 (11 to 102)	38 (11 to 95)	-0.53% (-75.33 to 356.5)	3.2 (0.9 to 8.7)	1.3 (0.4 to 3.3)	-2.2% (-2.69 to -1.7)
Bhutan	0 (0 to 1)	0 (0 to 0)	-79.8% (-94.49 to 16.65)	0.3 (0.1 to 0.8)	0.1 (0 to 0.2)	-4.79% (-5.56 to -4.02)
Bolivia (Plurinational State of)	67 (20 to 169)	14 (4 to 34)	-79.57% (-94.46 to -8.32)	5 (1.5 to 12.6)	0.7 (0.2 to 1.8)	-6.99% (-7.2 to -6.77)
Bosnia and Herzegovina	1 (0 to 2)	0 (0 to 0)	-97.35% (-99.05 to -91.43)	0.1 (0 to 0.4)	0 (0 to 0)	-11.2% (-12.68 to -9.7)
Botswana	11 (3 to 28)	3 (1 to 8)	-70.45% (-92.72 to 33.14)	3.7 (1 to 9.7)	0.9 (0.3 to 2.3)	-4.87% (-5.78 to -3.94)
Brazil	273 (181 to 392)	137 (88 to 204)	-49.73% (-71.74 to -11.77)	1.1 (0.7 to 1.5)	0.6 (0.4 to 0.9)	-1.15% (-1.54 to -0.76)
Bulgaria	1 (0 to 1)	0 (0 to 0)	-73.63% (-82.35 to -66.22)	0.1 (0 to 0.1)	0 (0 to 0.1)	-3.07% (-3.4 to -2.74)

Location	DALYs in 1990 (95% UI)	DALYs in 2019 (95% UI)	Relative change (%, 95 CI)	Age-standardized DALYs rate in 1990 (95% UI)	Age-standardized DALYs rate in 2019 (95% UI)	EAPC (%, 95 CI)
Burkina Faso	51 (15 to 129)	72 (20 to 189)	40.81% (-71.17 to 580.2)	2.2 (0.6 to 5.6)	1.4 (0.4 to 3.7)	-1.94% (-2.21 to -1.67)
Burundi	32 (8 to 86)	82 (22 to 229)	160.16% (-50.76 to 1089.55)	2.4 (0.6 to 6.5)	3 (0.8 to 8.4)	1.89% (0.39 to 3.42)
Cabo Verde	0 (0 to 1)	0 (0 to 0)	-87.1% (-95.56 to -57.86)	0.5 (0.2 to 1.2)	0.1 (0 to 0.1)	-7.91% (-8.37 to -7.45)
Cambodia	17 (4 to 45)	2 (1 to 4)	-89.96% (-97.41 to -52.91)	0.7 (0.2 to 1.9)	0.1 (0 to 0.2)	-9% (-10.23 to -7.75)
Cameroon	75 (22 to 196)	97 (27 to 245)	29.44% (-71.6 to 514.23)	3.2 (0.9 to 8.2)	1.6 (0.5 to 4.1)	-2.41% (-2.96 to -1.87)
Central African Republic	34 (8 to 93)	76 (22 to 189)	121.4% (-50.27 to 1016.52)	5.7 (1.4 to 15.4)	6.9 (2 to 16.9)	0.93% (0.68 to 1.18)
Chad	141 (36 to 362)	262 (74 to 658)	85.96% (-59.24 to 778.02)	9.7 (2.5 to 24.9)	6.4 (1.8 to 16)	-1.27% (-1.57 to -0.97)
China	600 (441 to 814)	8 (6 to 11)	-98.66% (-99.12 to -97.91)	0.4 (0.3 to 0.5)	0 (0 to 0)	-13.07% (-13.66 to -12.49)
Colombia	56 (35 to 85)	17 (11 to 27)	-69.31% (-83.48 to -42.16)	1 (0.6 to 1.5)	0.3 (0.2 to 0.5)	-3.38% (-3.77 to -2.98)

Location	DALYs in 1990 (95% UI)	DALYs in 2019 (95% UI)	Relative change (%, 95 CI)	Age-standardized DALYs rate in 1990 (95% UI)	Age-standardized DALYs rate in 2019 (95% UI)	EAPC (%, 95 CI)
Comoros	1 (0 to 1)	0 (0 to 1)	-34.81% (-84.51 to 248.65)	0.5 (0.1 to 1.4)	0.3 (0.1 to 0.7)	-2.49% (-3.1 to -1.87)
Congo	8 (2 to 22)	7 (2 to 18)	-5.04% (-79.28 to 375.24)	1.4 (0.3 to 4)	0.7 (0.2 to 1.8)	-1.87% (-2.92 to -0.8)
Costa Rica	1 (1 to 2)	0 (0 to 1)	-68.45% (-82.44 to -50.42)	0.2 (0.1 to 0.3)	0.1 (0 to 0.1)	-3.88% (-4.46 to -3.29)
Côte d'Ivoire	47 (13 to 117)	44 (15 to 116)	-6.87% (-77.7 to 336.89)	1.7 (0.5 to 4.2)	0.8 (0.3 to 2.3)	-2.1% (-2.5 to -1.7)
Cuba	1 (1 to 2)	1 (0 to 1)	-53.44% (-65.65 to -40.89)	0.1 (0.1 to 0.2)	0.1 (0 to 0.1)	-0.84% (-1.08 to -0.6)
Democratic People's Republic of Korea	18 (4 to 48)	5 (1 to 14)	-68.99% (-93.84 to 62.39)	0.5 (0.1 to 1.5)	0.2 (0.1 to 0.6)	-1.96% (-3.03 to -0.88)
Democratic Republic of the Congo	349 (84 to 939)	1145 (318 to 3027)	228.06% (-32.01 to 1550.92)	3.9 (0.9 to 10.4)	6.1 (1.7 to 16.2)	3.61% (2.14 to 5.09)
Djibouti	5 (1 to 15)	9 (2 to 23)	72.36% (-62.76 to 693.17)	5.1 (1.3 to 14.5)	4.7 (1.2 to 12.1)	-0.41% (-1.21 to 0.39)

Location	DALYs in 1990 (95% UI)	DALYs in 2019 (95% UI)	Relative change (%, 95 CI)	Age-standardized DALYs rate in 1990 (95% UI)	Age-standardized DALYs rate in 2019 (95% UI)	EAPC (%, 95 CI)
Dominica	0 (0 to 0)	0 (0 to 0)	-24.92% (-71.44 to 84.16)	1 (0.5 to 1.9)	1.3 (0.6 to 2.5)	3.03% (2.07 to 3.99)
Dominican Republic	7 (3 to 15)	4 (1 to 8)	-52.2% (-86.17 to 48.06)	0.5 (0.2 to 1.1)	0.2 (0.1 to 0.6)	-2.06% (-3.06 to -1.05)
Ecuador	20 (11 to 34)	6 (3 to 10)	-71.76% (-86.52 to -35.68)	1 (0.5 to 1.7)	0.2 (0.1 to 0.4)	-4.84% (-5.47 to -4.21)
Egypt	21 (7 to 50)	11 (4 to 28)	-49.11% (-87.09 to 89.08)	0.2 (0.1 to 0.5)	0.1 (0 to 0.2)	-3.78% (-5.36 to -2.16)
El Salvador	63 (17 to 165)	1 (1 to 3)	-97.7% (-99.41 to -90.15)	6 (1.6 to 15.8)	0.2 (0.1 to 0.4)	-10.33% (-12.17 to -8.45)
Equatorial Guinea	6 (2 to 18)	1 (0 to 3)	-85.12% (-96.76 to -16.46)	6.4 (1.5 to 17.9)	0.4 (0.1 to 1)	-10.71% (-11.24 to -10.17)
Eritrea	51 (12 to 141)	58 (16 to 150)	13.29% (-77.73 to 436.54)	7.4 (1.8 to 20.5)	4.4 (1.2 to 11.5)	-0.77% (-1.29 to -0.26)
Eswatini	2 (1 to 4)	1 (0 to 2)	-57.47% (-88.48 to 73.27)	0.8 (0.3 to 2)	0.3 (0.1 to 0.8)	-2.32% (-3.01 to -1.62)
Ethiopia	492 (188 to 1044)	270 (120 to 532)	-45.1% (-79.06 to 65.28)	4.1 (1.6 to 8.7)	1.2 (0.5 to 2.4)	-4.67% (-5.59 to -3.73)

Location	DALYs in 1990 (95% UI)	DALYs in 2019 (95% UI)	Relative change (%, 95 CI)	Age-standardized DALYs rate in 1990 (95% UI)	Age-standardized DALYs rate in 2019 (95% UI)	EAPC (%, 95 CI)
Fiji	0 (0 to 0)	0 (0 to 0)	-32.14% (-85.71 to 216.55)	0.1 (0 to 0.2)	0 (0 to 0.1)	-2.4% (-3.2 to -1.6)
Gabon	3 (1 to 9)	1 (0 to 2)	-69.46% (-92.75 to 40.14)	1.5 (0.4 to 4.3)	0.3 (0.1 to 0.8)	-5.24% (-5.88 to -4.59)
Gambia	8 (2 to 22)	8 (2 to 20)	-7.5% (-78.67 to 333.13)	3.6 (0.9 to 9.2)	1.7 (0.5 to 4.3)	-2.92% (-3.44 to -2.39)
Georgia	0 (0 to 0)	0 (0 to 0)	-60.03% (-68.89 to -49.31)	0 (0 to 0)	0 (0 to 0)	-1.41% (-2.04 to -0.79)
Ghana	18 (6 to 47)	10 (4 to 22)	-47.98% (-85.72 to 128.86)	0.6 (0.2 to 1.4)	0.2 (0.1 to 0.4)	-3.85% (-4.34 to -3.36)
Global	11776 (8951 to 15403)	6037 (4432 to 8494)	-48.73% (-65.16 to -25.37)	1.4 (1 to 1.8)	0.6 (0.5 to 0.9)	-2.38% (-2.65 to -2.11)
Grenada	0 (0 to 0)	0 (0 to 0)	-70.19% (-81.55 to -58.53)	0.1 (0.1 to 0.2)	0 (0 to 0.1)	-2.08% (-2.47 to -1.69)
Guatemala	45 (25 to 72)	6 (4 to 10)	-85.49% (-91.88 to -71.37)	2.5 (1.4 to 4)	0.2 (0.1 to 0.3)	-7.67% (-8.83 to -6.5)
Guinea	85 (21 to 221)	90 (25 to 236)	5.98% (-78.25 to 435.7)	6.1 (1.5 to 15.9)	3.2 (0.9 to 8.4)	-1.76% (-1.96 to -1.56)

Location	DALYs in 1990 (95% UI)	DALYs in 2019 (95% UI)	Relative change (%, 95 CI)	Age-standardized DALYs rate in 1990 (95% UI)	Age-standardized DALYs rate in 2019 (95% UI)	EAPC (%, 95 CI)
Guinea-Bissau	11 (3 to 28)	5 (2 to 13)	-52.71% (-88.97 to 118.46)	4.7 (1.3 to 11.9)	1.3 (0.4 to 3.2)	-4.08% (-4.77 to -3.39)
Guyana	1 (1 to 1)	0 (0 to 0)	-72.22% (-85.82 to -47.92)	0.6 (0.4 to 1)	0.2 (0.1 to 0.4)	-0.64% (-1.83 to 0.57)
Haiti	48 (11 to 130)	63 (16 to 164)	31.61% (-73.94 to 654.27)	3.5 (0.8 to 9.5)	3 (0.8 to 7.7)	-0.11% (-0.29 to 0.07)
Honduras	118 (34 to 278)	8 (3 to 19)	-93.59% (-98.34 to -68.08)	10.9 (3.2 to 25.7)	0.5 (0.2 to 1.2)	-11.26% (-11.6 to -10.91)
India	4607 (2550 to 7692)	365 (179 to 674)	-92.07% (-96.25 to -82.63)	2.9 (1.6 to 4.8)	0.2 (0.1 to 0.4)	-10.17% (-10.81 to -9.53)
Indonesia	70 (37 to 127)	8 (5 to 13)	-88.48% (-94.5 to -77.26)	0.2 (0.1 to 0.4)	0 (0 to 0)	-7.12% (-7.34 to -6.9)
Iran (Islamic Republic of)	62 (37 to 100)	6 (3 to 9)	-91.15% (-95.29 to -83.31)	0.5 (0.3 to 0.8)	0.1 (0 to 0.1)	-8.07% (-8.55 to -7.58)
Iraq	4 (2 to 9)	2 (1 to 4)	-36.83% (-75.64 to 39.38)	0.1 (0 to 0.2)	0 (0 to 0.1)	-2.95% (-3.44 to -2.46)
Jamaica	1 (0 to 1)	0 (0 to 0)	-79.12% (-87.55 to -69.34)	0.2 (0.1 to 0.3)	0.1 (0 to 0.1)	-5.4% (-6.19 to -4.6)

Location	DALYs in 1990 (95% UI)	DALYs in 2019 (95% UI)	Relative change (%, 95 CI)	Age-standardized DALYs rate in 1990 (95% UI)	Age-standardized DALYs rate in 2019 (95% UI)	EAPC (%, 95 CI)
Jordan	2 (1 to 6)	1 (0 to 3)	-49.16% (-87.83 to 122.87)	0.3 (0.1 to 0.7)	0.1 (0 to 0.2)	-6.51% (-7.21 to -5.81)
Kazakhstan	0 (0 to 0)	0 (0 to 0)	-4.18% (-40.41 to 63.84)	0 (0 to 0)	0 (0 to 0)	-0.12% (-0.41 to 0.16)
Kenya	101 (51 to 178)	56 (30 to 93)	-45.14% (-72.74 to 12.1)	1.8 (0.9 to 3.2)	0.6 (0.3 to 1)	-2.97% (-3.43 to -2.52)
Kiribati	0 (0 to 0)	0 (0 to 0)	-1.39% (-75.06 to 266.37)	0.2 (0.1 to 0.6)	0.2 (0.1 to 0.4)	-1.63% (-2.09 to -1.18)
Kyrgyzstan	0 (0 to 0)	0 (0 to 0)	10.77% (-39.31 to 111.48)	0 (0 to 0)	0 (0 to 0)	0.59% (-0.25 to 1.45)
Lao People's Democratic Republic	38 (11 to 107)	1 (0 to 3)	-96.31% (-99.07 to -83.35)	4.3 (1.2 to 11.9)	0.1 (0 to 0.3)	-11.17% (-11.72 to -10.62)
Lebanon	1 (0 to 2)	0 (0 to 0)	-82.68% (-94.32 to -44.92)	0.2 (0.1 to 0.4)	0 (0 to 0)	-6.82% (-7.03 to -6.61)
Lesotho	22 (6 to 56)	6 (2 to 14)	-73.17% (-94.88 to 31.09)	5.8 (1.5 to 14.8)	1.8 (0.5 to 4.4)	-3.04% (-3.55 to -2.52)

Location	DALYs in 1990 (95% UI)	DALYs in 2019 (95% UI)	Relative change (%, 95 CI)	Age-standardized DALYs rate in 1990 (95% UI)	Age-standardized DALYs rate in 2019 (95% UI)	EAPC (%, 95 CI)
Liberia	41 (11 to 113)	40 (11 to 103)	-2.34% (-78.73 to 372.48)	9.1 (2.4 to 25.2)	4.4 (1.2 to 11.3)	-4.29% (-5.24 to -3.33)
Libya	1 (0 to 2)	0 (0 to 0)	-84.84% (-94.72 to -54.69)	0.1 (0 to 0.2)	0 (0 to 0)	-5.5% (-6.28 to -4.71)
Madagascar	121 (32 to 298)	107 (29 to 277)	-11.15% (-82.36 to 305.2)	4.4 (1.2 to 10.9)	2 (0.5 to 5.1)	-2.11% (-2.53 to -1.7)
Malawi	44 (12 to 126)	46 (14 to 114)	4.56% (-73.5 to 357.97)	2 (0.5 to 5.7)	1.2 (0.3 to 2.9)	-1.74% (-2.16 to -1.31)
Malaysia	3 (1 to 8)	1 (0 to 1)	-78.29% (-92.68 to -30.65)	0.1 (0 to 0.3)	0 (0 to 0)	-6.15% (-6.7 to -5.59)
Maldives	0 (0 to 0)	0 (0 to 0)	-92.68% (-97.58 to -75.6)	0.1 (0 to 0.3)	0 (0 to 0)	-8.49% (-9.32 to -7.65)
Mali	323 (91 to 823)	318 (81 to 849)	-1.5% (-80.54 to 363.1)	15.9 (4.5 to 40.6)	6.3 (1.6 to 16.7)	-3.32% (-3.58 to -3.07)
Marshall Islands	0 (0 to 0)	0 (0 to 0)	-50.89% (-83.22 to 59.94)	0.2 (0.1 to 0.4)	0.1 (0 to 0.2)	-2.21% (-2.73 to -1.69)
Mauritania	30 (8 to 79)	9 (3 to 26)	-69.15% (-93.28 to 68.03)	6.6 (1.8 to 17.1)	1.2 (0.3 to 3.2)	-6.71% (-7.12 to -6.3)

Location	DALYs in 1990 (95% UI)	DALYs in 2019 (95% UI)	Relative change (%, 95 CI)	Age-standardized DALYs rate in 1990 (95% UI)	Age-standardized DALYs rate in 2019 (95% UI)	EAPC (%, 95 CI)
Mexico	64<br>(42 to 93)	22<br>(13 to 31)	-66.15%<br>(-80.41 to -40.36)	0.4<br>(0.3 to 0.6)	0.1<br>(0.1 to 0.2)	-3%<br>(-3.96 to -2.03)
Micronesia (Federated States of)	0<br>(0 to 0)	0<br>(0 to 0)	-70.48%<br>(-89.38 to -18.23)	0.3<br>(0.1 to 0.7)	0.1<br>(0.1 to 0.3)	-2.93%<br>(-3.05 to -2.82)
Mongolia	1<br>(0 to 1)	0<br>(0 to 0)	-78.46%<br>(-92.26 to -31.9)	0.1<br>(0 to 0.3)	0<br>(0 to 0)	-6.78%<br>(-7.29 to -6.26)
Montenegro	0<br>(0 to 0)	0<br>(0 to 0)	-59.23%<br>(-64.71 to -53.57)	0<br>(0 to 0)	0<br>(0 to 0)	-2.12%<br>(-2.31 to -1.92)
Morocco	57<br>(13 to 154)	5<br>(2 to 13)	-90.75%<br>(-97.8 to -53.46)	1.2<br>(0.3 to 3.2)	0.1<br>(0 to 0.3)	-7.95%<br>(-8.1 to -7.79)
Mozambique	107<br>(26 to 294)	53<br>(17 to 137)	-50.65%<br>(-88.8 to 125.56)	3.5<br>(0.9 to 9.7)	0.8<br>(0.2 to 2)	-4.42%<br>(-5 to -3.85)
Myanmar	68<br>(18 to 175)	17<br>(6 to 42)	-75.44%<br>(-94.07 to 19.5)	0.9<br>(0.2 to 2.3)	0.2<br>(0.1 to 0.6)	-4.87%<br>(-5.46 to -4.28)
Namibia	6<br>(1 to 17)	2<br>(1 to 6)	-61.78%<br>(-90.58 to 79.27)	2.1<br>(0.5 to 5.7)	0.6<br>(0.2 to 1.4)	-3.28%<br>(-3.85 to -2.7)
Nepal	65<br>(19 to 166)	4<br>(2 to 8)	-94.31%<br>(-98.35 to -78.21)	1.6<br>(0.5 to 4)	0.1<br>(0 to 0.2)	-9.94%<br>(-10.22 to -9.67)

Location	DALYs in 1990 (95% UI)	DALYs in 2019 (95% UI)	Relative change (%, 95 CI)	Age-standardized DALYs rate in 1990 (95% UI)	Age-standardized DALYs rate in 2019 (95% UI)	EAPC (%, 95 CI)
Nicaragua	20 (8 to 43)	2 (1 to 4)	-90.19% (-96.58 to -68.5)	2.2 (0.9 to 4.8)	0.2 (0.1 to 0.4)	-8.73% (-9.06 to -8.4)
Niger	158 (42 to 418)	294 (76 to 831)	86.11% (-59.71 to 850.31)	7.8 (2.1 to 20.7)	5 (1.3 to 14)	-1.59% (-1.82 to -1.35)
Nigeria	465 (224 to 824)	579 (264 to 1121)	24.44% (-50.03 to 195.68)	2.3 (1.1 to 4)	1.2 (0.6 to 2.4)	-2.91% (-3.59 to -2.22)
North Macedonia	0 (0 to 0)	0 (0 to 0)	-74.79% (-84.45 to -63.11)	0 (0 to 0.1)	0 (0 to 0)	-3.95% (-4.22 to -3.67)
Pakistan	385 (138 to 905)	240 (104 to 484)	-37.8% (-79.06 to 97)	1.6 (0.6 to 3.7)	0.6 (0.2 to 1.2)	-3.34% (-3.47 to -3.2)
Papua New Guinea	19 (5 to 55)	14 (4 to 36)	-28.26% (-85.57 to 247.3)	2.4 (0.7 to 6.8)	0.8 (0.2 to 2)	-3.21% (-3.76 to -2.66)
Paraguay	10 (4 to 21)	1 (1 to 3)	-85.2% (-95.67 to -52.37)	1.2 (0.5 to 2.5)	0.2 (0.1 to 0.3)	-7.58% (-8.63 to -6.51)
Peru	138 (38 to 364)	12 (4 to 30)	-91.46% (-97.92 to -61.04)	3.3 (0.9 to 8.8)	0.3 (0.1 to 0.7)	-8.49% (-8.84 to -8.14)
Philippines	17 (9 to 27)	5 (3 to 8)	-67.2% (-83 to -37.93)	0.1 (0.1 to 0.2)	0 (0 to 0)	-3.74% (-4.4 to -3.07)

Location	DALYs in 1990 (95% UI)	DALYs in 2019 (95% UI)	Relative change (%, 95 CI)	Age-standardized DALYs rate in 1990 (95% UI)	Age-standardized DALYs rate in 2019 (95% UI)	EAPC (%, 95 CI)
Republic of Moldova	0 (0 to 0)	0 (0 to 0)	-87.91% (-93.2 to -78.28)	0 (0 to 0)	0 (0 to 0)	-5.66% (-6.22 to -5.09)
Russian Federation	10 (6 to 15)	1 (1 to 2)	-87.6% (-93.25 to -77.16)	0.1 (0 to 0.1)	0 (0 to 0)	-7.81% (-8.61 to -7.01)
Rwanda	76 (23 to 198)	28 (9 to 73)	-62.71% (-91.06 to 64.09)	4.5 (1.3 to 11.6)	1.2 (0.4 to 3)	-4.51% (-5.71 to -3.3)
Saint Lucia	0 (0 to 0)	0 (0 to 0)	-65.67% (-77.42 to -48.11)	0.1 (0.1 to 0.2)	0.1 (0 to 0.1)	-1.32% (-1.79 to -0.85)
Saint Vincent and the Grenadines	0 (0 to 0)	0 (0 to 0)	-54.99% (-64.62 to -43.55)	0.1 (0.1 to 0.2)	0.1 (0 to 0.1)	-0.64% (-0.92 to -0.36)
Samoa	0 (0 to 0)	0 (0 to 0)	-78.07% (-93.01 to -11.53)	0.1 (0 to 0.4)	0 (0 to 0.1)	-6.51% (-7.1 to -5.93)
Senegal	60 (17 to 160)	39 (11 to 105)	-35.58% (-87.59 to 177.42)	3.3 (0.9 to 8.7)	1.3 (0.4 to 3.6)	-2.75% (-3.03 to -2.46)
Serbia	0 (0 to 1)	0 (0 to 0)	-68.25% (-85.35 to -38.04)	0 (0 to 0.1)	0 (0 to 0)	-2.38% (-2.76 to -1.99)
Sierra Leone	44 (12 to 120)	71 (19 to 179)	59.17% (-62.35 to 628.38)	5.5 (1.5 to 15)	4.2 (1.1 to 10.7)	-1.24% (-1.95 to -0.54)

Location	DALYs in 1990 (95% UI)	DALYs in 2019 (95% UI)	Relative change (%, 95 CI)	Age-standardized DALYs rate in 1990 (95% UI)	Age-standardized DALYs rate in 2019 (95% UI)	EAPC (%, 95 CI)
Solomon Islands	0 (0 to 1)	0 (0 to 1)	30.18% (-71.71 to 438.12)	0.4 (0.1 to 1)	0.3 (0.1 to 0.8)	-0.32% (-1.02 to 0.37)
Somalia	51 (12 to 145)	243 (63 to 617)	374.7% (-11.2 to 2342.61)	3.2 (0.7 to 9)	5.3 (1.4 to 13.4)	2.11% (1.83 to 2.4)
South Africa	43 (23 to 76)	4 (2 to 7)	-90.62% (-96.08 to -78.29)	0.7 (0.3 to 1.1)	0.1 (0 to 0.1)	-6.66% (-9.42 to -3.82)
South Sudan	11 (3 to 29)	20 (5 to 51)	79.33% (-55.27 to 652.13)	0.9 (0.3 to 2.3)	1 (0.3 to 2.5)	-0.19% (-0.59 to 0.21)
Sri Lanka	2 (1 to 5)	0 (0 to 1)	-84.63% (-95.39 to -41.53)	0.1 (0 to 0.2)	0 (0 to 0)	-6.15% (-6.64 to -5.66)
Sudan	172 (46 to 456)	89 (24 to 234)	-48.34% (-89.73 to 162.52)	3.9 (1 to 10.4)	1.2 (0.3 to 3.1)	-3.89% (-4.39 to -3.39)
Suriname	0 (0 to 1)	0 (0 to 0)	-25.51% (-69.8 to 88.11)	0.4 (0.2 to 1)	0.3 (0.1 to 0.6)	-0.2% (-0.86 to 0.46)
Syrian Arab Republic	9 (4 to 18)	1 (1 to 2)	-88.95% (-95.62 to -72.82)	0.3 (0.1 to 0.6)	0.1 (0 to 0.1)	-7.79% (-9 to -6.56)

Location	DALYs in 1990 (95% UI)	DALYs in 2019 (95% UI)	Relative change (%, 95 CI)	Age-standardized DALYs rate in 1990 (95% UI)	Age-standardized DALYs rate in 2019 (95% UI)	EAPC (%, 95 CI)
Tajikistan	0 (0 to 0)	0 (0 to 0)	-11.12% (-57.55 to 80.39)	0 (0 to 0)	0 (0 to 0)	-2.88% (-3.66 to -2.09)
Thailand	3 (1 to 5)	1 (0 to 2)	-64.9% (-83.84 to -36.07)	0 (0 to 0.1)	0 (0 to 0)	-1.22% (-1.58 to -0.85)
Timor-Leste	3 (1 to 8)	1 (0 to 2)	-72.45% (-94.26 to 45.29)	1.8 (0.4 to 5.1)	0.3 (0.1 to 0.9)	-7.3% (-8.27 to -6.33)
Togo	20 (6 to 50)	13 (4 to 32)	-32.87% (-84.02 to 212.79)	2.2 (0.6 to 5.7)	0.9 (0.3 to 2.1)	-3.12% (-3.57 to -2.67)
Tonga	0 (0 to 0)	0 (0 to 0)	-55.79% (-88.23 to 46.1)	0.1 (0 to 0.3)	0.1 (0 to 0.1)	-3.19% (-4 to -2.38)
Tunisia	10 (3 to 25)	1 (0 to 2)	-92.1% (-97.58 to -71.62)	0.6 (0.2 to 1.6)	0.1 (0 to 0.1)	-7.47% (-7.85 to -7.1)
Turkey	12 (4 to 30)	2 (1 to 3)	-86.39% (-95.39 to -51.67)	0.1 (0 to 0.3)	0 (0 to 0)	-5.93% (-6.47 to -5.38)
Turkmenistan	0 (0 to 0)	0 (0 to 0)	-28.78% (-53.17 to 7.41)	0 (0 to 0)	0 (0 to 0)	-1.46% (-2.11 to -0.81)
Tuvalu	0 (0 to 0)	0 (0 to 0)	-76.77% (-92.79 to -8.57)	0.3 (0.1 to 0.7)	0.1 (0 to 0.1)	-4.76% (-4.87 to -4.64)

Location	DALYs in 1990 (95% UI)	DALYs in 2019 (95% UI)	Relative change (%, 95 CI)	Age-standardized DALYs rate in 1990 (95% UI)	Age-standardized DALYs rate in 2019 (95% UI)	EAPC (%, 95 CI)
Uganda	55 (14 to 147)	96 (31 to 233)	72.58% (-57.47 to 714.2)	1.3 (0.3 to 3.5)	1 (0.3 to 2.5)	-0.53% (-1.26 to 0.2)
Ukraine	0 (0 to 1)	0 (0 to 1)	-20.27% (-52.24 to 38.19)	0 (0 to 0)	0 (0 to 0)	0.79% (0.16 to 1.43)
United Republic of Tanzania	129 (38 to 324)	152 (43 to 384)	18.14% (-74.59 to 392.32)	2.1 (0.6 to 5.4)	1.2 (0.4 to 3.1)	-0.14% (-0.82 to 0.55)
Uzbekistan	1 (0 to 1)	0 (0 to 1)	-58.17% (-78.25 to -20.27)	0 (0 to 0)	0 (0 to 0)	-3.29% (-4.05 to -2.52)
Vanuatu	0 (0 to 0)	0 (0 to 0)	56.25% (-55.97 to 480.08)	0.2 (0 to 0.4)	0.1 (0.1 to 0.4)	-0.96% (-1.37 to -0.55)
Venezuela (Bolivarian Republic of)	56 (36 to 79)	20 (13 to 31)	-63.5% (-79.63 to -32.46)	1.6 (1 to 2.3)	0.6 (0.4 to 0.9)	-3.3% (-3.74 to -2.84)
Viet Nam	8 (3 to 22)	1 (1 to 2)	-86.12% (-95.34 to -63.34)	0.1 (0 to 0.2)	0 (0 to 0)	-6.16% (-6.37 to -5.95)
Yemen	186 (43 to 494)	135 (34 to 370)	-27.61% (-85.64 to 270.15)	5.4 (1.2 to 14.3)	2.1 (0.5 to 5.9)	-4.59% (-5.05 to -4.13)

Location	DALYs in 1990 (95% UI)	DALYs in 2019 (95% UI)	Relative change (%, 95 CI)	Age-standardized DALYs rate in 1990 (95% UI)	Age-standardized DALYs rate in 2019 (95% UI)	EAPC (%, 95 CI)
Zambia	25 (8 to 69)	17 (6 to 41)	-31.16% (-83.42 to 209.01)	1.3 (0.4 to 3.6)	0.4 (0.2 to 1.1)	-3.39% (-4.11 to -2.68)
Zimbabwe	61 (17 to 152)	86 (24 to 221)	41.89% (-66.24 to 616.12)	2.5 (0.7 to 6.3)	2.9 (0.8 to 7.4)	3.81% (1.78 to 5.88)

CI – confidence interval, DALYs – disability-adjusted life years (per 100,000 population), EAPC – estimated annual percentage change, MSMI – maternal sepsis and other maternal infections, UI – uncertain interval.

Table S26. DALYs of MSMI in 131 low- and middle-income countries and territories with EAPC in age group of 15-19 years from 1990 and 2019.

Location	DALYs in 1990 (95% UI)	DALYs in 2019 (95% UI)	Relative change (%, 95 CI)	Age-standardized DALYs rate in 1990 (95% UI)	Age-standardized DALYs rate in 2019 (95% UI)	EAPC (%, 95 CI)
Afghanistan	2599 (1344 to 4543)	2689 (1375 to 4686)	3.48% (-53.7 to 134.51)	372.5 (192.7 to 651.3)	127.6 (65.2 to 222.3)	-4.02% (-5 to -3.03)
Albania	8 (3 to 14)	3 (1 to 7)	-54.56% (-79.24 to -12.05)	4.7 (1.9 to 8.6)	3.7 (1.3 to 7.3)	-1.14% (-1.49 to -0.78)
Algeria	3272 (1614 to 5469)	235 (122 to 412)	-92.83% (-96.85 to -83.39)	238.4 (117.6 to 398.6)	15.8 (8.2 to 27.7)	-9.18% (-9.5 to -8.86)
American Samoa	1 (0 to 1)	0 (0 to 1)	-41.71% (-75.06 to 43.68)	24.7 (12.9 to 43)	11.5 (5.6 to 21.9)	-2.91% (-3.18 to -2.64)
Angola	1017 (470 to 1825)	1158 (596 to 2055)	13.83% (-51.93 to 155.76)	195.2 (90.1 to 350.1)	74 (38.1 to 131.2)	-2.98% (-3.37 to -2.58)
Armenia	19 (10 to 33)	3 (1 to 6)	-82.84% (-90.32 to -72.25)	14 (7.2 to 23.9)	4.3 (1.7 to 8.1)	-4.22% (-4.9 to -3.54)
Azerbaijan	45 (26 to 75)	28 (11 to 52)	-39% (-69.19 to -3.35)	13.5 (7.8 to 22.3)	8.8 (3.5 to 16.6)	-1.96% (-2.23 to -1.69)
Bangladesh	7617 (4520 to 12268)	683 (337 to 1213)	-91.04% (-95.83 to -82.51)	128.7 (76.4 to 207.3)	8.6 (4.3 to 15.3)	-8.96% (-9.99 to -7.92)

Location	DALYs in 1990 (95% UI)	DALYs in 2019 (95% UI)	Relative change (%, 95 CI)	Age-standardized DALYs rate in 1990 (95% UI)	Age-standardized DALYs rate in 2019 (95% UI)	EAPC (%, 95 CI)
Belarus	27 (13 to 47)	6 (2 to 11)	-79.64% (-92.11 to -60.57)	7.5 (3.7 to 12.9)	2.6 (0.8 to 5.3)	-3.77% (-4.02 to -3.51)
Belize	4 (3 to 6)	5 (3 to 8)	36.75% (-18.13 to 138.75)	38.6 (24.4 to 57.2)	24.3 (14.9 to 36.3)	-0.24% (-0.65 to 0.17)
Benin	689 (382 to 1188)	644 (307 to 1128)	-6.6% (-58.54 to 95.44)	299.6 (165.9 to 516.4)	94 (44.8 to 164.7)	-3.34% (-3.73 to -2.96)
Bhutan	28 (14 to 48)	3 (1 to 4)	-90.91% (-95.83 to -80.55)	84.6 (41.2 to 142.7)	7.4 (4 to 12.8)	-8.38% (-8.59 to -8.16)
Bolivia (Plurinational State of)	800 (444 to 1362)	248 (126 to 430)	-69.01% (-86.25 to -33.43)	241.4 (134 to 410.6)	46.9 (23.8 to 81.3)	-6.19% (-6.42 to -5.95)
Bosnia and Herzegovina	21 (12 to 33)	1 (0 to 3)	-93.01% (-98.61 to -84.77)	11.2 (6.4 to 17.1)	1.6 (0.3 to 3.8)	-7.59% (-8.73 to -6.43)
Botswana	146 (75 to 258)	50 (24 to 89)	-65.75% (-85.1 to -18.01)	186.2 (96.6 to 330.3)	47 (23.1 to 83.8)	-5.02% (-5.7 to -4.34)
Brazil	4388 (2927 to 6174)	2032 (1349 to 2884)	-53.68% (-72.36 to -21.06)	57 (38 to 80.2)	25.1 (16.6 to 35.6)	-1.57% (-2 to -1.12)
Bulgaria	47 (27 to 76)	9 (4 to 17)	-80.7% (-89.37 to -69.97)	15.5 (8.7 to 25)	6 (2.5 to 11.1)	-2.91% (-3.29 to -2.51)

Location	DALYs in 1990 (95% UI)	DALYs in 2019 (95% UI)	Relative change (%, 95 CI)	Age-standardized DALYs rate in 1990 (95% UI)	Age-standardized DALYs rate in 2019 (95% UI)	EAPC (%, 95 CI)
Burkina Faso	1477 (792 to 2532)	1507 (710 to 2684)	2.08% (-55.73 to 127.97)	307 (164.7 to 526.6)	125.4 (59.1 to 223.3)	-3.65% (-4.12 to -3.18)
Burundi	794 (371 to 1440)	1479 (704 to 2469)	86.24% (-23.02 to 318.48)	291.3 (136.1 to 528.4)	225.9 (107.6 to 377.3)	-0.59% (-1.12 to -0.06)
Cabo Verde	14 (7 to 24)	1 (1 to 2)	-91.68% (-96.51 to -81.55)	74.7 (39.4 to 127.1)	4.7 (2.4 to 8.1)	-10.01% (-10.33 to -9.7)
Cambodia	799 (365 to 1501)	122 (66 to 205)	-84.69% (-93.35 to -61.87)	154.9 (70.7 to 291)	16.9 (9.2 to 28.3)	-8.73% (-9.54 to -7.91)
Cameroon	1494 (849 to 2444)	1771 (837 to 3307)	18.5% (-47.03 to 153.51)	277 (157.3 to 453.1)	110 (52 to 205.4)	-3.15% (-3.6 to -2.7)
Central African Republic	706 (363 to 1221)	1561 (789 to 2714)	121.23% (3.58 to 405.02)	506.4 (260.6 to 876.2)	526.3 (265.8 to 914.7)	0.27% (0.13 to 0.42)
Chad	2360 (1350 to 3779)	3507 (1904 to 5759)	48.61% (-22.79 to 188.42)	763.7 (437.1 to 1223.1)	387.3 (210.2 to 636)	-2.32% (-2.72 to -1.91)
China	12194 (9679 to 15620)	273 (150 to 460)	-97.76% (-98.77 to -96.21)	19.8 (15.7 to 25.3)	0.8 (0.4 to 1.3)	-10.13% (-10.85 to -9.41)

Location	DALYs in 1990 (95% UI)	DALYs in 2019 (95% UI)	Relative change (%, 95 CI)	Age-standardized DALYs rate in 1990 (95% UI)	Age-standardized DALYs rate in 2019 (95% UI)	EAPC (%, 95 CI)
Colombia	1000 (616 to 1544)	445 (269 to 689)	-55.53% (-74.88 to -18)	57.8 (35.6 to 89.2)	22.8 (13.8 to 35.4)	-3.17% (-3.63 to -2.69)
Comoros	17 (3 to 39)	8 (3 to 17)	-49.36% (-83.51 to 160.86)	64.1 (12.6 to 152.1)	23.4 (8 to 49)	-4.82% (-6.07 to -3.55)
Congo	255 (108 to 454)	198 (102 to 352)	-22.46% (-66.01 to 101.66)	184.2 (78.3 to 328)	76.3 (39.2 to 135.8)	-2.82% (-3.61 to -2.02)
Costa Rica	28 (18 to 43)	11 (5 to 20)	-60.99% (-79.85 to -35.2)	19.5 (12.1 to 29.6)	6.1 (2.7 to 11.1)	-4.35% (-4.95 to -3.76)
Côte d'Ivoire	1040 (541 to 1758)	982 (463 to 1734)	-5.61% (-58.73 to 110.42)	169.6 (88.2 to 286.6)	77 (36.3 to 136.1)	-2.38% (-2.83 to -1.92)
Cuba	96 (58 to 148)	25 (14 to 42)	-74.06% (-85.22 to -56.81)	17.3 (10.4 to 26.6)	7.5 (4.2 to 12.8)	-2.72% (-3.31 to -2.11)
Democratic People's Republic of Korea	386 (158 to 738)	196 (90 to 369)	-49.06% (-80.55 to 37.61)	37.9 (15.5 to 72.5)	22.2 (10.1 to 41.7)	-1.18% (-2.12 to -0.23)
Democratic Republic of the Congo	7959 (3797 to 13925)	19574 (10596 to 30638)	145.94% (23.17 to 406.68)	407.3 (194.3 to 712.7)	411 (222.5 to 643.3)	1.66% (0.41 to 2.92)

Location	DALYs in 1990 (95% UI)	DALYs in 2019 (95% UI)	Relative change (%, 95 CI)	Age-standardized DALYs rate in 1990 (95% UI)	Age-standardized DALYs rate in 2019 (95% UI)	EAPC (%, 95 CI)
Djibouti	99 (48 to 164)	115 (48 to 212)	15.94% (-56.43 to 162.87)	408.3 (197.4 to 675.1)	252.8 (106 to 464.4)	-1.31% (-2.04 to -0.59)
Dominica	3 (2 to 5)	2 (1 to 3)	-40.81% (-73.16 to 31.75)	79.9 (44.3 to 135.8)	64.3 (33.8 to 109.1)	0.4% (-0.16 to 0.96)
Dominican Republic	213 (136 to 327)	112 (64 to 187)	-47.3% (-71.87 to -2.17)	51 (32.4 to 78.1)	23.3 (13.2 to 38.7)	-2.42% (-3 to -1.83)
Ecuador	528 (325 to 789)	201 (116 to 323)	-61.94% (-78.85 to -28.73)	95.9 (59 to 143.4)	24.6 (14.2 to 39.6)	-4.27% (-4.89 to -3.65)
Egypt	3130 (1827 to 5161)	407 (202 to 682)	-86.98% (-93.68 to -74.72)	113.9 (66.5 to 187.8)	9.2 (4.5 to 15.4)	-9.17% (-9.65 to -8.7)
El Salvador	789 (440 to 1269)	42 (23 to 68)	-94.7% (-97.44 to -88.66)	260.4 (145 to 418.5)	14.4 (7.8 to 23.4)	-9.43% (-10.67 to -8.17)
Equatorial Guinea	135 (67 to 225)	26 (13 to 46)	-80.83% (-91.79 to -58.59)	621.5 (310.3 to 1036.3)	34.4 (17 to 61.6)	-10.84% (-11.41 to -10.27)
Eritrea	1163 (522 to 2043)	1221 (603 to 2206)	4.97% (-53.82 to 166.56)	736.7 (330.7 to 1293.9)	327.2 (161.6 to 591.1)	-2.02% (-2.51 to -1.53)

Location	DALYs in 1990 (95% UI)	DALYs in 2019 (95% UI)	Relative change (%, 95 CI)	Age-standardized DALYs rate in 1990 (95% UI)	Age-standardized DALYs rate in 2019 (95% UI)	EAPC (%, 95 CI)
Eswatini	37 (20 to 63)	18 (9 to 34)	-50.59% (-77.87 to 13.28)	79 (42.8 to 133.1)	30.7 (15 to 57.2)	-2.73% (-3.62 to -1.84)
Ethiopia	14458 (8030 to 24123)	7733 (4472 to 12680)	-46.52% (-73.77 to 5.61)	561.5 (311.9 to 936.9)	125.4 (72.5 to 205.6)	-5.11% (-6.13 to -4.08)
Fiji	9 (5 to 17)	5 (3 to 9)	-44.82% (-76.59 to 31.27)	23.9 (12 to 44.6)	13.4 (6.8 to 25.3)	-1.97% (-2.15 to -1.79)
Gabon	74 (34 to 128)	28 (14 to 48)	-62.12% (-82.45 to -13.49)	143.9 (66.1 to 247.9)	30.3 (15.4 to 52)	-5.65% (-6.18 to -5.12)
Gambia	129 (65 to 221)	157 (74 to 266)	21.82% (-44.4 to 158.24)	250.1 (126.8 to 429.1)	118.2 (56 to 200.8)	-3.01% (-3.47 to -2.55)
Georgia	22 (12 to 39)	9 (5 to 14)	-59.94% (-75.23 to -32.97)	10.9 (5.8 to 19)	9.8 (5.5 to 15.7)	0.8% (0.32 to 1.28)
Ghana	856 (431 to 1576)	385 (198 to 660)	-54.96% (-78.94 to 1.96)	111.6 (56.3 to 205.6)	23.6 (12.1 to 40.4)	-4.93% (-5.42 to -4.44)
Global	357050 (273775 to 455659)	134087 (108142 to 163063)	-62.45% (-71.79 to -49.95)	139.7 (107.1 to 178.3)	44.4 (35.8 to 54)	-4.05% (-4.35 to -3.74)
Grenada	1 (0 to 1)	0 (0 to 0)	-64.19% (-78.4 to -44.86)	15.4 (8.3 to 26.5)	5.8 (2.7 to 10.6)	-3.3% (-3.74 to -2.87)

Location	DALYs in 1990 (95% UI)	DALYs in 2019 (95% UI)	Relative change (%, 95 CI)	Age-standardized DALYs rate in 1990 (95% UI)	Age-standardized DALYs rate in 2019 (95% UI)	EAPC (%, 95 CI)
Guatemala	648 (390 to 1012)	278 (160 to 440)	-57.07% (-76.12 to -25.76)	156.8 (94.4 to 244.6)	29.8 (17.1 to 47.1)	-6.68% (-7.56 to -5.79)
Guinea	1431 (801 to 2373)	1599 (800 to 2681)	11.77% (-46.17 to 125.82)	497.3 (278.5 to 824.8)	233.8 (116.9 to 392)	-2.18% (-2.34 to -2.01)
Guinea-Bissau	148 (76 to 256)	83 (39 to 148)	-44.42% (-75.48 to 22.85)	272.9 (139.7 to 469.7)	79 (37.1 to 142)	-3.97% (-4.36 to -3.57)
Guyana	23 (14 to 36)	7 (4 to 10)	-70.76% (-84.37 to -45.56)	52.1 (32.4 to 80.9)	18.8 (10.9 to 28.9)	-2.33% (-2.93 to -1.71)
Haiti	859 (406 to 1554)	1200 (614 to 2066)	39.66% (-38.41 to 207.05)	262.9 (124.3 to 475.4)	194.8 (99.7 to 335.4)	-0.22% (-0.49 to 0.05)
Honduras	1104 (648 to 1697)	168 (84 to 290)	-84.82% (-92.79 to -69.38)	433.9 (254.7 to 666.9)	31.5 (15.8 to 54.5)	-9.36% (-9.84 to -8.87)
India	188693 (117191 to 280546)	19562 (11618 to 32046)	-89.63% (-94.5 to -79.75)	470.9 (292.4 to 700.1)	30.5 (18.1 to 49.9)	-9.79% (-10.68 to -8.89)
Indonesia	7072 (4103 to 11172)	913 (562 to 1379)	-87.09% (-93.31 to -75.84)	71.4 (41.4 to 112.8)	8.1 (5 to 12.3)	-7.22% (-7.32 to -7.13)

Location	DALYs in 1990 (95% UI)	DALYs in 2019 (95% UI)	Relative change (%, 95 CI)	Age-standardized DALYs rate in 1990 (95% UI)	Age-standardized DALYs rate in 2019 (95% UI)	EAPC (%, 95 CI)
Iran (Islamic Republic of)	1060 (708 to 1490)	128 (80 to 195)	-87.89% (-92.45 to -81.95)	35.4 (23.7 to 49.8)	4.8 (3 to 7.3)	-6.31% (-6.67 to -5.94)
Iraq	181 (92 to 306)	143 (62 to 265)	-21.07% (-57.72 to 28.52)	19 (9.7 to 32.1)	6.7 (2.9 to 12.3)	-3.56% (-3.92 to -3.19)
Jamaica	32 (20 to 50)	6 (3 to 11)	-81.08% (-90.19 to -68.23)	25 (15.2 to 38.5)	5.1 (2.3 to 9)	-5.73% (-6.37 to -5.08)
Jordan	72 (39 to 123)	62 (32 to 100)	-15.06% (-59.09 to 61.09)	32.9 (17.6 to 56)	10.9 (5.6 to 17.7)	-4.5% (-4.8 to -4.21)
Kazakhstan	53 (24 to 99)	28 (13 to 50)	-47.58% (-72.04 to -9.55)	7.5 (3.4 to 14.1)	4.8 (2.2 to 8.5)	-1.5% (-1.92 to -1.09)
Kenya	2368 (1418 to 3598)	1885 (1113 to 2919)	-20.41% (-56.16 to 38.41)	177.9 (106.5 to 270.3)	64.9 (38.3 to 100.6)	-2.69% (-3.15 to -2.23)
Kiribati	3 (1 to 5)	3 (1 to 6)	13.53% (-55.31 to 182.08)	73.2 (36.8 to 130.2)	52.4 (24.5 to 99.5)	-1.37% (-1.68 to -1.05)
Kyrgyzstan	38 (24 to 58)	29 (17 to 43)	-24.16% (-55.04 to 27.06)	17.8 (11.1 to 27)	11.1 (6.7 to 16.4)	-0.34% (-0.85 to 0.18)
Lao People's Democratic Republic	1067 (582 to 1744)	87 (46 to 149)	-91.88% (-96.29 to -82.99)	484.9 (264.4 to 792.6)	24.7 (13.1 to 42.6)	-9.9% (-10.39 to -9.41)

Location	DALYs in 1990 (95% UI)	DALYs in 2019 (95% UI)	Relative change (%, 95 CI)	Age-standardized DALYs rate in 1990 (95% UI)	Age-standardized DALYs rate in 2019 (95% UI)	EAPC (%, 95 CI)
Lebanon	44 (24 to 76)	13 (6 to 23)	-70.13% (-84.01 to -50.09)	29.2 (15.5 to 50.2)	8.2 (3.8 to 14.1)	-4.28% (-4.36 to -4.21)
Lesotho	299 (157 to 486)	141 (68 to 256)	-52.83% (-79.08 to 4.07)	297.4 (156.3 to 483.6)	129.1 (62.1 to 234.7)	-1.38% (-2.08 to -0.68)
Liberia	595 (338 to 976)	743 (391 to 1244)	24.9% (-39.98 to 147.8)	567.7 (322.7 to 931.3)	279.5 (146.9 to 467.6)	-3.69% (-4.71 to -2.67)
Libya	33 (17 to 56)	5 (2 to 9)	-85.06% (-93.64 to -69.81)	14 (7.2 to 24.1)	1.7 (0.6 to 3.3)	-6.85% (-7.32 to -6.38)
Madagascar	2909 (1600 to 4804)	2772 (1429 to 4850)	-4.7% (-55.32 to 101.85)	453.9 (249.7 to 749.6)	189.4 (97.6 to 331.2)	-2.91% (-3.25 to -2.57)
Malawi	1055 (567 to 1730)	1185 (576 to 2180)	12.32% (-50.34 to 140.65)	206.5 (110.9 to 338.6)	101.4 (49.3 to 186.5)	-1.93% (-2.65 to -1.2)
Malaysia	202 (104 to 358)	44 (20 to 83)	-78.27% (-91.88 to -45.98)	23 (11.8 to 40.8)	3.3 (1.5 to 6.2)	-7.02% (-7.48 to -6.55)
Maldives	3 (2 to 6)	0 (0 to 1)	-89.28% (-95.49 to -78.16)	28.4 (15.3 to 47.8)	2.5 (1.1 to 4.7)	-7.53% (-8.34 to -6.71)
Mali	3888 (2279 to 5867)	3396 (1681 to 6103)	-12.67% (-58.05 to 84.44)	943 (552.8 to 1422.9)	276.3 (136.8 to 496.6)	-4.34% (-4.54 to -4.13)

Location	DALYs in 1990 (95% UI)	DALYs in 2019 (95% UI)	Relative change (%, 95 CI)	Age-standardized DALYs rate in 1990 (95% UI)	Age-standardized DALYs rate in 2019 (95% UI)	EAPC (%, 95 CI)
Marshall Islands	1<br>(1 to 2)	1<br>(0 to 1)	-42.97%<br>(-73.96 to 28.1)	48<br>(24.7 to 84.4)	24<br>(11.5 to 41.8)	-2.35%<br>(-2.75 to -1.95)
Mauritania	573<br>(323 to 924)	169<br>(79 to 313)	-70.46%<br>(-86.6 to -36.26)	540.3<br>(304.3 to 870.2)	73.4<br>(34.3 to 135.7)	-7.36%<br>(-7.71 to -7.01)
Mexico	1513<br>(980 to 2261)	605<br>(375 to 921)	-59.98%<br>(-75.16 to -39.3)	30<br>(19.5 to 44.9)	11.1<br>(6.9 to 16.9)	-2.96%<br>(-3.18 to -2.73)
Micronesia (Federated States of)	4<br>(2 to 7)	1<br>(0 to 2)	-72.22%<br>(-88.81 to -32.83)	66<br>(30.2 to 122.3)	19.3<br>(9.2 to 35.1)	-4.36%<br>(-4.49 to -4.24)
Mongolia	82<br>(44 to 138)	15<br>(8 to 24)	-82.18%<br>(-91.89 to -62.33)	71.1<br>(37.8 to 119.8)	13.6<br>(7 to 22.7)	-6.92%<br>(-7.72 to -6.11)
Montenegro	1<br>(0 to 2)	0<br>(0 to 1)	-71.63%<br>(-93.39 to -24.49)	3.7<br>(1.2 to 7.6)	1.4<br>(0.2 to 3.3)	-3.99%<br>(-4.22 to -3.77)
Morocco	2535<br>(1287 to 4224)	227<br>(117 to 394)	-91.04%<br>(-95.95 to -81.1)	187.3<br>(95.1 to 312.2)	14.9<br>(7.7 to 25.8)	-8.56%<br>(-8.74 to -8.39)
Mozambique	2910<br>(1551 to 4787)	1735<br>(882 to 3035)	-40.4%<br>(-71.1 to 24.39)	411.3<br>(219.2 to 676.4)	101.9<br>(51.8 to 178.2)	-4.38%<br>(-4.72 to -4.05)
Myanmar	3949<br>(1956 to 6880)	823<br>(404 to 1530)	-79.15%<br>(-91.26 to -51.95)	185.2<br>(91.7 to 322.7)	33.2<br>(16.3 to 61.7)	-6.59%<br>(-7.11 to -6.07)
Namibia	129<br>(62 to 223)	46<br>(22 to 84)	-64.77%<br>(-85.11 to -15.32)	158.2<br>(75.6 to 272.5)	37.5<br>(18 to 69.4)	-4.4%<br>(-5.07 to -3.74)

Location	DALYs in 1990 (95% UI)	DALYs in 2019 (95% UI)	Relative change (%, 95 CI)	Age-standardized DALYs rate in 1990 (95% UI)	Age-standardized DALYs rate in 2019 (95% UI)	EAPC (%, 95 CI)
Nepal	3859 (2037 to 6264)	397 (219 to 655)	-89.72% (-95.08 to -78.3)	397 (209.6 to 644.4)	23.7 (13.1 to 39.2)	-9.59% (-9.82 to -9.36)
Nicaragua	226 (134 to 356)	51 (28 to 84)	-77.31% (-88.74 to -56.04)	103.9 (61.7 to 163.6)	16.7 (9.2 to 27.2)	-6.74% (-7.3 to -6.18)
Niger	2935 (1640 to 4851)	4842 (2344 to 8221)	64.96% (-21.92 to 259.58)	714.4 (399.2 to 1180.7)	372.8 (180.5 to 632.9)	-2.49% (-2.73 to -2.25)
Nigeria	7003 (3647 to 12197)	7261 (3692 to 13517)	3.68% (-50.29 to 100.24)	140.7 (73.3 to 245.1)	57.6 (29.3 to 107.2)	-3.85% (-4.61 to -3.08)
North Macedonia	7 (3 to 11)	1 (0 to 3)	-81.94% (-93.56 to -65.96)	7.9 (3.9 to 13.6)	2 (0.5 to 4.3)	-5.39% (-5.73 to -5.06)
Pakistan	11711 (6887 to 18692)	7403 (4477 to 11530)	-36.78% (-67.73 to 25.87)	207.6 (122.1 to 331.4)	61.8 (37.4 to 96.3)	-4.3% (-4.58 to -4.02)
Papua New Guinea	591 (281 to 1056)	554 (286 to 978)	-6.22% (-59.53 to 112.8)	285.8 (136.1 to 511.2)	123 (63.6 to 217.4)	-2.51% (-2.9 to -2.11)
Paraguay	235 (134 to 370)	59 (33 to 95)	-75.11% (-87.58 to -50.29)	117.7 (67 to 185.2)	18.5 (10.3 to 29.9)	-7.03% (-7.54 to -6.53)

Location	DALYs in 1990 (95% UI)	DALYs in 2019 (95% UI)	Relative change (%, 95 CI)	Age-standardized DALYs rate in 1990 (95% UI)	Age-standardized DALYs rate in 2019 (95% UI)	EAPC (%, 95 CI)
Peru	1503 (861 to 2402)	245 (133 to 408)	-83.67% (-91.71 to -69.81)	127.8 (73.1 to 204.1)	18.1 (9.8 to 30.1)	-7.27% (-7.62 to -6.91)
Philippines	885 (572 to 1273)	555 (337 to 840)	-37.27% (-62.17 to -2.41)	25.9 (16.7 to 37.3)	10.6 (6.4 to 16)	-2.4% (-2.67 to -2.14)
Republic of Moldova	30 (19 to 46)	4 (1 to 7)	-88.13% (-94.89 to -78.62)	17.7 (11.2 to 27.5)	3.7 (1.5 to 7.2)	-5.74% (-6.29 to -5.19)
Russian Federation	808 (520 to 1165)	124 (68 to 204)	-84.67% (-90.26 to -78.82)	15.6 (10.1 to 22.5)	3.4 (1.9 to 5.7)	-5.44% (-5.7 to -5.17)
Rwanda	1418 (754 to 2328)	648 (298 to 1255)	-54.31% (-80.82 to 6.22)	374.4 (199 to 614.7)	91.2 (41.9 to 176.6)	-4.68% (-5.74 to -3.6)
Saint Lucia	2 (1 to 3)	0 (0 to 1)	-77.63% (-87.6 to -64.49)	22.7 (14 to 35.2)	5.9 (3.2 to 10)	-3.66% (-4.36 to -2.97)
Saint Vincent and the Grenadines	1 (0 to 1)	0 (0 to 1)	-57.98% (-74.39 to -30.88)	12.2 (5.9 to 21.8)	7.1 (3.4 to 13.1)	-1.64% (-1.81 to -1.48)
Samoa	3 (1 to 6)	1 (0 to 1)	-76.27% (-90.82 to -38.62)	32.1 (15.2 to 60.6)	6.2 (2.6 to 12)	-6.61% (-7.18 to -6.03)
Senegal	1155 (608 to 2035)	788 (383 to 1411)	-31.82% (-70.27 to 49.26)	279.8 (147.1 to 492.9)	98 (47.7 to 175.5)	-3.32% (-3.6 to -3.04)
Serbia	21 (10 to 38)	5 (1 to 10)	-77.68% (-91.97 to -54.48)	6.2 (3 to 10.9)	1.9 (0.5 to 4.1)	-4.42% (-4.71 to -4.12)

Location	DALYs in 1990 (95% UI)	DALYs in 2019 (95% UI)	Relative change (%, 95 CI)	Age-standardized DALYs rate in 1990 (95% UI)	Age-standardized DALYs rate in 2019 (95% UI)	EAPC (%, 95 CI)
Sierra Leone	597 (308 to 997)	1168 (624 to 1970)	95.52% (-9.49 to 285.84)	330.7 (170.5 to 551.7)	246.5 (131.6 to 415.7)	-0.94% (-1.76 to -0.11)
Solomon Islands	26 (12 to 52)	33 (15 to 61)	28.21% (-44.9 to 222.37)	136 (62.4 to 273.5)	101.7 (46.6 to 188.3)	-0.69% (-1.29 to -0.08)
Somalia	1836 (853 to 3382)	6167 (3040 to 11122)	235.95% (64.08 to 643.45)	499.5 (232.1 to 920.1)	558.9 (275.5 to 1008.1)	0.67% (0.37 to 0.98)
South Africa	1329 (844 to 1931)	344 (188 to 559)	-74.15% (-86.15 to -53.64)	64 (40.7 to 93)	15.4 (8.4 to 25)	-2.68% (-3.92 to -1.42)
South Sudan	452 (206 to 871)	591 (262 to 1189)	30.72% (-44.65 to 225.78)	143.8 (65.5 to 277.2)	109.4 (48.6 to 220.3)	-1.15% (-1.41 to -0.9)
Sri Lanka	143 (76 to 250)	24 (12 to 42)	-83.14% (-92.89 to -65.01)	17 (9 to 29.6)	2.8 (1.3 to 4.8)	-6.2% (-6.39 to -6.01)
Sudan	4695 (2587 to 7403)	1887 (969 to 3359)	-59.81% (-81.81 to -11.56)	439.4 (242.2 to 692.8)	87.6 (45 to 156)	-5.27% (-5.76 to -4.79)
Suriname	6 (3 to 10)	5 (2 to 8)	-23.03% (-63.41 to 80.79)	30.8 (15.1 to 52.4)	19.9 (10.6 to 34)	-0.75% (-1.76 to 0.26)
Syrian Arab Republic	336 (180 to 551)	58 (28 to 102)	-82.63% (-91.75 to -65.89)	47.5 (25.5 to 78)	6.4 (3.1 to 11.1)	-7.83% (-8.24 to -7.43)

Location	DALYs in 1990 (95% UI)	DALYs in 2019 (95% UI)	Relative change (%, 95 CI)	Age-standardized DALYs rate in 1990 (95% UI)	Age-standardized DALYs rate in 2019 (95% UI)	EAPC (%, 95 CI)
Tajikistan	42<br>(23 to 71)	31<br>(14 to 58)	-27.41%<br>(-58.5 to 10.74)	15.2<br>(8.2 to 25.9)	7.3<br>(3.2 to 13.7)	-3.46%<br>(-3.85 to -3.08)
Thailand	260<br>(131 to 448)	95<br>(44 to 174)	-63.46%<br>(-82.61 to -32.09)	8.8<br>(4.4 to 15.2)	4.5<br>(2.1 to 8.3)	-1.88%<br>(-2.09 to -1.68)
Timor-Leste	101<br>(33 to 200)	48<br>(20 to 87)	-52.27%<br>(-82.65 to 42.57)	292.1<br>(95.8 to 577.2)	61.7<br>(26.1 to 111.1)	-7.23%<br>(-8.3 to -6.15)
Togo	371<br>(191 to 639)	283<br>(126 to 525)	-23.84%<br>(-66.75 to 71.78)	190.5<br>(98 to 327.7)	72.5<br>(32.3 to 134.6)	-2.97%<br>(-3.44 to -2.51)
Tonga	1<br>(1 to 2)	1<br>(0 to 1)	-30.38%<br>(-70.85 to 66.36)	20.1<br>(10.3 to 36.4)	15.1<br>(7.1 to 28.8)	-1.14%<br>(-1.85 to -0.43)
Tunisia	172<br>(88 to 299)	14<br>(6 to 26)	-91.77%<br>(-96.62 to -80.96)	39.5<br>(20.2 to 68.8)	3.6<br>(1.6 to 6.7)	-7.79%<br>(-8.22 to -7.36)
Turkey	1724<br>(913 to 3050)	145<br>(70 to 255)	-91.58%<br>(-96.14 to -83.08)	54.9<br>(29.1 to 97.1)	4.6<br>(2.2 to 8)	-9.27%<br>(-9.93 to -8.61)
Turkmenistan	10<br>(5 to 18)	7<br>(3 to 13)	-30.01%<br>(-63.01 to 22.47)	5.6<br>(2.8 to 9.7)	3.8<br>(1.7 to 6.8)	-1.03%<br>(-1.35 to -0.71)
Tuvalu	0<br>(0 to 1)	0<br>(0 to 0)	-71.58%<br>(-89.22 to -29.68)	72.2<br>(32.4 to 130.9)	15<br>(7.2 to 29.5)	-5%<br>(-5.09 to -4.91)

Location	DALYs in 1990 (95% UI)	DALYs in 2019 (95% UI)	Relative change (%, 95 CI)	Age-standardized DALYs rate in 1990 (95% UI)	Age-standardized DALYs rate in 2019 (95% UI)	EAPC (%, 95 CI)
Uganda	1852 (756 to 3427)	2565 (1259 to 4595)	38.53% (-42.21 to 271.04)	190.7 (77.9 to 352.9)	106 (52 to 189.9)	-2.24% (-2.89 to -1.58)
Ukraine	204 (112 to 345)	40 (18 to 75)	-80.58% (-89.28 to -68.64)	11.3 (6.2 to 19.1)	4 (1.8 to 7.6)	-3.98% (-4.47 to -3.49)
United Republic of Tanzania	3628 (2014 to 6034)	2993 (1564 to 5200)	-17.48% (-64.85 to 80.31)	245.4 (136.2 to 408.1)	95.2 (49.7 to 165.3)	-2.59% (-2.97 to -2.22)
Uzbekistan	173 (105 to 268)	113 (65 to 186)	-34.81% (-59.87 to 5.51)	16.4 (9.9 to 25.3)	8.1 (4.6 to 13.4)	-3.06% (-3.52 to -2.6)
Vanuatu	3 (2 to 6)	5 (2 to 9)	55.86% (-34.56 to 272.2)	44.1 (21.2 to 84.4)	36.7 (17.7 to 66.7)	-1.5% (-1.85 to -1.15)
Venezuela (Bolivarian Republic of)	879 (580 to 1228)	517 (326 to 784)	-41.14% (-66.36 to 0.79)	89.1 (58.8 to 124.4)	46.3 (29.1 to 70.1)	-2.04% (-2.97 to -1.1)
Viet Nam	522 (264 to 921)	79 (25 to 165)	-84.94% (-95.1 to -66.78)	14.5 (7.3 to 25.5)	2.4 (0.8 to 5)	-5.96% (-6.23 to -5.69)
Yemen	1432 (383 to 3347)	2037 (695 to 4333)	42.26% (-57.42 to 482.49)	226.6 (60.6 to 529.6)	123.8 (42.3 to 263.4)	-2.55% (-3.02 to -2.09)
Zambia	994 (558 to 1692)	681 (361 to 1180)	-31.49% (-66.98 to 42.69)	203.5 (114.2 to 346.5)	64.7 (34.3 to 112.2)	-3.66% (-4.5 to -2.81)

Location	DALYs in 1990 (95% UI)	DALYs in 2019 (95% UI)	Relative change (%, 95 CI)	Age-standardized DALYs rate in 1990 (95% UI)	Age-standardized DALYs rate in 2019 (95% UI)	EAPC (%, 95 CI)
Zimbabwe	1124 (661 to 1821)	2000 (989 to 3626)	77.98% (-14.46 to 271.21)	183.4 (107.9 to 297.1)	237.9 (117.7 to 431.3)	4.01% (2.62 to 5.43)

CI – confidence interval, DALYs – disability-adjusted life years (per 100,000 population), EAPC – estimated annual percentage change, MSMI – maternal sepsis and other maternal infections, UI – uncertain interval.

Table S27. DALYs of MSMI in 131 low- and middle-income countries and territories with EAPC in age group of 20-24 years from 1990 and 2019.

Location	DALYs in 1990 (95% UI)	DALYs in 2019 (95% UI)	Relative change (%, 95 CI)	Age-standardized DALYs rate in 1990 (95% UI)	Age-standardized DALYs rate in 2019 (95% UI)	EAPC (%, 95 CI)
Afghanistan	4211 (2351 to 6997)	4923 (2622 to 8518)	16.93% (-42.14 to 129.81)	823.4 (459.7 to 1368.2)	268.1 (142.8 to 463.7)	-4.23% (-5.35 to -3.09)
Albania	36 (18 to 61)	14 (6 to 25)	-61.26% (-74.46 to -45.5)	23.5 (11.8 to 40.6)	13.2 (6.1 to 24.3)	-2.11% (-2.29 to -1.92)
Algeria	5015 (2654 to 8543)	438 (252 to 711)	-91.26% (-95.64 to -82.64)	404.1 (213.9 to 688.4)	27.5 (15.8 to 44.6)	-9.19% (-9.59 to -8.79)
American Samoa	2 (1 to 3)	1 (0 to 1)	-58.2% (-80.39 to -15.43)	68.3 (38.4 to 115.9)	28.1 (15.2 to 46.5)	-3.45% (-3.61 to -3.28)
Angola	1941 (929 to 3340)	1775 (947 to 3041)	-8.55% (-58.93 to 102.93)	439.5 (210.5 to 756.4)	133.1 (71 to 228.1)	-3.82% (-4.25 to -3.39)
Armenia	64 (39 to 102)	17 (8 to 29)	-74.13% (-83.62 to -62.25)	45.5 (27.4 to 72.2)	18.1 (9.2 to 31.8)	-3.34% (-3.81 to -2.86)
Azerbaijan	152 (92 to 238)	63 (28 to 112)	-58.38% (-76.29 to -38.44)	41.9 (25.3 to 65.7)	16.4 (7.3 to 29.1)	-3.96% (-4.25 to -3.66)

Location	DALYs in 1990 (95% UI)	DALYs in 2019 (95% UI)	Relative change (%, 95 CI)	Age-standardized DALYs rate in 1990 (95% UI)	Age-standardized DALYs rate in 2019 (95% UI)	EAPC (%, 95 CI)
Bangladesh	8858 (5218 to 14210)	970 (466 to 1762)	-89.04% (-94.81 to -78.13)	170.3 (100.3 to 273.2)	12.5 (6 to 22.7)	-8.42% (-9.43 to -7.4)
Belarus	93 (50 to 161)	25 (11 to 48)	-72.76% (-82.69 to -60.91)	25.7 (13.7 to 44.5)	11.3 (4.8 to 21.5)	-2.37% (-2.62 to -2.11)
Belize	8 (5 to 12)	8 (5 to 13)	3.59% (-40.86 to 87.02)	93.9 (60.2 to 139.1)	41.9 (25.7 to 64.3)	-2.23% (-2.66 to -1.81)
Benin	1414 (816 to 2206)	1280 (633 to 2239)	-9.46% (-56.93 to 80.34)	646.4 (373.1 to 1008.5)	211.6 (104.6 to 369.9)	-3.26% (-3.56 to -2.95)
Bhutan	46 (24 to 79)	6 (3 to 10)	-86.58% (-93.8 to -71.66)	156.4 (80.8 to 268.5)	17.1 (8.3 to 28.9)	-7.71% (-8.02 to -7.4)
Bolivia (Plurinational State of)	1571 (899 to 2468)	498 (269 to 848)	-68.29% (-84.74 to -38.72)	553.3 (316.7 to 869)	94.3 (51 to 160.5)	-6.37% (-6.6 to -6.15)
Bosnia and Herzegovina	77 (47 to 115)	6 (3 to 13)	-91.6% (-96.31 to -85.19)	40.2 (24.5 to 60.4)	6.2 (2.4 to 12.3)	-7.75% (-8.95 to -6.53)
Botswana	230 (127 to 399)	74 (37 to 136)	-67.93% (-85.26 to -31.91)	356.9 (196.8 to 618.9)	70.5 (35 to 129.7)	-5.66% (-6.29 to -5.03)
Brazil	6380 (4195 to 9091)	2930 (1952 to 4127)	-54.08% (-72.13 to -24.03)	90.1 (59.2 to 128.4)	33.7 (22.5 to 47.5)	-2.33% (-2.77 to -1.9)

Location	DALYs in 1990 (95% UI)	DALYs in 2019 (95% UI)	Relative change (%, 95 CI)	Age-standardized DALYs rate in 1990 (95% UI)	Age-standardized DALYs rate in 2019 (95% UI)	EAPC (%, 95 CI)
Bulgaria	99<br>(60 to 147)	13<br>(6 to 24)	-86.5%<br>(-92.31 to -79.48)	34.7<br>(21 to 51.9)	9<br>(4.2 to 16.4)	-4.31%<br>(-4.77 to -3.84)
Burkina Faso	2728<br>(1526 to 4446)	2462<br>(1234 to 4370)	-9.78%<br>(-57.98 to 83.21)	681.6<br>(381.2 to 1110.7)	240.7<br>(120.6 to 427.3)	-4.18%<br>(-4.64 to -3.72)
Burundi	2601<br>(1387 to 4346)	3800<br>(2176 to 5944)	46.11%<br>(-24.69 to 181.94)	1068.5<br>(569.7 to 1785.4)	692.1<br>(396.2 to 1082.4)	-1.67%<br>(-2.26 to -1.09)
Cabo Verde	26<br>(14 to 43)	3<br>(1 to 5)	-89.47%<br>(-95.07 to -77.45)	156.5<br>(81 to 255.4)	11<br>(5.7 to 18.7)	-9.39%<br>(-9.62 to -9.15)
Cambodia	2713<br>(1409 to 4724)	396<br>(208 to 686)	-85.39%<br>(-93.25 to -70.53)	567.5<br>(294.8 to 988.2)	53.2<br>(28 to 92)	-9.14%<br>(-10.03 to -8.23)
Cameroon	2532<br>(1464 to 3981)	2868<br>(1299 to 5217)	13.27%<br>(-48.17 to 130.99)	530.4<br>(306.8 to 834.2)	205.9<br>(93.3 to 374.5)	-3.1%<br>(-3.63 to -2.56)
Central African Republic	1293<br>(707 to 2068)	2607<br>(1389 to 4297)	101.55%<br>(2.17 to 312.51)	987<br>(539.3 to 1578.3)	987.4<br>(526 to 1627.6)	0.15%<br>(-0.04 to 0.33)
Chad	2837<br>(1612 to 4400)	4545<br>(2464 to 7372)	60.21%<br>(-16 to 191.38)	1118.4<br>(635.6 to 1734.5)	640<br>(347 to 1038)	-1.88%<br>(-2.3 to -1.46)

Location	DALYs in 1990 (95% UI)	DALYs in 2019 (95% UI)	Relative change (%, 95 CI)	Age-standardized DALYs rate in 1990 (95% UI)	Age-standardized DALYs rate in 2019 (95% UI)	EAPC (%, 95 CI)
China	43945 (33089 to 57480)	1997 (899 to 3708)	-95.46% (-97.72 to -92.49)	67.9 (51.1 to 88.8)	5.1 (2.3 to 9.5)	-8.52% (-9.18 to -7.87)
Colombia	1473 (934 to 2264)	531 (309 to 852)	-63.95% (-80.97 to -35.86)	88 (55.8 to 135.3)	26 (15.1 to 41.7)	-3.89% (-4.35 to -3.43)
Comoros	29 (6 to 61)	15 (6 to 28)	-48.21% (-80.02 to 154.01)	136.8 (27.5 to 290.8)	45.3 (19.3 to 84.4)	-4.79% (-5.78 to -3.78)
Congo	439 (201 to 782)	286 (151 to 499)	-34.79% (-70.39 to 75.26)	383.4 (175.5 to 683.6)	127.3 (67.4 to 221.9)	-3.52% (-4.38 to -2.64)
Costa Rica	52 (33 to 75)	19 (10 to 32)	-63.02% (-81.07 to -41.6)	35.6 (22.5 to 51.6)	9.5 (4.9 to 15.9)	-4.92% (-5.71 to -4.12)
Côte d'Ivoire	1656 (891 to 2787)	1579 (734 to 2897)	-4.7% (-58.24 to 98.32)	286.6 (154.2 to 482.2)	135.6 (63.1 to 248.9)	-2.16% (-2.6 to -1.73)
Cuba	204 (128 to 298)	50 (29 to 81)	-75.54% (-86.05 to -59.12)	36 (22.7 to 52.7)	14.5 (8.3 to 23.7)	-2.63% (-3.02 to -2.24)
Democratic People's Republic of Korea	885 (466 to 1601)	505 (257 to 905)	-42.96% (-73.36 to 26.48)	84.2 (44.3 to 152.2)	50.7 (25.8 to 90.9)	-1.45% (-2.26 to -0.63)

Location	DALYs in 1990 (95% UI)	DALYs in 2019 (95% UI)	Relative change (%, 95 CI)	Age-standardized DALYs rate in 1990 (95% UI)	Age-standardized DALYs rate in 2019 (95% UI)	EAPC (%, 95 CI)
Democratic Republic of the Congo	12612 (6397 to 21223)	28182 (17382 to 44357)	123.45% (13.41 to 340.85)	750.6 (380.7 to 1263)	702.4 (433.2 to 1105.5)	1.31% (0.15 to 2.48)
Djibouti	167 (85 to 279)	176 (74 to 325)	5.85% (-55.25 to 146.98)	734 (374.1 to 1228.2)	382.5 (159.8 to 705.5)	-1.99% (-2.56 to -1.41)
Dominica	5 (3 to 9)	3 (1 to 5)	-50.18% (-77.78 to 13.73)	155.3 (88.3 to 262.6)	100.8 (51.9 to 176.8)	-0.29% (-1.1 to 0.53)
Dominican Republic	312 (199 to 471)	210 (112 to 342)	-32.72% (-64.01 to 20.1)	80.7 (51.5 to 121.9)	42.9 (23 to 70)	-1.52% (-2.22 to -0.81)
Ecuador	819 (524 to 1213)	314 (179 to 504)	-61.65% (-79.69 to -30)	168.9 (108.1 to 250.3)	39.5 (22.5 to 63.4)	-4.12% (-4.98 to -3.26)
Egypt	4889 (2952 to 7526)	889 (456 to 1495)	-81.82% (-91.24 to -66.81)	202.9 (122.5 to 312.3)	20.6 (10.6 to 34.7)	-7.94% (-8.31 to -7.58)
El Salvador	1081 (640 to 1621)	66 (36 to 112)	-93.89% (-97.01 to -87.79)	414.9 (245.9 to 622.3)	20.7 (11.2 to 35.3)	-9.48% (-10.69 to -8.25)
Equatorial Guinea	251 (142 to 402)	31 (14 to 56)	-87.65% (-94.35 to -73.39)	1341.9 (758 to 2150.7)	47.1 (22 to 84.8)	-12.25% (-12.92 to -11.58)

Location	DALYs in 1990 (95% UI)	DALYs in 2019 (95% UI)	Relative change (%, 95 CI)	Age-standardized DALYs rate in 1990 (95% UI)	Age-standardized DALYs rate in 2019 (95% UI)	EAPC (%, 95 CI)
Eritrea	2113 (1039 to 3453)	1872 (1029 to 3277)	-11.41% (-56.54 to 103.01)	1596.3 (785 to 2608.7)	571.4 (314.2 to 1000.5)	-2.58% (-3.14 to -2.01)
Eswatini	62 (33 to 107)	28 (13 to 54)	-54.44% (-80.77 to 11.89)	156.2 (81.8 to 266.7)	48.9 (23.2 to 92.7)	-3.23% (-4.25 to -2.19)
Ethiopia	35586 (19395 to 56009)	13448 (7137 to 22425)	-62.21% (-81.41 to -26.57)	1546.6 (843 to 2434.3)	253.4 (134.5 to 422.5)	-6.4% (-7.29 to -5.5)
Fiji	23 (12 to 38)	11 (6 to 17)	-53.98% (-77.34 to -5.09)	65 (34.7 to 108.1)	28.9 (16.2 to 47.7)	-3.32% (-3.65 to -2.99)
Gabon	136 (70 to 222)	46 (24 to 77)	-66.41% (-84.13 to -25.89)	303.5 (156.9 to 495.6)	51.7 (26.6 to 86.7)	-6.36% (-7 to -5.7)
Gambia	244 (127 to 407)	305 (162 to 502)	25.03% (-36.08 to 139.48)	519.3 (271.1 to 865.9)	267.7 (142 to 440.3)	-2.49% (-2.94 to -2.04)
Georgia	53 (30 to 84)	27 (15 to 41)	-49.88% (-67.05 to -19.96)	25.1 (14 to 40)	26.4 (15.2 to 41.1)	1.9% (1.1 to 2.71)
Ghana	2080 (1118 to 3538)	944 (520 to 1619)	-54.64% (-79.54 to -7.71)	300.2 (161.4 to 510.6)	59.1 (32.6 to 101.4)	-4.97% (-5.37 to -4.57)

Location	DALYs in 1990 (95% UI)	DALYs in 2019 (95% UI)	Relative change (%, 95 CI)	Age-standardized DALYs rate in 1990 (95% UI)	Age-standardized DALYs rate in 2019 (95% UI)	EAPC (%, 95 CI)
Global	619895 (489991 to 778300)	229815 (185098 to 278688)	-62.93% (-71.82 to -52.8)	253.7 (200.5 to 318.5)	77.7 (62.6 to 94.2)	-4.25% (-4.58 to -3.93)
Grenada	1 (1 to 2)	0 (0 to 1)	-67.72% (-81.62 to -50.3)	31.8 (18.7 to 50.1)	8.3 (4.1 to 15.3)	-3.89% (-4.34 to -3.44)
Guatemala	1393 (816 to 2116)	477 (278 to 767)	-65.75% (-81.21 to -36.85)	403.6 (236.5 to 613.1)	51.4 (29.9 to 82.7)	-8.01% (-8.88 to -7.14)
Guinea	2362 (1342 to 3799)	2779 (1527 to 4692)	17.63% (-39.12 to 121.28)	905.2 (514.4 to 1455.8)	465.5 (255.7 to 786.1)	-1.78% (-1.95 to -1.61)
Guinea-Bissau	240 (133 to 413)	136 (70 to 237)	-43.23% (-72.16 to 10.43)	520.8 (288.7 to 898.1)	144 (73.7 to 250.7)	-4.01% (-4.38 to -3.63)
Guyana	42 (25 to 66)	18 (10 to 29)	-58.56% (-79.27 to -18.16)	104.2 (61.2 to 161.1)	43.8 (26 to 72)	-3.06% (-3.48 to -2.63)
Haiti	2423 (1179 to 4245)	3277 (1766 to 5410)	35.25% (-31.4 to 177.87)	853.4 (415.3 to 1495.4)	572.9 (308.8 to 945.9)	-0.63% (-0.99 to -0.28)
Honduras	1857 (1085 to 2734)	255 (129 to 460)	-86.29% (-93.52 to -73.33)	918.6 (536.8 to 1352.7)	49.4 (25 to 89.3)	-10.42% (-10.79 to -10.05)

Location	DALYs in 1990 (95% UI)	DALYs in 2019 (95% UI)	Relative change (%, 95 CI)	Age-standardized DALYs rate in 1990 (95% UI)	Age-standardized DALYs rate in 2019 (95% UI)	EAPC (%, 95 CI)
India	289950 (178152 to 437481)	39207 (23154 to 63274)	-86.48% (-92.53 to -74.47)	778.1 (478.1 to 1174)	62.5 (36.9 to 100.9)	-8.96% (-9.7 to -8.21)
Indonesia	17082 (10031 to 26492)	2727 (1657 to 4191)	-84.03% (-91.19 to -71.02)	187.7 (110.2 to 291)	25.4 (15.4 to 39.1)	-6.46% (-6.72 to -6.2)
Iran (Islamic Republic of)	2096 (1358 to 3063)	286 (160 to 463)	-86.37% (-91.94 to -79.56)	76.9 (49.8 to 112.4)	10 (5.6 to 16.3)	-6.51% (-6.78 to -6.24)
Iraq	261 (136 to 450)	257 (115 to 478)	-1.49% (-40.38 to 44.16)	36.1 (18.8 to 62.2)	12.2 (5.5 to 22.7)	-3.71% (-4.04 to -3.37)
Jamaica	43 (25 to 65)	13 (7 to 23)	-70.04% (-82.54 to -50.56)	36.2 (21.3 to 54.4)	9.8 (5 to 17)	-5.51% (-6.14 to -4.87)
Jordan	198 (109 to 331)	100 (53 to 166)	-49.41% (-75.63 to 4.51)	108.1 (59.3 to 180.7)	20.1 (10.6 to 33.3)	-6.72% (-7.14 to -6.3)
Kazakhstan	155 (71 to 274)	121 (59 to 212)	-21.75% (-42.6 to 9.96)	23.8 (10.9 to 42.2)	19.4 (9.4 to 34)	-0.28% (-0.52 to -0.03)
Kenya	4040 (2522 to 6148)	3202 (1762 to 5044)	-20.75% (-56.26 to 43.91)	366.6 (228.8 to 557.9)	127.7 (70.3 to 201.2)	-3.22% (-3.65 to -2.79)

Location	DALYs in 1990 (95% UI)	DALYs in 2019 (95% UI)	Relative change (%, 95 CI)	Age-standardized DALYs rate in 1990 (95% UI)	Age-standardized DALYs rate in 2019 (95% UI)	EAPC (%, 95 CI)
Kiribati	8 (4 to 14)	7 (3 to 13)	-11.24% (-64.73 to 100.83)	215.4 (112.6 to 371.7)	132.7 (63.6 to 237.9)	-1.73% (-1.91 to -1.56)
Kyrgyzstan	102 (64 to 153)	104 (65 to 153)	1.95% (-34.2 to 53.19)	54.2 (34.2 to 81.2)	36.9 (23 to 54.1)	-0.3% (-0.95 to 0.36)
Lao People's Democratic Republic	1697 (969 to 2824)	181 (101 to 295)	-89.34% (-94.61 to -79.39)	924.5 (527.6 to 1538.4)	52.7 (29.3 to 85.9)	-9.65% (-10.24 to -9.05)
Lebanon	71 (41 to 113)	21 (10 to 36)	-71.02% (-84.03 to -52.96)	53.5 (30.9 to 85.1)	12.4 (6 to 21.9)	-4.88% (-5.04 to -4.71)
Lesotho	414 (237 to 679)	218 (103 to 391)	-47.25% (-76.56 to 13.61)	498.3 (285.4 to 817.7)	210.1 (99.5 to 376.3)	-1.01% (-1.87 to -0.14)
Liberia	619 (342 to 991)	1185 (631 to 1947)	91.5% (-7.28 to 294.75)	902.9 (499.4 to 1445.6)	514.4 (273.8 to 845.3)	-3.09% (-4.01 to -2.16)
Libya	52 (28 to 86)	11 (5 to 18)	-79.48% (-90.13 to -62.04)	28.2 (15.1 to 47)	3.7 (1.7 to 6.3)	-6.16% (-6.83 to -5.48)
Madagascar	4287 (2609 to 6724)	4847 (2747 to 7884)	13.07% (-43.38 to 108.83)	803.9 (489.3 to 1260.9)	380.4 (215.6 to 618.6)	-2.55% (-2.94 to -2.16)
Malawi	1812 (965 to 3027)	1560 (777 to 2699)	-13.9% (-61.62 to 84.48)	398.2 (212 to 665.2)	171.7 (85.6 to 297.1)	-2.74% (-3.27 to -2.2)

Location	DALYs in 1990 (95% UI)	DALYs in 2019 (95% UI)	Relative change (%, 95 CI)	Age-standardized DALYs rate in 1990 (95% UI)	Age-standardized DALYs rate in 2019 (95% UI)	EAPC (%, 95 CI)
Malaysia	685 (381 to 1157)	133 (72 to 223)	-80.65% (-91.26 to -60.18)	84.4 (47 to 142.4)	9.5 (5.2 to 16)	-8.12% (-8.69 to -7.54)
Maldives	10 (6 to 16)	1 (1 to 3)	-85.68% (-93.37 to -72.67)	94.7 (53.1 to 151.5)	8.9 (4.2 to 16.4)	-7.3% (-8.12 to -6.47)
Mali	5817 (3603 to 8886)	4469 (2241 to 7831)	-23.18% (-61.47 to 42.53)	1590.2 (984.8 to 2429)	437.8 (219.6 to 767.2)	-4.63% (-4.89 to -4.37)
Marshall Islands	3 (2 to 5)	2 (1 to 3)	-42.46% (-74.17 to 22.42)	162.5 (89.3 to 278.8)	75.3 (39.4 to 128.8)	-2.7% (-3.14 to -2.25)
Mauritania	1284 (750 to 1951)	332 (162 to 606)	-74.14% (-87.56 to -48.01)	1401.2 (817.8 to 2128.2)	171.4 (83.6 to 312.9)	-7.65% (-7.93 to -7.37)
Mexico	2309 (1514 to 3345)	1029 (618 to 1574)	-55.43% (-73.19 to -31.15)	53 (34.8 to 76.8)	19.1 (11.5 to 29.2)	-2.95% (-3.3 to -2.6)
Micronesia (Federated States of)	10 (5 to 18)	3 (2 to 6)	-65.59% (-86.42 to -20.06)	231.3 (112.6 to 421.3)	72.5 (32.5 to 132.6)	-4.09% (-4.22 to -3.97)
Mongolia	331 (185 to 540)	65 (36 to 104)	-80.45% (-90.13 to -60.86)	321.4 (180.2 to 525.3)	53.6 (29.8 to 86)	-7.39% (-7.95 to -6.82)
Montenegro	3 (1 to 6)	1 (0 to 2)	-62.2% (-78.9 to -40.83)	13.7 (5.7 to 25.9)	6.4 (2.4 to 12.4)	-2.98% (-3.14 to -2.83)
Morocco	3570 (1931 to 5982)	295 (161 to 520)	-91.73% (-95.99 to -82.84)	292.4 (158.2 to 489.9)	19.9 (10.9 to 35.1)	-9.02% (-9.21 to -8.83)

Location	DALYs in 1990 (95% UI)	DALYs in 2019 (95% UI)	Relative change (%, 95 CI)	Age-standardized DALYs rate in 1990 (95% UI)	Age-standardized DALYs rate in 2019 (95% UI)	EAPC (%, 95 CI)
Mozambique	4200 (2304 to 6912)	2253 (1176 to 3891)	-46.37% (-73.64 to 14.48)	754.3 (413.7 to 1241.2)	154.5 (80.7 to 266.9)	-5% (-5.47 to -4.52)
Myanmar	7796 (4201 to 13269)	1750 (897 to 3022)	-77.55% (-89.67 to -52.65)	398.8 (214.9 to 678.8)	73.7 (37.8 to 127.2)	-6.17% (-6.65 to -5.68)
Namibia	167 (83 to 282)	62 (31 to 112)	-63.09% (-83.39 to -13.65)	240.7 (119 to 406)	53 (26.5 to 96.6)	-4.67% (-5.15 to -4.18)
Nepal	7460 (4083 to 12094)	675 (385 to 1102)	-90.94% (-95.32 to -81.99)	851.5 (466.1 to 1380.5)	41.5 (23.6 to 67.6)	-10.34% (-10.56 to -10.13)
Nicaragua	358 (212 to 555)	64 (36 to 102)	-82.18% (-90.85 to -66.91)	196.9 (116.5 to 305)	21 (11.8 to 33.6)	-8% (-8.61 to -7.38)
Niger	3878 (2222 to 6089)	7152 (3715 to 11652)	84.42% (-8.31 to 254.21)	1138.2 (652.1 to 1787.2)	698.6 (362.9 to 1138.1)	-1.91% (-2.17 to -1.65)
Nigeria	11425 (5886 to 19492)	11578 (6172 to 20702)	1.34% (-52.2 to 106.86)	273.1 (140.7 to 465.9)	110.6 (58.9 to 197.7)	-3.91% (-4.67 to -3.14)
North Macedonia	21 (12 to 35)	4 (2 to 8)	-79.1% (-89.37 to -66.33)	25.8 (14.1 to 42.6)	6.4 (2.5 to 12.1)	-5.39% (-5.71 to -5.07)

Location	DALYs in 1990 (95% UI)	DALYs in 2019 (95% UI)	Relative change (%, 95 CI)	Age-standardized DALYs rate in 1990 (95% UI)	Age-standardized DALYs rate in 2019 (95% UI)	EAPC (%, 95 CI)
Pakistan	20368 (12298 to 30963)	11831 (7211 to 18775)	-41.91% (-69.96 to 9.98)	447.2 (270 to 679.8)	114.1 (69.6 to 181.1)	-4.96% (-5.25 to -4.66)
Papua New Guinea	1153 (609 to 1862)	1112 (586 to 1886)	-3.56% (-52.42 to 83.77)	613.5 (324.1 to 990.5)	250.2 (131.9 to 424.3)	-2.45% (-2.86 to -2.05)
Paraguay	435 (271 to 655)	90 (51 to 135)	-79.34% (-89.27 to -60.35)	247.8 (154.4 to 372.8)	28.6 (16.3 to 43.1)	-7.7% (-8.2 to -7.21)
Peru	3079 (1821 to 4934)	393 (205 to 633)	-87.23% (-93.36 to -76.98)	289.1 (171 to 463.3)	28.3 (14.7 to 45.6)	-8.78% (-9.22 to -8.33)
Philippines	2368 (1550 to 3512)	1322 (799 to 2066)	-44.19% (-67.16 to -10.4)	78.6 (51.4 to 116.5)	26.4 (15.9 to 41.3)	-3.22% (-3.42 to -3.02)
Republic of Moldova	77 (49 to 116)	10 (4 to 20)	-86.46% (-93.14 to -78.49)	50.3 (31.8 to 75.7)	10.1 (4.3 to 19.1)	-5.92% (-6.56 to -5.27)
Russian Federation	1657 (1046 to 2476)	410 (212 to 709)	-75.26% (-83.71 to -67.11)	33.9 (21.4 to 50.7)	11.8 (6.1 to 20.4)	-3.8% (-4.04 to -3.57)
Rwanda	4222 (2517 to 6452)	1462 (722 to 2579)	-65.39% (-84.75 to -30.31)	1342.9 (800.5 to 2051.9)	236 (116.6 to 416.4)	-6.2% (-7.17 to -5.22)
Saint Lucia	2 (1 to 3)	1 (0 to 1)	-68.66% (-82.31 to -49)	32.8 (20 to 50.5)	10.1 (5.5 to 16.4)	-3.06% (-3.77 to -2.34)

Location	DALYs in 1990 (95% UI)	DALYs in 2019 (95% UI)	Relative change (%, 95 CI)	Age-standardized DALYs rate in 1990 (95% UI)	Age-standardized DALYs rate in 2019 (95% UI)	EAPC (%, 95 CI)
Saint Vincent and the Grenadines	1 (0 to 2)	0 (0 to 1)	-52.87% (-68.41 to -28.74)	17.6 (9 to 31.4)	10.1 (5.1 to 18.2)	-1.66% (-1.98 to -1.33)
Samoa	6 (3 to 12)	2 (1 to 3)	-73.46% (-89.26 to -37.42)	88.7 (43.7 to 161.9)	19 (8.3 to 37.1)	-6.23% (-6.74 to -5.71)
Senegal	2085 (1190 to 3336)	1544 (810 to 2668)	-25.91% (-64.93 to 44.38)	608.2 (347.2 to 973.4)	219.8 (115.3 to 379.7)	-3.07% (-3.38 to -2.77)
Serbia	55 (28 to 94)	13 (4 to 24)	-77.28% (-89.21 to -63.37)	17.7 (8.9 to 30.1)	4.9 (1.7 to 9.3)	-5.08% (-5.41 to -4.75)
Sierra Leone	817 (424 to 1371)	1708 (909 to 2854)	109% (1.51 to 329.44)	489.9 (254.4 to 821.9)	402.3 (214.2 to 672.3)	-0.55% (-1.3 to 0.21)
Solomon Islands	94 (45 to 178)	108 (49 to 195)	15.64% (-51.1 to 174.41)	603.1 (291.9 to 1144.6)	375.6 (169.5 to 675.9)	-1.14% (-1.71 to -0.58)
Somalia	3037 (1450 to 5187)	11082 (6304 to 18403)	264.9% (103.81 to 622.97)	1336.9 (638.4 to 2283.5)	1226.4 (697.6 to 2036.6)	-0.02% (-0.3 to 0.25)
South Africa	3459 (2176 to 5079)	768 (392 to 1280)	-77.81% (-89.59 to -54.53)	181.3 (114 to 266.1)	33.5 (17.1 to 55.9)	-4.78% (-6.57 to -2.96)
South Sudan	857 (414 to 1571)	905 (430 to 1807)	5.65% (-51.55 to 151.42)	316.1 (152.6 to 579.7)	218.1 (103.7 to 435.2)	-1.43% (-1.65 to -1.2)

Location	DALYs in 1990 (95% UI)	DALYs in 2019 (95% UI)	Relative change (%, 95 CI)	Age-standardized DALYs rate in 1990 (95% UI)	Age-standardized DALYs rate in 2019 (95% UI)	EAPC (%, 95 CI)
Sri Lanka	301 (171 to 496)	59 (28 to 105)	-80.32% (-90.65 to -63.79)	37.2 (21.2 to 61.5)	7.3 (3.5 to 12.9)	-5.39% (-5.54 to -5.23)
Sudan	8713 (4987 to 13846)	2627 (1253 to 4668)	-69.85% (-87.08 to -34.53)	929.9 (532.3 to 1477.8)	135.1 (64.5 to 240.1)	-6.4% (-6.92 to -5.87)
Suriname	10 (5 to 17)	6 (3 to 10)	-40.14% (-68.33 to 14.78)	53.4 (28.8 to 88.6)	27 (15 to 43.2)	-2.52% (-3.03 to -2)
Syrian Arab Republic	552 (314 to 875)	83 (40 to 143)	-85% (-92.99 to -72.63)	96.8 (55.1 to 153.5)	11 (5.3 to 19)	-7.86% (-8.33 to -7.38)
Tajikistan	99 (53 to 168)	103 (43 to 186)	4.19% (-32.15 to 40.55)	40 (21.6 to 68.2)	23.7 (10 to 42.8)	-2.05% (-2.16 to -1.93)
Thailand	571 (304 to 979)	188 (88 to 323)	-67.08% (-82.28 to -43.09)	19.6 (10.4 to 33.6)	7.7 (3.6 to 13.3)	-3.27% (-3.52 to -3.03)
Timor-Leste	250 (108 to 455)	93 (22 to 168)	-62.96% (-89.83 to -3.19)	689.7 (299 to 1256.9)	139.8 (33.1 to 253.6)	-6.81% (-7.6 to -6)
Togo	753 (399 to 1274)	578 (278 to 1012)	-23.24% (-65.9 to 62.75)	446.8 (236.8 to 755.6)	164.7 (79.3 to 288.2)	-2.99% (-3.45 to -2.53)
Tonga	3 (1 to 5)	1 (1 to 3)	-51.16% (-77.43 to 1.45)	63.9 (33.5 to 107.3)	31.2 (15.6 to 57.7)	-2.4% (-2.5 to -2.3)

Location	DALYs in 1990 (95% UI)	DALYs in 2019 (95% UI)	Relative change (%, 95 CI)	Age-standardized DALYs rate in 1990 (95% UI)	Age-standardized DALYs rate in 2019 (95% UI)	EAPC (%, 95 CI)
Tunisia	342 (201 to 565)	39 (20 to 67)	-88.61% (-94.21 to -78.75)	85 (49.8 to 140.2)	9.6 (4.9 to 16.5)	-7.02% (-7.37 to -6.67)
Turkey	3937 (2194 to 6228)	449 (244 to 739)	-88.59% (-94.19 to -79.06)	143.6 (80 to 227.1)	13.8 (7.5 to 22.6)	-8.95% (-9.59 to -8.3)
Turkmenistan	54 (29 to 95)	40 (19 to 70)	-25.87% (-48.33 to 1.24)	31.6 (16.8 to 55.5)	20.8 (9.6 to 36.7)	-1.48% (-1.69 to -1.27)
Tuvalu	1 (1 to 2)	0 (0 to 0)	-78.93% (-90.91 to -50.05)	258.3 (125.9 to 453.2)	43.8 (21.6 to 80.4)	-5.64% (-5.88 to -5.41)
Uganda	2614 (1057 to 4684)	3551 (1759 to 6135)	35.89% (-41.08 to 232.78)	315.3 (127.6 to 565.1)	176.3 (87.3 to 304.7)	-2.37% (-3 to -1.73)
Ukraine	492 (278 to 817)	133 (65 to 233)	-73.01% (-82.32 to -61.17)	28.9 (16.3 to 48)	12.3 (6 to 21.5)	-2.75% (-3.11 to -2.39)
United Republic of Tanzania	5538 (3147 to 9189)	4538 (2350 to 7530)	-18.05% (-61.93 to 71.25)	447.5 (254.3 to 742.6)	166.4 (86.1 to 276.1)	-2.72% (-3.09 to -2.35)
Uzbekistan	617 (392 to 946)	580 (334 to 902)	-5.99% (-37.99 to 38.55)	64.9 (41.2 to 99.5)	39.6 (22.8 to 61.6)	-1.68% (-2.2 to -1.15)
Vanuatu	10 (5 to 19)	13 (6 to 24)	28.31% (-47.44 to 196.82)	151.6 (71.4 to 278.6)	96 (44.5 to 176.2)	-2.28% (-2.6 to -1.96)

Location	DALYs in 1990 (95% UI)	DALYs in 2019 (95% UI)	Relative change (%, 95 CI)	Age-standardized DALYs rate in 1990 (95% UI)	Age-standardized DALYs rate in 2019 (95% UI)	EAPC (%, 95 CI)
Venezuela (Bolivarian Republic of)	1296 (847 to 1812)	689 (401 to 1110)	-46.84% (-71.07 to -0.95)	143.8 (94 to 201.1)	65.5 (38.2 to 105.6)	-2.84% (-3.91 to -1.76)
Viet Nam	1374 (707 to 2373)	288 (111 to 579)	-79.01% (-91.06 to -59.96)	41.3 (21.2 to 71.3)	8.1 (3.1 to 16.2)	-5.31% (-5.62 to -5)
Yemen	3264 (1161 to 6470)	4016 (1634 to 7245)	23.06% (-50.67 to 295.82)	654.9 (233 to 1298.4)	298.3 (121.3 to 538.1)	-3.33% (-3.62 to -3.04)
Zambia	1641 (909 to 2724)	974 (514 to 1695)	-40.64% (-71.37 to 22.75)	412.1 (228.4 to 684.3)	105.2 (55.5 to 183.1)	-5.05% (-5.73 to -4.37)
Zimbabwe	1753 (1071 to 2683)	3035 (1662 to 4988)	73.14% (-6.6 to 232.78)	354.4 (216.6 to 542.5)	414.2 (226.8 to 680.7)	4.09% (2.51 to 5.68)

CI – confidence interval, DALYs – disability-adjusted life years (per 100,000 population), EAPC – estimated annual percentage change, MSMI – maternal sepsis and other maternal infections, UI – uncertain interval.

Table S28. DALYs of MSMI in 131 low- and middle-income countries and territories with EAPC in age group of 25-29 years from 1990 and 2019.

Location	DALYs in 1990 (95% UI)	DALYs in 2019 (95% UI)	Relative change (%, 95 CI)	Age-standardized DALYs rate in 1990 (95% UI)	Age-standardized DALYs rate in 2019 (95% UI)	EAPC (%, 95 CI)
Afghanistan	3128 (1636 to 5354)	4717 (2365 to 8189)	50.84% (-24.54 to 213.06)	908.1 (474.9 to 1554.5)	304.4 (152.6 to 528.4)	-4.25% (-5.39 to -3.1)
Albania	26 (14 to 42)	9 (4 to 17)	-63.27% (-78.58 to -44.88)	17 (9.3 to 28)	9.2 (4.2 to 16.8)	-1.82% (-2.06 to -1.58)
Algeria	4707 (2508 to 8250)	515 (292 to 825)	-89.07% (-94.77 to -78.6)	478.8 (255.1 to 839)	28.7 (16.3 to 46)	-9.46% (-9.89 to -9.03)
American Samoa	1 (1 to 2)	0 (0 to 1)	-65.13% (-83.78 to -26.08)	54.1 (28.4 to 92)	21.2 (11.7 to 36.1)	-3.62% (-3.86 to -3.38)
Angola	2121 (1029 to 3875)	1690 (881 to 2784)	-20.32% (-64.61 to 72.15)	541.3 (262.6 to 988.9)	144.5 (75.4 to 238.1)	-4.35% (-4.75 to -3.95)
Armenia	38 (23 to 59)	14 (7 to 24)	-64.5% (-80.92 to -44.98)	23.1 (14.1 to 35.5)	10.6 (5.4 to 18.3)	-2.49% (-3.11 to -1.86)
Azerbaijan	161 (96 to 247)	41 (18 to 76)	-74.51% (-87.69 to -55.63)	41.8 (25 to 64.2)	8.3 (3.6 to 15.3)	-6.89% (-7.35 to -6.42)
Bangladesh	5697 (3294 to 9269)	548 (279 to 962)	-90.38% (-95.62 to -79.49)	134.1 (77.5 to 218.1)	7.5 (3.8 to 13.2)	-9.29% (-10.61 to -7.94)

Location	DALYs in 1990 (95% UI)	DALYs in 2019 (95% UI)	Relative change (%, 95 CI)	Age-standardized DALYs rate in 1990 (95% UI)	Age-standardized DALYs rate in 2019 (95% UI)	EAPC (%, 95 CI)
Belarus	58 (32 to 101)	38 (16 to 74)	-34.26% (-62.8 to -1.76)	13.6 (7.5 to 23.6)	12.5 (5.2 to 24.3)	0.69% (0.13 to 1.26)
Belize	5 (3 to 7)	8 (5 to 13)	69% (-4.61 to 212.09)	68.2 (43.6 to 100.7)	46.5 (29 to 72.7)	-0.85% (-1.3 to -0.4)
Benin	1701 (960 to 2732)	1422 (728 to 2554)	-16.4% (-61.26 to 73.32)	848 (478.8 to 1361.9)	280.6 (143.6 to 504)	-3.22% (-3.51 to -2.93)
Bhutan	35 (18 to 58)	4 (2 to 6)	-88.97% (-94.85 to -76.21)	149.7 (76.4 to 249.7)	11 (5.7 to 18.4)	-9.17% (-9.51 to -8.82)
Bolivia (Plurinational State of)	1871 (1052 to 3027)	526 (252 to 920)	-71.87% (-87.25 to -44.4)	761.7 (428.2 to 1232.4)	104.1 (49.8 to 182.1)	-7.09% (-7.41 to -6.76)
Bosnia and Herzegovina	62 (38 to 97)	5 (2 to 11)	-91.65% (-96.89 to -83.36)	32.2 (19.5 to 50.1)	5.2 (1.9 to 10.7)	-7.4% (-8.98 to -5.8)
Botswana	194 (92 to 338)	65 (30 to 126)	-66.29% (-85.47 to -19.5)	358.1 (169.4 to 623.7)	58.9 (27.3 to 113.6)	-6.36% (-7.14 to -5.56)
Brazil	5541 (3596 to 8219)	2522 (1707 to 3601)	-54.49% (-73.24 to -22.86)	84.3 (54.7 to 125.1)	29.1 (19.7 to 41.6)	-2.58% (-3.07 to -2.09)
Bulgaria	51 (31 to 77)	13 (6 to 23)	-74.99% (-88.37 to -54.02)	18.2 (11.2 to 27.4)	6.7 (3 to 12.3)	-3.75% (-4.21 to -3.29)

Location	DALYs in 1990 (95% UI)	DALYs in 2019 (95% UI)	Relative change (%, 95 CI)	Age-standardized DALYs rate in 1990 (95% UI)	Age-standardized DALYs rate in 2019 (95% UI)	EAPC (%, 95 CI)
Burkina Faso	2725 (1458 to 4518)	2367 (1146 to 4183)	-13.12% (-58.93 to 79.47)	782 (418.3 to 1296.5)	268.9 (130.1 to 475)	-4.2% (-4.61 to -3.78)
Burundi	3308 (1741 to 5634)	3725 (2183 to 5910)	12.6% (-40.33 to 130.98)	1481 (779.4 to 2521.8)	806.4 (472.6 to 1279.3)	-2.44% (-2.95 to -1.94)
Cabo Verde	22 (12 to 36)	2 (1 to 4)	-89.34% (-95.1 to -78.41)	151.4 (84.8 to 248)	9.3 (4.8 to 15.2)	-10.06% (-10.33 to -9.79)
Cambodia	3768 (1915 to 6860)	557 (286 to 1002)	-85.23% (-93.55 to -66.88)	880.2 (447.3 to 1602.6)	73.4 (37.7 to 132.1)	-9.59% (-10.59 to -8.58)
Cameroon	2498 (1446 to 3931)	2815 (1328 to 4906)	12.71% (-47.16 to 134.11)	607.4 (351.7 to 956)	226.1 (106.7 to 394)	-3.22% (-3.72 to -2.72)
Central African Republic	1635 (886 to 2640)	2905 (1540 to 4966)	77.7% (-13.29 to 252.57)	1462 (792.7 to 2361.5)	1378.4 (730.9 to 2356.5)	-0.09% (-0.28 to 0.1)
Chad	3129 (1802 to 4801)	4425 (2373 to 6976)	41.39% (-25.15 to 170.62)	1447 (833 to 2220.1)	770.5 (413.3 to 1214.9)	-2.13% (-2.59 to -1.68)
China	32715 (24579 to 42819)	2813 (1324 to 5340)	-91.4% (-95.73 to -85.1)	61 (45.8 to 79.8)	5.2 (2.4 to 9.8)	-8.39% (-9.23 to -7.55)

Location	DALYs in 1990 (95% UI)	DALYs in 2019 (95% UI)	Relative change (%, 95 CI)	Age-standardized DALYs rate in 1990 (95% UI)	Age-standardized DALYs rate in 2019 (95% UI)	EAPC (%, 95 CI)
Colombia	983 (599 to 1532)	359 (201 to 572)	-63.44% (-81.9 to -30.46)	64 (39 to 99.8)	17.9 (10 to 28.5)	-4.22% (-4.66 to -3.78)
Comoros	76 (15 to 148)	47 (19 to 88)	-38.36% (-77.15 to 248.13)	443.6 (85.7 to 863.5)	157.3 (62.3 to 296.5)	-4.35% (-5.05 to -3.63)
Congo	471 (202 to 824)	338 (158 to 601)	-28.3% (-70.15 to 79.43)	504.5 (216.6 to 882.5)	159.6 (74.7 to 283.7)	-3.66% (-4.5 to -2.81)
Costa Rica	61 (40 to 87)	18 (10 to 30)	-70.44% (-84.03 to -48.28)	43.6 (28.2 to 62.1)	8.7 (4.7 to 14.2)	-5.77% (-6.79 to -4.73)
Côte d'Ivoire	1961 (1060 to 3237)	1890 (922 to 3390)	-3.65% (-57.22 to 100.44)	394 (212.9 to 650.3)	173.1 (84.4 to 310.4)	-2.38% (-2.89 to -1.88)
Cuba	238 (152 to 353)	54 (33 to 83)	-77.41% (-87.33 to -60.27)	43.5 (27.9 to 64.6)	14.5 (8.9 to 22.4)	-3.64% (-4.12 to -3.16)
Democratic People's Republic of Korea	763 (385 to 1360)	545 (269 to 977)	-28.5% (-67.65 to 60.53)	83.4 (42.1 to 148.7)	51.5 (25.4 to 92.4)	-1.42% (-2.28 to -0.56)
Democratic Republic of the Congo	13288 (7155 to 22465)	29911 (17427 to 45878)	125.09% (15.9 to 327.41)	920.8 (495.8 to 1556.6)	878.7 (512 to 1347.8)	1.35% (0.16 to 2.56)

Location	DALYs in 1990 (95% UI)	DALYs in 2019 (95% UI)	Relative change (%, 95 CI)	Age-standardized DALYs rate in 1990 (95% UI)	Age-standardized DALYs rate in 2019 (95% UI)	EAPC (%, 95 CI)
Djibouti	151 (76 to 246)	199 (87 to 362)	31.68% (-43.87 to 188.65)	875.5 (441.8 to 1424.8)	405.9 (178 to 737.5)	-2.39% (-2.88 to -1.9)
Dominica	4 (2 to 6)	2 (1 to 4)	-45.52% (-75.8 to 16.25)	141.1 (77.4 to 225.9)	85.4 (45.1 to 146.1)	-1.67% (-1.97 to -1.37)
Dominican Republic	247 (147 to 381)	183 (97 to 303)	-25.64% (-63.52 to 46.8)	75.7 (45.2 to 116.8)	39.3 (20.9 to 65)	-1.71% (-2.28 to -1.13)
Ecuador	603 (365 to 910)	236 (136 to 380)	-60.79% (-80.16 to -27.34)	142.8 (86.4 to 215.7)	32.6 (18.7 to 52.3)	-4.48% (-5.42 to -3.54)
Egypt	4852 (2818 to 7534)	686 (375 to 1101)	-85.87% (-93.06 to -71.79)	228.5 (132.7 to 354.8)	16.9 (9.3 to 27.2)	-9% (-9.62 to -8.39)
El Salvador	827 (472 to 1255)	54 (25 to 97)	-93.5% (-96.94 to -86.9)	385.2 (219.8 to 584.5)	18.6 (8.8 to 33.5)	-9.74% (-10.99 to -8.48)
Equatorial Guinea	277 (151 to 442)	29 (13 to 54)	-89.57% (-95.7 to -75.87)	1755.6 (959.8 to 2803.6)	50.8 (23.4 to 95.6)	-12.87% (-13.57 to -12.16)
Eritrea	2517 (1376 to 4075)	1975 (1033 to 3450)	-21.54% (-61.08 to 60.07)	2229.2 (1218.2 to 3608.8)	708.1 (370.4 to 1236.9)	-2.96% (-3.56 to -2.35)
Eswatini	54 (29 to 100)	26 (11 to 53)	-51.87% (-80.55 to 14.73)	165.6 (87.1 to 304.2)	47.6 (19.8 to 95.9)	-3.15% (-4.24 to -2.05)

Location	DALYs in 1990 (95% UI)	DALYs in 2019 (95% UI)	Relative change (%, 95 CI)	Age-standardized DALYs rate in 1990 (95% UI)	Age-standardized DALYs rate in 2019 (95% UI)	EAPC (%, 95 CI)
Ethiopia	45100 (26131 to 70865)	13305 (7139 to 21377)	-70.5% (-85.76 to -43.15)	2297.8 (1331.4 to 3610.6)	306.5 (164.5 to 492.4)	-7.11% (-7.97 to -6.24)
Fiji	14 (8 to 26)	8 (4 to 13)	-47.12% (-76.97 to 20.2)	43.6 (22.8 to 78.5)	22 (12.1 to 37.8)	-2.76% (-3.01 to -2.52)
Gabon	135 (69 to 231)	47 (23 to 83)	-65.42% (-83.4 to -20.88)	351.3 (179.4 to 600.9)	57.5 (27.8 to 102.6)	-6.47% (-7.12 to -5.81)
Gambia	268 (141 to 444)	310 (170 to 514)	15.53% (-41.64 to 129.28)	631.9 (333.3 to 1046.8)	318.4 (175.1 to 528.8)	-2.57% (-2.97 to -2.17)
Georgia	46 (28 to 73)	27 (16 to 42)	-42.56% (-67.9 to 0.22)	19.1 (11.3 to 29.8)	21.4 (12.7 to 33.6)	2.36% (1.1 to 3.64)
Ghana	2284 (1210 to 3813)	1027 (521 to 1830)	-55.02% (-79.06 to -6.5)	369.4 (195.7 to 616.6)	69.3 (35.1 to 123.4)	-5.13% (-5.46 to -4.8)
Global	523802 (426844 to 632636)	221685 (182076 to 267770)	-57.68% (-67.07 to -46.65)	238 (193.9 to 287.4)	73.7 (60.6 to 89.1)	-4.03% (-4.44 to -3.62)
Grenada	1 (1 to 1)	0 (0 to 1)	-66.31% (-82.83 to -40.73)	30.2 (18.8 to 45.1)	7.6 (3.9 to 13.5)	-3.43% (-4.02 to -2.83)
Guatemala	1096 (645 to 1772)	363 (205 to 587)	-66.91% (-82.72 to -39.23)	384 (225.9 to 620.8)	42.9 (24.3 to 69.4)	-8.31% (-9.15 to -7.48)

Location	DALYs in 1990 (95% UI)	DALYs in 2019 (95% UI)	Relative change (%, 95 CI)	Age-standardized DALYs rate in 1990 (95% UI)	Age-standardized DALYs rate in 2019 (95% UI)	EAPC (%, 95 CI)
Guinea	2709 (1532 to 4375)	2855 (1521 to 4863)	5.37% (-45.24 to 103.6)	1136.1 (642.5 to 1834.8)	539.5 (287.4 to 919.1)	-2.02% (-2.21 to -1.84)
Guinea-Bissau	255 (132 to 433)	149 (72 to 262)	-41.63% (-73.75 to 25.29)	643.6 (334.7 to 1095.6)	169.3 (81.7 to 298.4)	-4.15% (-4.5 to -3.79)
Guyana	38 (21 to 61)	16 (9 to 27)	-58.69% (-79.91 to -17.15)	106.8 (60 to 170.7)	45 (24.6 to 76.1)	-3.23% (-3.71 to -2.74)
Haiti	2974 (1517 to 5152)	4165 (2282 to 6728)	40.05% (-30.58 to 197.23)	1163.4 (593.3 to 2015.5)	732.7 (401.5 to 1183.7)	-0.91% (-1.24 to -0.58)
Honduras	1714 (1030 to 2603)	228 (108 to 434)	-86.7% (-94.03 to -71.08)	997.6 (599.2 to 1514.5)	50.2 (23.8 to 95.5)	-10.41% (-10.88 to -9.94)
India	195551 (116665 to 291492)	31860 (18561 to 50740)	-83.71% (-91.39 to -69.32)	588.7 (351.2 to 877.5)	54.9 (32 to 87.5)	-8.46% (-9.18 to -7.73)
Indonesia	19021 (10743 to 30428)	3056 (1890 to 4716)	-83.94% (-91.5 to -69.55)	231.7 (130.9 to 370.7)	30 (18.5 to 46.2)	-6.57% (-6.83 to -6.29)
Iran (Islamic Republic of)	2289 (1527 to 3282)	372 (225 to 582)	-83.77% (-90.51 to -71.78)	104.2 (69.5 to 149.4)	10.7 (6.5 to 16.7)	-7.75% (-8.3 to -7.2)
Iraq	194 (108 to 321)	196 (96 to 352)	1.24% (-44.36 to 61.09)	32.2 (17.9 to 53.3)	10.1 (4.9 to 18)	-4.29% (-4.68 to -3.9)

Location	DALYs in 1990 (95% UI)	DALYs in 2019 (95% UI)	Relative change (%, 95 CI)	Age-standardized DALYs rate in 1990 (95% UI)	Age-standardized DALYs rate in 2019 (95% UI)	EAPC (%, 95 CI)
Jamaica	35 (22 to 53)	12 (6 to 19)	-66.58% (-82.37 to -38.74)	33.2 (20.5 to 50.7)	9 (5 to 14.5)	-6.62% (-7.61 to -5.62)
Jordan	223 (124 to 368)	92 (52 to 153)	-58.94% (-79.93 to -14.13)	164.6 (91.5 to 271)	20 (11.3 to 33.4)	-8.38% (-8.96 to -7.8)
Kazakhstan	95 (45 to 176)	113 (58 to 201)	19.33% (-18.95 to 82.36)	12.6 (6 to 23.4)	14.4 (7.4 to 25.6)	1.19% (0.83 to 1.55)
Kenya	3690 (2185 to 5879)	3108 (1705 to 5148)	-15.79% (-56.58 to 55.29)	414.4 (245.3 to 660.2)	145.2 (79.7 to 240.6)	-3.32% (-3.86 to -2.77)
Kiribati	8 (4 to 15)	6 (3 to 11)	-17.13% (-67.72 to 105.76)	227.5 (107.2 to 439)	119.5 (57.1 to 212)	-2.4% (-2.59 to -2.21)
Kyrgyzstan	71 (45 to 108)	87 (54 to 132)	22.14% (-24.09 to 102.65)	35.9 (22.6 to 54.5)	29.2 (18.3 to 44.4)	0.6% (-0.13 to 1.33)
Lao People's Democratic Republic	1856 (1105 to 2989)	179 (97 to 300)	-90.35% (-95.45 to -81.07)	1173.3 (698.4 to 1889.6)	54.4 (29.4 to 90.9)	-10.45% (-11.09 to -9.8)
Lebanon	63 (34 to 108)	23 (11 to 40)	-63.4% (-82.49 to -33.05)	50.7 (27.3 to 86.7)	10.2 (5 to 17.8)	-5.45% (-5.94 to -4.96)
Lesotho	412 (213 to 660)	265 (119 to 509)	-35.69% (-73.45 to 39.23)	579.5 (300 to 929.3)	273.6 (123.1 to 525.7)	-0.36% (-1.34 to 0.63)

Location	DALYs in 1990 (95% UI)	DALYs in 2019 (95% UI)	Relative change (%, 95 CI)	Age-standardized DALYs rate in 1990 (95% UI)	Age-standardized DALYs rate in 2019 (95% UI)	EAPC (%, 95 CI)
Liberia	834 (468 to 1324)	1317 (703 to 2163)	57.81% (-21.02 to 199.84)	1191.7 (668 to 1891.2)	670.3 (357.8 to 1101)	-3.18% (-4.17 to -2.17)
Libya	41 (22 to 67)	20 (9 to 36)	-51.86% (-77.32 to -17.51)	29.5 (15.6 to 47.3)	6.8 (3.1 to 12.3)	-4.17% (-4.8 to -3.53)
Madagascar	3649 (2055 to 5825)	4204 (2197 to 7082)	15.23% (-40.91 to 128.24)	807.2 (454.6 to 1288.7)	388.9 (203.3 to 655.1)	-2.42% (-2.79 to -2.05)
Malawi	1814 (994 to 2982)	1297 (642 to 2256)	-28.5% (-67.79 to 49.82)	473 (259.3 to 777.6)	177.7 (87.9 to 309.3)	-3.25% (-3.75 to -2.75)
Malaysia	861 (481 to 1429)	220 (116 to 369)	-74.47% (-88.16 to -47.04)	112.5 (62.9 to 186.8)	15.8 (8.3 to 26.5)	-6.96% (-7.46 to -6.45)
Maldives	11 (6 to 18)	2 (1 to 3)	-81.91% (-91.82 to -64.07)	127.4 (71.2 to 214.3)	9.9 (5.1 to 16.8)	-8.43% (-9.37 to -7.48)
Mali	6822 (4273 to 10275)	4476 (2438 to 7803)	-34.39% (-65.87 to 22.12)	2131.4 (1335 to 3210.3)	541.5 (294.9 to 944)	-4.91% (-5.19 to -4.63)
Marshall Islands	3 (1 to 5)	2 (1 to 3)	-36.88% (-78.95 to 41.53)	156.2 (80.6 to 285.9)	72.8 (28.9 to 137.7)	-2.8% (-3.45 to -2.15)
Mauritania	1458 (879 to 2184)	354 (177 to 627)	-75.74% (-88.01 to -53.03)	1866.1 (1125.7 to 2796.4)	218.7 (109.4 to 387.7)	-7.76% (-8.03 to -7.49)

Location	DALYs in 1990 (95% UI)	DALYs in 2019 (95% UI)	Relative change (%, 95 CI)	Age-standardized DALYs rate in 1990 (95% UI)	Age-standardized DALYs rate in 2019 (95% UI)	EAPC (%, 95 CI)
Mexico	1722 (1133 to 2522)	826 (479 to 1339)	-52.04% (-73.23 to -21.37)	48.2 (31.7 to 70.6)	16 (9.3 to 26)	-3.43% (-3.82 to -3.04)
Micronesia (Federated States of)	11 (5 to 20)	3 (1 to 6)	-72.37% (-91.59 to -30.76)	285.2 (130.5 to 540.9)	73.2 (26.5 to 152.7)	-4.81% (-5.11 to -4.49)
Mongolia	370 (192 to 624)	74 (39 to 130)	-79.91% (-90.59 to -56.97)	383.8 (199.1 to 647.9)	49.5 (25.9 to 86.3)	-8.14% (-8.55 to -7.72)
Montenegro	2 (1 to 4)	1 (0 to 2)	-34.85% (-64.76 to 8.7)	7.6 (3.1 to 14.8)	6.2 (2.3 to 12.2)	-0.84% (-0.9 to -0.77)
Morocco	3982 (2177 to 6470)	315 (164 to 557)	-92.09% (-96.36 to -83.8)	374.1 (204.6 to 607.9)	21.1 (11 to 37.2)	-9.64% (-9.92 to -9.35)
Mozambique	3021 (1616 to 5103)	1659 (806 to 3034)	-45.1% (-74.91 to 17.37)	657.5 (351.6 to 1110.4)	141.3 (68.7 to 258.4)	-4.79% (-5.24 to -4.33)
Myanmar	7152 (3792 to 12429)	1612 (866 to 2862)	-77.46% (-89.27 to -49.34)	410.6 (217.7 to 713.6)	70.9 (38.1 to 126)	-6.49% (-7.02 to -5.96)
Namibia	164 (72 to 290)	63 (28 to 124)	-61.54% (-83.89 to 1)	288.3 (126 to 510.3)	57.6 (25.2 to 112.9)	-5.1% (-5.63 to -4.56)
Nepal	6508 (3438 to 10581)	517 (266 to 876)	-92.06% (-96.43 to -83.86)	852 (450.1 to 1385.2)	35.6 (18.4 to 60.4)	-10.73% (-10.92 to -10.54)

Location	DALYs in 1990 (95% UI)	DALYs in 2019 (95% UI)	Relative change (%, 95 CI)	Age-standardized DALYs rate in 1990 (95% UI)	Age-standardized DALYs rate in 2019 (95% UI)	EAPC (%, 95 CI)
Nicaragua	233 (141 to 355)	38 (20 to 60)	-83.83% (-92.41 to -69.22)	156.2 (94.5 to 237.8)	12.9 (6.9 to 20.6)	-9.11% (-9.68 to -8.54)
Niger	4425 (2434 to 6946)	6814 (3713 to 11246)	53.98% (-19.98 to 184.96)	1532.6 (842.9 to 2405.6)	890.4 (485.2 to 1469.4)	-2.13% (-2.41 to -1.85)
Nigeria	11688 (6092 to 21171)	11921 (5993 to 21730)	1.99% (-53.69 to 111.1)	345 (179.8 to 625)	138.5 (69.6 to 252.5)	-3.95% (-4.73 to -3.16)
North Macedonia	12 (7 to 19)	4 (2 to 8)	-65.15% (-83 to -40.49)	15.4 (8.8 to 24.3)	5.4 (2.3 to 10.6)	-3.75% (-4.14 to -3.35)
Pakistan	19706 (11804 to 30367)	10262 (5752 to 16441)	-47.93% (-74.72 to 1.49)	542.6 (325.1 to 836.2)	118 (66.1 to 189)	-5.57% (-5.99 to -5.14)
Papua New Guinea	1066 (593 to 1784)	1061 (560 to 1816)	-0.52% (-50.68 to 100.62)	642.3 (357.1 to 1074.5)	253.7 (133.9 to 434.3)	-2.44% (-2.91 to -1.96)
Paraguay	402 (239 to 630)	86 (46 to 147)	-78.55% (-89.74 to -57.23)	255.4 (151.4 to 399.7)	28.4 (15.2 to 48.4)	-8.13% (-8.67 to -7.57)
Peru	2300 (1321 to 3589)	337 (176 to 600)	-85.34% (-92.66 to -69.84)	255.4 (146.7 to 398.5)	23.8 (12.4 to 42.3)	-8.77% (-9.2 to -8.34)
Philippines	2460 (1525 to 3654)	1220 (725 to 1915)	-50.4% (-73.02 to -11.99)	95.4 (59.1 to 141.6)	26.1 (15.5 to 40.9)	-4.29% (-4.45 to -4.13)

Location	DALYs in 1990 (95% UI)	DALYs in 2019 (95% UI)	Relative change (%, 95 CI)	Age-standardized DALYs rate in 1990 (95% UI)	Age-standardized DALYs rate in 2019 (95% UI)	EAPC (%, 95 CI)
Republic of Moldova	59 (37 to 88)	12 (5 to 23)	-79.71% (-89.97 to -67.2)	31.8 (19.6 to 46.9)	9.2 (3.9 to 17.6)	-4.62% (-5.23 to -4)
Russian Federation	1189 (761 to 1750)	613 (309 to 1127)	-48.47% (-68.82 to -29.24)	19.6 (12.6 to 28.9)	13.6 (6.9 to 25)	-1.13% (-1.32 to -0.95)
Rwanda	5319 (3206 to 8342)	1470 (720 to 2691)	-72.37% (-87.49 to -45.45)	1960.6 (1181.8 to 3075)	292.9 (143.4 to 536.1)	-6.92% (-7.79 to -6.05)
Saint Lucia	2 (1 to 3)	1 (0 to 1)	-64.01% (-80.52 to -36.87)	34.4 (21.9 to 52.4)	10.5 (6.2 to 16.6)	-3.12% (-3.99 to -2.24)
Saint Vincent and the Grenadines	1 (0 to 1)	0 (0 to 1)	-41.02% (-64 to -5.14)	12.9 (7.2 to 22.1)	8.6 (4.6 to 14.7)	-1.64% (-1.99 to -1.29)
Samoa	5 (3 to 10)	1 (0 to 2)	-76.44% (-91.88 to -41.13)	89.4 (44.3 to 160)	16.4 (6.4 to 31)	-6.74% (-7.24 to -6.23)
Senegal	2080 (1171 to 3353)	1521 (786 to 2600)	-26.87% (-65.5 to 48.28)	728.2 (409.8 to 1173.6)	243.8 (126 to 416.8)	-3.45% (-3.76 to -3.13)
Serbia	35 (19 to 58)	12 (5 to 23)	-67.48% (-85.88 to -41)	10.7 (5.9 to 17.5)	4.2 (1.8 to 8.2)	-3.43% (-3.76 to -3.09)
Sierra Leone	918 (490 to 1528)	1746 (923 to 2927)	90.08% (-11.01 to 314.37)	614.5 (327.6 to 1022.2)	476.6 (252 to 799.2)	-0.67% (-1.5 to 0.16)

Location	DALYs in 1990 (95% UI)	DALYs in 2019 (95% UI)	Relative change (%, 95 CI)	Age-standardized DALYs rate in 1990 (95% UI)	Age-standardized DALYs rate in 2019 (95% UI)	EAPC (%, 95 CI)
Solomon Islands	85 (37 to 161)	106 (47 to 202)	24.26% (-49.27 to 196.56)	705 (304.7 to 1335.3)	412.8 (183 to 789.7)	-1.31% (-1.97 to -0.65)
Somalia	4088 (2082 to 6985)	11345 (6051 to 19815)	177.51% (38.74 to 462.93)	1888.6 (961.9 to 3226.9)	1597.4 (852 to 2790)	-0.44% (-0.67 to -0.21)
South Africa	4369 (2648 to 6716)	1300 (573 to 2409)	-70.24% (-88.49 to -32.78)	259.7 (157.4 to 399.1)	50.2 (22.1 to 93)	-5.31% (-7.52 to -3.04)
South Sudan	898 (403 to 1672)	684 (308 to 1265)	-23.76% (-68.36 to 85.99)	410.6 (184.2 to 764.9)	251.6 (113.4 to 465.1)	-1.82% (-2.04 to -1.61)
Sri Lanka	243 (143 to 385)	72 (36 to 132)	-70.35% (-85.91 to -47.1)	31.9 (18.8 to 50.6)	9 (4.6 to 16.5)	-4.09% (-4.31 to -3.87)
Sudan	8976 (5251 to 14119)	2338 (987 to 4353)	-73.95% (-89.49 to -43.74)	1188.6 (695.3 to 1869.6)	133 (56.1 to 247.5)	-7.35% (-7.82 to -6.89)
Suriname	8 (4 to 13)	7 (4 to 12)	-17.34% (-63.66 to 90.27)	49.1 (25.7 to 81.4)	31.4 (16.8 to 54)	-1.42% (-2.11 to -0.74)
Syrian Arab Republic	487 (266 to 830)	41 (21 to 68)	-91.59% (-96.08 to -82.85)	106.3 (58.1 to 181.3)	11.3 (5.8 to 18.8)	-8.11% (-8.81 to -7.4)
Tajikistan	74 (40 to 120)	81 (38 to 148)	8.64% (-33.23 to 56.82)	32 (17.4 to 51.5)	18.5 (8.7 to 33.9)	-2.32% (-2.6 to -2.05)

Location	DALYs in 1990 (95% UI)	DALYs in 2019 (95% UI)	Relative change (%, 95 CI)	Age-standardized DALYs rate in 1990 (95% UI)	Age-standardized DALYs rate in 2019 (95% UI)	EAPC (%, 95 CI)
Thailand	459 (243 to 780)	196 (100 to 338)	-57.33% (-79.64 to -17.42)	16.9 (8.9 to 28.6)	8.1 (4.1 to 13.9)	-2.83% (-3.73 to -1.92)
Timor-Leste	302 (144 to 518)	82 (14 to 151)	-72.84% (-95.77 to -34.44)	886.2 (422.6 to 1520.7)	151.9 (25.9 to 279.7)	-7.24% (-7.93 to -6.54)
Togo	979 (551 to 1585)	763 (368 to 1351)	-22.04% (-63.1 to 58.45)	667.1 (375.4 to 1080.3)	228.2 (110 to 403.9)	-3.27% (-3.76 to -2.79)
Tonga	2 (1 to 4)	1 (1 to 2)	-51.64% (-78.95 to 3.92)	71.3 (38.3 to 124.4)	31.7 (16.4 to 55.2)	-2.72% (-2.88 to -2.57)
Tunisia	326 (180 to 548)	53 (27 to 89)	-83.87% (-92.13 to -66.86)	92.4 (50.9 to 155.4)	11.3 (5.8 to 19.1)	-6.26% (-6.84 to -5.68)
Turkey	3709 (2043 to 6245)	475 (255 to 810)	-87.2% (-93.99 to -75.22)	153.7 (84.7 to 258.8)	14.1 (7.6 to 24)	-9.18% (-10.02 to -8.33)
Turkmenistan	50 (28 to 85)	36 (18 to 66)	-28.47% (-51.6 to 1.41)	29.4 (16.5 to 49.6)	17.5 (8.6 to 32)	-1.81% (-2.03 to -1.59)
Tuvalu	1 (0 to 2)	0 (0 to 0)	-82.84% (-92.55 to -60.4)	272 (125 to 516)	40.1 (19.9 to 77)	-6.14% (-6.4 to -5.88)
Uganda	2708 (1171 to 4850)	3090 (1671 to 5525)	14.12% (-44.72 to 200.44)	401.6 (173.6 to 719.2)	188.3 (101.8 to 336.7)	-2.9% (-3.54 to -2.25)

Location	DALYs in 1990 (95% UI)	DALYs in 2019 (95% UI)	Relative change (%, 95 CI)	Age-standardized DALYs rate in 1990 (95% UI)	Age-standardized DALYs rate in 2019 (95% UI)	EAPC (%, 95 CI)
Ukraine	280 (154 to 470)	170 (81 to 312)	-39.41% (-61.27 to -13.96)	14.2 (7.8 to 23.9)	11.8 (5.6 to 21.7)	-0.15% (-0.39 to 0.08)
United Republic of Tanzania	5185 (2907 to 8377)	3843 (2027 to 6469)	-25.87% (-65.43 to 60.35)	504.8 (283 to 815.6)	166.9 (88 to 280.9)	-3.09% (-3.45 to -2.72)
Uzbekistan	480 (300 to 717)	384 (224 to 590)	-19.92% (-53.17 to 32.49)	51 (31.8 to 76.2)	24.9 (14.5 to 38.2)	-2.02% (-2.76 to -1.28)
Vanuatu	9 (4 to 17)	10 (4 to 20)	10.96% (-64.83 to 166.04)	154 (72.2 to 280.6)	81.3 (28.4 to 154.4)	-2.85% (-3.22 to -2.49)
Venezuela (Bolivarian Republic of)	1261 (829 to 1800)	614 (341 to 1041)	-51.33% (-75.64 to -10.45)	152.9 (100.6 to 218.3)	56.3 (31.3 to 95.5)	-3.2% (-4.33 to -2.06)
Viet Nam	1255 (639 to 2302)	302 (124 to 615)	-75.97% (-91.02 to -48.43)	39.9 (20.3 to 73.1)	6.9 (2.8 to 14.2)	-5.69% (-6.23 to -5.15)
Yemen	4311 (2012 to 7711)	4584 (2122 to 8025)	6.33% (-57.48 to 139.48)	936.4 (437 to 1674.9)	361.8 (167.4 to 633.4)	-4.03% (-4.29 to -3.77)
Zambia	1525 (862 to 2405)	822 (416 to 1391)	-46.11% (-74.65 to 5.34)	506.4 (286.3 to 798.5)	105 (53.2 to 177.8)	-6.03% (-6.73 to -5.33)
Zimbabwe	1331 (782 to 2129)	2736 (1440 to 4686)	105.6% (0.33 to 307.85)	339.7 (199.6 to 543.6)	435.7 (229.3 to 746.2)	4.63% (2.93 to 6.35)

CI – confidence interval, DALYs – disability-adjusted life years (per 100,000 population), EAPC – estimated annual percentage change, MSMI – maternal sepsis and other maternal infections, UI – uncertain interval.

Table S29. DALYs of MSMI in 131 low- and middle-income countries and territories with EAPC in age group of 30-34 years from 1990 and 2019.

Location	DALYs in 1990 (95% UI)	DALYs in 2019 (95% UI)	Relative change (%, 95 CI)	Age-standardized DALYs rate in 1990 (95% UI)	Age-standardized DALYs rate in 2019 (95% UI)	EAPC (%, 95 CI)
Afghanistan	1674 (880 to 2917)	2756 (1333 to 4756)	64.61% (-23.27 to 241.83)	770.6 (405.2 to 1342.8)	267.1 (129.2 to 461)	-4.14% (-5.28 to -2.98)
Albania	13 (7 to 21)	3 (1 to 6)	-73.01% (-88.52 to -47.27)	9.2 (5.2 to 15)	3.8 (1.7 to 7)	-1.85% (-2.61 to -1.07)
Algeria	3458 (1956 to 5699)	486 (265 to 837)	-85.93% (-93.23 to -70.44)	448.4 (253.7 to 739.1)	26 (14.2 to 44.7)	-9.56% (-9.99 to -9.12)
American Samoa	1 (1 to 2)	0 (0 to 1)	-69.08% (-87.17 to -24.1)	66 (34.1 to 117.7)	22.4 (11.8 to 38.1)	-4.19% (-4.41 to -3.97)
Angola	1535 (734 to 2685)	1367 (685 to 2441)	-10.9% (-61.04 to 107.55)	478.5 (228.7 to 837.2)	136.9 (68.5 to 244.4)	-4.14% (-4.5 to -3.78)
Armenia	22 (13 to 36)	6 (3 to 11)	-71.9% (-86.56 to -43.09)	13.9 (8 to 22.4)	4.5 (2.1 to 7.8)	-4.03% (-4.81 to -3.24)
Azerbaijan	92 (54 to 144)	14 (6 to 27)	-84.32% (-93.03 to -69.35)	29 (16.9 to 45.6)	2.9 (1.2 to 5.5)	-9.17% (-9.72 to -8.61)
Bangladesh	3545 (1956 to 5747)	271 (137 to 475)	-92.35% (-96.61 to -83.35)	106.9 (59 to 173.3)	4.1 (2.1 to 7.2)	-10.78% (-12.02 to -9.53)

Location	DALYs in 1990 (95% UI)	DALYs in 2019 (95% UI)	Relative change (%, 95 CI)	Age-standardized DALYs rate in 1990 (95% UI)	Age-standardized DALYs rate in 2019 (95% UI)	EAPC (%, 95 CI)
Belarus	38 (21 to 61)	31 (12 to 64)	-17.93% (-57.82 to 39.18)	8.6 (4.8 to 13.8)	8.1 (3.2 to 16.6)	1.44% (0.67 to 2.21)
Belize	3 (2 to 5)	7 (4 to 11)	123.01% (12.33 to 337.85)	56 (34 to 84.7)	44.7 (26.1 to 69.4)	-0.98% (-1.59 to -0.36)
Benin	1524 (849 to 2446)	1321 (675 to 2380)	-13.3% (-57.12 to 77.78)	955.4 (532.6 to 1533.5)	326.9 (167 to 588.8)	-3.13% (-3.42 to -2.84)
Bhutan	24 (11 to 43)	3 (1 to 5)	-88.56% (-94.82 to -73.73)	130.1 (61.5 to 234)	8.5 (4.3 to 14.4)	-9.38% (-9.68 to -9.07)
Bolivia (Plurinational State of)	1481 (844 to 2430)	498 (222 to 881)	-66.38% (-86.17 to -28.65)	696.5 (396.9 to 1142.7)	111.6 (49.8 to 197.5)	-6.49% (-6.83 to -6.14)
Bosnia and Herzegovina	34 (19 to 54)	3 (1 to 6)	-92.02% (-97.79 to -79.3)	18.2 (10.2 to 29.3)	2.5 (0.6 to 5.9)	-8.91% (-10.82 to -6.97)
Botswana	128 (60 to 233)	47 (22 to 86)	-63.08% (-84.83 to -11.79)	290.2 (136.7 to 528.5)	42.7 (19.7 to 78.3)	-6.67% (-7.58 to -5.75)
Brazil	5122 (3303 to 7419)	2049 (1346 to 2981)	-59.98% (-77.82 to -26.47)	90.1 (58.1 to 130.5)	22.9 (15.1 to 33.4)	-3.62% (-4.26 to -2.98)
Bulgaria	18 (10 to 28)	7 (3 to 14)	-59.97% (-83.69 to -10.38)	6.1 (3.5 to 9.4)	3.1 (1.3 to 5.8)	-4.09% (-4.76 to -3.41)

Location	DALYs in 1990 (95% UI)	DALYs in 2019 (95% UI)	Relative change (%, 95 CI)	Age-standardized DALYs rate in 1990 (95% UI)	Age-standardized DALYs rate in 2019 (95% UI)	EAPC (%, 95 CI)
Burkina Faso	2124 (1164 to 3583)	2010 (1030 to 3475)	-5.38% (-56.3 to 97.27)	732.6 (401.5 to 1235.6)	262.7 (134.7 to 454.2)	-4.21% (-4.62 to -3.8)
Burundi	2971 (1561 to 5041)	3697 (2083 to 5721)	24.42% (-37.31 to 158.17)	1571.6 (825.8 to 2666.1)	930.4 (524.2 to 1439.7)	-2.18% (-2.69 to -1.66)
Cabo Verde	19 (10 to 32)	2 (1 to 4)	-88.12% (-94.31 to -76.02)	167.6 (91.6 to 282.1)	9.1 (5 to 15.1)	-10.38% (-10.72 to -10.04)
Cambodia	2682 (1297 to 4754)	506 (254 to 926)	-81.12% (-91.7 to -58.49)	734.7 (355.1 to 1302.2)	71.7 (36 to 131.1)	-8.99% (-9.97 to -8)
Cameroon	2043 (1163 to 3352)	2513 (1184 to 4446)	22.97% (-42.29 to 151.01)	628 (357.4 to 1030.2)	233.8 (110.2 to 413.8)	-3.23% (-3.74 to -2.72)
Central African Republic	1136 (604 to 1854)	2238 (1117 to 3851)	97.04% (-2.48 to 304.71)	1261.2 (670.5 to 2058.9)	1248.1 (622.8 to 2147.9)	0.1% (-0.11 to 0.31)
Chad	2575 (1476 to 4193)	3730 (1984 to 6195)	44.85% (-25.97 to 182.29)	1433.6 (821.7 to 2334.7)	796.7 (423.8 to 1323.1)	-1.98% (-2.4 to -1.56)
China	19451 (14994 to 24829)	1589 (771 to 3015)	-91.83% (-95.88 to -84.95)	45.9 (35.4 to 58.6)	2.5 (1.2 to 4.7)	-9.82% (-10.57 to -9.06)
Colombia	866 (518 to 1365)	335 (194 to 542)	-61.34% (-81.3 to -20.47)	67.5 (40.4 to 106.5)	18.5 (10.7 to 30)	-4.14% (-4.67 to -3.61)

Location	DALYs in 1990 (95% UI)	DALYs in 2019 (95% UI)	Relative change (%, 95 CI)	Age-standardized DALYs rate in 1990 (95% UI)	Age-standardized DALYs rate in 2019 (95% UI)	EAPC (%, 95 CI)
Comoros	75 (15 to 141)	58 (25 to 111)	-22.43% (-69.02 to 278.76)	549 (107.9 to 1034.5)	223.5 (96.2 to 426.7)	-3.63% (-4.22 to -3.04)
Congo	341 (154 to 619)	296 (145 to 544)	-13.19% (-63.73 to 112)	460 (207 to 834.5)	146.1 (71.5 to 268.4)	-3.64% (-4.5 to -2.77)
Costa Rica	72 (45 to 106)	17 (9 to 26)	-77.09% (-87.9 to -58.91)	59.8 (37.1 to 87.7)	8 (4.5 to 12.6)	-6.92% (-8.19 to -5.63)
Côte d'Ivoire	1533 (836 to 2668)	1859 (909 to 3269)	21.26% (-45.76 to 165.33)	398.9 (217.5 to 694.2)	187.9 (91.9 to 330.4)	-2.21% (-2.7 to -1.72)
Cuba	183 (114 to 275)	50 (29 to 78)	-72.62% (-85.61 to -48.57)	47.4 (29.4 to 70.9)	12.9 (7.6 to 20)	-3.68% (-4.12 to -3.23)
Democratic People's Republic of Korea	562 (277 to 1012)	454 (189 to 853)	-19.34% (-67.28 to 100.87)	76 (37.4 to 136.6)	48.3 (20.1 to 90.8)	-1.32% (-2.29 to -0.35)
Democratic Republic of the Congo	9643 (5017 to 16065)	23973 (13240 to 38039)	148.59% (22.4 to 417.44)	820.1 (426.6 to 1366.2)	839.8 (463.8 to 1332.7)	1.61% (0.39 to 2.85)
Djibouti	115 (60 to 196)	221 (92 to 413)	91.08% (-19.02 to 327.02)	845.2 (442.1 to 1435.1)	409.3 (170.6 to 766)	-2.29% (-2.78 to -1.81)

Location	DALYs in 1990 (95% UI)	DALYs in 2019 (95% UI)	Relative change (%, 95 CI)	Age-standardized DALYs rate in 1990 (95% UI)	Age-standardized DALYs rate in 2019 (95% UI)	EAPC (%, 95 CI)
Dominica	3<br>(2 to 5)	2<br>(1 to 4)	-30.56%<br>(-69.63 to 51.86)	133.8<br>(74 to 213.6)	81.2<br>(42.5 to 140.1)	-1.8%<br>(-2.16 to -1.44)
Dominican Republic	166<br>(98 to 284)	132<br>(66 to 251)	-20.52%<br>(-65.44 to 76.6)	64.2<br>(37.8 to 109.4)	31.8<br>(15.9 to 60.2)	-1.71%<br>(-2.35 to -1.07)
Ecuador	508<br>(314 to 762)	203<br>(108 to 347)	-60.08%<br>(-81.33 to -21.07)	144.2<br>(89.1 to 216.3)	30.8<br>(16.3 to 52.7)	-4.3%<br>(-5.32 to -3.28)
Egypt	3235<br>(1877 to 5315)	414<br>(228 to 700)	-87.2%<br>(-93.41 to -74.83)	179.4<br>(104.1 to 294.8)	10.9<br>(6 to 18.5)	-9.85%<br>(-10.4 to -9.29)
El Salvador	643<br>(375 to 1024)	41<br>(20 to 76)	-93.68%<br>(-97.17 to -86.5)	374.9<br>(218.6 to 597.1)	17.2<br>(8.6 to 31.9)	-9.99%<br>(-11.43 to -8.52)
Equatorial Guinea	202<br>(104 to 331)	21<br>(9 to 41)	-89.56%<br>(-95.8 to -75.93)	1540<br>(794.2 to 2522.8)	43.6<br>(18.9 to 85.5)	-12.94%<br>(-13.65 to -12.23)
Eritrea	2233<br>(1200 to 3757)	1760<br>(929 to 3016)	-21.22%<br>(-61.47 to 52.12)	2354.3<br>(1265 to 3959.9)	752.8<br>(397.3 to 1290.3)	-2.95%<br>(-3.55 to -2.34)
Eswatini	49<br>(24 to 87)	26<br>(11 to 52)	-46.74%<br>(-79.06 to 35.98)	190.6<br>(93.3 to 340.6)	53.3<br>(23.2 to 105.9)	-3.24%<br>(-4.39 to -2.07)
Ethiopia	38256<br>(21734 to 62239)	12044<br>(6634 to 18904)	-68.52%<br>(-84.17 to -39.72)	2454.3<br>(1394.4 to 3992.9)	337.9<br>(186.1 to 530.4)	-7.1%<br>(-7.92 to -6.28)
Fiji	13<br>(6 to 23)	7<br>(4 to 12)	-46.2%<br>(-77.78 to 27.09)	44.7<br>(22.3 to 79.7)	20.7<br>(10.5 to 36.7)	-2.78%<br>(-2.91 to -2.65)

Location	DALYs in 1990 (95% UI)	DALYs in 2019 (95% UI)	Relative change (%, 95 CI)	Age-standardized DALYs rate in 1990 (95% UI)	Age-standardized DALYs rate in 2019 (95% UI)	EAPC (%, 95 CI)
Gabon	84 (40 to 151)	30 (16 to 56)	-63.83% (-83.83 to -15.65)	287.9 (137.8 to 516.4)	43.7 (22.8 to 80.1)	-6.72% (-7.38 to -6.05)
Gambia	218 (115 to 355)	292 (151 to 514)	34.17% (-36.87 to 191.08)	661.5 (347.8 to 1077.9)	361.4 (186.8 to 635.4)	-2.27% (-2.65 to -1.89)
Georgia	27 (16 to 43)	20 (11 to 33)	-27% (-64.56 to 51.27)	12.1 (7.1 to 18.9)	15 (8.4 to 24.6)	3.21% (1.52 to 4.93)
Ghana	1925 (996 to 3258)	931 (463 to 1658)	-51.64% (-76.25 to 3.32)	378.9 (196 to 641.4)	72.1 (35.9 to 128.5)	-5.04% (-5.31 to -4.78)
Global	372251 (307466 to 459303)	179234 (147168 to 215704)	-51.85% (-62.42 to -39.44)	195.8 (161.7 to 241.5)	60 (49.3 to 72.2)	-3.81% (-4.11 to -3.5)
Grenada	1 (0 to 1)	0 (0 to 0)	-65.28% (-81.28 to -37.85)	25.8 (16 to 39.5)	7.2 (3.9 to 12)	-3.95% (-4.55 to -3.35)
Guatemala	743 (434 to 1197)	280 (158 to 459)	-62.37% (-80.11 to -28.87)	302.6 (176.5 to 487.5)	39.7 (22.4 to 65)	-8.33% (-9.22 to -7.44)
Guinea	2279 (1330 to 3628)	2529 (1376 to 4307)	10.95% (-41.29 to 111.95)	1087.5 (634.5 to 1730.9)	558.8 (304.1 to 951.5)	-1.76% (-1.93 to -1.59)
Guinea-Bissau	212 (112 to 353)	136 (69 to 241)	-35.86% (-71.32 to 30.91)	634.1 (336.2 to 1054)	179.8 (91.8 to 318.4)	-3.88% (-4.24 to -3.53)

Location	DALYs in 1990 (95% UI)	DALYs in 2019 (95% UI)	Relative change (%, 95 CI)	Age-standardized DALYs rate in 1990 (95% UI)	Age-standardized DALYs rate in 2019 (95% UI)	EAPC (%, 95 CI)
Guyana	36 (20 to 60)	9 (5 to 17)	-73.34% (-87.48 to -38.58)	123.4 (67.9 to 210)	35.2 (19.5 to 63.5)	-3.78% (-4.27 to -3.3)
Haiti	2684 (1315 to 4749)	4369 (2224 to 7413)	62.8% (-21.83 to 242.45)	1208.9 (592.2 to 2139.1)	801.1 (407.9 to 1359.3)	-0.73% (-1.07 to -0.38)
Honduras	1149 (685 to 1795)	192 (83 to 363)	-83.32% (-93 to -62.98)	806.7 (480.5 to 1259.5)	50.5 (21.7 to 95.7)	-9.63% (-10.19 to -9.08)
India	118608 (70882 to 187532)	19929 (11120 to 32158)	-83.2% (-91.61 to -67.76)	417 (249.2 to 659.3)	36.7 (20.5 to 59.3)	-8.56% (-9.26 to -7.87)
Indonesia	14894 (8249 to 24491)	2457 (1514 to 3825)	-83.5% (-91.45 to -67.74)	215.3 (119.2 to 354)	24.5 (15.1 to 38.2)	-6.96% (-7.32 to -6.59)
Iran (Islamic Republic of)	1743 (1152 to 2457)	318 (196 to 497)	-81.76% (-89.44 to -67.75)	96.7 (63.9 to 136.4)	7.3 (4.5 to 11.4)	-8.75% (-9.31 to -8.18)
Iraq	125 (66 to 216)	117 (56 to 215)	-6.56% (-51.65 to 68.39)	24.4 (13 to 42.2)	7.2 (3.5 to 13.3)	-4.3% (-4.79 to -3.8)
Jamaica	35 (21 to 54)	11 (6 to 18)	-68.88% (-85.58 to -37.85)	41.7 (25 to 63.8)	9.5 (5.1 to 15.8)	-6.5% (-7.73 to -5.25)
Jordan	137 (74 to 238)	55 (29 to 95)	-59.53% (-80.54 to -12.78)	140.6 (75.7 to 244.2)	14.4 (7.7 to 24.6)	-9.02% (-9.59 to -8.45)

Location	DALYs in 1990 (95% UI)	DALYs in 2019 (95% UI)	Relative change (%, 95 CI)	Age-standardized DALYs rate in 1990 (95% UI)	Age-standardized DALYs rate in 2019 (95% UI)	EAPC (%, 95 CI)
Kazakhstan	46 (24 to 80)	77 (44 to 124)	65.08% (-3.37 to 189.44)	6.7 (3.4 to 11.6)	9.4 (5.5 to 15.3)	2.54% (1.94 to 3.14)
Kenya	2613 (1525 to 4246)	2874 (1646 to 4742)	9.98% (-39.71 to 97.98)	395.2 (230.6 to 642.1)	153.7 (88 to 253.7)	-3% (-3.62 to -2.36)
Kiribati	5 (2 to 10)	5 (2 to 9)	1.34% (-62.63 to 158.66)	185.7 (88.7 to 354.8)	112.2 (50.2 to 203.2)	-1.8% (-2.02 to -1.58)
Kyrgyzstan	55 (33 to 86)	68 (42 to 104)	23.8% (-30.05 to 121.74)	32.3 (19.6 to 50.4)	24.9 (15.4 to 38.2)	1.07% (0.14 to 2)
Lao People's Democratic Republic	1210 (715 to 1943)	125 (62 to 220)	-89.7% (-95.47 to -79.1)	889.3 (525.5 to 1428.2)	42.3 (21.2 to 74.8)	-10.35% (-10.96 to -9.73)
Lebanon	43 (23 to 74)	15 (7 to 26)	-65.71% (-84.58 to -31.38)	42 (22.4 to 72.4)	5.9 (2.8 to 10.5)	-6.65% (-7.17 to -6.12)
Lesotho	314 (157 to 527)	214 (91 to 414)	-31.8% (-72.46 to 54.28)	555.3 (276.7 to 932)	255.1 (108.9 to 492.4)	-0.57% (-1.5 to 0.37)
Liberia	811 (476 to 1296)	1336 (750 to 2157)	64.64% (-17.11 to 201.71)	1279.3 (751.1 to 2042.7)	783.3 (439.6 to 1264.8)	-2.92% (-3.93 to -1.9)

Location	DALYs in 1990 (95% UI)	DALYs in 2019 (95% UI)	Relative change (%, 95 CI)	Age-standardized DALYs rate in 1990 (95% UI)	Age-standardized DALYs rate in 2019 (95% UI)	EAPC (%, 95 CI)
Libya	44<br>(23 to 76)	22<br>(11 to 40)	-50.27%<br>(-76.71 to 8.08)	40<br>(20.6 to 69.1)	7.3<br>(3.7 to 13.4)	-5.56%<br>(-6.27 to -4.85)
Madagascar	2844<br>(1615 to 4594)	3494<br>(1799 to 5844)	22.86%<br>(-40.58 to 137.27)	754.3<br>(428.4 to 1218.6)	385.1<br>(198.2 to 644.1)	-2.26%<br>(-2.66 to -1.85)
Malawi	1387<br>(754 to 2313)	1180<br>(605 to 2108)	-14.92%<br>(-61.98 to 90.12)	483.2<br>(262.7 to 805.7)	184.2<br>(94.4 to 329)	-3.29%<br>(-3.75 to -2.83)
Malaysia	690<br>(386 to 1172)	196<br>(109 to 331)	-71.61%<br>(-87.37 to -40.99)	101.4<br>(56.7 to 172.3)	14.8<br>(8.2 to 25.1)	-6.94%<br>(-7.42 to -6.45)
Maldives	8<br>(4 to 13)	2<br>(1 to 3)	-79.03%<br>(-90.47 to -56.25)	123.6<br>(68.1 to 210.4)	7.5<br>(3.8 to 13.1)	-9.32%<br>(-10.29 to -8.35)
Mali	5905<br>(3693 to 8656)	3894<br>(2088 to 6464)	-34.05%<br>(-66.42 to 16.82)	2175.3<br>(1360.3 to 3188.7)	587.7<br>(315.1 to 975.5)	-4.74%<br>(-5.04 to -4.44)
Marshall Islands	2<br>(1 to 4)	2<br>(1 to 4)	-21.01%<br>(-74.79 to 90.04)	175.6<br>(87.6 to 309.8)	88.5<br>(36.3 to 172.5)	-2.63%<br>(-3.29 to -1.96)
Mauritania	1337<br>(832 to 1989)	351<br>(179 to 614)	-73.77%<br>(-86.93 to -50.43)	2055.2<br>(1279.5 to 3057.5)	256.8<br>(130.8 to 449.5)	-7.56%<br>(-7.81 to -7.3)
Mexico	1208<br>(770 to 1789)	605<br>(365 to 957)	-49.89%<br>(-71.99 to -14.07)	40.6<br>(25.9 to 60.2)	12.4<br>(7.5 to 19.5)	-3.96%<br>(-4.35 to -3.57)

Location	DALYs in 1990 (95% UI)	DALYs in 2019 (95% UI)	Relative change (%, 95 CI)	Age-standardized DALYs rate in 1990 (95% UI)	Age-standardized DALYs rate in 2019 (95% UI)	EAPC (%, 95 CI)
Micronesia (Federated States of)	11<br>(5 to 21)	3<br>(1 to 6)	-73.41%<br>(-94.81 to -31.89)	316.7<br>(143.1 to 614.8)	82.7<br>(15.7 to 170.6)	-5.19%<br>(-5.65 to -4.73)
Mongolia	251<br>(135 to 419)	76<br>(38 to 139)	-69.77%<br>(-86.47 to -27.44)	333.4<br>(178.8 to 556.4)	46.7<br>(23.2 to 85.6)	-7.68%<br>(-8.13 to -7.23)
Montenegro	1<br>(0 to 1)	1<br>(0 to 1)	0.02%<br>(-63.36 to 167.47)	2.4<br>(0.7 to 5)	2.7<br>(0.7 to 5.9)	0.41%<br>(0.33 to 0.49)
Morocco	3243<br>(1764 to 5455)	279<br>(141 to 522)	-91.4%<br>(-95.85 to -81.72)	369.7<br>(201.1 to 621.9)	19.5<br>(9.9 to 36.4)	-9.83%<br>(-10.13 to -9.53)
Mozambique	3061<br>(1701 to 5066)	1265<br>(616 to 2273)	-58.68%<br>(-81.58 to -14.87)	724.8<br>(402.9 to 1199.7)	141.5<br>(69 to 254.3)	-5.15%<br>(-5.6 to -4.71)
Myanmar	4946<br>(2342 to 9104)	1412<br>(704 to 2486)	-71.45%<br>(-87.55 to -34.31)	334.3<br>(158.2 to 615.2)	64.7<br>(32.2 to 113.8)	-6.04%<br>(-6.58 to -5.5)
Namibia	116<br>(51 to 196)	48<br>(21 to 100)	-58.19%<br>(-83.98 to 15.21)	257.1<br>(112.8 to 435.7)	50.7<br>(21.8 to 105.2)	-5.28%<br>(-5.81 to -4.76)
Nepal	4752<br>(2547 to 7918)	420<br>(198 to 752)	-91.17%<br>(-96.21 to -81.16)	754.6<br>(404.5 to 1257.3)	33.3<br>(15.7 to 59.6)	-10.58%<br>(-10.78 to -10.38)
Nicaragua	176<br>(100 to 285)	29<br>(16 to 48)	-83.27%<br>(-91.95 to -65.24)	144.9<br>(82.6 to 235)	11.1<br>(5.9 to 18.3)	-8.99%<br>(-9.56 to -8.43)

Location	DALYs in 1990 (95% UI)	DALYs in 2019 (95% UI)	Relative change (%, 95 CI)	Age-standardized DALYs rate in 1990 (95% UI)	Age-standardized DALYs rate in 2019 (95% UI)	EAPC (%, 95 CI)
Niger	4015 (2198 to 6210)	5453 (2827 to 9069)	35.82% (-32.54 to 154.82)	1641.8 (898.7 to 2539.3)	942.8 (488.8 to 1568.1)	-2.2% (-2.46 to -1.94)
Nigeria	9106 (4862 to 16605)	10007 (5306 to 17762)	9.9% (-47.62 to 135.61)	339 (181 to 618.3)	142 (75.3 to 252.1)	-3.76% (-4.51 to -3.01)
North Macedonia	6 (4 to 11)	2 (1 to 4)	-69.84% (-90.53 to -30.98)	8.3 (4.6 to 13.8)	2.4 (0.8 to 5.2)	-5.53% (-6.33 to -4.72)
Pakistan	14875 (8486 to 22764)	8542 (4677 to 14379)	-42.57% (-71.53 to 16.67)	501.5 (286.1 to 767.6)	114.3 (62.6 to 192.4)	-5.42% (-5.86 to -4.98)
Papua New Guinea	775 (394 to 1297)	879 (434 to 1585)	13.39% (-50.37 to 136.81)	576 (293.1 to 964.3)	236.6 (116.8 to 426.9)	-2.36% (-2.82 to -1.91)
Paraguay	334 (196 to 518)	74 (40 to 125)	-77.94% (-89.07 to -52.68)	250 (146.9 to 388)	26.4 (14.3 to 44.7)	-7.81% (-8.39 to -7.22)
Peru	2190 (1312 to 3521)	276 (141 to 499)	-87.38% (-94.15 to -74.85)	292.9 (175.4 to 470.8)	20.8 (10.7 to 37.6)	-9.83% (-10.36 to -9.3)
Philippines	1947 (1230 to 2937)	916 (551 to 1433)	-52.96% (-75.58 to -15.92)	89.6 (56.6 to 135.1)	22.9 (13.8 to 35.8)	-4.48% (-4.63 to -4.34)
Republic of Moldova	38 (23 to 57)	9 (3 to 18)	-77.4% (-90.79 to -58)	19.5 (11.8 to 29.5)	5.3 (2 to 11.2)	-4.12% (-4.82 to -3.41)

Location	DALYs in 1990 (95% UI)	DALYs in 2019 (95% UI)	Relative change (%, 95 CI)	Age-standardized DALYs rate in 1990 (95% UI)	Age-standardized DALYs rate in 2019 (95% UI)	EAPC (%, 95 CI)
Russian Federation	845 (543 to 1240)	615 (317 to 1129)	-27.21% (-59.61 to 11.58)	12.8 (8.2 to 18.8)	9.9 (5.1 to 18.1)	-0.36% (-0.66 to -0.06)
Rwanda	5129 (3093 to 7636)	1686 (841 to 2941)	-67.13% (-84.94 to -34.76)	2157 (1300.9 to 3211.7)	338.7 (169 to 590.9)	-6.81% (-7.66 to -5.94)
Saint Lucia	1 (1 to 2)	1 (0 to 1)	-60.35% (-80.13 to -22.62)	30.8 (18.9 to 48.3)	9 (5.2 to 14.9)	-2.58% (-3.56 to -1.59)
Saint Vincent and the Grenadines	0 (0 to 1)	0 (0 to 0)	-33.09% (-62.47 to 15.91)	9.8 (5.7 to 15.9)	6.3 (3.3 to 10.8)	-1.59% (-1.97 to -1.21)
Samoa	4 (2 to 8)	1 (0 to 2)	-73.25% (-91.27 to -27.97)	87.5 (41.1 to 166.7)	16.2 (5.6 to 31.3)	-6.7% (-7.23 to -6.17)
Senegal	1795 (1034 to 2909)	1505 (781 to 2608)	-16.16% (-56.7 to 66.52)	774.6 (446.1 to 1255.4)	286.1 (148.5 to 495.8)	-3.17% (-3.47 to -2.88)
Serbia	19 (10 to 32)	6 (2 to 13)	-69.34% (-89.89 to -25.56)	5.4 (2.7 to 9)	2.1 (0.6 to 4.4)	-3.11% (-3.57 to -2.65)
Sierra Leone	836 (451 to 1417)	1593 (862 to 2711)	90.65% (-5.49 to 286.19)	655.4 (354 to 1111)	523 (282.9 to 890)	-0.56% (-1.37 to 0.26)
Solomon Islands	67 (31 to 124)	100 (43 to 188)	48.14% (-42.32 to 257.85)	717.8 (327.7 to 1325.3)	430.4 (186.2 to 813.3)	-1.23% (-1.9 to -0.56)

Location	DALYs in 1990 (95% UI)	DALYs in 2019 (95% UI)	Relative change (%, 95 CI)	Age-standardized DALYs rate in 1990 (95% UI)	Age-standardized DALYs rate in 2019 (95% UI)	EAPC (%, 95 CI)
Somalia	4124 (2004 to 6998)	9169 (5013 to 14908)	122.35% (15.61 to 342.86)	1886.5 (917 to 3201.4)	1680.3 (918.7 to 2732)	-0.27% (-0.48 to -0.06)
South Africa	3476 (2149 to 5330)	1156 (573 to 2113)	-66.76% (-85.36 to -29.18)	247.1 (152.8 to 378.8)	45.2 (22.4 to 82.6)	-5.52% (-7.88 to -3.1)
South Sudan	711 (332 to 1372)	769 (350 to 1490)	8.15% (-52.45 to 160.4)	415 (193.6 to 800.1)	267.3 (121.6 to 517.6)	-1.7% (-1.91 to -1.48)
Sri Lanka	169 (95 to 269)	53 (27 to 95)	-68.54% (-83.95 to -37.88)	24.8 (14 to 39.4)	6.7 (3.3 to 12)	-4.19% (-4.38 to -3.99)
Sudan	8010 (4470 to 12712)	2147 (904 to 3915)	-73.2% (-89.69 to -43.88)	1248.5 (696.7 to 1981.3)	137.9 (58.1 to 251.6)	-7.52% (-7.97 to -7.06)
Suriname	5 (3 to 9)	6 (3 to 11)	14.43% (-50.03 to 174.71)	39.9 (20.7 to 71.2)	27.8 (14.8 to 50.4)	-1.34% (-2.09 to -0.59)
Syrian Arab Republic	344 (182 to 591)	35 (18 to 62)	-89.75% (-95.48 to -76.51)	97.2 (51.3 to 167)	8.6 (4.3 to 15.2)	-9.39% (-10.17 to -8.59)
Tajikistan	79 (47 to 123)	47 (24 to 79)	-40.53% (-70.05 to 9.23)	43.9 (26 to 68.7)	11.6 (5.9 to 19.6)	-5.71% (-6.18 to -5.24)
Thailand	304 (149 to 522)	155 (73 to 275)	-49.13% (-77.73 to 10.82)	12.4 (6.1 to 21.3)	6.3 (3 to 11.2)	-2.02% (-2.88 to -1.16)

Location	DALYs in 1990 (95% UI)	DALYs in 2019 (95% UI)	Relative change (%, 95 CI)	Age-standardized DALYs rate in 1990 (95% UI)	Age-standardized DALYs rate in 2019 (95% UI)	EAPC (%, 95 CI)
Timor-Leste	238 (113 to 416)	65 (10 to 121)	-72.55% (-95.68 to -31.56)	853.6 (404.5 to 1488.4)	160.5 (23.8 to 296.8)	-7.02% (-7.73 to -6.31)
Togo	725 (396 to 1186)	742 (367 to 1299)	2.4% (-53.82 to 119.99)	612.6 (334.9 to 1002.8)	234.8 (116.2 to 411.1)	-2.93% (-3.32 to -2.53)
Tonga	3 (2 to 6)	1 (1 to 3)	-58.07% (-81.39 to -3.78)	122.8 (63.3 to 211)	42.3 (20.6 to 75.1)	-4.02% (-4.18 to -3.86)
Tunisia	265 (142 to 442)	44 (23 to 77)	-83.29% (-92.51 to -65.08)	90 (48.5 to 150.5)	9.1 (4.7 to 15.8)	-6.82% (-7.35 to -6.3)
Turkey	2741 (1491 to 4634)	299 (158 to 515)	-89.08% (-95.35 to -76.27)	131.3 (71.4 to 221.9)	8.9 (4.7 to 15.3)	-10.4% (-11.37 to -9.43)
Turkmenistan	29 (17 to 46)	21 (11 to 37)	-27.01% (-56.86 to 16.85)	21 (11.9 to 33.3)	11 (5.9 to 19.2)	-2.1% (-2.29 to -1.9)
Tuvalu	1 (1 to 2)	0 (0 to 0)	-83.81% (-93.6 to -56.61)	297.5 (139 to 542.9)	46.3 (20.9 to 91.3)	-5.95% (-6.19 to -5.72)
Uganda	1663 (669 to 3082)	2115 (1083 to 3782)	27.16% (-47.11 to 225.29)	342.1 (137.6 to 634.1)	162.7 (83.3 to 291)	-2.87% (-3.48 to -2.26)
Ukraine	146 (82 to 248)	133 (65 to 253)	-8.95% (-46.07 to 46.28)	7.1 (4 to 12)	7.3 (3.5 to 13.8)	0.82% (0.43 to 1.2)

Location	DALYs in 1990 (95% UI)	DALYs in 2019 (95% UI)	Relative change (%, 95 CI)	Age-standardized DALYs rate in 1990 (95% UI)	Age-standardized DALYs rate in 2019 (95% UI)	EAPC (%, 95 CI)
United Republic of Tanzania	3703 (2048 to 6139)	3098 (1661 to 5328)	-16.36% (-62.25 to 81.87)	472.4 (261.3 to 783)	163.9 (87.9 to 281.8)	-2.97% (-3.29 to -2.64)
Uzbekistan	361 (223 to 522)	232 (131 to 358)	-35.7% (-67.32 to 16.62)	47.8 (29.5 to 69.2)	15.9 (9 to 24.5)	-2.59% (-3.41 to -1.77)
Vanuatu	8 (3 to 16)	11 (3 to 21)	27.7% (-61.64 to 226.74)	163 (66.7 to 314.3)	97.4 (27.3 to 192.4)	-2.6% (-3 to -2.19)
Venezuela (Bolivarian Republic of)	1050 (672 to 1497)	456 (261 to 729)	-56.56% (-77.29 to -13.45)	145.9 (93.5 to 208.1)	40.8 (23.3 to 65.2)	-4.19% (-5.22 to -3.14)
Viet Nam	879 (416 to 1659)	162 (63 to 337)	-81.54% (-93.86 to -55.47)	33 (15.6 to 62.2)	3.8 (1.5 to 7.9)	-7.29% (-7.91 to -6.67)
Yemen	3580 (1485 to 6518)	4145 (1744 to 7294)	15.78% (-50.98 to 168.13)	869.5 (360.6 to 1583)	345.4 (145.3 to 607.7)	-3.97% (-4.25 to -3.69)
Zambia	1037 (546 to 1679)	642 (319 to 1111)	-38.06% (-71.37 to 31.28)	462 (243.2 to 747.8)	101 (50.1 to 174.6)	-5.9% (-6.65 to -5.15)
Zimbabwe	1084 (632 to 1709)	2373 (1215 to 4236)	118.9% (8.61 to 329.84)	345.3 (201.4 to 544.3)	420 (215 to 749.6)	4.38% (2.72 to 6.06)

CI – confidence interval, DALYs – disability-adjusted life years (per 100,000 population), EAPC – estimated annual percentage change, MSMI – maternal sepsis and other maternal infections, UI – uncertain interval.

Table S30. DALYs of MSMI in 131 low- and middle-income countries and territories with EAPC in age group of 35-39 years from 1990 and 2019.

Location	DALYs in 1990 (95% UI)	DALYs in 2019 (95% UI)	Relative change (%, 95 CI)	Age-standardized DALYs rate in 1990 (95% UI)	Age-standardized DALYs rate in 2019 (95% UI)	EAPC (%, 95 CI)
Afghanistan	2117 (1064 to 3808)	2219 (1087 to 3980)	4.83% (-51.6 to 128.04)	783.9 (394.1 to 1410.3)	280.2 (137.3 to 502.6)	-4.14% (-5.28 to -2.98)
Albania	5 (3 to 8)	2 (1 to 3)	-65.52% (-86.61 to -23.69)	4.9 (2.6 to 8.2)	2.1 (0.9 to 3.9)	-1.47% (-2.44 to -0.48)
Algeria	2408 (1297 to 4186)	361 (187 to 603)	-85.02% (-93.04 to -69.74)	389.1 (209.6 to 676.5)	20.6 (10.7 to 34.5)	-9.89% (-10.29 to -9.5)
American Samoa	1 (1 to 3)	1 (0 to 1)	-64.42% (-84.53 to -16.22)	102 (53.9 to 183.2)	32.2 (16.1 to 56.4)	-4.41% (-4.61 to -4.2)
Angola	1704 (797 to 3086)	1465 (711 to 2686)	-14.05% (-63.34 to 106.56)	623.9 (292 to 1129.9)	184.4 (89.5 to 338.1)	-4.11% (-4.5 to -3.73)
Armenia	6 (4 to 10)	2 (1 to 4)	-64.73% (-85.31 to -24.84)	5.2 (2.9 to 8.4)	1.8 (0.8 to 3.4)	-3.54% (-4.31 to -2.76)
Azerbaijan	55 (31 to 91)	8 (4 to 15)	-85.41% (-93.65 to -68.52)	24.7 (13.8 to 41)	2 (0.9 to 3.6)	-9.9% (-10.63 to -9.16)
Bangladesh	2907 (1427 to 5156)	190 (99 to 313)	-93.47% (-97.06 to -84.19)	112.9 (55.5 to 200.4)	3.3 (1.7 to 5.5)	-11.58% (-12.63 to -10.51)

Location	DALYs in 1990 (95% UI)	DALYs in 2019 (95% UI)	Relative change (%, 95 CI)	Age-standardized DALYs rate in 1990 (95% UI)	Age-standardized DALYs rate in 2019 (95% UI)	EAPC (%, 95 CI)
Belarus	17 (10 to 27)	12 (4 to 25)	-29.28% (-74.57 to 41.42)	4.4 (2.6 to 7)	3.2 (1.1 to 6.7)	0.32% (-0.51 to 1.17)
Belize	2 (1 to 3)	4 (2 to 6)	87.36% (-5.27 to 246.2)	43.7 (27.4 to 65.3)	24.8 (14.3 to 39.5)	-1.66% (-2.1 to -1.22)
Benin	1157 (668 to 1895)	1016 (533 to 1786)	-12.21% (-55.96 to 80.39)	926.3 (534.4 to 1516.2)	301.5 (158.1 to 530)	-3.26% (-3.58 to -2.93)
Bhutan	17 (7 to 30)	2 (1 to 3)	-88.4% (-94.74 to -72.91)	111.8 (49.6 to 197.6)	6.9 (3.6 to 12.3)	-9.58% (-9.83 to -9.32)
Bolivia (Plurinational State of)	1277 (669 to 2178)	470 (216 to 839)	-63.17% (-84.75 to -16.65)	686.2 (359.3 to 1170.7)	118.2 (54.2 to 211)	-6.28% (-6.67 to -5.88)
Bosnia and Herzegovina	20 (11 to 35)	1 (0 to 3)	-95.51% (-99.64 to -85.78)	12.1 (6.3 to 20.6)	0.8 (0.1 to 2.3)	-11.66% (-13.63 to -9.65)
Botswana	80 (38 to 147)	31 (14 to 59)	-61.23% (-84.88 to -4.41)	230.1 (108.8 to 420.2)	31.5 (14.2 to 59.4)	-7.01% (-7.97 to -6.04)
Brazil	3748 (2376 to 5550)	1492 (986 to 2211)	-60.2% (-77.77 to -29.46)	77.3 (49 to 114.4)	16.7 (11 to 24.7)	-4.12% (-4.8 to -3.44)
Bulgaria	39 (22 to 63)	5 (2 to 8)	-88.2% (-94.9 to -74.02)	12.7 (7.2 to 20.3)	2 (0.9 to 3.6)	-6.83% (-7.62 to -6.04)

Location	DALYs in 1990 (95% UI)	DALYs in 2019 (95% UI)	Relative change (%, 95 CI)	Age-standardized DALYs rate in 1990 (95% UI)	Age-standardized DALYs rate in 2019 (95% UI)	EAPC (%, 95 CI)
Burkina Faso	1883<br>(1048 to 3058)	1636<br>(835 to 2730)	-13.13%<br>(-59.93 to 67.16)	798.6<br>(444.6 to 1297.1)	256<br>(130.7 to 427.4)	-4.7%<br>(-5.12 to -4.29)
Burundi	2185<br>(1124 to 3617)	2781<br>(1523 to 4605)	27.27%<br>(-37.84 to 181.48)	1525.1<br>(784.3 to 2524.7)	875<br>(479.2 to 1449)	-2.44%<br>(-2.97 to -1.91)
Cabo Verde	9<br>(5 to 15)	2<br>(1 to 3)	-81.32%<br>(-90.93 to -62.04)	111.9<br>(60.6 to 186.5)	8.4<br>(4.5 to 14.1)	-9.36%<br>(-9.65 to -9.08)
Cambodia	2021<br>(969 to 3742)	462<br>(225 to 817)	-77.13%<br>(-90.73 to -46.04)	692.7<br>(332 to 1282.5)	77.1<br>(37.5 to 136.4)	-8.65%<br>(-9.65 to -7.63)
Cameroon	1482<br>(823 to 2438)	1791<br>(939 to 3245)	20.84%<br>(-43.85 to 154.57)	584.9<br>(324.7 to 962.1)	210.4<br>(110.3 to 381.1)	-3.33%<br>(-3.8 to -2.86)
Central African Republic	1189<br>(646 to 1948)	2540<br>(1315 to 4368)	113.56%<br>(6.11 to 328.62)	1753.5<br>(952.8 to 2872.9)	1690.8<br>(875.8 to 2908.3)	-0.09%<br>(-0.24 to 0.07)
Chad	2232<br>(1268 to 3651)	2931<br>(1669 to 4929)	31.28%<br>(-30.72 to 142.25)	1413.3<br>(802.5 to 2311.3)	771.7<br>(439.5 to 1298)	-2.06%<br>(-2.44 to -1.68)
China	13692<br>(10531 to 17344)	562<br>(332 to 941)	-95.9%<br>(-97.63 to -92.93)	31<br>(23.8 to 39.3)	1.1<br>(0.7 to 1.9)	-10.47%<br>(-11.11 to -9.83)
Colombia	626<br>(375 to 960)	262<br>(143 to 431)	-58.1%<br>(-79.26 to -12.52)	60.5<br>(36.2 to 92.8)	14.8<br>(8.1 to 24.2)	-4.57%<br>(-5.18 to -3.97)

Location	DALYs in 1990 (95% UI)	DALYs in 2019 (95% UI)	Relative change (%, 95 CI)	Age-standardized DALYs rate in 1990 (95% UI)	Age-standardized DALYs rate in 2019 (95% UI)	EAPC (%, 95 CI)
Comoros	40 (8 to 77)	26 (11 to 48)	-33.94% (-76.04 to 269.53)	358.1 (68.2 to 692.6)	115.5 (49.1 to 214)	-4.54% (-5.1 to -3.98)
Congo	353 (160 to 630)	347 (168 to 646)	-1.82% (-56.82 to 141.82)	566.4 (256 to 1009.9)	186.4 (90 to 346.9)	-3.54% (-4.31 to -2.76)
Costa Rica	39 (24 to 58)	11 (6 to 18)	-72.03% (-86.19 to -47.62)	40.9 (25.4 to 61.2)	5.8 (3.3 to 9.6)	-6.79% (-8.04 to -5.53)
Cuba	96 (59 to 144)	29 (17 to 46)	-70.09% (-84.67 to -41.07)	26.9 (16.6 to 40.5)	8.8 (5.2 to 14.1)	-3.2% (-3.76 to -2.64)
Côte d'Ivoire	1114 (575 to 1899)	1441 (721 to 2552)	29.33% (-36.77 to 172.07)	402.2 (207.7 to 685.6)	179.1 (89.6 to 317.1)	-2.4% (-2.84 to -1.96)
Democratic People's Republic of Korea	457 (212 to 900)	415 (177 to 788)	-9.2% (-66.55 to 145.6)	73.5 (34.1 to 144.8)	46.9 (20 to 89.2)	-1.21% (-2.27 to -0.14)
Democratic Republic of the Congo	9480 (4902 to 15972)	23862 (13091 to 37915)	151.72% (27.8 to 408.92)	987 (510.4 to 1662.9)	1031.5 (565.9 to 1639)	1.61% (0.47 to 2.76)
Djibouti	92 (46 to 160)	171 (75 to 330)	84.94% (-20.88 to 321.01)	782.2 (389.5 to 1355.1)	358 (157.2 to 691.7)	-2.46% (-2.96 to -1.95)

Location	DALYs in 1990 (95% UI)	DALYs in 2019 (95% UI)	Relative change (%, 95 CI)	Age-standardized DALYs rate in 1990 (95% UI)	Age-standardized DALYs rate in 2019 (95% UI)	EAPC (%, 95 CI)
Dominica	2 (1 to 3)	1 (1 to 2)	-30.77% (-68.64 to 57.14)	82.1 (45.9 to 135.8)	48.7 (25.7 to 82.7)	-3.11% (-3.85 to -2.37)
Dominican Republic	98 (53 to 161)	77 (38 to 149)	-20.89% (-65.2 to 87.27)	46.4 (25.2 to 76.6)	20.9 (10.3 to 40.1)	-2.15% (-2.83 to -1.46)
Ecuador	402 (242 to 621)	145 (80 to 248)	-63.9% (-83.63 to -27.63)	137.2 (82.5 to 212.2)	23.7 (13 to 40.5)	-5.2% (-6.28 to -4.1)
Egypt	3198 (1833 to 5112)	272 (142 to 472)	-91.49% (-96.06 to -82.26)	200.4 (114.8 to 320.3)	8 (4.2 to 13.9)	-11.48% (-12.03 to -10.92)
El Salvador	486 (274 to 778)	30 (15 to 55)	-93.74% (-97.1 to -86.31)	340 (191.7 to 544.9)	13.8 (6.8 to 24.8)	-10.59% (-12 to -9.15)
Equatorial Guinea	239 (120 to 400)	21 (9 to 41)	-91.35% (-96.67 to -78.12)	1902.2 (951.7 to 3179.1)	53.1 (22.9 to 104.7)	-13.01% (-13.74 to -12.28)
Eritrea	1559 (792 to 2675)	1252 (653 to 2173)	-19.7% (-63.38 to 74.06)	1991.5 (1011.2 to 3417.4)	641.5 (334.6 to 1113.3)	-2.95% (-3.51 to -2.38)
Eswatini	48 (23 to 86)	25 (10 to 51)	-47.65% (-78.91 to 29.54)	239 (116.8 to 429.1)	64.8 (26.7 to 130.8)	-3.41% (-4.6 to -2.22)
Ethiopia	34717 (18430 to 55255)	10665 (6086 to 17363)	-69.28% (-84.44 to -42.07)	2584.1 (1371.8 to 4112.8)	352.6 (201.2 to 574.1)	-7.09% (-7.88 to -6.3)
Fiji	15 (7 to 27)	9 (4 to 16)	-42.42% (-76.89 to 39.52)	62.5 (30.6 to 113.1)	26.2 (12.8 to 47.3)	-3.18% (-3.4 to -2.96)

Location	DALYs in 1990 (95% UI)	DALYs in 2019 (95% UI)	Relative change (%, 95 CI)	Age-standardized DALYs rate in 1990 (95% UI)	Age-standardized DALYs rate in 2019 (95% UI)	EAPC (%, 95 CI)
Gabon	85 (41 to 146)	35 (16 to 64)	-58.81% (-82.77 to -6.34)	353.4 (170.7 to 606.2)	58.9 (26.9 to 108)	-6.36% (-7.02 to -5.71)
Gambia	160 (83 to 271)	257 (135 to 446)	60.64% (-19.98 to 217.75)	682.7 (354.6 to 1155.7)	391.9 (206.4 to 680.5)	-2.15% (-2.46 to -1.84)
Georgia	16 (8 to 27)	13 (7 to 22)	-15.75% (-60.92 to 84.86)	8.1 (4.3 to 13.5)	10.5 (5.7 to 17.6)	3.78% (2.09 to 5.51)
Ghana	1338 (693 to 2230)	641 (353 to 1105)	-52.06% (-77.64 to -2.76)	334.5 (173.3 to 557.6)	59 (32.5 to 101.6)	-5.48% (-5.72 to -5.23)
Global	272825 (228787 to 327491)	146974 (122142 to 177855)	-46.13% (-57.08 to -33.01)	157 (131.7 to 188.5)	54.8 (45.5 to 66.3)	-3.53% (-3.72 to -3.33)
Grenada	0 (0 to 1)	0 (0 to 0)	-60.47% (-79.79 to -29.18)	15.2 (9.3 to 22.8)	4.7 (2.5 to 8.1)	-4.27% (-4.88 to -3.65)
Guatemala	580 (332 to 951)	232 (132 to 394)	-60.05% (-80.21 to -19.81)	274.3 (156.9 to 449.5)	36.6 (20.8 to 62.1)	-8.44% (-9.41 to -7.46)
Guinea	1985 (1125 to 3080)	1994 (1047 to 3443)	0.47% (-50.74 to 95.43)	1172.9 (664.8 to 1820.4)	557.3 (292.6 to 962.3)	-2.04% (-2.21 to -1.87)
Guinea-Bissau	172 (87 to 295)	110 (54 to 202)	-35.68% (-72.32 to 40.09)	626.5 (319 to 1074.5)	187 (92 to 341.1)	-3.74% (-4.1 to -3.38)

Location	DALYs in 1990 (95% UI)	DALYs in 2019 (95% UI)	Relative change (%, 95 CI)	Age-standardized DALYs rate in 1990 (95% UI)	Age-standardized DALYs rate in 2019 (95% UI)	EAPC (%, 95 CI)
Guyana	15 (8 to 25)	5 (3 to 9)	-65.7% (-84.67 to -20.55)	64.1 (34.6 to 108.6)	20.6 (11.2 to 36)	-3.92% (-4.51 to -3.33)
Haiti	2001 (931 to 3645)	3502 (1897 to 5931)	74.98% (-18.24 to 275.15)	1037.5 (482.5 to 1889.6)	737.7 (399.8 to 1249.4)	-0.42% (-0.76 to -0.08)
Honduras	854 (471 to 1376)	155 (70 to 296)	-81.89% (-92.18 to -59.76)	714.9 (394.7 to 1152.3)	47.3 (21.4 to 90.4)	-9.67% (-9.99 to -9.36)
India	71816 (40720 to 111827)	12982 (7386 to 21840)	-81.92% (-90.84 to -63.93)	282.3 (160.1 to 439.6)	26.2 (14.9 to 44)	-8.32% (-9.03 to -7.61)
Indonesia	8763 (4764 to 14786)	1643 (968 to 2637)	-81.25% (-90.76 to -60.39)	159 (86.5 to 268.4)	16.2 (9.5 to 26)	-7.4% (-7.75 to -7.05)
Iran (Islamic Republic of)	1199 (795 to 1731)	212 (134 to 323)	-82.36% (-89.7 to -69.51)	84.9 (56.3 to 122.6)	5.1 (3.2 to 7.8)	-9.56% (-10.39 to -8.72)
Iraq	85 (44 to 149)	60 (30 to 104)	-29.15% (-67.67 to 43.39)	20.1 (10.3 to 35.2)	4.9 (2.4 to 8.4)	-4.96% (-5.57 to -4.35)
Jamaica	24 (14 to 41)	6 (3 to 10)	-76.53% (-88.96 to -50.07)	36.6 (21.3 to 62.4)	5.5 (3 to 9.3)	-8.24% (-9.44 to -7.02)
Jordan	105 (56 to 170)	42 (22 to 74)	-59.89% (-82.89 to -6.22)	129.7 (69.1 to 211.1)	11.3 (6 to 19.7)	-9.56% (-10.14 to -8.97)

Location	DALYs in 1990 (95% UI)	DALYs in 2019 (95% UI)	Relative change (%, 95 CI)	Age-standardized DALYs rate in 1990 (95% UI)	Age-standardized DALYs rate in 2019 (95% UI)	EAPC (%, 95 CI)
Kazakhstan	23 (12 to 39)	42 (23 to 68)	80.24% (-10.64 to 285.93)	4 (2.1 to 6.7)	6.2 (3.4 to 10)	2.91% (2.24 to 3.59)
Kenya	1750 (1034 to 2790)	2262 (1246 to 3800)	29.23% (-28.7 to 144.64)	353.2 (208.7 to 563.1)	145.3 (80.1 to 244.1)	-2.74% (-3.44 to -2.04)
Kiribati	6 (3 to 11)	7 (3 to 13)	18.09% (-51.31 to 192.96)	275.3 (137.4 to 510.8)	177.6 (84.3 to 328.1)	-1.67% (-1.87 to -1.46)
Kyrgyzstan	25 (15 to 39)	43 (26 to 66)	70.41% (-11.67 to 229.97)	18.9 (11.1 to 29.6)	20.2 (12.2 to 31.4)	2.75% (1.67 to 3.84)
Lao People's Democratic Republic	649 (357 to 1087)	75 (38 to 133)	-88.41% (-94.7 to -76.12)	601.8 (330.8 to 1008.6)	30.9 (15.8 to 54.4)	-10.12% (-10.69 to -9.54)
Lebanon	35 (19 to 63)	7 (4 to 13)	-79.41% (-91.88 to -54.18)	37 (19.5 to 65.8)	3.4 (1.7 to 6.2)	-8.17% (-8.77 to -7.56)
Lesotho	288 (149 to 476)	181 (81 to 349)	-37.17% (-75.54 to 46.74)	636.7 (328.2 to 1051.8)	250.3 (111.7 to 482.7)	-1.62% (-2.37 to -0.86)
Liberia	769 (433 to 1229)	1228 (647 to 2066)	59.77% (-19.23 to 213.08)	1344.2 (757.6 to 2148.7)	803.8 (423.5 to 1352.1)	-3.07% (-4.05 to -2.09)

Location	DALYs in 1990 (95% UI)	DALYs in 2019 (95% UI)	Relative change (%, 95 CI)	Age-standardized DALYs rate in 1990 (95% UI)	Age-standardized DALYs rate in 2019 (95% UI)	EAPC (%, 95 CI)
Libya	41<br>(21 to 70)	19<br>(9 to 34)	-53.28%<br>(-79.31 to 2.17)	46.3<br>(23.6 to 80)	6.4<br>(3.2 to 11.5)	-6.96%<br>(-7.88 to -6.02)
Madagascar	1913<br>(1092 to 3103)	2890<br>(1500 to 5095)	51.09%<br>(-30.06 to 231.79)	657<br>(374.9 to 1065.6)	376<br>(195.2 to 662.8)	-1.97%<br>(-2.45 to -1.49)
Malawi	1306<br>(722 to 2064)	1025<br>(509 to 1829)	-21.57%<br>(-65.42 to 70.94)	559.6<br>(309.5 to 884.1)	195<br>(96.8 to 347.9)	-3.59%<br>(-4.08 to -3.1)
Malaysia	552<br>(303 to 919)	138<br>(74 to 240)	-74.98%<br>(-89.23 to -45.32)	97.1<br>(53.2 to 161.5)	12.4<br>(6.6 to 21.4)	-6.93%<br>(-7.45 to -6.4)
Maldives	5<br>(3 to 9)	1<br>(1 to 2)	-80.95%<br>(-90.79 to -56.24)	124.6<br>(61.7 to 215.8)	5.6<br>(2.9 to 9.6)	-10.91%<br>(-11.78 to -10.02)
Mali	4770<br>(2928 to 7316)	3351<br>(1735 to 5862)	-29.74%<br>(-65.1 to 29.77)	2173.9<br>(1334.6 to 3334.5)	632.2<br>(327.3 to 1105.7)	-4.44%<br>(-4.72 to -4.17)
Marshall Islands	3<br>(1 to 5)	3<br>(1 to 5)	-3.66%<br>(-61.96 to 133.18)	247.2<br>(120.1 to 434)	133.3<br>(52.7 to 250.2)	-2.45%<br>(-3.15 to -1.76)
Mauritania	1222<br>(716 to 1928)	302<br>(154 to 531)	-75.32%<br>(-88.04 to -52.45)	2203.5<br>(1291.2 to 3477.6)	263.7<br>(134.7 to 464.4)	-7.63%<br>(-7.86 to -7.4)
Mexico	911<br>(564 to 1372)	387<br>(239 to 597)	-57.53%<br>(-77.55 to -24.42)	37.2<br>(23.1 to 56.1)	8.3<br>(5.2 to 12.9)	-4.8%<br>(-5.27 to -4.33)

Location	DALYs in 1990 (95% UI)	DALYs in 2019 (95% UI)	Relative change (%, 95 CI)	Age-standardized DALYs rate in 1990 (95% UI)	Age-standardized DALYs rate in 2019 (95% UI)	EAPC (%, 95 CI)
Micronesia (Federated States of)	12 (6 to 21)	3 (0 to 7)	-75.87% (-97.24 to -28.5)	434.4 (211.3 to 786)	93.3 (11 to 214.9)	-5.94% (-6.25 to -5.63)
Mongolia	257 (136 to 421)	61 (27 to 113)	-76.12% (-90.05 to -47.73)	502.3 (265.3 to 823.5)	45.3 (19.9 to 83.6)	-8.87% (-9.38 to -8.37)
Montenegro	0 (0 to 1)	0 (0 to 1)	14.83% (-85.19 to 489.58)	0.9 (0.2 to 2.2)	1 (0.1 to 2.6)	0.53% (0.43 to 0.63)
Morocco	2622 (1404 to 4466)	239 (115 to 455)	-90.88% (-96.01 to -79.79)	355.1 (190.1 to 604.8)	17.7 (8.5 to 33.7)	-9.99% (-10.4 to -9.57)
Mozambique	2526 (1317 to 4202)	1009 (458 to 1833)	-60.07% (-83.38 to -12.03)	673.5 (351.2 to 1120.4)	132.9 (60.3 to 241.4)	-5.03% (-5.54 to -4.52)
Myanmar	2977 (1436 to 5380)	1131 (584 to 1961)	-62.02% (-82.84 to -12.9)	239.6 (115.6 to 433)	55.1 (28.4 to 95.5)	-5.56% (-6.1 to -5.01)
Namibia	116 (54 to 202)	44 (21 to 88)	-61.82% (-82.97 to -1.05)	323.1 (151.1 to 563.5)	55.6 (25.9 to 110.4)	-5.89% (-6.5 to -5.28)
Nepal	3459 (1818 to 5742)	338 (157 to 610)	-90.23% (-95.68 to -79.52)	650.8 (342 to 1080.3)	30.8 (14.3 to 55.7)	-10.42% (-10.6 to -10.24)
Nicaragua	112 (61 to 184)	21 (12 to 36)	-80.85% (-90.7 to -59.03)	114.4 (62 to 189.1)	9.1 (5.1 to 15.3)	-9.16% (-9.66 to -8.66)

Location	DALYs in 1990 (95% UI)	DALYs in 2019 (95% UI)	Relative change (%, 95 CI)	Age-standardized DALYs rate in 1990 (95% UI)	Age-standardized DALYs rate in 2019 (95% UI)	EAPC (%, 95 CI)
Niger	2928 (1632 to 4634)	4801 (2559 to 8179)	63.98% (-15.06 to 213.38)	1677.8 (935.4 to 2655.5)	977.6 (521 to 1665.3)	-2.15% (-2.39 to -1.9)
Nigeria	5893 (2918 to 10467)	7010 (3480 to 12603)	18.95% (-44.51 to 164.88)	286.4 (141.8 to 508.7)	120.6 (59.9 to 216.7)	-3.74% (-4.47 to -3)
North Macedonia	5 (3 to 8)	1 (0 to 2)	-81.88% (-95.27 to -54.74)	6.2 (3.4 to 10.5)	1 (0.3 to 2.1)	-7.26% (-7.99 to -6.53)
Pakistan	13338 (7842 to 20903)	8282 (4374 to 14321)	-37.91% (-71.44 to 30.94)	515.3 (303 to 807.6)	127.4 (67.3 to 220.2)	-5.05% (-5.64 to -4.46)
Papua New Guinea	614 (296 to 1084)	774 (373 to 1401)	25.99% (-39.82 to 178.4)	568 (273.9 to 1001.7)	242.2 (116.7 to 438.2)	-2.32% (-2.73 to -1.92)
Paraguay	263 (154 to 416)	52 (27 to 90)	-80.24% (-90.92 to -56.58)	231.4 (135.7 to 365.8)	21.4 (11.2 to 36.9)	-8.63% (-9.25 to -7.99)
Peru	1669 (975 to 2726)	227 (115 to 399)	-86.42% (-93.69 to -72.96)	267.6 (156.3 to 437)	18 (9.1 to 31.6)	-10.06% (-10.64 to -9.47)
Philippines	1330 (794 to 2015)	688 (411 to 1042)	-48.26% (-72.19 to 1.01)	74.5 (44.4 to 112.8)	19 (11.3 to 28.8)	-4.43% (-4.57 to -4.29)
Republic of Moldova	29 (17 to 45)	3 (1 to 7)	-88.34% (-96.32 to -74.51)	15.8 (9.4 to 24.3)	2.2 (0.7 to 4.7)	-6.56% (-7.47 to -5.64)

Location	DALYs in 1990 (95% UI)	DALYs in 2019 (95% UI)	Relative change (%, 95 CI)	Age-standardized DALYs rate in 1990 (95% UI)	Age-standardized DALYs rate in 2019 (95% UI)	EAPC (%, 95 CI)
Russian Federation	455 (301 to 665)	281 (150 to 510)	-38.16% (-66.65 to 7.3)	7.5 (4.9 to 10.9)	4.7 (2.5 to 8.5)	-1.35% (-1.76 to -0.94)
Rwanda	4535 (2814 to 7148)	1515 (805 to 2613)	-66.6% (-84.58 to -32.07)	2396.9 (1487.3 to 3778)	362.4 (192.6 to 625.3)	-7.05% (-7.89 to -6.2)
Saint Lucia	1 (0 to 1)	0 (0 to 1)	-49.56% (-75.08 to -2.47)	21.6 (12.9 to 34.2)	5.9 (3.3 to 9.5)	-3.34% (-4.47 to -2.19)
Saint Vincent and the Grenadines	0 (0 to 0)	0 (0 to 0)	-1.53% (-51.73 to 81.52)	5.9 (3.3 to 9.9)	4.1 (2 to 7)	-1.03% (-1.48 to -0.58)
Samoa	4 (2 to 8)	1 (0 to 2)	-74.23% (-90.93 to -31.54)	101.8 (46.6 to 199.5)	17.7 (7.1 to 33.5)	-7.04% (-7.62 to -6.46)
Senegal	1359 (749 to 2162)	1156 (593 to 1961)	-14.92% (-60.06 to 66.03)	743.2 (409.8 to 1182.6)	270.6 (138.7 to 459)	-3.15% (-3.5 to -2.81)
Serbia	10 (5 to 17)	3 (1 to 6)	-70.03% (-91.35 to -27.36)	2.9 (1.6 to 5)	1 (0.3 to 2.1)	-3.74% (-4.21 to -3.27)
Sierra Leone	647 (336 to 1081)	1108 (574 to 1889)	71.12% (-22.64 to 235.06)	633 (328.9 to 1057.3)	469.8 (243.3 to 801)	-0.82% (-1.58 to -0.06)
Solomon Islands	67 (30 to 125)	116 (53 to 210)	71.91% (-28.02 to 309.22)	900.7 (397.7 to 1663.2)	561.3 (256.9 to 1019.2)	-1.16% (-1.8 to -0.52)

Location	DALYs in 1990 (95% UI)	DALYs in 2019 (95% UI)	Relative change (%, 95 CI)	Age-standardized DALYs rate in 1990 (95% UI)	Age-standardized DALYs rate in 2019 (95% UI)	EAPC (%, 95 CI)
Somalia	3931 (1733 to 6779)	8558 (4338 to 14283)	117.7% (1.26 to 395)	1817.3 (801 to 3133.9)	1652 (837.5 to 2757.3)	-0.18% (-0.39 to 0.03)
South Africa	1807 (1046 to 2825)	587 (291 to 1036)	-67.54% (-84.99 to -28.96)	159.9 (92.6 to 249.9)	27.2 (13.5 to 48)	-5.09% (-6.59 to -3.56)
South Sudan	490 (237 to 934)	673 (309 to 1273)	37.34% (-42.19 to 200.89)	387.5 (187.1 to 738.8)	247 (113.6 to 467.3)	-1.66% (-1.85 to -1.47)
Sri Lanka	118 (64 to 195)	36 (17 to 65)	-69.39% (-86.8 to -35.99)	19.6 (10.7 to 32.5)	4.3 (2.1 to 7.7)	-4.88% (-5.09 to -4.66)
Sudan	7752 (4518 to 12487)	2070 (912 to 3980)	-73.3% (-88.95 to -42.42)	1474.8 (859.5 to 2375.7)	155.7 (68.6 to 299.4)	-7.78% (-8.21 to -7.35)
Suriname	4 (2 to 6)	4 (2 to 7)	9.44% (-53.54 to 163.46)	35 (18.2 to 61.3)	20 (9.9 to 35.2)	-1.49% (-2.16 to -0.82)
Syrian Arab Republic	240 (121 to 421)	48 (23 to 83)	-79.96% (-91.53 to -52.4)	85.9 (43.5 to 150.9)	8.5 (4.1 to 14.6)	-9.13% (-9.93 to -8.33)
Tajikistan	32 (18 to 52)	16 (7 to 28)	-51.78% (-79.19 to -1.68)	24.6 (13.9 to 40.2)	5.2 (2.4 to 9.1)	-6.15% (-6.6 to -5.69)
Thailand	175 (86 to 313)	127 (62 to 230)	-27.52% (-69.16 to 73.98)	8.7 (4.2 to 15.5)	4.6 (2.3 to 8.4)	-1.03% (-1.61 to -0.44)

Location	DALYs in 1990 (95% UI)	DALYs in 2019 (95% UI)	Relative change (%, 95 CI)	Age-standardized DALYs rate in 1990 (95% UI)	Age-standardized DALYs rate in 2019 (95% UI)	EAPC (%, 95 CI)
Timor-Leste	189 (93 to 332)	61 (8 to 113)	-67.59% (-95.49 to -17.54)	865.4 (425.3 to 1525.1)	193.3 (26.5 to 356.7)	-6.53% (-7.28 to -5.76)
Togo	725 (395 to 1168)	747 (387 to 1352)	3.09% (-51.03 to 109.24)	770.3 (419.9 to 1240.5)	280.6 (145.1 to 507.6)	-3.1% (-3.53 to -2.67)
Tonga	4 (2 to 7)	2 (1 to 3)	-56.65% (-81.34 to -2.69)	169.3 (86.3 to 285.4)	53.8 (25.4 to 99.4)	-4.17% (-4.28 to -4.05)
Tunisia	165 (87 to 279)	32 (16 to 58)	-80.37% (-91.14 to -56.03)	71.8 (38.1 to 121.5)	6.5 (3.2 to 11.6)	-7.52% (-8.12 to -6.9)
Turkey	2013 (1042 to 3386)	180 (97 to 302)	-91.07% (-95.76 to -80.53)	115.7 (59.9 to 194.7)	5.5 (3 to 9.2)	-11.61% (-12.62 to -10.59)
Turkmenistan	22 (12 to 35)	12 (6 to 20)	-43.75% (-70.64 to 2.21)	20.5 (11.7 to 32.5)	7.3 (3.9 to 12.2)	-3.42% (-3.69 to -3.15)
Tuvalu	1 (1 to 2)	0 (0 to 0)	-84.62% (-94.13 to -65.29)	397.3 (193 to 728.6)	61.7 (26.6 to 123.5)	-6.07% (-6.24 to -5.9)
Uganda	1127 (508 to 2020)	1349 (712 to 2320)	19.76% (-46.09 to 184.75)	303.1 (136.6 to 543.4)	134 (70.7 to 230.3)	-3.02% (-3.6 to -2.44)
Ukraine	68 (36 to 113)	58 (25 to 110)	-14.27% (-60.99 to 69.91)	3.4 (1.8 to 5.7)	3.2 (1.4 to 6)	0.83% (0.31 to 1.36)

Location	DALYs in 1990 (95% UI)	DALYs in 2019 (95% UI)	Relative change (%, 95 CI)	Age-standardized DALYs rate in 1990 (95% UI)	Age-standardized DALYs rate in 2019 (95% UI)	EAPC (%, 95 CI)
United Republic of Tanzania	2943 (1651 to 4753)	2502 (1364 to 4222)	-15% (-60.56 to 77.34)	473.7 (265.7 to 765.1)	156.8 (85.5 to 264.6)	-3% (-3.39 to -2.6)
Uzbekistan	234 (143 to 357)	98 (53 to 159)	-58.38% (-78.66 to -16.12)	39 (23.7 to 59.4)	8 (4.4 to 13.1)	-4.15% (-4.83 to -3.47)
Vanuatu	9 (4 to 16)	11 (4 to 22)	30.74% (-55.58 to 238.33)	208.4 (91.8 to 394.4)	128.5 (50.3 to 248)	-2.51% (-2.93 to -2.09)
Venezuela (Bolivarian Republic of)	692 (447 to 986)	299 (173 to 474)	-56.84% (-77.96 to -17.12)	112.9 (72.9 to 160.9)	27.3 (15.8 to 43.4)	-5.13% (-5.93 to -4.32)
Viet Nam	411 (197 to 729)	69 (23 to 151)	-83.11% (-95.03 to -59.32)	20.4 (9.8 to 36.3)	1.7 (0.6 to 3.8)	-8.48% (-9.05 to -7.9)
Yemen	3136 (1465 to 5511)	3554 (1530 to 6520)	13.33% (-54.8 to 159.82)	932.5 (435.4 to 1638.6)	346.5 (149.1 to 635.6)	-4.3% (-4.59 to -4.01)
Zambia	587 (323 to 997)	390 (201 to 697)	-33.58% (-69.1 to 44.11)	349.2 (192.2 to 593.1)	76.2 (39.3 to 136.2)	-6.1% (-6.85 to -5.35)
Zimbabwe	851 (477 to 1376)	1920 (982 to 3318)	125.51% (6.92 to 368.19)	331.2 (185.6 to 535.4)	385.3 (197 to 665.8)	4.04% (2.46 to 5.66)

CI – confidence interval, DALYs – disability-adjusted life years (per 100,000 population), EAPC – estimated annual percentage change, MSMI – maternal sepsis and other maternal infections, UI – uncertain interval.

Table S31. DALYs of MSMI in 131 low- and middle-income countries and territories with EAPC in age group of 40-44 years from 1990 and 2019.

Location	DALYs in 1990 (95% UI)	DALYs in 2019 (95% UI)	Relative change (%, 95 CI)	Age-standardized DALYs rate in 1990 (95% UI)	Age-standardized DALYs rate in 2019 (95% UI)	EAPC (%, 95 CI)
Afghanistan	1540 (704 to 2855)	1498 (682 to 2865)	-2.69% (-59.34 to 140.58)	624.2 (285.1 to 1157.3)	224.6 (102.3 to 429.4)	-4.29% (-5.34 to -3.23)
Albania	2 (1 to 3)	1 (0 to 1)	-60.29% (-82.76 to -12.24)	2.7 (1.5 to 4.6)	1 (0.5 to 1.8)	-1.9% (-2.89 to -0.89)
Algeria	740 (341 to 1366)	149 (75 to 261)	-79.88% (-91.39 to -53.76)	164.9 (75.9 to 304.2)	10.4 (5.3 to 18.3)	-9.21% (-9.73 to -8.68)
American Samoa	1 (0 to 1)	0 (0 to 1)	-54.84% (-83.29 to 24.36)	60.6 (28.8 to 111.8)	18.3 (8.2 to 34.8)	-4.56% (-4.77 to -4.36)
Angola	967 (435 to 1871)	1145 (537 to 2150)	18.45% (-49.9 to 178.77)	502.3 (226.3 to 972.1)	164.1 (77 to 308.1)	-3.84% (-4.15 to -3.52)
Armenia	2 (1 to 3)	1 (1 to 2)	-42% (-70.04 to 12.04)	2.2 (1.3 to 3.5)	1 (0.6 to 1.6)	-2.88% (-3.6 to -2.16)
Azerbaijan	19 (10 to 33)	6 (3 to 10)	-70.82% (-87.22 to -35.46)	13.7 (7 to 23.9)	1.6 (0.8 to 3)	-8.01% (-8.67 to -7.34)
Bangladesh	1155 (589 to 2067)	121 (68 to 206)	-89.53% (-95.53 to -75.36)	57.4 (29.3 to 102.7)	2.3 (1.3 to 4)	-10.39% (-11.26 to -9.52)

Location	DALYs in 1990 (95% UI)	DALYs in 2019 (95% UI)	Relative change (%, 95 CI)	Age-standardized DALYs rate in 1990 (95% UI)	Age-standardized DALYs rate in 2019 (95% UI)	EAPC (%, 95 CI)
Belarus	6 (4 to 9)	3 (1 to 6)	-56.06% (-89.58 to 9.84)	2 (1.2 to 3)	0.8 (0.2 to 1.7)	-2.68% (-3.42 to -1.94)
Belize	1 (0 to 1)	1 (1 to 2)	77.62% (-8.01 to 238.73)	21.9 (13.5 to 33.1)	10.1 (5.8 to 16.2)	-2.25% (-2.85 to -1.64)
Benin	730 (398 to 1220)	689 (334 to 1257)	-5.61% (-55.16 to 102.44)	836.4 (455.8 to 1398)	247.7 (120.2 to 452)	-3.56% (-3.91 to -3.2)
Bhutan	6 (3 to 12)	1 (0 to 2)	-85.76% (-93.85 to -66.15)	56.8 (25.8 to 107.7)	4.2 (2.2 to 7.7)	-8.75% (-8.98 to -8.52)
Bolivia (Plurinational State of)	732 (396 to 1247)	367 (169 to 671)	-49.9% (-80.18 to 14.43)	495.3 (268.3 to 844.2)	104.6 (48.3 to 191.5)	-5.61% (-6 to -5.22)
Bosnia and Herzegovina	8 (4 to 14)	0 (0 to 0)	-96.61% (-98.53 to -91.63)	5.8 (2.9 to 10.1)	0.2 (0.1 to 0.4)	-13.63% (-15.33 to -11.89)
Botswana	26 (11 to 49)	10 (5 to 19)	-61.61% (-84.67 to -1.24)	103.2 (44.7 to 194.7)	13.2 (6.4 to 25.6)	-7.35% (-8.25 to -6.43)
Brazil	1647 (1007 to 2461)	641 (418 to 937)	-61.1% (-79.11 to -29.21)	41.9 (25.6 to 62.6)	7.9 (5.2 to 11.6)	-4.75% (-5.37 to -4.12)
Bulgaria	33 (19 to 54)	4 (2 to 6)	-87.78% (-94.01 to -74.37)	10 (5.7 to 16.4)	1.5 (0.9 to 2.5)	-7.63% (-8.5 to -6.74)
Burkina Faso	1018 (530 to 1808)	766 (377 to 1435)	-24.71% (-66.69 to 61.32)	524.7 (273.2 to 932.1)	149.6 (73.5 to 280)	-5.13% (-5.58 to -4.67)

Location	DALYs in 1990 (95% UI)	DALYs in 2019 (95% UI)	Relative change (%, 95 CI)	Age-standardized DALYs rate in 1990 (95% UI)	Age-standardized DALYs rate in 2019 (95% UI)	EAPC (%, 95 CI)
Burundi	1219<br>(587 to 2174)	1715<br>(896 to 2977)	40.71%<br>(-39.24 to 203.35)	1167.3<br>(562.2 to 2082.5)	742.4<br>(387.9 to 1288.9)	-2.08%<br>(-2.54 to -1.62)
Cabo Verde	3<br>(2 to 5)	1<br>(0 to 1)	-73.34%<br>(-87.67 to -40.34)	55.9<br>(30 to 96.5)	5.3<br>(2.8 to 8.7)	-8.78%<br>(-9.08 to -8.48)
Cambodia	1019<br>(447 to 1935)	298<br>(131 to 566)	-70.79%<br>(-88.53 to -25.85)	443<br>(194.4 to 841.3)	64.1<br>(28.3 to 121.9)	-7.6%<br>(-8.6 to -6.59)
Cameroon	1048<br>(559 to 1698)	1063<br>(480 to 2001)	1.48%<br>(-56.19 to 129.15)	520.7<br>(277.7 to 843.8)	165.1<br>(74.5 to 310.7)	-3.76%<br>(-4.22 to -3.29)
Central African Republic	715<br>(354 to 1249)	1617<br>(775 to 2931)	126.23%<br>(0.62 to 414.58)	1277.8<br>(632.9 to 2231.6)	1309.2<br>(627.7 to 2372.5)	0.18%<br>(0 to 0.37)
Chad	1450<br>(758 to 2379)	1886<br>(918 to 3361)	30.04%<br>(-37.01 to 172.33)	1256.1<br>(656.7 to 2061.2)	624.1<br>(303.7 to 1112.4)	-2.47%<br>(-2.82 to -2.12)
China	3658<br>(2815 to 4717)	255<br>(158 to 400)	-93.02%<br>(-95.81 to -88.29)	11.4<br>(8.8 to 14.7)	0.5<br>(0.3 to 0.8)	-9.6%<br>(-10.39 to -8.8)
Colombia	223<br>(129 to 357)	94<br>(53 to 155)	-57.88%<br>(-79.41 to -15.14)	27.5<br>(16 to 44.1)	6<br>(3.4 to 10)	-5.13%<br>(-5.65 to -4.61)
Comoros	25<br>(7 to 49)	19<br>(9 to 36)	-24.48%<br>(-71.22 to 159.72)	277.2<br>(75.8 to 537.8)	93.4<br>(43.5 to 179)	-4.18%<br>(-4.54 to -3.83)
Congo	168<br>(79 to 301)	209<br>(92 to 399)	24.45%<br>(-51.59 to 209.34)	385.4<br>(181.3 to 690.2)	135.1<br>(59.3 to 257.5)	-3.15%<br>(-3.74 to -2.55)

Location	DALYs in 1990 (95% UI)	DALYs in 2019 (95% UI)	Relative change (%, 95 CI)	Age-standardized DALYs rate in 1990 (95% UI)	Age-standardized DALYs rate in 2019 (95% UI)	EAPC (%, 95 CI)
Costa Rica	14 (8 to 22)	4 (2 to 7)	-69% (-84.52 to -37.96)	18.6 (10.9 to 29.1)	2.7 (1.5 to 4.4)	-7.4% (-8.41 to -6.37)
Côte d'Ivoire	669 (341 to 1171)	769 (340 to 1501)	14.84% (-49.22 to 166.89)	312.1 (159.2 to 546)	130.5 (57.8 to 254.8)	-2.76% (-3.11 to -2.4)
Cuba	27 (15 to 43)	10 (6 to 16)	-63.86% (-82.19 to -23.99)	7.9 (4.4 to 12.5)	2.8 (1.6 to 4.5)	-3.54% (-3.93 to -3.14)
Democratic People's Republic of Korea	192 (84 to 407)	190 (79 to 365)	-0.87% (-65.73 to 168.8)	32.4 (14.1 to 68.7)	22.7 (9.4 to 43.4)	-0.78% (-1.76 to 0.21)
Democratic Republic of the Congo	6870 (3375 to 12024)	22179 (11723 to 37420)	222.85% (41.21 to 601.97)	967.8 (475.4 to 1693.8)	1214.6 (642 to 2049.2)	2.32% (1.07 to 3.58)
Djibouti	49 (22 to 91)	94 (41 to 187)	92.66% (-24.9 to 380.84)	553.9 (245.1 to 1029.2)	251.8 (110.5 to 498)	-2.41% (-2.84 to -1.97)
Dominica	1 (0 to 1)	0 (0 to 1)	-27.35% (-69.71 to 67.2)	35.2 (17.9 to 60.4)	21.2 (10.8 to 37.5)	-0.89% (-1.46 to -0.32)
Dominican Republic	38 (21 to 66)	28 (13 to 50)	-28.4% (-71.09 to 77.57)	23.6 (12.9 to 40.4)	8.3 (3.9 to 15.2)	-2.91% (-3.58 to -2.22)
Ecuador	202 (119 to 322)	63 (32 to 114)	-68.92% (-86.6 to -32.97)	86.6 (51.1 to 138.2)	11.6 (6 to 21.2)	-6.06% (-6.87 to -5.23)

Location	DALYs in 1990 (95% UI)	DALYs in 2019 (95% UI)	Relative change (%, 95 CI)	Age-standardized DALYs rate in 1990 (95% UI)	Age-standardized DALYs rate in 2019 (95% UI)	EAPC (%, 95 CI)
Egypt	1218 (642 to 1976)	106 (53 to 188)	-91.25% (-96.2 to -80.62)	92.5 (48.8 to 150.2)	3.9 (1.9 to 6.8)	-11.49% (-12.01 to -10.97)
El Salvador	209 (106 to 365)	15 (7 to 27)	-92.95% (-96.88 to -82.3)	179 (90.5 to 312.2)	6.8 (3.2 to 12.6)	-10.75% (-12.2 to -9.27)
Equatorial Guinea	130 (63 to 234)	13 (6 to 28)	-89.76% (-95.88 to -74.02)	1588.7 (768.9 to 2854.9)	45.9 (19.4 to 96.8)	-12.93% (-13.67 to -12.18)
Eritrea	781 (374 to 1405)	711 (339 to 1313)	-8.97% (-58.57 to 114.08)	1408.7 (675.3 to 2536.5)	448.9 (214.1 to 829.2)	-2.96% (-3.53 to -2.38)
Eswatini	30 (14 to 54)	13 (5 to 26)	-55.95% (-83.52 to 13.83)	183.4 (89.2 to 333.3)	45.6 (18.1 to 92.5)	-3.7% (-4.9 to -2.5)
Ethiopia	21493 (11806 to 34654)	7758 (4409 to 13140)	-63.91% (-81.61 to -27.09)	2357.9 (1295.1 to 3801.7)	329.7 (187.4 to 558.4)	-7.08% (-7.85 to -6.3)
Fiji	7 (3 to 14)	4 (2 to 8)	-41.09% (-77.84 to 56.89)	35.7 (17.2 to 68.7)	15 (7.1 to 29.3)	-2.74% (-2.98 to -2.5)
Gabon	46 (23 to 82)	27 (12 to 51)	-41.35% (-75.58 to 33.38)	313.8 (159.9 to 561.7)	53.5 (24.2 to 102.3)	-6.25% (-6.96 to -5.53)
Gambia	109 (58 to 191)	177 (91 to 307)	62.18% (-22.47 to 244.22)	632.5 (334.1 to 1102.8)	350.3 (180.4 to 606.6)	-2.32% (-2.58 to -2.05)

Location	DALYs in 1990 (95% UI)	DALYs in 2019 (95% UI)	Relative change (%, 95 CI)	Age-standardized DALYs rate in 1990 (95% UI)	Age-standardized DALYs rate in 2019 (95% UI)	EAPC (%, 95 CI)
Georgia	4 (2 to 7)	5 (3 to 9)	33.09% (-34.23 to 183.63)	2.6 (1.5 to 4.5)	4.4 (2.4 to 7.5)	3.69% (2.14 to 5.27)
Ghana	555 (278 to 982)	289 (145 to 510)	-47.86% (-76.55 to 15.91)	182.3 (91.2 to 322.7)	33.1 (16.6 to 58.4)	-5.34% (-5.88 to -4.8)
Global	143587 (117670 to 170145)	99662 (80765 to 125155)	-30.59% (-46.33 to -10.74)	102.5 (84 to 121.4)	40.7 (33 to 51.1)	-3.18% (-3.39 to -2.97)
Grenada	0 (0 to 0)	0 (0 to 0)	-25.64% (-66.74 to 50.05)	5.6 (3.2 to 8.8)	2.1 (0.9 to 4)	-2.63% (-3.2 to -2.07)
Guatemala	419 (232 to 665)	153 (83 to 276)	-63.48% (-82.08 to -21.58)	258.2 (142.8 to 409)	29.3 (15.8 to 52.7)	-8.94% (-10.12 to -7.74)
Guinea	1276 (652 to 2166)	1182 (563 to 2087)	-7.38% (-57.02 to 102.1)	925.2 (473.1 to 1570.5)	434.1 (206.7 to 766.6)	-2.12% (-2.29 to -1.95)
Guinea-Bissau	108 (53 to 197)	69 (32 to 125)	-36.23% (-74.59 to 43.95)	532.1 (259.9 to 970.4)	159.2 (73.2 to 288)	-3.73% (-4.1 to -3.36)
Guyana	5 (3 to 9)	2 (1 to 4)	-58.48% (-81.49 to -6.64)	30.3 (15.9 to 51.9)	9 (4.6 to 15.1)	-3.3% (-3.98 to -2.62)
Haiti	982 (458 to 1871)	2112 (908 to 3909)	115.04% (-15.53 to 437.47)	708.5 (330.2 to 1349.4)	563.5 (242.3 to 1042.7)	0.06% (-0.43 to 0.56)

Location	DALYs in 1990 (95% UI)	DALYs in 2019 (95% UI)	Relative change (%, 95 CI)	Age-standardized DALYs rate in 1990 (95% UI)	Age-standardized DALYs rate in 2019 (95% UI)	EAPC (%, 95 CI)
Honduras	316<br>(168 to 510)	79<br>(35 to 163)	-74.85%<br>(-89.77 to -42.98)	351.4<br>(186.9 to 567.4)	28.4<br>(12.4 to 58.3)	-9.08%<br>(-9.4 to -8.76)
India	32552<br>(19153 to 49600)	6342<br>(3640 to 10188)	-80.52%<br>(-90.24 to -62.68)	159<br>(93.5 to 242.3)	14.9<br>(8.6 to 23.9)	-8.3%<br>(-8.79 to -7.81)
Indonesia	4176<br>(2364 to 6989)	686<br>(393 to 1088)	-83.58%<br>(-91.94 to -65.59)	94.2<br>(53.3 to 157.6)	7.1<br>(4.1 to 11.3)	-8.84%<br>(-9.14 to -8.54)
Iran (Islamic Republic of)	539<br>(347 to 786)	70<br>(44 to 105)	-87.08%<br>(-92.51 to -76.52)	49.6<br>(31.9 to 72.3)	2.2<br>(1.4 to 3.3)	-10.76%<br>(-11.56 to -9.96)
Iraq	52<br>(25 to 92)	35<br>(18 to 65)	-31.36%<br>(-70.8 to 57.85)	15.3<br>(7.5 to 27.3)	3.1<br>(1.6 to 5.7)	-5.32%<br>(-6.11 to -4.52)
Jamaica	7<br>(4 to 11)	2<br>(1 to 3)	-70.32%<br>(-87.92 to -35.31)	13.3<br>(7.3 to 21.8)	2.2<br>(1 to 3.8)	-7.78%<br>(-8.99 to -6.55)
Jordan	69<br>(36 to 121)	22<br>(10 to 43)	-67.74%<br>(-87.36 to -18.11)	107.2<br>(56.2 to 188.4)	7.1<br>(3.3 to 13.6)	-10.88%<br>(-11.61 to -10.14)
Kazakhstan	8<br>(4 to 14)	17<br>(9 to 27)	118.45%<br>(-9.28 to 440.45)	1.9<br>(0.9 to 3.3)	2.7<br>(1.4 to 4.3)	2.32%<br>(1.67 to 2.97)
Kenya	1087<br>(604 to 1752)	1565<br>(843 to 2597)	44.05%<br>(-24.72 to 171.73)	282.7<br>(157.2 to 455.8)	127.2<br>(68.6 to 211.1)	-2.32%<br>(-3.04 to -1.59)

Location	DALYs in 1990 (95% UI)	DALYs in 2019 (95% UI)	Relative change (%, 95 CI)	Age-standardized DALYs rate in 1990 (95% UI)	Age-standardized DALYs rate in 2019 (95% UI)	EAPC (%, 95 CI)
Kiribati	3 (1 to 5)	3 (2 to 7)	27.69% (-53.11 to 241.28)	149.6 (73.2 to 266.1)	94.6 (43.9 to 188.4)	-1.68% (-1.91 to -1.45)
Kyrgyzstan	9 (5 to 14)	15 (9 to 23)	69.93% (-12.62 to 227.94)	10.5 (6.2 to 16.5)	8.1 (4.9 to 12.6)	1.47% (0.38 to 2.57)
Lao People's Democratic Republic	234 (122 to 419)	35 (16 to 68)	-85% (-94.04 to -64.03)	269.9 (140.8 to 484.5)	17.4 (8.2 to 33.7)	-9.51% (-10.14 to -8.89)
Lebanon	16 (8 to 31)	3 (1 to 5)	-84.05% (-93.29 to -59.82)	18.5 (8.9 to 35.2)	1.5 (0.8 to 2.7)	-8.91% (-9.49 to -8.33)
Lesotho	194 (97 to 338)	121 (49 to 234)	-37.81% (-77.04 to 60.47)	519.8 (260.3 to 904.7)	219.9 (89.6 to 425.3)	-1.03% (-1.95 to -0.1)
Liberia	502 (270 to 800)	972 (517 to 1570)	93.46% (-6.07 to 301.96)	1276.9 (687 to 2032.1)	766.7 (407.8 to 1238.4)	-3.02% (-4.03 to -2)
Libya	28 (14 to 53)	20 (9 to 34)	-29.04% (-72.75 to 65.29)	40.4 (20 to 77.7)	7 (3.4 to 12.4)	-5.59% (-6.25 to -4.92)
Madagascar	1072 (607 to 1775)	2058 (995 to 3567)	91.99% (-13.44 to 291.59)	489.3 (277.3 to 810.3)	321.1 (155.2 to 556.6)	-1.49% (-1.96 to -1.02)
Malawi	1082 (576 to 1855)	813 (397 to 1464)	-24.86% (-67.97 to 64.75)	573 (305 to 982.6)	209.2 (102.2 to 376.9)	-3.43% (-3.9 to -2.96)
Malaysia	342 (167 to 597)	72 (35 to 128)	-79.02% (-91.72 to -48.75)	75.5 (37 to 132)	7.6 (3.7 to 13.5)	-7.93% (-8.33 to -7.53)

Location	DALYs in 1990 (95% UI)	DALYs in 2019 (95% UI)	Relative change (%, 95 CI)	Age-standardized DALYs rate in 1990 (95% UI)	Age-standardized DALYs rate in 2019 (95% UI)	EAPC (%, 95 CI)
Maldives	3 (2 to 6)	0 (0 to 1)	-84.69% (-93.3 to -63.72)	97.2 (47.3 to 172.7)	3.7 (1.9 to 6.2)	-10.95% (-11.88 to -10.01)
Mali	4221 (2511 to 6255)	2588 (1340 to 4525)	-38.69% (-69.54 to 12.88)	2339.9 (1392.2 to 3467.9)	621.4 (321.8 to 1086.5)	-5.08% (-5.48 to -4.68)
Marshall Islands	1 (1 to 2)	1 (1 to 3)	-0.82% (-63.77 to 170.82)	164.2 (74.6 to 299.7)	75.8 (31.8 to 144.5)	-2.97% (-3.56 to -2.38)
Mauritania	798 (457 to 1290)	215 (107 to 392)	-73.04% (-87.62 to -46.12)	1956.7 (1120.6 to 3164.8)	225.3 (112 to 410.4)	-7.78% (-8 to -7.56)
Mexico	397 (247 to 612)	162 (102 to 247)	-59.08% (-77.2 to -25.99)	20.4 (12.7 to 31.4)	3.7 (2.3 to 5.7)	-5.84% (-6.35 to -5.34)
Micronesia (Federated States of)	5 (2 to 10)	1 (0 to 3)	-74.87% (-97.58 to -13.77)	273.9 (118.7 to 538.3)	46.9 (5.2 to 116.1)	-5.98% (-6.17 to -5.79)
Mongolia	96 (44 to 172)	32 (15 to 62)	-66.34% (-87.07 to -11.36)	270.3 (124 to 482.9)	26.4 (12 to 50.2)	-8.36% (-8.67 to -8.06)
Montenegro	0 (0 to 0)	0 (0 to 0)	-2.81% (-26.27 to 22.13)	0.4 (0.2 to 0.7)	0.3 (0.2 to 0.6)	-0.46% (-0.57 to -0.36)
Morocco	1326 (664 to 2301)	134 (65 to 246)	-89.92% (-95.71 to -76.5)	235.5 (117.9 to 408.6)	10.9 (5.3 to 20.2)	-10.44% (-10.95 to -9.91)
Mozambique	1792 (984 to 3015)	726 (339 to 1354)	-59.49% (-83.05 to -9.49)	595.7 (327.1 to 1002.6)	118.3 (55.4 to 220.7)	-5.03% (-5.63 to -4.43)

Location	DALYs in 1990 (95% UI)	DALYs in 2019 (95% UI)	Relative change (%, 95 CI)	Age-standardized DALYs rate in 1990 (95% UI)	Age-standardized DALYs rate in 2019 (95% UI)	EAPC (%, 95 CI)
Myanmar	800 (393 to 1432)	513 (247 to 935)	-35.86% (-73.67 to 47.6)	90 (44.2 to 161.1)	27.2 (13.1 to 49.5)	-4.53% (-5.09 to -3.97)
Namibia	78 (35 to 135)	30 (13 to 62)	-61.12% (-84.34 to 2.11)	284.8 (128.6 to 495.8)	45.5 (19.1 to 93.5)	-6.17% (-6.8 to -5.53)
Nepal	1545 (765 to 2822)	214 (99 to 406)	-86.13% (-94.58 to -67.66)	357.7 (177 to 653.3)	23.1 (10.7 to 43.8)	-9.08% (-9.43 to -8.73)
Nicaragua	45 (23 to 75)	9 (4 to 16)	-80.02% (-91.25 to -52.26)	61.3 (31 to 101.9)	4.5 (2.2 to 8.1)	-9.08% (-9.62 to -8.54)
Niger	2059 (1063 to 3509)	3028 (1496 to 5144)	47.09% (-34.15 to 208.9)	1304.8 (673.7 to 2224.2)	745.8 (368.5 to 1267)	-2.16% (-2.37 to -1.95)
Nigeria	3548 (1781 to 6223)	4561 (2347 to 7915)	28.58% (-41.25 to 174.99)	231.7 (116.3 to 406.4)	94.8 (48.8 to 164.4)	-3.76% (-4.51 to -3.01)
North Macedonia	1 (1 to 2)	0 (0 to 0)	-81.61% (-90.96 to -62.6)	1.9 (1 to 3.2)	0.3 (0.2 to 0.5)	-7.85% (-8.48 to -7.21)
Pakistan	6762 (3811 to 11357)	4429 (2276 to 7693)	-34.51% (-70.59 to 43.69)	296.4 (167.1 to 497.8)	79.2 (40.7 to 137.6)	-4.84% (-5.45 to -4.22)
Papua New Guinea	291 (134 to 520)	358 (173 to 675)	22.95% (-48.74 to 195.37)	328.3 (150.8 to 586.8)	131.9 (63.9 to 248.8)	-2.65% (-3.1 to -2.19)

Location	DALYs in 1990 (95% UI)	DALYs in 2019 (95% UI)	Relative change (%, 95 CI)	Age-standardized DALYs rate in 1990 (95% UI)	Age-standardized DALYs rate in 2019 (95% UI)	EAPC (%, 95 CI)
Paraguay	106 (62 to 171)	18 (9 to 32)	-82.61% (-92.46 to -62.6)	124.8 (72.7 to 201.5)	9.2 (4.5 to 16.1)	-9.18% (-9.75 to -8.61)
Peru	921 (500 to 1569)	138 (67 to 261)	-84.99% (-93.36 to -67.15)	180.8 (98.2 to 308.1)	12 (5.8 to 22.6)	-10.44% (-11.21 to -9.67)
Philippines	561 (357 to 874)	328 (199 to 517)	-41.61% (-69.88 to 6.08)	39.3 (25 to 61.2)	10.3 (6.2 to 16.2)	-4.27% (-4.44 to -4.1)
Republic of Moldova	9 (5 to 15)	1 (1 to 1)	-89.8% (-95.18 to -79.94)	7.3 (4.1 to 11.8)	0.7 (0.4 to 1.1)	-8.29% (-9.21 to -7.36)
Russian Federation	131 (81 to 197)	66 (36 to 109)	-49.66% (-74.11 to -5.4)	2.7 (1.7 to 4.1)	1.2 (0.7 to 2)	-2.93% (-3.36 to -2.49)
Rwanda	3212 (1920 to 5006)	1380 (704 to 2332)	-57.04% (-79.94 to -13.43)	2647.6 (1582.9 to 4126.1)	430 (219.5 to 726.5)	-6.9% (-7.8 to -5.99)
Saint Lucia	0 (0 to 0)	0 (0 to 0)	-38.76% (-71.42 to 17.82)	10.3 (6.3 to 15.7)	2.8 (1.5 to 4.8)	-3.28% (-4.32 to -2.23)
Saint Vincent and the Grenadines	0 (0 to 0)	0 (0 to 0)	28.86% (-41.65 to 162.77)	2.7 (1.5 to 4.4)	1.8 (0.9 to 3.2)	-0.54% (-1.31 to 0.24)
Samoa	1 (1 to 3)	0 (0 to 1)	-69.95% (-88.9 to -19.12)	43.7 (19.2 to 87.8)	7.9 (3.5 to 15)	-6.98% (-7.58 to -6.38)
Senegal	853 (452 to 1394)	747 (364 to 1325)	-12.52% (-60.62 to 87.8)	573.3 (303.7 to 936.2)	214.3 (104.4 to 380.3)	-2.98% (-3.48 to -2.48)

Location	DALYs in 1990 (95% UI)	DALYs in 2019 (95% UI)	Relative change (%, 95 CI)	Age-standardized DALYs rate in 1990 (95% UI)	Age-standardized DALYs rate in 2019 (95% UI)	EAPC (%, 95 CI)
Serbia	2 (1 to 4)	1 (0 to 1)	-56.9% (-81.07 to -15.1)	0.7 (0.4 to 1.3)	0.3 (0.2 to 0.5)	-3.29% (-3.79 to -2.78)
Sierra Leone	423 (218 to 743)	659 (309 to 1249)	55.97% (-30.77 to 253.87)	527.1 (271.9 to 926.5)	357.3 (167.6 to 677)	-1.18% (-1.92 to -0.43)
Solomon Islands	30 (12 to 62)	56 (27 to 107)	87.46% (-25.62 to 422.36)	498.1 (199.3 to 1022.6)	309.1 (146.6 to 590.5)	-1.18% (-1.77 to -0.58)
Somalia	2587 (1139 to 4851)	7185 (3473 to 12805)	177.69% (23.7 to 520.38)	1578.1 (694.7 to 2958.6)	1535.9 (742.4 to 2737.3)	0.09% (-0.13 to 0.31)
South Africa	557 (345 to 867)	211 (116 to 346)	-62.21% (-81.37 to -26.68)	63.3 (39.1 to 98.4)	12.1 (6.6 to 19.8)	-4.03% (-4.9 to -3.16)
South Sudan	353 (153 to 699)	480 (197 to 980)	35.98% (-43.35 to 211.82)	321.7 (139.1 to 636.9)	207.4 (85.1 to 423.6)	-1.6% (-1.78 to -1.41)
Sri Lanka	45 (24 to 78)	13 (6 to 24)	-71.58% (-89.46 to -23.95)	9 (4.8 to 15.5)	1.7 (0.7 to 3.1)	-5.33% (-5.56 to -5.11)
Sudan	5398 (3047 to 8631)	1976 (854 to 3853)	-63.38% (-84.93 to -14.71)	1328.6 (750 to 2124.4)	190.5 (82.3 to 371.4)	-6.54% (-7.04 to -6.04)
Suriname	1 (1 to 2)	1 (1 to 2)	21.52% (-51.85 to 208.84)	12.1 (6.1 to 21.4)	7.7 (3.8 to 13.3)	-1.38% (-1.73 to -1.04)
Syrian Arab Republic	84 (41 to 143)	27 (12 to 52)	-67.89% (-87.49 to -17.72)	38.9 (19.2 to 66.5)	5.3 (2.3 to 10.2)	-8.13% (-8.86 to -7.39)

Location	DALYs in 1990 (95% UI)	DALYs in 2019 (95% UI)	Relative change (%, 95 CI)	Age-standardized DALYs rate in 1990 (95% UI)	Age-standardized DALYs rate in 2019 (95% UI)	EAPC (%, 95 CI)
Tajikistan	32 (17 to 56)	18 (9 to 33)	-45.22% (-76.58 to 22.02)	41 (22.1 to 71)	7.3 (3.6 to 13.7)	-6.45% (-7.01 to -5.9)
Thailand	103 (49 to 194)	88 (46 to 160)	-14.88% (-63.93 to 115.27)	6.5 (3.1 to 12.2)	3.1 (1.6 to 5.6)	-1.08% (-1.72 to -0.43)
Timor-Leste	131 (57 to 233)	62 (10 to 115)	-52.63% (-91.78 to 19.68)	740.1 (323.6 to 1321.2)	211.7 (35.2 to 393.6)	-5.59% (-6.41 to -4.77)
Togo	422 (222 to 715)	454 (214 to 823)	7.55% (-51.99 to 137.24)	572.7 (301.3 to 970.8)	211.9 (99.9 to 384.4)	-2.98% (-3.39 to -2.57)
Tonga	2 (1 to 4)	1 (0 to 2)	-57.61% (-83.29 to 2.24)	100.4 (49.3 to 185)	31.8 (14.4 to 60.6)	-4.31% (-4.59 to -4.04)
Tunisia	60 (28 to 106)	14 (7 to 24)	-75.89% (-89.82 to -45.53)	32.7 (15.4 to 58.4)	3.2 (1.5 to 5.5)	-7.53% (-8.1 to -6.95)
Turkey	885 (416 to 1616)	102 (52 to 172)	-88.54% (-95.28 to -72.62)	62.4 (29.4 to 114)	3.2 (1.7 to 5.5)	-10.89% (-11.68 to -10.09)
Turkmenistan	8 (5 to 14)	7 (4 to 13)	-10.51% (-58.07 to 78.59)	14.6 (8 to 23.6)	4.9 (2.5 to 8.5)	-3.69% (-3.98 to -3.4)
Tuvalu	1 (0 to 1)	0 (0 to 0)	-83.07% (-93.54 to -55.42)	228.5 (103.2 to 447.6)	35.8 (15.5 to 71.1)	-6.1% (-6.24 to -5.95)
Uganda	702 (316 to 1267)	893 (461 to 1607)	27.22% (-45.79 to 212.59)	251.9 (113.5 to 454.7)	111.9 (57.7 to 201.2)	-2.89% (-3.42 to -2.35)

Location	DALYs in 1990 (95% UI)	DALYs in 2019 (95% UI)	Relative change (%, 95 CI)	Age-standardized DALYs rate in 1990 (95% UI)	Age-standardized DALYs rate in 2019 (95% UI)	EAPC (%, 95 CI)
Ukraine	16 (10 to 24)	13 (7 to 23)	-14.92% (-52.2 to 28.2)	1 (0.6 to 1.5)	0.8 (0.4 to 1.4)	-0.77% (-1.43 to -0.12)
United Republic of Tanzania	2117 (1140 to 3480)	2203 (1121 to 3844)	4.04% (-52.41 to 134.79)	461.9 (248.8 to 759.2)	165 (83.9 to 287.9)	-2.45% (-2.95 to -1.94)
Uzbekistan	82 (49 to 127)	42 (23 to 68)	-48.96% (-73.89 to -0.37)	29.1 (17.4 to 45.1)	4 (2.2 to 6.5)	-5.43% (-6.18 to -4.67)
Vanuatu	4 (2 to 8)	5 (2 to 11)	40.65% (-52 to 284.72)	126.6 (50.8 to 256.8)	72.4 (29.8 to 145.8)	-2.67% (-3.04 to -2.31)
Venezuela (Bolivarian Republic of)	359 (234 to 525)	141 (80 to 234)	-60.6% (-80.64 to -22.06)	77.2 (50.4 to 113)	14 (7.9 to 23.1)	-6.14% (-7 to -5.27)
Viet Nam	101 (47 to 199)	21 (11 to 37)	-79.45% (-91.05 to -51.18)	8.2 (3.8 to 16.2)	0.6 (0.3 to 1.1)	-9.26% (-9.75 to -8.76)
Yemen	1334 (572 to 2513)	1710 (786 to 3234)	28.15% (-50.36 to 254.35)	612.3 (262.7 to 1153.4)	222.4 (102.2 to 420.7)	-4.52% (-4.84 to -4.2)
Zambia	322 (163 to 560)	210 (100 to 389)	-34.73% (-74.06 to 46.18)	230.4 (116.6 to 401.4)	53.1 (25.4 to 98.4)	-6.09% (-6.77 to -5.42)
Zimbabwe	496 (273 to 810)	1092 (521 to 1947)	119.85% (1.49 to 362.97)	270.8 (148.8 to 442.1)	280.7 (133.9 to 500.8)	3.41% (1.88 to 4.97)

CI – confidence interval, DALYs – disability-adjusted life years (per 100,000 population), EAPC – estimated annual percentage change, MSMI – maternal sepsis and other maternal infections, UI – uncertain interval.

Table S32. DALYs of MSMI in 131 low- and middle-income countries and territories with EAPC in age group of 45-49 years from 1990 and 2019.

Location	DALYs in 1990 (95% UI)	DALYs in 2019 (95% UI)	Relative change (%, 95 CI)	Age-standardized DALYs rate in 1990 (95% UI)	Age-standardized DALYs rate in 2019 (95% UI)	EAPC (%, 95 CI)
Afghanistan	975 (377 to 2126)	1050 (427 to 2181)	7.71% (-64.39 to 228.44)	402.9 (155.7 to 879.1)	128.2 (52.2 to 266.3)	-4.88% (-6.18 to -3.57)
Albania	0 (0 to 1)	0 (0 to 0)	-28.03% (-71.78 to 74.49)	0.6 (0.3 to 1)	0.3 (0.1 to 0.5)	-0.52% (-1.74 to 0.7)
Algeria	152 (60 to 300)	28 (14 to 53)	-81.31% (-92.5 to -49.65)	39.4 (15.5 to 77.6)	2.3 (1.1 to 4.4)	-9.25% (-10.05 to -8.45)
American Samoa	0 (0 to 1)	0 (0 to 0)	-39.71% (-80.64 to 90.28)	46.2 (17.7 to 97.3)	15 (6.3 to 30.7)	-4.23% (-5.18 to -3.26)
Angola	355 (127 to 766)	406 (164 to 907)	14.32% (-61.27 to 290.79)	211.3 (75.3 to 456.2)	77.5 (31.3 to 173.2)	-3.51% (-3.78 to -3.23)
Armenia	0 (0 to 1)	1 (1 to 1)	132.88% (20.17 to 388.86)	0.7 (0.4 to 1.1)	1 (0.6 to 1.6)	2.96% (1.97 to 3.95)
Azerbaijan	2 (1 to 4)	1 (1 to 2)	-49.32% (-82.35 to 38.5)	2 (0.9 to 3.7)	0.4 (0.2 to 0.7)	-6.44% (-7.47 to -5.4)
Bangladesh	514 (232 to 1005)	80 (44 to 140)	-84.37% (-93.34 to -59.12)	32.9 (14.8 to 64.3)	1.8 (1 to 3.2)	-9.81% (-10.72 to -8.9)

Location	DALYs in 1990 (95% UI)	DALYs in 2019 (95% UI)	Relative change (%, 95 CI)	Age-standardized DALYs rate in 1990 (95% UI)	Age-standardized DALYs rate in 2019 (95% UI)	EAPC (%, 95 CI)
Belarus	0 (0 to 1)	0 (0 to 0)	-48.35% (-71.6 to -8.29)	0.1 (0.1 to 0.2)	0.1 (0 to 0.1)	-3.37% (-3.88 to -2.85)
Belize	0 (0 to 0)	0 (0 to 1)	150.67% (32.64 to 404.96)	6.1 (3.7 to 9.4)	3.6 (2.1 to 5.7)	-0.1% (-0.83 to 0.63)
Benin	406 (195 to 753)	317 (134 to 642)	-21.81% (-68.58 to 100.69)	549.1 (264.2 to 1019.5)	150.2 (63.2 to 303.8)	-3.68% (-4.16 to -3.19)
Bhutan	2 (1 to 5)	0 (0 to 1)	-80.42% (-92.29 to -46.09)	23 (9.2 to 51.3)	2.3 (1.2 to 4.1)	-7.73% (-7.97 to -7.48)
Bolivia (Plurinational State of)	197 (88 to 377)	126 (47 to 273)	-35.82% (-78.2 to 84.1)	165 (73.6 to 316.3)	43.6 (16.1 to 94.3)	-4.96% (-5.27 to -4.64)
Bosnia and Herzegovina	3 (2 to 7)	0 (0 to 0)	-98.34% (-99.26 to -95.57)	3 (1.3 to 5.7)	0 (0 to 0.1)	-16.46% (-18.15 to -14.72)
Botswana	5 (2 to 12)	2 (1 to 4)	-61.18% (-86.14 to 1.33)	26.3 (10.6 to 57.9)	3.7 (1.8 to 7.1)	-7.56% (-8.13 to -6.99)
Brazil	1091 (697 to 1626)	205 (130 to 298)	-81.18% (-89.64 to -65.05)	35 (22.3 to 52.1)	2.9 (1.8 to 4.2)	-7.45% (-8.49 to -6.4)
Bulgaria	10 (5 to 16)	2 (1 to 4)	-75.97% (-88.56 to -46.41)	3.6 (2 to 6.1)	0.9 (0.5 to 1.6)	-4.4% (-5.04 to -3.76)

Location	DALYs in 1990 (95% UI)	DALYs in 2019 (95% UI)	Relative change (%, 95 CI)	Age-standardized DALYs rate in 1990 (95% UI)	Age-standardized DALYs rate in 2019 (95% UI)	EAPC (%, 95 CI)
Burkina Faso	483 (213 to 879)	293 (122 to 588)	-39.19% (-78.73 to 53.04)	294.5 (129.7 to 536.5)	73 (30.3 to 146.4)	-5.97% (-6.99 to -4.95)
Burundi	495 (197 to 1007)	665 (295 to 1325)	34.38% (-50.73 to 282.01)	600.4 (239.2 to 1221.7)	383 (169.9 to 763.3)	-2.1% (-2.55 to -1.65)
Cabo Verde	1 (1 to 2)	0 (0 to 1)	-67.13% (-86.43 to -19.98)	22.8 (10.5 to 44.8)	2.8 (1.4 to 5)	-7.89% (-8.21 to -7.58)
Cambodia	481 (181 to 1032)	167 (63 to 354)	-65.38% (-88.95 to 8.16)	271.5 (101.9 to 581.7)	40.9 (15.5 to 87)	-7.34% (-8.31 to -6.37)
Cameroon	572 (270 to 1017)	514 (194 to 1109)	-10.15% (-69.66 to 138.06)	337.1 (159.2 to 599.7)	103.8 (39.1 to 224.2)	-3.72% (-4.15 to -3.28)
Central African Republic	195 (79 to 383)	462 (168 to 1039)	136.77% (-19.38 to 570.75)	398.5 (160.8 to 782.2)	466.6 (170.2 to 1050.7)	0.67% (0.39 to 0.95)
Chad	568 (246 to 1092)	727 (295 to 1487)	28.12% (-52.33 to 249.16)	583.5 (252.4 to 1122.7)	311 (126.3 to 636.1)	-2.11% (-2.33 to -1.89)
China	843 (623 to 1125)	164 (89 to 275)	-80.57% (-89.71 to -66.67)	3.5 (2.6 to 4.6)	0.3 (0.1 to 0.5)	-7.88% (-8.89 to -6.86)

Location	DALYs in 1990 (95% UI)	DALYs in 2019 (95% UI)	Relative change (%, 95 CI)	Age-standardized DALYs rate in 1990 (95% UI)	Age-standardized DALYs rate in 2019 (95% UI)	EAPC (%, 95 CI)
Colombia	93 (55 to 146)	64 (34 to 113)	-30.54% (-67.14 to 54.97)	14.7 (8.7 to 23.2)	4.4 (2.3 to 7.6)	-3.7% (-4.47 to -2.93)
Comoros	13 (4 to 28)	10 (4 to 22)	-22.49% (-74.59 to 209.87)	165.2 (48.9 to 370.3)	55.4 (21.6 to 123.4)	-4.14% (-4.47 to -3.8)
Congo	83 (32 to 181)	97 (36 to 202)	17.22% (-67.39 to 246.08)	215.9 (84.2 to 471.9)	80.3 (29.8 to 167.8)	-2.95% (-3.67 to -2.23)
Costa Rica	1 (1 to 2)	1 (1 to 2)	-2.65% (-52.21 to 106.63)	2.2 (1.2 to 3.7)	0.8 (0.5 to 1.3)	-2.66% (-3.65 to -1.66)
Côte d'Ivoire	418 (189 to 778)	409 (159 to 829)	-2.09% (-65.89 to 144.75)	243.7 (110.3 to 453.6)	93.3 (36.3 to 189.1)	-3.02% (-3.4 to -2.65)
Cuba	3 (2 to 6)	3 (2 to 4)	-17.86% (-60.94 to 83.56)	1.1 (0.6 to 1.9)	0.6 (0.3 to 0.9)	-2.08% (-2.45 to -1.7)
Democratic People's Republic of Korea	38 (13 to 88)	46 (16 to 103)	21.84% (-63.95 to 340)	5.8 (1.9 to 13.5)	4.5 (1.6 to 10.2)	-0.36% (-1.16 to 0.44)
Democratic Republic of the Congo	2988 (1218 to 5826)	9750 (3853 to 19397)	226.27% (9.18 to 798.38)	484 (197.2 to 943.6)	653.8 (258.3 to 1300.7)	2.77% (1.53 to 4.03)

Location	DALYs in 1990 (95% UI)	DALYs in 2019 (95% UI)	Relative change (%, 95 CI)	Age-standardized DALYs rate in 1990 (95% UI)	Age-standardized DALYs rate in 2019 (95% UI)	EAPC (%, 95 CI)
Djibouti	14 (6 to 30)	26 (10 to 56)	83.21% (-39.83 to 459.94)	210.5 (85.2 to 454)	92.9 (34.3 to 203.9)	-2.41% (-2.77 to -2.06)
Dominica	0 (0 to 0)	0 (0 to 0)	4.57% (-61.93 to 176.51)	9.7 (4.4 to 17.7)	7 (3 to 13.5)	0.84% (-0.01 to 1.68)
Dominican Republic	8 (4 to 14)	6 (3 to 13)	-15.14% (-68.15 to 134)	6 (3.1 to 11.3)	2.2 (0.9 to 4.4)	-3.04% (-3.98 to -2.09)
Ecuador	31 (17 to 54)	24 (12 to 43)	-24.35% (-69.15 to 78.75)	17.1 (9 to 29.2)	5 (2.4 to 9.1)	-3.23% (-4.33 to -2.12)
Egypt	367 (179 to 675)	29 (14 to 59)	-92.01% (-96.86 to -78.09)	33.1 (16.1 to 60.8)	1.3 (0.6 to 2.6)	-11.97% (-12.78 to -11.15)
El Salvador	31 (13 to 63)	3 (1 to 6)	-91.16% (-97.03 to -74.09)	31.1 (13.4 to 63.2)	1.4 (0.6 to 3)	-9.87% (-11.41 to -8.31)
Equatorial Guinea	61 (23 to 128)	5 (2 to 13)	-91.07% (-97.18 to -69.6)	733.6 (270.2 to 1541.3)	23.6 (9 to 54.2)	-12.51% (-13.2 to -11.81)
Eritrea	225 (85 to 482)	223 (86 to 490)	-0.97% (-69.77 to 204.27)	437.2 (165.2 to 933.9)	177.2 (68.5 to 389.1)	-2.01% (-2.54 to -1.47)

Location	DALYs in 1990 (95% UI)	DALYs in 2019 (95% UI)	Relative change (%, 95 CI)	Age-standardized DALYs rate in 1990 (95% UI)	Age-standardized DALYs rate in 2019 (95% UI)	EAPC (%, 95 CI)
Eswatini	13 (5 to 28)	6 (2 to 13)	-56.79% (-87.44 to 50.89)	104 (39 to 220.9)	26.3 (9 to 60.1)	-3.67% (-4.79 to -2.54)
Ethiopia	12181 (6410 to 20360)	3841 (2024 to 6635)	-68.47% (-84.39 to -34.88)	1549.4 (815.3 to 2589.8)	231.2 (121.8 to 399.3)	-6.93% (-7.67 to -6.18)
Fiji	5 (2 to 10)	3 (1 to 6)	-43.95% (-81.67 to 81.49)	29.9 (11.7 to 62.9)	11.2 (4.4 to 23.6)	-3.61% (-3.92 to -3.3)
Gabon	24 (11 to 46)	12 (5 to 27)	-48.44% (-81.76 to 42.27)	146.6 (65.7 to 282)	28.7 (11.4 to 62)	-5.93% (-6.66 to -5.2)
Gambia	67 (30 to 121)	119 (51 to 235)	75.81% (-34.27 to 359.6)	538.6 (235.7 to 962.7)	324.2 (138.2 to 642.3)	-1.99% (-2.28 to -1.71)
Georgia	1 (0 to 1)	1 (0 to 1)	56.12% (-27.37 to 226.46)	0.4 (0.2 to 0.6)	0.7 (0.4 to 1.1)	4.94% (3.4 to 6.5)
Ghana	186 (81 to 382)	54 (26 to 98)	-71.2% (-88.5 to -26.75)	76.2 (33.3 to 156.4)	7.7 (3.7 to 14)	-8.1% (-9.22 to -6.98)
Global	60692 (50182 to 73386)	42877 (32999 to 56156)	-29.35% (-47.43 to -6.46)	53.3 (44.1 to 64.4)	18.2 (14 to 23.9)	-3.42% (-3.69 to -3.16)
Grenada	0 (0 to 0)	0 (0 to 0)	-44.53% (-71.6 to 9.85)	1.4 (0.9 to 2.2)	0.3 (0.2 to 0.5)	-3.84% (-4.59 to -3.08)

Location	DALYs in 1990 (95% UI)	DALYs in 2019 (95% UI)	Relative change (%, 95 CI)	Age-standardized DALYs rate in 1990 (95% UI)	Age-standardized DALYs rate in 2019 (95% UI)	EAPC (%, 95 CI)
Guatemala	47 (25 to 81)	27 (15 to 47)	-42.33% (-72 to 18.88)	37.1 (19.5 to 63.4)	6.8 (3.6 to 11.8)	-7.23% (-8.38 to -6.07)
Guinea	688 (307 to 1274)	574 (238 to 1212)	-16.57% (-69.07 to 119.88)	635.5 (284 to 1176.9)	269.6 (111.7 to 569.2)	-2.38% (-2.59 to -2.17)
Guinea-Bissau	68 (27 to 136)	39 (15 to 78)	-43.3% (-81.03 to 58.31)	419.2 (166 to 833.1)	119.5 (45 to 242.2)	-3.96% (-4.34 to -3.59)
Guyana	1 (1 to 2)	1 (0 to 1)	-41.08% (-74.9 to 30.69)	7.4 (4 to 13)	2.7 (1.4 to 4.8)	-2.21% (-2.78 to -1.63)
Haiti	203 (82 to 416)	517 (190 to 1189)	155.12% (-16.25 to 638.17)	177.9 (72 to 365.6)	182 (67 to 418.7)	1.09% (0.54 to 1.64)
Honduras	37 (17 to 70)	16 (6 to 34)	-58.13% (-85.83 to 14.02)	50.9 (23.7 to 95.1)	6.9 (2.6 to 14.9)	-7.7% (-8.29 to -7.1)
India	10825 (6287 to 17210)	2188 (1303 to 3489)	-79.79% (-89.64 to -62.26)	64.3 (37.4 to 102.3)	5.9 (3.5 to 9.4)	-7.98% (-8.31 to -7.65)
Indonesia	1538 (869 to 2678)	222 (132 to 354)	-85.55% (-92.87 to -69.65)	39.9 (22.6 to 69.5)	2.6 (1.5 to 4.1)	-9.47% (-9.95 to -8.98)
Iran (Islamic Republic of)	156 (104 to 225)	16 (10 to 24)	-89.63% (-94.2 to -82.03)	19.2 (12.8 to 27.7)	0.6 (0.4 to 0.9)	-12.04% (-12.89 to -11.18)

Location	DALYs in 1990 (95% UI)	DALYs in 2019 (95% UI)	Relative change (%, 95 CI)	Age-standardized DALYs rate in 1990 (95% UI)	Age-standardized DALYs rate in 2019 (95% UI)	EAPC (%, 95 CI)
Iraq	30 (13 to 61)	20 (10 to 36)	-34.63% (-75.22 to 81.69)	11.3 (4.8 to 23.1)	1.9 (0.9 to 3.6)	-5.85% (-6.59 to -5.1)
Jamaica	2 (1 to 4)	1 (0 to 1)	-68.54% (-86.08 to -32.89)	5.5 (3.3 to 9)	0.9 (0.5 to 1.4)	-5.2% (-7.3 to -3.04)
Jordan	16 (6 to 32)	6 (2 to 11)	-64.75% (-87.61 to -0.3)	29.4 (11.6 to 59.9)	2 (0.9 to 4)	-10.26% (-10.99 to -9.53)
Kazakhstan	1 (1 to 2)	5 (3 to 9)	406.83% (133.63 to 1005.53)	0.3 (0.2 to 0.6)	0.9 (0.5 to 1.5)	4.5% (4.03 to 4.97)
Kenya	566 (311 to 895)	784 (406 to 1297)	38.39% (-27.37 to 154.89)	182.1 (100.2 to 288)	81.9 (42.4 to 135.6)	-2.35% (-3.07 to -1.62)
Kiribati	3 (1 to 5)	2 (1 to 5)	-14.96% (-72.27 to 174.14)	191.8 (78.7 to 383.3)	84.3 (30.4 to 179.5)	-3.16% (-3.38 to -2.94)
Kyrgyzstan	1 (0 to 1)	2 (1 to 3)	274.94% (111.28 to 608.73)	1 (0.6 to 1.5)	1.3 (0.8 to 2)	3.13% (2.15 to 4.13)
Lao People's Democratic Republic	55 (23 to 107)	13 (5 to 26)	-76.96% (-92.54 to -32.46)	72.5 (30.7 to 141.8)	7.3 (3 to 15.3)	-8.54% (-9.3 to -7.77)

Location	DALYs in 1990 (95% UI)	DALYs in 2019 (95% UI)	Relative change (%, 95 CI)	Age-standardized DALYs rate in 1990 (95% UI)	Age-standardized DALYs rate in 2019 (95% UI)	EAPC (%, 95 CI)
Lebanon	4 (2 to 8)	1 (0 to 2)	-76.69% (-91.07 to -38.91)	5.1 (2.2 to 10.4)	0.5 (0.3 to 1)	-8.09% (-8.55 to -7.64)
Lesotho	99 (42 to 188)	57 (21 to 126)	-42.52% (-81.28 to 74.89)	328.6 (139.2 to 619.8)	135.8 (49.3 to 299.1)	-1.12% (-2.04 to -0.2)
Liberia	319 (156 to 568)	535 (237 to 1054)	67.58% (-35.41 to 328.33)	983.4 (480.6 to 1748.8)	519.8 (230.6 to 1024.5)	-3.4% (-4.36 to -2.43)
Libya	6 (3 to 12)	4 (2 to 8)	-33.96% (-76.76 to 83.55)	10.1 (4.6 to 20.9)	1.6 (0.7 to 3.4)	-5.92% (-6.71 to -5.12)
Madagascar	500 (245 to 909)	1054 (398 to 2282)	110.85% (-29.32 to 420.07)	283 (138.6 to 514.7)	202.8 (76.6 to 439.3)	-1.34% (-1.9 to -0.78)
Malawi	986 (498 to 1749)	644 (295 to 1289)	-34.63% (-75.11 to 60.4)	684.7 (346.2 to 1214.9)	219.9 (100.6 to 439.9)	-3.71% (-4.31 to -3.11)
Malaysia	134 (56 to 287)	37 (17 to 78)	-71.97% (-90.21 to -9.67)	41.2 (17.2 to 88.6)	4.6 (2 to 9.5)	-7.52% (-7.98 to -7.07)
Maldives	3 (1 to 6)	0 (0 to 0)	-92.13% (-97.18 to -76.43)	88.8 (33.3 to 189.7)	2.1 (1 to 4.1)	-12.75% (-13.72 to -11.77)
Mali	2243 (1232 to 3866)	1235 (516 to 2369)	-44.95% (-79.18 to 33.68)	1532.1 (841.3 to 2640)	382.1 (159.8 to 732.9)	-5.55% (-6.11 to -5)

Location	DALYs in 1990 (95% UI)	DALYs in 2019 (95% UI)	Relative change (%, 95 CI)	Age-standardized DALYs rate in 1990 (95% UI)	Age-standardized DALYs rate in 2019 (95% UI)	EAPC (%, 95 CI)
Marshall Islands	1<br>(0 to 1)	1<br>(0 to 2)	20.21%<br>(-64.8 to 285.67)	108.2<br>(43.2 to 232.4)	47.7<br>(17.1 to 107.2)	-3.14%<br>(-3.64 to -2.65)
Mauritania	520<br>(246 to 911)	143<br>(60 to 278)	-72.4%<br>(-89.73 to -31.56)	1588.8<br>(751.4 to 2785.7)	183.8<br>(77.3 to 356)	-7.71%<br>(-7.94 to -7.47)
Mexico	106<br>(68 to 161)	121<br>(80 to 178)	13.75%<br>(-36.44 to 105.07)	6.8<br>(4.4 to 10.3)	3<br>(2 to 4.4)	-1.71%<br>(-2.84 to -0.57)
Micronesia (Federated States of)	3<br>(1 to 5)	1<br>(0 to 3)	-55.09%<br>(-94.22 to 70.83)	171.6<br>(64.2 to 366.3)	41.8<br>(5.3 to 98.3)	-4.79%<br>(-4.92 to -4.66)
Mongolia	6<br>(2 to 11)	3<br>(1 to 7)	-42.73%<br>(-82.14 to 86.52)	16.5<br>(6.4 to 33.4)	3<br>(1.1 to 6.5)	-6.47%<br>(-6.77 to -6.17)
Montenegro	0<br>(0 to 0)	0<br>(0 to 0)	1.97%<br>(-32.39 to 36.07)	0.1<br>(0 to 0.1)	0.1<br>(0 to 0.1)	-0.91%<br>(-1.03 to -0.79)
Morocco	332<br>(162 to 615)	35<br>(15 to 69)	-89.59%<br>(-96.32 to -73.42)	68.5<br>(33.4 to 127.1)	3.2<br>(1.4 to 6.4)	-10.05%<br>(-10.54 to -9.55)
Mozambique	538<br>(265 to 986)	250<br>(100 to 511)	-53.55%<br>(-84.82 to 18.63)	220.3<br>(108.5 to 403.7)	50<br>(20.1 to 102.2)	-4.85%<br>(-5.5 to -4.2)
Myanmar	118<br>(55 to 224)	152<br>(65 to 308)	28.93%<br>(-51.33 to 223.46)	15.5<br>(7.2 to 29.5)	8.8<br>(3.8 to 17.8)	-2.42%<br>(-2.71 to -2.13)

Location	DALYs in 1990 (95% UI)	DALYs in 2019 (95% UI)	Relative change (%, 95 CI)	Age-standardized DALYs rate in 1990 (95% UI)	Age-standardized DALYs rate in 2019 (95% UI)	EAPC (%, 95 CI)
Namibia	48 (21 to 87)	19 (8 to 41)	-59.97% (-85.68 to 18.31)	206.5 (92.8 to 377.4)	34.8 (13.9 to 75)	-6.12% (-6.86 to -5.38)
Nepal	461 (187 to 1016)	111 (43 to 238)	-75.89% (-92.14 to -31.39)	128.6 (52.2 to 283.4)	14.4 (5.5 to 30.8)	-6.07% (-6.86 to -5.28)
Nicaragua	4 (2 to 7)	2 (1 to 4)	-44.85% (-79.47 to 46.74)	6.4 (3.2 to 11.7)	1.2 (0.6 to 2.2)	-6.03% (-6.39 to -5.66)
Niger	1012 (452 to 1936)	1552 (603 to 3052)	53.39% (-40.04 to 280.45)	835.5 (373.3 to 1598.9)	490.7 (190.7 to 964.9)	-2.07% (-2.27 to -1.87)
Nigeria	590 (304 to 1060)	953 (528 to 1671)	61.56% (-16.94 to 227.66)	44 (22.7 to 79.1)	23.5 (13 to 41.2)	-2.49% (-2.84 to -2.14)
North Macedonia	0 (0 to 0)	0 (0 to 0)	-69.92% (-86.65 to -33.81)	0.3 (0.2 to 0.6)	0.1 (0 to 0.1)	-6.47% (-7.03 to -5.9)
Pakistan	2499 (1313 to 4575)	1297 (634 to 2510)	-48.11% (-79.37 to 23.47)	124.7 (65.5 to 228.3)	28 (13.7 to 54.3)	-5.4% (-6.14 to -4.66)
Papua New Guinea	116 (44 to 246)	121 (45 to 256)	4.79% (-66.48 to 218.66)	164.5 (62.6 to 350.5)	55.7 (20.7 to 117.8)	-3.68% (-4 to -3.36)
Paraguay	19 (10 to 33)	6 (3 to 13)	-65.73% (-86.56 to -13.58)	25.5 (12.9 to 44.8)	3.8 (1.7 to 7.6)	-6.78% (-7.21 to -6.36)

Location	DALYs in 1990 (95% UI)	DALYs in 2019 (95% UI)	Relative change (%, 95 CI)	Age-standardized DALYs rate in 1990 (95% UI)	Age-standardized DALYs rate in 2019 (95% UI)	EAPC (%, 95 CI)
Peru	346 (160 to 620)	65 (25 to 139)	-81.25% (-93.98 to -50.53)	84.7 (39.3 to 152)	6.4 (2.4 to 13.8)	-9.42% (-9.91 to -8.92)
Philippines	191 (115 to 293)	115 (65 to 188)	-40.02% (-69.71 to 19.74)	16.6 (10 to 25.5)	4.1 (2.3 to 6.7)	-4.71% (-4.85 to -4.57)
Republic of Moldova	1 (1 to 2)	0 (0 to 0)	-93% (-96.5 to -85.08)	1 (0.5 to 1.7)	0.1 (0 to 0.1)	-9.91% (-11.04 to -8.77)
Russian Federation	9 (5 to 13)	6 (4 to 10)	-28.04% (-58.81 to 17.05)	0.2 (0.2 to 0.4)	0.1 (0.1 to 0.2)	-3.86% (-4.45 to -3.26)
Rwanda	2397 (1271 to 4092)	1042 (449 to 1978)	-56.53% (-84.12 to 4.48)	2362.2 (1252.7 to 4032)	424.5 (182.7 to 805.6)	-6.36% (-7.18 to -5.53)
Saint Lucia	0 (0 to 0)	0 (0 to 0)	-34.15% (-67.62 to 31.53)	2.5 (1.4 to 3.8)	0.6 (0.3 to 0.9)	-3.33% (-4.53 to -2.12)
Saint Vincent and the Grenadines	0 (0 to 0)	0 (0 to 0)	28.16% (-24.63 to 123.11)	0.7 (0.4 to 1.1)	0.4 (0.2 to 0.6)	-1.28% (-1.93 to -0.63)
Samoa	0 (0 to 1)	0 (0 to 0)	-56.05% (-84.85 to 38.1)	13 (4.9 to 28.2)	3.1 (1.4 to 6.3)	-6.1% (-6.76 to -5.44)
Senegal	430 (189 to 798)	400 (176 to 808)	-6.92% (-64.64 to 141.68)	374 (164.8 to 695)	140.7 (61.8 to 284.3)	-3.05% (-3.49 to -2.6)

Location	DALYs in 1990 (95% UI)	DALYs in 2019 (95% UI)	Relative change (%, 95 CI)	Age-standardized DALYs rate in 1990 (95% UI)	Age-standardized DALYs rate in 2019 (95% UI)	EAPC (%, 95 CI)
Serbia	0 (0 to 1)	0 (0 to 0)	-31.99% (-65.21 to 33.24)	0.1 (0.1 to 0.2)	0.1 (0.1 to 0.2)	-1.2% (-1.72 to -0.69)
Sierra Leone	213 (94 to 417)	283 (117 to 571)	33.32% (-46.94 to 254.96)	349.9 (154.7 to 686.5)	204.7 (84.5 to 412.5)	-1.79% (-2.49 to -1.09)
Solomon Islands	14 (5 to 33)	27 (9 to 61)	96.85% (-41.36 to 618.11)	274.9 (98.6 to 651.9)	185 (63.6 to 413.7)	-0.97% (-1.53 to -0.41)
Somalia	1267 (477 to 2676)	3251 (1149 to 6774)	156.57% (-15.4 to 616.14)	1000.5 (377 to 2112.8)	988.3 (349.2 to 2059.3)	0.16% (-0.08 to 0.39)
South Africa	387 (228 to 614)	93 (50 to 158)	-75.89% (-88.97 to -51.87)	53.3 (31.5 to 84.5)	6 (3.2 to 10.1)	-5.68% (-7.25 to -4.09)
South Sudan	156 (58 to 333)	237 (81 to 528)	51.93% (-51.36 to 346.06)	202.7 (75.6 to 433)	128.8 (44.2 to 287.3)	-1.65% (-1.81 to -1.49)
Sri Lanka	10 (5 to 19)	3 (1 to 6)	-66.85% (-87.29 to -12.97)	2.5 (1.2 to 4.9)	0.5 (0.2 to 0.8)	-5.28% (-5.62 to -4.94)
Sudan	1889 (920 to 3535)	1122 (457 to 2401)	-40.59% (-79.15 to 62.47)	557.5 (271.5 to 1043.1)	141.5 (57.6 to 302.7)	-4.11% (-5.29 to -2.9)

Location	DALYs in 1990 (95% UI)	DALYs in 2019 (95% UI)	Relative change (%, 95 CI)	Age-standardized DALYs rate in 1990 (95% UI)	Age-standardized DALYs rate in 2019 (95% UI)	EAPC (%, 95 CI)
Suriname	0 (0 to 0)	0 (0 to 1)	30.62% (-57.57 to 274.31)	3 (1.4 to 5.7)	1.8 (0.7 to 3.6)	-1.51% (-1.75 to -1.26)
Syrian Arab Republic	18 (8 to 37)	10 (4 to 21)	-44.06% (-80.74 to 58.09)	10.1 (4.6 to 20.2)	2.2 (0.9 to 4.5)	-5.97% (-6.61 to -5.32)
Tajikistan	2 (1 to 3)	1 (1 to 2)	-35.39% (-68.89 to 42.04)	2.7 (1.3 to 4.7)	0.5 (0.3 to 0.9)	-6.42% (-6.83 to -6.01)
Thailand	58 (23 to 130)	95 (40 to 192)	62.66% (-47.44 to 417.8)	4.4 (1.8 to 9.8)	3.2 (1.3 to 6.5)	1.4% (0.5 to 2.3)
Timor-Leste	65 (26 to 128)	35 (12 to 73)	-45.93% (-84.83 to 60.98)	467.5 (186.6 to 916.2)	133.1 (45.8 to 275.2)	-5.51% (-6.38 to -4.62)
Togo	206 (95 to 377)	220 (81 to 459)	6.89% (-63.36 to 196.05)	362.2 (166.8 to 664.4)	127.9 (47.1 to 267)	-3.34% (-3.73 to -2.95)
Tonga	1 (1 to 3)	1 (0 to 1)	-61.66% (-88.91 to 22.04)	76 (32.3 to 163)	21.7 (8.2 to 48.9)	-4.61% (-4.86 to -4.36)
Tunisia	9 (4 to 20)	3 (1 to 6)	-69.51% (-89.08 to -18.57)	6.2 (2.7 to 12.9)	0.8 (0.4 to 1.6)	-6.83% (-7.4 to -6.25)
Turkey	208 (83 to 456)	30 (15 to 60)	-85.45% (-95.21 to -56.37)	17.9 (7.1 to 39.3)	1.2 (0.6 to 2.3)	-9.98% (-10.58 to -9.37)

Location	DALYs in 1990 (95% UI)	DALYs in 2019 (95% UI)	Relative change (%, 95 CI)	Age-standardized DALYs rate in 1990 (95% UI)	Age-standardized DALYs rate in 2019 (95% UI)	EAPC (%, 95 CI)
Turkmenistan	1 (0 to 1)	1 (0 to 1)	30.23% (-29.62 to 156.4)	1.1 (0.7 to 1.8)	0.6 (0.3 to 1)	-2.78% (-3.15 to -2.41)
Tuvalu	0 (0 to 1)	0 (0 to 0)	-78.9% (-93.32 to -26.88)	137.4 (48.8 to 299.1)	24.7 (8.5 to 55)	-5.69% (-5.81 to -5.57)
Uganda	450 (191 to 849)	521 (232 to 1018)	15.86% (-55.07 to 217.43)	187.8 (79.8 to 354.6)	82.9 (37 to 162)	-2.54% (-3.03 to -2.05)
Ukraine	1 (1 to 2)	1 (1 to 2)	32.42% (-17.06 to 89.36)	0.1 (0 to 0.1)	0.1 (0 to 0.1)	0.43% (-0.24 to 1.11)
United Republic of Tanzania	1855 (942 to 3274)	1397 (633 to 2754)	-24.69% (-71.23 to 90.85)	472.4 (239.9 to 833.8)	131.3 (59.5 to 259)	-3.18% (-3.72 to -2.64)
Uzbekistan	8 (5 to 13)	16 (9 to 26)	100.07% (-3.85 to 304.43)	2.6 (1.5 to 4.2)	1.8 (1 to 2.9)	-0.03% (-0.75 to 0.69)
Vanuatu	2 (1 to 4)	3 (1 to 6)	43.31% (-55.5 to 413.64)	78.4 (26.9 to 173.3)	45.6 (17 to 104.6)	-2.66% (-3 to -2.32)
Venezuela (Bolivarian Republic of)	76 (47 to 115)	45 (25 to 73)	-41.05% (-70.53 to 21.96)	22.7 (13.9 to 34.3)	5.1 (2.9 to 8.2)	-5.24% (-5.89 to -4.58)
Viet Nam	22 (9 to 48)	5 (2 to 11)	-75.77% (-93 to -29.12)	2 (0.8 to 4.4)	0.2 (0.1 to 0.3)	-8.72% (-9.14 to -8.31)

Location	DALYs in 1990 (95% UI)	DALYs in 2019 (95% UI)	Relative change (%, 95 CI)	Age-standardized DALYs rate in 1990 (95% UI)	Age-standardized DALYs rate in 2019 (95% UI)	EAPC (%, 95 CI)
Yemen	296 (108 to 645)	274 (99 to 592)	-7.55% (-70.47 to 196.88)	162 (59.2 to 352.8)	51.4 (18.6 to 110.9)	-5.14% (-5.55 to -4.72)
Zambia	189 (90 to 343)	91 (38 to 183)	-51.72% (-83.03 to 27.6)	159.3 (76.1 to 289.6)	31.3 (13.1 to 62.8)	-6.93% (-7.71 to -6.15)
Zimbabwe	295 (148 to 503)	459 (182 to 906)	55.55% (-43 to 278.79)	200.7 (100.2 to 341.8)	160.5 (63.5 to 316.5)	2.08% (0.66 to 3.53)

CI – confidence interval, DALYs – disability-adjusted life years (per 100,000 population), EAPC – estimated annual percentage change, MSMI – maternal sepsis and other maternal infections, UI – uncertain interval.

Table S33. DALYs of MSMI in 131 low- and middle-income countries and territories with EAPC in age group of 50-54 years from 1990 and 2019.

Location	DALYs in 1990 (95% UI)	DALYs in 2019 (95% UI)	Relative change (%, 95 CI)	Age-standardized DALYs rate in 1990 (95% UI)	Age-standardized DALYs rate in 2019 (95% UI)	EAPC (%, 95 CI)
Afghanistan	71 (24 to 177)	63 (20 to 147)	-10.93% (-77.13 to 207.13)	32.7 (11.1 to 81.5)	11.1 (3.6 to 25.7)	-4.56% (-5.65 to -3.46)
Albania	0 (0 to 0)	0 (0 to 1)	253.31% (27.11 to 790.76)	0.1 (0.1 to 0.2)	0.3 (0.1 to 0.6)	10.77% (5.06 to 16.79)
Algeria	12 (4 to 28)	4 (2 to 8)	-68.71% (-89.98 to 6.01)	3.4 (1.2 to 7.7)	0.4 (0.2 to 0.7)	-7.11% (-7.78 to -6.43)
American Samoa	0 (0 to 0)	0 (0 to 0)	-33.36% (-76.6 to 101.05)	4.5 (1.8 to 10.2)	1.3 (0.6 to 2.5)	-5.73% (-6.52 to -4.93)
Angola	14 (5 to 31)	21 (10 to 43)	51.18% (-45.8 to 332.99)	10.2 (3.8 to 23)	4.8 (2.2 to 9.9)	-2.51% (-2.81 to -2.22)
Armenia	0 (0 to 0)	0 (0 to 0)	-72.22% (-86.05 to -51.44)	0 (0 to 0)	0 (0 to 0)	-4.68% (-5.28 to -4.08)
Azerbaijan	0 (0 to 0)	0 (0 to 0)	-48.62% (-77.65 to 13.52)	0.1 (0 to 0.1)	0 (0 to 0)	-4.98% (-5.54 to -4.41)
Bangladesh	933 (429 to 1737)	91 (40 to 180)	-90.2% (-96.29 to -70.67)	76.3 (35.1 to 142.1)	2.5 (1.1 to 5)	-8.65% (-10.82 to -6.44)

Location	DALYs in 1990 (95% UI)	DALYs in 2019 (95% UI)	Relative change (%, 95 CI)	Age-standardized DALYs rate in 1990 (95% UI)	Age-standardized DALYs rate in 2019 (95% UI)	EAPC (%, 95 CI)
Belarus	0<br>(0 to 0)	0<br>(0 to 0)	-44.57%<br>(-67.2 to -20.53)	0<br>(0 to 0)	0<br>(0 to 0)	-2.09%<br>(-2.3 to -1.88)
Belize	0<br>(0 to 0)	0<br>(0 to 0)	64.1%<br>(-29.59 to 225.94)	0.5<br>(0.3 to 0.8)	0.2<br>(0.1 to 0.4)	-2.82%<br>(-3.26 to -2.37)
Benin	15<br>(5 to 38)	13<br>(5 to 28)	-15.27%<br>(-77.05 to 196.32)	27.2<br>(9.1 to 68.5)	8.1<br>(3 to 17.9)	-3.27%<br>(-3.79 to -2.74)
Bhutan	1<br>(0 to 2)	0<br>(0 to 0)	-73.71%<br>(-91.53 to -15.62)	11.2<br>(3.6 to 28.1)	1.5<br>(0.7 to 3.1)	-6.82%<br>(-7.01 to -6.63)
Bolivia (Plurinational State of)	3<br>(1 to 7)	3<br>(1 to 6)	-15.35%<br>(-77.42 to 234.73)	3.2<br>(1 to 7.7)	1<br>(0.4 to 2.4)	-4.52%<br>(-4.79 to -4.24)
Bosnia and Herzegovina	0<br>(0 to 0)	0<br>(0 to 0)	-86.55%<br>(-94.89 to -67.9)	0.2<br>(0.1 to 0.3)	0<br>(0 to 0.1)	-8.05%<br>(-9.36 to -6.72)
Botswana	0<br>(0 to 1)	0<br>(0 to 1)	9.17%<br>(-52.49 to 119.56)	2.2<br>(1 to 4.3)	0.9<br>(0.4 to 1.8)	-3.11%<br>(-3.29 to -2.93)
Brazil	25<br>(17 to 37)	22<br>(12 to 39)	-13.51%<br>(-48.21 to 33.24)	0.9<br>(0.6 to 1.4)	0.3<br>(0.2 to 0.6)	-3.16%<br>(-3.65 to -2.66)

Location	DALYs in 1990 (95% UI)	DALYs in 2019 (95% UI)	Relative change (%, 95 CI)	Age-standardized DALYs rate in 1990 (95% UI)	Age-standardized DALYs rate in 2019 (95% UI)	EAPC (%, 95 CI)
Bulgaria	2 (2 to 3)	0 (0 to 0)	-90.01% (-94.19 to -84.41)	0.9 (0.7 to 1.1)	0.1 (0.1 to 0.2)	-8.37% (-9.53 to -7.2)
Burkina Faso	12 (5 to 26)	11 (5 to 21)	-9.48% (-65.56 to 166.34)	9.1 (3.6 to 19.5)	3.5 (1.7 to 6.7)	-3.93% (-4.27 to -3.59)
Burundi	42 (13 to 105)	49 (16 to 117)	17.19% (-67.93 to 352.61)	59.1 (18.8 to 147.2)	34.7 (11.1 to 81.8)	-2.67% (-3.17 to -2.16)
Cabo Verde	0 (0 to 0)	0 (0 to 0)	19.64% (-46.36 to 100.83)	1.7 (0.8 to 3.3)	1 (0.4 to 2.1)	-2.24% (-2.35 to -2.13)
Cambodia	11 (3 to 27)	6 (2 to 13)	-47.53% (-85.81 to 120.7)	6.7 (2 to 17.1)	1.4 (0.5 to 3.2)	-6.29% (-7.07 to -5.5)
Cameroon	23 (8 to 52)	21 (8 to 47)	-7.43% (-71.23 to 204.43)	15.8 (5.5 to 35.8)	5.5 (2.2 to 12.1)	-3.4% (-3.79 to -3.01)
Central African Republic	8 (3 to 21)	17 (6 to 41)	114.64% (-46.6 to 653.05)	19.4 (6.3 to 49.8)	21.3 (7.2 to 50)	0.44% (0.2 to 0.68)
Chad	25 (8 to 60)	29 (11 to 68)	19.19% (-64.68 to 330.05)	31.3 (10.3 to 77.2)	17.1 (6.4 to 40.1)	-1.94% (-2.24 to -1.64)

Location	DALYs in 1990 (95% UI)	DALYs in 2019 (95% UI)	Relative change (%, 95 CI)	Age-standardized DALYs rate in 1990 (95% UI)	Age-standardized DALYs rate in 2019 (95% UI)	EAPC (%, 95 CI)
China	260 (184 to 371)	31 (19 to 50)	-88.22% (-93.31 to -79.73)	1.2 (0.8 to 1.7)	0 (0 to 0.1)	-9.91% (-10.48 to -9.35)
Colombia	6 (4 to 9)	4 (2 to 8)	-31.71% (-68.99 to 39.93)	1.1 (0.7 to 1.6)	0.3 (0.1 to 0.5)	-5.35% (-6.13 to -4.56)
Comoros	1 (0 to 3)	1 (0 to 2)	-11.93% (-72.97 to 197.11)	16.3 (4.7 to 39.9)	6.2 (2.6 to 14.2)	-3.65% (-3.95 to -3.35)
Congo	3 (1 to 8)	4 (2 to 8)	17.11% (-58.01 to 274.03)	9.8 (3.4 to 22.7)	4.4 (1.9 to 9)	-2.42% (-3.02 to -1.81)
Costa Rica	0 (0 to 0)	0 (0 to 0)	178.07% (-45.39 to 895.97)	0.1 (0 to 0.3)	0.1 (0 to 0.3)	-0.38% (-0.46 to -0.3)
Côte d'Ivoire	15 (5 to 34)	17 (7 to 36)	13.12% (-64.9 to 261.07)	11.1 (3.9 to 25.5)	4.9 (2 to 10.7)	-2.65% (-2.99 to -2.32)
Cuba	0 (0 to 1)	0 (0 to 1)	81.23% (-87.47 to 750.47)	0.1 (0 to 0.3)	0.1 (0 to 0.2)	-0.53% (-0.67 to -0.4)
Democratic People's Republic of Korea	3 (1 to 7)	4 (1 to 11)	63.6% (-58.85 to 524.35)	0.4 (0.1 to 1)	0.4 (0.1 to 1.1)	0.68% (-0.3 to 1.68)

Location	DALYs in 1990 (95% UI)	DALYs in 2019 (95% UI)	Relative change (%, 95 CI)	Age-standardized DALYs rate in 1990 (95% UI)	Age-standardized DALYs rate in 2019 (95% UI)	EAPC (%, 95 CI)
Democratic Republic of the Congo	126 (44 to 305)	409 (139 to 912)	223.4% (-16.64 to 1121.3)	22.7 (7.8 to 54.8)	32.9 (11.2 to 73.3)	2.89% (1.52 to 4.28)
Djibouti	1 (0 to 3)	2 (1 to 5)	83.13% (-47.71 to 567.46)	22.6 (7.3 to 55.8)	10.6 (3.9 to 25.7)	-2.25% (-2.69 to -1.81)
Dominica	0 (0 to 0)	0 (0 to 0)	1.8% (-58.17 to 164.87)	0.6 (0.3 to 1.1)	0.4 (0.2 to 0.7)	-1.44% (-2.15 to -0.73)
Dominican Republic	1 (0 to 2)	1 (0 to 2)	-10.74% (-72.16 to 168.39)	0.8 (0.3 to 1.9)	0.3 (0.1 to 0.6)	-3.04% (-3.81 to -2.27)
Ecuador	1 (0 to 1)	1 (0 to 1)	-18.12% (-72.42 to 98.97)	0.4 (0.2 to 0.7)	0.1 (0 to 0.3)	-3.86% (-4.41 to -3.31)
Egypt	137 (53 to 288)	9 (4 to 18)	-93.57% (-97.98 to -78.89)	15.1 (5.8 to 31.8)	0.4 (0.2 to 0.9)	-12.86% (-13.57 to -12.15)
El Salvador	1 (0 to 2)	0 (0 to 1)	-67.42% (-91.67 to 14.89)	1.1 (0.4 to 2.5)	0.2 (0.1 to 0.4)	-5.87% (-6.94 to -4.79)
Equatorial Guinea	2 (1 to 6)	0 (0 to 1)	-81.93% (-94.31 to -39.75)	32.6 (10.5 to 82.3)	2.3 (1.1 to 4.3)	-10.03% (-10.83 to -9.22)

Location	DALYs in 1990 (95% UI)	DALYs in 2019 (95% UI)	Relative change (%, 95 CI)	Age-standardized DALYs rate in 1990 (95% UI)	Age-standardized DALYs rate in 2019 (95% UI)	EAPC (%, 95 CI)
Eritrea	23 (7 to 58)	18 (6 to 42)	-21.31% (-77.38 to 213.45)	55.4 (16.4 to 143.2)	17.7 (6.1 to 41.7)	-2.96% (-3.56 to -2.35)
Eswatini	1 (0 to 2)	0 (0 to 1)	-36.76% (-78.09 to 79.62)	7.4 (2.8 to 16.9)	2.5 (1.1 to 5.2)	-2.91% (-3.7 to -2.12)
Ethiopia	1167 (527 to 2224)	353 (183 to 626)	-69.79% (-87.01 to -26.59)	184.9 (83.5 to 352.3)	28.6 (14.9 to 50.8)	-6.69% (-7.52 to -5.85)
Fiji	0 (0 to 1)	0 (0 to 1)	-16.15% (-71.95 to 180.62)	2.8 (1.1 to 6.5)	1.3 (0.6 to 2.5)	-2.86% (-3.33 to -2.38)
Gabon	1 (0 to 3)	1 (0 to 2)	-34.06% (-76.09 to 74.04)	7.5 (2.9 to 15.7)	2.3 (1.1 to 4.5)	-4.26% (-4.77 to -3.74)
Gambia	3 (1 to 8)	5 (2 to 11)	49.57% (-62.31 to 428.25)	32.7 (11.3 to 84.1)	16.7 (5.9 to 39.5)	-2.8% (-3.1 to -2.49)
Georgia	0 (0 to 0)	0 (0 to 0)	-3.97% (-45.83 to 74.13)	0 (0 to 0)	0 (0 to 0)	3.38% (2.37 to 4.4)
Ghana	5 (2 to 9)	7 (3 to 14)	40.87% (-35.76 to 145.98)	2.4 (1.2 to 4.6)	1.2 (0.5 to 2.5)	-2.13% (-2.3 to -1.96)

Location	DALYs in 1990 (95% UI)	DALYs in 2019 (95% UI)	Relative change (%, 95 CI)	Age-standardized DALYs rate in 1990 (95% UI)	Age-standardized DALYs rate in 2019 (95% UI)	EAPC (%, 95 CI)
Global	8617 (6490 to 11464)	4298 (3232 to 5642)	-50.13% (-65.75 to -30.13)	8.2 (6.2 to 10.9)	2 (1.5 to 2.6)	-4.81% (-4.96 to -4.66)
Grenada	0 (0 to 0)	0 (0 to 0)	38.34% (-70.99 to 295.98)	0.2 (0.1 to 0.4)	0.1 (0 to 0.2)	-1.86% (-2.13 to -1.59)
Guatemala	1 (1 to 2)	2 (1 to 3)	26.9% (-33.28 to 136.37)	1.2 (0.7 to 2)	0.6 (0.4 to 0.9)	-3.74% (-4.62 to -2.85)
Guinea	27 (9 to 59)	23 (8 to 56)	-13.04% (-77.29 to 191.62)	31.8 (11.2 to 69.9)	14.6 (5.1 to 35.1)	-2.15% (-2.36 to -1.94)
Guinea-Bissau	3 (1 to 6)	1 (1 to 3)	-41% (-83.06 to 92.97)	19.5 (7 to 48.4)	5.9 (2.5 to 13.5)	-3.86% (-4.1 to -3.62)
Guyana	0 (0 to 0)	0 (0 to 0)	-41.91% (-76.59 to 30.32)	0.6 (0.3 to 1)	0.2 (0.1 to 0.4)	-3.77% (-4.49 to -3.04)
Haiti	8 (2 to 20)	22 (7 to 57)	195.23% (-33.1 to 1299.14)	7.8 (2.1 to 20.3)	9.9 (2.9 to 25.1)	1.73% (1.23 to 2.24)
Honduras	2 (1 to 4)	1 (0 to 2)	-40.33% (-81.7 to 93.94)	2.8 (1 to 7.2)	0.6 (0.2 to 1.3)	-6.1% (-6.57 to -5.63)

Location	DALYs in 1990 (95% UI)	DALYs in 2019 (95% UI)	Relative change (%, 95 CI)	Age-standardized DALYs rate in 1990 (95% UI)	Age-standardized DALYs rate in 2019 (95% UI)	EAPC (%, 95 CI)
India	2879 (1606 to 4640)	1112 (625 to 1869)	-61.38% (-80.48 to -23.9)	20.9 (11.7 to 33.7)	3.5 (2 to 5.9)	-6.18% (-6.67 to -5.69)
Indonesia	30 (18 to 48)	18 (8 to 38)	-39.15% (-71.23 to 1.73)	0.9 (0.5 to 1.4)	0.2 (0.1 to 0.5)	-4.08% (-4.53 to -3.62)
Iran (Islamic Republic of)	27 (17 to 39)	5 (3 to 7)	-82.57% (-90.25 to -69.25)	3.6 (2.3 to 5.1)	0.2 (0.1 to 0.3)	-10.39% (-11.1 to -9.68)
Iraq	3 (1 to 8)	5 (2 to 9)	37.4% (-56.11 to 324.6)	1.6 (0.6 to 3.5)	0.6 (0.3 to 1.3)	-2.81% (-3.54 to -2.07)
Jamaica	0 (0 to 0)	0 (0 to 0)	-46.02% (-92.95 to 50.8)	0.4 (0.2 to 0.6)	0.1 (0 to 0.3)	-4.4% (-5.48 to -3.3)
Jordan	1 (0 to 2)	1 (0 to 1)	-31.86% (-79.12 to 116.01)	1.9 (0.7 to 4.3)	0.3 (0.1 to 0.6)	-7.19% (-7.98 to -6.4)
Kazakhstan	0 (0 to 0)	0 (0 to 0)	46.88% (9.3 to 128.28)	0 (0 to 0)	0 (0 to 0)	2.24% (1.71 to 2.78)
Kenya	46 (27 to 76)	59 (33 to 94)	28.03% (-30.68 to 133.85)	18.5 (10.7 to 30.8)	8 (4.5 to 12.8)	-2.5% (-2.73 to -2.27)
Kiribati	0 (0 to 1)	0 (0 to 1)	23.56% (-71.81 to 422.04)	23 (7.2 to 57.7)	13.3 (4.5 to 32.4)	-2.1% (-2.29 to -1.91)

Location	DALYs in 1990 (95% UI)	DALYs in 2019 (95% UI)	Relative change (%, 95 CI)	Age-standardized DALYs rate in 1990 (95% UI)	Age-standardized DALYs rate in 2019 (95% UI)	EAPC (%, 95 CI)
Kyrgyzstan	0 (0 to 0)	0 (0 to 0)	15.1% (-15.41 to 47.22)	0 (0 to 0)	0 (0 to 0)	-0.83% (-1.26 to -0.4)
Lao People's Democratic Republic	2 (1 to 6)	1 (0 to 1)	-72.34% (-91.68 to -0.46)	3.7 (1.2 to 9.7)	0.5 (0.2 to 1)	-7.86% (-8.47 to -7.24)
Lebanon	0 (0 to 1)	0 (0 to 1)	-17.94% (-75.88 to 107.39)	0.5 (0.2 to 1)	0.2 (0.1 to 0.5)	-3.09% (-3.42 to -2.75)
Lesotho	7 (2 to 16)	4 (1 to 9)	-41.3% (-83.33 to 115.17)	25.2 (8.7 to 58.3)	10.2 (3.5 to 23.5)	-1.54% (-2.27 to -0.81)
Liberia	15 (5 to 35)	17 (6 to 41)	14.59% (-71.52 to 313.73)	56.8 (18.6 to 130.9)	27.7 (9.2 to 65.7)	-3.6% (-4.57 to -2.62)
Libya	0 (0 to 0)	0 (0 to 1)	95.05% (-36.21 to 358.12)	0.3 (0.2 to 0.6)	0.2 (0.1 to 0.4)	-1.8% (-2.12 to -1.48)
Madagascar	41 (14 to 96)	85 (27 to 204)	108.86% (-41 to 647.83)	26.9 (9.3 to 63.3)	20.9 (6.7 to 49.9)	-0.96% (-1.54 to -0.38)
Malawi	56 (19 to 126)	37 (14 to 84)	-33.41% (-80.78 to 141.26)	48.2 (16.2 to 109)	17.2 (6.7 to 39.2)	-3.41% (-3.78 to -3.05)

Location	DALYs in 1990 (95% UI)	DALYs in 2019 (95% UI)	Relative change (%, 95 CI)	Age-standardized DALYs rate in 1990 (95% UI)	Age-standardized DALYs rate in 2019 (95% UI)	EAPC (%, 95 CI)
Malaysia	9 (3 to 22)	3 (1 to 6)	-66.07% (-89.24 to 24.43)	3 (1.1 to 7.3)	0.4 (0.2 to 0.9)	-7.31% (-7.61 to -7.02)
Maldives	0 (0 to 0)	0 (0 to 0)	-83.09% (-95.09 to -35.32)	3.1 (1 to 7.3)	0.2 (0.1 to 0.4)	-9.36% (-10.55 to -8.15)
Mali	110 (34 to 251)	78 (26 to 202)	-28.96% (-80.59 to 201.7)	90.5 (28 to 205.6)	30.2 (9.9 to 77.7)	-3.95% (-4.24 to -3.67)
Marshall Islands	0 (0 to 0)	0 (0 to 0)	56.39% (-54.88 to 472.01)	9.7 (3.1 to 23.9)	5.2 (1.7 to 12.3)	-2.35% (-2.81 to -1.88)
Mauritania	29 (8 to 66)	7 (2 to 15)	-76.33% (-93.62 to -13.1)	107.8 (31.5 to 249.2)	10.9 (3.8 to 24.3)	-8.2% (-8.45 to -7.96)
Mexico	6 (4 to 9)	5 (2 to 9)	-16.43% (-52.68 to 32.89)	0.4 (0.3 to 0.7)	0.1 (0.1 to 0.3)	-4.6% (-5.2 to -3.98)
Micronesia (Federated States of)	0 (0 to 0)	0 (0 to 0)	-22.56% (-81.08 to 202.56)	14.4 (4.3 to 36.6)	5.3 (1.8 to 13)	-3.58% (-3.64 to -3.52)
Mongolia	0 (0 to 1)	0 (0 to 0)	-32.34% (-81.72 to 148.96)	1 (0.3 to 2.4)	0.2 (0.1 to 0.4)	-5.75% (-6.28 to -5.21)
Montenegro	0 (0 to 0)	0 (0 to 0)	7.2% (-2.45 to 15.61)	0 (0 to 0.1)	0 (0 to 0.1)	-0.03% (-0.07 to 0.01)

Location	DALYs in 1990 (95% UI)	DALYs in 2019 (95% UI)	Relative change (%, 95 CI)	Age-standardized DALYs rate in 1990 (95% UI)	Age-standardized DALYs rate in 2019 (95% UI)	EAPC (%, 95 CI)
Morocco	19 (6 to 47)	3 (1 to 7)	-82.29% (-94.81 to -36.48)	4.7 (1.6 to 11.7)	0.4 (0.1 to 0.7)	-8.96% (-9.6 to -8.31)
Mozambique	30 (12 to 67)	18 (8 to 37)	-40.21% (-81.04 to 70.78)	14.8 (5.9 to 33)	4.8 (2.2 to 9.8)	-3.07% (-3.66 to -2.47)
Myanmar	6 (2 to 14)	10 (4 to 20)	64.21% (-43.01 to 420.6)	0.9 (0.3 to 2)	0.6 (0.3 to 1.3)	-1.32% (-2.15 to -0.48)
Namibia	3 (1 to 7)	1 (1 to 4)	-46.97% (-84.19 to 83.28)	14.8 (5.3 to 36.7)	3.3 (1.3 to 8)	-5.09% (-5.63 to -4.55)
Nepal	151 (45 to 353)	29 (11 to 68)	-80.96% (-94.16 to -29.3)	52.5 (15.5 to 122.6)	4.5 (1.8 to 10.5)	-8.24% (-8.65 to -7.83)
Nicaragua	0 (0 to 0)	0 (0 to 1)	105.85% (-50.74 to 413.19)	0.2 (0.1 to 0.5)	0.1 (0 to 0.4)	-1.45% (-1.66 to -1.24)
Niger	36 (11 to 85)	74 (24 to 175)	102.75% (-46.26 to 649.5)	42.3 (13.1 to 99.4)	27.7 (9.1 to 66)	-1.62% (-1.86 to -1.39)
Nigeria	109 (58 to 188)	198 (109 to 339)	82.6% (3.26 to 199.34)	10.3 (5.5 to 17.8)	5.9 (3.2 to 10.1)	-2.42% (-2.95 to -1.88)
North Macedonia	0 (0 to 0)	0 (0 to 0)	-2.07% (-38.85 to 27.17)	0 (0 to 0.1)	0 (0 to 0.1)	-0.77% (-1.08 to -0.47)

Location	DALYs in 1990 (95% UI)	DALYs in 2019 (95% UI)	Relative change (%, 95 CI)	Age-standardized DALYs rate in 1990 (95% UI)	Age-standardized DALYs rate in 2019 (95% UI)	EAPC (%, 95 CI)
Pakistan	1213 (623 to 2416)	510 (243 to 1005)	-58% (-85.11 to 15.48)	76.5 (39.3 to 152.5)	14.6 (7 to 28.8)	-5.78% (-6.6 to -4.94)
Papua New Guinea	13 (4 to 33)	14 (5 to 34)	8.39% (-70.62 to 318.94)	23.7 (7.7 to 58.8)	8.6 (3.2 to 20)	-3.32% (-3.59 to -3.04)
Paraguay	1 (0 to 1)	0 (0 to 1)	-38.78% (-81.52 to 57.82)	0.9 (0.4 to 1.8)	0.2 (0.1 to 0.5)	-5.69% (-6.14 to -5.23)
Peru	5 (2 to 11)	2 (1 to 4)	-62.67% (-89.36 to 28.79)	1.4 (0.5 to 3.4)	0.2 (0.1 to 0.4)	-7.4% (-7.82 to -6.98)
Philippines	20 (13 to 30)	11 (7 to 17)	-44.8% (-71.17 to -2.57)	2.1 (1.3 to 3.2)	0.5 (0.3 to 0.7)	-5.51% (-6.19 to -4.81)
Republic of Moldova	0 (0 to 0)	0 (0 to 0)	-40.3% (-63.32 to -20.95)	0 (0 to 0)	0 (0 to 0)	-1.08% (-1.64 to -0.51)
Russian Federation	3 (2 to 5)	2 (1 to 3)	-39.26% (-55.19 to -25.37)	0.1 (0 to 0.1)	0 (0 to 0.1)	-0.64% (-1.09 to -0.19)
Rwanda	238 (85 to 570)	84 (30 to 203)	-64.67% (-90.63 to 35.44)	249.9 (88.8 to 598.5)	41.4 (14.8 to 100)	-6.83% (-7.62 to -6.03)
Saint Lucia	0 (0 to 0)	0 (0 to 0)	28.09% (-82.18 to 248.22)	0.2 (0.1 to 0.4)	0.1 (0 to 0.3)	-2.35% (-2.98 to -1.71)

Location	DALYs in 1990 (95% UI)	DALYs in 2019 (95% UI)	Relative change (%, 95 CI)	Age-standardized DALYs rate in 1990 (95% UI)	Age-standardized DALYs rate in 2019 (95% UI)	EAPC (%, 95 CI)
Saint Vincent and the Grenadines	0 (0 to 0)	0 (0 to 0)	84.09% (-67.33 to 548.14)	0.1 (0 to 0.3)	0.1 (0 to 0.3)	-0.51% (-0.66 to -0.36)
Samoa	0 (0 to 0)	0 (0 to 0)	-13.94% (-66.06 to 108.59)	1.7 (0.7 to 4)	0.9 (0.4 to 1.6)	-3.28% (-3.68 to -2.88)
Senegal	16 (6 to 41)	17 (6 to 37)	1.29% (-71.2 to 251.91)	17.1 (6 to 43.1)	7.1 (2.5 to 15.8)	-2.57% (-3.03 to -2.11)
Serbia	0 (0 to 0)	0 (0 to 0)	-13.85% (-20.95 to -6.4)	0 (0 to 0.1)	0 (0 to 0.1)	0.08% (-0.02 to 0.17)
Sierra Leone	8 (3 to 18)	10 (4 to 22)	28.02% (-60.75 to 332.23)	15.8 (5.7 to 35.7)	9.6 (3.8 to 20.9)	-1.55% (-2.26 to -0.83)
Solomon Islands	1 (0 to 2)	2 (1 to 5)	131.44% (-44.93 to 846.96)	20.2 (5.9 to 53.7)	18.8 (5.7 to 46.1)	0.2% (-0.41 to 0.82)
Somalia	104 (31 to 244)	210 (62 to 502)	102.11% (-49.69 to 770.27)	107.1 (31.4 to 251.2)	111.9 (32.8 to 267)	0.46% (0.14 to 0.78)
South Africa	15 (9 to 24)	16 (8 to 26)	2.5% (-39.94 to 63.87)	2.5 (1.5 to 4)	1.1 (0.6 to 1.9)	-0.8% (-1.57 to -0.02)

Location	DALYs in 1990 (95% UI)	DALYs in 2019 (95% UI)	Relative change (%, 95 CI)	Age-standardized DALYs rate in 1990 (95% UI)	Age-standardized DALYs rate in 2019 (95% UI)	EAPC (%, 95 CI)
South Sudan	13 (4 to 32)	22 (7 to 53)	71.85% (-50.92 to 518.33)	22.4 (7.2 to 56)	15.7 (5.2 to 38.1)	-1.27% (-1.36 to -1.18)
Sri Lanka	1 (0 to 2)	1 (0 to 2)	16.7% (-73.9 to 222.71)	0.2 (0.1 to 0.5)	0.1 (0 to 0.3)	-1.98% (-2.06 to -1.89)
Sudan	94 (31 to 226)	98 (32 to 241)	4.99% (-72.46 to 325.51)	35.9 (11.8 to 86.5)	16.5 (5.4 to 40.5)	-2.18% (-3.62 to -0.71)
Suriname	0 (0 to 0)	0 (0 to 0)	32.08% (-57.7 to 249.29)	0.3 (0.1 to 0.7)	0.2 (0.1 to 0.4)	-1.88% (-2.38 to -1.37)
Syrian Arab Republic	2 (1 to 3)	2 (1 to 3)	-12.49% (-66.27 to 132.73)	1.2 (0.5 to 2.2)	0.4 (0.2 to 0.7)	-5.02% (-5.47 to -4.57)
Tajikistan	0 (0 to 0)	0 (0 to 0)	-51.25% (-79.83 to 22.44)	0.2 (0.1 to 0.4)	0 (0 to 0.1)	-6.37% (-7.04 to -5.69)
Thailand	2 (1 to 4)	3 (1 to 9)	61.6% (-58.38 to 421.69)	0.2 (0.1 to 0.3)	0.1 (0 to 0.3)	-0.99% (-1.23 to -0.75)
Timor-Leste	2 (1 to 5)	1 (0 to 2)	-48.23% (-87.49 to 127.69)	17.1 (4.8 to 44.9)	4.8 (1.6 to 11.6)	-5.55% (-6.51 to -4.57)

Location	DALYs in 1990 (95% UI)	DALYs in 2019 (95% UI)	Relative change (%, 95 CI)	Age-standardized DALYs rate in 1990 (95% UI)	Age-standardized DALYs rate in 2019 (95% UI)	EAPC (%, 95 CI)
Togo	7 (2 to 17)	9 (3 to 19)	21.78% (-63.39 to 333.26)	17.1 (5.6 to 40.7)	6.6 (2.6 to 14.5)	-2.87% (-3.25 to -2.49)
Tonga	0 (0 to 0)	0 (0 to 0)	-62.64% (-87.87 to 15.39)	8 (2.8 to 18.8)	2.3 (1 to 4.9)	-4.91% (-5.25 to -4.58)
Tunisia	1 (0 to 2)	1 (0 to 1)	-15.09% (-74.84 to 136.51)	0.6 (0.2 to 1.2)	0.2 (0.1 to 0.4)	-3.09% (-3.45 to -2.72)
Turkey	9 (4 to 20)	5 (2 to 12)	-39.67% (-80.61 to 78.3)	0.9 (0.3 to 1.9)	0.2 (0.1 to 0.5)	-4.74% (-5.11 to -4.38)
Turkmenistan	0 (0 to 0)	0 (0 to 0)	-9.04% (-59.33 to 84.56)	0.2 (0.1 to 0.4)	0.1 (0 to 0.2)	-3.15% (-3.44 to -2.85)
Tuvalu	0 (0 to 0)	0 (0 to 0)	-68.04% (-91.58 to 29.47)	13.3 (4.2 to 34.3)	3.1 (1.1 to 7.5)	-4.78% (-4.84 to -4.72)
Uganda	36 (13 to 83)	50 (22 to 100)	37.78% (-52.45 to 351.57)	18.3 (6.8 to 42.1)	10.2 (4.4 to 20.4)	-1.98% (-2.29 to -1.67)
Ukraine	0 (0 to 1)	0 (0 to 1)	-19.17% (-27.86 to -12.77)	0 (0 to 0)	0 (0 to 0)	0.29% (0.15 to 0.43)

Location	DALYs in 1990 (95% UI)	DALYs in 2019 (95% UI)	Relative change (%, 95 CI)	Age-standardized DALYs rate in 1990 (95% UI)	Age-standardized DALYs rate in 2019 (95% UI)	EAPC (%, 95 CI)
United Republic of Tanzania	96 (34 to 215)	94 (37 to 205)	-2.42% (-71.93 to 291.09)	28.6 (10.1 to 64)	11.4 (4.5 to 25)	-1.97% (-2.46 to -1.47)
Uzbekistan	0 (0 to 1)	0 (0 to 0)	-51.4% (-74.24 to -5.27)	0.1 (0.1 to 0.2)	0 (0 to 0)	-6.06% (-6.89 to -5.22)
Vanuatu	0 (0 to 0)	0 (0 to 0)	65.26% (-53.45 to 509.52)	6.6 (2.3 to 16.2)	4 (1.4 to 9.3)	-2.53% (-2.93 to -2.12)
Venezuela (Bolivarian Republic of)	3 (2 to 4)	3 (2 to 5)	8.31% (-44.86 to 113.11)	1 (0.6 to 1.4)	0.4 (0.2 to 0.6)	-3.57% (-4.15 to -2.99)
Viet Nam	2 (0 to 4)	3 (1 to 9)	91.93% (-63.75 to 561.77)	0.2 (0 to 0.4)	0.1 (0 to 0.3)	-1.08% (-1.24 to -0.93)
Yemen	10 (3 to 25)	16 (5 to 38)	53.46% (-61.9 to 561.02)	7.3 (2.2 to 18.1)	3.9 (1.3 to 9.4)	-3.37% (-3.74 to -3)
Zambia	11 (4 to 24)	8 (4 to 15)	-28.98% (-75.54 to 108.98)	11.5 (4.4 to 25.4)	3.6 (1.7 to 6.9)	-4.39% (-5 to -3.78)

Location	DALYs in 1990 (95% UI)	DALYs in 2019 (95% UI)	Relative change (%, 95 CI)	Age-standardized DALYs rate in 1990 (95% UI)	Age-standardized DALYs rate in 2019 (95% UI)	EAPC (%, 95 CI)
Zimbabwe	18 (6 to 40)	28 (10 to 63)	54.43% (-57.66 to 449.27)	14.1 (5 to 31.9)	12.6 (4.6 to 28.8)	2.71% (1.31 to 4.12)

CI – confidence interval, DALYs – disability-adjusted life years (per 100,000 population), EAPC – estimated annual percentage change, MSMI – maternal sepsis and other maternal infections, UI – uncertain interval..

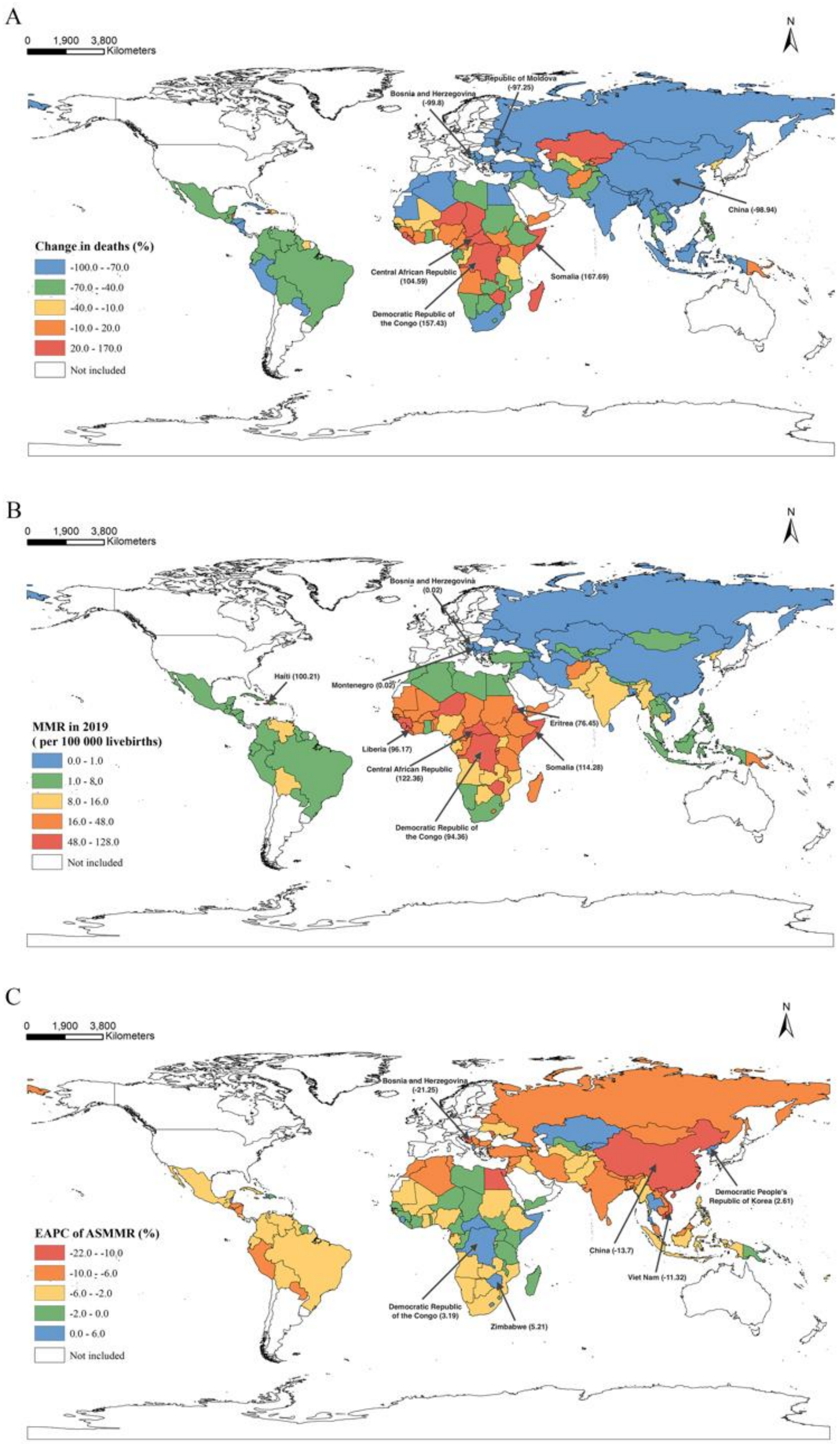

Figure S1. National trends in the deaths of MSMI in 131 LIMCs.

Notes: The percentage of relative percent change in deaths of MSMI between 1990 and 2019 (A), age-standardized maternal mortality ratios (ASMMRs) of MSMI in 2019 (B), and estimated annual percentage changes (EAPCs) of ASMMRs of MSMI from 1990 to 2019 (C) are reported. LIMCs – low- and middle-income countries and territories, MSMI – maternal sepsis and other maternal infections.

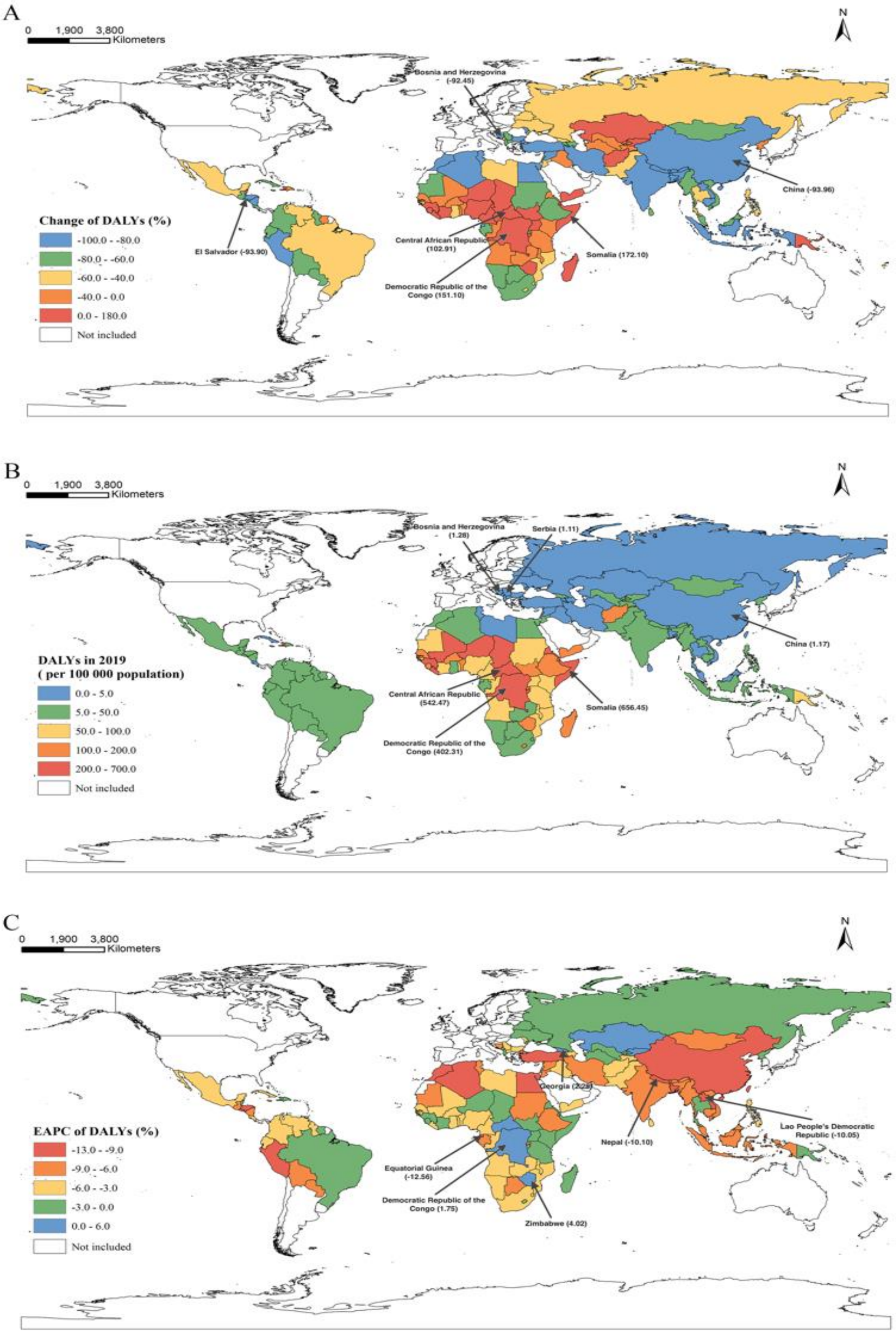

Figure S2. National trends in the DALYs of MSMI in 131 LIMCs.

Notes: The percentage of relative percent change in DALYs of MSMI between 1990 and 2019 (A), age-standardized DALYs rates of MSMI in 2019 (B), and estimated annual percentage changes (EAPCs) of age-standardized DALYs rates of MSMI from 1990 to 2019 (C) are reported. DALYs – disability-adjusted life years (per 100,000 population), LIMCs – low- and middle-income countries and territories, MSMI – maternal sepsis and other maternal infections.

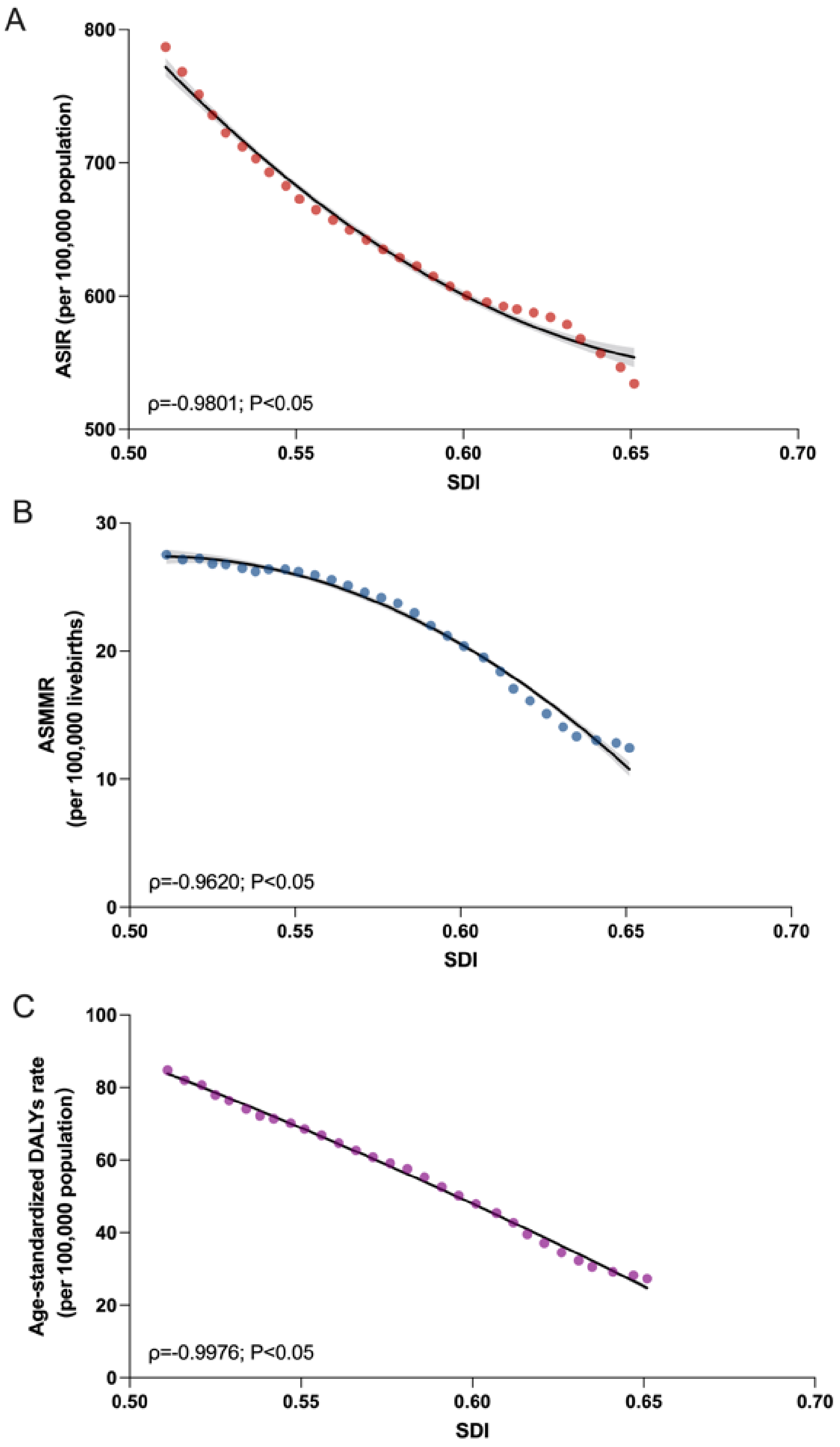

Figure S3. ASIR (A), ASMMR (B), and age-standardized DALY rates (C) for MSMSI globally by SDI, 1990–2019

Notes: Pearson correlation analyses were employed. ASIR – age-standardized incidence rate (per 100,000 population), ASMMR – age-standardized maternal mortality ratio (per 100,000 livebirths), DALYs – disability-adjusted life years (per

100,000 population), MSMI – maternal sepsis and other maternal infections, SDI – Socio-demographic Index.

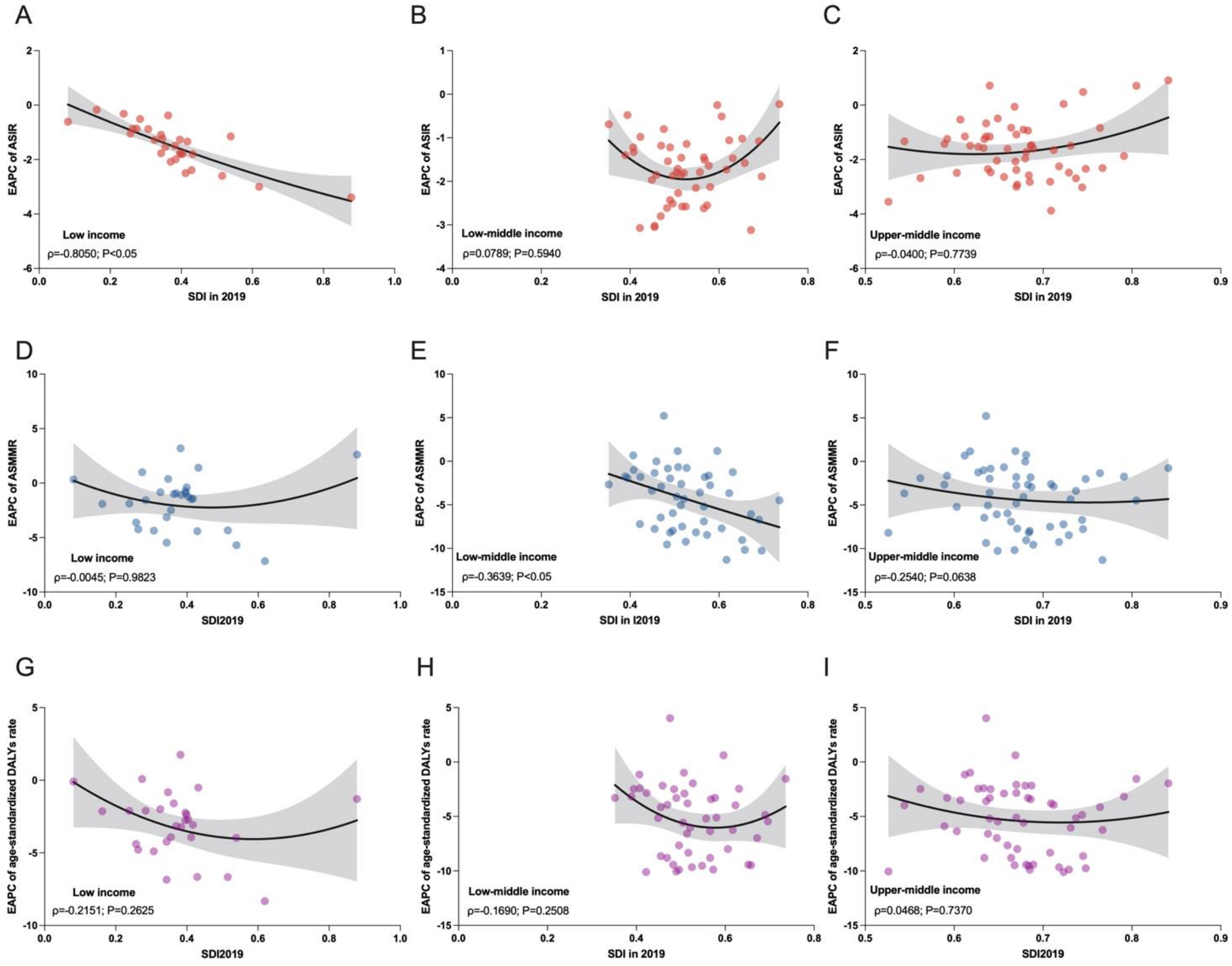

Figure S4. The correlation between the EAPC of ASIR/ASMMR/ age-standardized DALY rates of MSMI and SDI (in 2019) in LICs, LMCs, and UMCs.

Notes: Spearman correlation analyses were employed. ASIR – age-standardized incidence rate (per 100,000 population), ASMMR – age-standardized maternal mortality ratio (per 100,000 livebirths), DALYs – disability-adjusted life years (per 100,000 population), LICs – low-income countries and territories, LMCs – lower-middle-income countries and territories, UMCs – Upper-middle-income countries and territories, MSMI – maternal sepsis and other maternal infections, SDI – socio-demographic index.

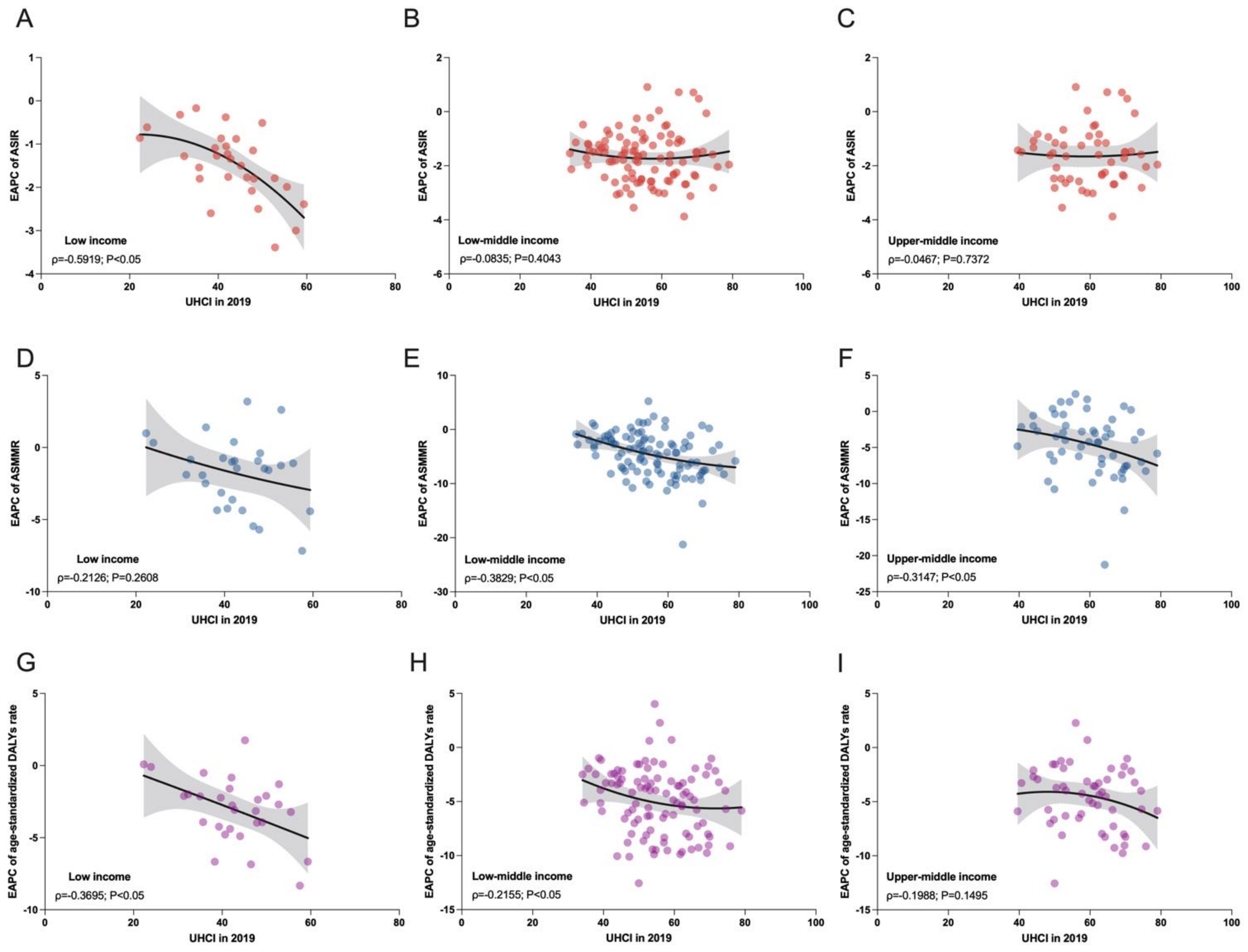

Figure S5. The correlation between the EAPC of ASIR/ASMMR/ age-standardized DALY rates of MSMI and UHCI (in 2019) in LICs, LMCs, and UMCs.

Notes: Spearman correlation analyses were employed. ASIR – age-standardized incidence rate (per 100,000 population), ASMMR – age-standardized maternal mortality ratio (per 100,000 livebirths), DALYs – disability-adjusted life years (per 100,000 population), LICs – low-income countries and territories, LMCs – lower-middle-income countries and territories, UMCs – Upper-middle-income countries and territories, MSMI – maternal sepsis and other maternal infections, UHCI – universal health coverage effective coverage index.
